# Cooperation of the BTB-Zinc finger protein, Abrupt, with cytoskeletal regulators in *Drosophila* epithelial tumorigenesis

**DOI:** 10.1242/bio.012815

**Published:** 2015-07-17

**Authors:** Nezaket Turkel, Marta Portela, Carole Poon, Jason Li, Anthony M. Brumby, Helena E. Richardson

**Affiliations:** 1 Cell Cycle and Development Laboratory, Peter MacCallum Cancer Centre, Melbourne, Victoria 3002, Australia; 2 Bioinformatics Core Facility, Peter MacCallum Cancer Centre, Melbourne, Victoria 3002, Australia; 3 Sir Peter MacCallum Department of Oncology, Department of Anatomy and Neuroscience, Department of Biochemistry and Molecular Biology, University of Melbourne, Melbourne, Victoria 3010, Australia; 4 School of Molecular Sciences, La Trobe University, Melbourne, Victoria 3086, Australia

**Keywords:** *Drosophila*, Eye-antennal disc, Apico-basal cell polarity, Actin cytoskeletal regulators, BTB-ZF, Abrupt, RhoGEF2, Rac1, Src, Scribble

## Abstract

The deregulation of cell polarity or cytoskeletal regulators is a common occurrence in human epithelial cancers. Moreover, there is accumulating evidence in human epithelial cancer that BTB-ZF genes, such as *Bcl6* and *ZBTB7A*, are oncogenic. From our previous studies in the vinegar fly, *Drosophila melanogaster*, we have identified a cooperative interaction between a mutation in the apico-basal cell polarity regulator Scribble (Scrib) and overexpression of the BTB-ZF protein Abrupt (Ab). Herein, we show that co-expression of *ab* with actin cytoskeletal regulators, *RhoGEF2* or *Src64B,* in the developing eye-antennal epithelial tissue results in the formation of overgrown amorphous tumours, whereas *ab* and *DRac1* co-expression leads to non-cell autonomous overgrowth. Together with *ab*, these genes affect the expression of differentiation genes, resulting in tumours locked in a progenitor cell fate. Finally, we show that the expression of two mammalian genes related to *ab*, *Bcl6* and *ZBTB7A*, which are oncogenes in mammalian epithelial cancers, significantly correlate with the upregulation of cytoskeletal genes or downregulation of apico-basal cell polarity neoplastic tumour suppressor genes in colorectal, lung and other human epithelial cancers. Altogether, this analysis has revealed that upregulation of cytoskeletal regulators cooperate with Abrupt in *Drosophila* epithelial tumorigenesis, and that high expression of human BTB-ZF genes, *Bcl6* and *ZBTB7A,* shows significant correlations with cytoskeletal and cell polarity gene expression in specific epithelial tumour types. This highlights the need for further investigation of the cooperation between these genes in mammalian systems.

## INTRODUCTION

Cancer is a cooperative process involving many mutations that lead to the deregulation of the normal controls that regulate cell proliferation, survival, differentiation and migration, amongst other processes (Hanahan and Weinberg, 2011). Understanding the molecular events that occur during cooperative tumorigenesis is critical in order to develop therapeutics to combat cancer. The model organism, *Drosophila melanogaster* (vinegar fly), has proven to be an excellent model for the discovery of new tumorigenic genes and the dissection of their roles in tumour progression, and has proven relevance to human cancer (Brumby and Richardson, 2005; Cheng et al., 2013; Gonzalez, 2013; Rudrapatna et al., 2012; Stefanatos and Vidal, 2011).

Recently, the disruption of apical-basal cell polarity, which affects cell adhesion and signalling pathways and leads to an epithelial to mesenchymal transition (EMT), has been realized as a new hallmark of cancer (Elsum et al., 2012; Hanahan and Weinberg, 2011; Humbert et al., 2008). Central to cell polarity regulation are the Scribble module (Scribble (Scrib), Dlg and Lgl), the Crumbs module (Crumbs, Pals and Patj) and the Par module (Par6, Par3 and aPKC), which undergo positive or negative interactions in the establishment and maintenance of the apical and basolateral domains of an epithelial cell. Whole organism or tissue-specific depletion of genes in the Scribble module lead to a loss of cell polarity and aberrant signalling, leading to the formation of neoplastic tumour in *Drosophila* epithelial tissues (Elsum et al., 2012; Humbert et al., 2008). However, when *scrib, dlg* or *lgl* are mutated in patches of cells within the developing eye-antennal tissue, despite deregulation of signalling pathways and cell proliferation, tissue overgrowth does not ensue due to cell differentiation and Jun N-terminal Kinase (JNK)-mediated apoptosis (Brumby and Richardson, 2003; Doggett et al., 2011; Grzeschik et al., 2007, 2010; Igaki et al., 2006; Uhlirova and Bohmann, 2006; Uhlirova et al., 2005). In investigating cooperation between polarity loss and oncogenic pathways in epithelial tumorigenesis, we discovered that activation of the small GTPase, Ras (*Ras85D^V12^*, referred to as *Ras^ACT^* herein) or activated Notch (*Notch^ICD^*, referred to as *Notch^ACT^* herein) cooperated with *scrib* loss-of-function to form massive invasive tumours (Brumby and Richardson, 2003). Subsequent analysis showed that cooperation depended upon activation of the JNK and downregulation of the Hippo negative tissue growth control pathways, thereby promoting tumour growth, inhibiting differentiation and promoting an invasive phenotype (Doggett et al., 2011; Igaki et al., 2006; Leong et al., 2009; Uhlirova and Bohmann, 2006; Uhlirova et al., 2005). This mechanism is conserved in mammalian cells and mouse models, where depletion or knockout of *scrib* leads to hyperplasia, and additional expression of the Ras oncogene (*Ha-Ras^V12^*) cooperates with *scrib* loss-of-function to promote tumorigenesis (Dow et al., 2008; Elsum et al., 2013; Godde et al., 2014; Pearson et al., 2011). Moreover, similar to that observed in *Drosophila*, the expression of JNK is able to cooperate with *Ha-Ras^V12^* to promote invasive growth in 3D matrigel cultures (Brumby et al., 2011).

To further investigate cooperative tumorigenesis, we carried out a screen for genes that when over-expressed in eye-antennal disc clones act similarly to *Ras^ACT^* or *Notch^ACT^* in cooperation with *scrib* loss-of-function (Turkel et al., 2013). In this screen, we identified *abrupt* (*ab*), which in cooperation with *scrib* loss-of-function promotes the retention of a progenitor-like cell state by blocking expression of differentiation genes, as well as promoting tumour growth and invasion. Abrupt encodes a Broad-Complex, Tramtrack, Bric-a-brac domain (BTB)-zinc-finger (ZF) transcription factor with roles in neuromuscular junction and dendrite morphogenesis, ovarian border cell migration and imaginal disc epithelial development (Grieder et al., 2007; Hattori et al., 2013; Hu et al., 1995; Jang et al., 2009). BTB-ZF transcription factors are a large family of proteins, with 47 human members, many of which have been shown to be associated with cancer (Costoya, 2007; Kelly and Daniel, 2006). The most well known of the BTB-ZF mammalian family members are Bcl6 and ZBTB7 (LRF/Pokemon), which function as proto-oncogenes in lymphomas, leukaemias and solid cancers (Hatzi and Melnick, 2014; Maeda et al., 2005). In solid cancers, Bcl6 is upregulated in breast, colorectal and squamous head and neck epithelial cancers, and contributes to their growth and progression (Sena et al., 2014; Walker et al., 2014; Worsham et al., 2012; Wu et al., 2014). ZBTB7A is upregulated in colorectal, bladder, breast, prostate, non-small cell lung cancer and liver cancers and reducing its expression blocks tumour development (Aggarwal et al., 2010, 2011; Guo et al., 2014; Jeon et al., 2008; Liu et al., 2012; Qu et al., 2010; Zhang et al., 2013; Zhao et al., 2013, 2008).

In a *Drosophila* genetic screen for Ras-cooperating genes (using *ey>Ras^ACT^*, where expression of *Ras^ACT^* is driven via the *eyeless* promoter throughout the developing eye), we identified the actin cytoskeletal regulatory genes, *RhoGEF2* and *DRac1* (Brumby et al., 2011)*.* These genes enhanced the *ey>Ras^ACT^* hyperplastic adult eye phenotype and also resulted in morphological and differentiation defects (Brumby et al., 2011). Furthermore, *RhoGEF2* and *DRac1* showed neoplastic growth in cooperation with *Ras^ACT^* in a clonal context in the eye-antennal disc (Brumby et al., 2011). DRac1 (*Drosophila* Rac1) is a member of Rho/Rac/Cdc42 small-GTPase superfamily, key regulators of the actin cytoskeleton (Jaffe and Hall, 2005; Szczepanowska, 2009), and is involved in morphological cell shape changes during *Drosophila* development (Harden et al., 1995; Settleman, 1999; Van Aelst and D'Souza-Schorey, 1997). Indeed, constitutive activation of *Rac1* during tube morphogenesis of the *Drosophila* salivary gland causes changes in epithelial cell morphology, resembling an epithelial to mesenchymal transition (EMT) by mislocalization or loss of expression of the apical polarity regulators, Crumbs and aPKC, and the adherens junction proteins E-cadherin and β-catenin (Pirraglia et al., 2006; Pirraglia and Myat, 2010). It is therefore likely that these downstream effects of Rac1 also contribute to its cooperative effects with *Ras^ACT^* in tumorigenesis in the eye-antennal disc (Brumby et al., 2011).

RhoGEF2 is a guanine nucleotide exchange factor (GEF) (Schmidt and Hall, 2002) that acts via activating the small GTPase, Rho1, in morphological cell shape changes during *Drosophila* development (Barrett et al., 1997; Häcker and Perrimon, 1998; Mulinari et al., 2008; Nikolaidou and Barrett, 2004; Padash Barmchi et al., 2005; Rogers et al., 2004). Consistent with RhoGEF2 functioning via Rho1, we also found that an activated allele of Rho1 (*Rho1^V14^*) was also a *Ras^ACT^* cooperating oncogene in epithelial tumorigenesis (Brumby et al., 2011). RhoGEF2 cooperates with *Ras^ACT^* in tumorigenesis by activating the Rho1-Rok-MyoII-JNK pathway (Khoo et al., 2013). Interestingly, MyoII activity (pMRLC) is increased in *scrib^−^ Ras^ACT^* eye-antennal disc clones and contributes to *scrib Ras^ACT^* tumorigenesis (Kulshammer and Uhlirova, 2013), as does JNK activation (Igaki et al., 2006; Leong et al., 2009; Uhlirova and Bohmann, 2006).

Furthermore, in this genetic screen, we identified another cytoskeletal regulator, *Src42A,* a *Drosophila* homolog of the Src tyrosine kinase (Thomas and Brugge, 1997), but were unable to confirm its cooperative interaction with *Ras^ACT^* with an independent transgene (Brumby et al., 2011). However, we found that overexpression of the second *Drosophila* Src family member, *Src64B,* using a transgenic line (Dodson et al., 1998), showed strong cooperation with *Ras^ACT^* when expressed globally in the developing eye or in eye-antennal disc MARCM clones (C.P., A.B., H.R., unpublished data). *Src64B* also functions in regulation of the actin cytoskeleton and cell shape changes during development in *Drosophila* (Dodson et al., 1998; Guarnieri et al., 1998; Kelso et al., 2002; O'Reilly et al., 2006; Roulier et al., 1998; Strong and Thomas, 2011; Takahashi et al., 1996). Depending on the context, upregulation of *Src64B* or *Src42A* activity (via overexpression of the Src genes or Csk downregulation) can lead to either increased proliferation, or apoptosis and invasion (Pedraza et al., 2004; Read et al., 2004; Vidal et al., 2006, 2007). Recent studies have also shown that overexpression of *Src42A* or *Src64B* in *Drosophila* adult intestinal progenitor cells results in progenitor cell over-proliferation (Cordero et al., 2014; Kohlmaier et al., 2014). Furthermore, in the developing wing epithelium blocking apoptosis in tissues expressing *Src64B* results in overgrowth (Fernández et al., 2014), and in the eye-antennal epithelium *Src64B* or *Src42A* upregulation (or downregulation of the Src negative regulator, Csk) cooperates with *Ras^ACT^* to result in neoplastic tumour formation (Enomoto and Igaki, 2013; Vidal et al., 2010, 2007).

Since *RhoGEF2, DRac1* or *Src* are cooperating oncogenes with *Ras^ACT^*, and *ab* overexpression phenocopies *Ras^ACT^* or *Notch^ACT^* in cooperative tumorigenesis with *scrib* loss-of-function (Turkel et al., 2013), we sought to determine whether *ab* could also cooperate with *RhoGEF2, DRac1* or *Src64B* in tumorigenesis. Herein, we describe the effect of co-expression of *ab* with *RhoGEF2, DRac1* or *Src64B* in the developing eye-antennal epithelium. We show that co-expression of *ab* with *RhoGEF2* or *Src64B* results in neoplastic tumour formation, whereas *ab* and *DRac1* co-expression leads to non-cell autonomous overgrowth. We show that together with *ab* these genes affect the expression of differentiation genes. Finally, we investigate whether the expression of two mammalian genes related to *ab*, *Bcl-6* and *ZBTB7A*, which are oncogenic in mammalian cancer, are correlated with the upregulation of cytoskeletal genes or downregulation of apico-basal cell polarity neoplastic tumour suppressor genes in human epithelial cancers.

## RESULTS

### Cooperation of *abrupt* with *RhoGEF2*

**Fig. 1.**
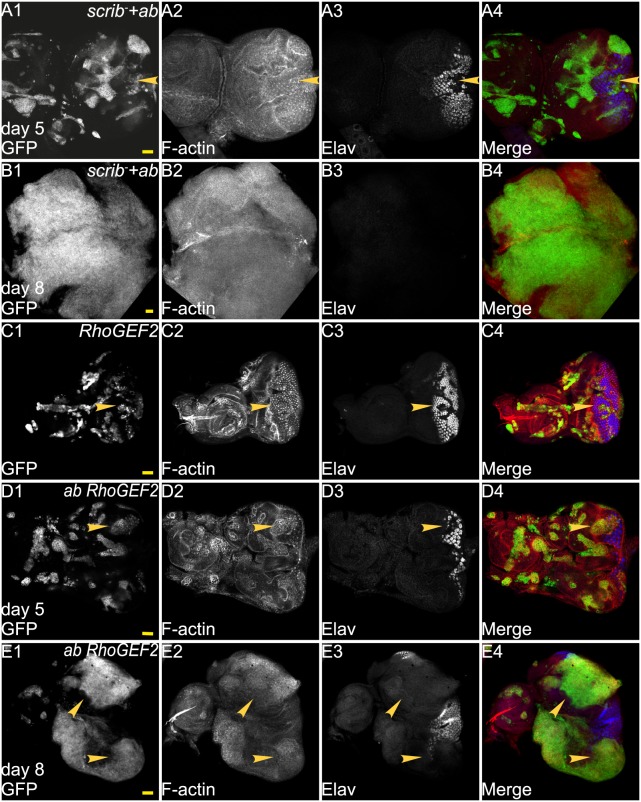
***RhoGEF2* cooperates with *ab* to form large tumours.** Confocal planar images of mosaic larval eye-antennal discs stained for F-actin (with Phalloidin, grey or red in merge) and Elav (grey or blue in merge); mutant clones are GFP^+^ and wild-type tissue is GFP^−^ (grey or green in merge). Eye-antennal discs are orientated with posterior to the left in this and all other figures. (A) *ab scrib^1^* mosaic eye-antennal disc at day 5 AED. (B) *ab scrib^1^* mosaic eye-antennal disc at day 8 AED. (C) *RhoGEF2* mosaic eye-antennal disc at day 5 AED. (D) *ab RhoGEF2* mosaic eye-antennal disc at day 5 AED. (E) *ab RhoGEF2* mosaic eye-antennal disc at day 8 AED. Arrowheads point to patches of mutant tissue showing differentiation defects. Genotypes: (A,B) *ey-FLP, UAS-GFP;; UAS-ab^55^, FRT82B, scrib^1^/tubGAL4; FRT82B, tubGAL80*. (C) *ey-FLP, UAS-GFP; UAS-RhoGEF2*; *FRT82B/tubGAL4; FRT82B, tubGAL80*. (D,E) *ey-FLP, UAS-GFP; UAS-RhoGEF2*; *UAS-ab^55^, FRT82B/tubGAL4; FRT82B, tubGAL80*. Scale bars=50 μM.

To determine if *ab* cooperates with Ras-cooperative oncogene, *RhoGEF2,* to drive tumorigenesis, we generated clones expressing *ab* and *RhoGEF2* using the MARCM system (Lee and Luo, 1999), and compared tumour development to *scrib^−^ ab*-expressing clones in the *Drosophila* developing eye-antennal epithelium (Fig. 1). Our previous studies have shown that the overexpression of *ab* in otherwise wild-type eye disc clones promoted antennal disc overgrowth, but did not block photoreceptor differentiation. Mutation of *scrib* alone in clones results in cell morphology changes and disorganisation, but does not dramatically affect differentiation as revealed by Elav staining or lead to tissue overgrowth and larvae enter pupariation normally at day 5/6 after egg deposition (AED) (Brumby and Richardson, 2003; Turkel et al., 2013). However, *scrib^−^ ab*-expressing clones (marked by GFP) overgrow at the expense of the surrounding normal tissue (GFP negative) over an extended larval period and form massive tumours (Fig. 1A,B), which fuse with the surrounding tissue and invade into the brain (Turkel et al., 2013), similar to that observed for *scrib^−^ Ras^ACT^* tumours (Brumby and Richardson, 2003). *scrib^−^ ab*-expressing clones showed cell morphology defects, as revealed by F-actin staining (Fig. 1A2,B2), and an inhibition of photoreceptor cell differentiation in the eye epithelium, as revealed by Elav staining (arrowheads, Fig. 1A1-A4).

*RhoGEF2* expression in mosaic disc produced small clones with increased F-actin levels, and cell morphology and differentiation defects (arrowheads, Fig. 1C1,C3,C4) (Brumby et al., 2011; Khoo et al., 2013). At day 5/6 AED *ab RhoGEF2* co-expressing clones were smaller than the surrounding wild-type clones (Fig. 1D1,D4) and accumulated F-actin (Fig. 1D2). *ab RhoGEF2* mosaic discs also showed non-cell autonomous effects, as the surrounding wild-type tissue exhibited folding and distortion around the clonal tissue at day 5 (Fig. 1D2). At day 8/9 AED, *ab RhoGEF2* eye disc clones were overgrown relative to wild-type tissue (Fig. 1E), although folded wild-type tissue was present around clonal tissue. However, antennal disc clones did not overgrow and remained a similar size as day 5 clones. Differentiation as marked by Elav was reduced in eye disc clones throughout larval development (arrowheads, Fig. 1D1-D4,E1-E4). The effect of *ab RhoGEF2* cooperation led to a failure in pupation and the formation of giant larvae (not shown), similar to *RhoGEF2 Ras^ACT^* cooperation (Khoo et al., 2013). However, in comparison to *scrib^−^ ab* tumours, which exhibit fusion of the two eye-antennal discs that is associated with an invasive phenotype (Turkel et al., 2013), *ab RhoGEF2* did not show strong invasive properties, since the two eye-antennal discs did not fuse together (data not shown). Indeed, the cooperative tumorigenic effect of *ab RhoGEF2* was most similar to *Ras^ACT^ RhoGEF2* cooperation, with the exception of the effect on the antennal disc (Brumby et al., 2011; Khoo et al., 2013). Taken together, these data show that *RhoGEF2* is capable of cooperating with *ab* to produce overgrown, undifferentiated and amorphous tumours.

### Cooperation of *abrupt* with *Src64B*

**Fig. 2.**
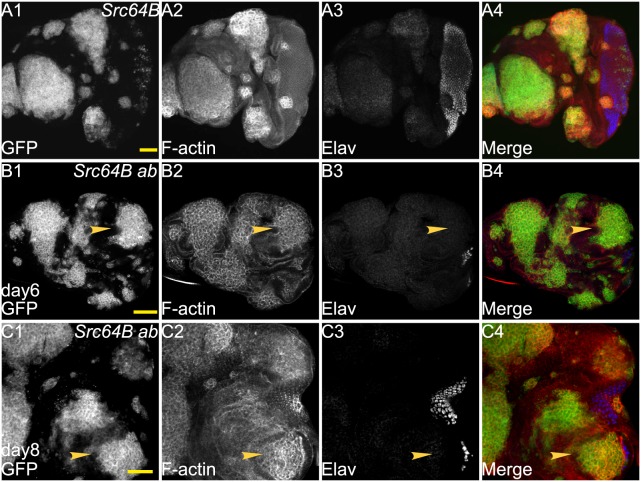
***Src64B* cooperates with *ab* to form large tumours.** Confocal planar images of mosaic larval eye-antennal discs stained for F-actin (with Phalloidin, grey or red in merge) and Elav (grey or blue in merge); mutant clones are GFP^+^ and wild-type tissue is GFP^−^ (grey or green in merge). (A) *Src64B* mosaic eye-antennal disc at day 5 AED. (B) *ab Src64B* mosaic eye-antennal disc at day 5 AED. (C) *ab Src64B* mosaic eye-antennal disc at day 8 AED. Arrowheads point to patches of mutant tissue showing differentiation defects. Genotypes: (A) *ey-FLP, UAS-GFP; UAS-Src64B; FRT82B/tubGAL4; FRT82B, tubGAL80*. (B,C) *ey-FLP, UAS-GFP; UAS-Src64B*; *UAS-ab^55^, FRT82B/tubGAL4; FRT82B, tubGAL80*. Scale bars=50 μM.

Since *Src64B* can cooperate with *Ras^ACT^* (see introduction), we wished to determine if *ab* also cooperates with *Src64B*. When expressed alone, *Src64B* resulted in large clones in the antennal and the anterior portion of the eye disc, which showed high levels of F-actin accumulation (Fig. 2A2). Clones in the posterior differentiated region of the eye disc proper were very small and did not noticeably affect differentiation, although larger clones were observed in the overlying peripodial layer leading to the displacement of the underlying differentiated tissue (Fig. 2A1,A3,A4). *Src64B*-expressing mosaic larvae pupated normally, but were delayed in development and eclosed 1–2 days after their control counterparts (not shown). Co-expression of *Src64B* and *ab* resulted in large clones in the antenna and the eye discs, including the posterior region of the eye disc at day 5/6 AED (Fig. 2B1), however these were not significantly overgrown relative to the wild-type tissue. However at day 8/9 AED, *Src64B ab* eye disc clones were clearly overgrown relative to wild-type tissue. *Src64B ab* co-expressing clones had rounded-edges with high levels of F-actin at day 5/6 and day 8 AED (Fig. 2B2,C2). Differentiation, as revealed by Elav staining, was abolished in clones in the posterior region of the eye disc (arrowheads, Fig. 2B1-B4,C1-C4). The overall size of Day 8/9 *Src64B ab* mosaic eye-antennal discs were overgrown relative to wild-type mosaic eye-antennal discs, however there was slightly more wild-type tissue remaining at day 8/9 AED compared to *scrib*^−^ *ab* mosaic discs (compare Fig. 2C with Fig. 1B). *Src64B ab* cooperation led to the formation of giant larvae and a failure of pupation (not shown), however they did not result in the fusion of the two eye-antennal discs (not shown), as occurs with *scrib^−^ ab* tumours. Altogether, these data indicates that *ab* cooperates with *Src64B* to promote overgrown, undifferentiated and amorphous tumours.

### Cooperation of *abrupt* with *DRac1*

**Fig. 3.**
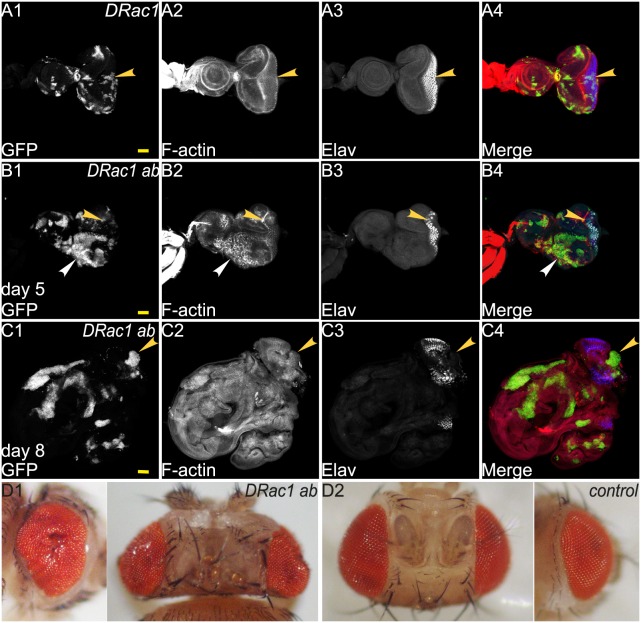
**Co-expression of *DRac1* with *ab* results in non-cell autonomous overgrowth.** Confocal planar images of mosaic larval eye-antennal discs stained for F-actin (with Phalloidin, grey or red in merge) and Elav (grey or blue in merge); mutant clones are GFP^+^ and wild-type tissue is GFP^−^ (grey or green in merge). (A) *DRac1* mosaic eye-antennal disc at day 5 AED. (B) *ab DRac1* mosaic eye-antennal disc at day 5 AED. (C) *ab DRac1* mosaic eye-antennal disc at day 8 AED. (D1) *ab DRac1* escaper adult fly heads, side and dorsal views. (D2) wild-type (control) adult fly heads, side and dorsal views. Yellow arrowheads point to patches of mutant tissue showing differentiation defects. Note that in panel C differentiation was observed in the wild-type tissue in the posterior region of the eye disc, but the highly folded nature of the wild-type tissue makes this difficult to image in a single Z section. White arrowheads point to an example of elevated F-actin. Genotypes: (A) *ey-FLP, UAS-GFP; UAS-DRac1; FRT82B/tubGAL4; FRT82B, tubGAL80*. (B,C,D1) *ey-FLP, UAS-GFP; UAS-DRac1*; *UAS-ab^55^, FRT82B/tubGAL4; FRT82B, tubGAL80*. (D2) *ey-GAL4.* Scale bars=50 μM.

Since *DRac1* and *Ras^ACT^* cooperate to form invasive tumours in the eye-antennal epithelium (Brumby et al., 2011), we sought to investigate if *ab* and *DRac1* also cooperate in tumorigenesis. In mosaic eye-antennal discs at day 5 AED, *DRac1* over-expression produced small clones with cell morphology defects (although F-actin levels were only slightly increased, Fig. 3A2) and disrupted Elav expression (yellow arrowheads, Fig. 3A1-A4). Over-expression of *ab* with *DRac1* resulted in large clones mostly in the anterior region of the eye disc (Fig. 3B), although overall there was less mutant clonal tissue in the eye-antennal disc compared with the wild-type mosaic eye-antennal disc. At later times (day 8/9 AED), *DRac1 ab* co-expression resulted in strong non-cell autonomous effects, as indicated by the highly folded wild-type tissue surrounding the clonal tissue and greater representation of GFP^−^ tissue (Fig. 3C). *DRac1 ab* co-expression resulted in rounded clones with elevated F-actin levels at day 5 AED (white arrowheads, Fig. 3B1,B2,B4) although at day 8/9 AED F-actin appeared elevated throughout the tissue (Fig. 3C2). In the posterior region of the eye disc, *DRac1 ab* expressing clones showed reduced Elav expression (yellow arrowheads, Fig. 3B1-B4,C1-C4). *DRac1 ab* larvae were delayed in development and pupated 1–2 days after the wild-type controls (data not shown). Most died at the pupal stage, however the occasional adult emerged (∼1/50 of expected numbers) with overgrown distorted eyes (Fig. 3D1) compared with the wild-type controls (Fig. 3D2). In summary, although *ab* cooperated with *DRac1*, this overgrowth was non-cell autonomous and the cooperation was not sufficient to form neoplastic tumours as observed with *DRac1 Ras^ACT^* (Brumby et al., 2011).

### Comparison of cooperative interactions relative to *scrib^−^ ab* tumours

**Fig. 4.**
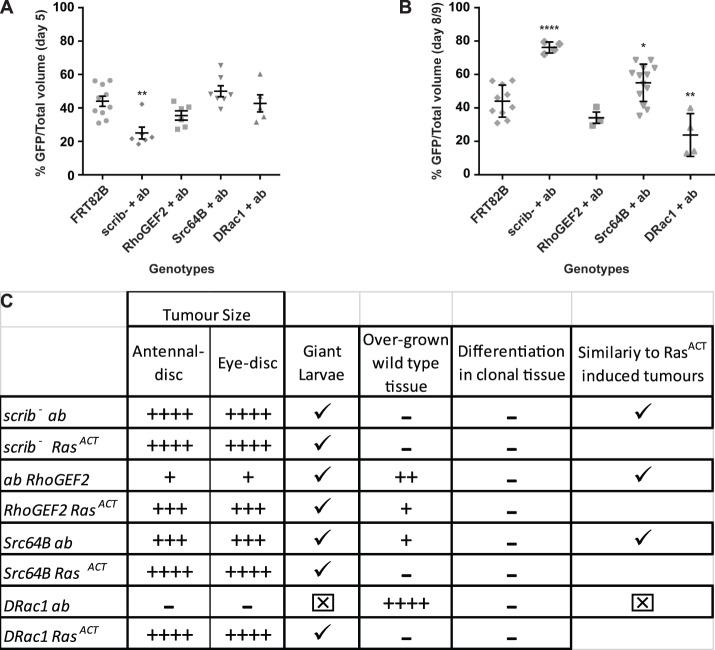
**Quantification of clonal overgrowth and summary of tumorigenic properties of *ab* with *scrib^−^, RhoGEF2, Src64B* or *DRac1*.** (A) Quantification of Day 5/6 larval eye-antennal disc GFP^+^ clonal volume relative to total disc volume normalized to the Day 5 FRP control. (B) Quantification of Day 8/9 larval eye-antennal disc GFP^+^ clonal volume relative to total disc volume, normalized to the Day 5 FRT control. The volume of GFP^+^ clonal tissue relative to total disc volume was measured from confocal sections covering the whole eye-antennal disc of at least 3 samples per genotype. The data is presented as a percentage of GFP^+^ tissue versus total tissue volume. The data was compared using unpaired *t*-test (two-tailed with 99% confidence level); error bars represent standard error of the mean (s.e.m.). *P<0.05, **P<0.007, and ****P<0.0001. (C) Summary of the tumorigenic phenotypes in comparison with Ras^V12^-driven tumours.

The comparative overgrowth at day 5/6 AED and day 8/9 AED for expression of the actin cytoskeletal genes with *ab* relative to *scrib^−^ ab* is summarized in Fig. 4. To determine the relative overgrowth of the mutant tissue to wild-type tissue we quantified the volume of GFP^+^ tissue to total eye-antennal disc volume for all genotypes at day 5/6 and day 8/9 (Fig. 4A,B). At day 5/6 AED the GFP^+^ tumour volume relative to the total disc volume for *RhoGEF2 ab*, *Src64B ab* and *DRac1 ab* was similar to the *FRT* control, but *scrib^−^ ab* clonal tissue was slightly reduced relative to wild-type tissue (Fig. 4A). However, at day 8/9 AED, *scrib^−^ ab* GFP^+^ tumours represented the majority of the overgrown discs (Fig. 4B). *Src64B ab* GFP^+^ tumours were also more greatly represented relative to the wild-type tissue, however although the whole tissue was overgrown *RhoGEF2 ab* GFP^+^ tumours did not overgrow relative to the wild-type tissue (Fig. 4B). By contrast, *DRac1 ab* clones were underrepresented in the overgrown discs, suggesting that non-cell autonomous overgrowth had occurred (Fig. 4B). All cooperative interactions affected differentiation of photoreceptor development as judged by ELAV staining (Fig. 4C). Relative to *scrib^−^ ab* cooperative tumorigenesis, co-expression of the cytoskeletal genes with *ab* resulted in less potent cooperative overgrowth at day 8/9 AED (Fig. 4A-C), which was correlated with non-cell autonomous tissue growth effects. Except for *DRac1 ab*, all cytoskeletal genes showed similar properties in cooperation with *ab* as with *Ras^V12^* (Fig. 4C).

### Cooperation of *abrupt* with *RhoGEF2, Src64B* or *DRac1* affects tissue growth via effects on cell proliferation and cell death

**Fig. 5.**
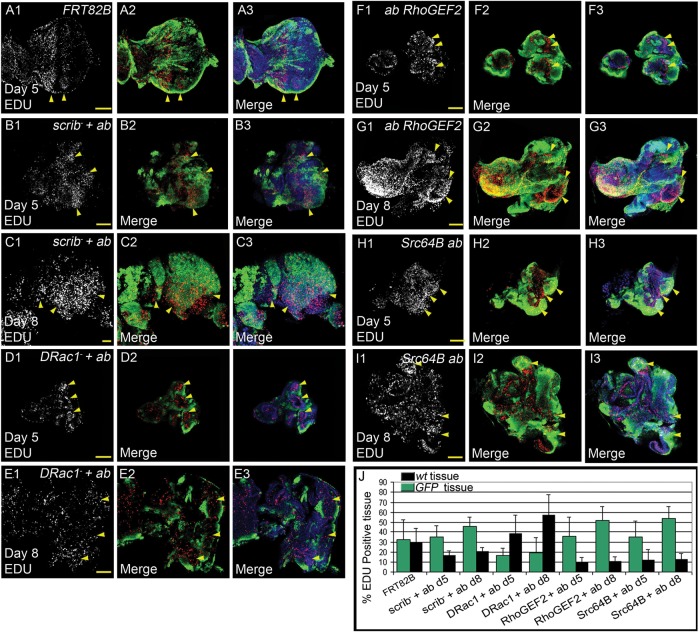
**Comparison of cell proliferation levels in *ab* with *scrib^1^*, *DRac1, RhoGEF2* or *Src64B.*** Confocal planar images of mosaic larval eye-antennal discs labelled with EdU for S-phases (grey or red in merge) and DAPI (blue in merge); mutant clones are GFP^+^ and wild-type tissue is GFP^−^ (green in merge). Arrowheads point to patches of tissue showing alterations in cell proliferation. (A) wild-type control clones. (B) *ab scrib^1^* mosaic eye-antennal disc at day 5. (C) *ab scrib^1^* mosaic eye-antennal disc at day 8. (D) *DRac1 ab* mosaic eye-antennal disc at day 5. (E) *DRac1 ab* mosaic eye-antennal disc at day 8. (F) *RhoGEF2 ab* mosaic eye-antennal disc at day 5. (G) *RhoGEF2 ab* mosaic eye-antennal disc at day 8. (H) *Src64B ab* mosaic eye-antennal disc at day 5. (I) *Src64B ab* mosaic eye-antennal disc at day 8. (J) Quantification showing the percentage of EdU positive tissue in wild-type versus mutant clones of the listed genotypes. Error bars represent s.e.m. Genotypes: (A) *ey-FLP, UAS-GFP; FRT82B/tubGAL4; FRT82B, tubGAL80*. (B-C) *ey-FLP, UAS-GFP;; UAS-ab^55^, FRT82B, scrib^1^/tubGAL4; FRT82B, tubGAL80*. (D-E) *ey-FLP, UAS-GFP; UAS-DRac1*; *UAS-ab^55^, FRT82B/tubGAL4; FRT82B, tubGAL80*. (F-G) *ey-FLP, UAS-GFP; UAS-RhoGEF2*; *UAS-ab^55^, FRT82B/tubGAL4; FRT82B, tubGAL80*. (H-I) *ey-FLP, UAS-GFP; UAS-Src64B*; *UAS-ab^55^, FRT82B/tubGAL4; FRT82B, tubGAL80*. Scale bars=50 μM.

**Fig. 6.**
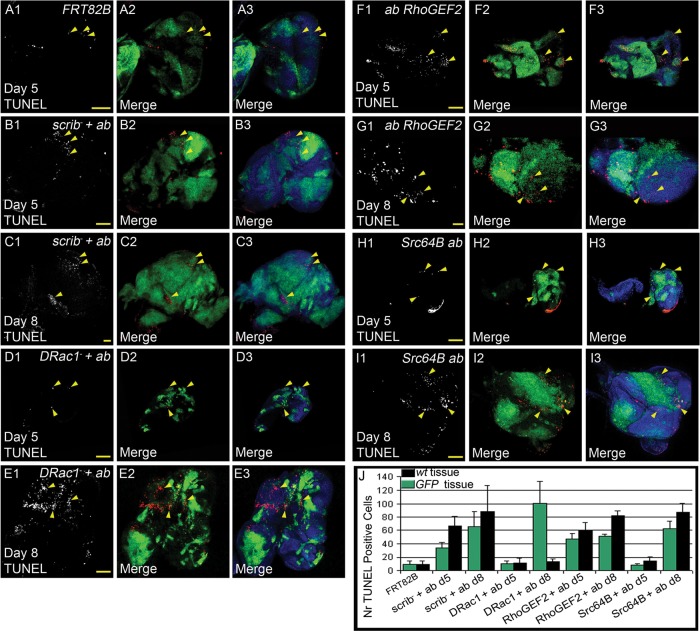
**Comparison of cell death levels in *ab* with *scrib^1^, DRac1, RhoGEF2* or *Src64B*.** Confocal planar images of mosaic larval eye-antennal discs labelled with TUNEL as an apoptotic marker (grey or red in merge) and DAPI (blue in merge); mutant clones are GFP^+^ and wild-type tissue is GFP^−^ (green in merge). Arrowheads point to patches of tissue showing alterations in cell death. (A) wild-type control clones. (B) *ab scrib^1^* mosaic eye-antennal disc at day 5. (C) *ab scrib^1^* mosaic eye-antennal disc at day 8. (D) *DRac1 ab* mosaic eye-antennal disc at day 5. (E) *DRac1 ab* mosaic eye-antennal disc at day 8. (F) *RhoGEF2 ab* mosaic eye-antennal disc at day 5. (G) *RhoGEF2 ab* mosaic eye-antennal disc at day 8. (H) *Src64B ab* mosaic eye-antennal disc at day 5. (I) *Src64B ab* mosaic eye-antennal disc at day 8. (J) Quantification showing the number of TUNEL positive cells in wild-type versus mutant clones of the listed genotypes. Error bars represent s.e.m. Genotypes: (A) *ey-FLP, UAS-GFP; FRT82B/tubGAL4; FRT82B, tubGAL80*. (B-C) *ey-FLP, UAS-GFP;; UAS-ab^55^, FRT82B, scrib^1^/tubGAL4; FRT82B, tubGAL80*. (D-E) *ey-FLP, UAS-GFP; UAS-DRac1*; *UAS-ab^55^, FRT82B/tubGAL4; FRT82B, tubGAL80*. (F-G) *ey-FLP, UAS-GFP; UAS-RhoGEF2*; *UAS-ab^55^, FRT82B/tubGAL4; FRT82B, tubGAL80*. (H-I) *ey-FLP, UAS-GFP; UAS-Src64B*; *UAS-ab^55^, FRT82B/tubGAL4; FRT82B, tubGAL80*. Scale bars=50 μM.

In order to assess how *ab* was cooperating with *RhoGEF2, Src64B* or *DRac1* relative to *scrib^−^* to affect tissue growth, we performed EdU labelling to reveal proliferating cells and TUNEL labelling to detect dying cells in mosaic eye-antennal discs from all genotypes at day 5/6 and day 8/9 (Figs 5 and 6). The EdU labelling experiment revealed that relative to the FRT control where cell proliferation ceases in the posterior region of the eye disc (Fig. 5A), *scrib^−^ ab*, *RhoGEF2 ab,* and *Src64B ab* GFP^+^ clones showed increased numbers of EdU^+^ cells in the posterior region as well as throughout the eye-antennal discs at day 5 and day 8 AED (Fig. 5B,C,F-J), however *DRac1 ab* GFP^+^ clones showed a reduction in EdU incorporation relative to the surrounding wild-type tissue (Fig. 5D,E,J). The analysis of cell death, revealed that there were more dying cells in the wild-type tissue (GFP^−^) in *scrib^−^ ab*, *RhoGEF2 ab,* and *Src64B ab* mosaic discs at day 5 and day 8 AED (Fig. 6B,C,F-J) versus the FRT control that showed only low levels of TUNEL^+^ cells (Fig. 6A). Conversely, *DRac1 ab* GFP^+^ clones showed more dying cells relative to the wild-type tissue at day 8 AED (Fig. 6E,J), although similarly low numbers of TUNEL^+^ cells were present in the mutant tissue (GFP^+^) versus wild-type tissue (GFP^−^) at day 5 AED (Fig. 6D,J). Altogether, these results show that increased cell proliferation of the mutant tissue and increased cell death of the wild-type tissue occurs in *scrib^−^ ab*, *RhoGEF2 ab,* and *Src64B ab* mosaic discs, whilst the opposite occurs in *DRac1 ab* mosaic discs. The EdU and TUNEL patterns are generally consistent with the tissue overgrowth data at day 8 (Fig. 4B), with the exception of *RhoGEF2 ab*, where the tumour did not overgrow relative to the wild-type tissue. Since EdU measures S phase cells, it is possible there might be delays in G2/M phase in the mutant tissue in this genotype to account for this effect. At day 5, the tumour volume was similar to wild-type for all samples, except for *scrib^−^ ab* where mutant tissue was less represented (Fig. 4A), therefore the EdU and TUNEL data at day 5 does not reflect tumour volume at this stage, but predicts what occurs later in tumour development (i.e. day 8).

### Cooperation of *abrupt* with *RhoGEF2, Src64B* or *DRac1* affects expression of critical eye and antennal differentiation genes

We have previously shown, by ChIP sequencing of Ab targets and expression array analysis, that Ab regulates the expression of eye-antennal cell fate genes and that this effect is enhanced or altered in *ab scrib^−^* tumours (Turkel et al., 2013). Since co-expression of *ab* with *RhoGEF2, Src64B* or *DRac1* also affects expression of the eye differentiation factor, Elav, we sought to determine whether other cell fate genes in eye and antennal development were also affected in these tumours. In eye development, Dachshund (Dac) is one of the earliest transcriptional regulators that drives cell fate determination in the developing eye (Chen et al., 1997; Shen and Mardon, 1997), and expression of Dac is blocked in *ab scrib^−^* tumours (Turkel et al., 2013). We therefore wished to determine if this was also the case in *ab* cytoskeletal gene cooperative tumours.

**Fig. 7.**
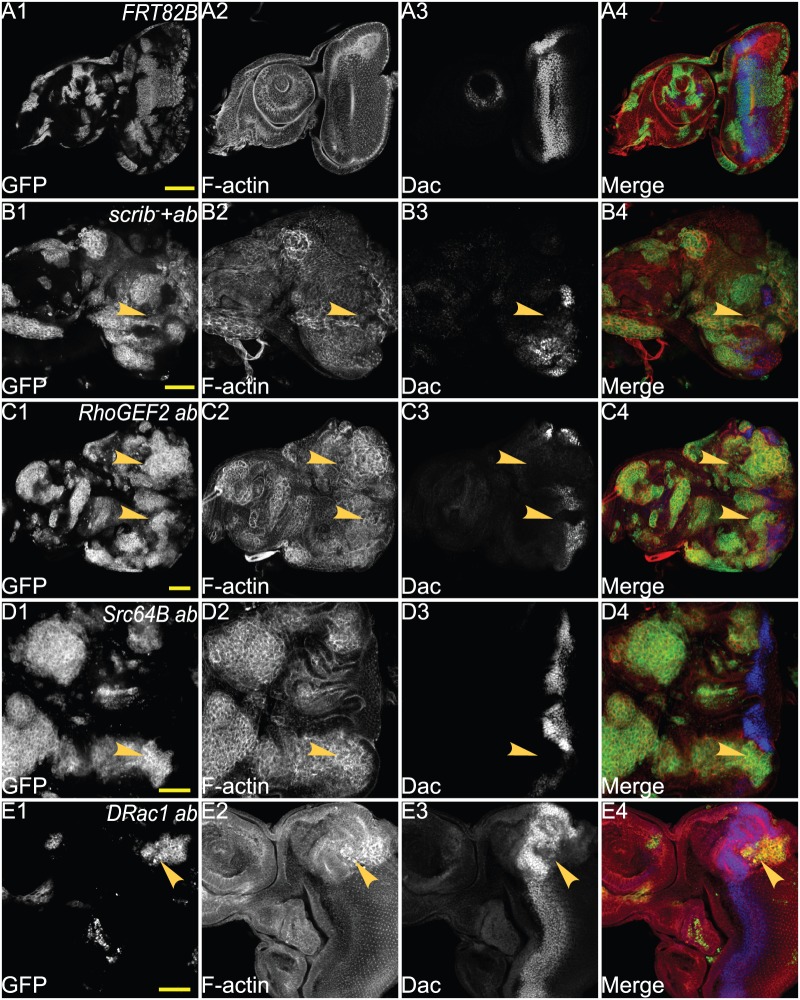
**Co-expression of *ab* with *scrib^−^*, *RhoGEF2, Src64B* or *DRac1* prevents the expression of the eye cell fate gene, Dac.** Confocal planar images of mosaic larval eye-antennal discs stained for F-actin (with Phalloidin, grey or red in merge) and Dac (grey or blue in merge); mutant clones are GFP^+^ and wild-type tissue is GFP^−^ (grey or green in merge). (A) wild-type control clones. (B) *ab scrib^1^* mosaic eye-antennal disc at day 5 AED. (C) *RhoGEF2 ab* mosaic eye-antennal disc at day 5 AED. (D) *Src64B ab* mosaic eye-antennal disc at day 5 AED. (E) *DRac1 ab* mosaic eye-antennal disc at day 5 AED. Arrowheads point to patches of mutant tissue showing lack of Dac expression. Genotypes: (A) *ey-FLP, UAS-GFP; FRT82B/tubGAL4; FRT82B, tubGAL80*. (B) *ey-FLP, UAS-GFP;; UAS-ab^55^, FRT82B, scrib^1^/tubGAL4; FRT82B, tubGAL80*. (C) *ey-FLP, UAS-GFP; UAS-RhoGEF2*; *UAS-ab^55^, FRT82B/tubGAL4; FRT82B, tubGAL80*. (D) *ey-FLP, UAS-GFP; UAS-Src64B*; *UAS-ab^55^, FRT82B/tubGAL4; FRT82B, tubGAL80*. (E) *ey-FLP, UAS-GFP; UAS-DRac1*; *UAS-ab^55^, FRT82B/tubGAL4; FRT82B, tubGAL80.* Scale bars=50 μM.

In wild-type eye-antennal discs, Dac is expressed in a broad band in the middle of the eye disc and also in a crescent in the antennal disc (Fig. 7A). *scrib^1^ ab* clones do not express Dac in the eye disc (arrowheads, Fig. 7B1-B4) or in the antennal disc. Dac expression is only slightly reduced in *scrib^1^* clones and unaffected in *ab* overexpressing clones in the eye disc (Turkel et al., 2013). In *RhoGEF2 ab* clones in the eye disc, Dac expression was blocked (arrowheads, Fig. 7C1-C4). Similarly, Dac expression was blocked in *Src64B ab* clones (arrowheads, Fig. 7D1-D4) and in *DRac1 ab* clones (arrowheads, Fig. 5E1-E4). Dac expression was also blocked in the antennal disc in *ab RhoGEF2, ab Src64B* or *ab DRac1* co-expressing clones (Fig. 7C-E; data not shown). Thus, similarly to *ab scrib^−^* tumours, *ab* cytoskeletal gene tumours appear to be blocked in differentiation prior to Dac expression.

In antennal disc differentiation, initial expression domains of the transcription factors Homothorax (Hth), Cut (Ct) and Distal-less (Dll) during 2nd instar larval development establish the early proximo-distal axis of the antenna (Dominguez and Casares, 2005). We have previously shown that *scrib^−^ ab* clones retain the expression of Dll within the growing tumour, but downstream regulated genes, such as Dac, are not retained (Turkel et al., 2013). We therefore tested if Dll was still expressed in *ab* cytoskeletal gene tumours.

**Fig. 8.**
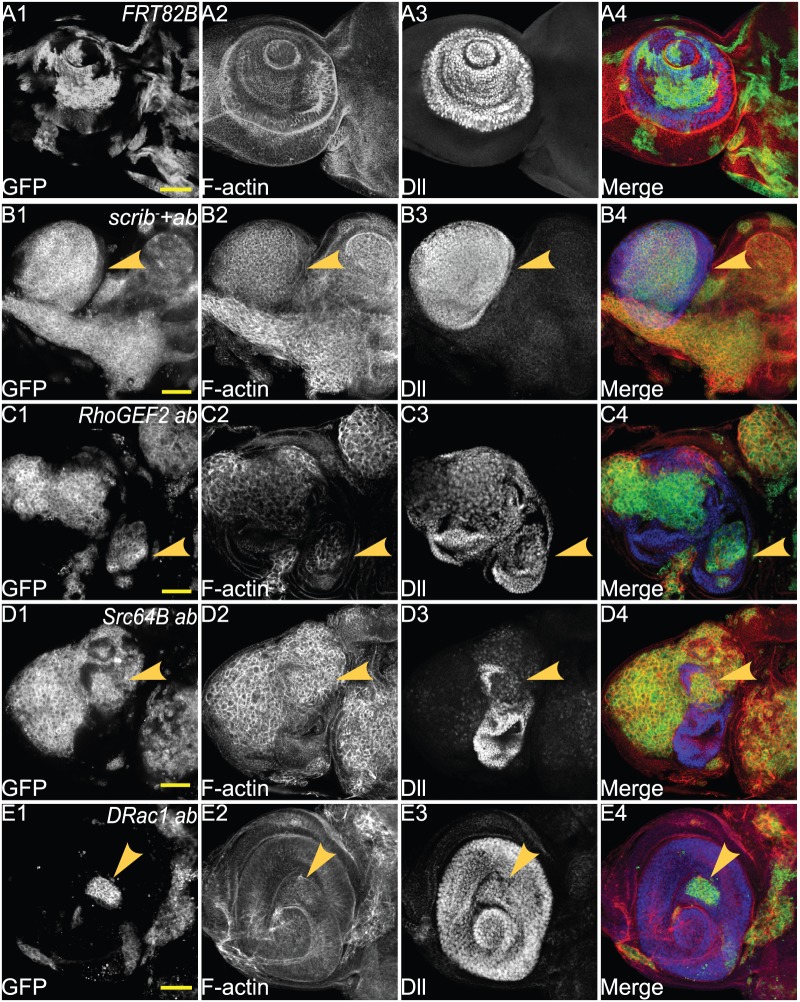
**Co-expression of *ab* with *Src64B* reduces expression of the antennal cell fate gene, Dll, but expression is retained in *ab scrib^−^*, *ab RhoGEF2* and *ab DRac1* clones.** Confocal planar images of mosaic larval antennal discs stained for F-actin (with Phalloidin, grey or red in merge) and Dll (grey or blue in merge); mutant clones are GFP^+^ and wild-type tissue is GFP^−^ (grey or green in merge). (A) wild-type control clones in the antennal disc at day 5 AED. (B) *ab scrib^1^* mosaic antennal disc at day 5 AED. (C) *RhoGEF2 ab* mosaic antennal disc at day 5 AED. (D) *Src64B ab* mosaic antennal disc at day 5 AED. (E) *DRac1 ab* mosaic antennal disc at day 5 AED. Arrowheads point to Dll expression. Genotypes: (A) *ey-FLP, UAS-GFP; FRT82B/tubGAL4; FRT82B, tubGAL80*. (B) *eyFLP, UAS-GFP;; UAS-ab^55^, FRT82B, scrib^1^/tubGAL4; FRT82B, tubGAL80*. (C) *eyFLP, UAS-GFP; UAS-RhoGEF2*; *UAS-ab^55^, FRT82B/tubGAL4; FRT82B, tubGAL80*. (D) *eyFLP, UAS-GFP; UAS-Src64B*; *UAS-ab^55^, FRT82B/tubGAL4; FRT82B, tubGAL80*. (E) *eyFLP, UAS-GFP; UAS-DRac1*; *UAS-ab^55^, FRT82B/tubGAL4; FRT82B, tubGAL80*. Scale bars=50 μM.

**Fig. 9.**
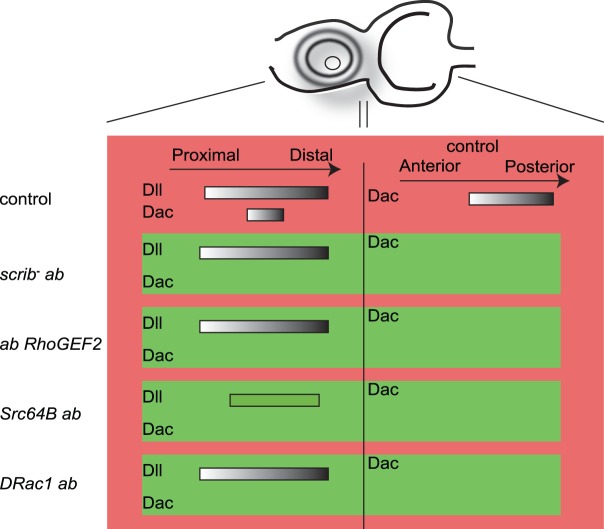
**Summary of the effects of *ab scrib^−^*, *ab Src64B*, *ab RhoGEF2* and *ab DRac1* on Dac and Dll expression.** A schematic of an eye-antennal disc showing the normal expression of Dac and Dll, and the effect of *ab scrib^−^*, *ab Src64B*, *ab RhoGEF2* and *ab DRac1* on their expression patterns.

In wild-type antennal discs, Dll is expressed in more distally destined cells in the antennae (Fig. 8A), and *scrib^−^ ab* clones retain this expression (arrowheads, Fig. 8B1-B4). Co-expression of *RhoGEF2* with *ab* did not block Dll expression, and instead an enlarged Dll-expression domain was observed (arrowheads, Fig. 8C1-C4), probably due to a partial duplication of the antennae, which is sometimes observed in *ab*-expressing clones (Turkel et al., 2013). Surprisingly, *Src64B ab* clones showed reduced expression of Dll (arrowheads, Fig. 8D1-D4) and distortion of the antennal structures due to cell morphology changes (Fig. 8D2). In *DRac1 ab* clones, normal expression of Dll was also observed (arrowheads, Fig. 8E1-E4). Altogether, these results show that *RhoGEF2 ab* and *DRac1 ab* are similar to *scrib^−^ ab* in cell fate status, however *Src64B ab* tumours are blocked at an earlier progenitor cell state than *scrib^−^ ab* tumours (summarized in Fig. 9).

### Correlation in expression of oncogenic BTB-Zinc finger genes, *Bcl6* and *ZBTB7A*, with apico-basal cell polarity and cytoskeletal genes in human epithelial cancer

**Fig. 10.**
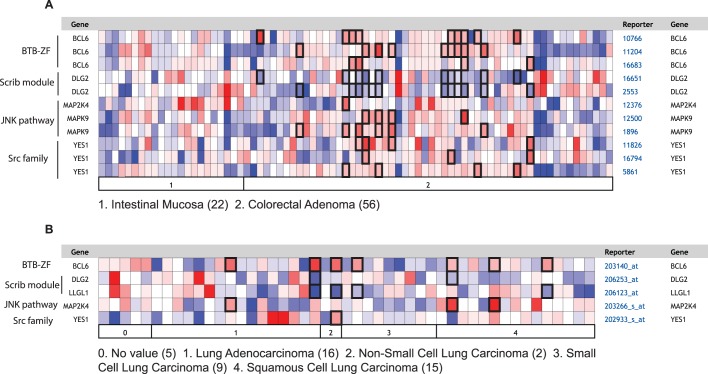
**Heatmaps of expression of *Bcl6* relative to polarity or cytoskeletal regulatory genes in the Gaspar colon and Rohrbeck Lung datasets.** (A) Gaspar Colon data set. (B) Rohrbeck Lung data set. Samples are stratified into normal tissue (intestinal mucosa for Gaspar Colon or no value for Rohrbeck Lung) and cancer grades for Rohrbeck Lung. Relative expression levels of the indicated gene probesets are indicated. The Gaspar Colon dataset has 3 probes to *Bcl6* and *Yes1*, and 2 probes to *Dlg2* and *MAPK9*. Red is high expression and blue is low expression. The outlined samples indicate those where *Bcl6* is high and *Dlg2* or *Llgl1* are low or *MAP2K4, MAPK9* or *Yes1* are high.

Since we have shown here that *ab* cooperates with the cytoskeletal regulators, *RhoGEF2* and *Src64B,* to result in cooperative tumorigenesis, we wished to determine whether the expression of human homologs of these genes showed cooperation with BTB-Zn finger genes in human cancers. Since our previous studies had also shown that the cell polarity tumour suppressor, *scrib*, showed cooperative tumorigenesis with *ab* (Turkel et al., 2013), we also sought to determine whether human homologs of the Scribble module were downregulated in human tumours, showing high expression of BTB-Zn finger genes. Furthermore, since we have shown that the JNK signalling pathway was important in the invasive properties of these tumours and sufficient to cooperate with *Ras^ACT^* in *Drosophila* and mammalian invasive tumour growth (Brumby et al., 2011), we wished to examine the correlation of expression of the human JNKK and JNK homologs with BTB-Zn finger genes in human cancer. Of the human BTB-Zn finger genes, there is greatest evidence for *Bcl6* and *ZBTB7A* as oncogenes in human epithelial cancer (see Introduction), so we focused our analysis on these genes. Using Oncomine, we analysed collections of human epithelial cancers for expression correlation with *Bcl6* or *ZBTB7A* and human *RhoGEF2* homologs (*ARHGEF1, ARHGEF11, ARHGEF12*), *Src* homologs (*Src, Yes, Fyn*), Scribble module genes (*hscrib, llgl1, llgl2, dlg1, dlg2, dlg3, dlg4*), JNKK homologs (*MAP2K4, MAP2K7*) and JNK homologs (*MAPK8, MAPK9, MAPK10*). In this analysis, the cancer samples were compared with normal tissues where available. The correlation of expression of each of these polarity and cytoskeletal genes in each dataset revealed that there were significant correlations (*P*<0.05) for several cytoskeletal or polarity regulatory genes with *Bcl6* or *ZBTB7A* in several cancer types (Table 1; supplementary material Table S1 and supplementary data). Most interestingly, in the Gaspar Colon colorectal adenoma dataset (Gaspar et al., 2008) *Bcl6* expression was significantly positively correlated with *MAPK9, MAP2K4* and *Yes1*, and negatively with *Dlg2*, relative to normal intestinal mucosa (Fig. 10A, Table 1). The heatmap of individual samples, revealed a trend of high *Bcl6*, low *Dlg2* and high *MAPK9* in many colorectal carcinoma samples relative to the normal intestinal mucosa (Fig. 10A). Furthermore, in the Rohrbeck Lung (all-Lung, cancer only) dataset (Rohrbeck et al., 2008) *Bcl6* expression was positively correlated with *MAP2K4, Yes1* and negatively correlated with *Dlg2* and *Lgl1* (Fig. 10B, Table 1). Stratification of the Rohrbeck Lung cancers dataset into different stages showed that there were several samples of lung adenocarcinoma or lung squamous cell carcinoma having high *Bcl6* expression and high *MAP2K4* expression relative to normal lung (No value), whereas high *Bcl6* expression correlated with low *Dlg2* or *Llgl1* in some samples from all forms of lung cancers relative to normal lung (Fig. 10B). Also significantly positively correlated with a stronger trend compared with normal tissue were *Bcl6* and *MAP2K7* in the Boersma breast epithelial cancer dataset (Boersma et al., 2008) and *ZBTB7A* and *MAP2K7* in the Zhai cervical squamous carcinoma dataset (Zhai et al., 2007) (Table 1). Also significant was that in the Toruner Head-Neck all oral squamous carcinoma (cancer only) dataset (Toruner et al., 2004) positive correlations were seen between *Bcl6* and *MAPK10* and between *ZBTB7A* and *ArhGef12*, and in the Tomlins prostate carcinoma dataset (Tomlins et al., 2007) Bcl6 expression was positively correlated with *ArhGef11* and *MAPK8* (Table 1). Significant positive correlations were also observed in the Collisson Pancreatic adenocarcinoma (cancer only) dataset (Collisson et al., 2011) between *ZBTB7A* and *Src* (Table 1)*.* Furthermore, in the Grutzmann pancreatic ductal adenocarcinoma dataset (Grützmann et al., 2004), although of borderline significance, a positive correlation was observed between *ZBTB7A* and *MAP2K7* that showed a stronger trend compared with normal tissue (Table 1). Thus, taken together, these data show that in certain epithelial cancers the upregulation of *Bcl6* or *ZBTB7A* expression is significantly correlated with reduced expression of *Dlg2* or *Llgl1* cell polarity genes or high expression of *ArhGef11, ArhGef12, MAP2K4, MAP2K7, MAPK8, MAPK9, MAPK10, Src* or *Yes1* cytoskeletal genes. Based on our functional data in *Drosophila* and mammalian cells (this study; Brumby et al., 2011; Khoo et al., 2013; Turkel et al., 2013; C.P., A.B., H.R., unpublished data), we would expect the concordant expression of *Bcl6* or *ZBTB7A* with these genes should result in tumour growth, morphology changes, differentiation blockage and invasive properties.

**Table 1.**
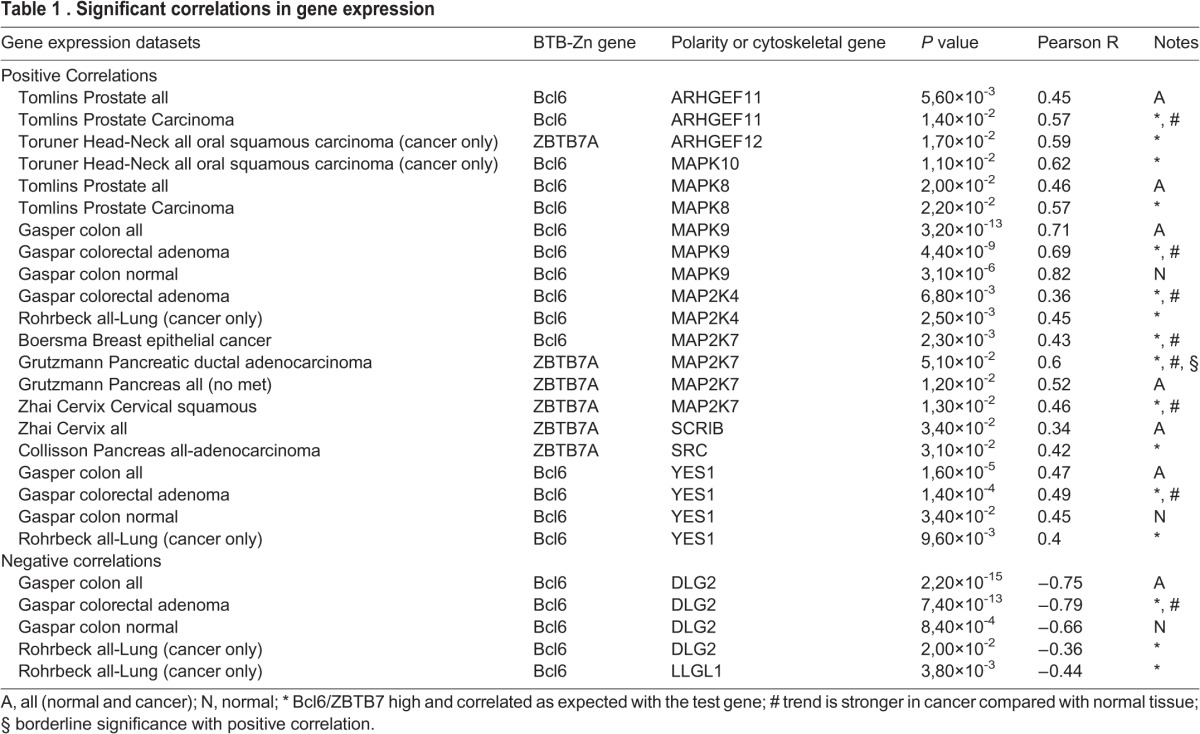
**Significant correlations in gene expression**

## DISCUSSION

In this study, we have shown that over-expression of the Ab BTB-ZF protein cooperates with upregulation of RhoGEF2 or Src64B in tumorigenesis, whereas Ab and DRac1 do not cooperate. Furthermore, we show that expression of Ab with each of these cytoskeletal regulators results in disruption to differentiation, in that the photoreceptor cell marker, Elav, and the early cell fate gene, Dac, are not expressed, although the antennal cell fate gene, Dll, is retained in all except *ab Src64B* co-expressing clones. Finally, we have found significant correlations in human epithelial cancer datasets between the high expression of BTB-ZF oncogenes, *Bcl6* and *ZBTB7A*, and low expression of *Dlg2* or *Llgl1* cell polarity genes or high expression of *ArhGef11*, *ArhGef12, MAP2K4, MAP2K7, MAPK8, MAPK9, MAPK10, Src* or *Yes1* cytoskeletal genes. This data suggests that cooperation between these genes may occur in some human epithelial cancers.

### Comparison of tumorigenic properties

*RhoGEF2 ab* or *Src64B ab* tumours showed overgrowth during an extended larval period resulting in giant larvae and loss of differentiation (Fig. 4C). However, unlike *scrib^−^ ab* tumours there was also non-cell autonomous proliferation and the tumours did not appear to be as invasive as *scrib*^−^ *ab* tumours, although a more detailed analysis of this is required. By contrast, co-expression of *DRac1* and *ab* did not result in cooperative tumorigenesis, but rather non-cell autonomous proliferation. Relative to the cooperation of these cytoskeletal genes with *Ras^V12^* (Brumby et al., 2011; Khoo et al., 2013; C.P., A.B., H.R., unpublished data), *RhoGEF2* or *Src64B* cooperation with *ab* showed similar properties (Fig. 4C). By contrast, *DRac1 Ras^V12^* tumours showed strong cell-autonomous overgrowth and invasive properties, whereas *DRac1 ab* expressing cells did not overgrow relative to wild-type tissue, but instead the surrounding wild-type tissue was induced to overgrow (Fig. 4C).

The phenomenon of non-cell autonomous overgrowth observed in *DRac1 ab* mosaic eye-antennal discs (and to some extent in *ab RhoGEF2* and *ab Src64B* mosaic discs) is similar to the effect that “undead” cells (cells where apoptosis is initiated by activation of initiator caspases, but effector caspase activation is blocked – and thus cell death – by expression of the inhibitor, p35) have upon their surrounding wild-type neighbours (Martin et al., 2009; Morata et al., 2011; Perez-Garijo et al., 2009; Ryoo and Bergmann, 2012). This occurs by the release of Wingless (Wg) and Decapentaplegic (Dpp) and perhaps other morphogens from the undead cells, which promote compensatory proliferation in the surrounding wild-type cells. The similarity of these phenotypes suggests that *DRac1 ab* expressing cells might be in an “undead” state, and release Dpp and Wg, thereby inducing proliferative overgrowth of the surrounding wild-type cells. Alternatively, these cells might be deficient in mitochondrial function, which together with expression of a cell-survival factor, such as *Ras^V12^*, results in non-cell autonomous overgrowth without evidence of caspase activation (Ohsawa et al., 2012). In this scenario, the mitochondrial dysfunction results in increased reactive oxygen species (ROS) that activate JNK signalling, which subsequently inactivates Hippo pathway signalling, leading to increased expression of the target genes Wingless and Unpaired (Upd) that activate Wg signalling and Jak/Stat signalling, respectively, in the neighbouring wild-type cells. However, since we observed TUNEL-positive cells in *DRac1 ab, RhoGEF2 ab* and *Src64B ab* expressing clones, it is more likely that the first of these mechanisms is responsible for the non-cell autonomous overgrowth, however this requires further investigation. Interestingly, in undead cells JNK activation is required for Dpp and Wg production and non-cell autonomous overgrowth (Morata et al., 2011; Perez-Garijo et al., 2009). Furthermore, strong activation of JNK signalling together with *Ras^V12^* results in non-cell autonomous overgrowth (Uhlirova et al., 2005), although at presumably lower levels of JNK activation, cell autonomous overgrowth occurs (Brumby et al., 2011; Igaki et al., 2006; Uhlirova and Bohmann, 2006). Therefore it is possible that the different effects on non-cell autonomous versus autonomous cell overgrowth in *DRac1 ab* versus *RhoGEF2 ab* or *Src64B ab-*expressing cells might depend on the level of JNK activation. Nonetheless, at early stages, *ab*-driven R*hoGEF2, Src64B* or *DRac1* tumours were similar in inducing non-cell autonomous effects, but at later times the *RhoGEF2 ab* and *Src64B ab-*expressing cells showed more predominant autonomous cell overgrowth, whilst the *DRac1 ab* expressing cells did not, suggesting that there are likely to be molecular differences between *DRac1* and *RhoGEF2* or *Src64B* in their cooperative interactions with *ab* that impact on cell proliferation or survival of the tumour cells.

Our profiling of Ab targets and deregulated genes revealed that *dac, dan, eya* and *ct* eye-antennal differentiation genes were repressed, along with changes in expression of cell growth/proliferation and survival genes that would be expected to promote tumorigenic growth in cooperation with *scrib* loss-of-function (Turkel et al., 2013). *scrib*^−^ *ab* tumours showed downregulation of Dac, but the antennal cell fate expression domain of Dll was not affected (Turkel et al., 2013). Similarly, *ab* expression with either of the cytoskeletal genes resulted in repression of Dac, however *Src64B ab* tumours additionally repressed Dll, in contrast to *DRac1 ab, RhoGEF2 ab* and *scrib^−^ ab* tumours where Dll was unaffected. This data suggests that *Src64B* expression exerts an additional effect on *ab*-expressing cells to inhibit Dll gene expression and differentiation. *Src* upregulation activates the JNK and Stat signalling pathways, affects adherens junction function and represses Hippo signalling (Enomoto and Igaki, 2013; Kohlmaier et al., 2014; Ma et al., 2013; Read et al., 2004; Sotillos et al., 2013; Vidal et al., 2006). Furthermore, recent studies have shown that overexpression of *Src64B* in the *Drosophila* intestinal stem cells can alter differentiation and result in amplification of progenitor cell pools (Cordero et al., 2014; Kohlmaier et al., 2014). *scrib* mutant cells also upregulate JNK, downregulate the E-cadherin/β-catenin adhesion complex and repress Hippo signalling (Brumby and Richardson, 2003; Doggett et al., 2011; Igaki et al., 2006; Leong et al., 2009; Uhlirova and Bohmann, 2006). Furthermore, the Jak/Stat ligand, Upd3, is also upregulated in the *scrib*^−^ cells, where it drives tumour overgrowth, and is also required to activate Jak/Stat signalling in the wild-type neighbouring cells in cell competition (Bunker et al., 2015; Chen et al., 2012; Schroeder et al., 2013). RhoGEF2 and DRac1 also upregulate JNK signalling (Brumby et al., 2011; Khoo et al., 2013), and might also repress Hippo signalling to promote tissue growth, since regulators of actin cytoskeletal tension, such as activated Rok and Myosin II regulatory light chain, induce Yki target gene expression (Fernandez et al., 2011; Halder et al., 2012; Rauskolb et al., 2014; Sansores-Garcia et al., 2011). However, in *Drosophila* it is unknown if RhoGEF2 or DRac1 affect Jak/Stat signalling. Since *scrib* loss-of-function and *Src* activation deregulate similar pathways, the precise mechanism by which *Src64B* cooperates with *ab* to block expression of Dll in the developing eye-antennal disc remains to be determined.

### Cooperation of BTB-ZF transcription factors with deregulated cytoskeletal or polarity genes in human cancer

Our finding that there was a significant correlation between increased expression of human BTB-ZF oncogenic genes, *Bcl6* or *ZBTB7A*, and downregulation of the cell polarity genes, *Dlg2* and *Llgl1*, or homologs of *JNKK* (*MAPK2K4, MAPK2K7*), *JNK* (*MAPK8, MAPK9*, *MAPK10*), *RhoGEF2* (*ArhGEF11*, *ArhGEF12*) or *Src* (*Yes1, Src*) cytoskeletal genes in various epithelial cancers, suggests that the concordant expression of these genes might be contributing to human epithelial cancer initiation and progression. Whilst this study only focused on two of the 47 BTB-ZF genes in the human genome, it raises the question of whether other BTB-ZF genes might also show correlations with the expression of cytoskeletal or cell polarity genes in human epithelial cancers. However, tissue and cancer-grade specific effects might be observed, as a recently published study revealed that *ZBTB7A* was commonly deleted in late stage oesophageal, bladder, colorectal, lung, ovarian and uterine cancers (Liu et al., 2014). Moreover, they found that low *ZBTB7A* expression correlates with poor prognosis in colon cancer patients, suggesting that *ZBTB7A* plays a tumour suppressor function in these cancers. Interestingly, this study also found that in colon cancer xenografts, *ZBTB7A* represses the expression of genes in the glycolytic pathway, a metabolic pathway that is required for aggressive tumour growth, and that inhibition of this pathway reduces tumour growth. Pertinent to this finding, we found that blocking glycolytic pathways in *Drosophila* polarity-impaired tumours, impedes tumour growth without substantially affecting normal tissues (Willoughby et al., 2013), suggesting that downregulation of the Scribble polarity module might upregulate glycolytic metabolic pathways and be dependent on them for tumour growth and survival. It is therefore possible that the cooperation between *ab* and *scrib*^−^ or cytoskeletal genes in *Drosophila* may also reflect a need for upregulation of the glycolytic pathway. In human epithelial cancers, the correlations observed between elevated *ZBTB7A* expression and reduced expression of the Scribble polarity module gene (or high expression of cytoskeletal genes) might also indicate a requirement for glycolytic pathway activation for tumorigenesis. Further studies are clearly required to examine the cooperative effects of *Bcl6* or *ZBTB7A* with deregulated cytoskeletal or cell polarity genes in human epithelial cell lines and mouse models in order to discern whether our findings in *Drosophila* are indeed conserved in mammalian systems.

Identifying cooperative interactions in cancer is likely to provide novel therapeutic approaches in combating the tumour. Indeed, recently a small molecule inhibitor targeting Bcl6 has been developed, and combining this with a Stat3 inhibitor resulted in enhanced cell killing in triple negative breast cancer cell lines (Walker et al., 2014). Since in *Drosophila* and human cells, Src upregulates Stat activity (Cordero et al., 2014; Frame, 2004; Kohlmaier et al., 2014; Read et al., 2004; Sotillos et al., 2013), tumours showing high Bcl6 and Src or Yes1 expression would be predicted to be sensitive to this combined therapeutic regime. Interestingly, a predominance of the significant correlations that were observed in the human epithelial cancer datasets with either *Bcl6* or *ZBTB7A* involved upregulation of JNKK and JNK family genes. Since JNK signalling is central to many cooperative interactions examined by us and others (Brumby et al., 2011; Brumby and Richardson, 2003; Enomoto and Igaki, 2013; Igaki et al., 2006; Leong et al., 2009; Turkel et al., 2013; Uhlirova and Bohmann, 2006), inhibiting the JNK pathway in addition to Bcl6 in Bcl6-driven cancers might also be a promising therapeutic approach to combat these cancers. In summary, our functional studies in *Drosophila* and bioinformatics analysis of human cancers has shown that cooperative tumorigenic interactions occur between BTB-ZF genes and cell polarity or cytoskeletal genes, and warrants further investigation to determine whether restoring normal expression of these genes or downstream pathways in human cancer cells can reduce tumorigenesis.

## MATERIALS AND METHODS

### *Drosophila* stocks

The following *Drosophila* stocks were used: *ey-FLP1*, *UAS-mCD8-GFP;; Tub-GAL4*, *FRT82B*, *Tub-GAL80* (Lee and Treisman, 2001); *UAS-ab^55^ (III)* (Cook et al., 2004); *UAS-RhoGEF2 (II)* (Padash Barmchi et al., 2005); *UAS-Src64B (II)* (R. Cagan, Mount Sinai School of Medicine, New York, USA); *UAS-DRac1 (II)* (Luo et al., 1994); *scrib^1^* (Bilder and Perrimon, 2000) and *ey-GAL4* (Bloomington Stock Centre)*. FRT82B* recombinant stocks were generated for all transgenic lines for mosaic analysis.

### Mosaic analysis

Clonal analysis utilised MARCM (mosaic analysis with repressible cell marker) (Lee and Luo, 1999) with *FRT82B* and *ey*-*FLP1* to induce clones and *mCD8-GFP* expression to mark mutant tissue. All fly crosses were carried out at 25°C and grown on standard fly media.

### Immunostaining

Third-instar larval eye-antennal discs were dissected in phosphate-buffered saline (PBS), fixed in 4% paraformaldehyde for 30 min, and washed in PBS+0.1% Triton X-100 (PBT). Samples were blocked in 2% NGS in PBT with 1.5% saponin for 1 h in room temperature and then incubated in primary antibodies over night at 4°C in 2% NGS in PBT. Samples were then washed two times in PBT for 30 min before addition of the secondary antibody. EdU and TUNEL labelling were performed as previously described (Turkel et al., 2013).

Antibodies used were: mouse anti-Elav (DSHB, 1/20), mouse anti-Dll (Duncan et al., 1998, 1/500) and mouse anti-Dac (DSHB, 1/10). Secondary antibodies were: anti-mouse Alexa 568 or 633 (Invitrogen) at 1/400 dilution. F-actin was detected with phalloidin–tetramethylrhodamine isothioblueate (TRITC; Sigma, 0.3 µM, 1/1000) and DNA was detected using DAPI staining. Samples were mounted in 80% (v/v) glycerol/PBS.

### Imaging

Images of fixed and mounted samples onto the glass slides were captured using BioRad, Olympus Fluoview FV100 and Leica TCS SP5 confocal laser microscopes. Single optical sections were selected in FluoView software before being processed in Adobe Photoshop CS6 and assembled into figures in Adobe Illustrator CS6.

Adult flies were frozen at −20°C before imaging in order to facilitate positioning them under the microscope. Images were captured on Lumenera Infinity 1 camera attached to Olympus SZX7 dissection microscope and processed using Adobe Photoshop CS3.

### Quantification of clone volume

Volumetric clone analysis was performed using Volocity 3D Image Analysis Software (Perkin-Elmer). To determine the ratio of clonal tissue volume to total volume of the eye-antennal disc for each genotype and time point, GFP^+^ clonal tissue relative to total disc area (as marked by Phallodin to visualize the cells) was measured from confocal Z sections encompassing the entire eye-antennal disc. The data for each genotype was compared using GraphPad Prism 6 using unpaired *t*-tests. Error bars represent s.e.m. and the significance was set at *P*<0.05.

### Quantification of EdU and TUNEL staining

For TUNEL and EdU labelling, 6 to 10 discs for each genotype were analysed. TUNEL was quantified using Photoshop 5.1 Extended. EDU was quantified using a program designed by David Tapiador, available at https://github.com/nogates/counting-semaphore.

### Analysis of published datasets

Using Oncomine (Research Premium Edition), we identified 18 published gene expression data sets that contain epithelial cancer samples. Data was filtered down to the genes of interest and was downloaded for further analysis. Eleven of the 18 data sets that have at least 30 samples and contain at least three quarters of our query genes were analysed for correlation of expression levels between BCL6/ZBTB7A and each of the genes in our gene panel. These data sets were: Boersma Breast (Boersma et al., 2008), Collisson Pancreas all-adenocarcinoma (Collisson et al., 2011), Gaspar Colon (Gaspar et al., 2008), Grützmann Pancreas (Grützmann et al., 2004), Ma Breast 2 (Ma et al., 2004), Ma Breast 4 (Ma et al., 2009), Rohrbeck Lung (Rohrbeck et al., 2008), Skrzypczak Colorectal 2 (Skrzypczak et al., 2010), Tomlins Prostate (Tomlins et al., 2007), Toruner Head-Neck all-oral squamous carcinoma (Toruner et al., 2004) and Zhai Cervix (Zhai et al., 2007). Where data is available, samples are stratified into normal (no cancer) and cancer for separate analysis to identify cancer-specific gene expression correlations. All analyses were done using the R software package.

## Supplementary Material

Supplementary Material
