# Supplementary material for: Cooperation of the BTB-Zinc finger protein, Abrupt, with cytoskeletal regulators in Drosophila epithelial tumorigenesis
Source: Biol Open. 2015 Jul 17;4(8):1024–39. doi: 10.1242/bio.012815 (PMC4542289; doi:10.1242/bio.012815)
Supplement: Supplementary Material [file supp_4_8_1024__index.html]

Cooperation of the BTB-Zinc finger protein, Abrupt, with cytoskeletal regulators in Drosophila epithelial tumorigenesis — Cooperation of the BTB-Zinc finger protein, Abrupt, with cytoskeletal regulators in Drosophila epithelial tumorigenesis — Supplementary Material 

# Cooperation of the BTB-Zinc finger protein, Abrupt, with cytoskeletal regulators in *Drosophila* epithelial tumorigenesis

## BIO012815 Supplementary Material

- Supplementary Material
