# Supplementary material for: Cooperation of the BTB-Zinc finger protein, Abrupt, with cytoskeletal regulators in Drosophila epithelial tumorigenesis
Source: Biol Open. 2015 Jul 17;4(8):1024–39. doi: 10.1242/bio.012815 (PMC4542289; doi:10.1242/bio.012815)
Supplement: Supplementary Material [file supp_bio.012815_BIO012815supp.pdf]

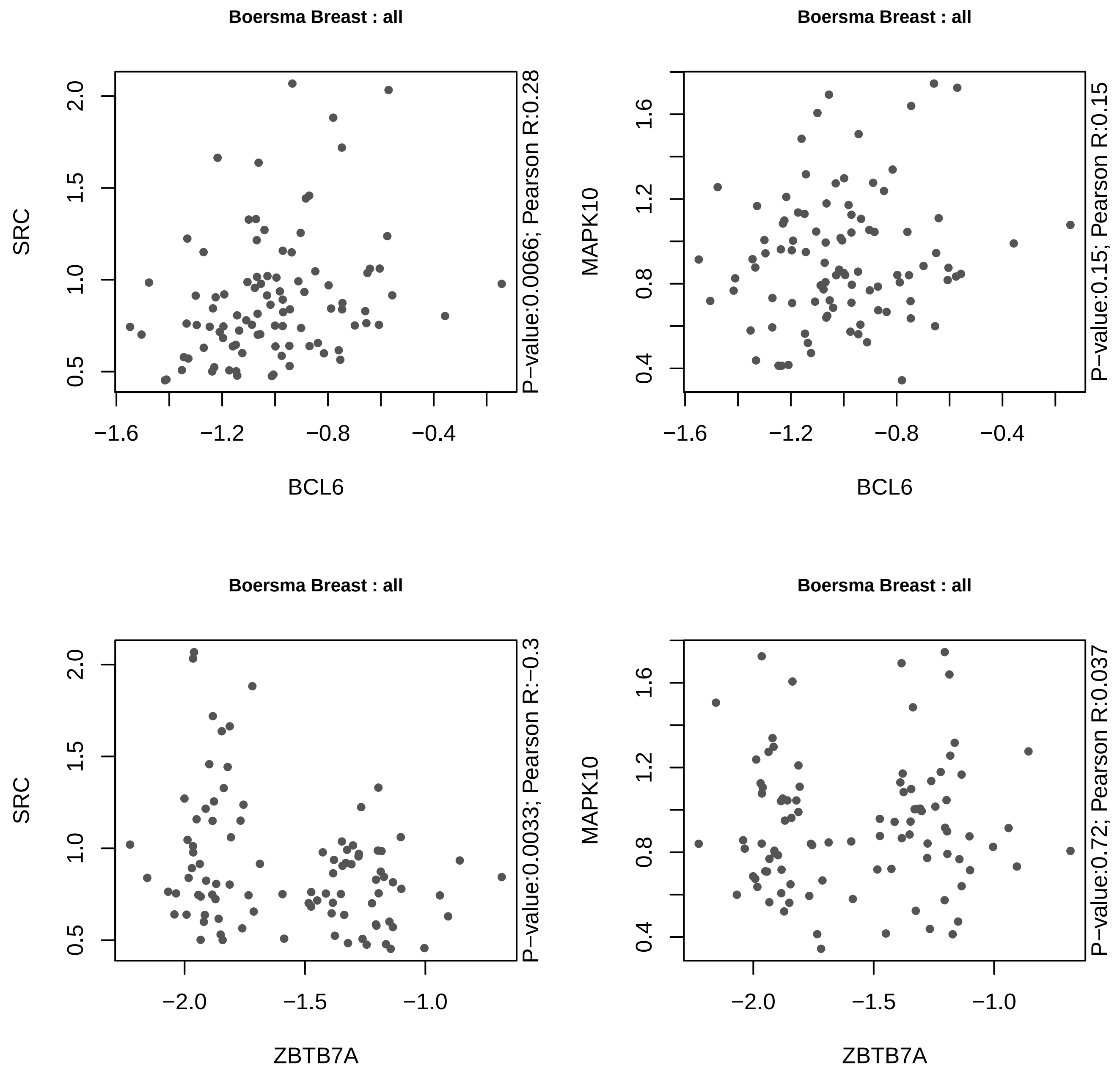
Boersma Breast : all
SRC
BCL6
P−value:0.0066; Pearson R:0.28
Boersma Breast : all
MAPK10
BCL6
P−value:0.15; Pearson R:0.15
Boersma Breast : all
SRC
ZBTB7A
P−value:0.0033; Pearson R:−0.3
Boersma Breast : all
MAPK10
ZBTB7A
P−value:0.72; Pearson R:0.037

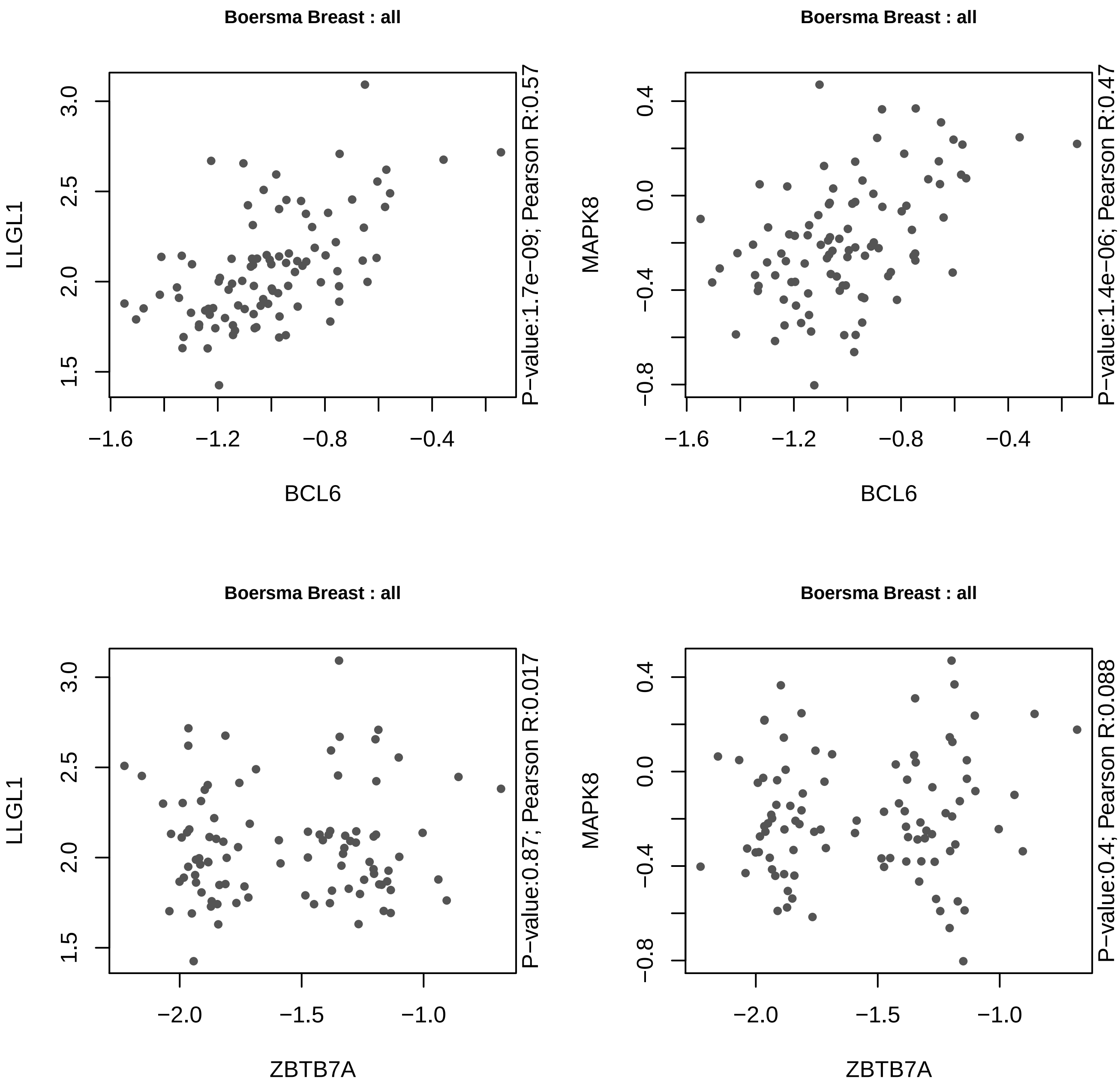
Boersma Breast : all
LLGL1
BCL6
P−value:1.7e−09; Pearson R:0.57
Boersma Breast : all
MAPK8
BCL6
P−value:1.4e−06; Pearson R:0.47
Boersma Breast : all
LLGL1
ZBTB7A
P−value:0.87; Pearson R:0.017
Boersma Breast : all
MAPK8
ZBTB7A
P−value:0.4; Pearson R:0.088

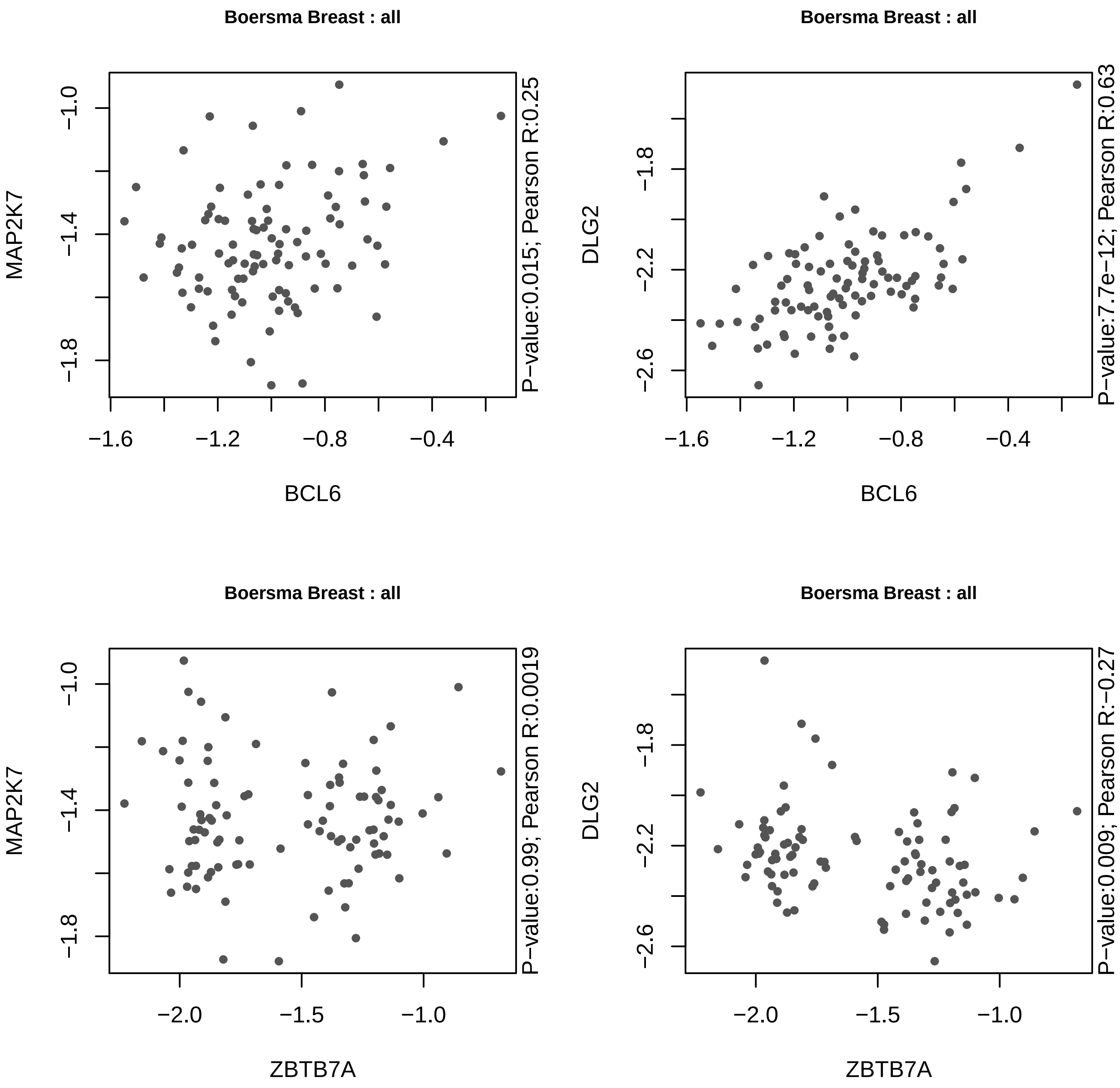
Boersma Breast : all
MAP2K7
BCL6
P−value:0.015; Pearson R:0.25
Boersma Breast : all
DLG2
BCL6
P−value:7.7e−12; Pearson R:0.63
Boersma Breast : all
MAP2K7
ZBTB7A
P−value:0.99; Pearson R:0.0019
Boersma Breast : all
DLG2
ZBTB7A
P−value:0.009; Pearson R:−0.27

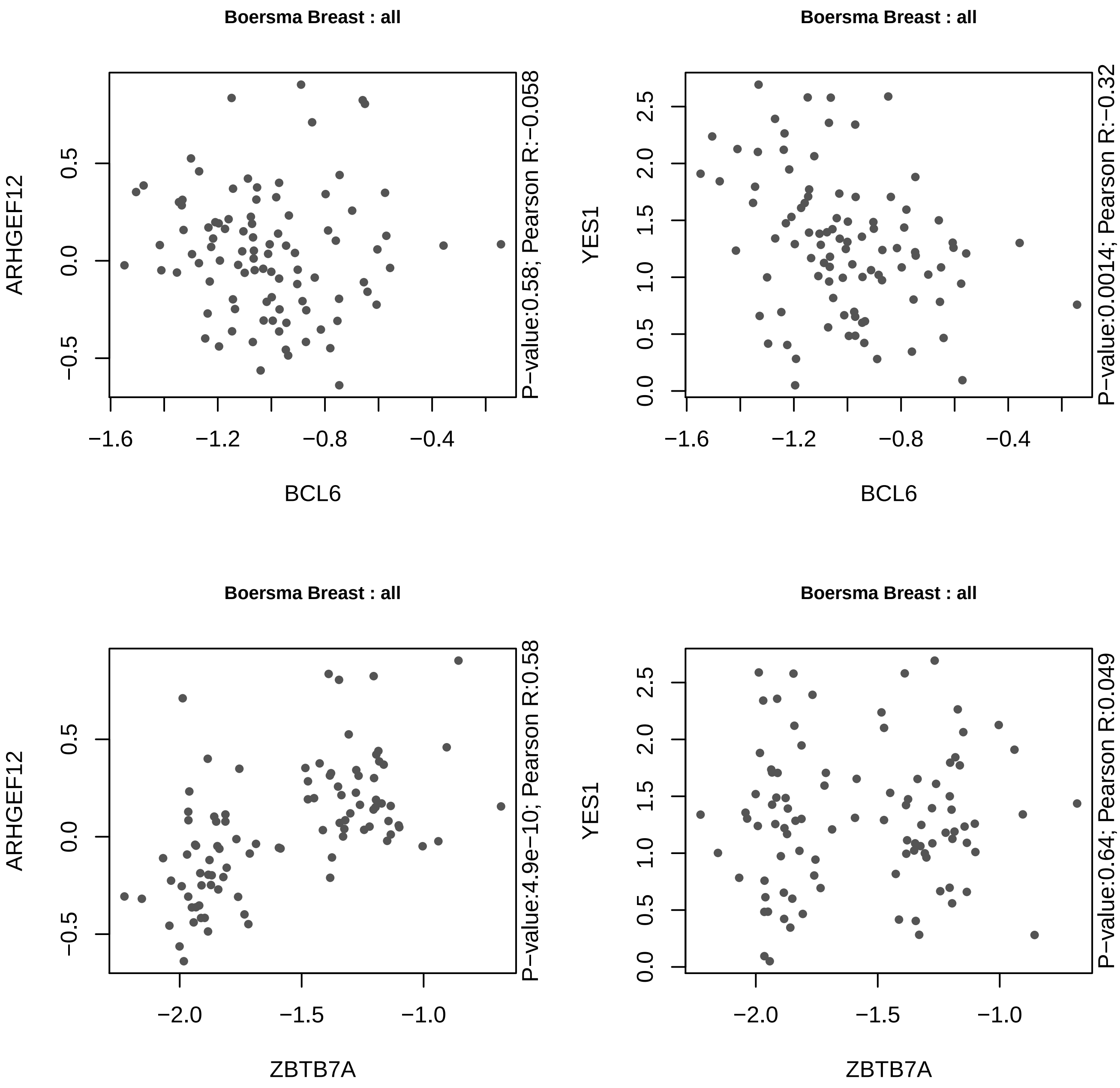
Boersma Breast : all
ARHGEF12
BCL6
P−value:0.58; Pearson R:−0.058
Boersma Breast : all
YES1
BCL6
P−value:0.0014; Pearson R:−0.32
Boersma Breast : all
ARHGEF12
ZBTB7A
P−value:4.9e−10; Pearson R:0.58
Boersma Breast : all
YES1
ZBTB7A
P−value:0.64; Pearson R:0.049

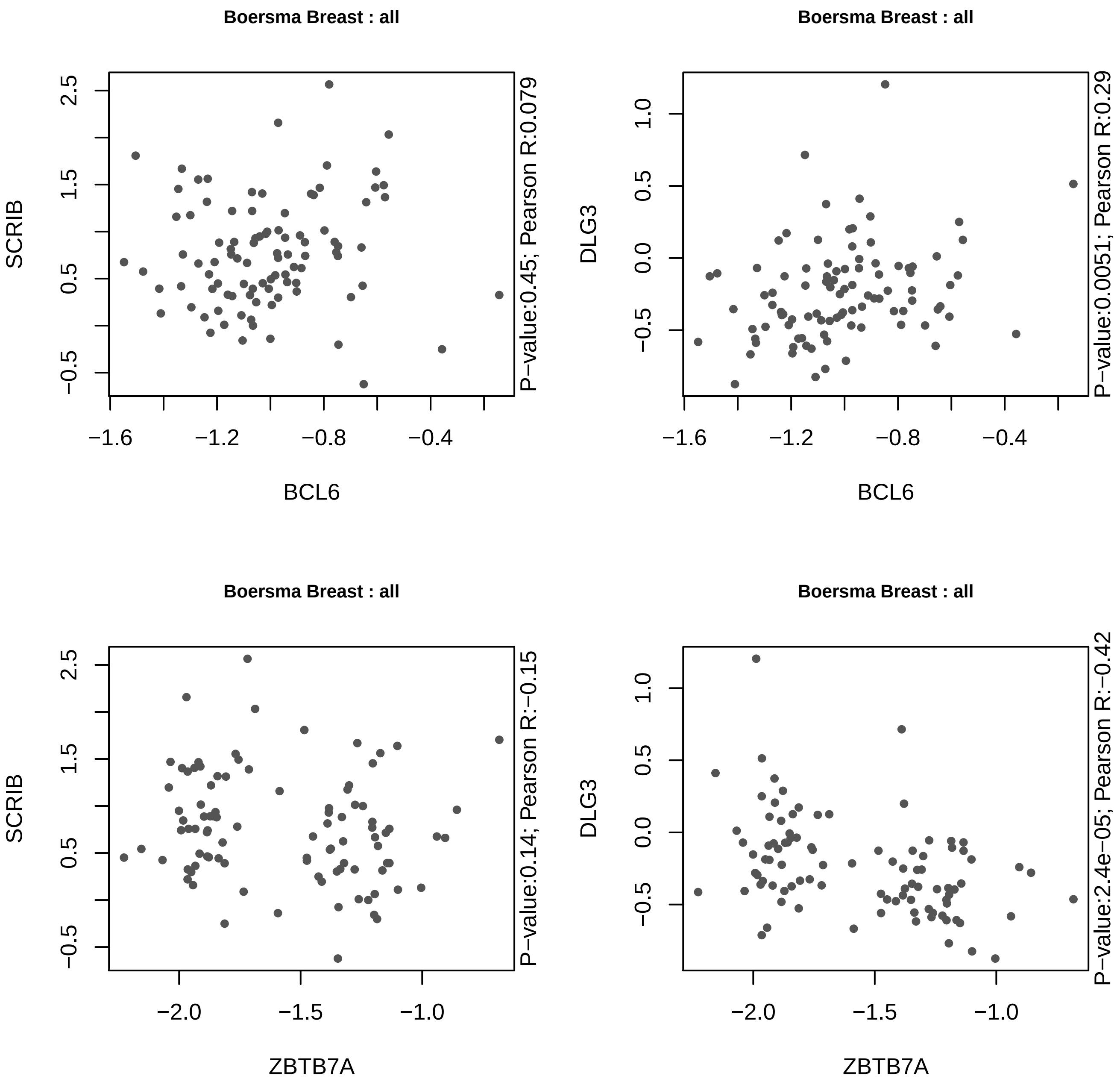
Boersma Breast : all
SCRIB
BCL6
P−value:0.45; Pearson R:0.079
Boersma Breast : all
DLG3
BCL6
P−value:0.0051; Pearson R:0.29
Boersma Breast : all
SCRIB
ZBTB7A
P−value:0.14; Pearson R:−0.15
Boersma Breast : all
DLG3
ZBTB7A
P−value:2.4e−05; Pearson R:−0.42

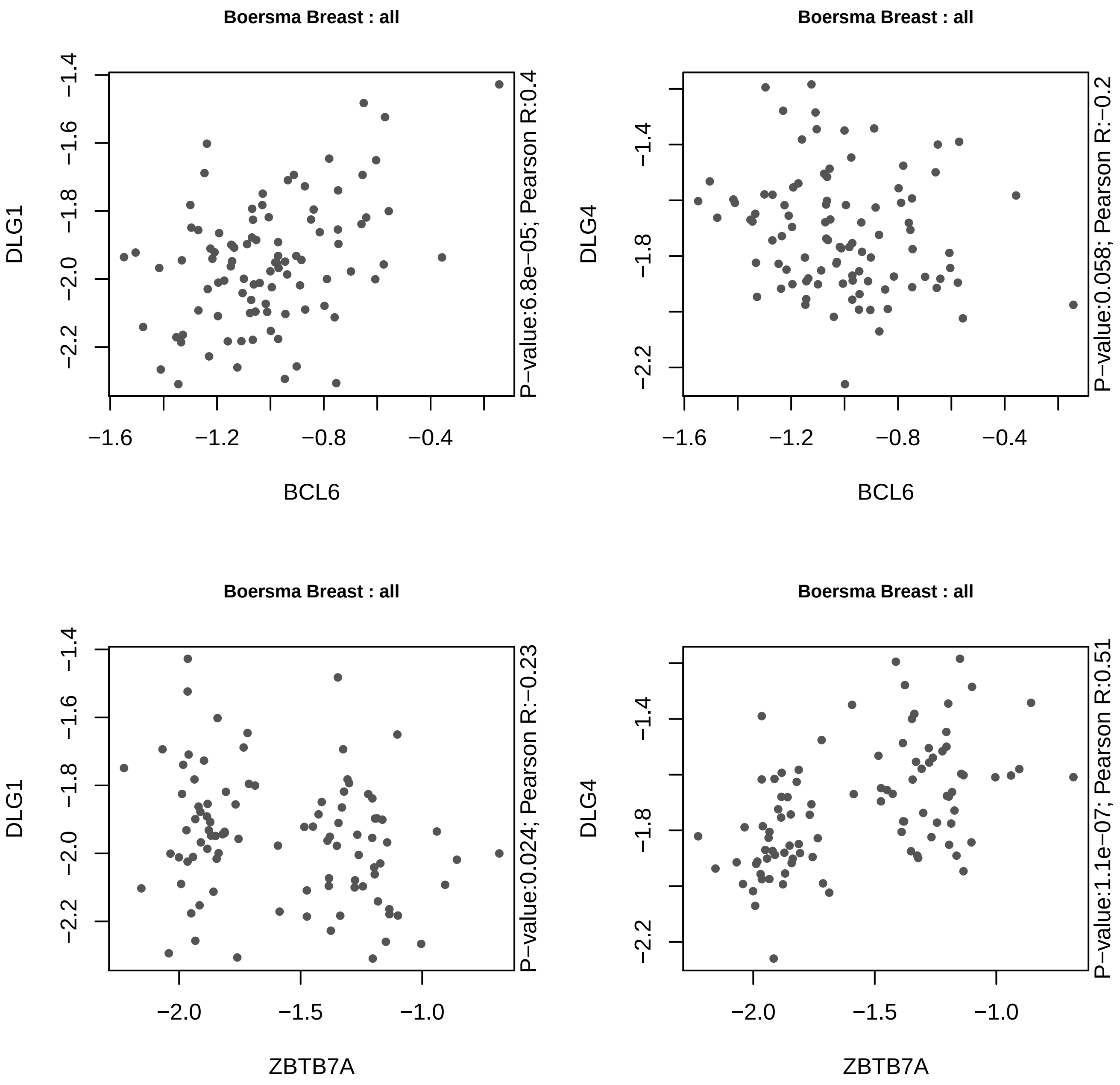
Boersma Breast : all
DLG1
BCL6
P−value:6.8e−05; Pearson R:0.4
−1.4
−1.6
−1.8
−2.0
−2.2
−1.6
−1.2
−0.8
−0.4
Boersma Breast : all
DLG4
BCL6
P−value:0.058; Pearson R:−0.2
−1.4
−1.8
−2.2
−1.6
−1.2
−0.8
−0.4
Boersma Breast : all
DLG1
ZBTB7A
P−value:0.024; Pearson R:−0.23
−1.4
−1.6
−1.8
−2.0
−2.2
−2.0
−1.5
−1.0
Boersma Breast : all
DLG4
ZBTB7A
P−value:1.1e−07; Pearson R:0.51
−1.4
−1.8
−2.2
−2.0
−1.5
−1.0

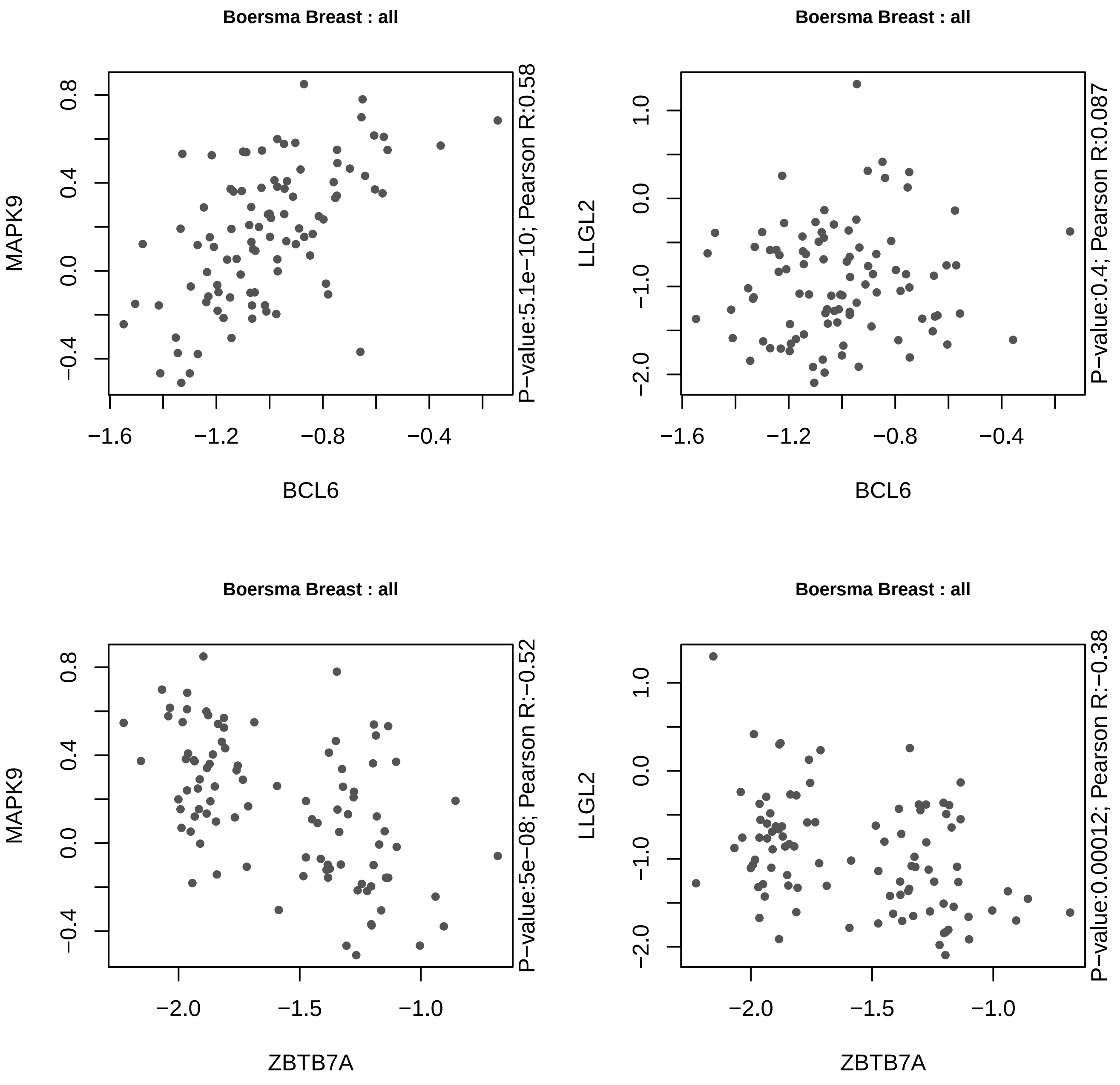
Boersma Breast : all
MAPK9
BCL6
P−value:5.1e−10; Pearson R:0.58
Boersma Breast : all
LLGL2
BCL6
P−value:0.4; Pearson R:0.087
Boersma Breast : all
MAPK9
ZBTB7A
P−value:5e−08; Pearson R:−0.52
Boersma Breast : all
LLGL2
ZBTB7A
P−value:0.00012; Pearson R:−0.38

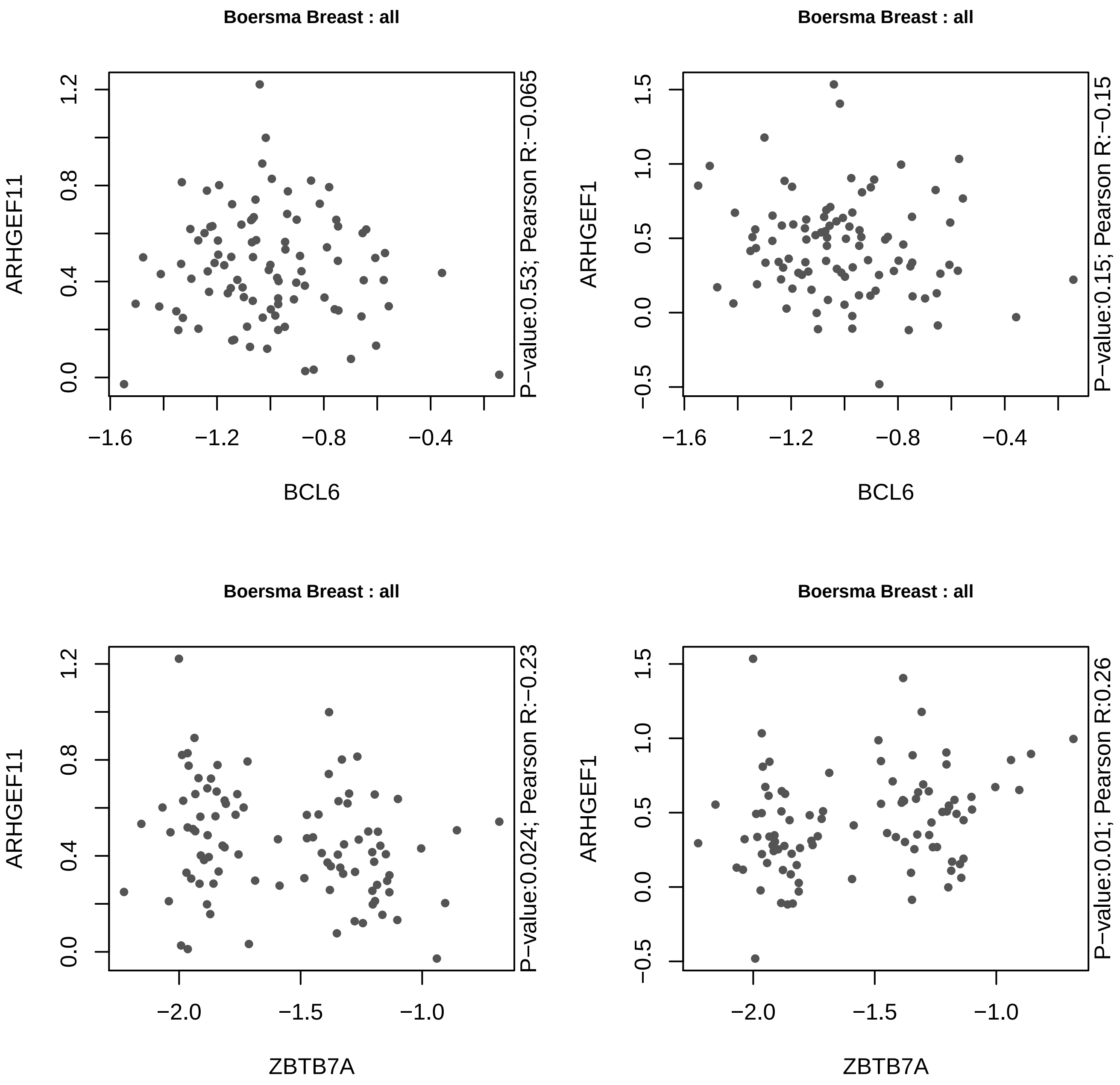
Boersma Breast : all
ARHGEF11
BCL6
P−value:0.53; Pearson R:−0.065
Boersma Breast : all
ARHGEF1
BCL6
P−value:0.15; Pearson R:−0.15
Boersma Breast : all
ARHGEF11
ZBTB7A
P−value:0.024; Pearson R:−0.23
Boersma Breast : all
ARHGEF1
ZBTB7A
P−value:0.01; Pearson R:0.26

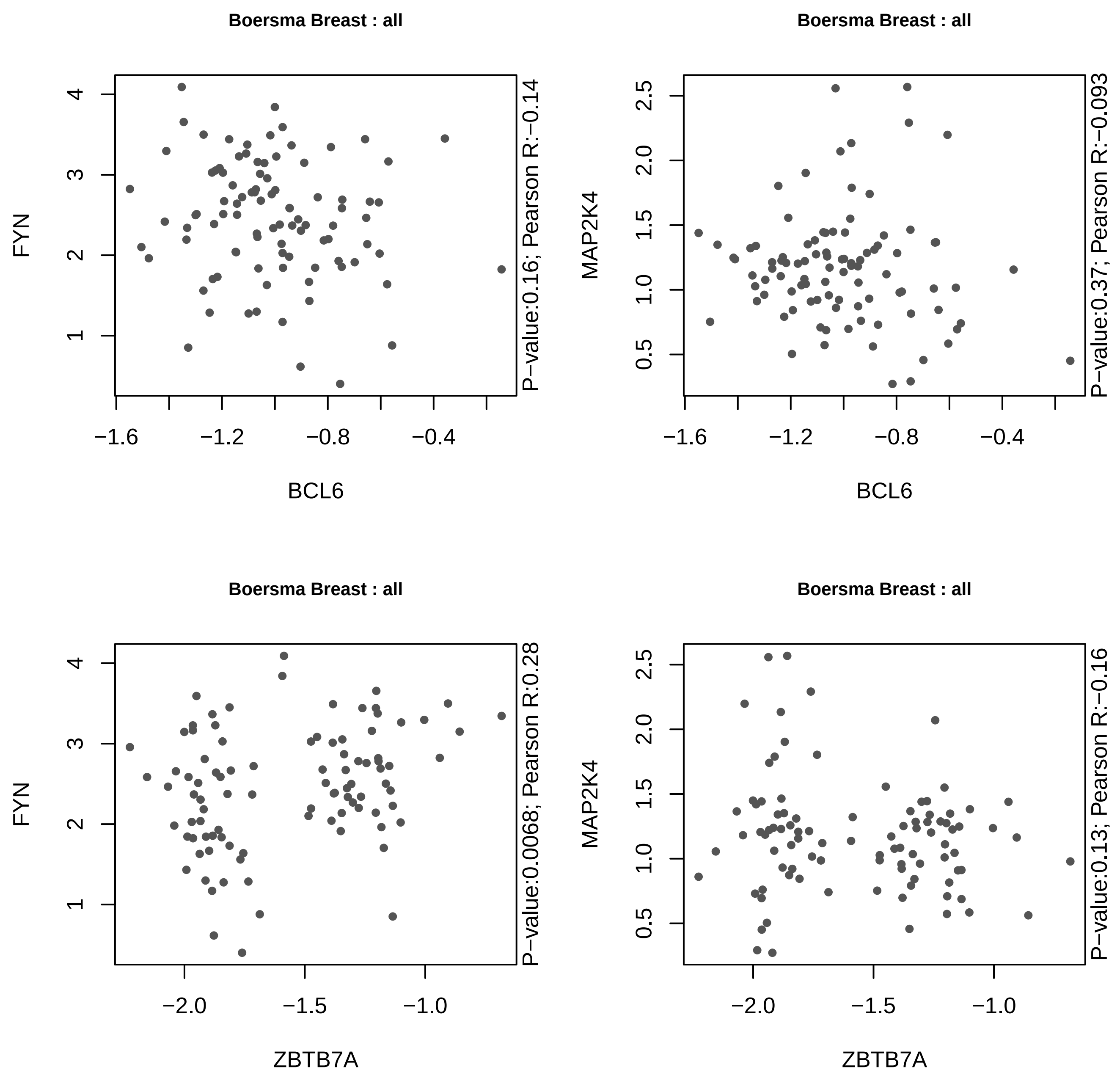
Boersma Breast : all
FYN
BCL6
P−value:0.16; Pearson R:−0.14
Boersma Breast : all
MAP2K4
BCL6
P−value:0.37; Pearson R:−0.093
Boersma Breast : all
FYN
ZBTB7A
P−value:0.0068; Pearson R:0.28
Boersma Breast : all
MAP2K4
ZBTB7A
P−value:0.13; Pearson R:−0.16

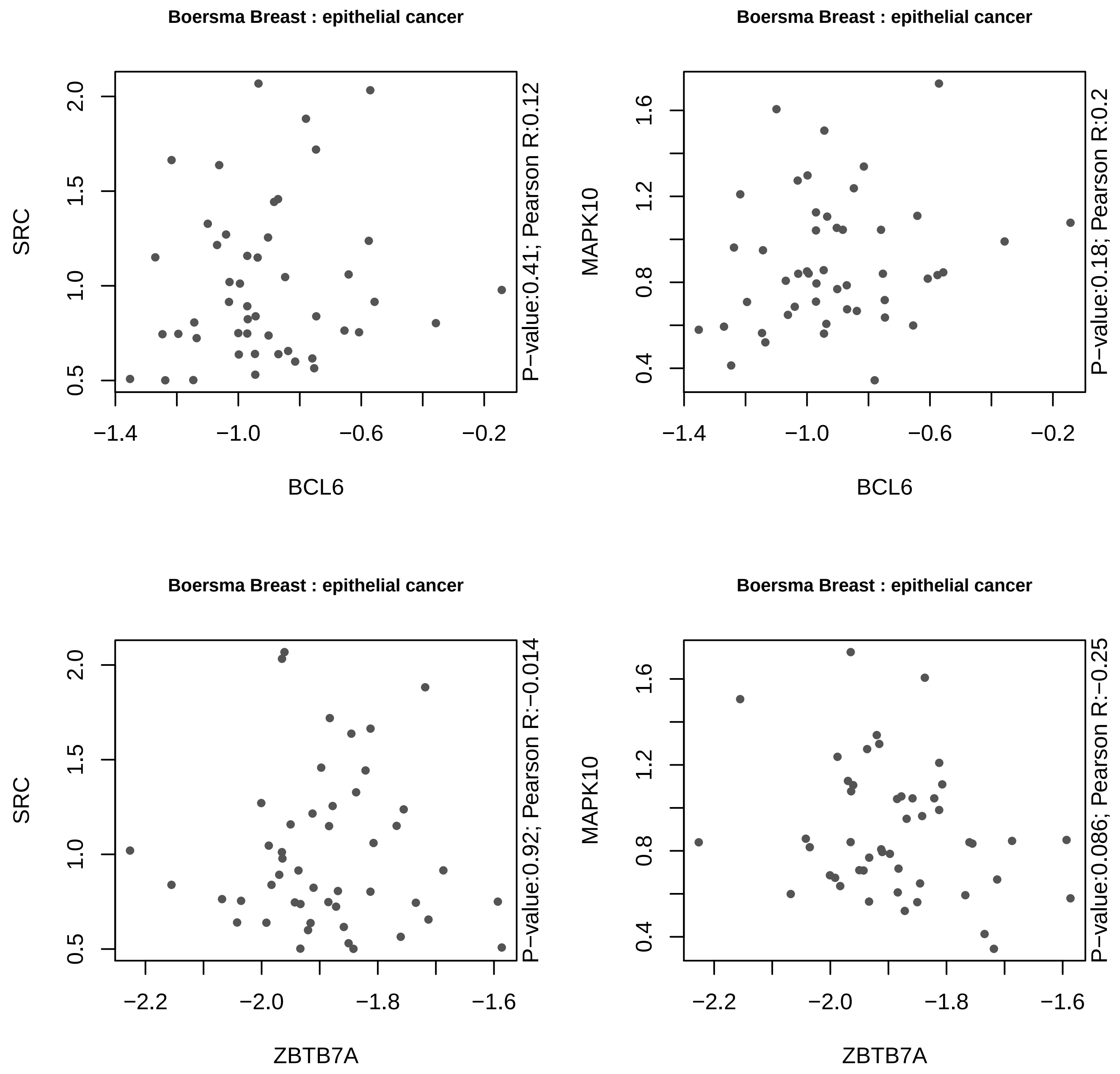
Boersma Breast : epithelial cancer
SRC
BCL6
P−value:0.41; Pearson R:0.12
Boersma Breast : epithelial cancer
MAPK10
BCL6
P−value:0.18; Pearson R:0.2
Boersma Breast : epithelial cancer
SRC
ZBTB7A
P−value:0.92; Pearson R:−0.014
Boersma Breast : epithelial cancer
MAPK10
ZBTB7A
P−value:0.086; Pearson R:−0.25

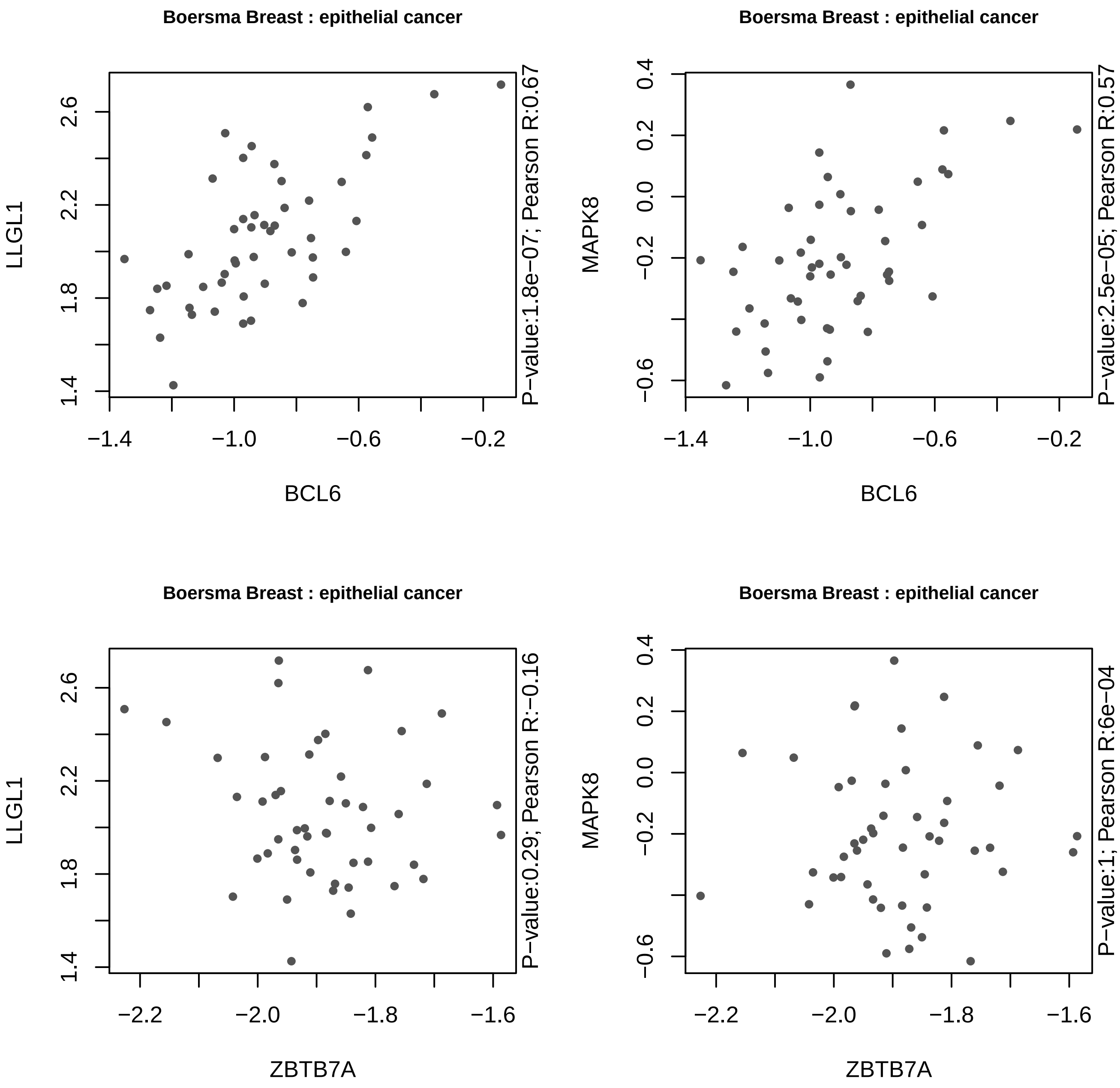
Boersma Breast : epithelial cancer
LLGL1
BCL6
P−value:1.8e−07; Pearson R:0.67
Boersma Breast : epithelial cancer
MAPK8
BCL6
P−value:2.5e−05; Pearson R:0.57
Boersma Breast : epithelial cancer
LLGL1
ZBTB7A
P−value:0.29; Pearson R:−0.16
Boersma Breast : epithelial cancer
MAPK8
ZBTB7A
P−value:1; Pearson R:6e−04

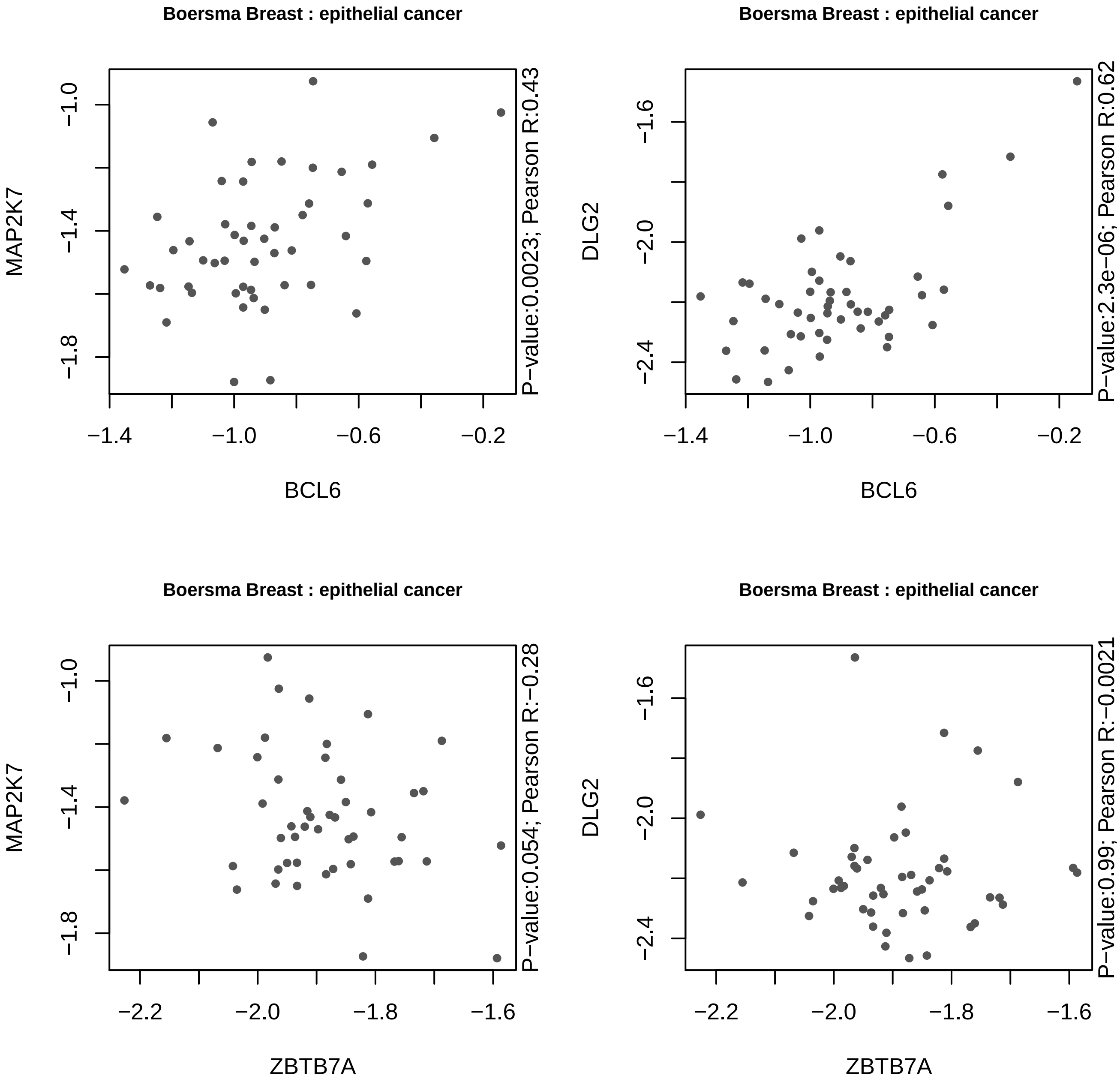
Boersma Breast : epithelial cancer
MAP2K7
BCL6
P−value:0.0023; Pearson R:0.43
Boersma Breast : epithelial cancer
DLG2
BCL6
P−value:2.3e−06; Pearson R:0.62
Boersma Breast : epithelial cancer
MAP2K7
ZBTB7A
P−value:0.054; Pearson R:−0.28
Boersma Breast : epithelial cancer
DLG2
ZBTB7A
P−value:0.99; Pearson R:−0.0021

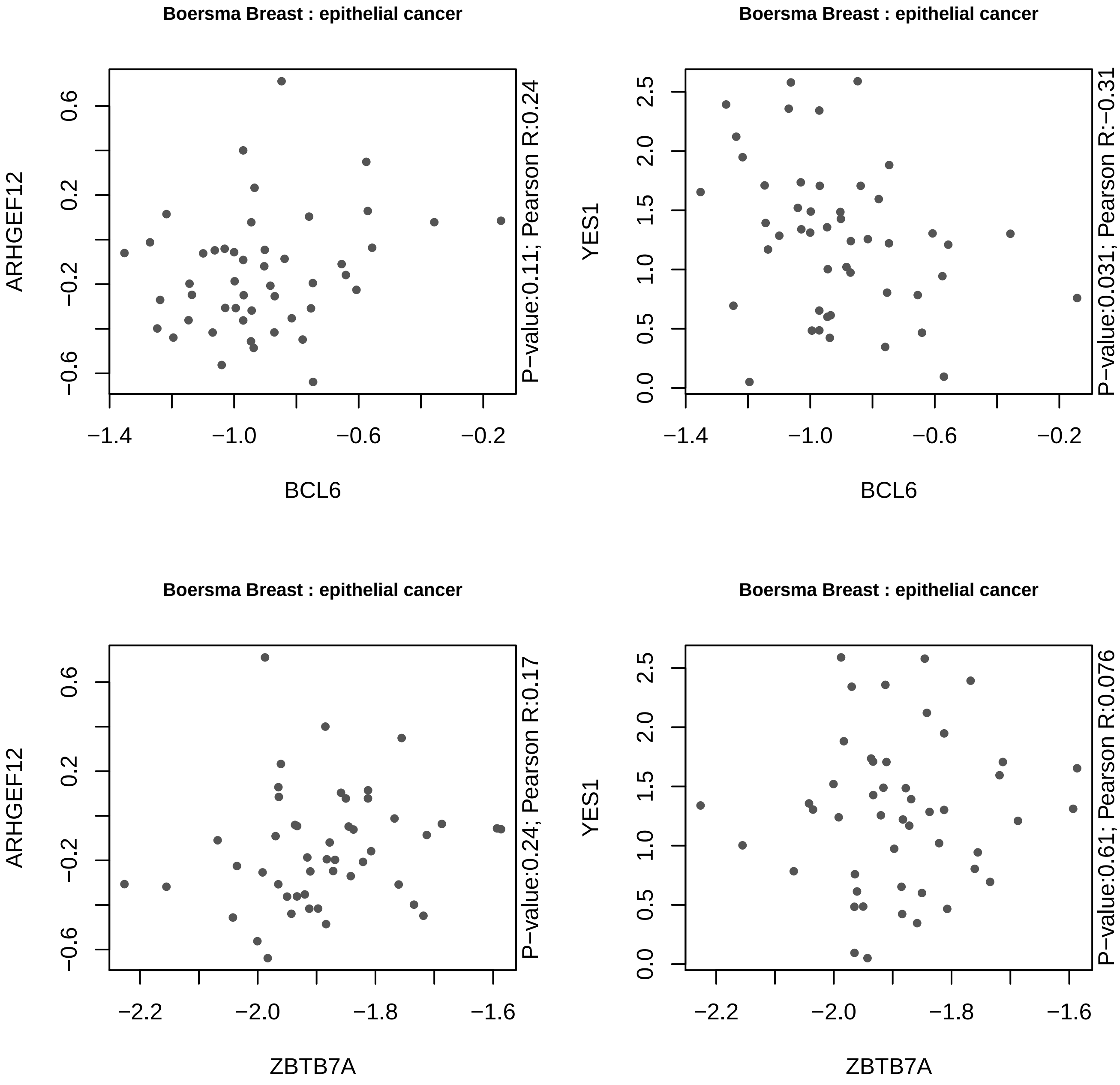
Boersma Breast : epithelial cancer
ARHGEF12
BCL6
P−value:0.11; Pearson R:0.24
Boersma Breast : epithelial cancer
YES1
BCL6
P−value:0.031; Pearson R:−0.31
Boersma Breast : epithelial cancer
ARHGEF12
ZBTB7A
P−value:0.24; Pearson R:0.17
Boersma Breast : epithelial cancer
YES1
ZBTB7A
P−value:0.61; Pearson R:0.076

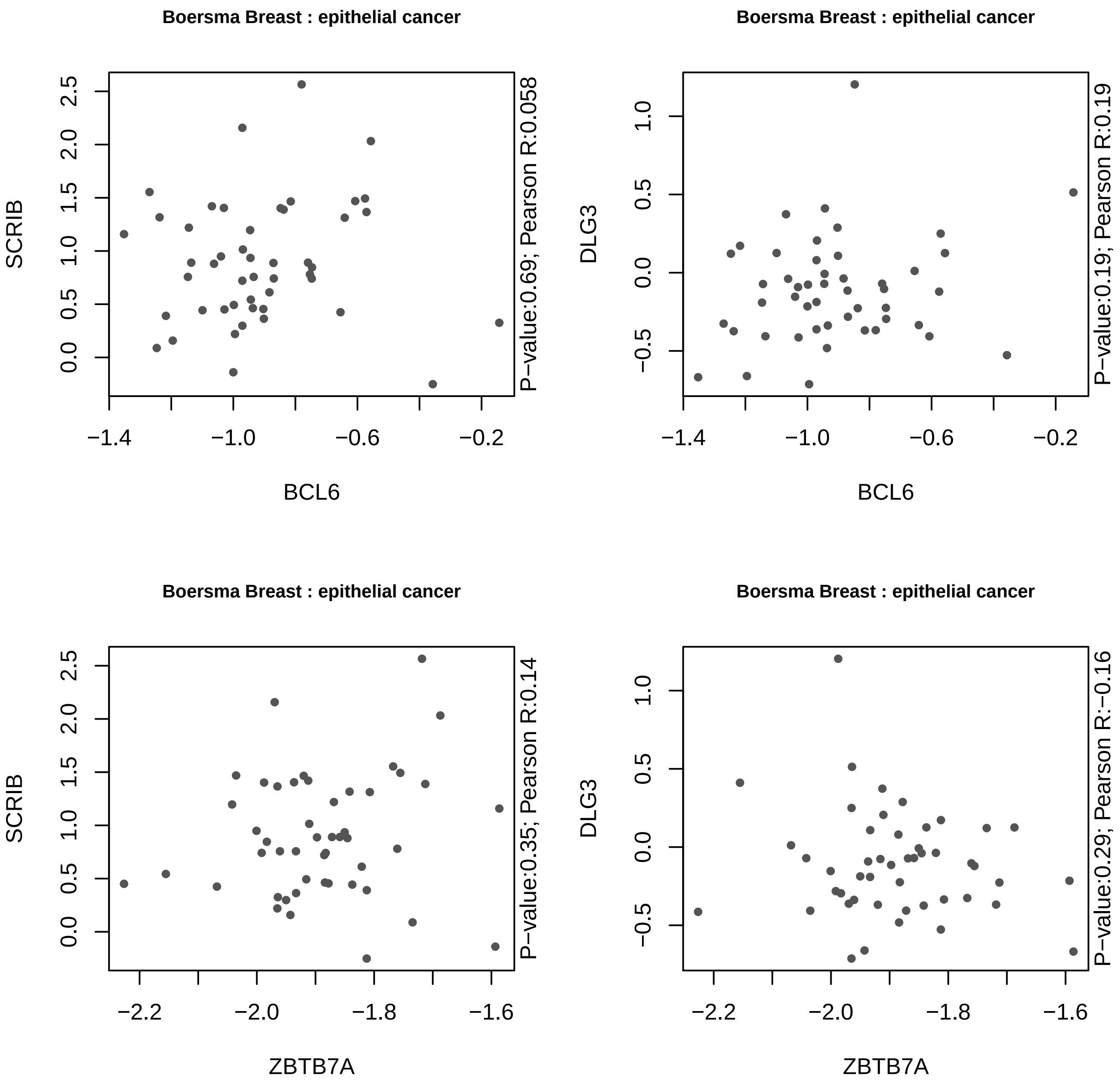
Boersma Breast : epithelial cancer
SCRIB
BCL6
P−value:0.69; Pearson R:0.058
Boersma Breast : epithelial cancer
DLG3
BCL6
P−value:0.19; Pearson R:0.19
Boersma Breast : epithelial cancer
SCRIB
ZBTB7A
P−value:0.35; Pearson R:0.14
Boersma Breast : epithelial cancer
DLG3
ZBTB7A
P−value:0.29; Pearson R:−0.16

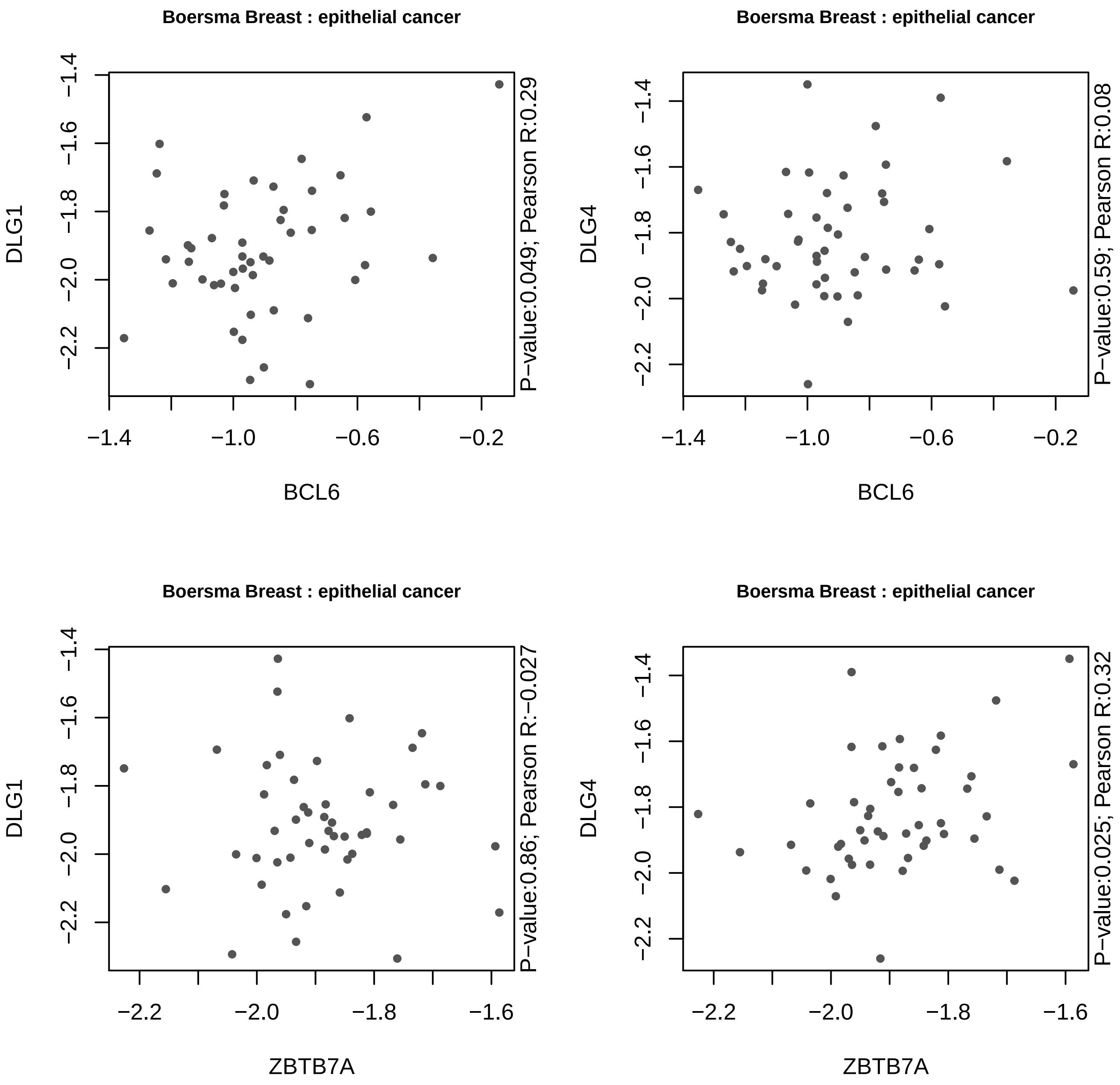
Boersma Breast : epithelial cancer
DLG1
BCL6
P−value:0.049; Pearson R:0.29
Boersma Breast : epithelial cancer
DLG4
BCL6
P−value:0.59; Pearson R:0.08
Boersma Breast : epithelial cancer
DLG1
ZBTB7A
P−value:0.86; Pearson R:−0.027
Boersma Breast : epithelial cancer
DLG4
ZBTB7A
P−value:0.025; Pearson R:0.32

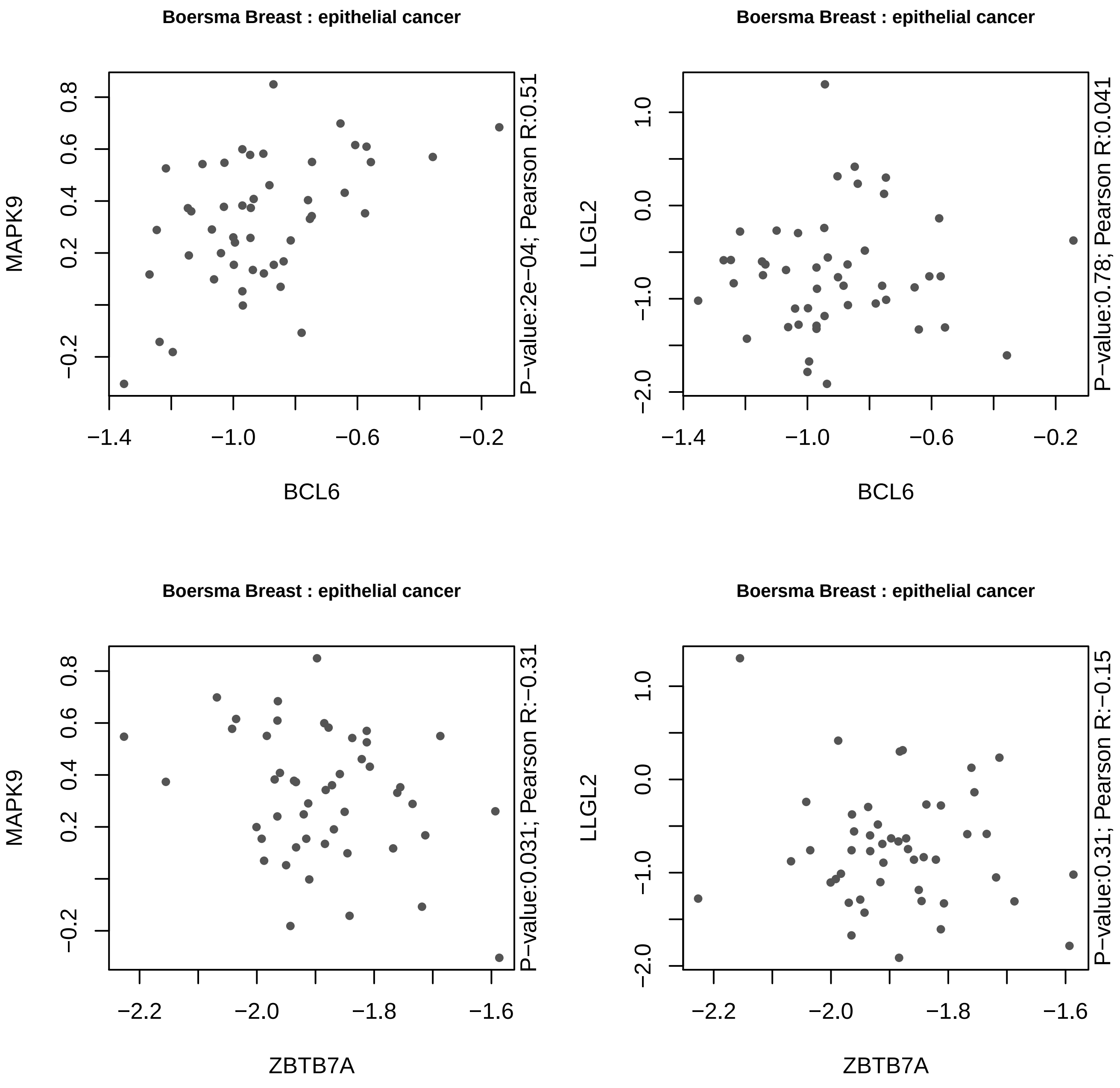
Boersma Breast : epithelial cancer
MAPK9
BCL6
P−value:2e−04; Pearson R:0.51
Boersma Breast : epithelial cancer
LLGL2
BCL6
P−value:0.78; Pearson R:0.041
Boersma Breast : epithelial cancer
MAPK9
ZBTB7A
P−value:0.031; Pearson R:−0.31
Boersma Breast : epithelial cancer
LLGL2
ZBTB7A
P−value:0.31; Pearson R:−0.15

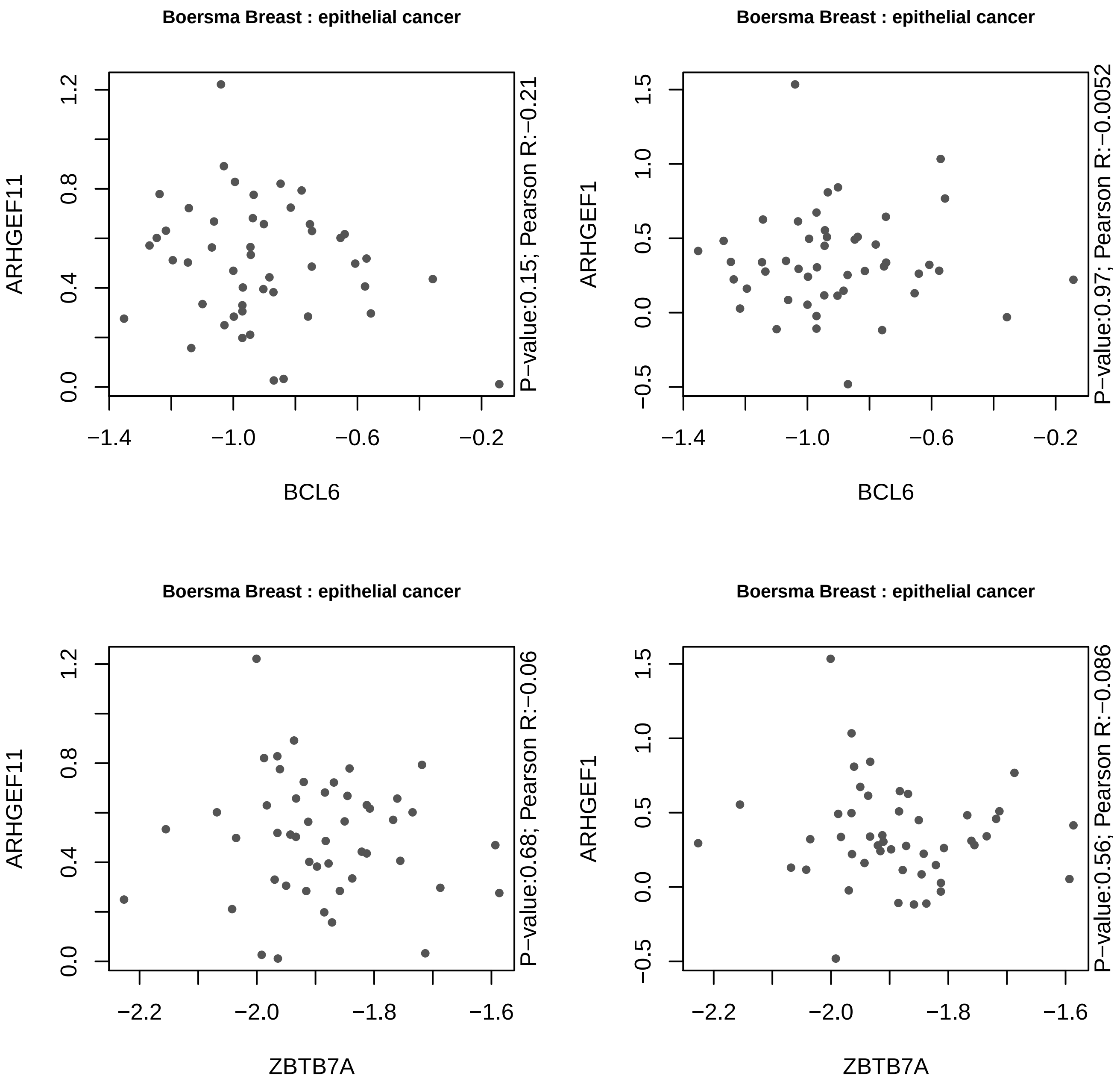
Boersma Breast : epithelial cancer
ARHGEF11
BCL6
P−value:0.15; Pearson R:−0.21
Boersma Breast : epithelial cancer
ARHGEF1
BCL6
P−value:0.97; Pearson R:−0.0052
Boersma Breast : epithelial cancer
ARHGEF11
ZBTB7A
P−value:0.68; Pearson R:−0.06
Boersma Breast : epithelial cancer
ARHGEF1
ZBTB7A
P−value:0.56; Pearson R:−0.086

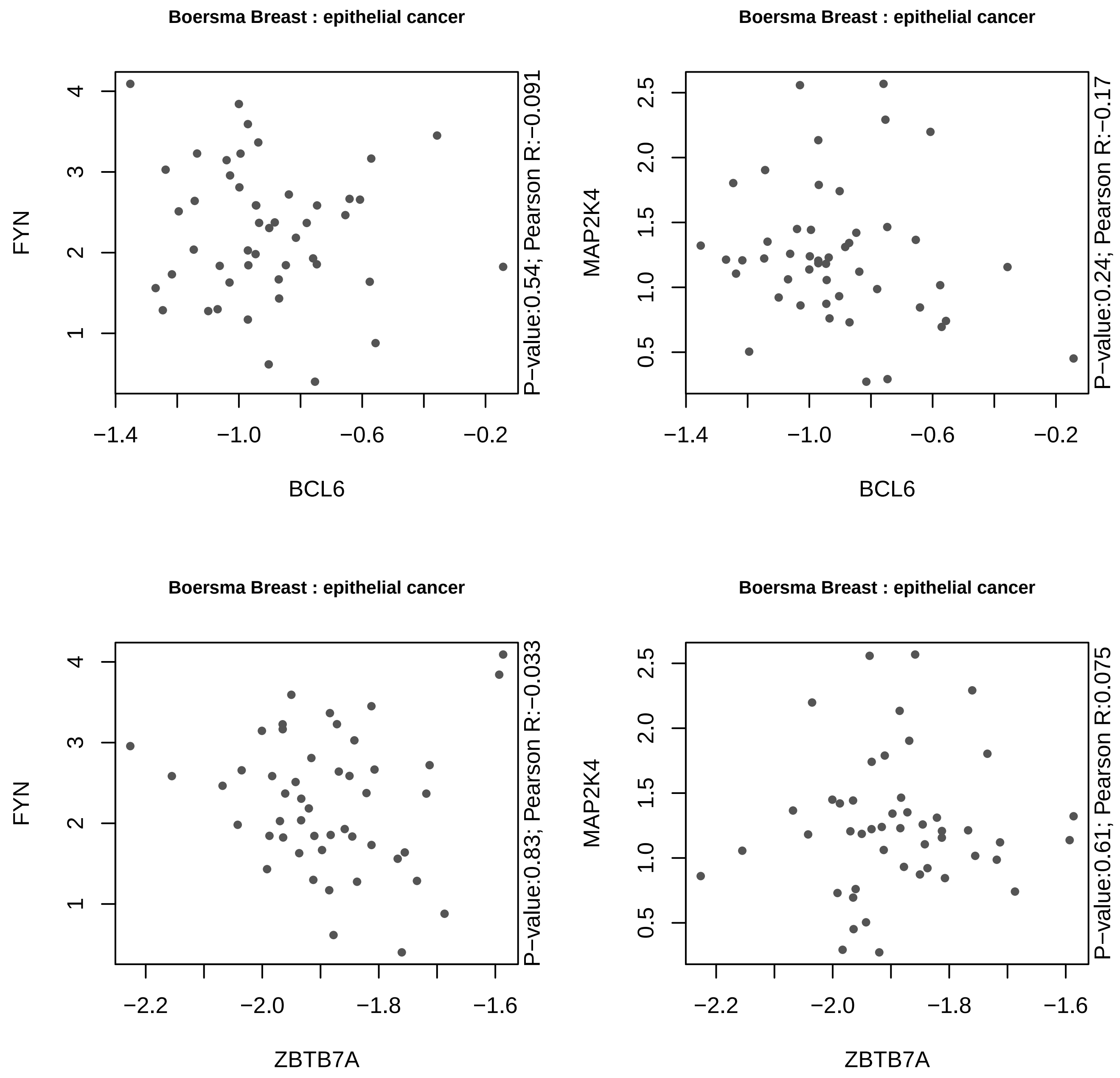

Boersma Breast : epithelial cancer
FYN
BCL6
P−value:0.54; Pearson R:−0.091
Boersma Breast : epithelial cancer
MAP2K4
BCL6
P−value:0.24; Pearson R:−0.17
Boersma Breast : epithelial cancer
FYN
ZBTB7A
P−value:0.83; Pearson R:−0.033
Boersma Breast : epithelial cancer
MAP2K4
ZBTB7A
P−value:0.61; Pearson R:0.075

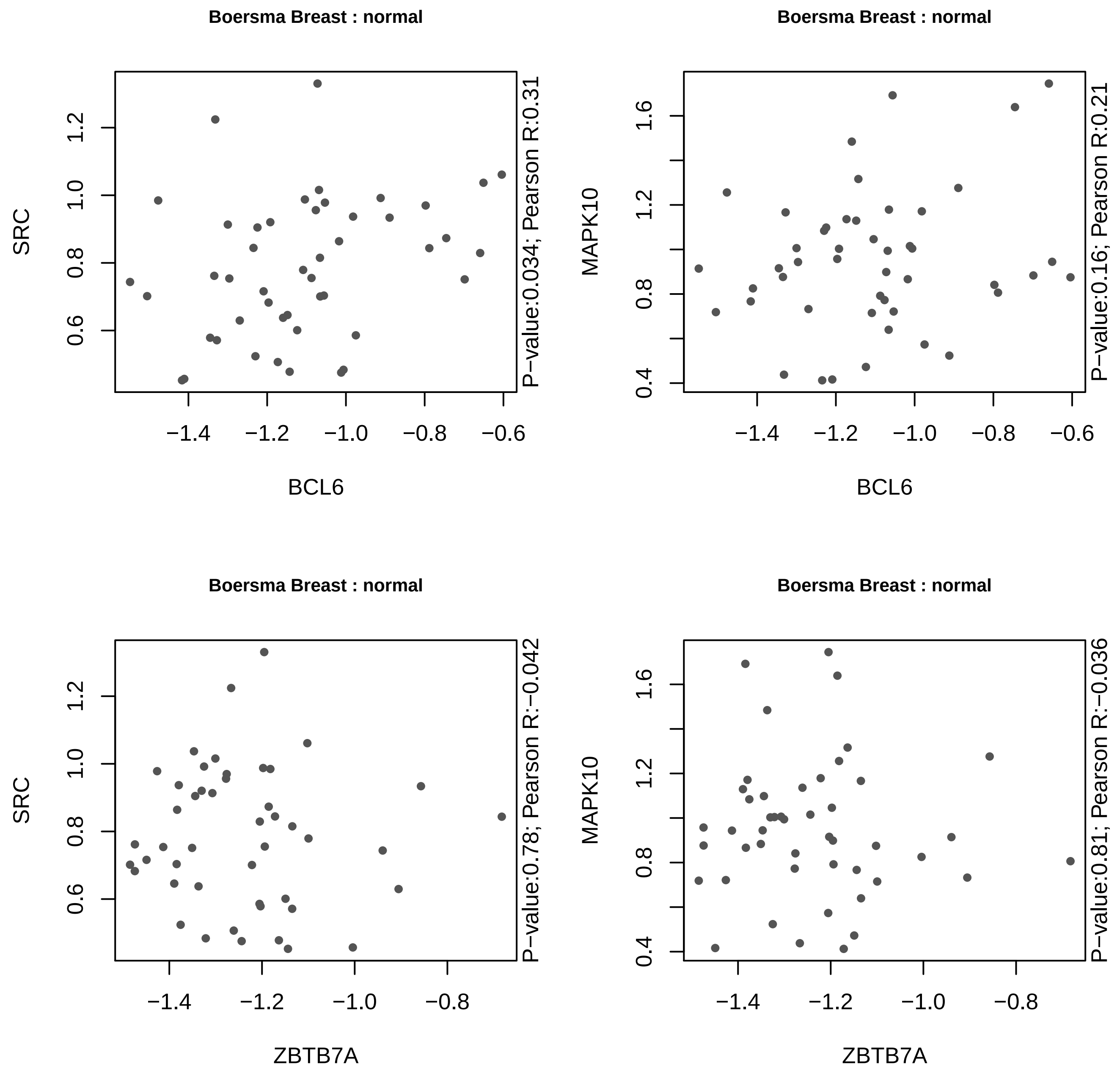
Boersma Breast : normal
SRC
BCL6
P−value:0.034; Pearson R:0.31
Boersma Breast : normal
MAPK10
BCL6
P−value:0.16; Pearson R:0.21
Boersma Breast : normal
SRC
ZBTB7A
P−value:0.78; Pearson R:−0.042
Boersma Breast : normal
MAPK10
ZBTB7A
P−value:0.81; Pearson R:−0.036

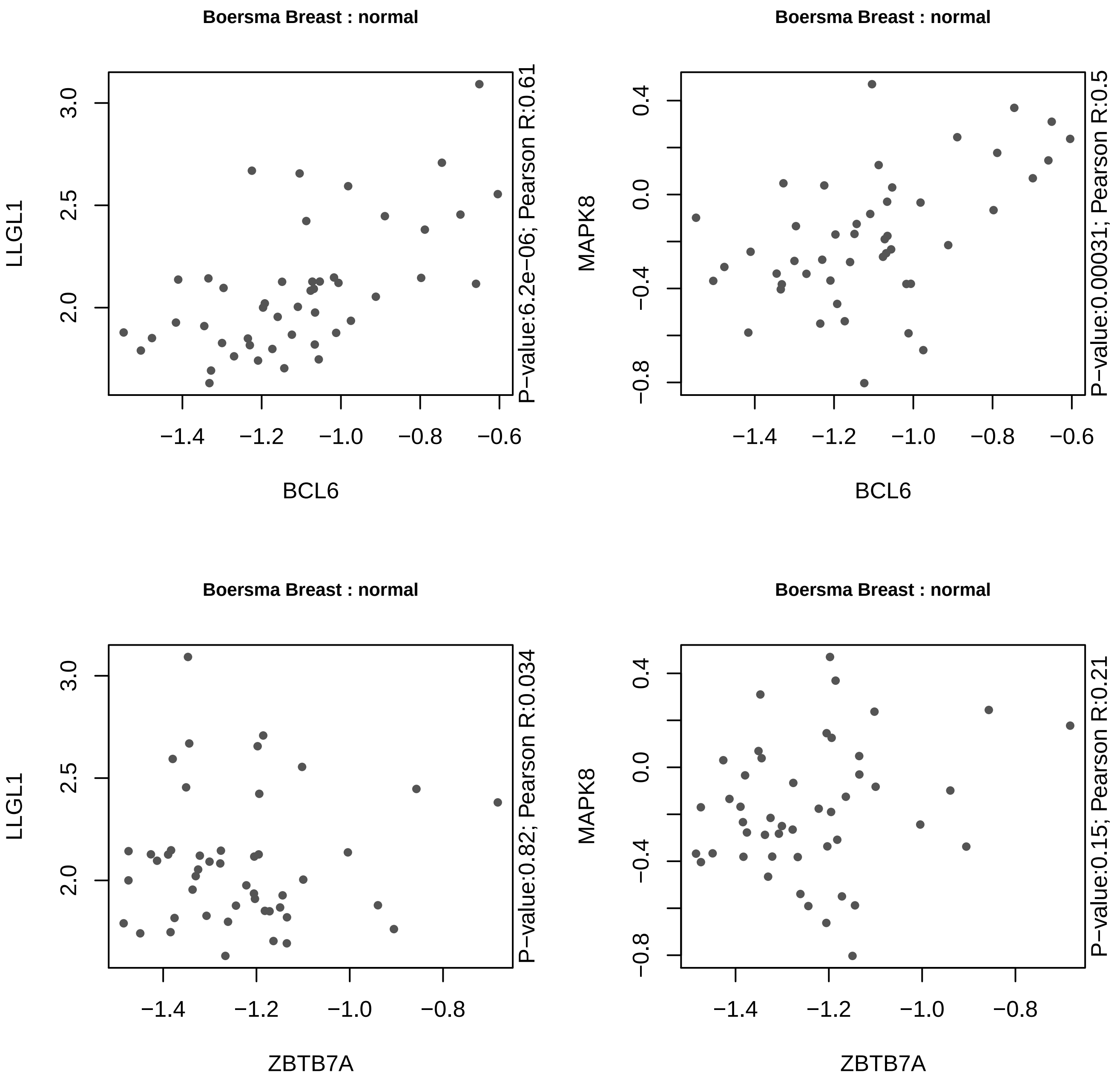

Boersma Breast : normal
LLGL1
BCL6
P−value:6.2e−06; Pearson R:0.61
Boersma Breast : normal
MAPK8
BCL6
P−value:0.00031; Pearson R:0.5
Boersma Breast : normal
LLGL1
ZBTB7A
P−value:0.82; Pearson R:0.034
Boersma Breast : normal
MAPK8
ZBTB7A
P−value:0.15; Pearson R:0.21

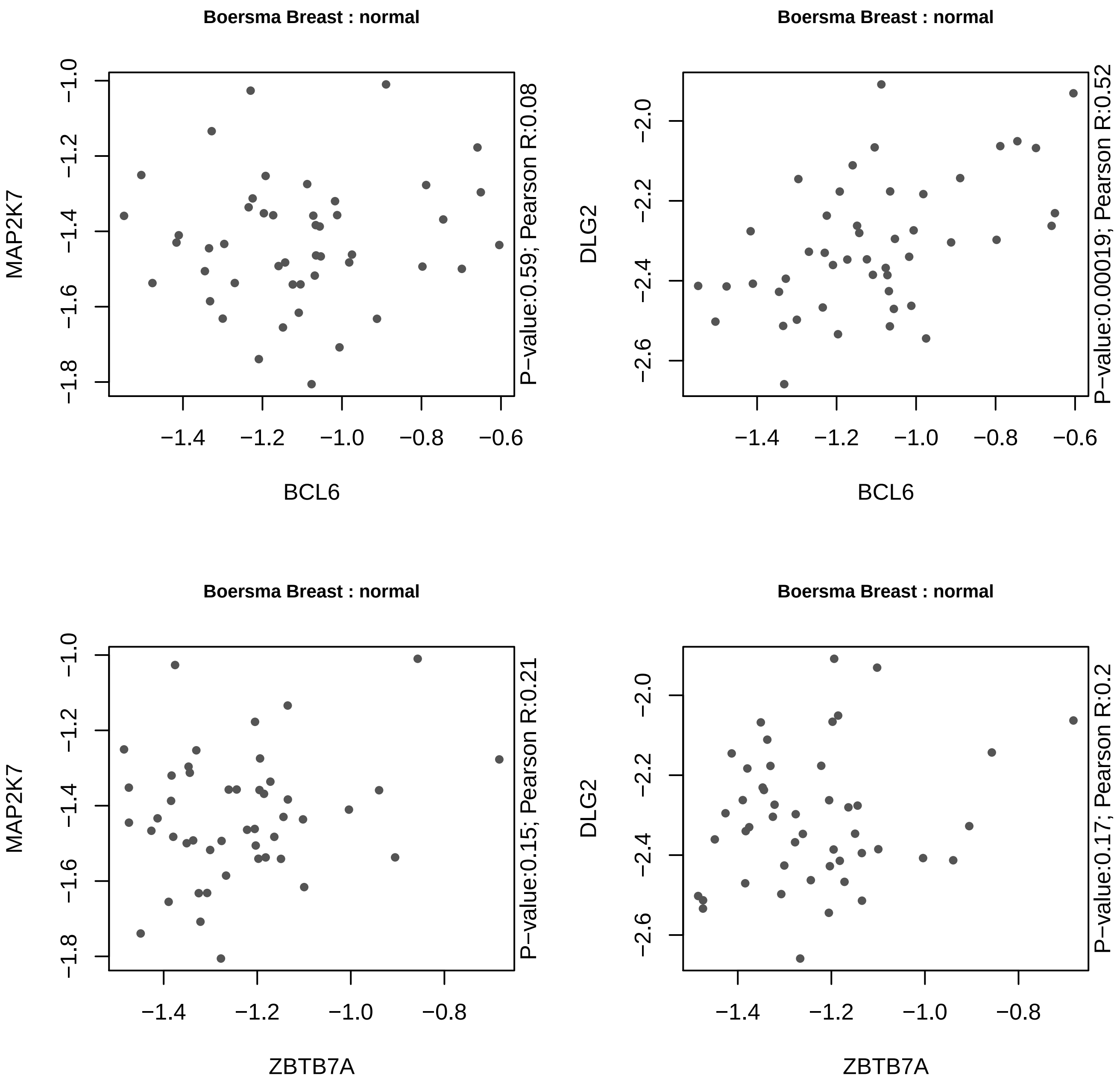
Boersma Breast : normal
MAP2K7
BCL6
P−value:0.59; Pearson R:0.08
Boersma Breast : normal
DLG2
BCL6
P−value:0.00019; Pearson R:0.52
Boersma Breast : normal
MAP2K7
ZBTB7A
P−value:0.15; Pearson R:0.21
Boersma Breast : normal
DLG2
ZBTB7A
P−value:0.17; Pearson R:0.2

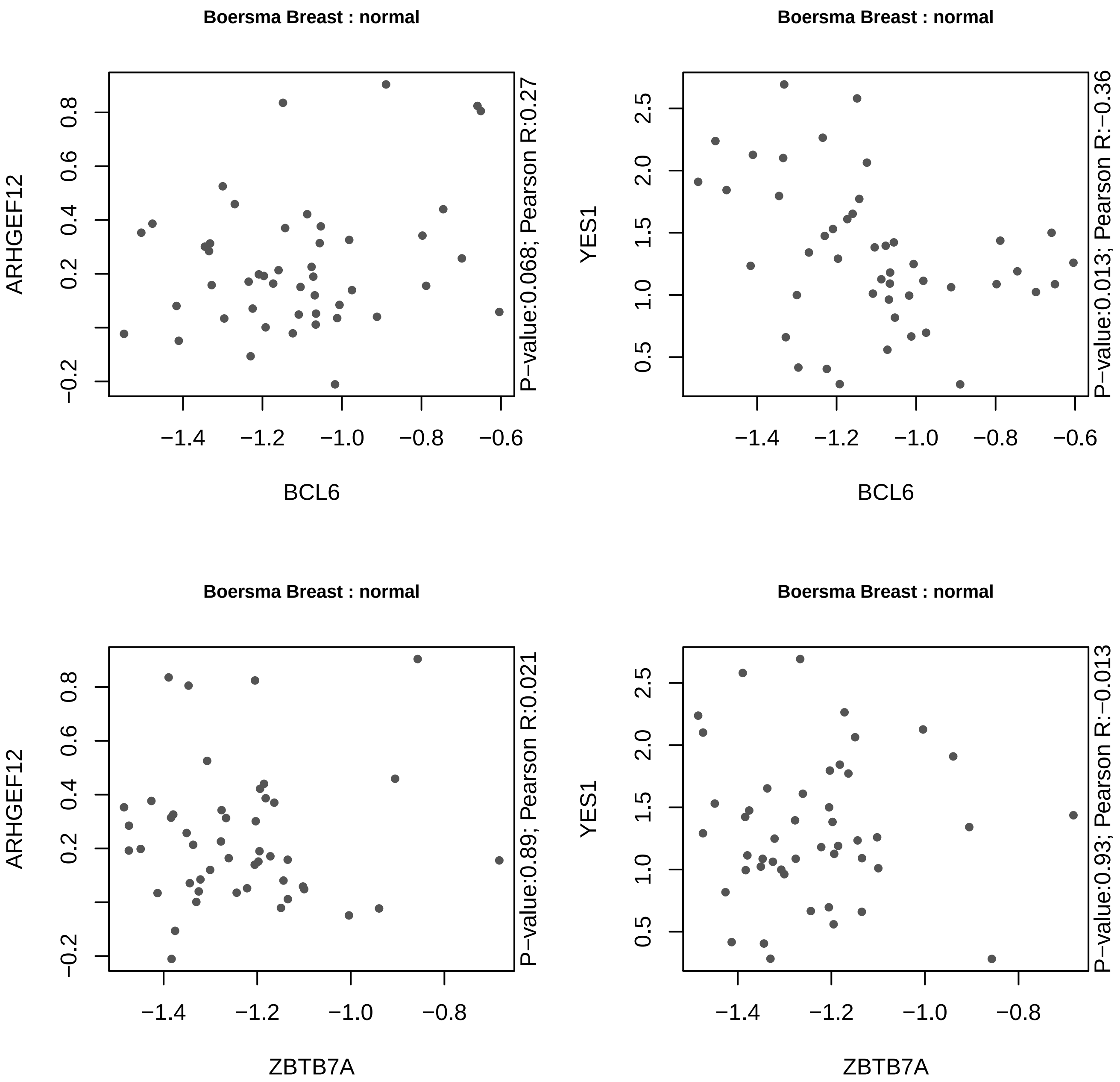
Boersma Breast : normal
ARHGEF12
BCL6
P−value:0.068; Pearson R:0.27
Boersma Breast : normal
YES1
BCL6
P−value:0.013; Pearson R:−0.36
Boersma Breast : normal
ARHGEF12
ZBTB7A
P−value:0.89; Pearson R:0.021
Boersma Breast : normal
YES1
ZBTB7A
P−value:0.93; Pearson R:−0.013

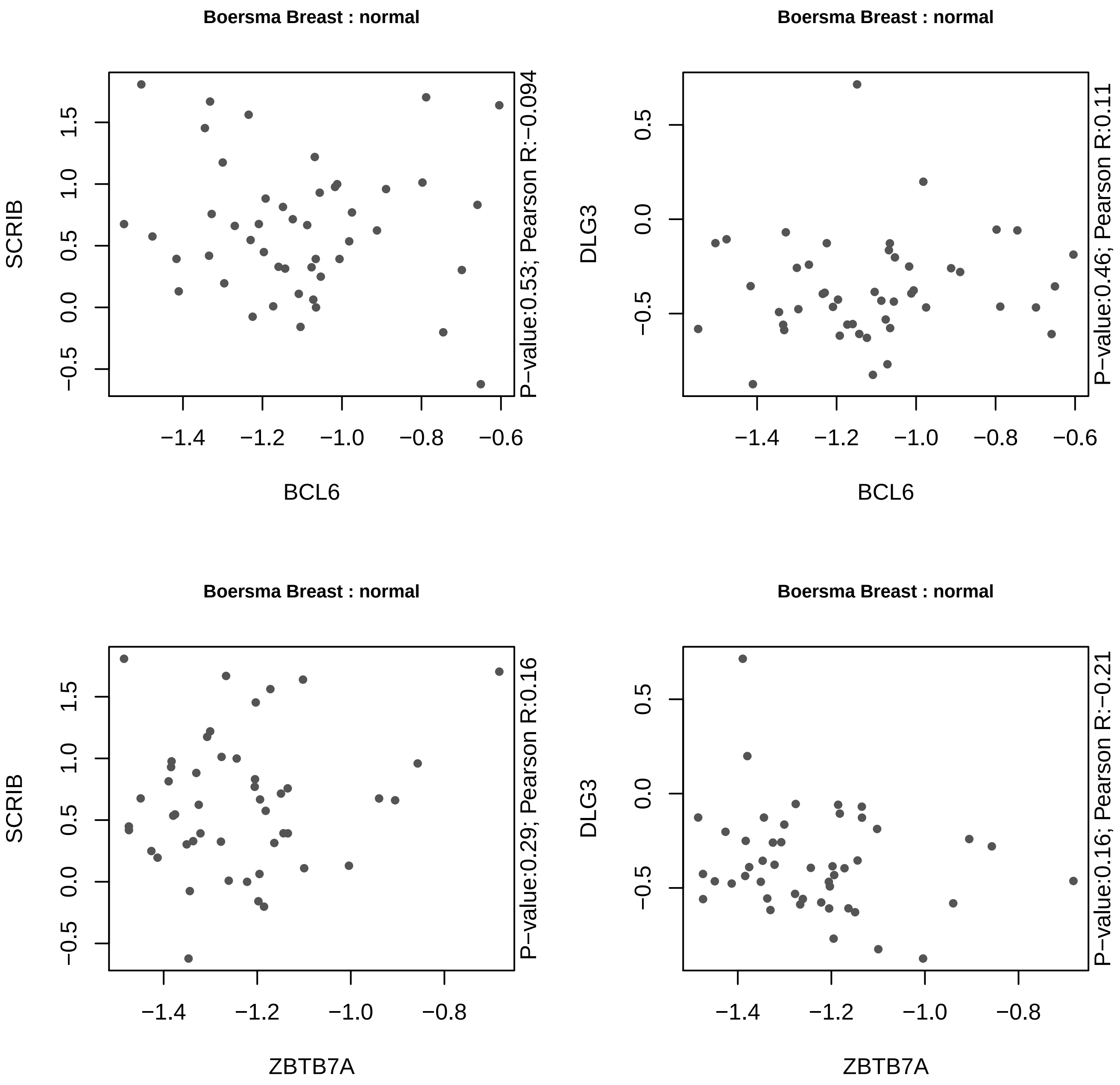
Boersma Breast : normal
SCRIB
BCL6
P−value:0.53; Pearson R:−0.094
Boersma Breast : normal
DLG3
BCL6
P−value:0.46; Pearson R:0.11
Boersma Breast : normal
SCRIB
ZBTB7A
P−value:0.29; Pearson R:0.16
Boersma Breast : normal
DLG3
ZBTB7A
P−value:0.16; Pearson R:−0.21

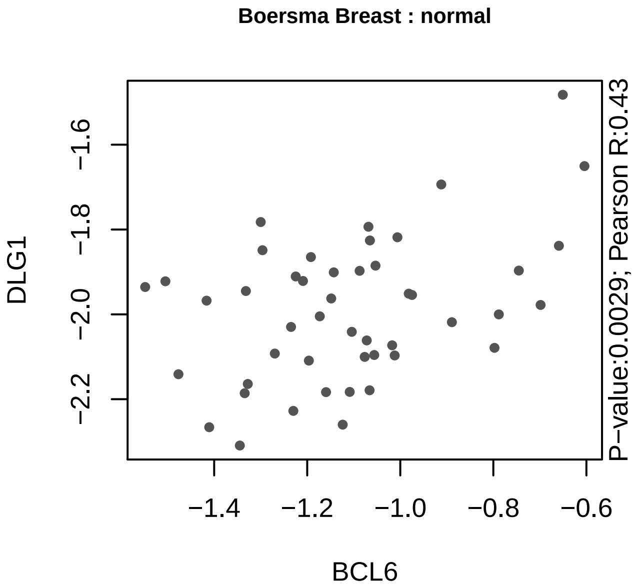
Boersma Breast : normal
DLG1
BCL6
P−value:0.0029; Pearson R:0.43
−1.6
−1.8
−2.0
−2.2
−1.4
−1.2
−1.0
−0.8
−0.6

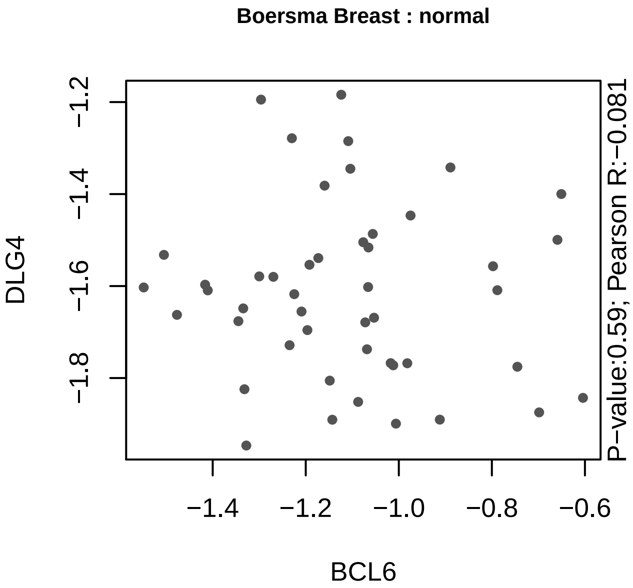
Boersma Breast : normal
DLG4
BCL6
P−value:0.59; Pearson R:−0.081
−1.2
−1.4
−1.6
−1.8
−1.4
−1.2
−1.0
−0.8
−0.6

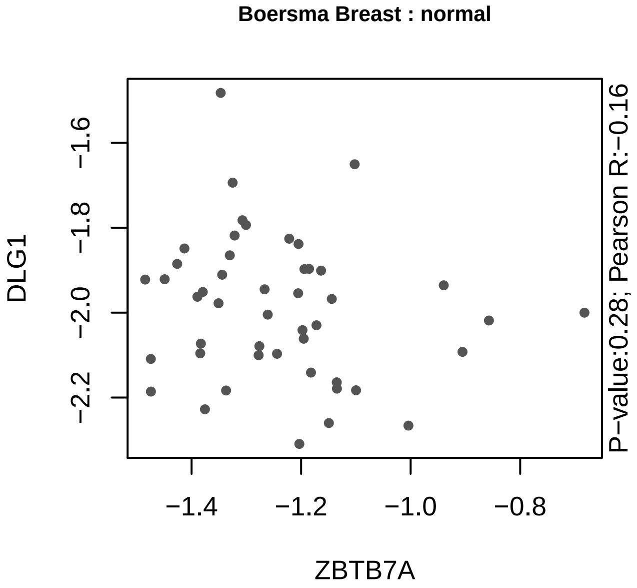
Boersma Breast : normal
DLG1
ZBTB7A
P−value:0.28; Pearson R:−0.16
−1.6
−1.8
−2.0
−2.2
−1.4
−1.2
−1.0
−0.8

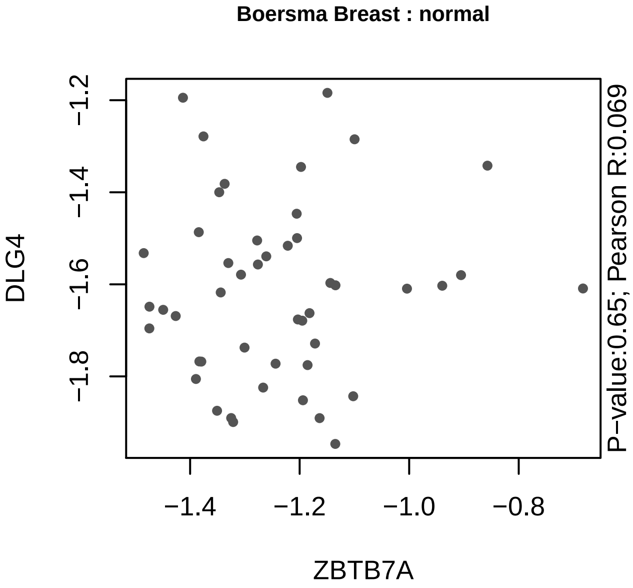
Boersma Breast : normal
DLG4
ZBTB7A
P−value:0.65; Pearson R:0.069
−1.2
−1.4
−1.6
−1.8
−1.4
−1.2
−1.0
−0.8

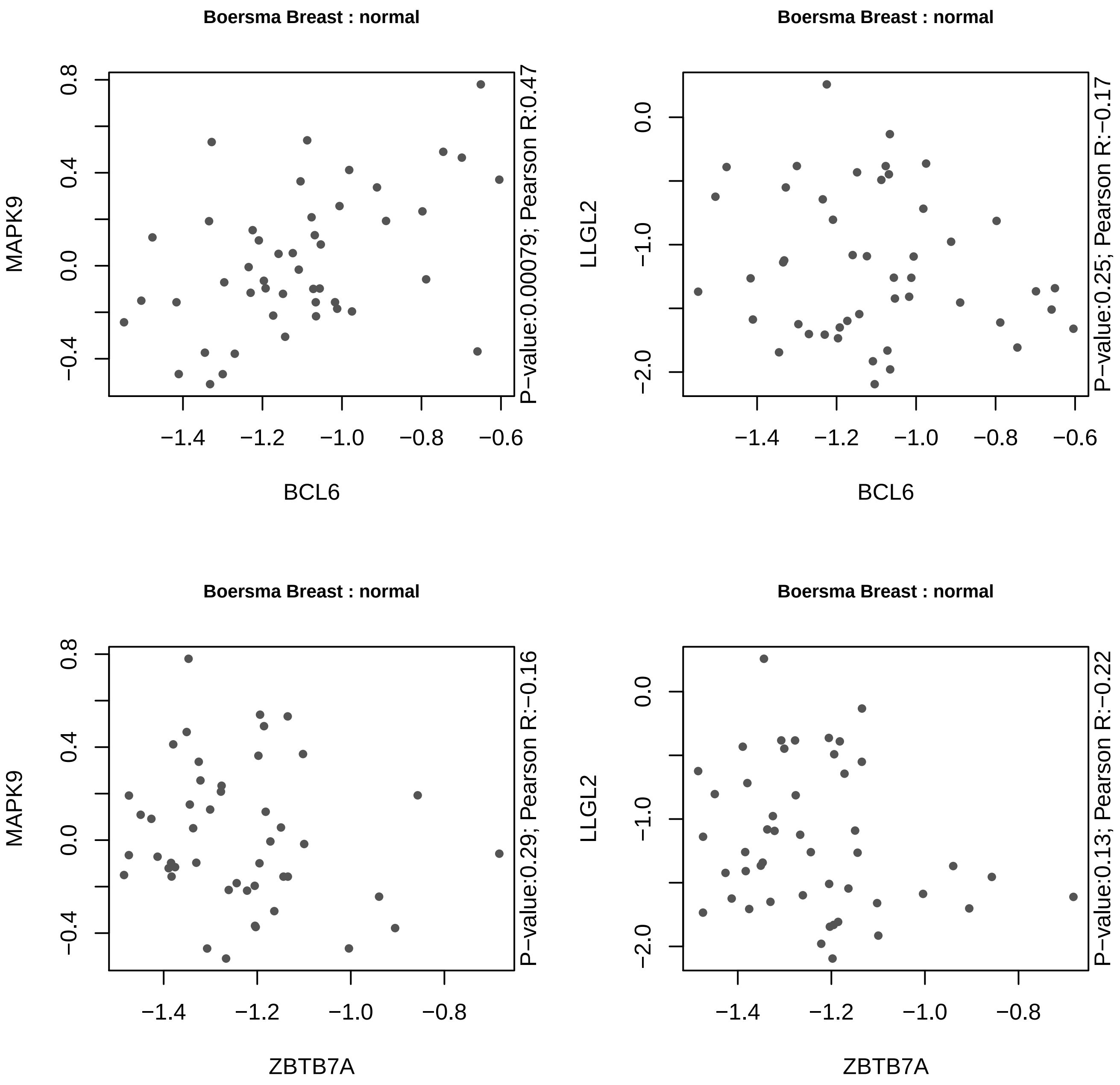
Boersma Breast : normal
MAPK9
BCL6
P−value:0.00079; Pearson R:0.47
Boersma Breast : normal
LLGL2
BCL6
P−value:0.25; Pearson R:−0.17
Boersma Breast : normal
MAPK9
ZBTB7A
P−value:0.29; Pearson R:−0.16
Boersma Breast : normal
LLGL2
ZBTB7A
P−value:0.13; Pearson R:−0.22

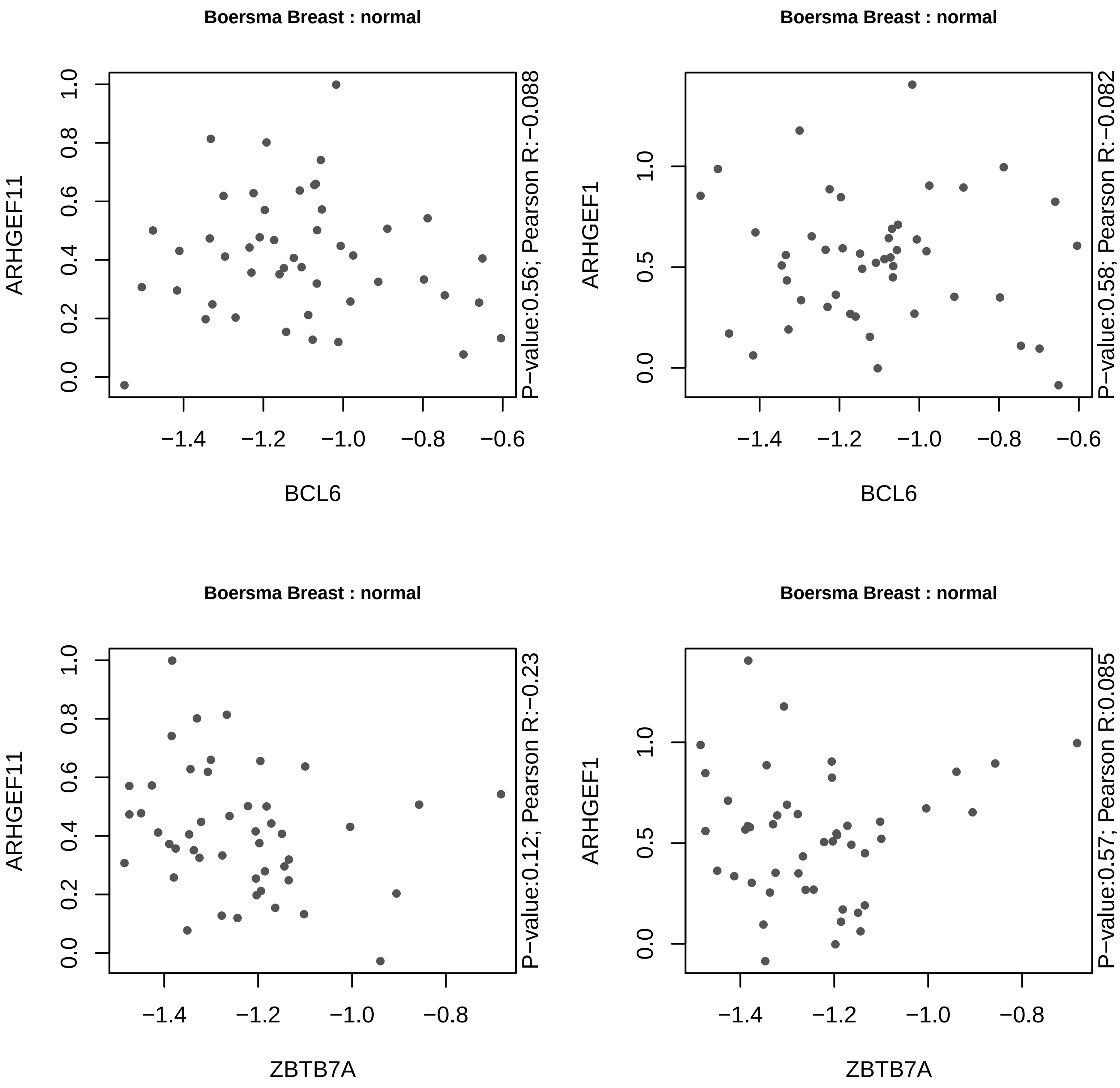
Boersma Breast : normal
ARHGEF11
BCL6
P−value:0.56; Pearson R:−0.088
Boersma Breast : normal
ARHGEF1
BCL6
P−value:0.58; Pearson R:−0.082
Boersma Breast : normal
ARHGEF11
ZBTB7A
P−value:0.12; Pearson R:−0.23
Boersma Breast : normal
ARHGEF1
ZBTB7A
P−value:0.57; Pearson R:0.085

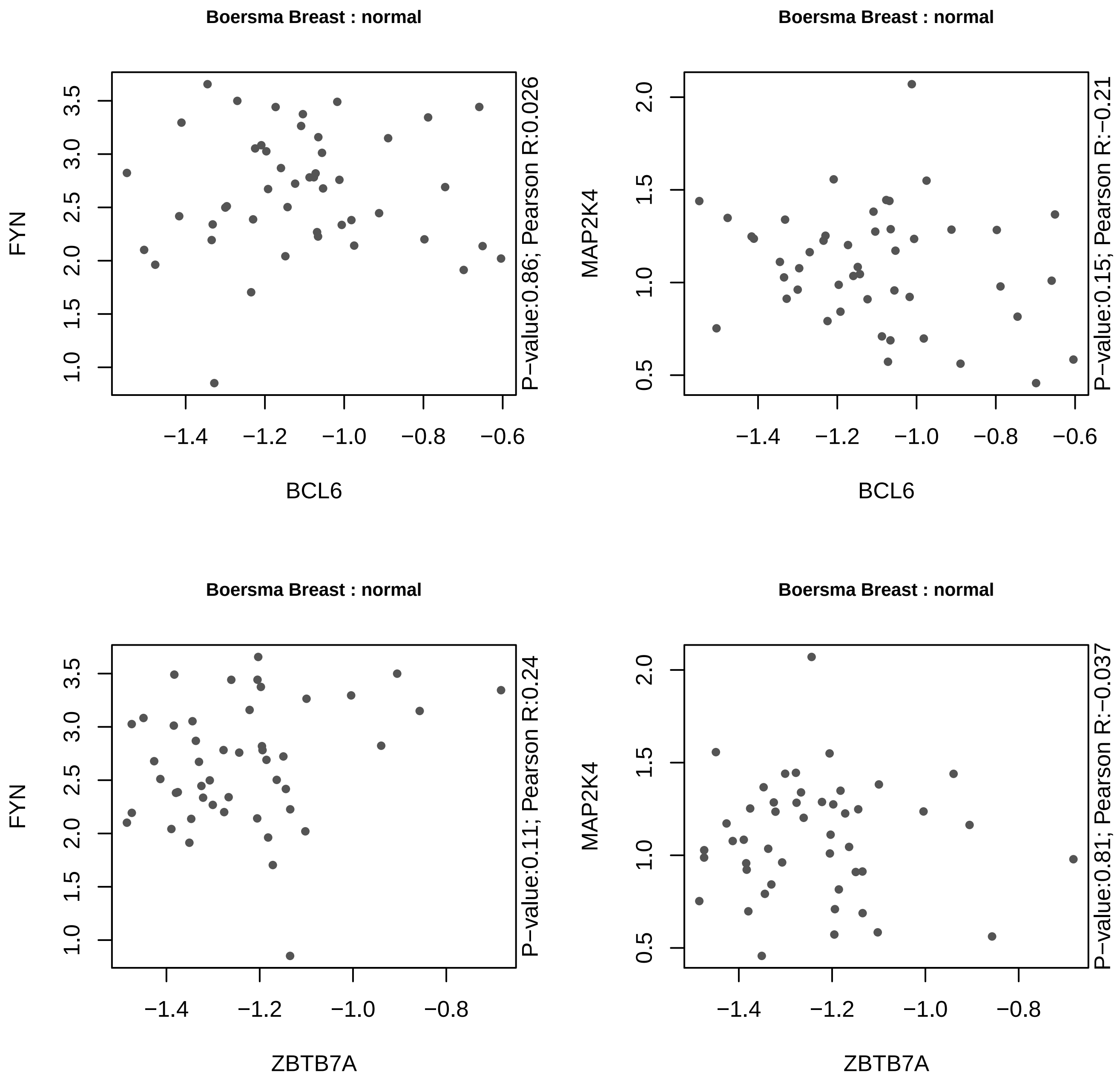
Boersma Breast : normal
FYN
BCL6
P−value:0.86; Pearson R:0.026
Boersma Breast : normal
MAP2K4
BCL6
P−value:0.15; Pearson R:−0.21
Boersma Breast : normal
FYN
ZBTB7A
P−value:0.11; Pearson R:0.24
Boersma Breast : normal
MAP2K4
ZBTB7A
P−value:0.81; Pearson R:−0.037

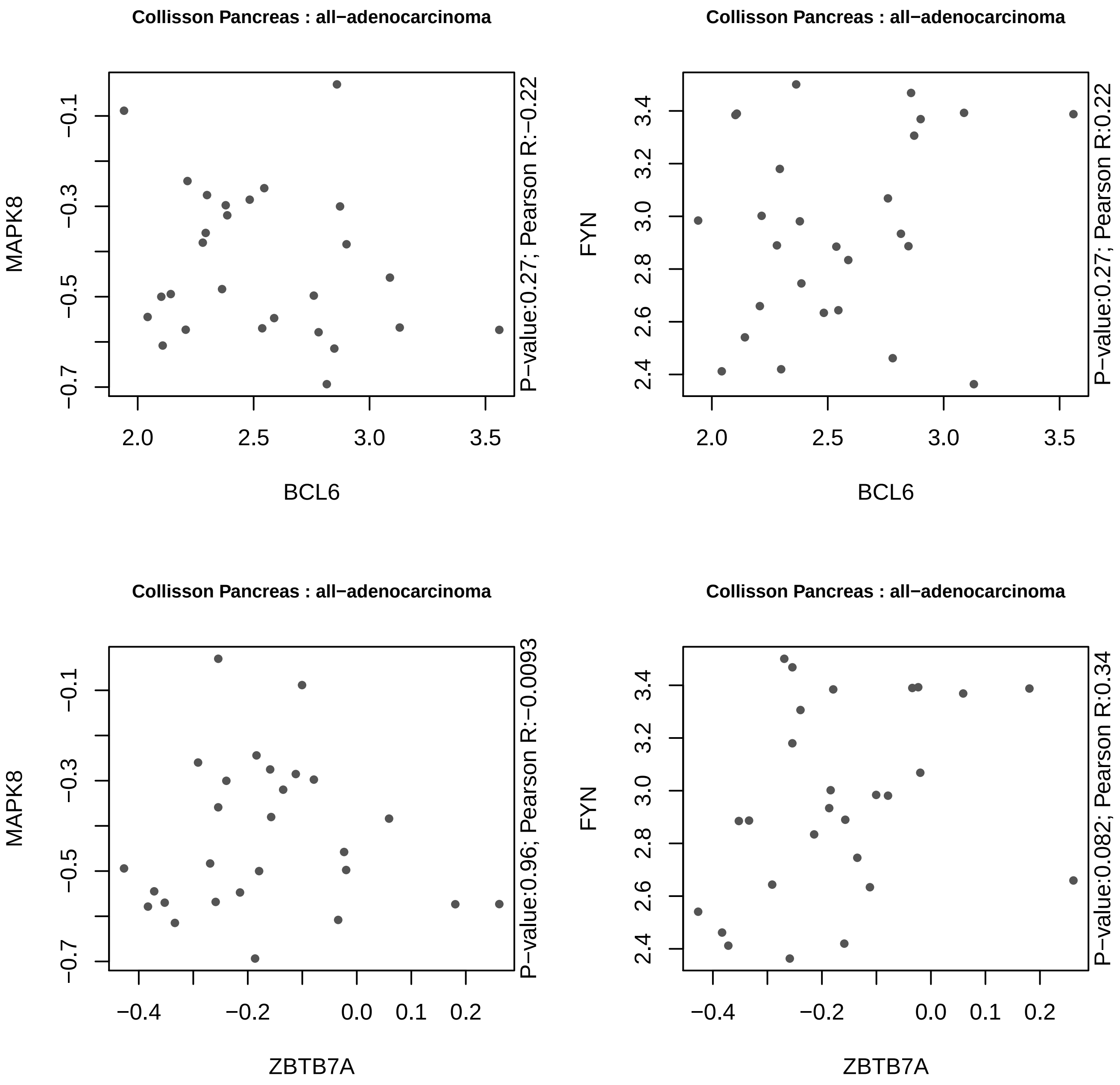
Collisson Pancreas : all−adenocarcinoma
MAPK8
BCL6
P−value:0.27; Pearson R:−0.22
Collisson Pancreas : all−adenocarcinoma
FYN
BCL6
P−value:0.27; Pearson R:0.22
Collisson Pancreas : all−adenocarcinoma
MAPK8
ZBTB7A
P−value:0.96; Pearson R:−0.0093
Collisson Pancreas : all−adenocarcinoma
FYN
ZBTB7A
P−value:0.082; Pearson R:0.34

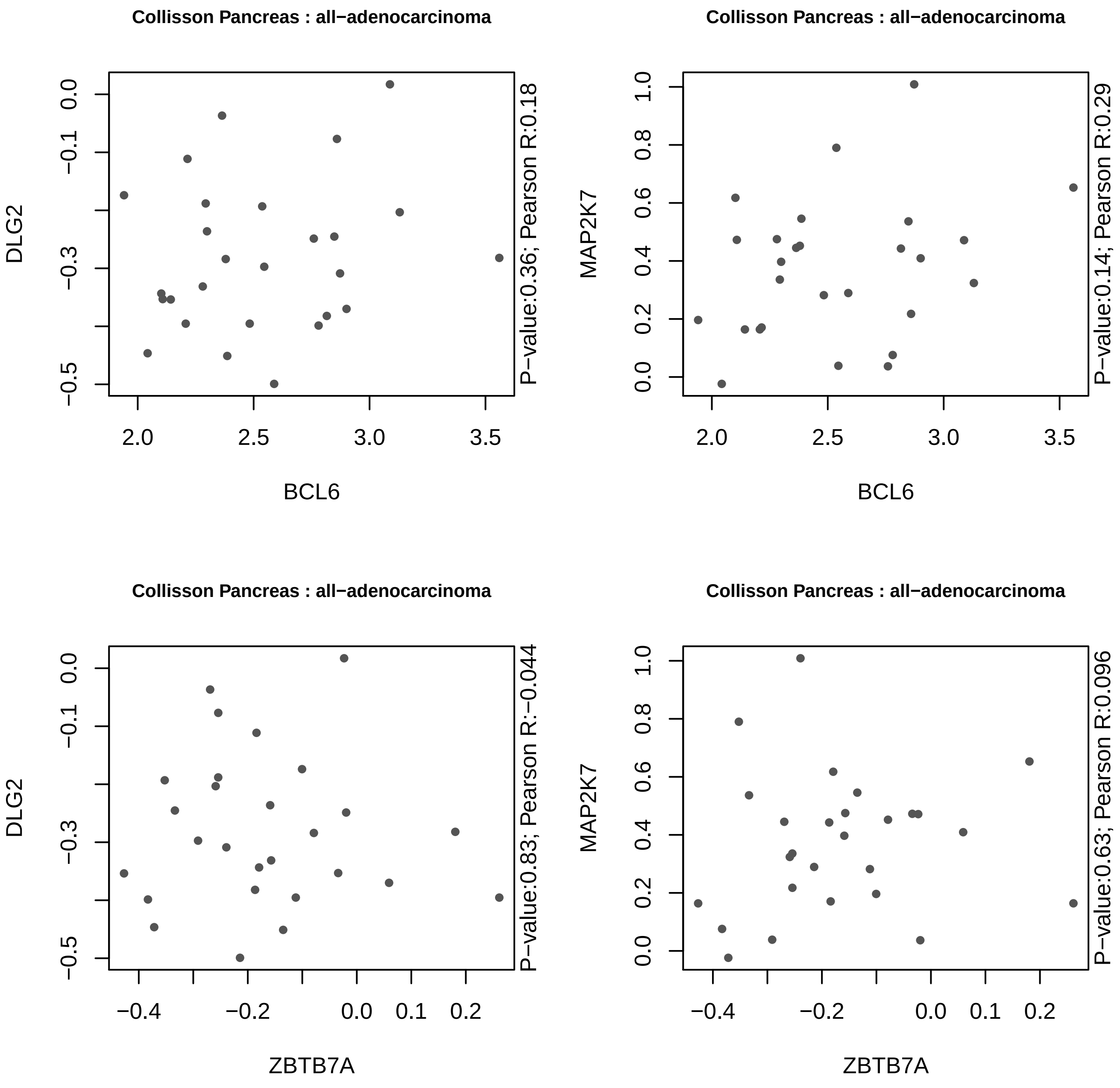
Collisson Pancreas : all−adenocarcinoma
DLG2
BCL6
P−value:0.36; Pearson R:0.18
Collisson Pancreas : all−adenocarcinoma
MAP2K7
BCL6
P−value:0.14; Pearson R:0.29
Collisson Pancreas : all−adenocarcinoma
DLG2
ZBTB7A
P−value:0.83; Pearson R:−0.044
Collisson Pancreas : all−adenocarcinoma
MAP2K7
ZBTB7A
P−value:0.63; Pearson R:0.096

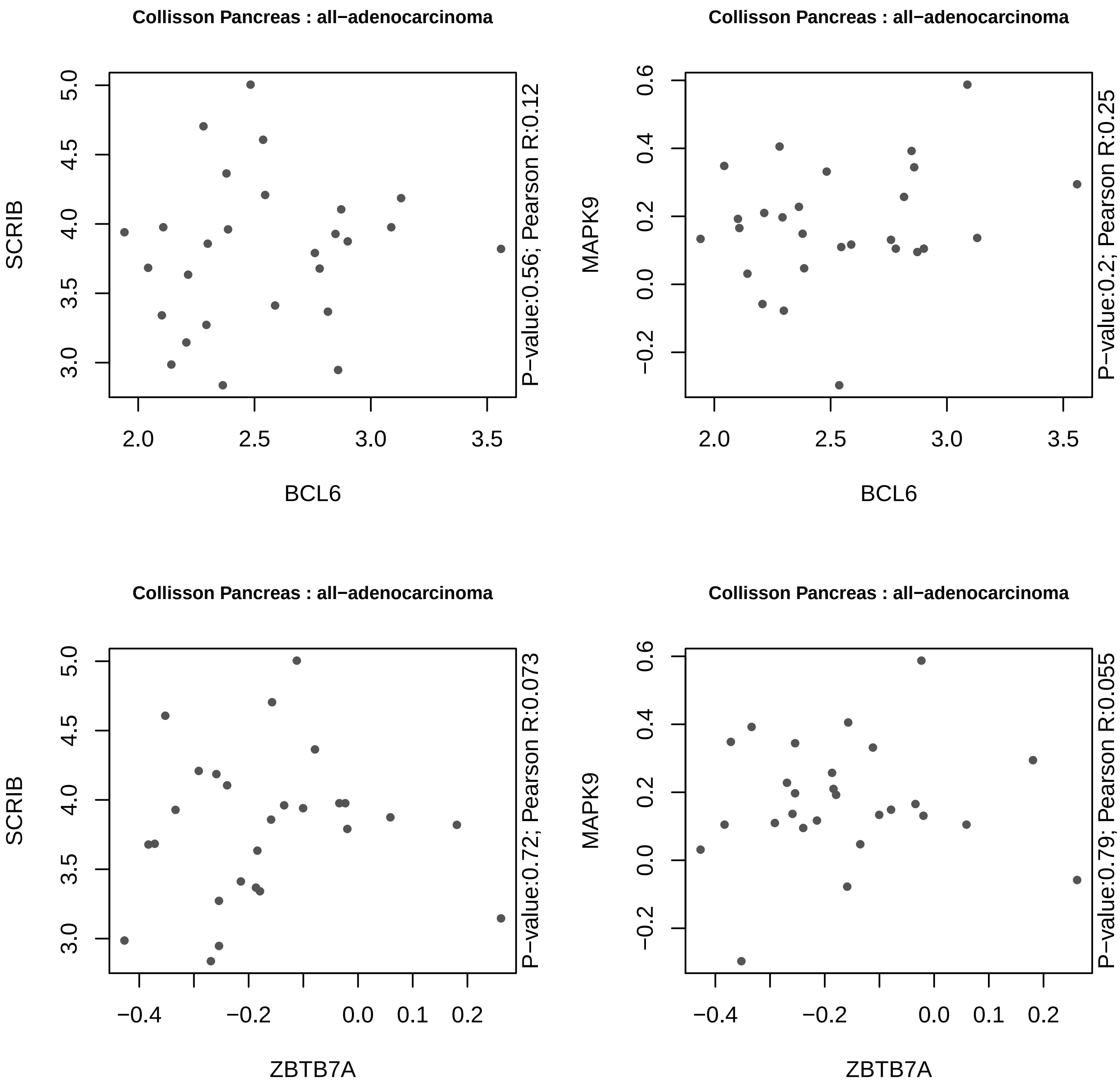
Collisson Pancreas : all−adenocarcinoma
SCRIB
BCL6
P−value:0.56; Pearson R:0.12
Collisson Pancreas : all−adenocarcinoma
MAPK9
BCL6
P−value:0.2; Pearson R:0.25
Collisson Pancreas : all−adenocarcinoma
SCRIB
ZBTB7A
P−value:0.72; Pearson R:0.073
Collisson Pancreas : all−adenocarcinoma
MAPK9
ZBTB7A
P−value:0.79; Pearson R:0.055

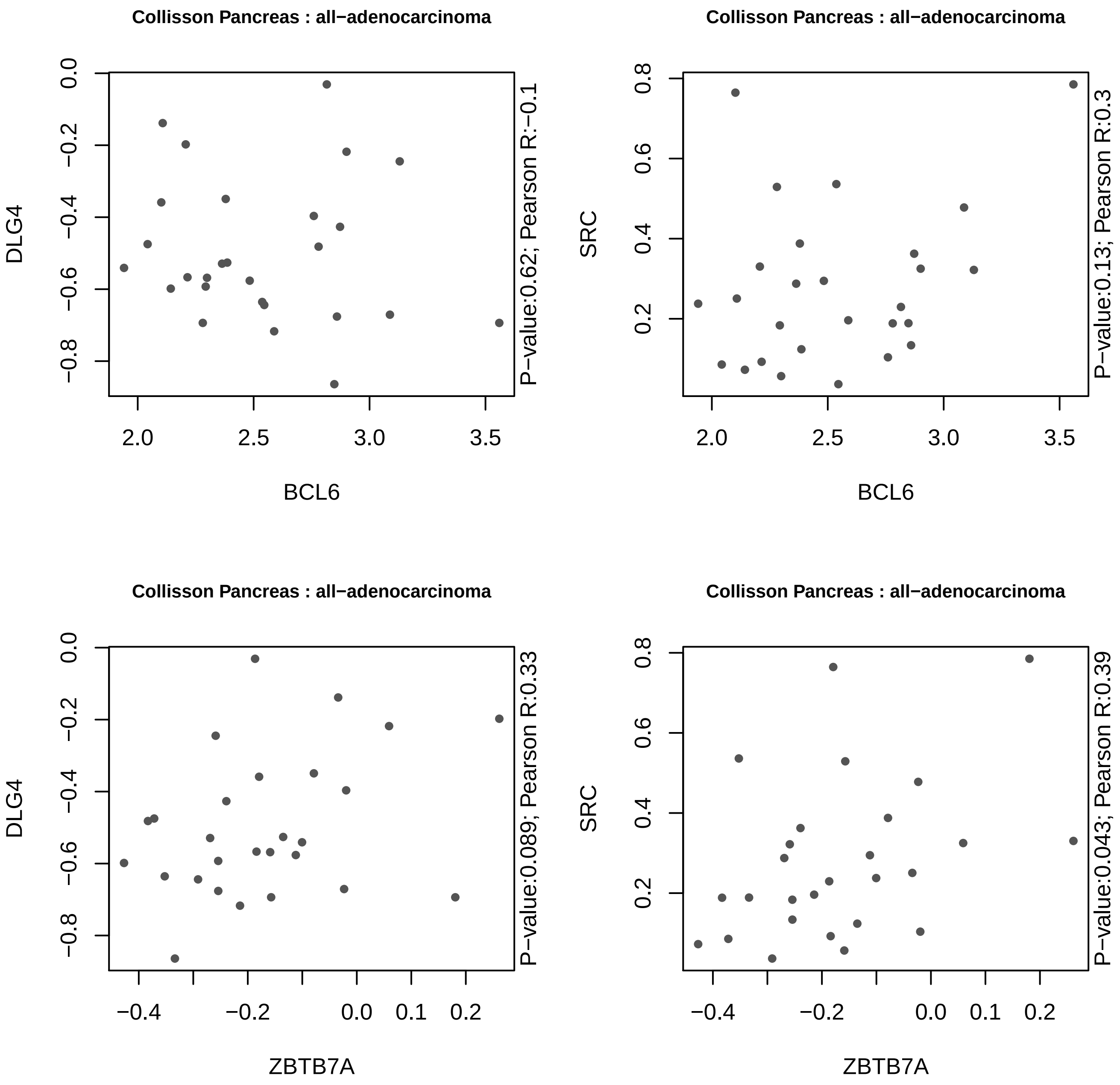
Collisson Pancreas : all−adenocarcinoma
DLG4
BCL6
P−value:0.62; Pearson R:−0.1
Collisson Pancreas : all−adenocarcinoma
SRC
BCL6
P−value:0.13; Pearson R:0.3
Collisson Pancreas : all−adenocarcinoma
DLG4
ZBTB7A
P−value:0.089; Pearson R:0.33
Collisson Pancreas : all−adenocarcinoma
SRC
ZBTB7A
P−value:0.043; Pearson R:0.39

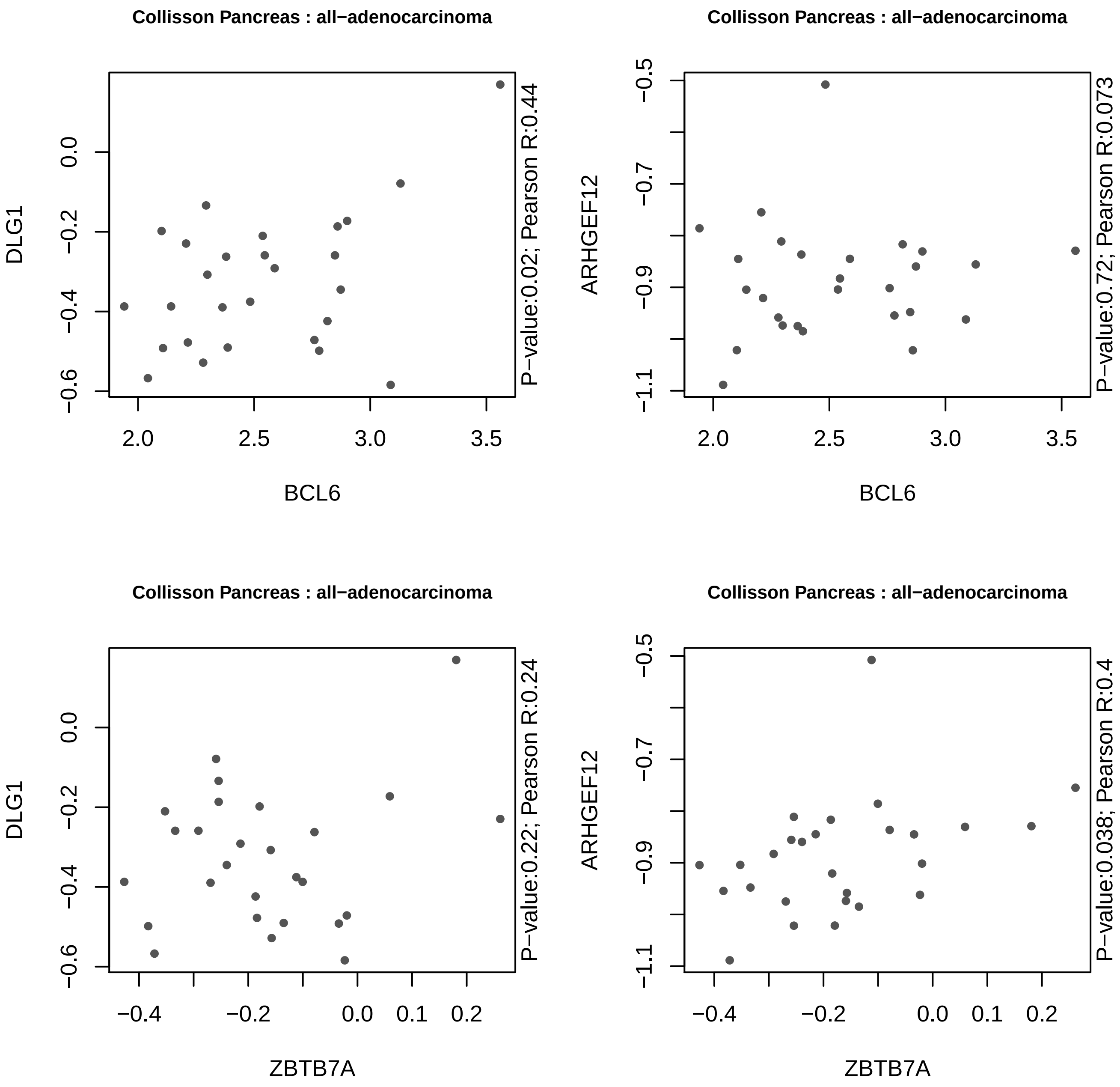
Collisson Pancreas : all−adenocarcinoma
DLG1
BCL6
P−value:0.02; Pearson R:0.44
Collisson Pancreas : all−adenocarcinoma
ARHGEF12
BCL6
P−value:0.72; Pearson R:0.073
Collisson Pancreas : all−adenocarcinoma
DLG1
ZBTB7A
P−value:0.22; Pearson R:0.24
Collisson Pancreas : all−adenocarcinoma
ARHGEF12
ZBTB7A
P−value:0.038; Pearson R:0.4

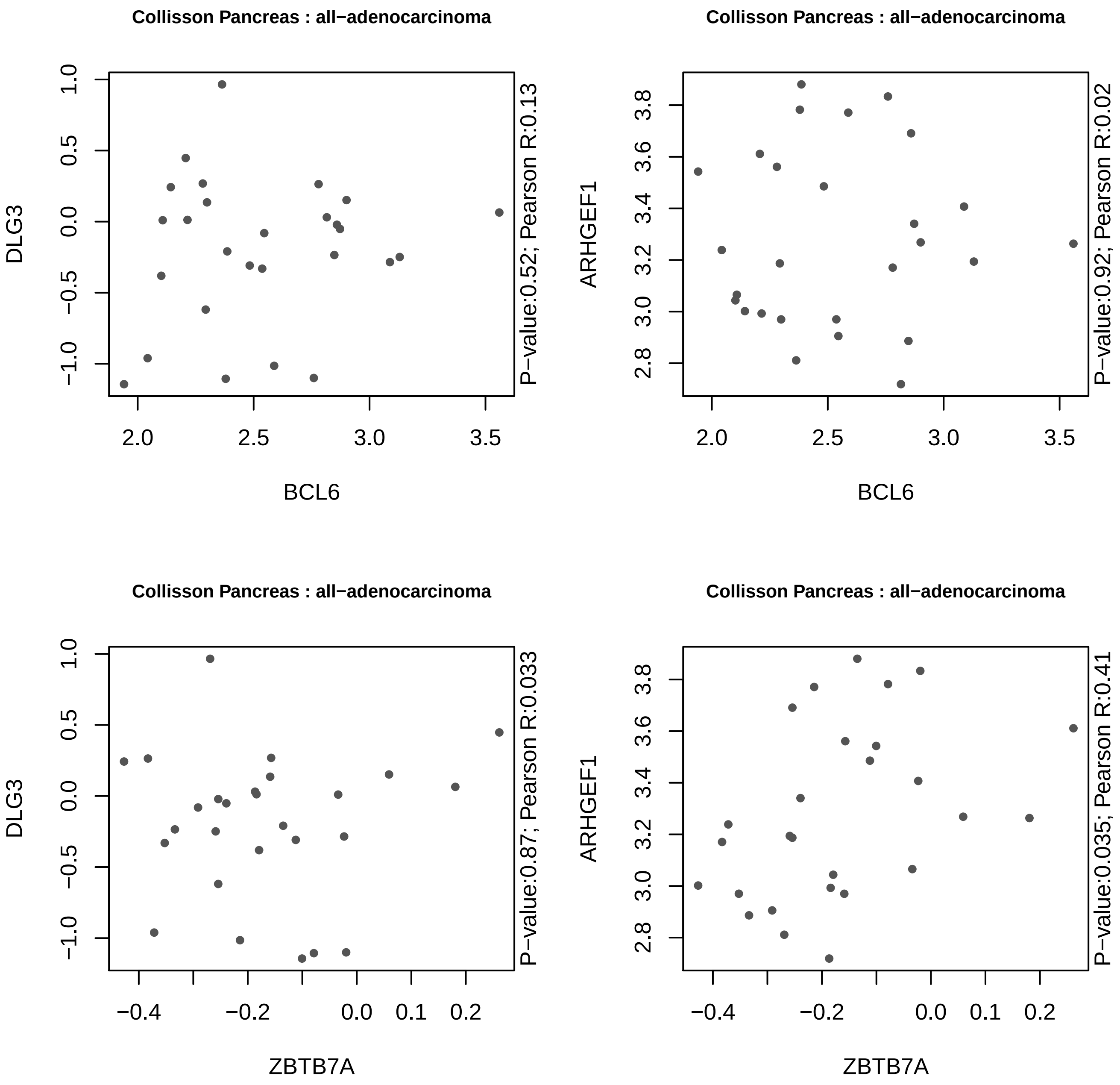
Collisson Pancreas : all−adenocarcinoma
DLG3
BCL6
P−value:0.52; Pearson R:0.13
Collisson Pancreas : all−adenocarcinoma
ARHGEF1
BCL6
P−value:0.92; Pearson R:0.02
Collisson Pancreas : all−adenocarcinoma
DLG3
ZBTB7A
P−value:0.87; Pearson R:0.033
Collisson Pancreas : all−adenocarcinoma
ARHGEF1
ZBTB7A
P−value:0.035; Pearson R:0.41

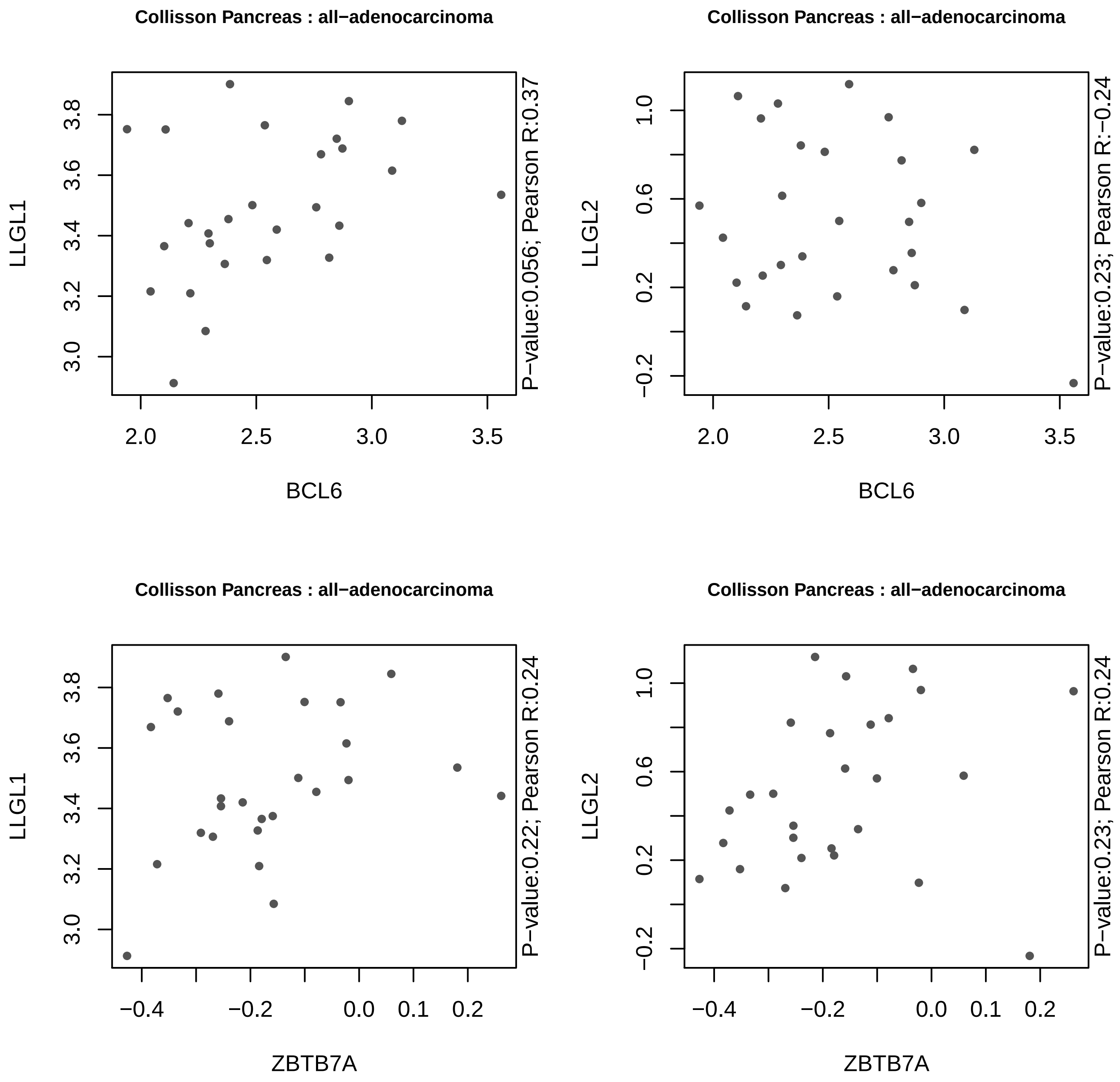

Collisson Pancreas : all−adenocarcinoma
LLGL1
BCL6
P−value:0.056; Pearson R:0.37
Collisson Pancreas : all−adenocarcinoma
LLGL2
BCL6
P−value:0.23; Pearson R:−0.24
Collisson Pancreas : all−adenocarcinoma
LLGL1
ZBTB7A
P−value:0.22; Pearson R:0.24
Collisson Pancreas : all−adenocarcinoma
LLGL2
ZBTB7A
P−value:0.23; Pearson R:0.24

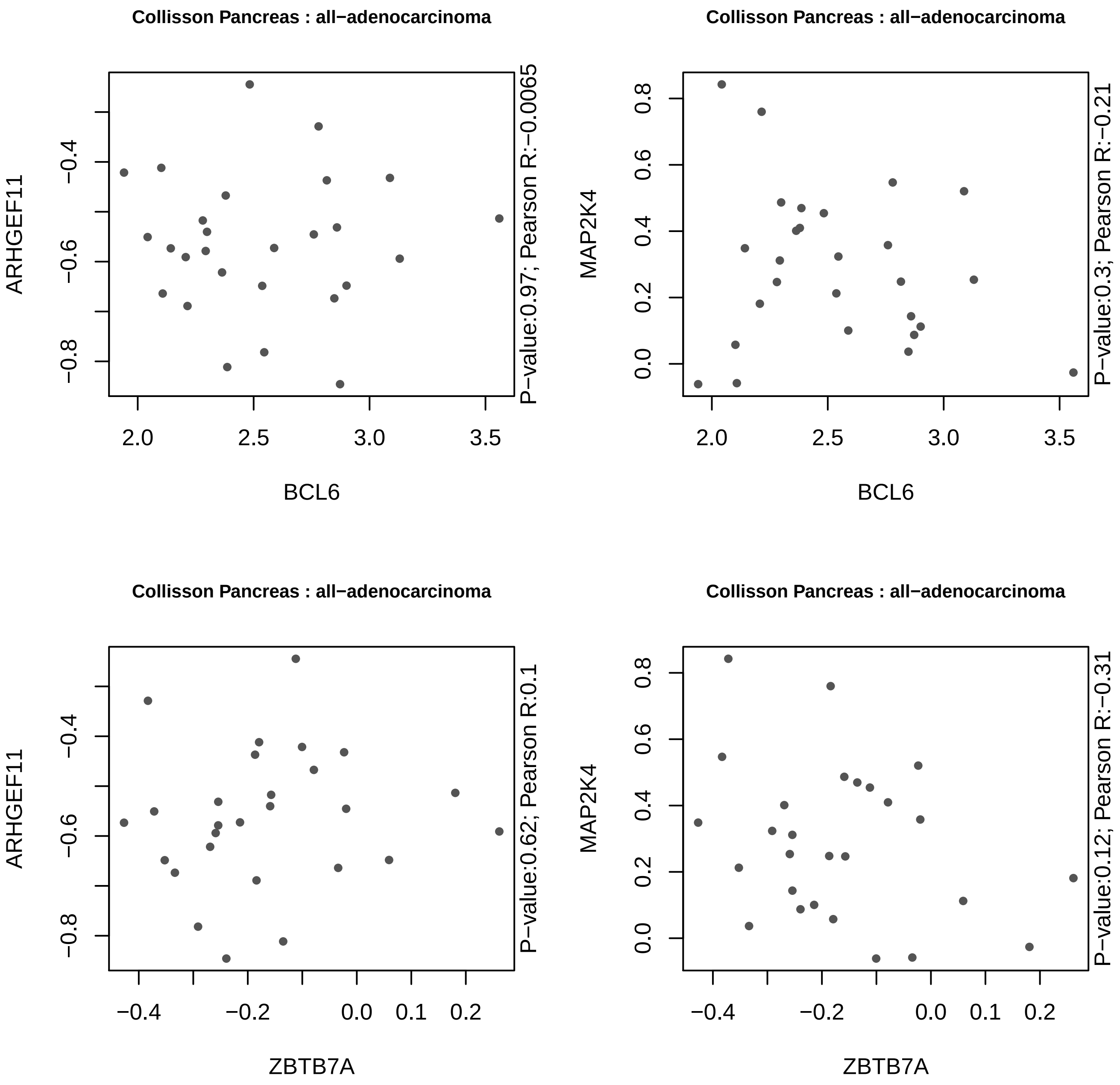
Collisson Pancreas : all−adenocarcinoma
ARHGEF11
BCL6
P−value:0.97; Pearson R:−0.0065
Collisson Pancreas : all−adenocarcinoma
MAP2K4
BCL6
P−value:0.3; Pearson R:−0.21
Collisson Pancreas : all−adenocarcinoma
ARHGEF11
ZBTB7A
P−value:0.62; Pearson R:0.1
Collisson Pancreas : all−adenocarcinoma
MAP2K4
ZBTB7A
P−value:0.12; Pearson R:−0.31

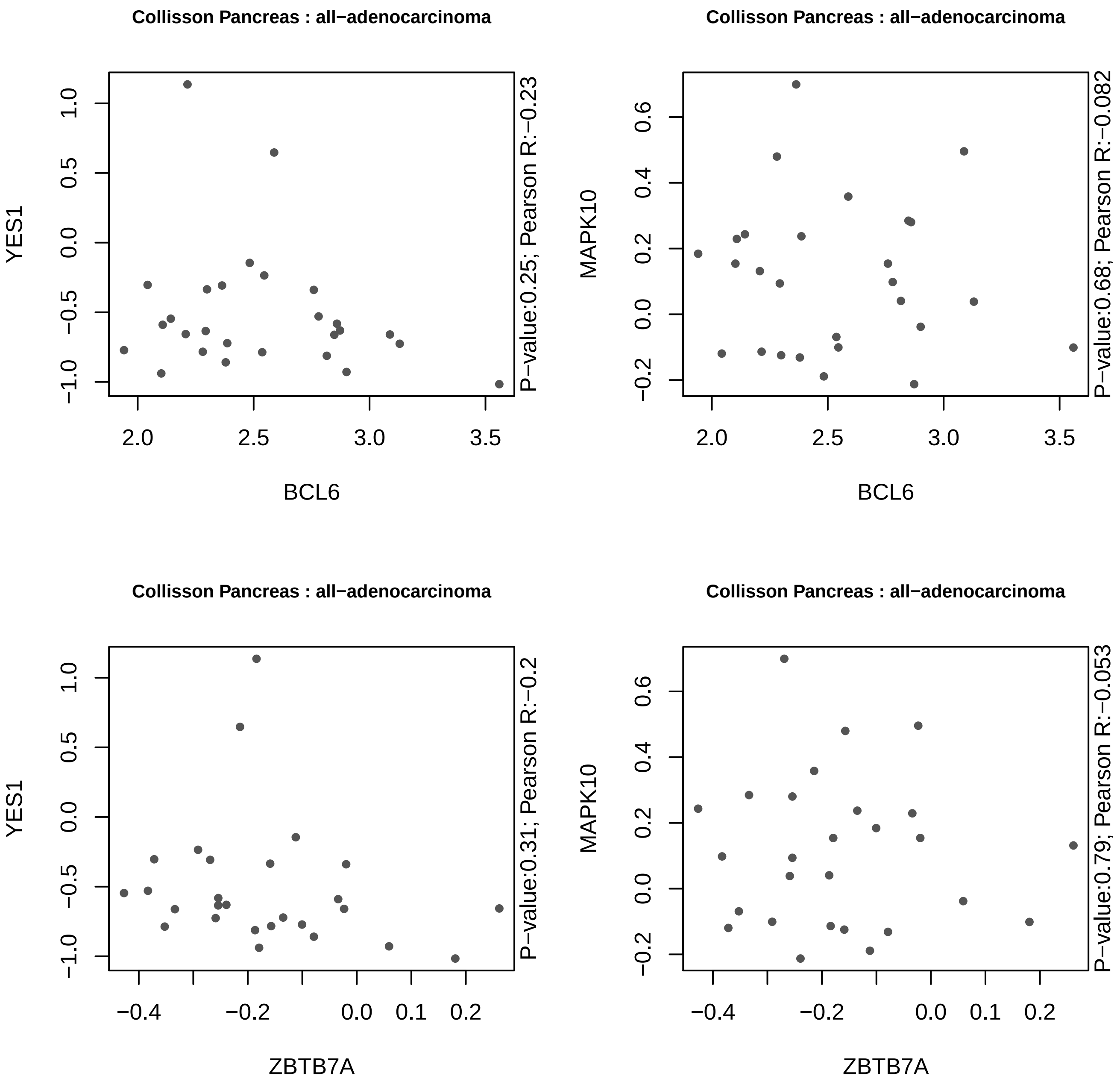
Collisson Pancreas : all−adenocarcinoma
YES1
BCL6
P−value:0.25; Pearson R:−0.23
Collisson Pancreas : all−adenocarcinoma
MAPK10
BCL6
P−value:0.68; Pearson R:−0.082
Collisson Pancreas : all−adenocarcinoma
YES1
ZBTB7A
P−value:0.31; Pearson R:−0.2
Collisson Pancreas : all−adenocarcinoma
MAPK10
ZBTB7A
P−value:0.79; Pearson R:−0.053

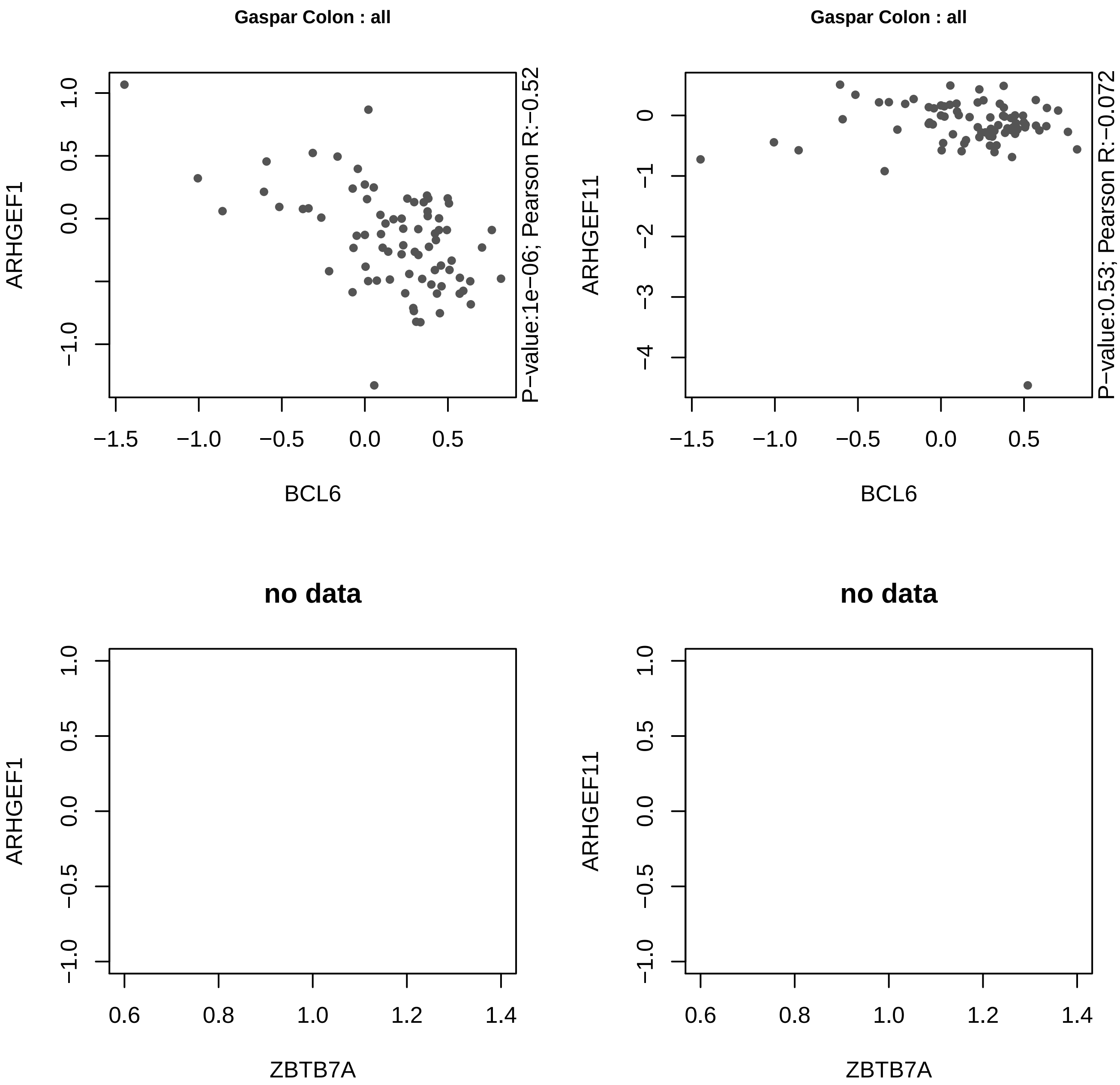
Gaspar Colon : all
ARHGEF1
BCL6
P−value:1e−06; Pearson R:−0.52
Gaspar Colon : all
ARHGEF11
BCL6
P−value:0.53; Pearson R:−0.072
no data
ARHGEF1
ZBTB7A
no data
ARHGEF11
ZBTB7A

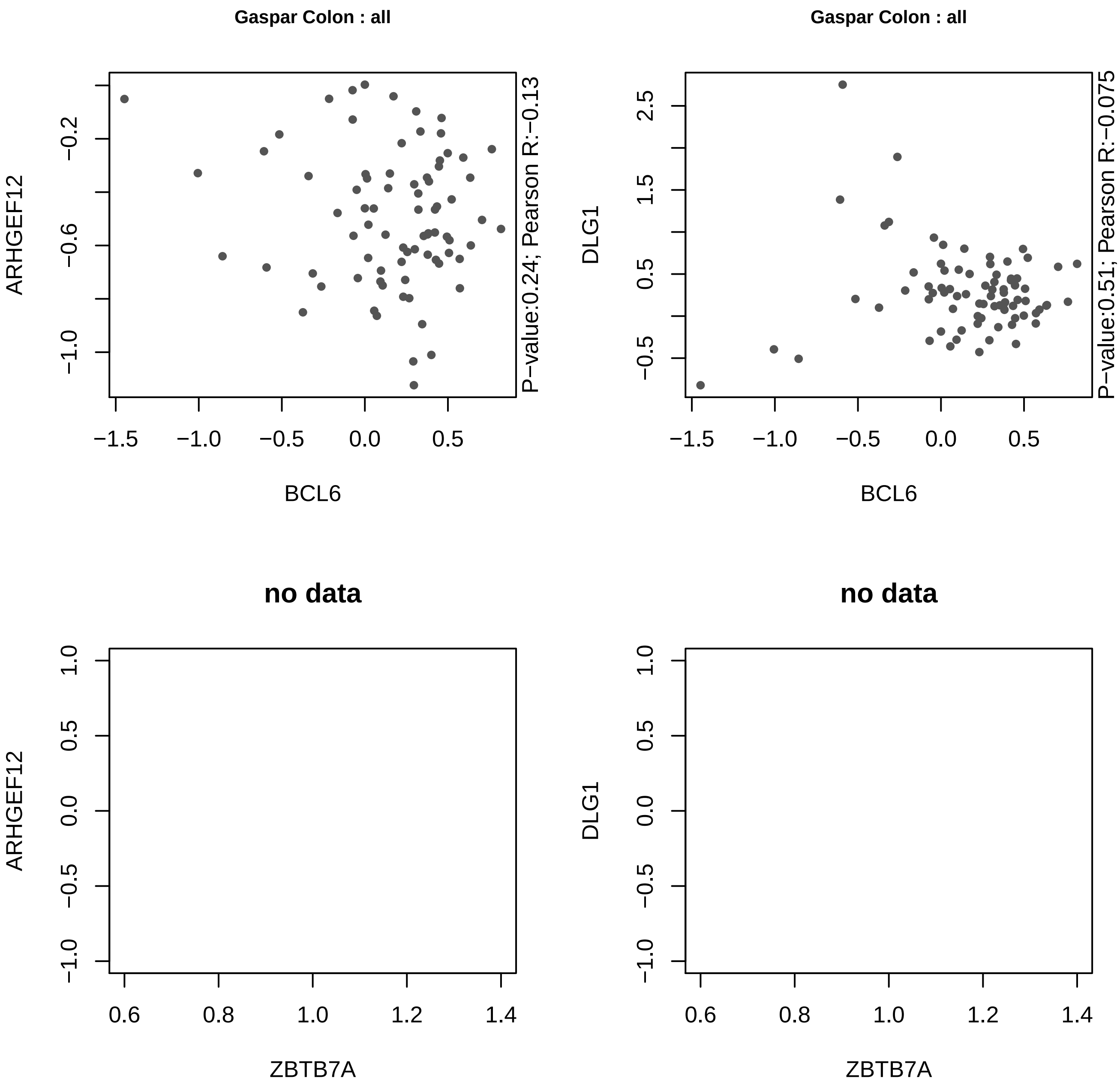
Gaspar Colon : all
ARHGEF12
−0.2
−0.6
−1.0
P−value:0.24; Pearson R:−0.13
−1.5
−1.0
−0.5
0.0
0.5
BCL6
Gaspar Colon : all
DLG1
2.5
1.5
0.5
−0.5
P−value:0.51; Pearson R:−0.075
BCL6
no data
ARHGEF12
1.0
0.5
0.0
−0.5
−1.0
0.6
0.8
1.0
1.2
1.4
ZBTB7A
no data
DLG1
ZBTB7A

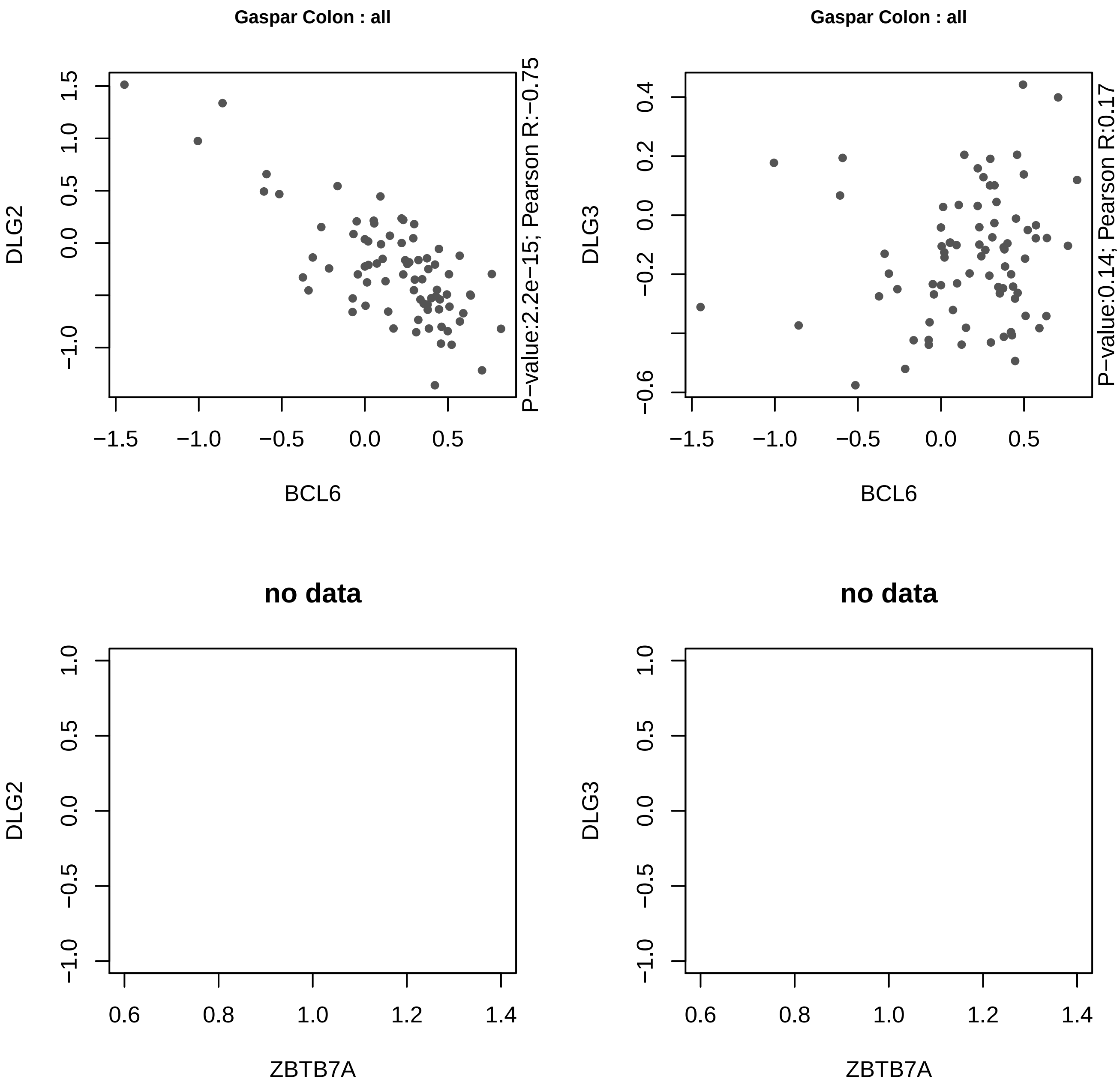
Gaspar Colon : all
DLG2
BCL6
P−value:2.2e−15; Pearson R:−0.75
Gaspar Colon : all
DLG3
BCL6
P−value:0.14; Pearson R:0.17
no data
DLG2
ZBTB7A
no data
DLG3
ZBTB7A

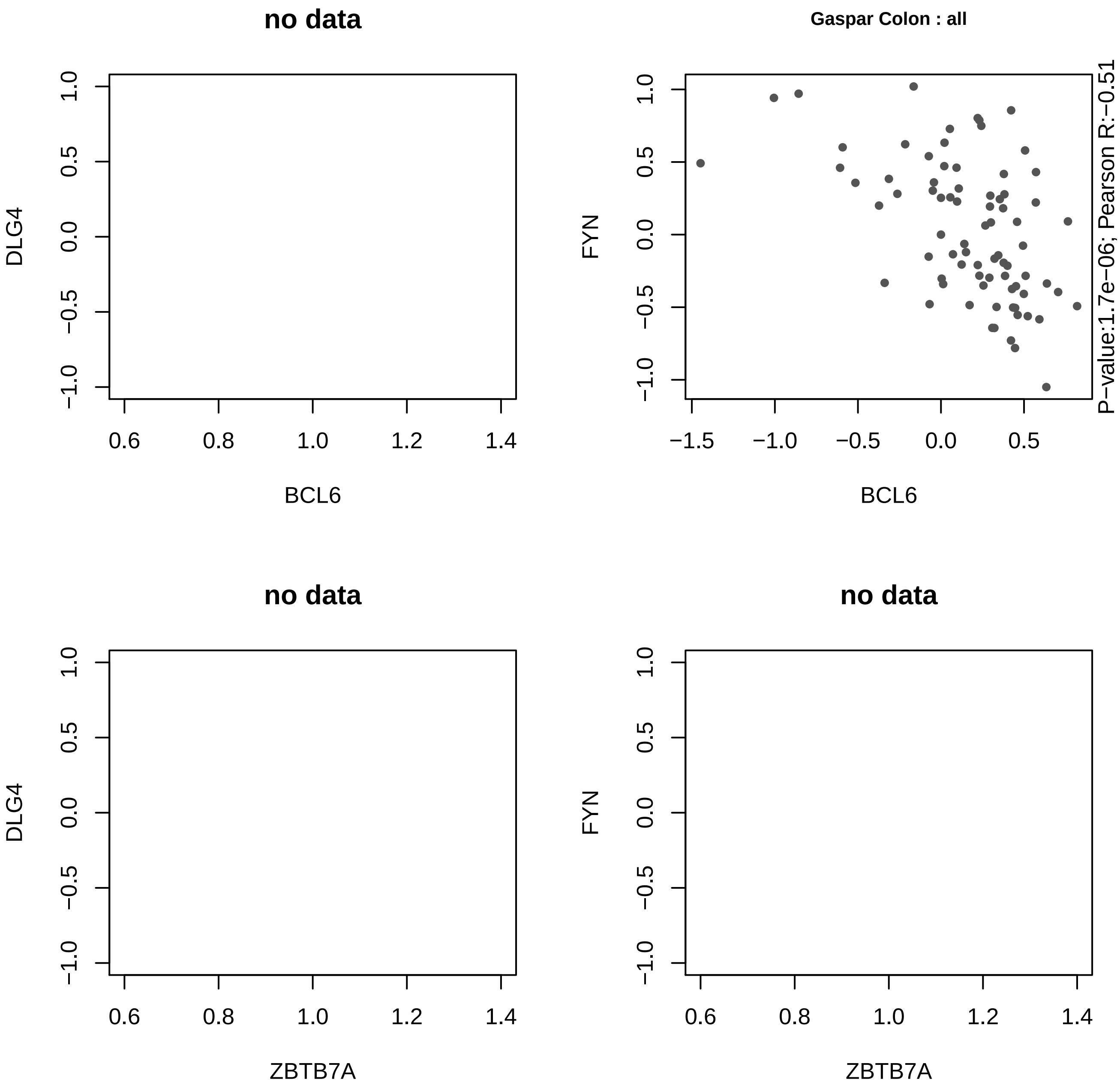
no data
DLG4
1.0
0.5
0.0
−0.5
−1.0
0.6
0.8
1.0
1.2
1.4
BCL6
Gaspar Colon : all
FYN
−1.5
−1.0
−0.5
0.0
0.5
P−value:1.7e−06; Pearson R:−0.51
no data
ZBTB7A
no data
ZBTB7A

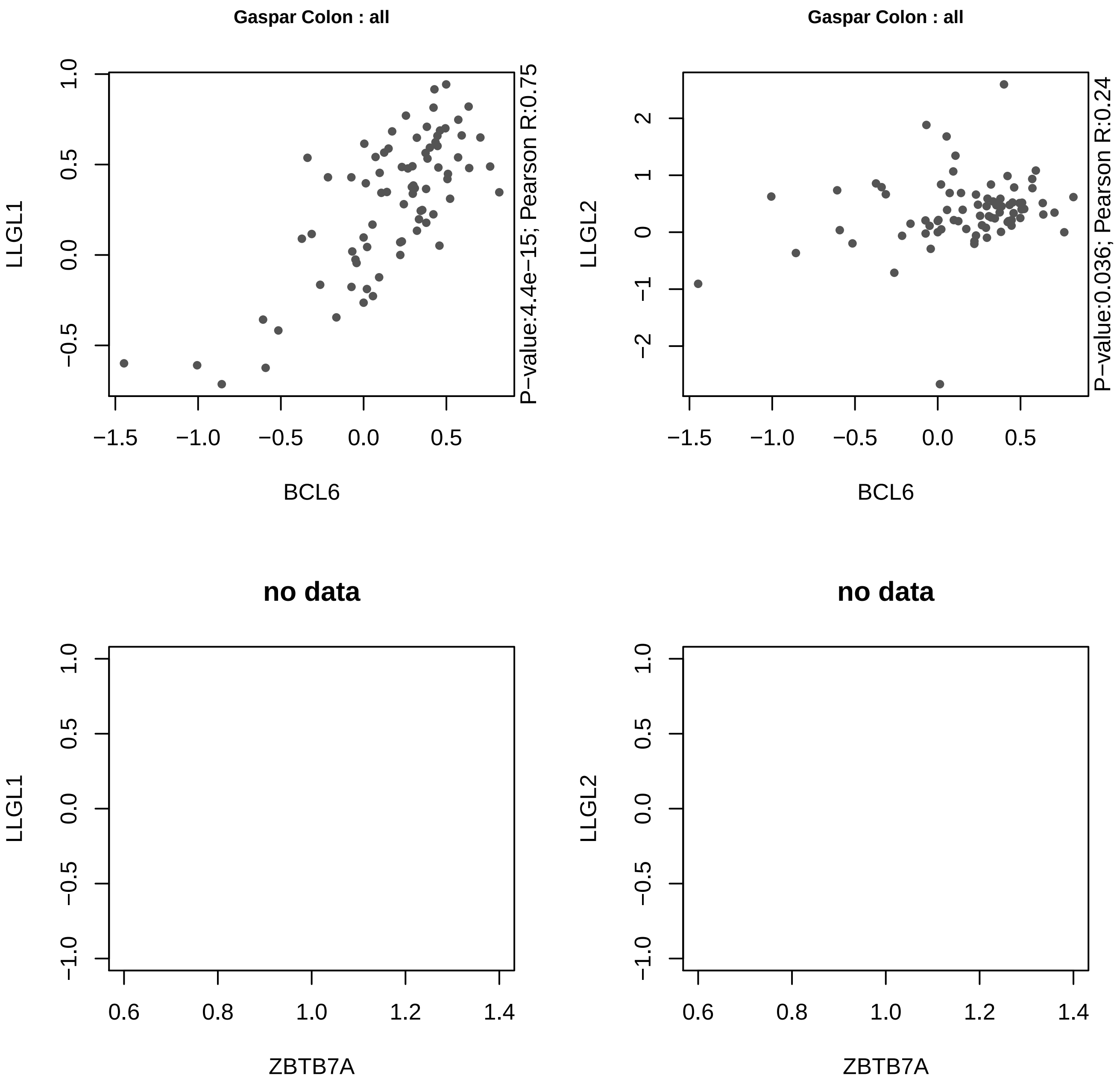
Gaspar Colon : all
LLGL1
BCL6
P−value:4.4e−15; Pearson R:0.75
Gaspar Colon : all
LLGL2
BCL6
P−value:0.036; Pearson R:0.24
no data
LLGL1
ZBTB7A
no data
LLGL2
ZBTB7A

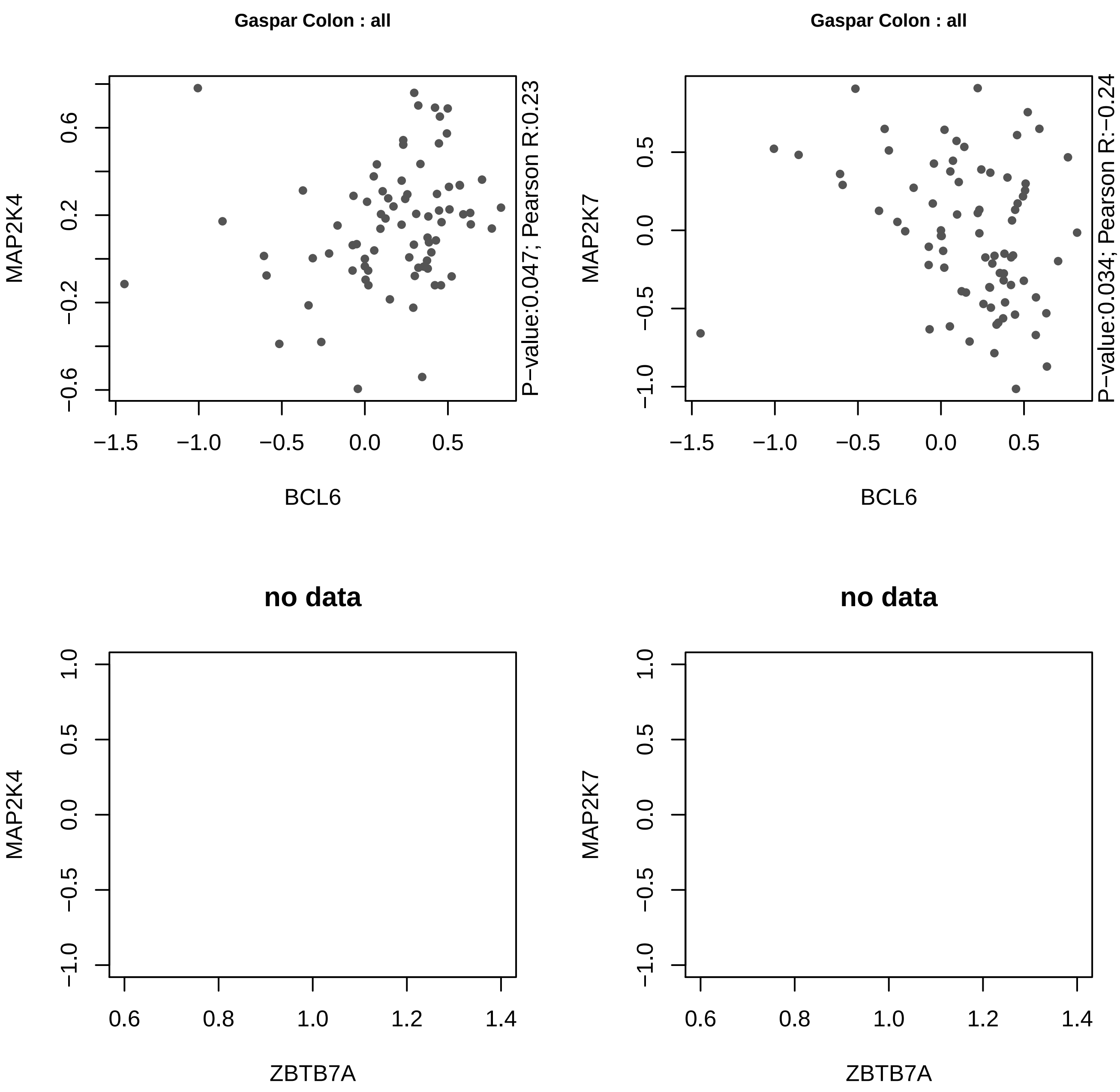
Gaspar Colon : all
MAP2K4
BCL6
P−value:0.047; Pearson R:0.23
Gaspar Colon : all
MAP2K7
BCL6
P−value:0.034; Pearson R:−0.24
no data
MAP2K4
ZBTB7A
no data
MAP2K7
ZBTB7A

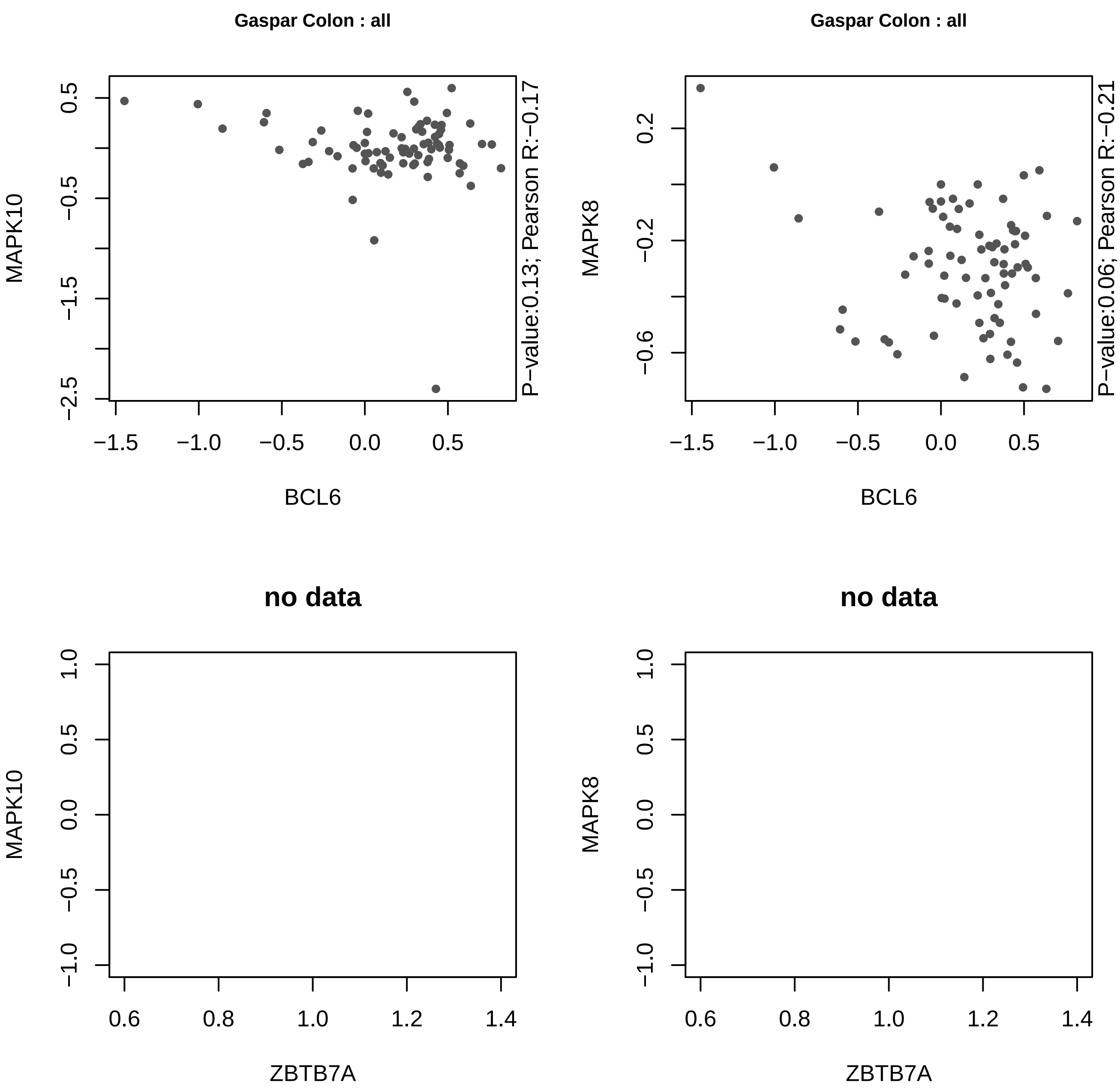
Gaspar Colon : all
MAPK10
BCL6
P−value:0.13; Pearson R:−0.17
Gaspar Colon : all
MAPK8
BCL6
P−value:0.06; Pearson R:−0.21
no data
MAPK10
ZBTB7A
no data
MAPK8
ZBTB7A

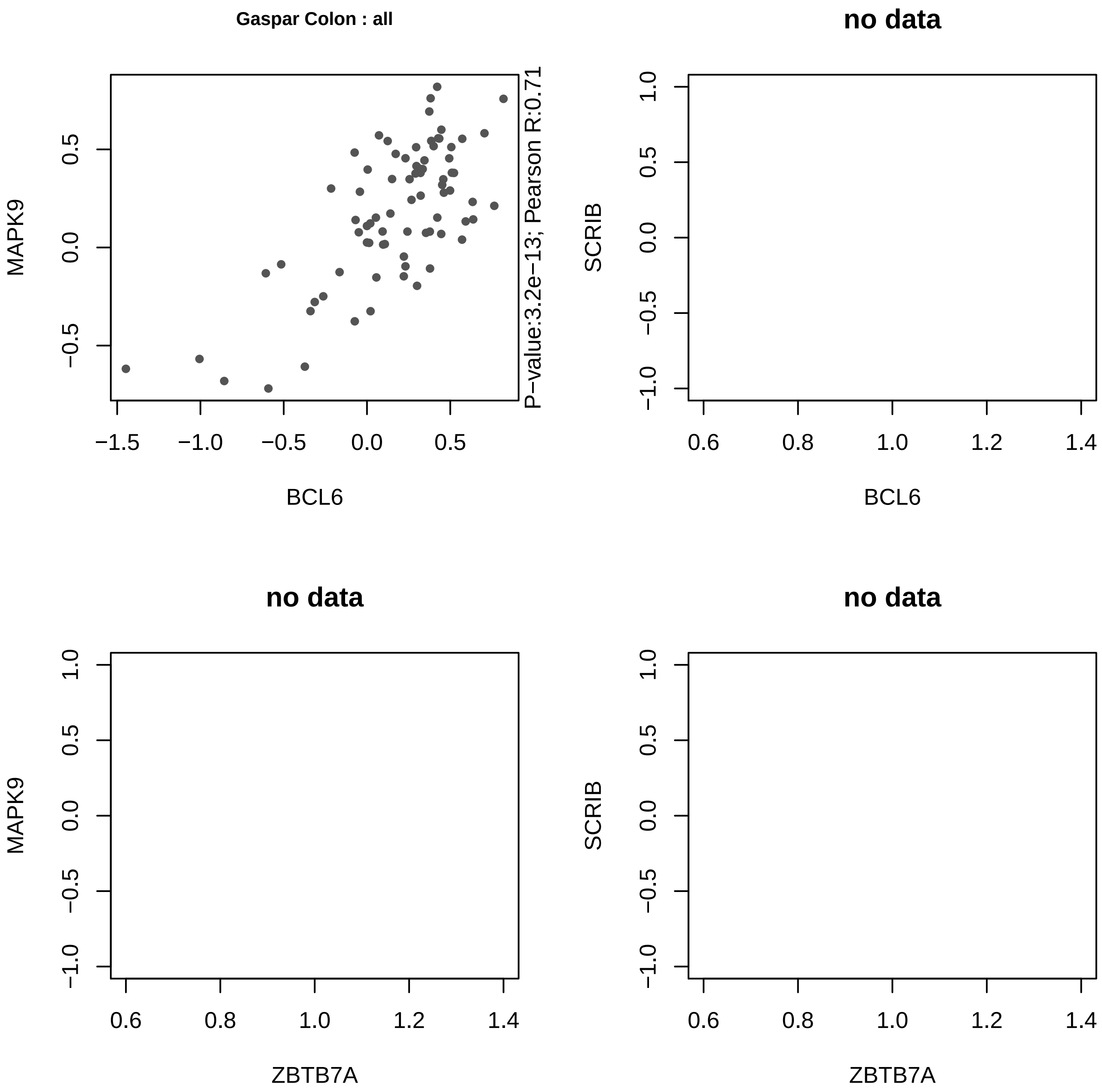
Gaspar Colon : all
MAPK9
BCL6
P−value:3.2e−13; Pearson R:0.71
−1.5
−1.0
−0.5
0.0
0.5
no data
SCRIB
BCL6
no data
MAPK9
ZBTB7A
no data
SCRIB
ZBTB7A
0.6
0.8
1.0
1.2
1.4

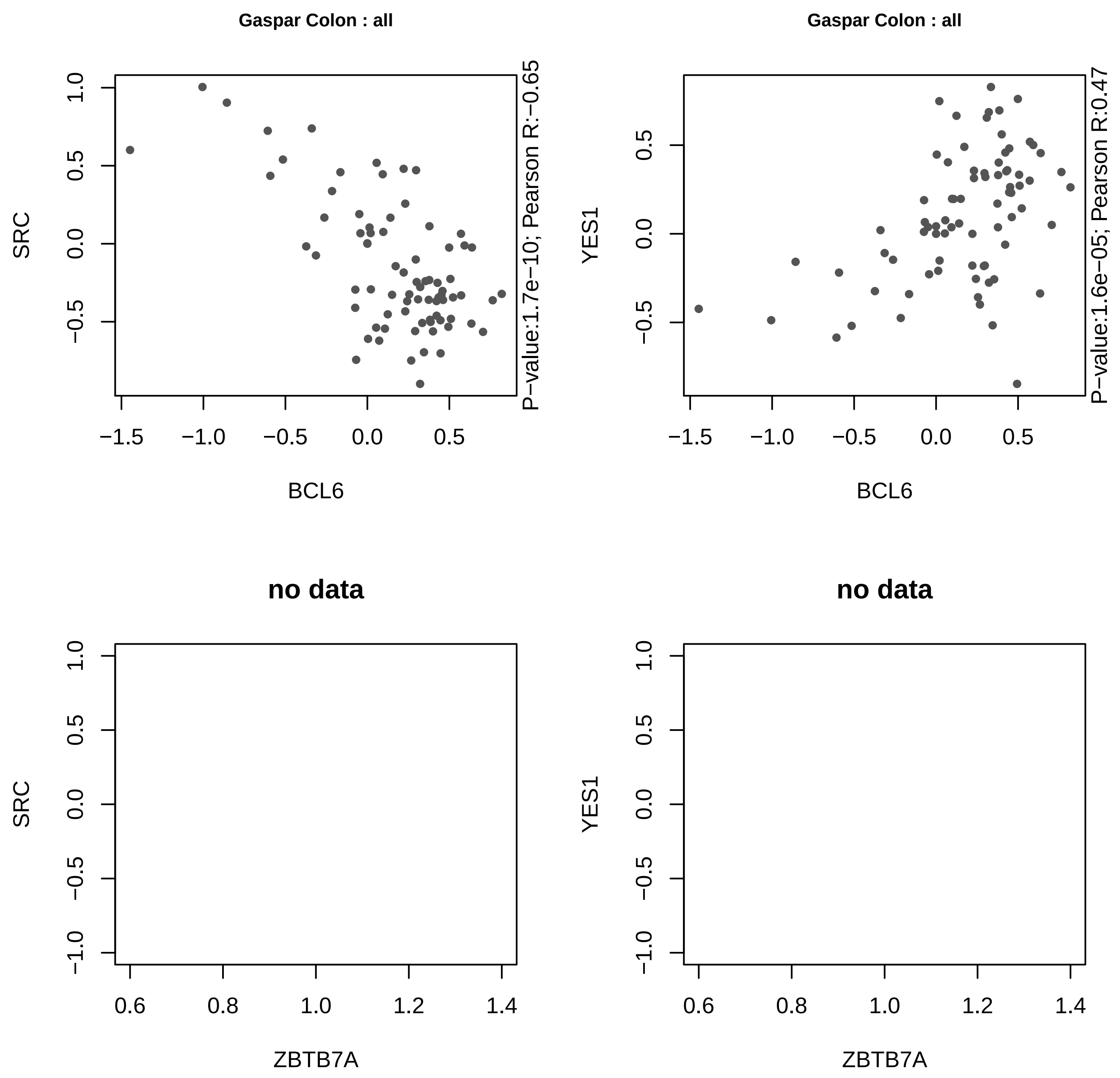
Gaspar Colon : all
SRC
BCL6
P−value:1.7e−10; Pearson R:−0.65
Gaspar Colon : all
YES1
BCL6
P−value:1.6e−05; Pearson R:0.47
no data
SRC
ZBTB7A
no data
YES1
ZBTB7A

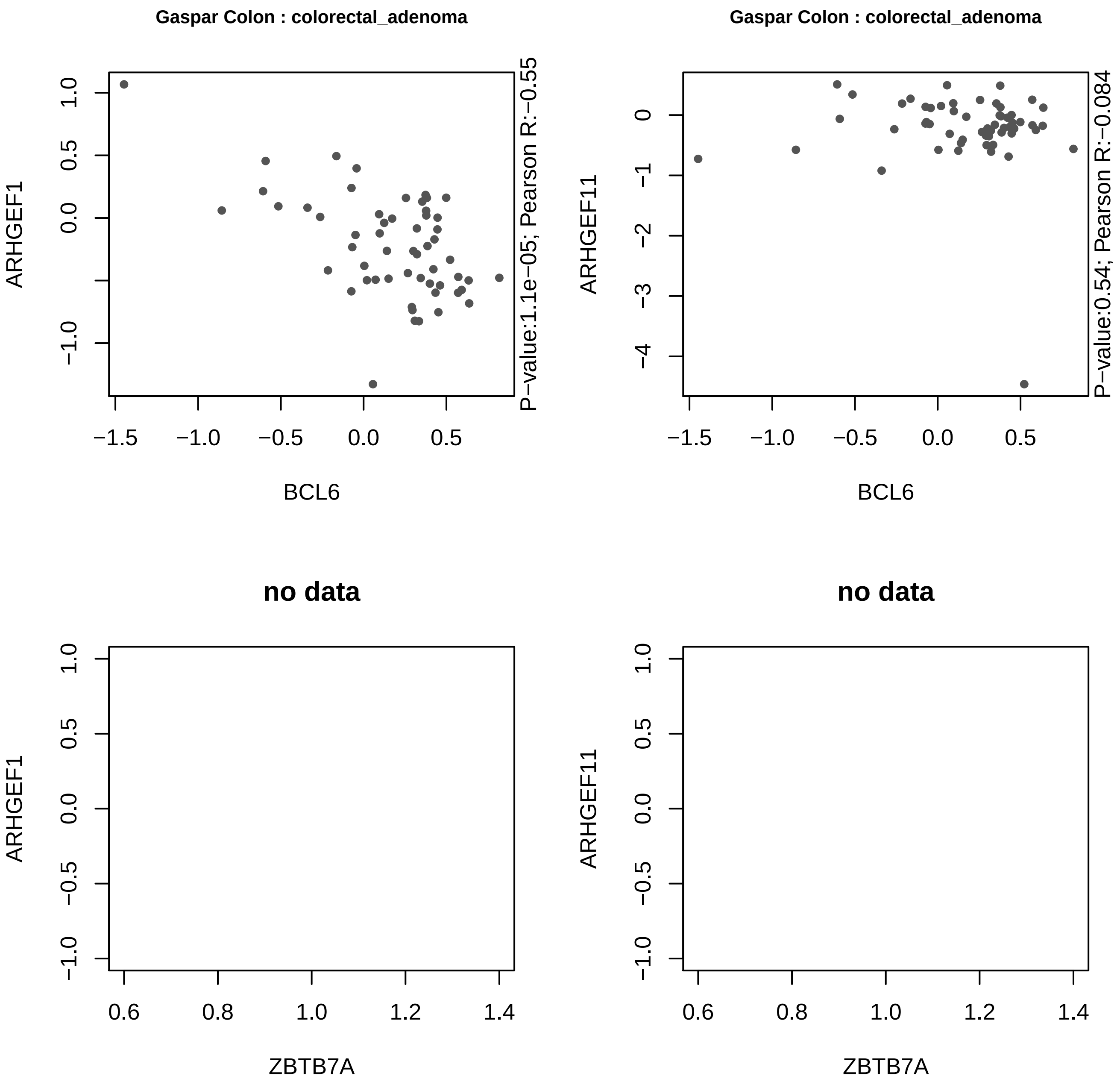
Gaspar Colon : colorectal_adenoma
ARHGEF1
BCL6
P−value:1.1e−05; Pearson R:−0.55
Gaspar Colon : colorectal_adenoma
ARHGEF11
BCL6
P−value:0.54; Pearson R:−0.084
no data
ARHGEF1
ZBTB7A
no data
ARHGEF11
ZBTB7A

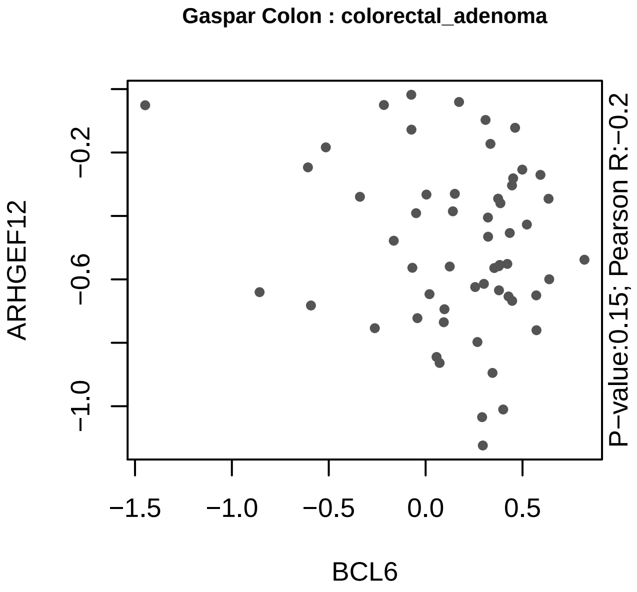
Gaspar Colon : colorectal_adenoma
ARHGEF12
BCL6
P−value:0.15; Pearson R:−0.2
−0.2
−0.6
−1.0
−1.5
−1.0
−0.5
0.0
0.5

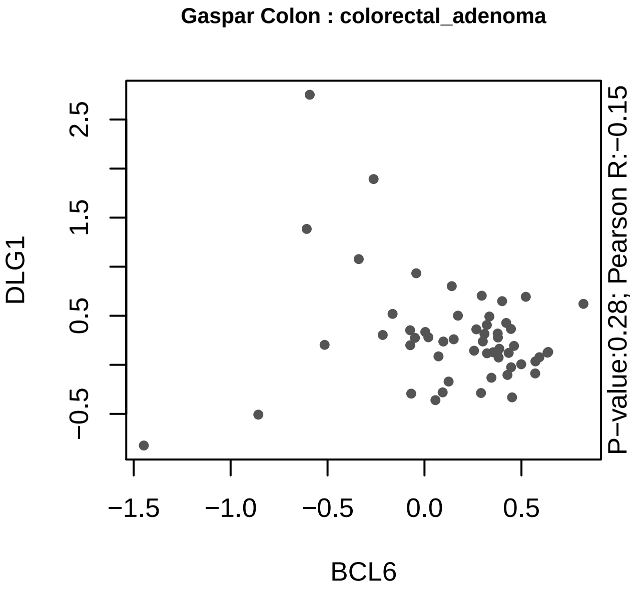
Gaspar Colon : colorectal_adenoma
DLG1
BCL6
P−value:0.28; Pearson R:−0.15
2.5
1.5
0.5
−0.5
−1.5
−1.0
−0.5
0.0
0.5

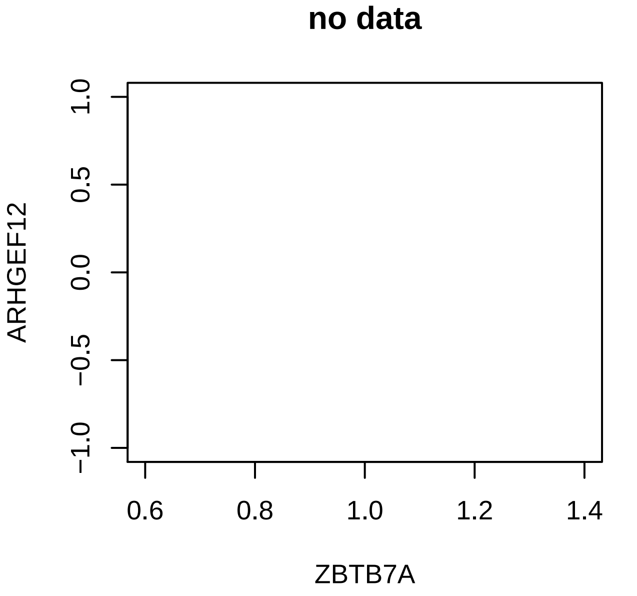
no data
ARHGEF12
ZBTB7A
1.0
0.5
0.0
−0.5
−1.0
0.6
0.8
1.0
1.2
1.4

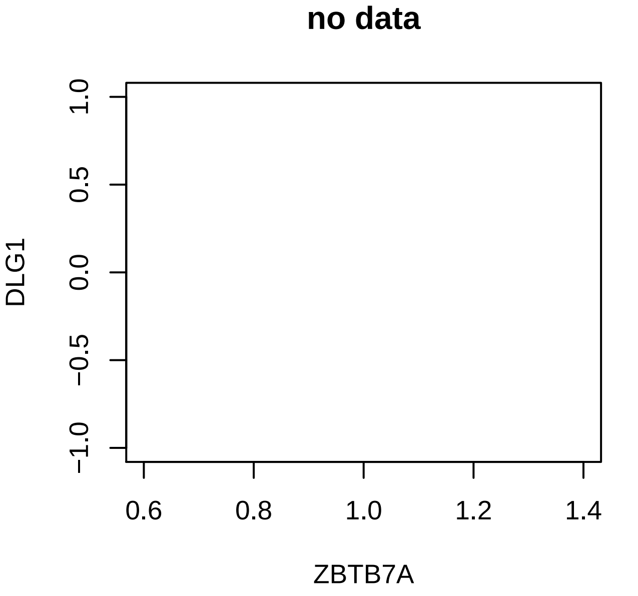
no data
DLG1
ZBTB7A
1.0
0.5
0.0
−0.5
−1.0
0.6
0.8
1.0
1.2
1.4

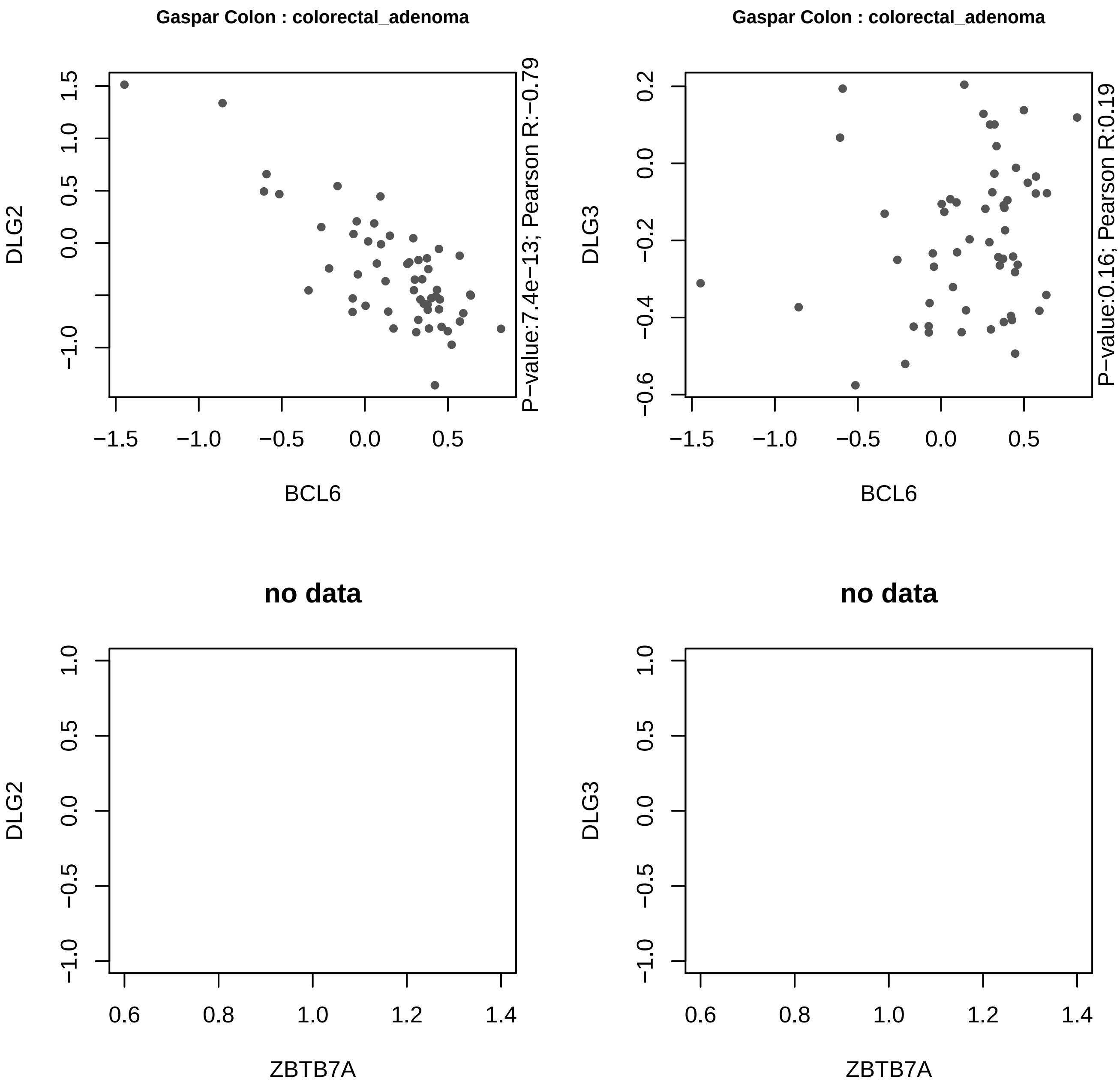
Gaspar Colon : colorectal_adenoma
DLG2
BCL6
P−value:7.4e−13; Pearson R:−0.79
Gaspar Colon : colorectal_adenoma
DLG3
BCL6
P−value:0.16; Pearson R:0.19
no data
DLG2
ZBTB7A
no data
DLG3
ZBTB7A

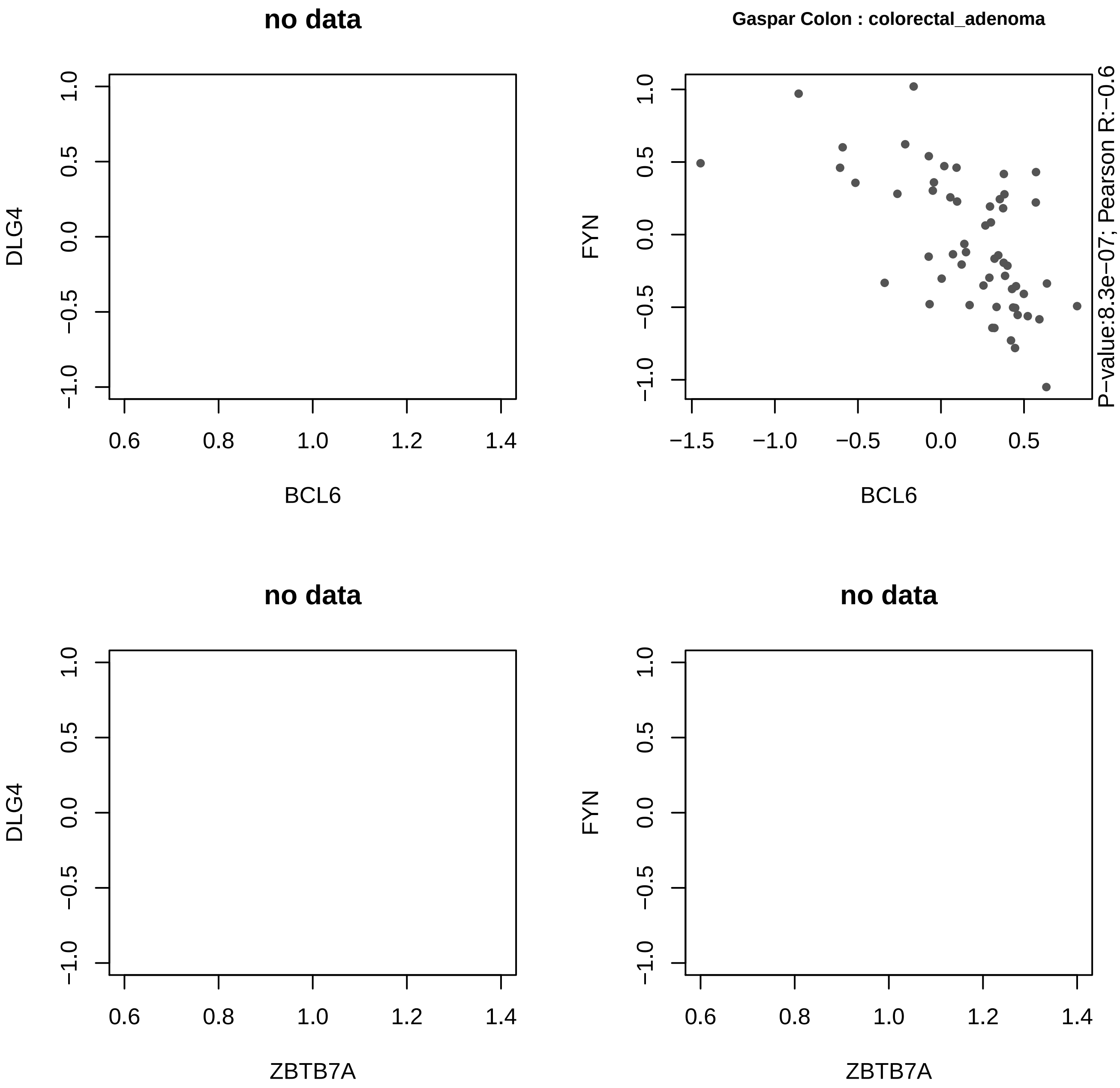
no data
DLG4
BCL6
1.0
0.5
0.0
−0.5
−1.0
0.6
0.8
1.0
1.2
1.4
Gaspar Colon : colorectal_adenoma
FYN
BCL6
P−value:8.3e−07; Pearson R:−0.6
−1.5
−1.0
−0.5
0.0
0.5
no data
DLG4
ZBTB7A
no data
FYN
ZBTB7A

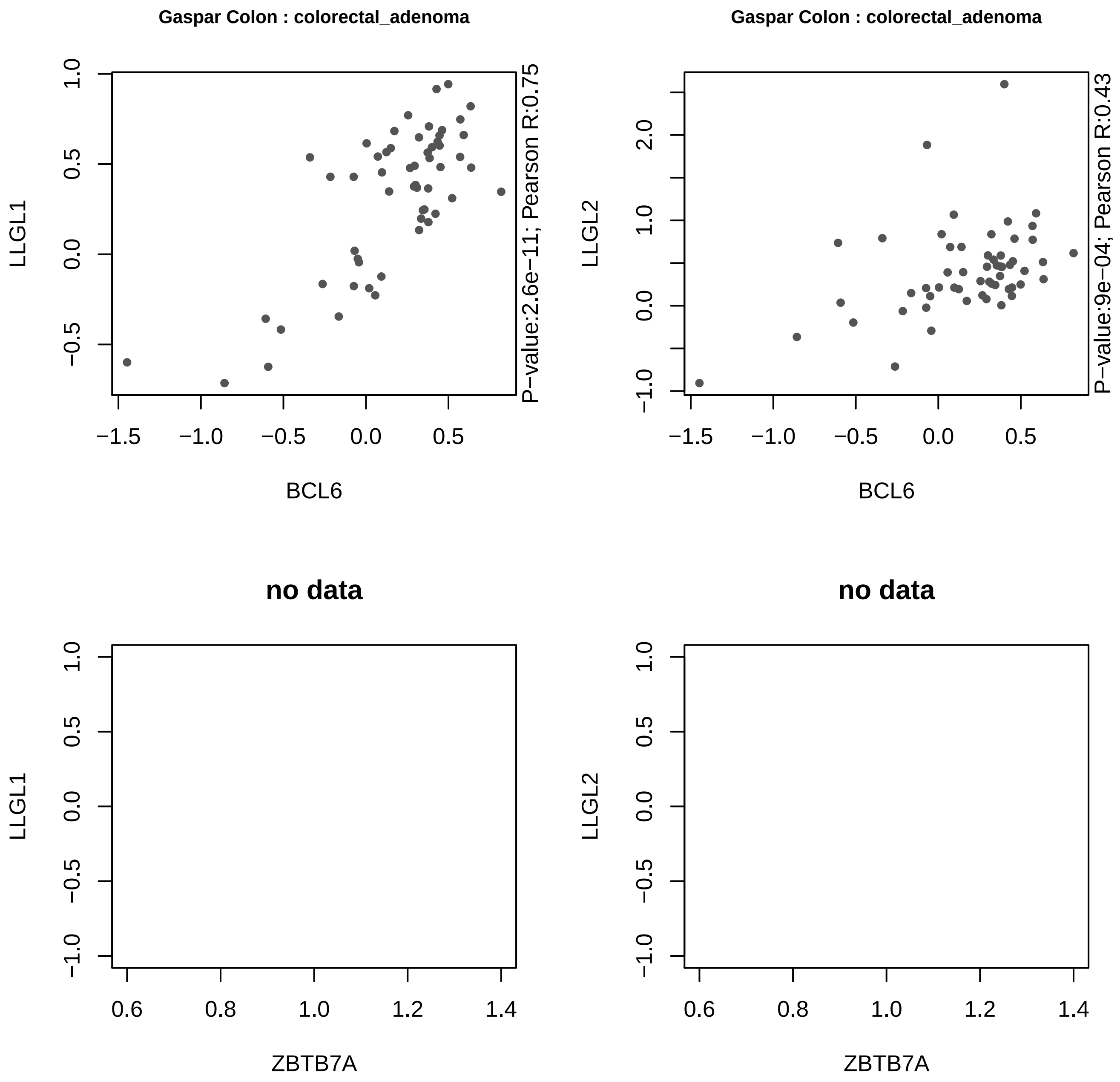
Gaspar Colon : colorectal_adenoma
LLGL1
BCL6
P−value:2.6e−11; Pearson R:0.75
Gaspar Colon : colorectal_adenoma
LLGL2
BCL6
P−value:9e−04; Pearson R:0.43
no data
LLGL1
ZBTB7A
no data
LLGL2
ZBTB7A

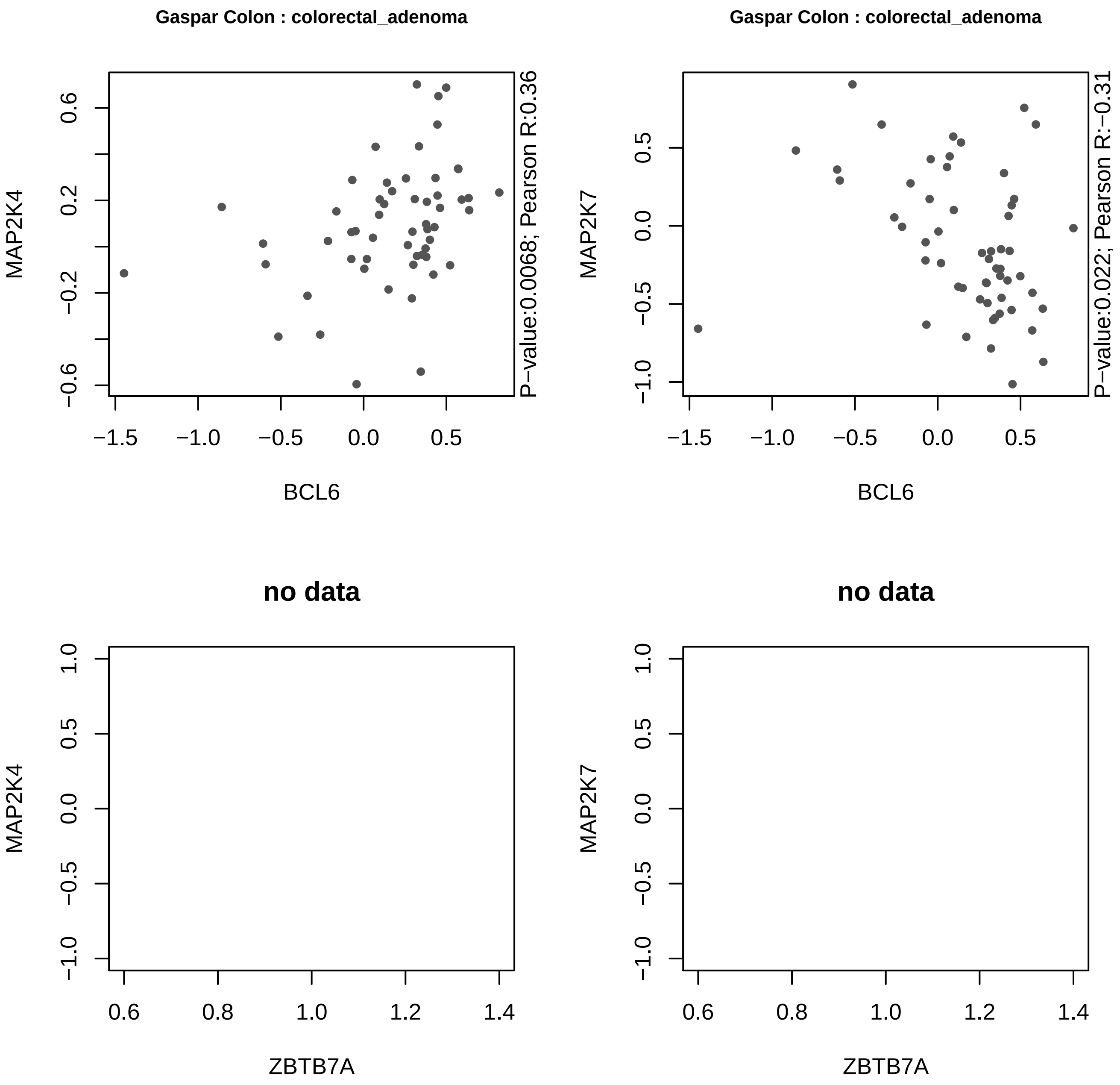
Gaspar Colon : colorectal_adenoma
MAP2K4
BCL6
P−value:0.0068; Pearson R:0.36
Gaspar Colon : colorectal_adenoma
MAP2K7
BCL6
P−value:0.022; Pearson R:−0.31
no data
MAP2K4
ZBTB7A
no data
MAP2K7
ZBTB7A

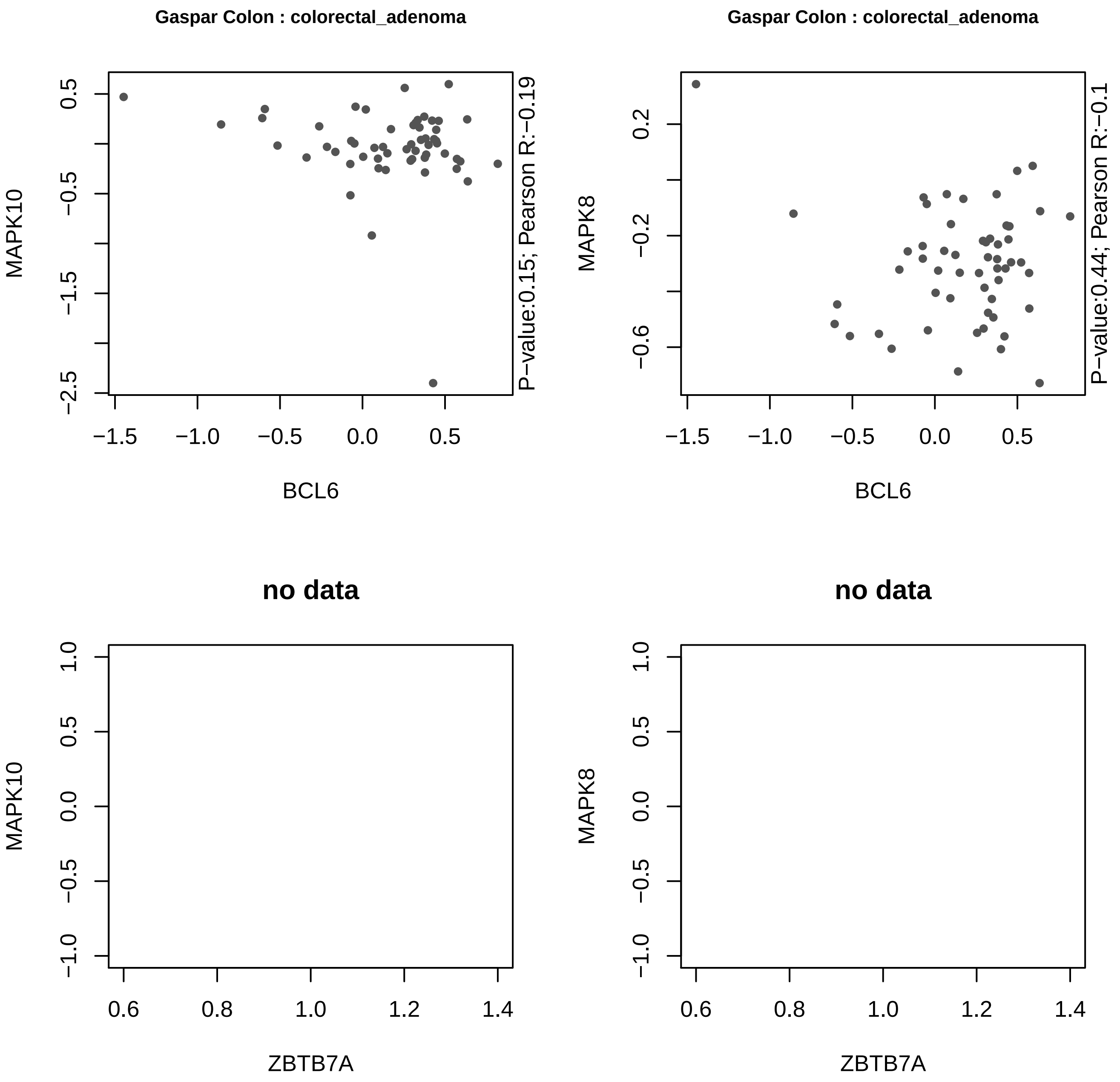
Gaspar Colon : colorectal_adenoma
MAPK10
BCL6
P−value:0.15; Pearson R:−0.19
Gaspar Colon : colorectal_adenoma
MAPK8
BCL6
P−value:0.44; Pearson R:−0.1
no data
MAPK10
ZBTB7A
no data
MAPK8
ZBTB7A

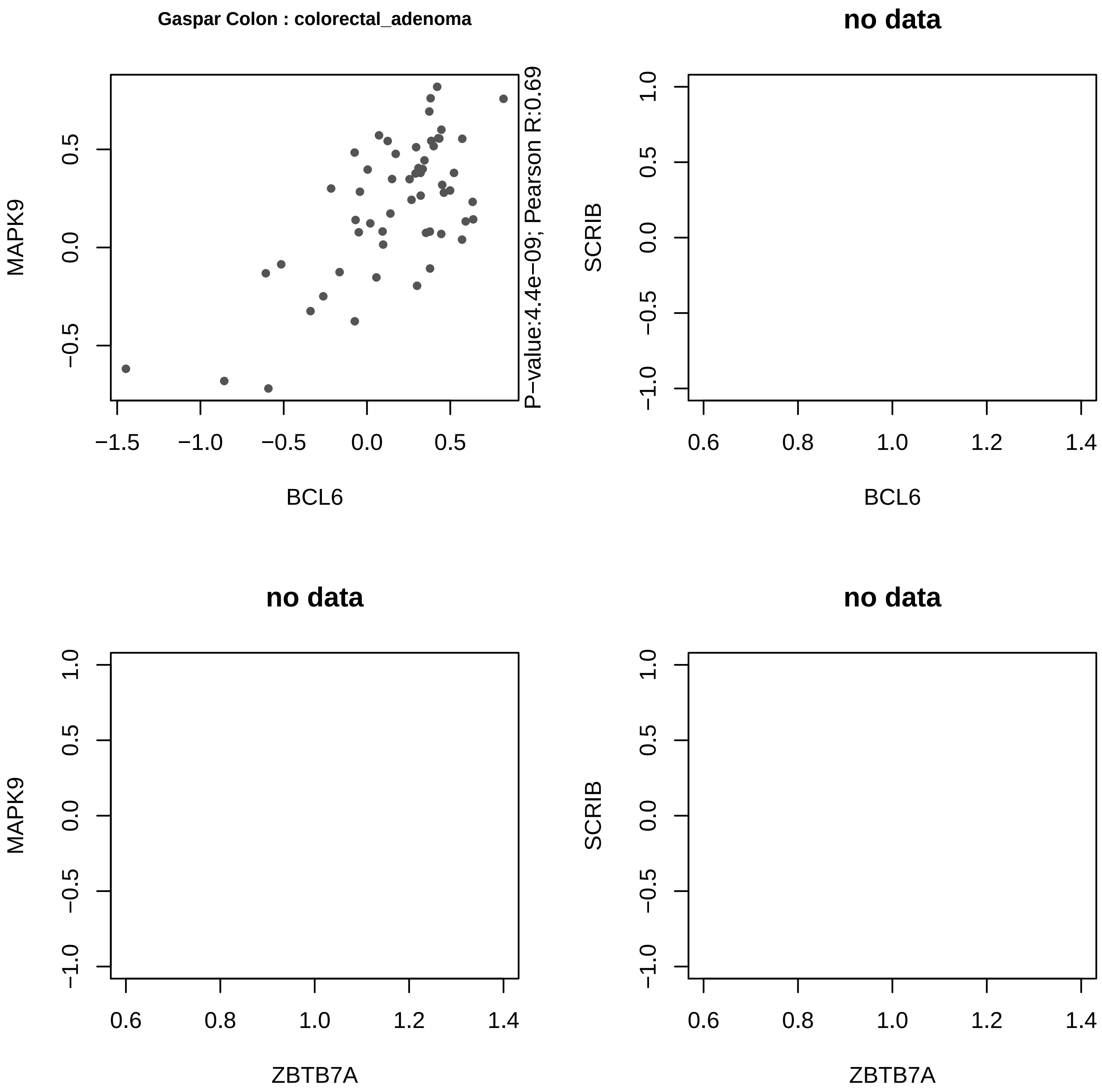
Gaspar Colon : colorectal_adenoma
MAPK9
BCL6
P−value:4.4e−09; Pearson R:0.69
no data
SCRIB
BCL6
no data
MAPK9
ZBTB7A
no data
SCRIB
ZBTB7A

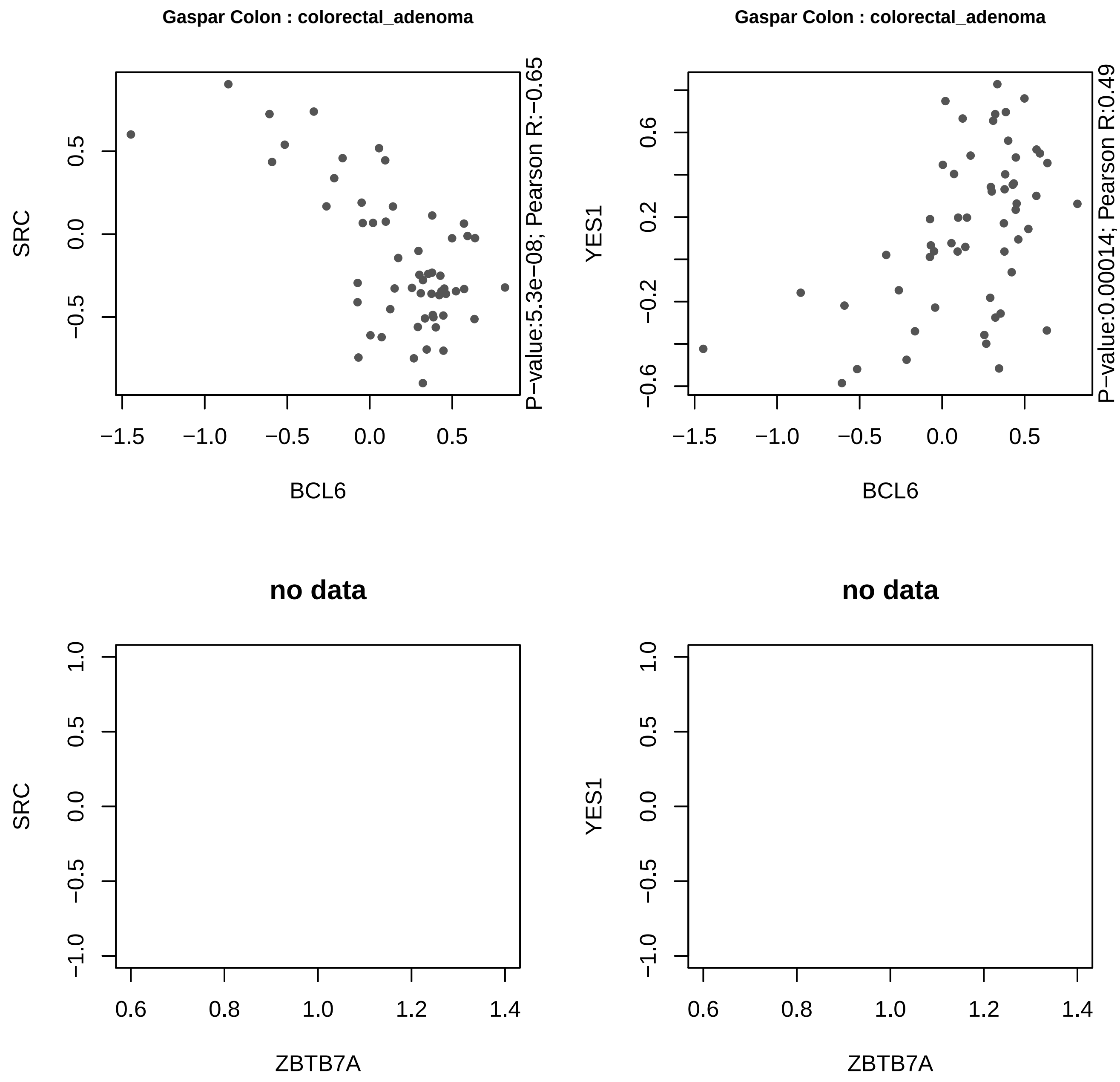
Gaspar Colon : colorectal_adenoma
SRC
BCL6
P−value:5.3e−08; Pearson R:−0.65
Gaspar Colon : colorectal_adenoma
YES1
BCL6
P−value:0.00014; Pearson R:0.49
no data
SRC
ZBTB7A
no data
YES1
ZBTB7A

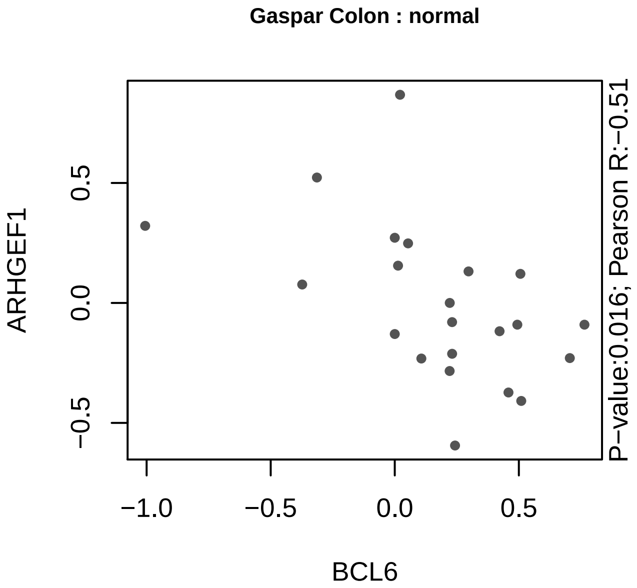
Gaspar Colon : normal
ARHGEF1
BCL6
P−value:0.016; Pearson R:−0.51
0.5
0.0
−0.5
−1.0
−0.5
0.0
0.5

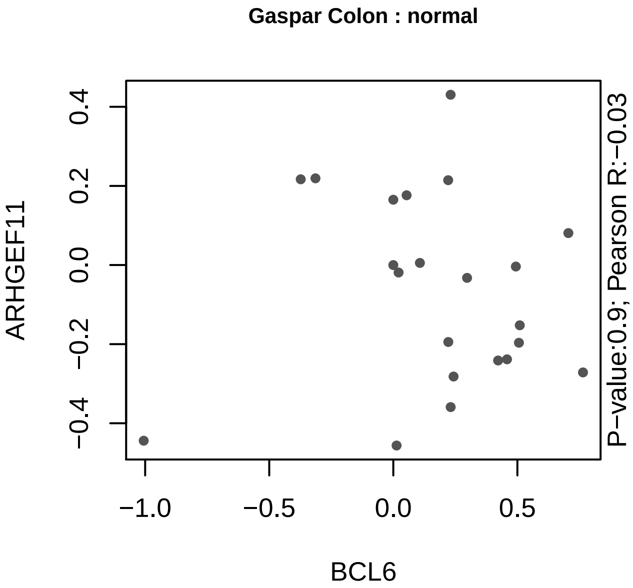
Gaspar Colon : normal
ARHGEF11
BCL6
P−value:0.9; Pearson R:−0.03
0.4
0.2
0.0
−0.2
−0.4
−1.0
−0.5
0.0
0.5

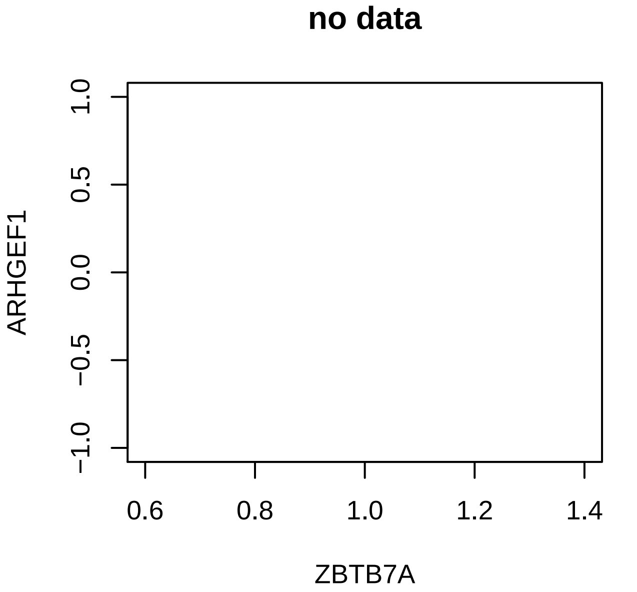
no data
ARHGEF1
ZBTB7A
1.0
0.5
0.0
−0.5
−1.0
0.6
0.8
1.0
1.2
1.4

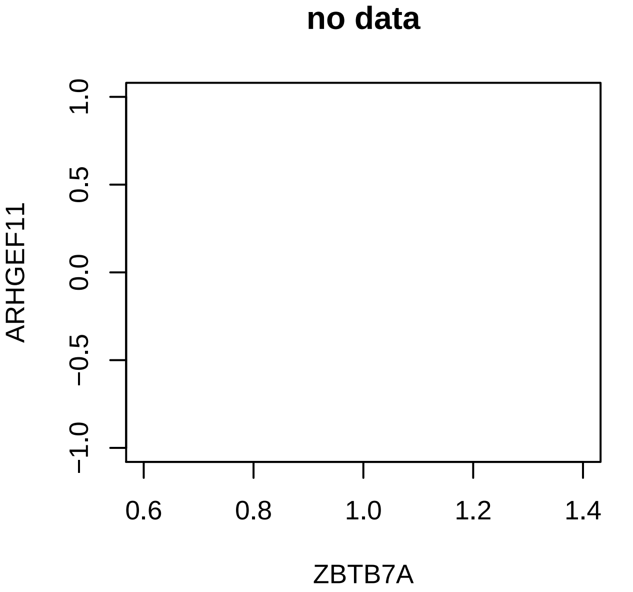
no data
ARHGEF11
ZBTB7A
1.0
0.5
0.0
−0.5
−1.0
0.6
0.8
1.0
1.2
1.4

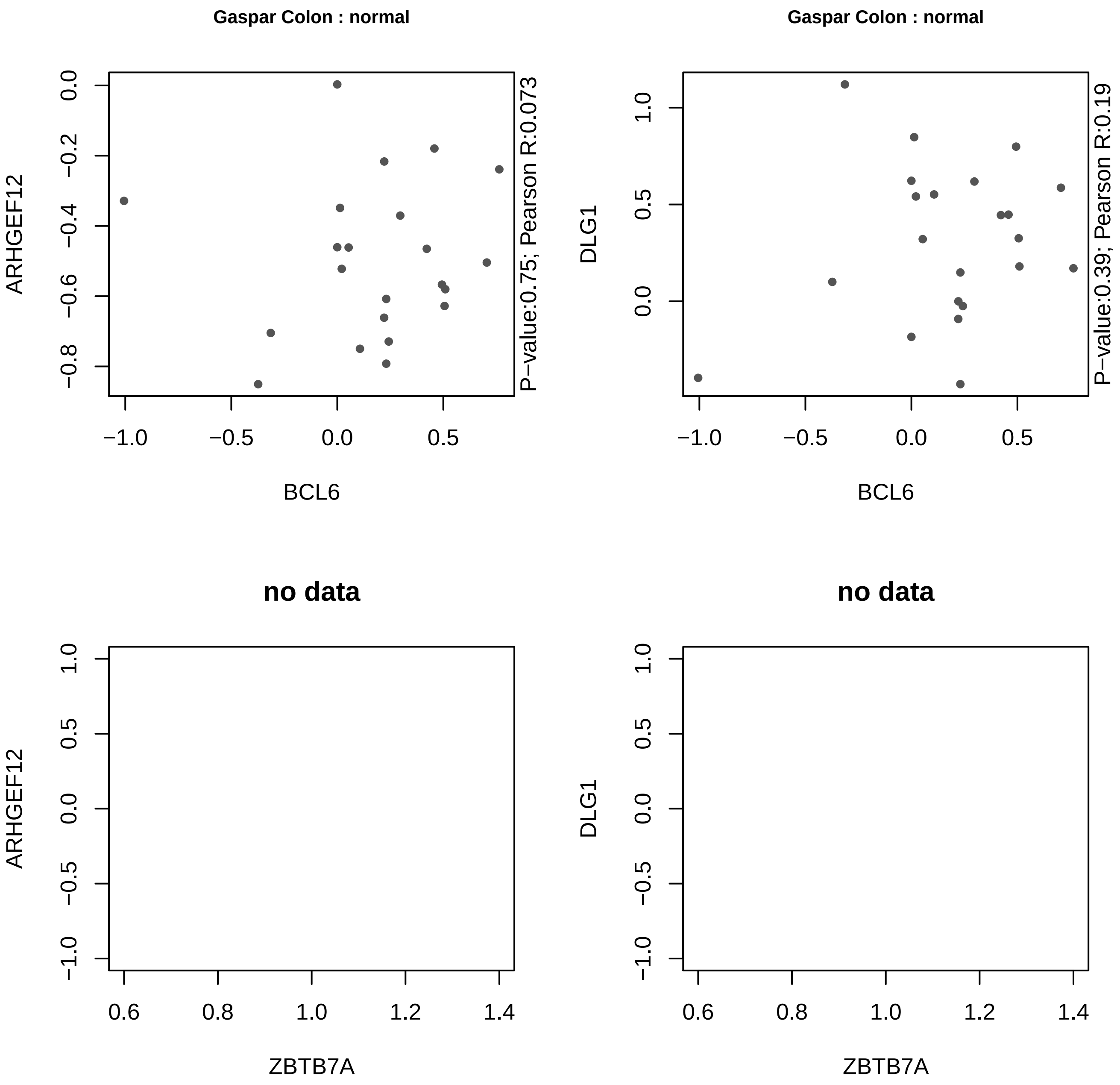
Gaspar Colon : normal
ARHGEF12
BCL6
P−value:0.75; Pearson R:0.073
Gaspar Colon : normal
DLG1
BCL6
P−value:0.39; Pearson R:0.19
no data
ARHGEF12
ZBTB7A
no data
DLG1
ZBTB7A

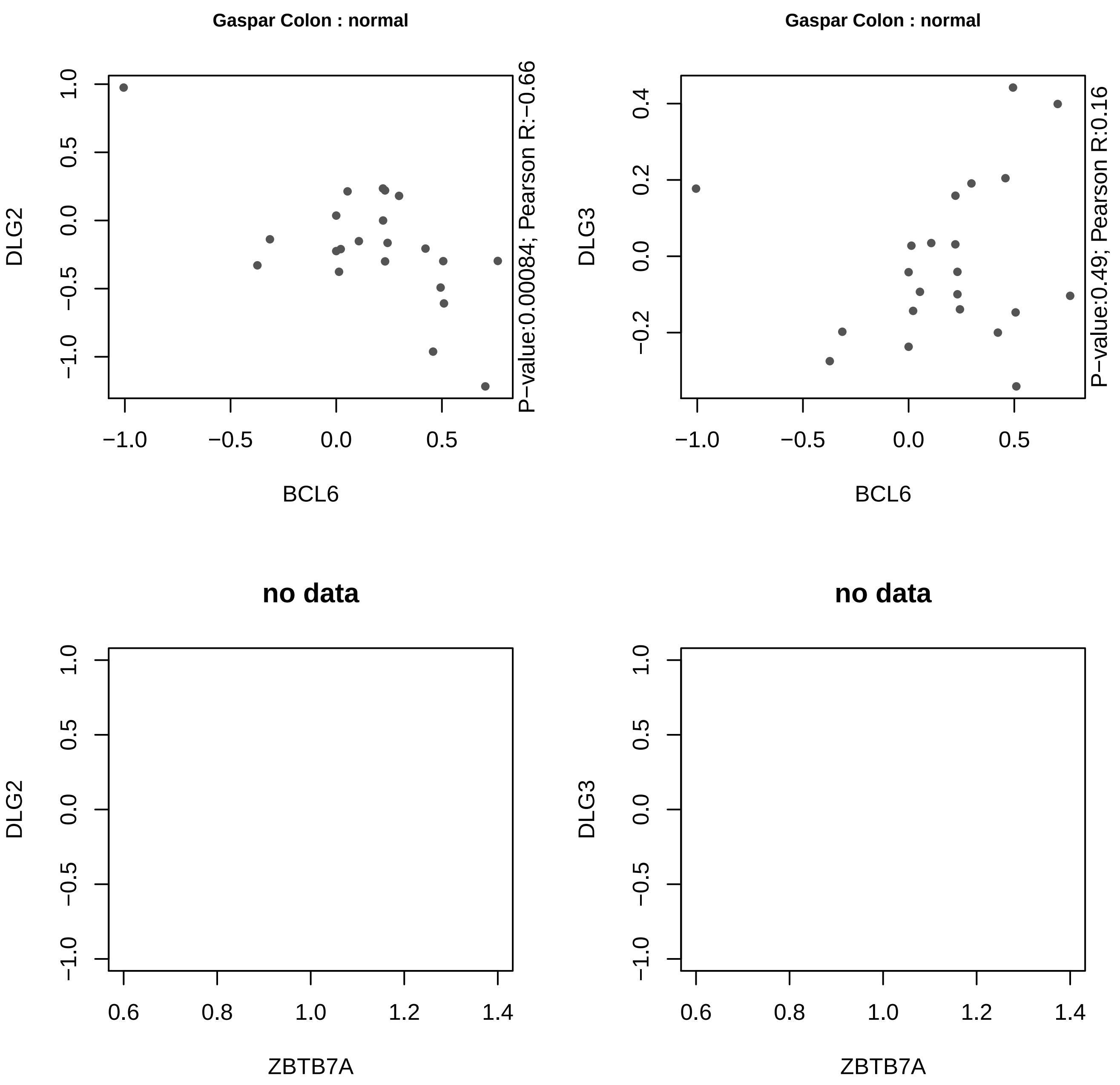
Gaspar Colon : normal
DLG2
BCL6
P−value:0.00084; Pearson R:−0.66
Gaspar Colon : normal
DLG3
BCL6
P−value:0.49; Pearson R:0.16
no data
DLG2
ZBTB7A
no data
DLG3
ZBTB7A

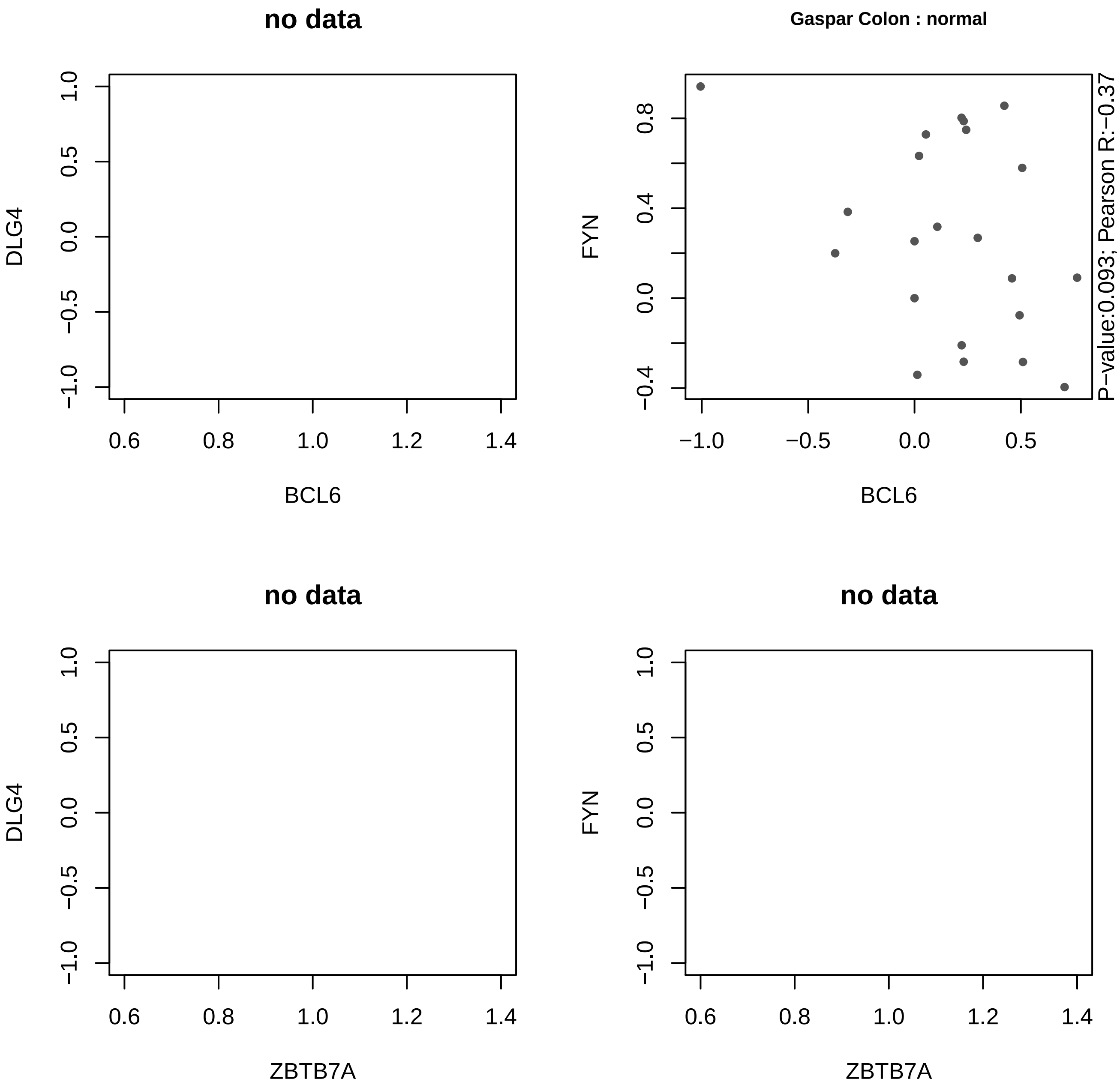
no data
DLG4
1.0
0.5
0.0
−0.5
−1.0
0.6
0.8
1.0
1.2
1.4
BCL6
Gaspar Colon : normal
FYN
0.8
0.4
0.0
−0.4
−1.0
−0.5
0.0
0.5
BCL6
P−value:0.093; Pearson R:−0.37
no data
DLG4
ZBTB7A
no data
FYN
ZBTB7A

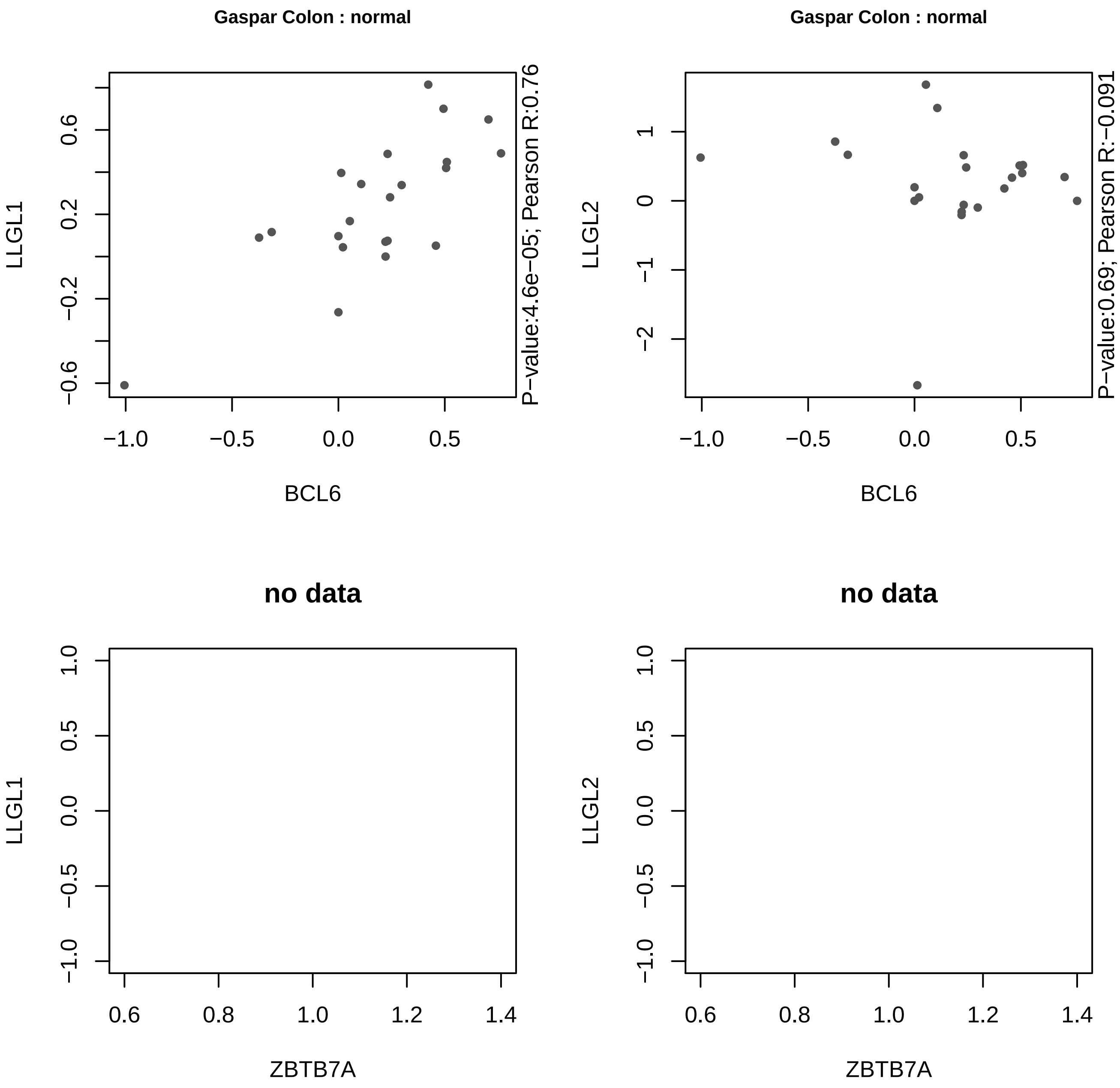
Gaspar Colon : normal
LLGL1
BCL6
0.6
0.2
−0.2
−0.6
−1.0
−0.5
0.0
0.5
P−value:4.6e−05; Pearson R:0.76
Gaspar Colon : normal
LLGL2
BCL6
1
0
−1
−2
−1.0
−0.5
0.0
0.5
P−value:0.69; Pearson R:−0.091
no data
LLGL1
ZBTB7A
1.0
0.5
0.0
−0.5
−1.0
0.6
0.8
1.0
1.2
1.4
no data
LLGL2
ZBTB7A
1.0
0.5
0.0
−0.5
−1.0
0.6
0.8
1.0
1.2
1.4

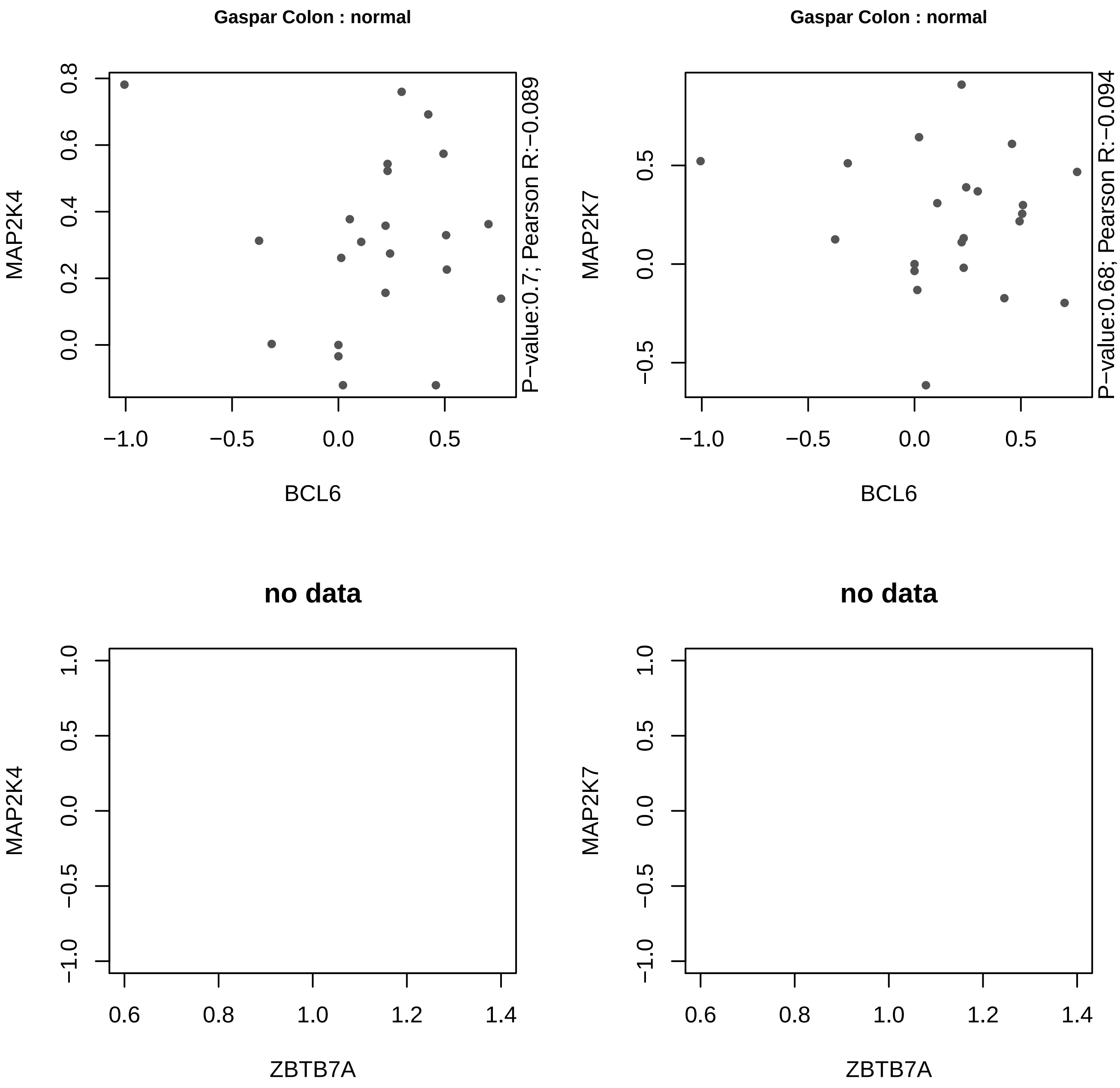
Gaspar Colon : normal
MAP2K4
BCL6
P−value:0.7; Pearson R:−0.089
Gaspar Colon : normal
MAP2K7
BCL6
P−value:0.68; Pearson R:−0.094
no data
MAP2K4
ZBTB7A
no data
MAP2K7
ZBTB7A

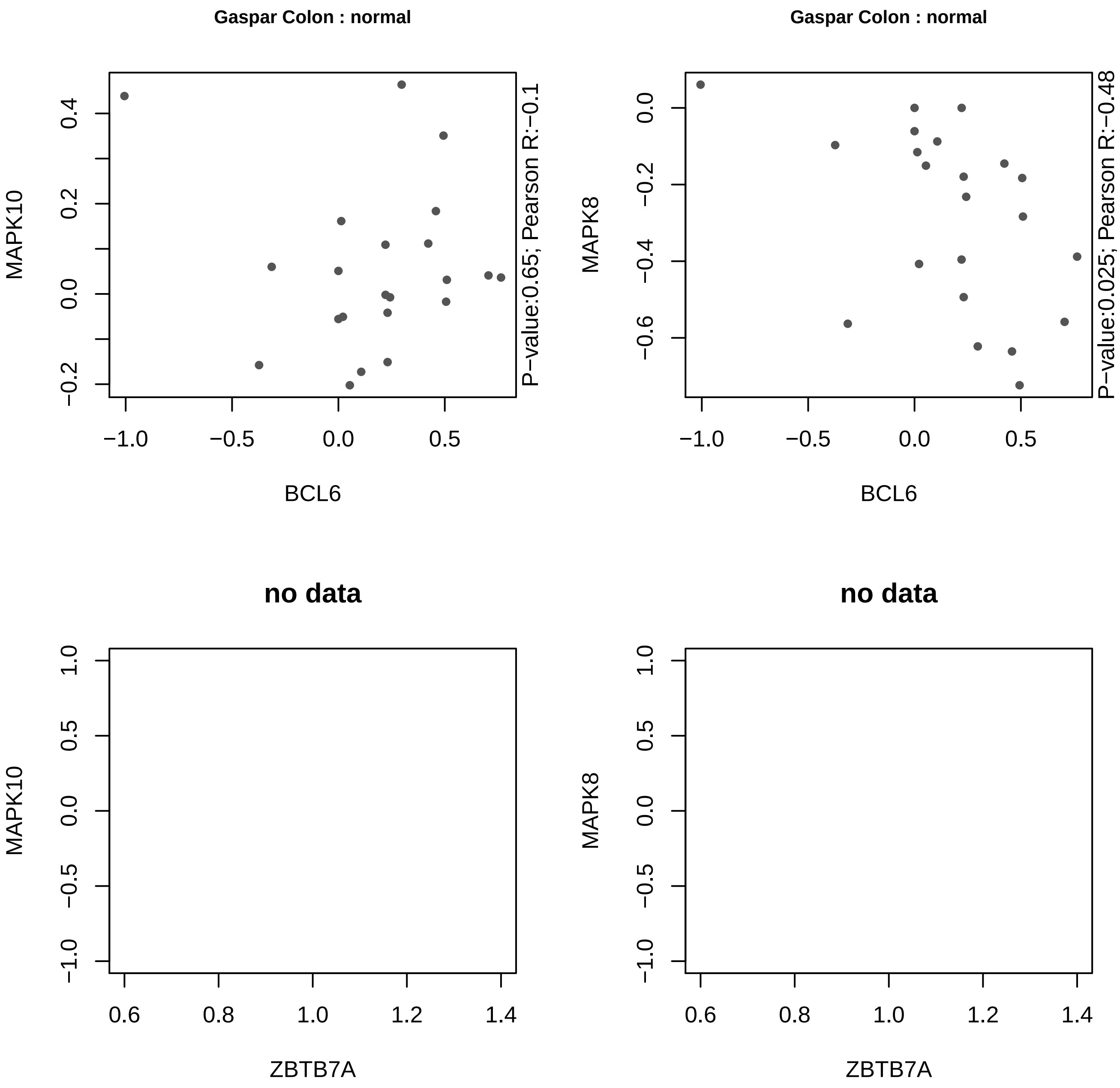
Gaspar Colon : normal
MAPK10
BCL6
P−value:0.65; Pearson R:−0.1
Gaspar Colon : normal
MAPK8
BCL6
P−value:0.025; Pearson R:−0.48
no data
MAPK10
ZBTB7A
no data
MAPK8
ZBTB7A

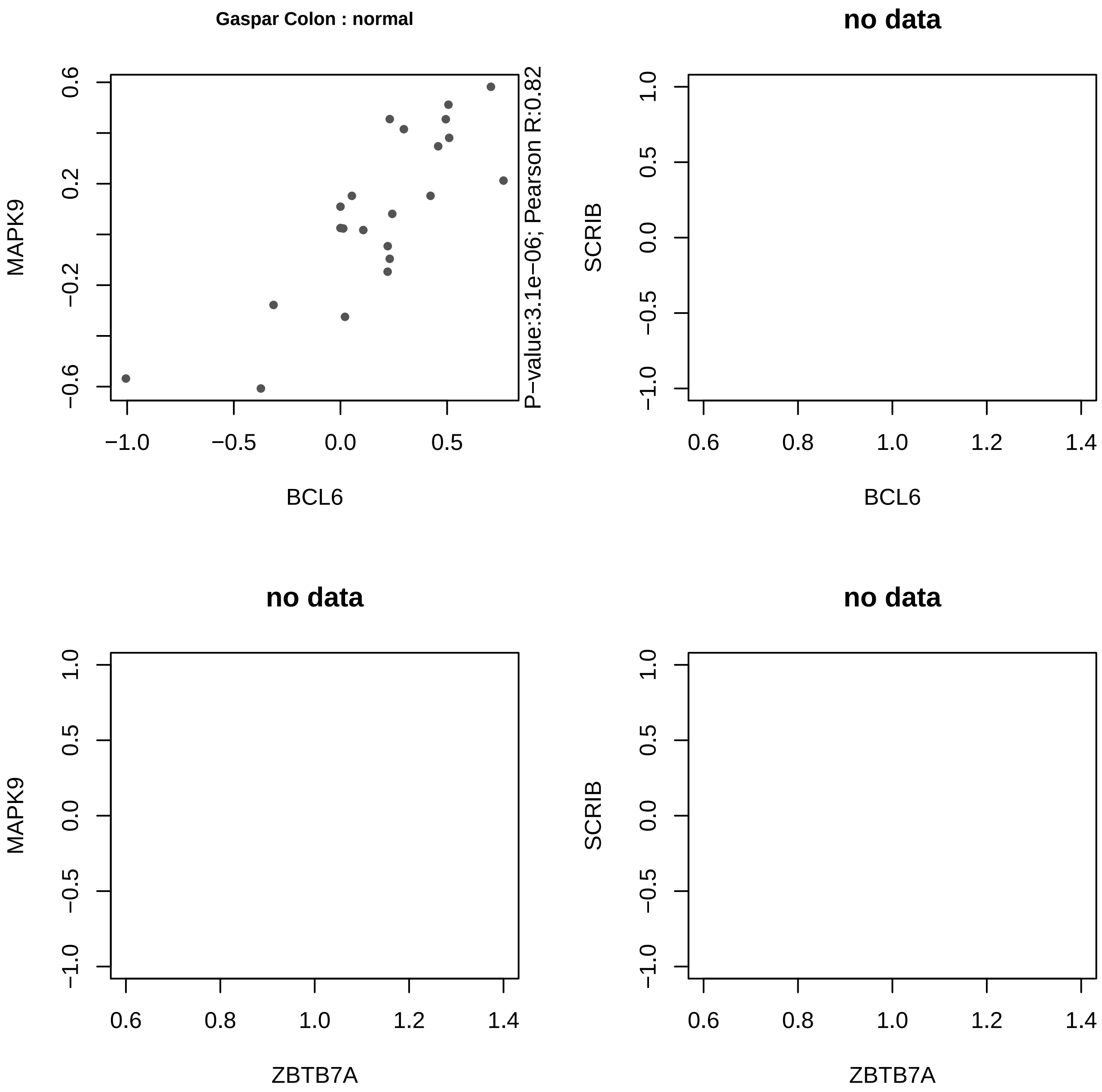
Gaspar Colon : normal
P−value:3.1e−06; Pearson R:0.82
MAPK9
BCL6
no data
SCRIB
BCL6
no data
MAPK9
ZBTB7A
no data
SCRIB
ZBTB7A

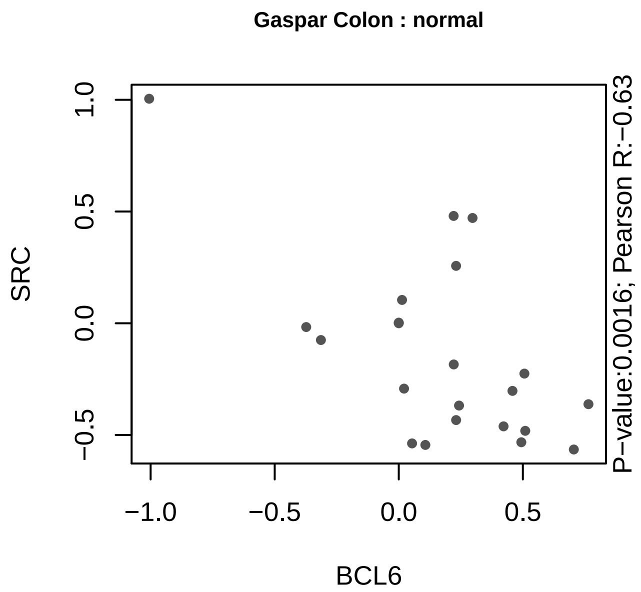
Gaspar Colon : normal
SRC
BCL6
P−value:0.0016; Pearson R:−0.63
1.0
0.5
0.0
−0.5
−1.0
−0.5
0.0
0.5

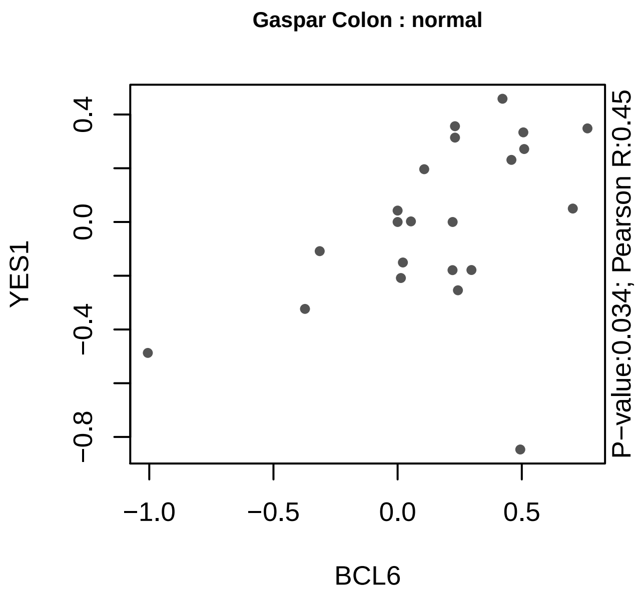
Gaspar Colon : normal
YES1
BCL6
P−value:0.034; Pearson R:0.45
0.4
0.0
−0.4
−0.8
−1.0
−0.5
0.0
0.5

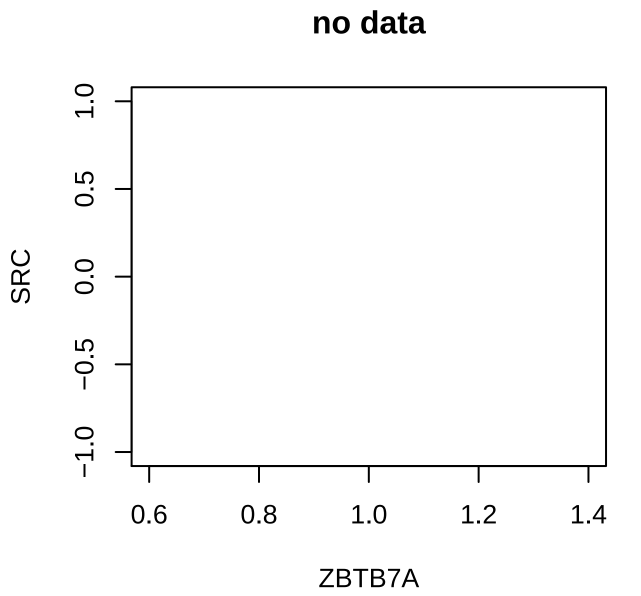
no data
SRC
ZBTB7A
1.0
0.5
0.0
−0.5
−1.0
0.6
0.8
1.0
1.2
1.4

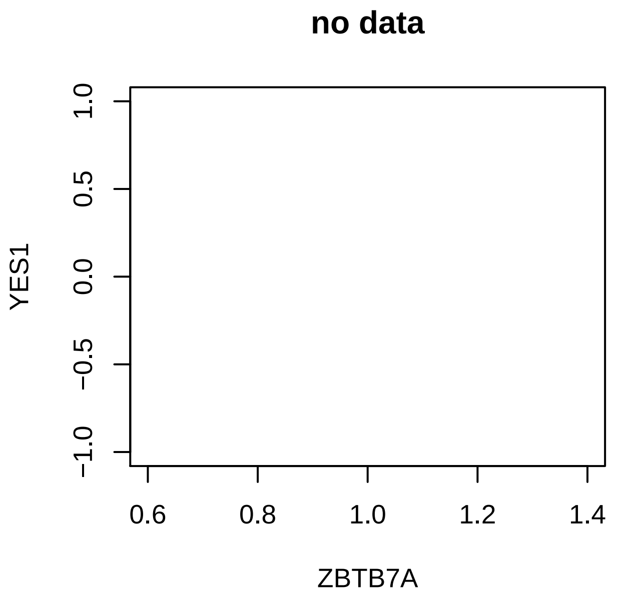
no data
YES1
ZBTB7A
1.0
0.5
0.0
−0.5
−1.0
0.6
0.8
1.0
1.2
1.4

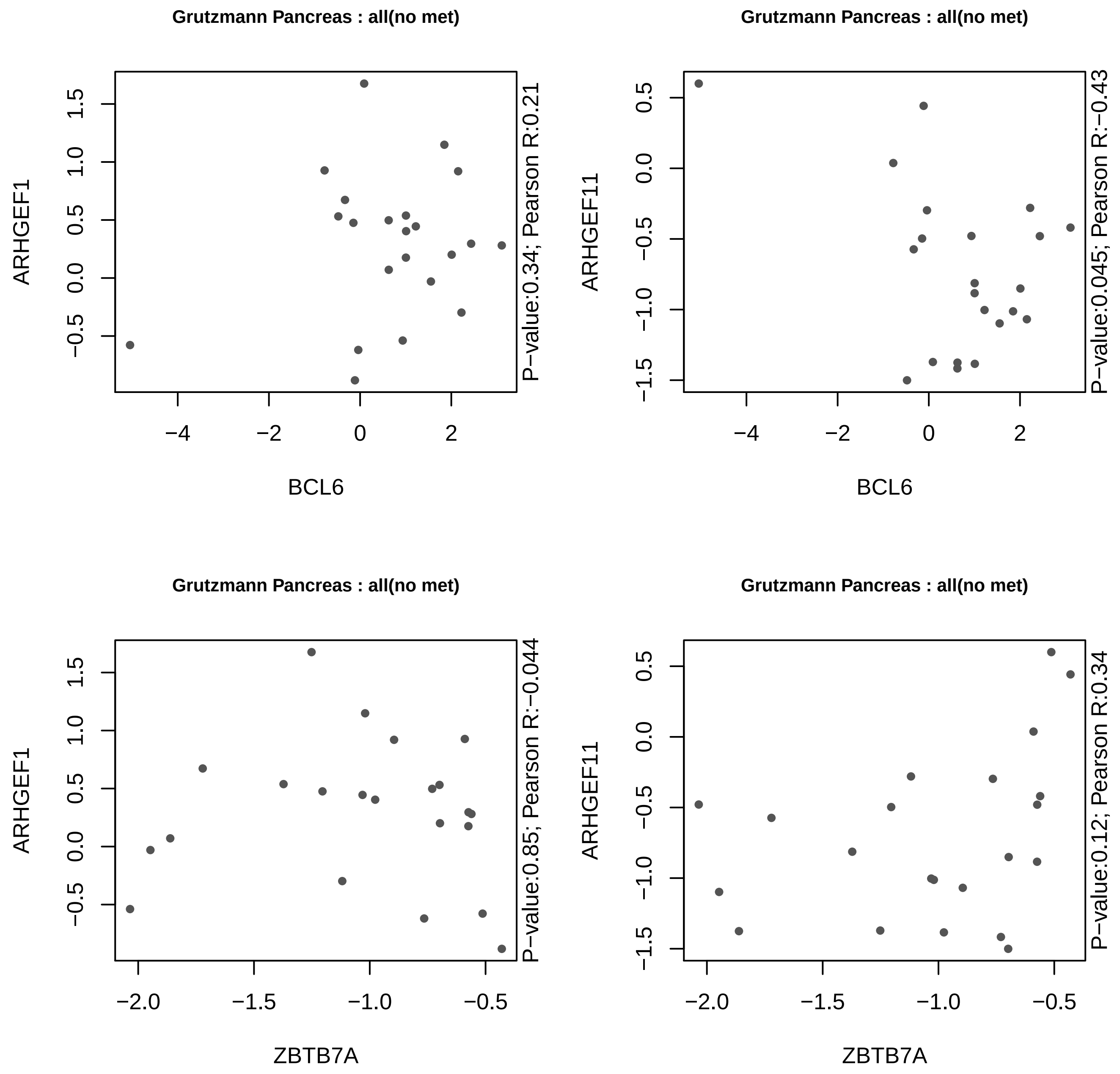
Grutzmann Pancreas : all(no met)
ARHGEF1
BCL6
P−value:0.34; Pearson R:0.21
Grutzmann Pancreas : all(no met)
ARHGEF11
BCL6
P−value:0.045; Pearson R:−0.43
Grutzmann Pancreas : all(no met)
ARHGEF1
ZBTB7A
P−value:0.85; Pearson R:−0.044
Grutzmann Pancreas : all(no met)
ARHGEF11
ZBTB7A
P−value:0.12; Pearson R:0.34

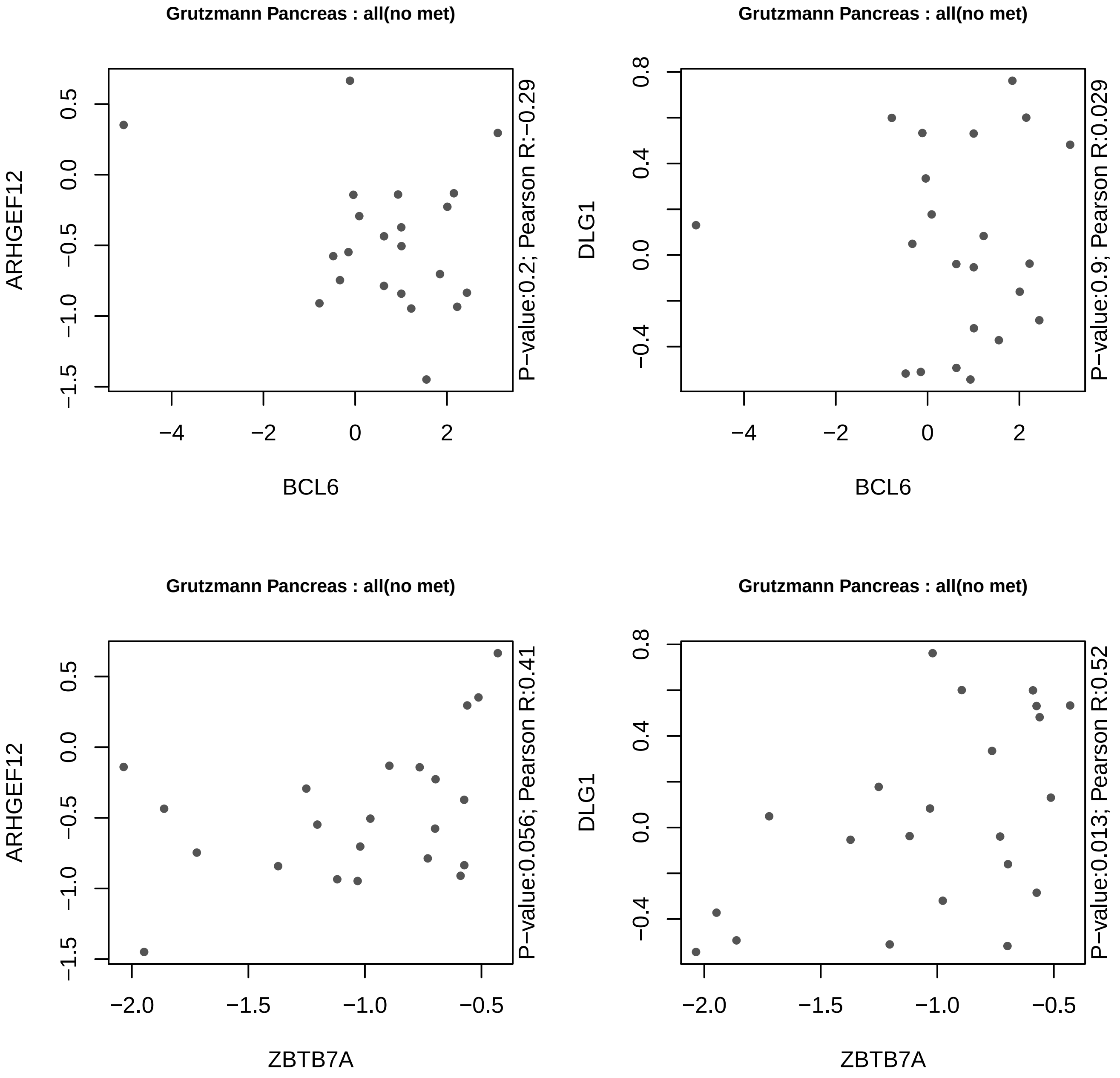
Grutzmann Pancreas : all(no met)
ARHGEF12
BCL6
P−value:0.2; Pearson R:−0.29
0.5
0.0
−0.5
−1.0
−1.5
−4
−2
0
2
Grutzmann Pancreas : all(no met)
DLG1
BCL6
P−value:0.9; Pearson R:0.029
0.8
0.4
0.0
−0.4
Grutzmann Pancreas : all(no met)
ARHGEF12
ZBTB7A
P−value:0.056; Pearson R:0.41
−2.0
−1.5
−1.0
−0.5
Grutzmann Pancreas : all(no met)
DLG1
ZBTB7A
P−value:0.013; Pearson R:0.52

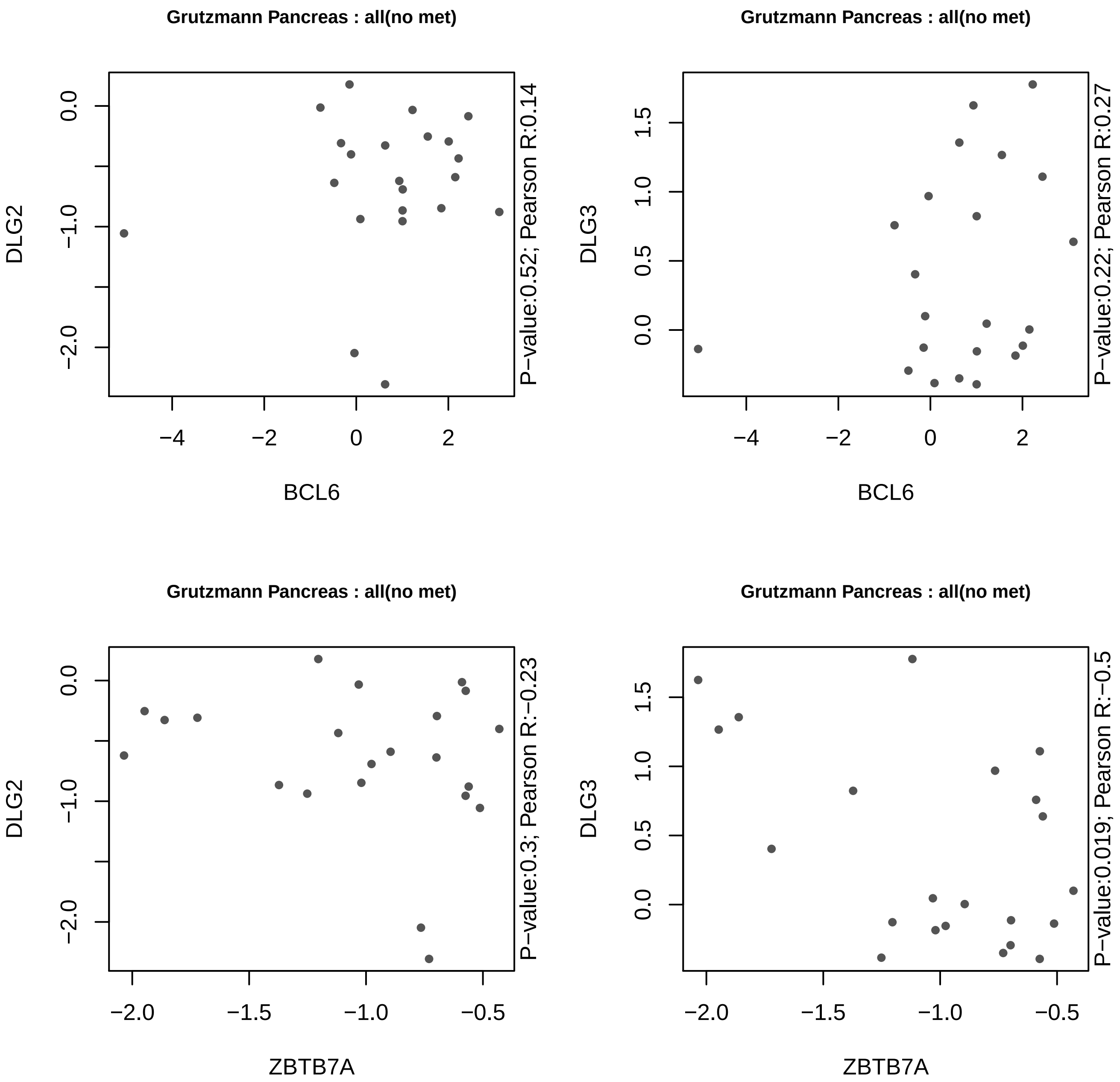

Grutzmann Pancreas : all(no met)
DLG2
BCL6
P−value:0.52; Pearson R:0.14
Grutzmann Pancreas : all(no met)
DLG3
BCL6
P−value:0.22; Pearson R:0.27
Grutzmann Pancreas : all(no met)
DLG2
ZBTB7A
P−value:0.3; Pearson R:−0.23
Grutzmann Pancreas : all(no met)
DLG3
ZBTB7A
P−value:0.019; Pearson R:−0.5

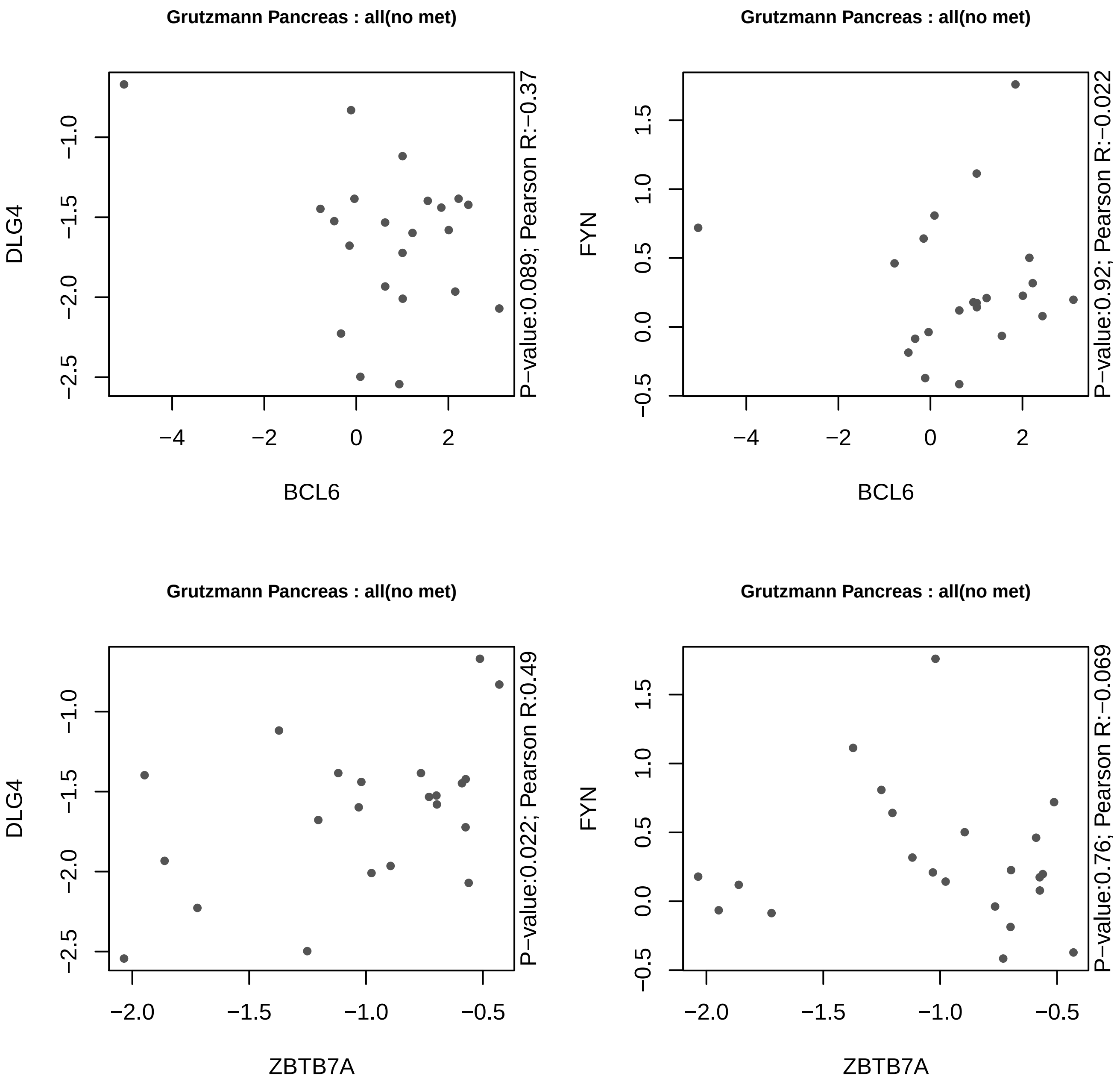
Grutzmann Pancreas : all(no met)
DLG4
BCL6
P−value:0.089; Pearson R:−0.37
Grutzmann Pancreas : all(no met)
FYN
BCL6
P−value:0.92; Pearson R:−0.022
Grutzmann Pancreas : all(no met)
DLG4
ZBTB7A
P−value:0.022; Pearson R:0.49
Grutzmann Pancreas : all(no met)
FYN
ZBTB7A
P−value:0.76; Pearson R:−0.069

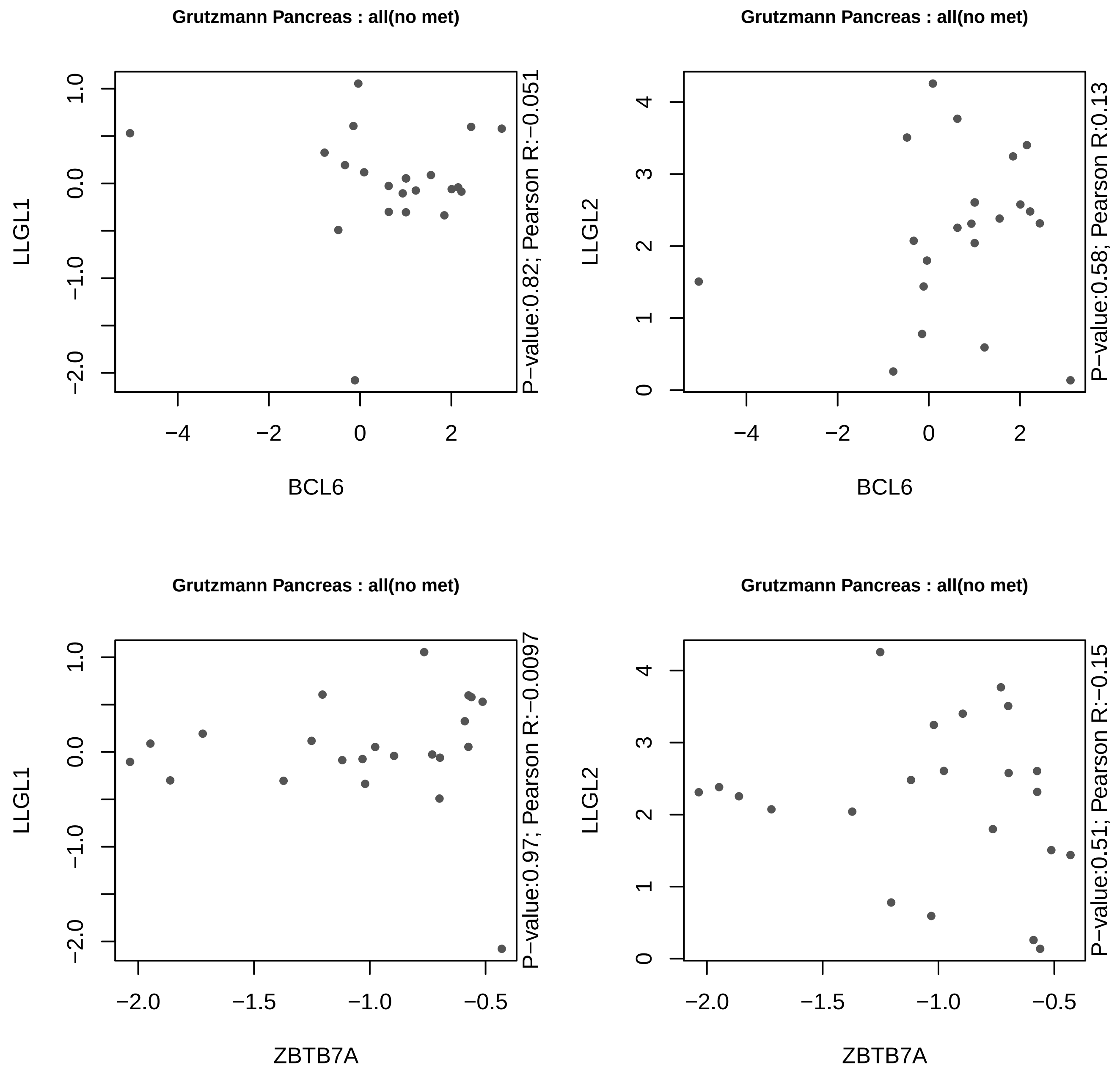
Grutzmann Pancreas : all(no met)
LLGL1
BCL6
P−value:0.82; Pearson R:−0.051
Grutzmann Pancreas : all(no met)
LLGL2
BCL6
P−value:0.58; Pearson R:0.13
Grutzmann Pancreas : all(no met)
LLGL1
ZBTB7A
P−value:0.97; Pearson R:−0.0097
Grutzmann Pancreas : all(no met)
LLGL2
ZBTB7A
P−value:0.51; Pearson R:−0.15

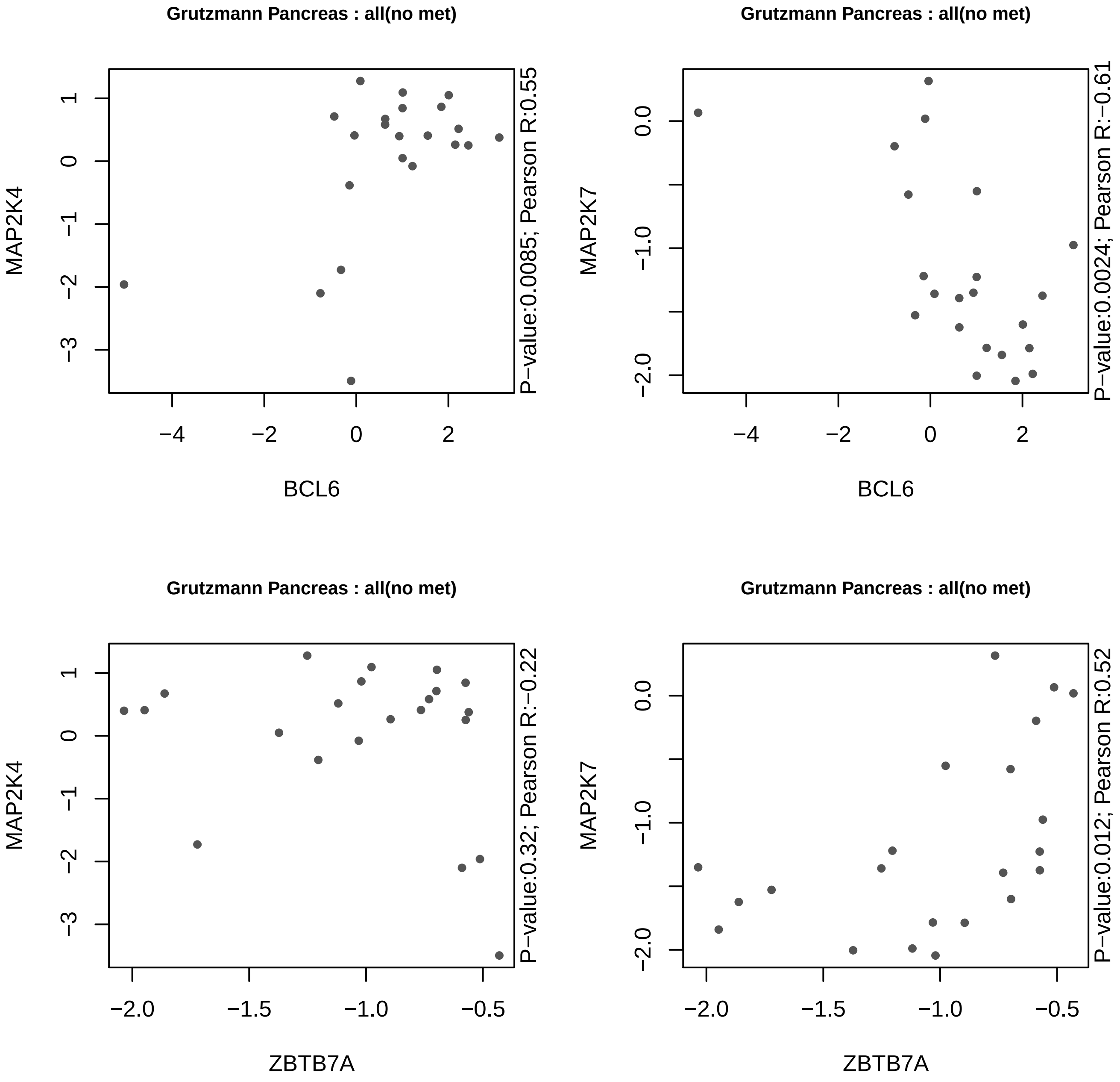
Grutzmann Pancreas : all(no met)
MAP2K4
BCL6
P−value:0.0085; Pearson R:0.55
Grutzmann Pancreas : all(no met)
MAP2K7
BCL6
P−value:0.0024; Pearson R:−0.61
Grutzmann Pancreas : all(no met)
MAP2K4
ZBTB7A
P−value:0.32; Pearson R:−0.22
Grutzmann Pancreas : all(no met)
MAP2K7
ZBTB7A
P−value:0.012; Pearson R:0.52

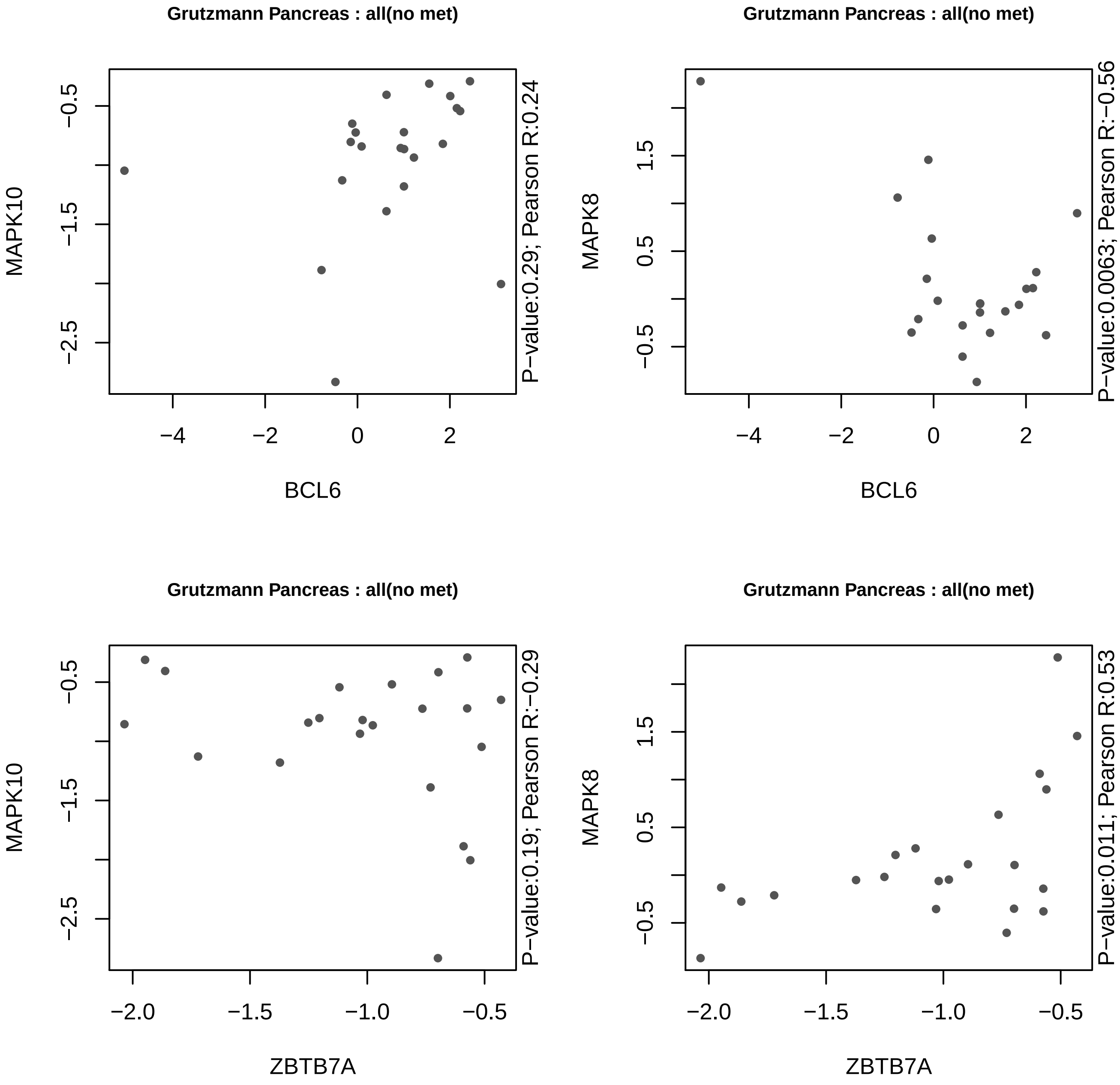
Grutzmann Pancreas : all(no met)
MAPK10
BCL6
P−value:0.29; Pearson R:0.24
Grutzmann Pancreas : all(no met)
MAPK8
BCL6
P−value:0.0063; Pearson R:−0.56
Grutzmann Pancreas : all(no met)
MAPK10
ZBTB7A
P−value:0.19; Pearson R:−0.29
Grutzmann Pancreas : all(no met)
MAPK8
ZBTB7A
P−value:0.011; Pearson R:0.53

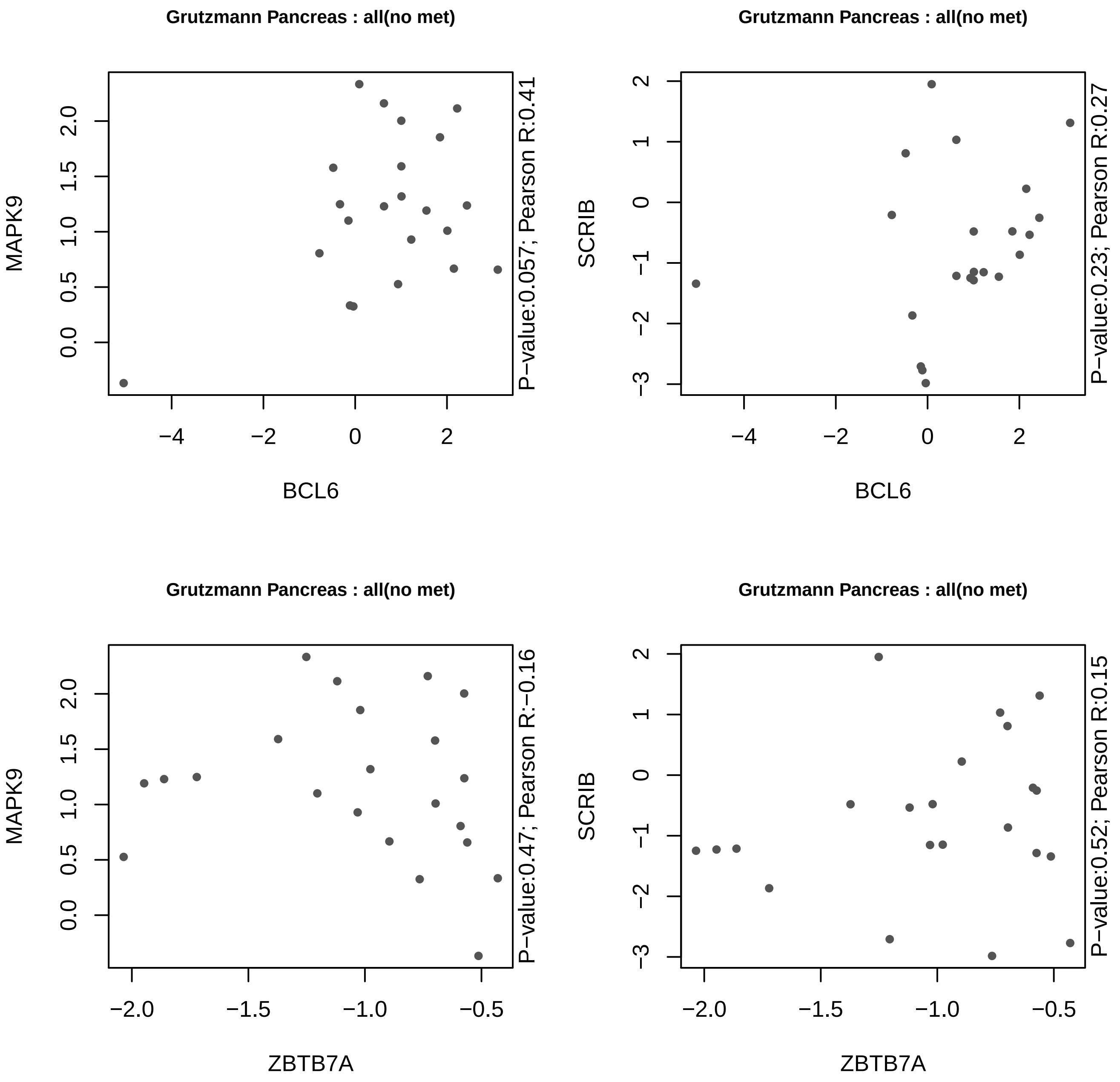
Grutzmann Pancreas : all(no met)
MAPK9
BCL6
P−value:0.057; Pearson R:0.41
Grutzmann Pancreas : all(no met)
SCRIB
BCL6
P−value:0.23; Pearson R:0.27
Grutzmann Pancreas : all(no met)
MAPK9
ZBTB7A
P−value:0.47; Pearson R:−0.16
Grutzmann Pancreas : all(no met)
SCRIB
ZBTB7A
P−value:0.52; Pearson R:0.15

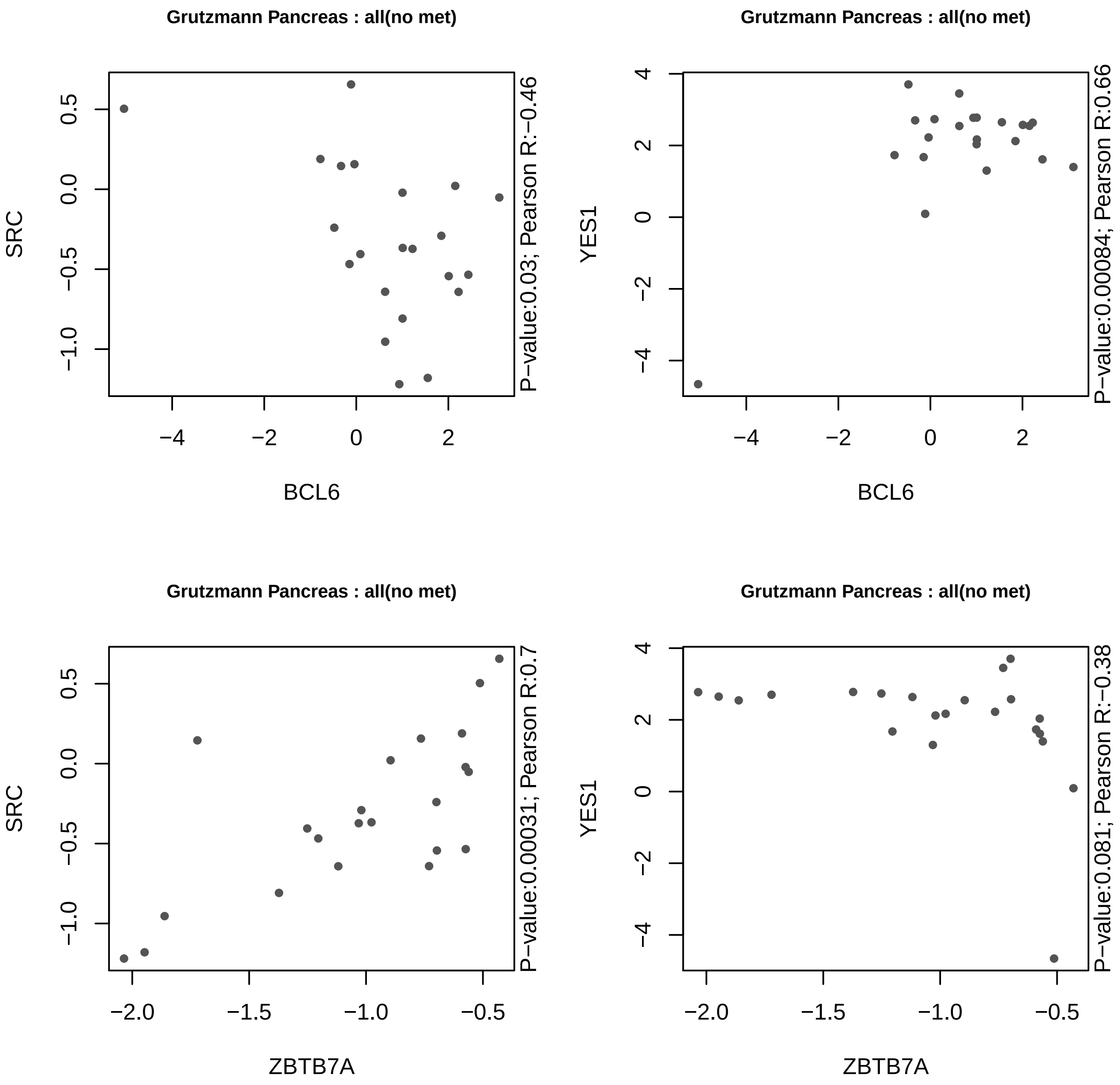
Grutzmann Pancreas : all(no met)
SRC
BCL6
P−value:0.03; Pearson R:−0.46
Grutzmann Pancreas : all(no met)
YES1
BCL6
P−value:0.00084; Pearson R:0.66
Grutzmann Pancreas : all(no met)
SRC
ZBTB7A
P−value:0.00031; Pearson R:0.7
Grutzmann Pancreas : all(no met)
YES1
ZBTB7A
P−value:0.081; Pearson R:−0.38

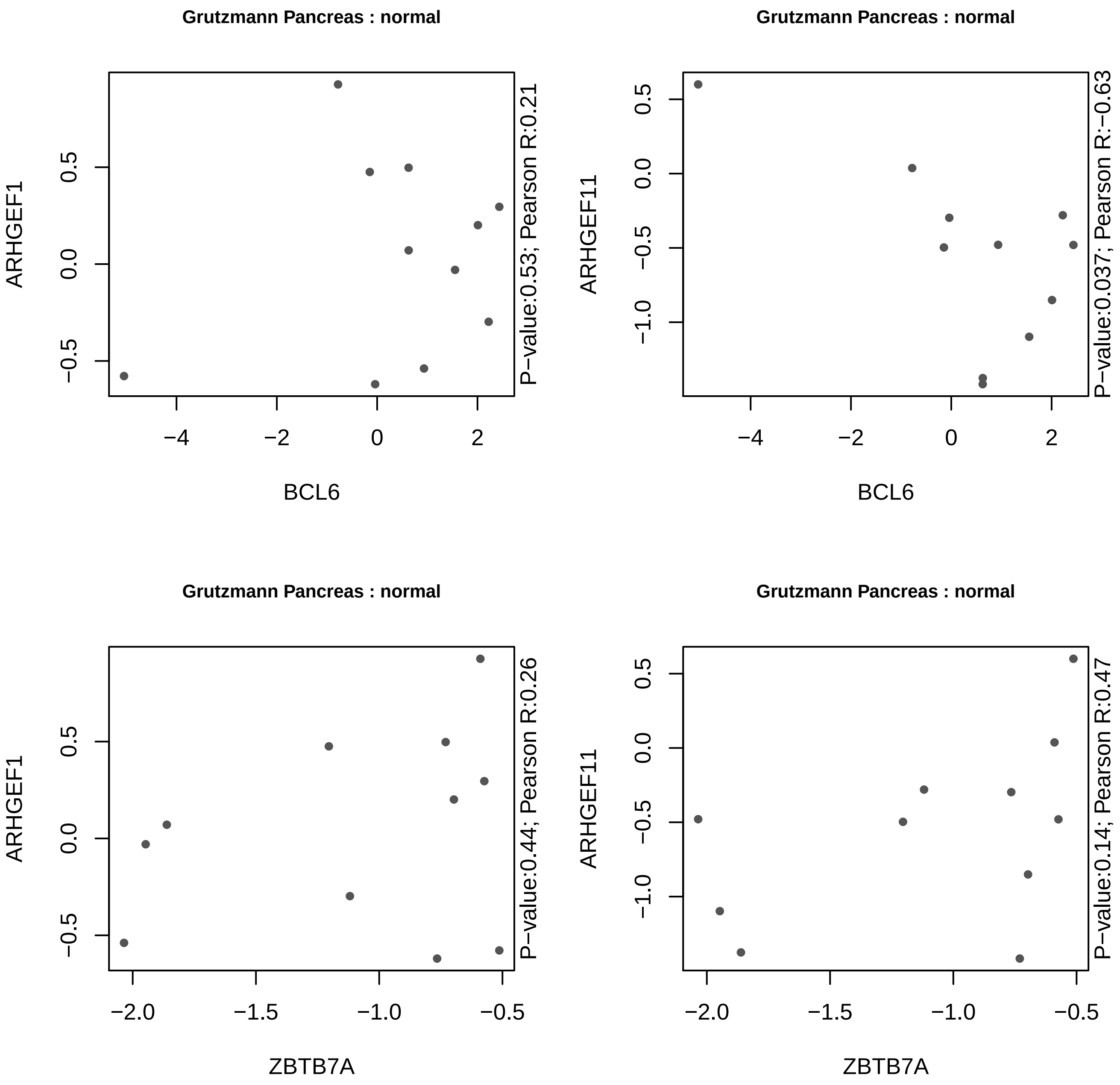
Grutzmann Pancreas : normal
ARHGEF1
BCL6
P−value:0.53; Pearson R:0.21
Grutzmann Pancreas : normal
ARHGEF11
BCL6
P−value:0.037; Pearson R:−0.63
Grutzmann Pancreas : normal
ARHGEF1
ZBTB7A
P−value:0.44; Pearson R:0.26
Grutzmann Pancreas : normal
ARHGEF11
ZBTB7A
P−value:0.14; Pearson R:0.47

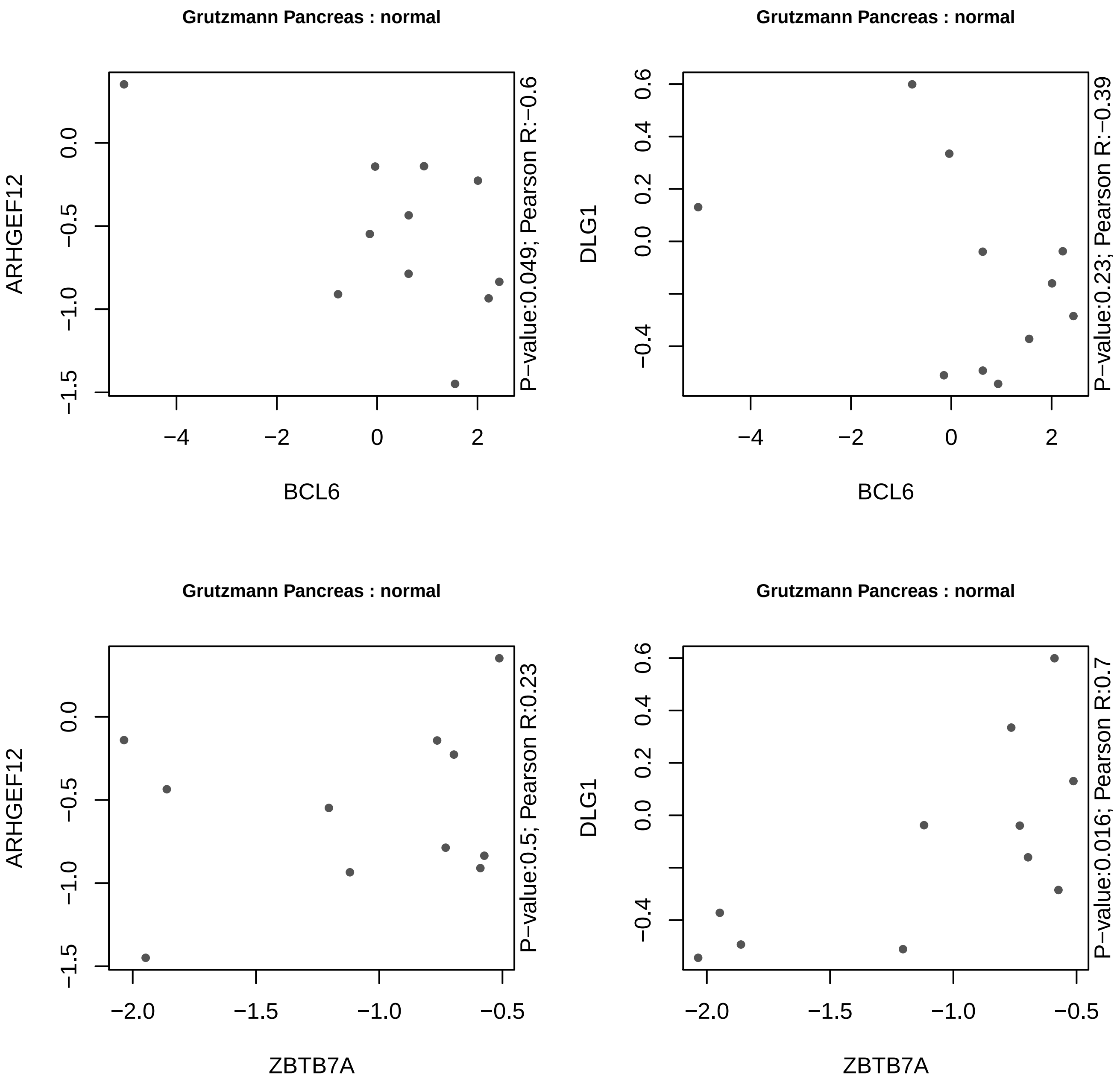
Grutzmann Pancreas : normal
ARHGEF12
BCL6
P−value:0.049; Pearson R:−0.6
Grutzmann Pancreas : normal
DLG1
BCL6
P−value:0.23; Pearson R:−0.39
Grutzmann Pancreas : normal
ARHGEF12
ZBTB7A
P−value:0.5; Pearson R:0.23
Grutzmann Pancreas : normal
DLG1
ZBTB7A
P−value:0.016; Pearson R:0.7

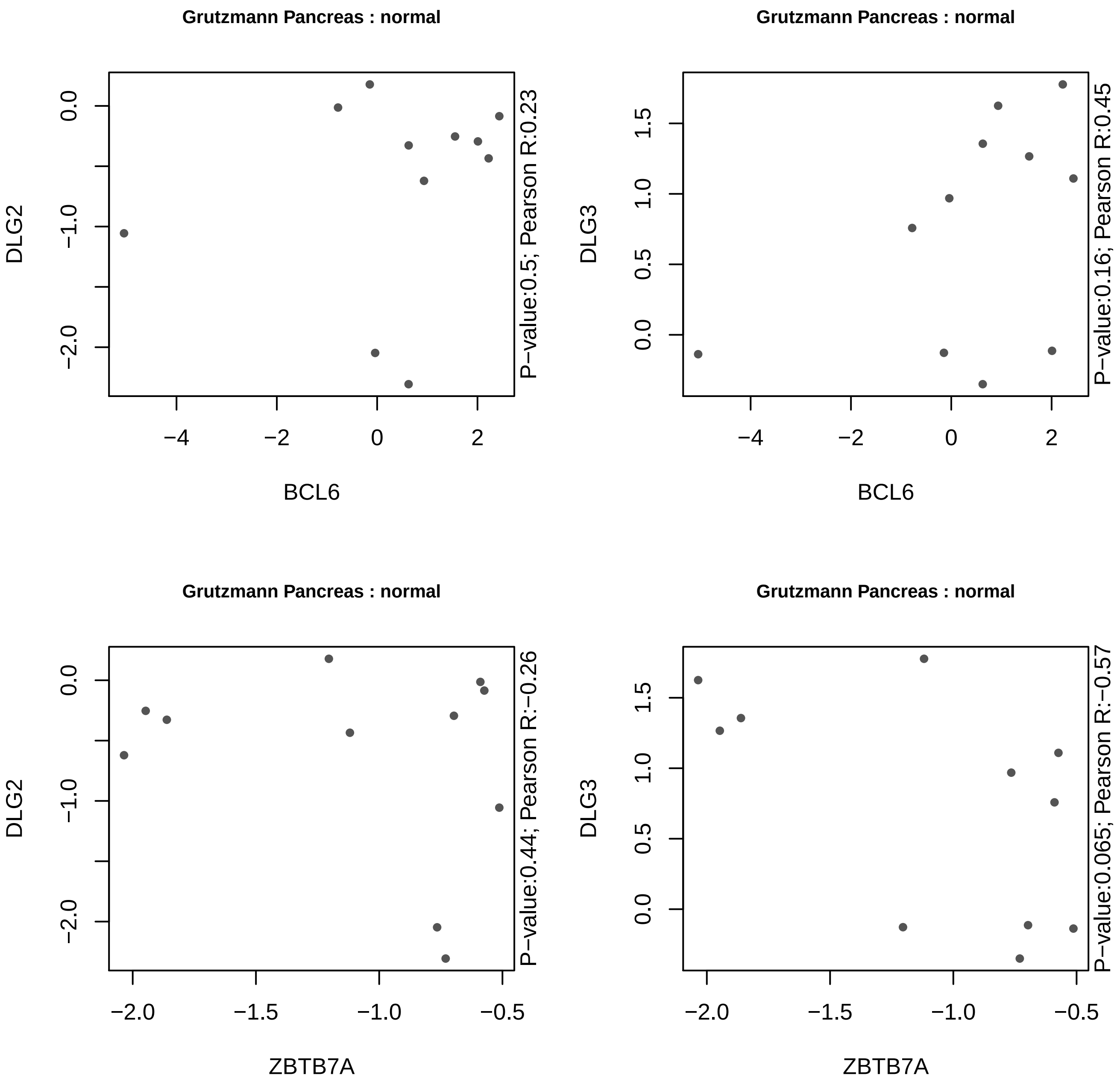
Grutzmann Pancreas : normal
DLG2
BCL6
P−value:0.5; Pearson R:0.23
Grutzmann Pancreas : normal
DLG3
BCL6
P−value:0.16; Pearson R:0.45
Grutzmann Pancreas : normal
DLG2
ZBTB7A
P−value:0.44; Pearson R:−0.26
Grutzmann Pancreas : normal
DLG3
ZBTB7A
P−value:0.065; Pearson R:−0.57

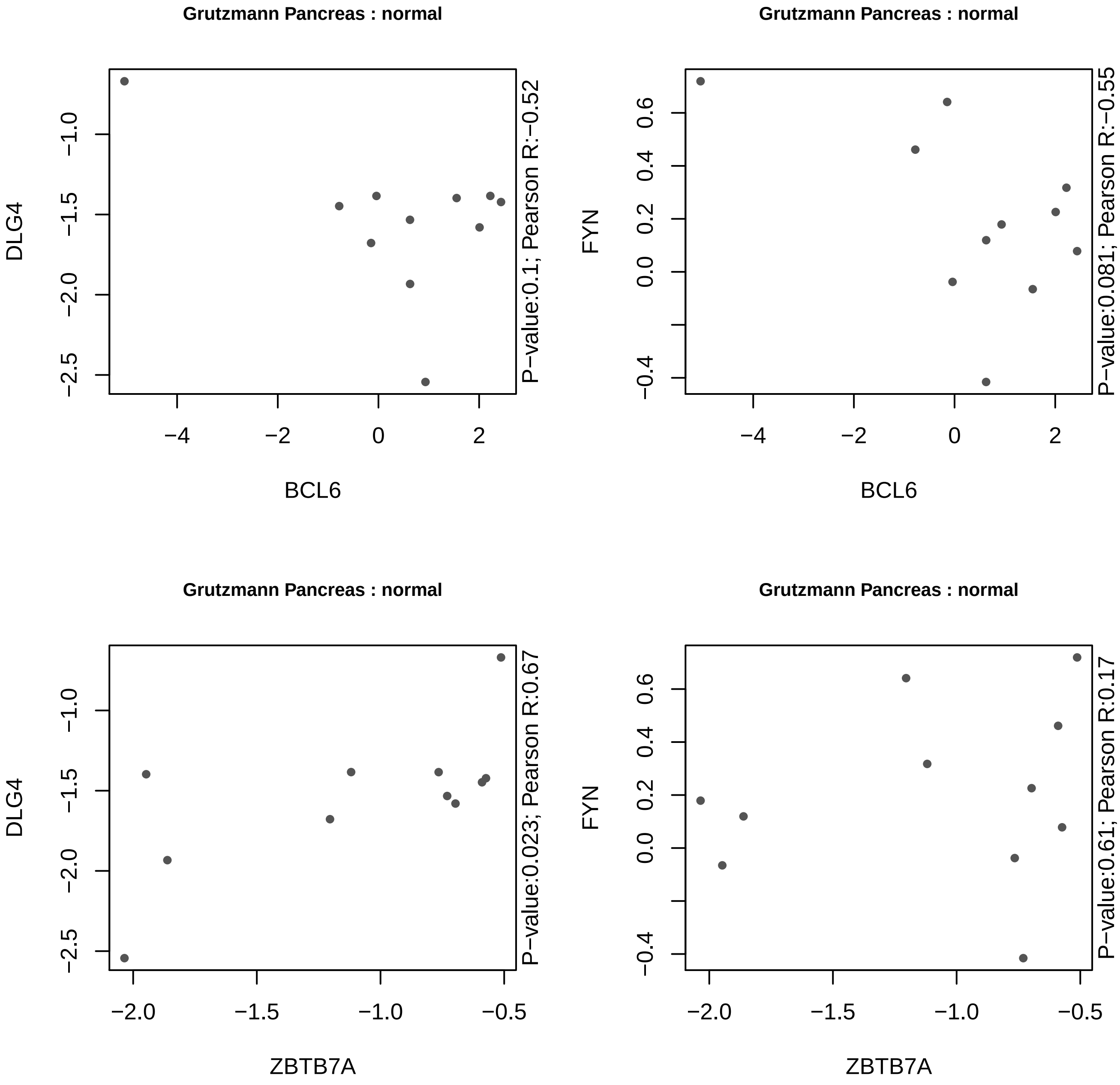
Grutzmann Pancreas : normal
DLG4
BCL6
P−value:0.1; Pearson R:−0.52
Grutzmann Pancreas : normal
FYN
BCL6
P−value:0.081; Pearson R:−0.55
Grutzmann Pancreas : normal
DLG4
ZBTB7A
P−value:0.023; Pearson R:0.67
Grutzmann Pancreas : normal
FYN
ZBTB7A
P−value:0.61; Pearson R:0.17

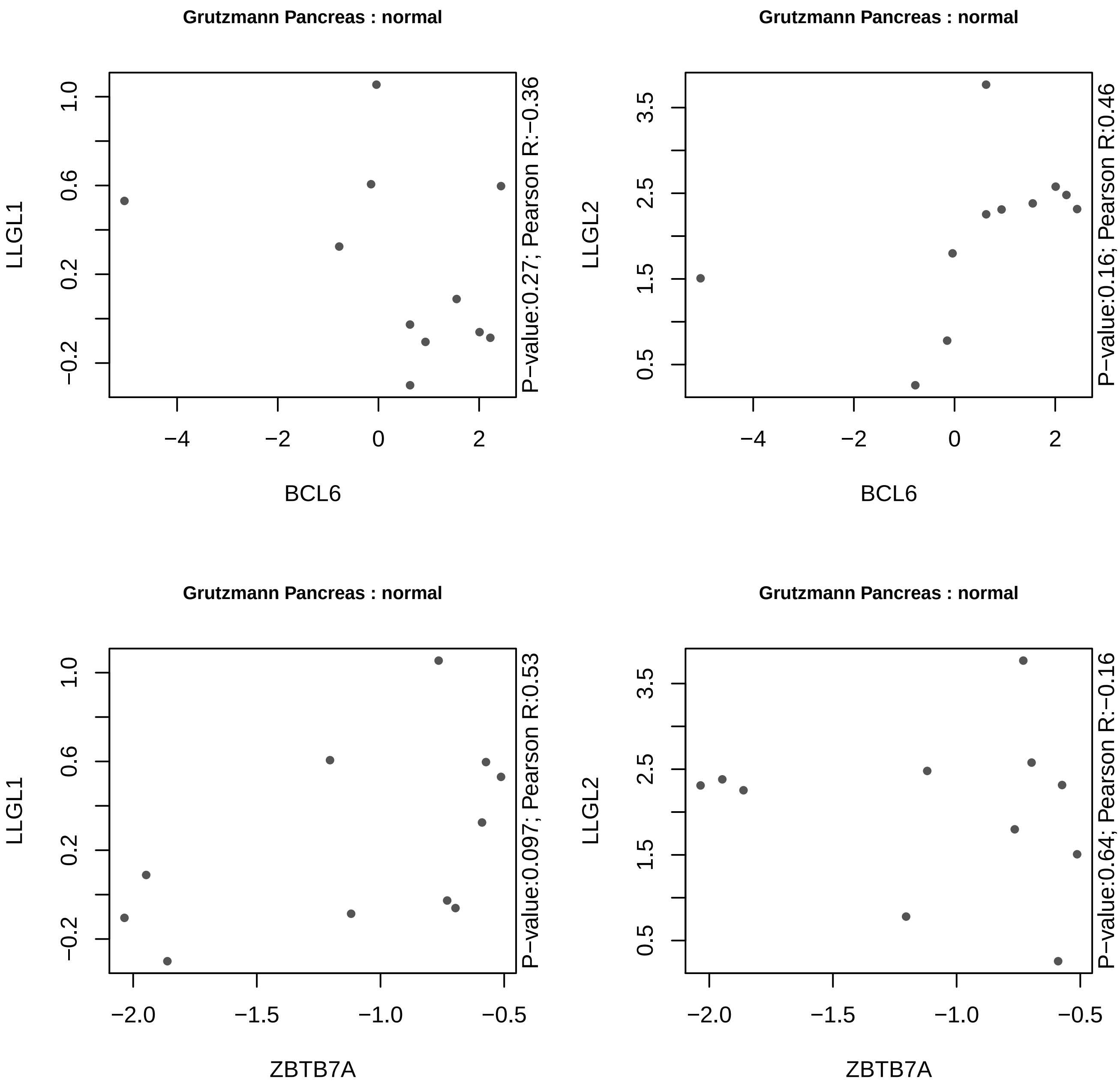
Grutzmann Pancreas : normal
LLGL1
BCL6
P−value:0.27; Pearson R:−0.36
Grutzmann Pancreas : normal
LLGL2
BCL6
P−value:0.16; Pearson R:0.46
Grutzmann Pancreas : normal
LLGL1
ZBTB7A
P−value:0.097; Pearson R:0.53
Grutzmann Pancreas : normal
LLGL2
ZBTB7A
P−value:0.64; Pearson R:−0.16

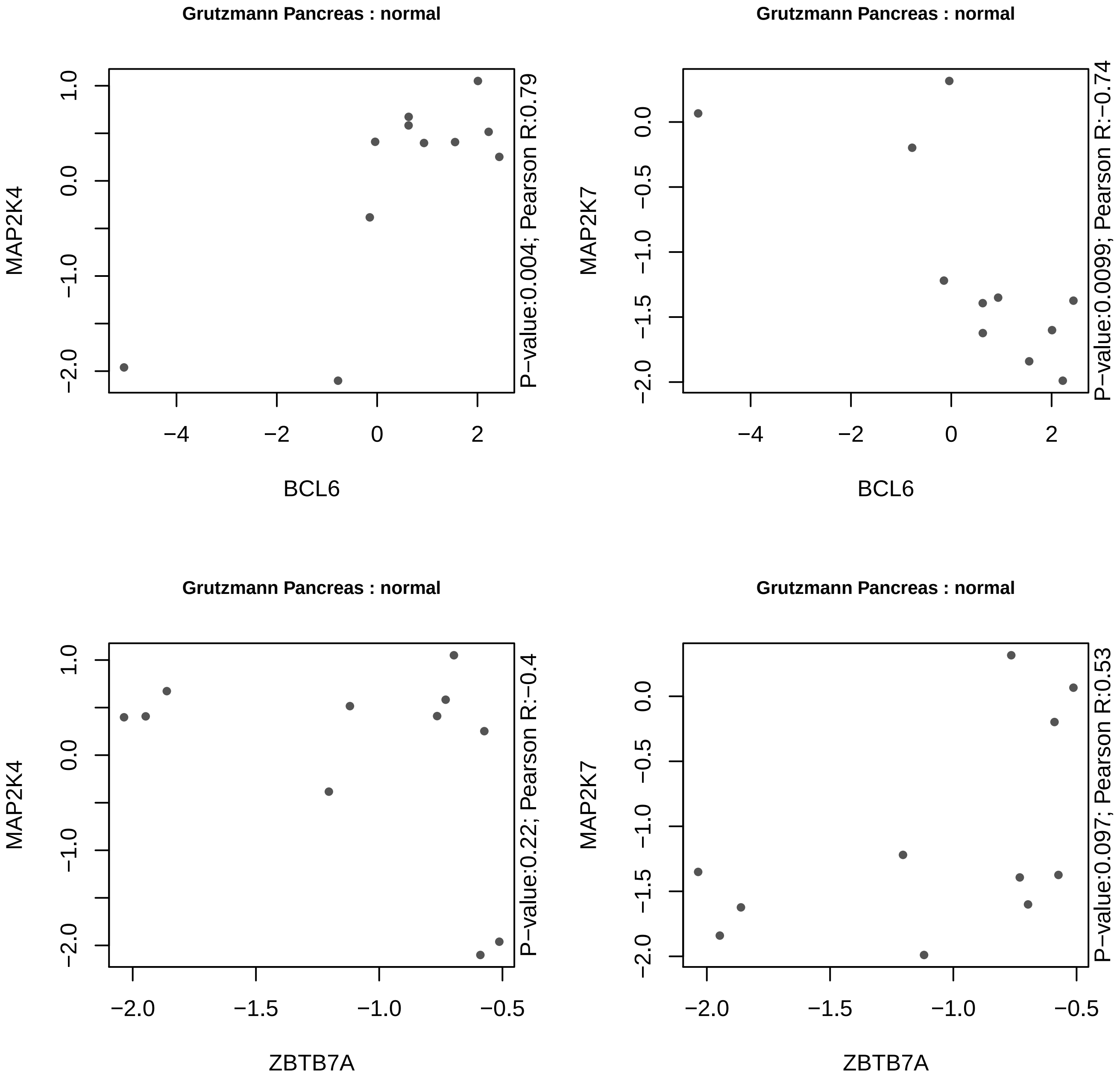
Grutzmann Pancreas : normal
MAP2K4
BCL6
P−value:0.004; Pearson R:0.79
Grutzmann Pancreas : normal
MAP2K7
BCL6
P−value:0.0099; Pearson R:−0.74
Grutzmann Pancreas : normal
MAP2K4
ZBTB7A
P−value:0.22; Pearson R:−0.4
Grutzmann Pancreas : normal
MAP2K7
ZBTB7A
P−value:0.097; Pearson R:0.53

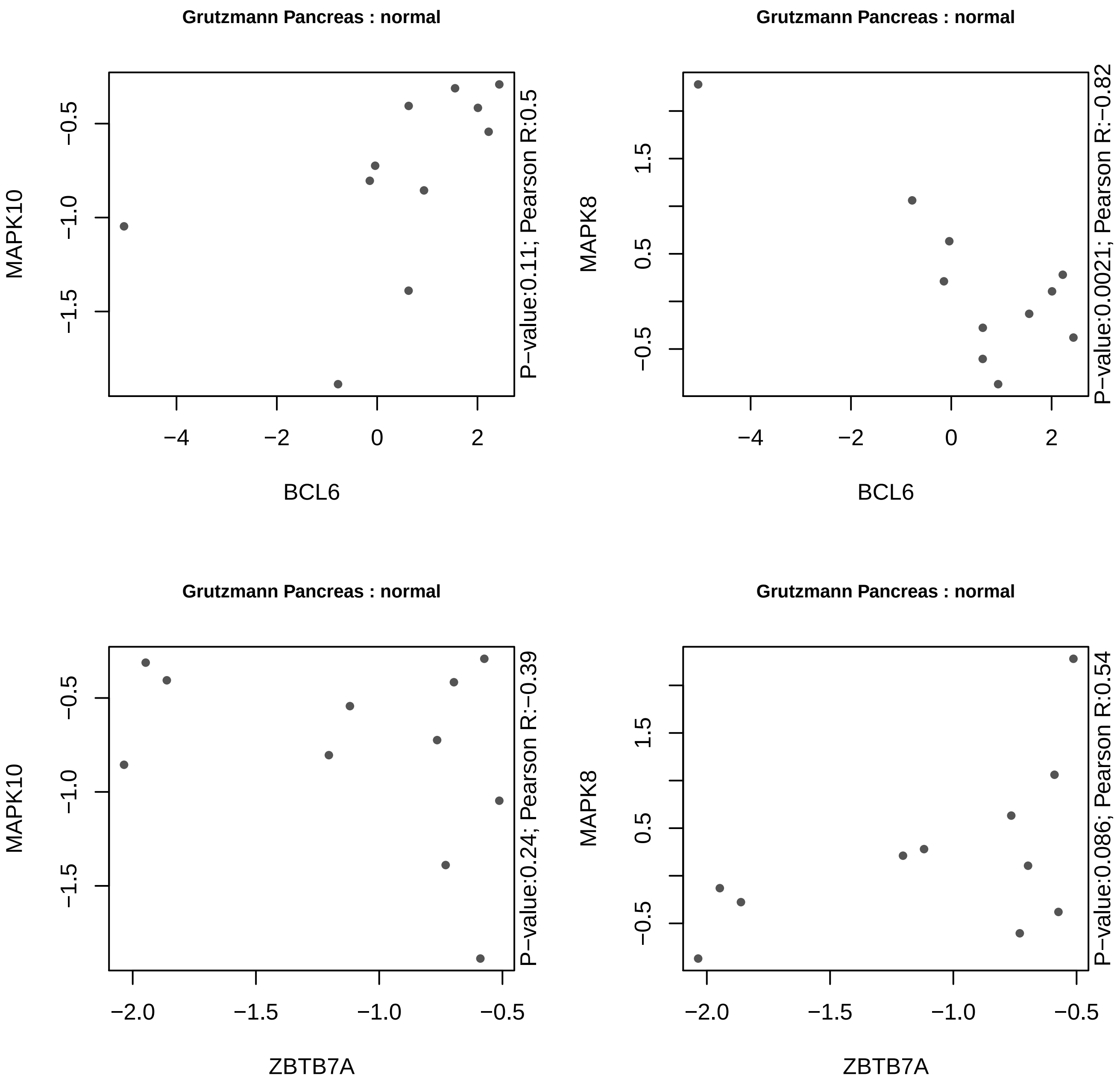
Grutzmann Pancreas : normal
MAPK10
BCL6
−0.5
−1.0
−1.5
−4
−2
0
2
P−value:0.11; Pearson R:0.5
Grutzmann Pancreas : normal
MAPK8
BCL6
1.5
0.5
−0.5
−4
−2
0
2
P−value:0.0021; Pearson R:−0.82
Grutzmann Pancreas : normal
MAPK10
ZBTB7A
−0.5
−1.0
−1.5
−2.0
−1.5
−1.0
−0.5
P−value:0.24; Pearson R:−0.39
Grutzmann Pancreas : normal
MAPK8
ZBTB7A
1.5
0.5
−0.5
−2.0
−1.5
−1.0
−0.5
P−value:0.086; Pearson R:0.54

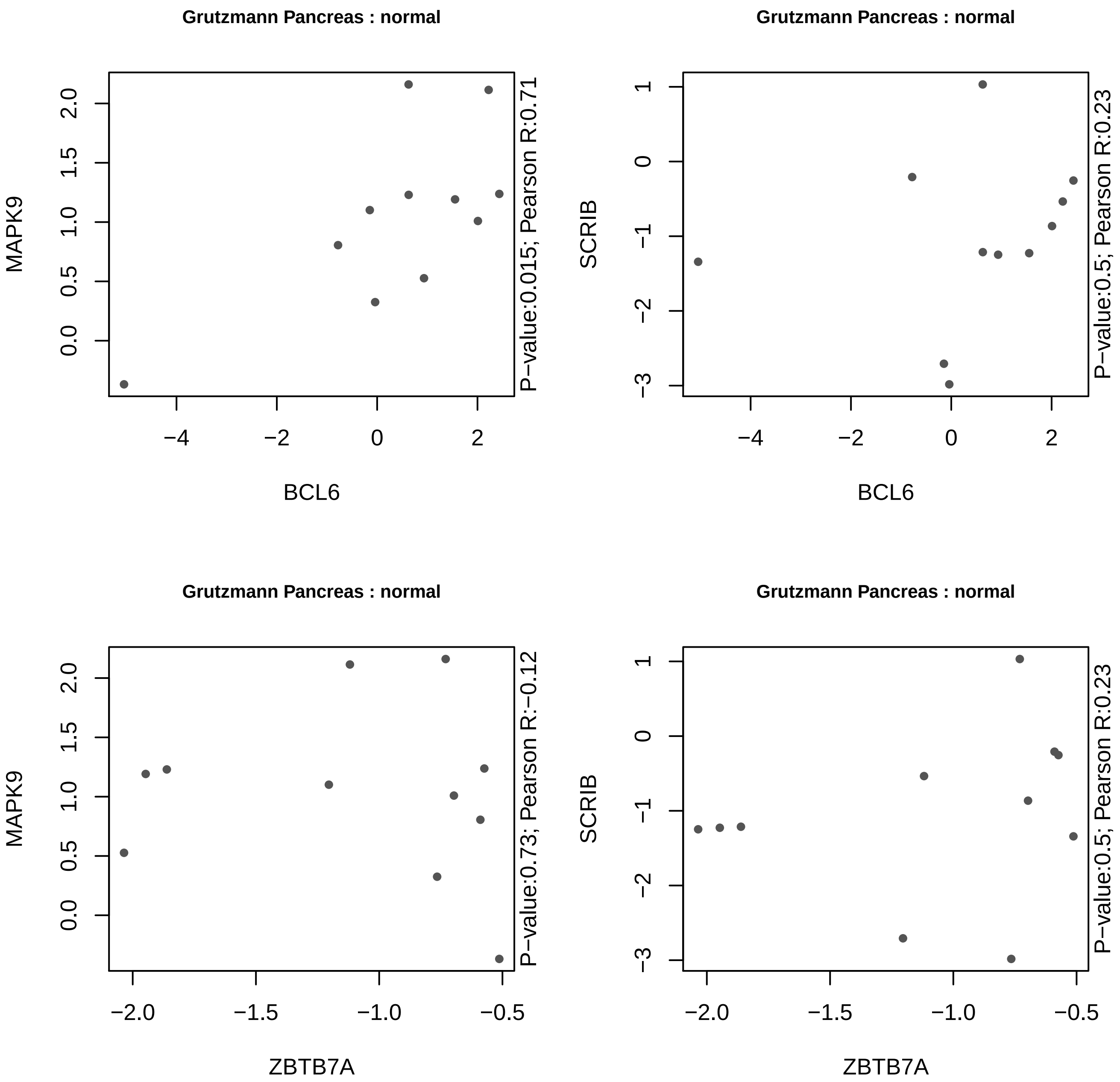
Grutzmann Pancreas : normal
MAPK9
BCL6
P−value:0.015; Pearson R:0.71
Grutzmann Pancreas : normal
SCRIB
BCL6
P−value:0.5; Pearson R:0.23
Grutzmann Pancreas : normal
MAPK9
ZBTB7A
P−value:0.73; Pearson R:−0.12
Grutzmann Pancreas : normal
SCRIB
ZBTB7A
P−value:0.5; Pearson R:0.23

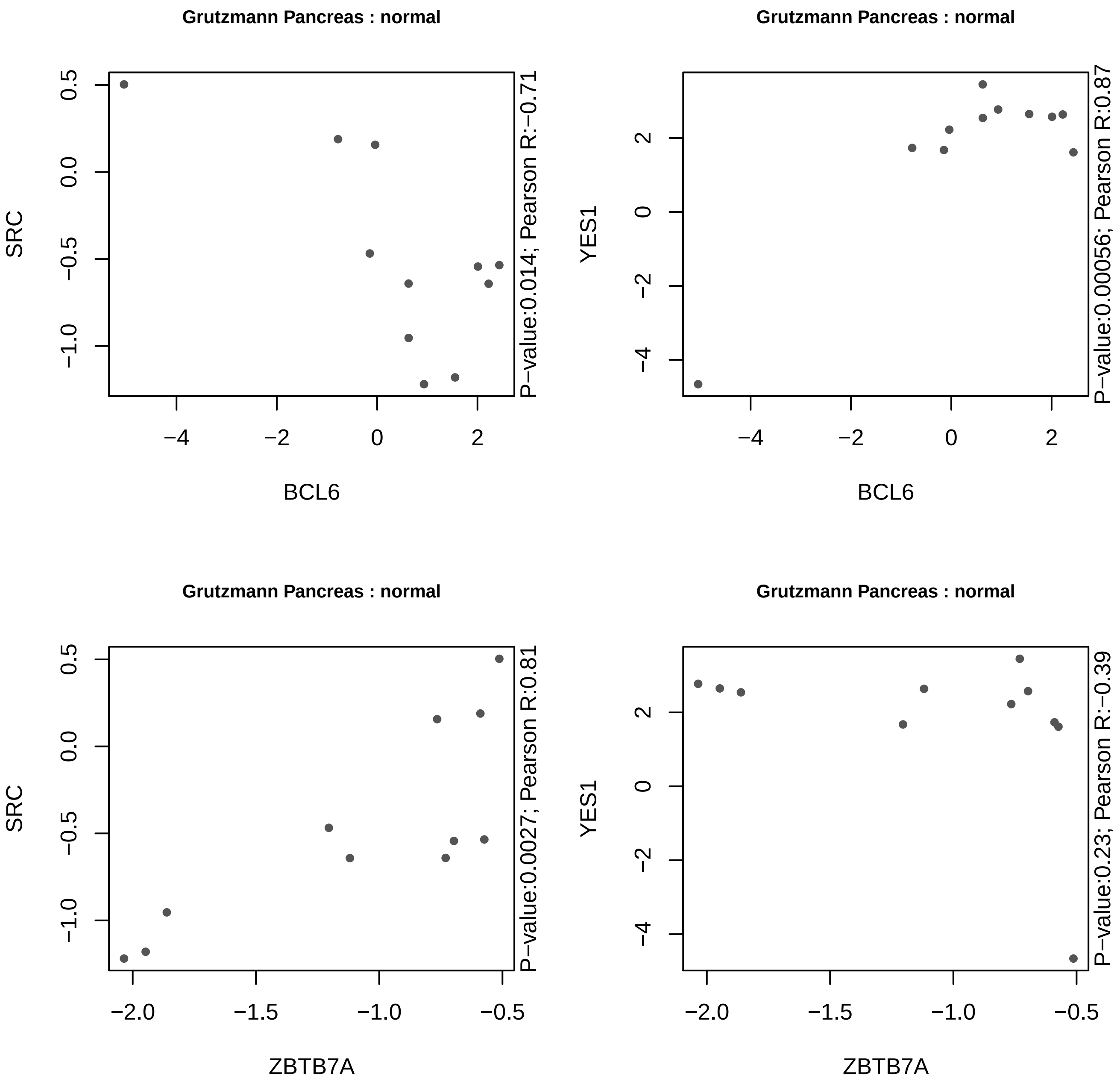
Grutzmann Pancreas : normal
SRC
BCL6
P−value:0.014; Pearson R:−0.71
Grutzmann Pancreas : normal
YES1
BCL6
P−value:0.00056; Pearson R:0.87
Grutzmann Pancreas : normal
SRC
ZBTB7A
P−value:0.0027; Pearson R:0.81
Grutzmann Pancreas : normal
YES1
ZBTB7A
P−value:0.23; Pearson R:−0.39

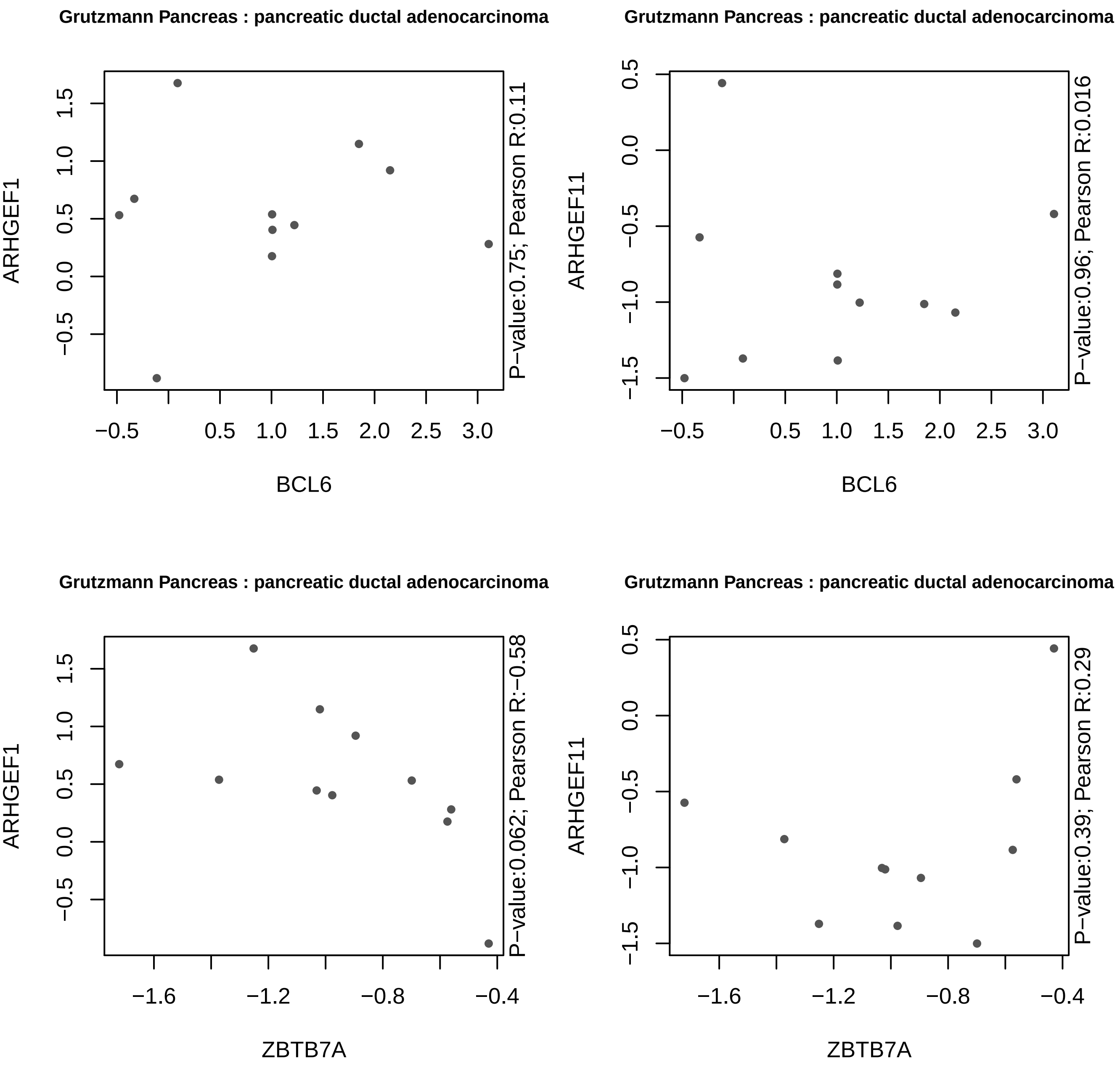
Grutzmann Pancreas : pancreatic ductal adenocarcinoma
ARHGEF1
BCL6
P−value:0.75; Pearson R:0.11
Grutzmann Pancreas : pancreatic ductal adenocarcinoma
ARHGEF11
BCL6
P−value:0.96; Pearson R:0.016
Grutzmann Pancreas : pancreatic ductal adenocarcinoma
ARHGEF1
ZBTB7A
P−value:0.062; Pearson R:−0.58
Grutzmann Pancreas : pancreatic ductal adenocarcinoma
ARHGEF11
ZBTB7A
P−value:0.39; Pearson R:0.29

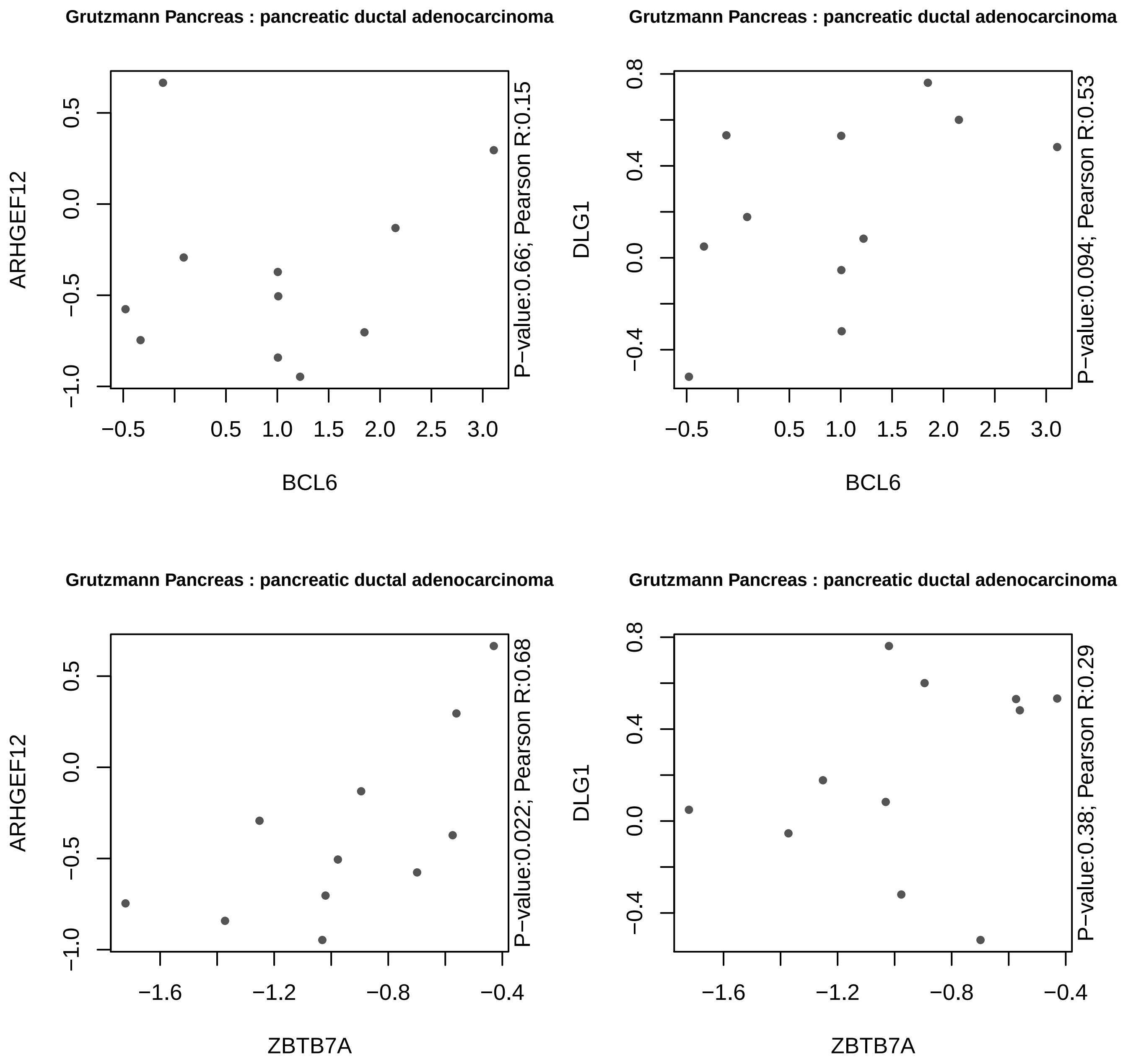
Grutzmann Pancreas : pancreatic ductal adenocarcinoma
ARHGEF12
BCL6
P−value:0.66; Pearson R:0.15
Grutzmann Pancreas : pancreatic ductal adenocarcinoma
DLG1
BCL6
P−value:0.094; Pearson R:0.53
Grutzmann Pancreas : pancreatic ductal adenocarcinoma
ARHGEF12
ZBTB7A
P−value:0.022; Pearson R:0.68
Grutzmann Pancreas : pancreatic ductal adenocarcinoma
DLG1
ZBTB7A
P−value:0.38; Pearson R:0.29

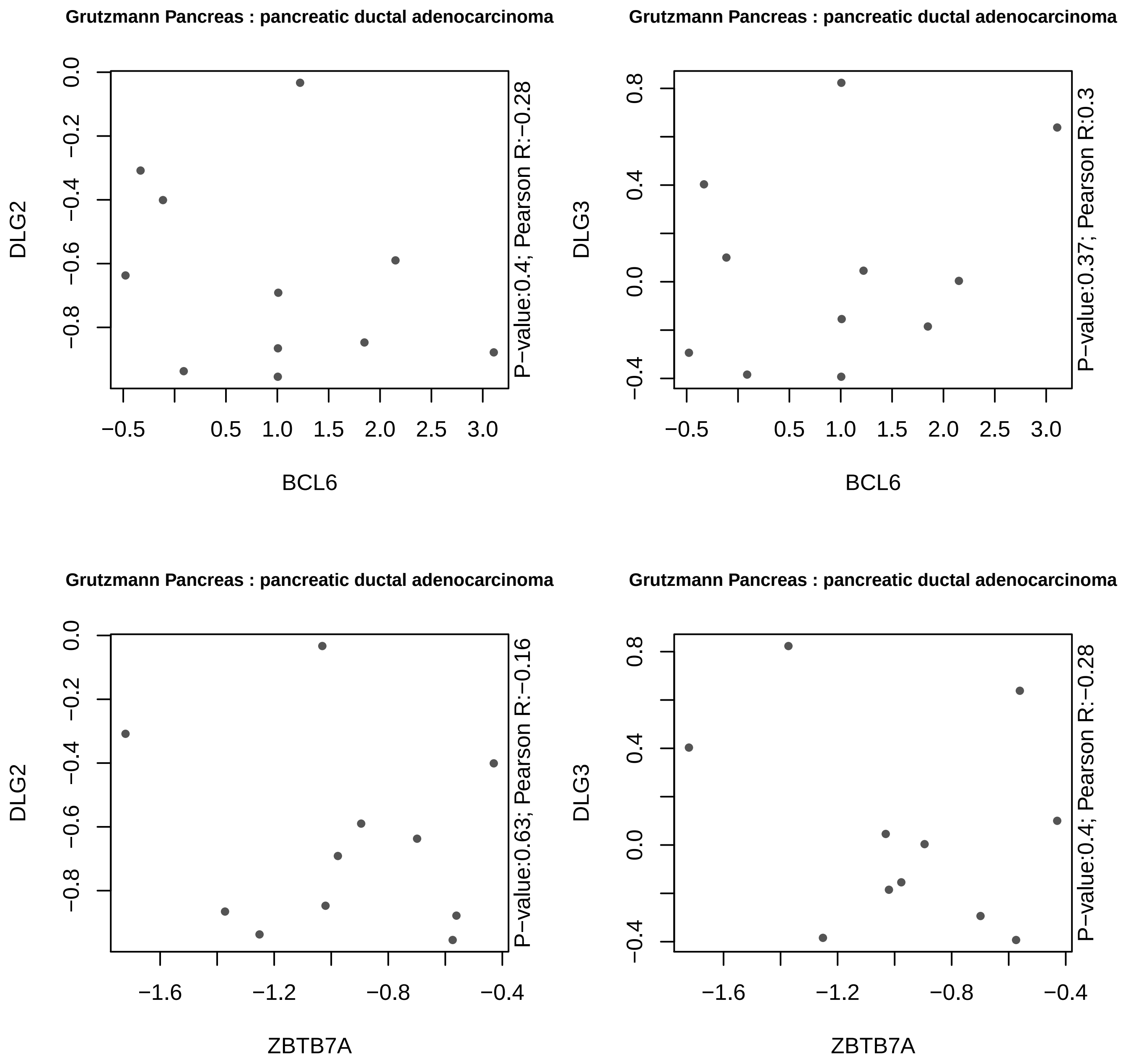
Grutzmann Pancreas : pancreatic ductal adenocarcinoma
DLG2
BCL6
P−value:0.4; Pearson R:−0.28
Grutzmann Pancreas : pancreatic ductal adenocarcinoma
DLG3
BCL6
P−value:0.37; Pearson R:0.3
Grutzmann Pancreas : pancreatic ductal adenocarcinoma
DLG2
ZBTB7A
P−value:0.63; Pearson R:−0.16
Grutzmann Pancreas : pancreatic ductal adenocarcinoma
DLG3
ZBTB7A
P−value:0.4; Pearson R:−0.28

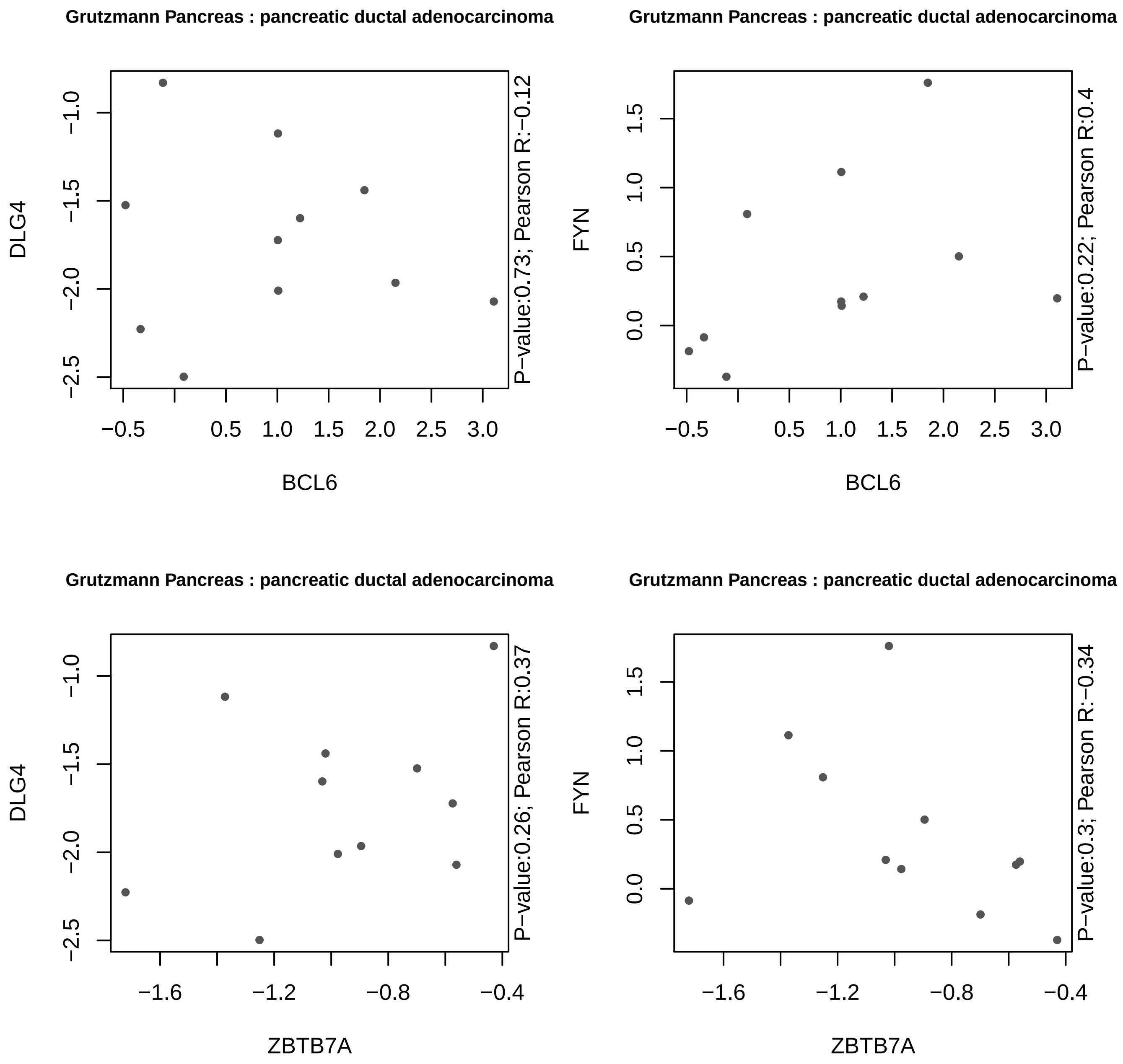
Grutzmann Pancreas : pancreatic ductal adenocarcinoma
DLG4
BCL6
P−value:0.73; Pearson R:−0.12
Grutzmann Pancreas : pancreatic ductal adenocarcinoma
FYN
BCL6
P−value:0.22; Pearson R:0.4
Grutzmann Pancreas : pancreatic ductal adenocarcinoma
DLG4
ZBTB7A
P−value:0.26; Pearson R:0.37
Grutzmann Pancreas : pancreatic ductal adenocarcinoma
FYN
ZBTB7A
P−value:0.3; Pearson R:−0.34

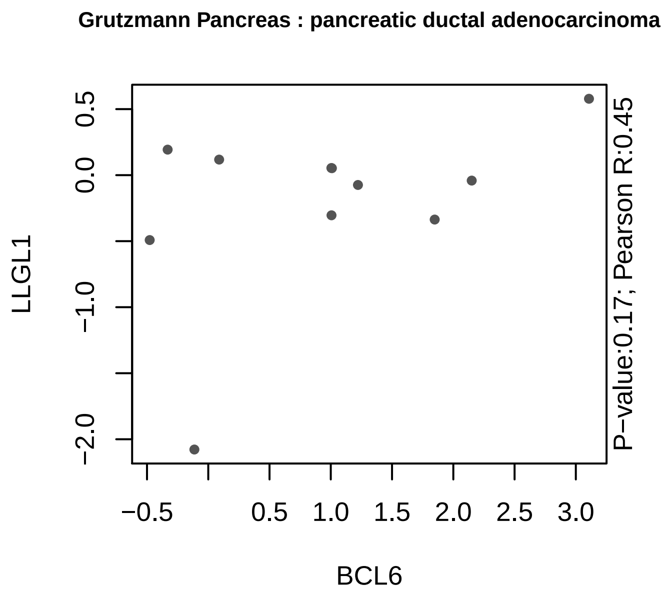
Grutzmann Pancreas : pancreatic ductal adenocarcinoma
LLGL1
BCL6
P−value:0.17; Pearson R:0.45

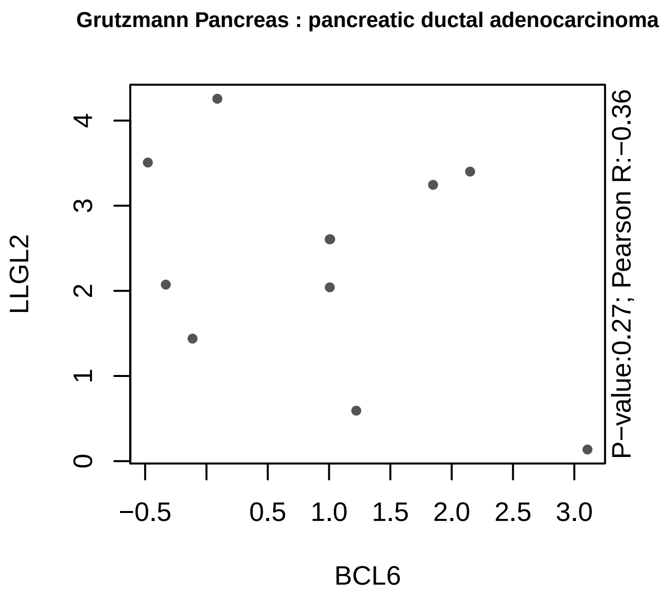
Grutzmann Pancreas : pancreatic ductal adenocarcinoma
LLGL2
BCL6
P−value:0.27; Pearson R:−0.36

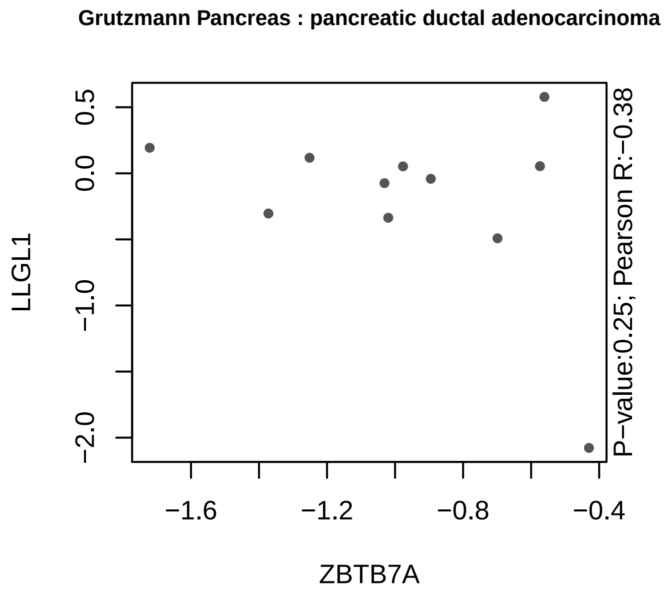
Grutzmann Pancreas : pancreatic ductal adenocarcinoma
LLGL1
ZBTB7A
P−value:0.25; Pearson R:−0.38

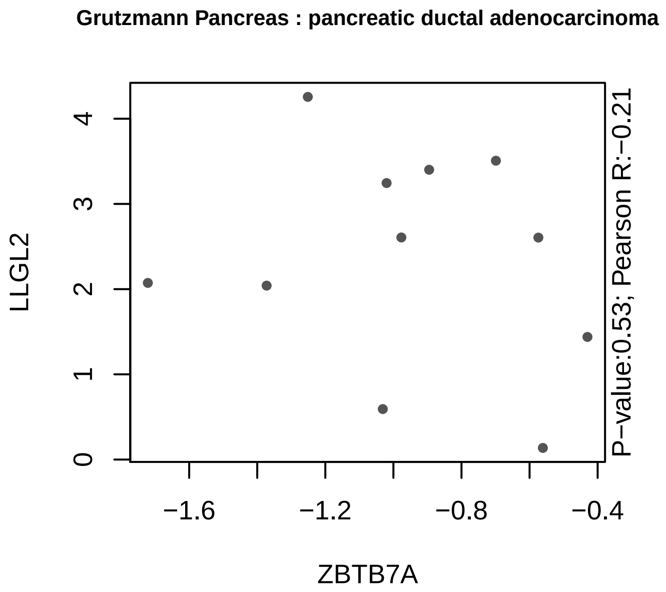
Grutzmann Pancreas : pancreatic ductal adenocarcinoma
LLGL2
ZBTB7A
P−value:0.53; Pearson R:−0.21

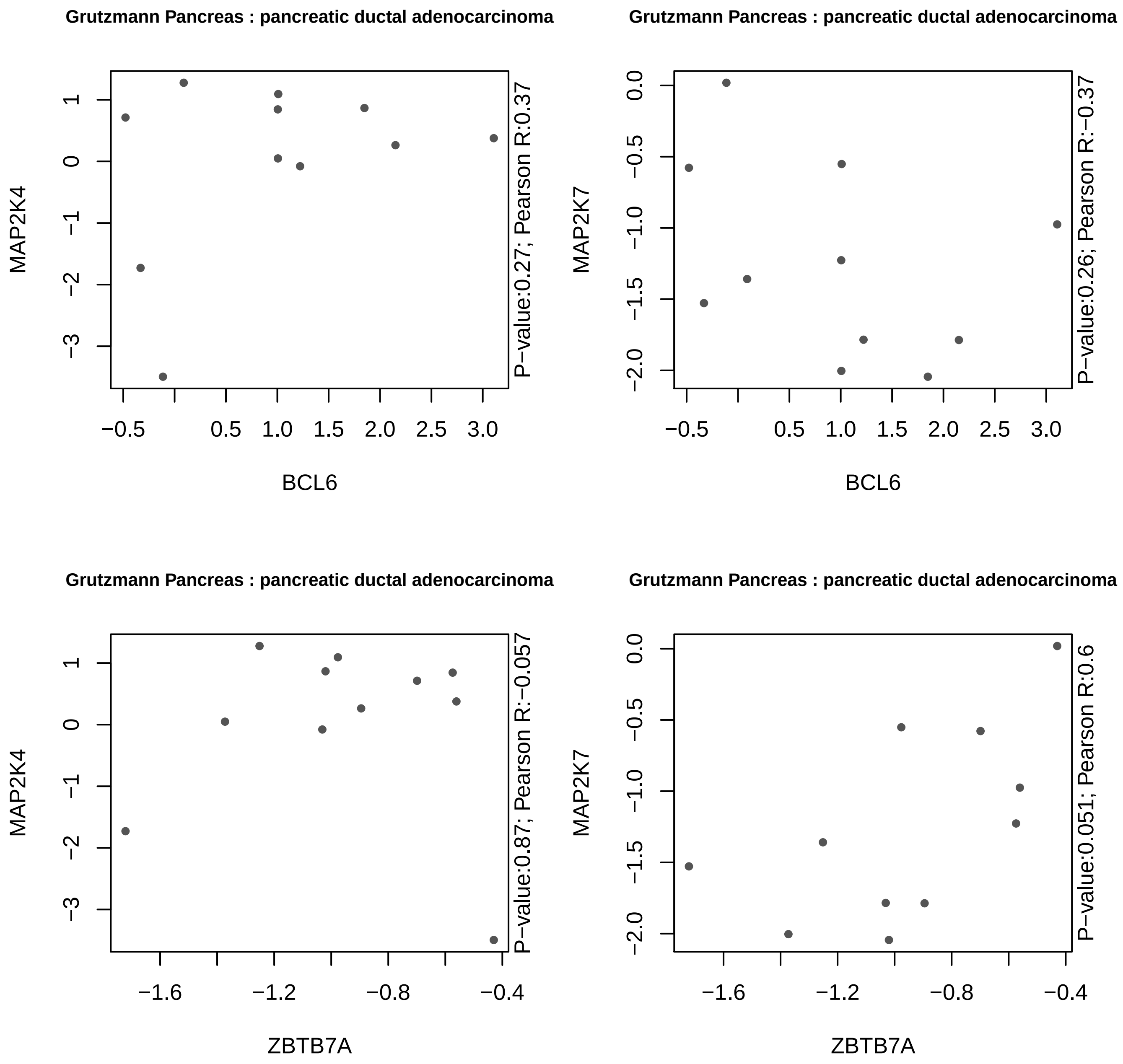
Grutzmann Pancreas : pancreatic ductal adenocarcinoma
MAP2K4
BCL6
P−value:0.27; Pearson R:0.37
Grutzmann Pancreas : pancreatic ductal adenocarcinoma
MAP2K7
BCL6
P−value:0.26; Pearson R:−0.37
Grutzmann Pancreas : pancreatic ductal adenocarcinoma
MAP2K4
ZBTB7A
P−value:0.87; Pearson R:−0.057
Grutzmann Pancreas : pancreatic ductal adenocarcinoma
MAP2K7
ZBTB7A
P−value:0.051; Pearson R:0.6

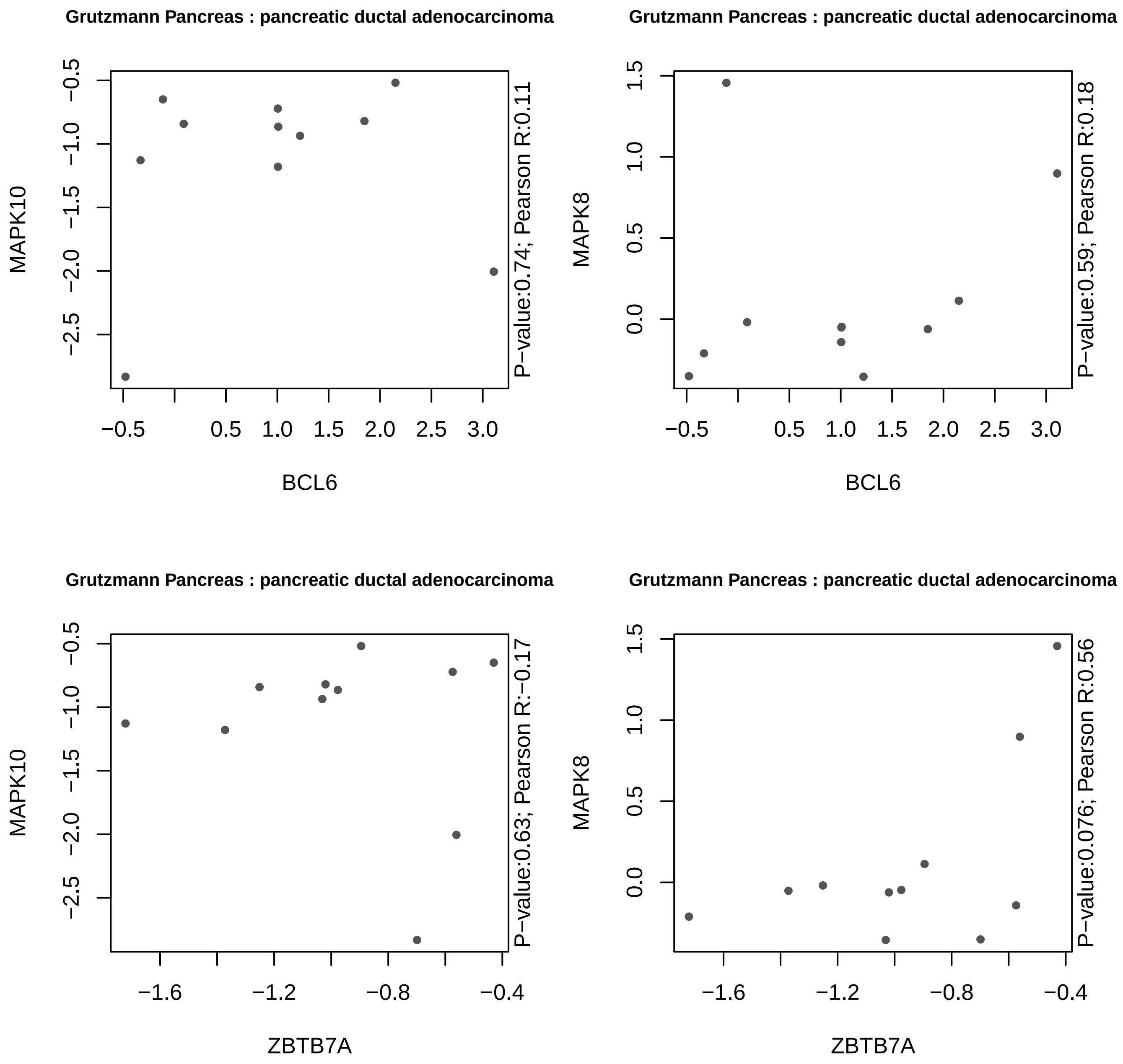
Grutzmann Pancreas : pancreatic ductal adenocarcinoma
MAPK10
BCL6
P−value:0.74; Pearson R:0.11
Grutzmann Pancreas : pancreatic ductal adenocarcinoma
MAPK8
BCL6
P−value:0.59; Pearson R:0.18
Grutzmann Pancreas : pancreatic ductal adenocarcinoma
MAPK10
ZBTB7A
P−value:0.63; Pearson R:−0.17
Grutzmann Pancreas : pancreatic ductal adenocarcinoma
MAPK8
ZBTB7A
P−value:0.076; Pearson R:0.56

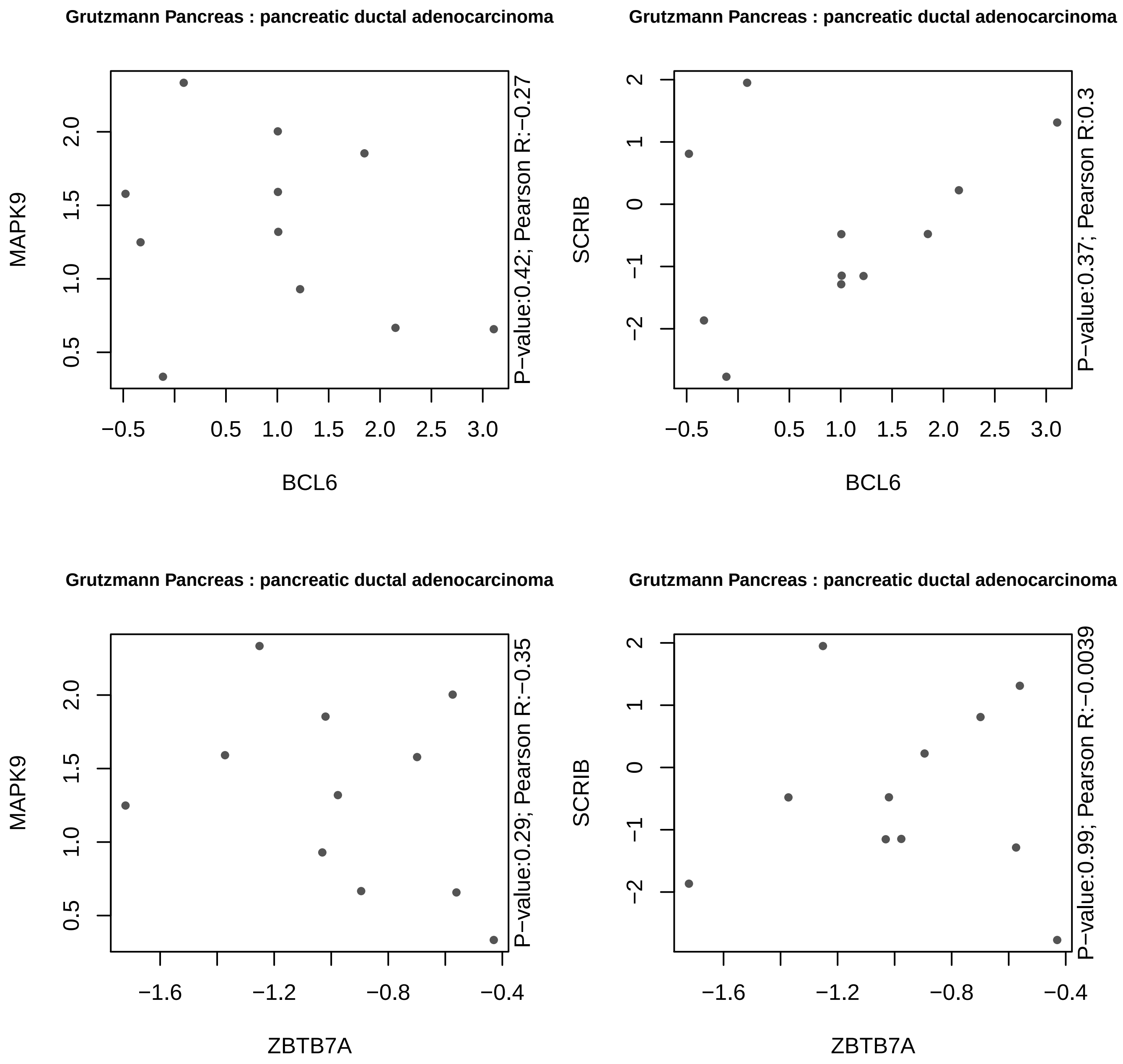
Grutzmann Pancreas : pancreatic ductal adenocarcinoma
MAPK9
BCL6
P−value:0.42; Pearson R:−0.27
Grutzmann Pancreas : pancreatic ductal adenocarcinoma
SCRIB
BCL6
P−value:0.37; Pearson R:0.3
Grutzmann Pancreas : pancreatic ductal adenocarcinoma
MAPK9
ZBTB7A
P−value:0.29; Pearson R:−0.35
Grutzmann Pancreas : pancreatic ductal adenocarcinoma
SCRIB
ZBTB7A
P−value:0.99; Pearson R:−0.0039

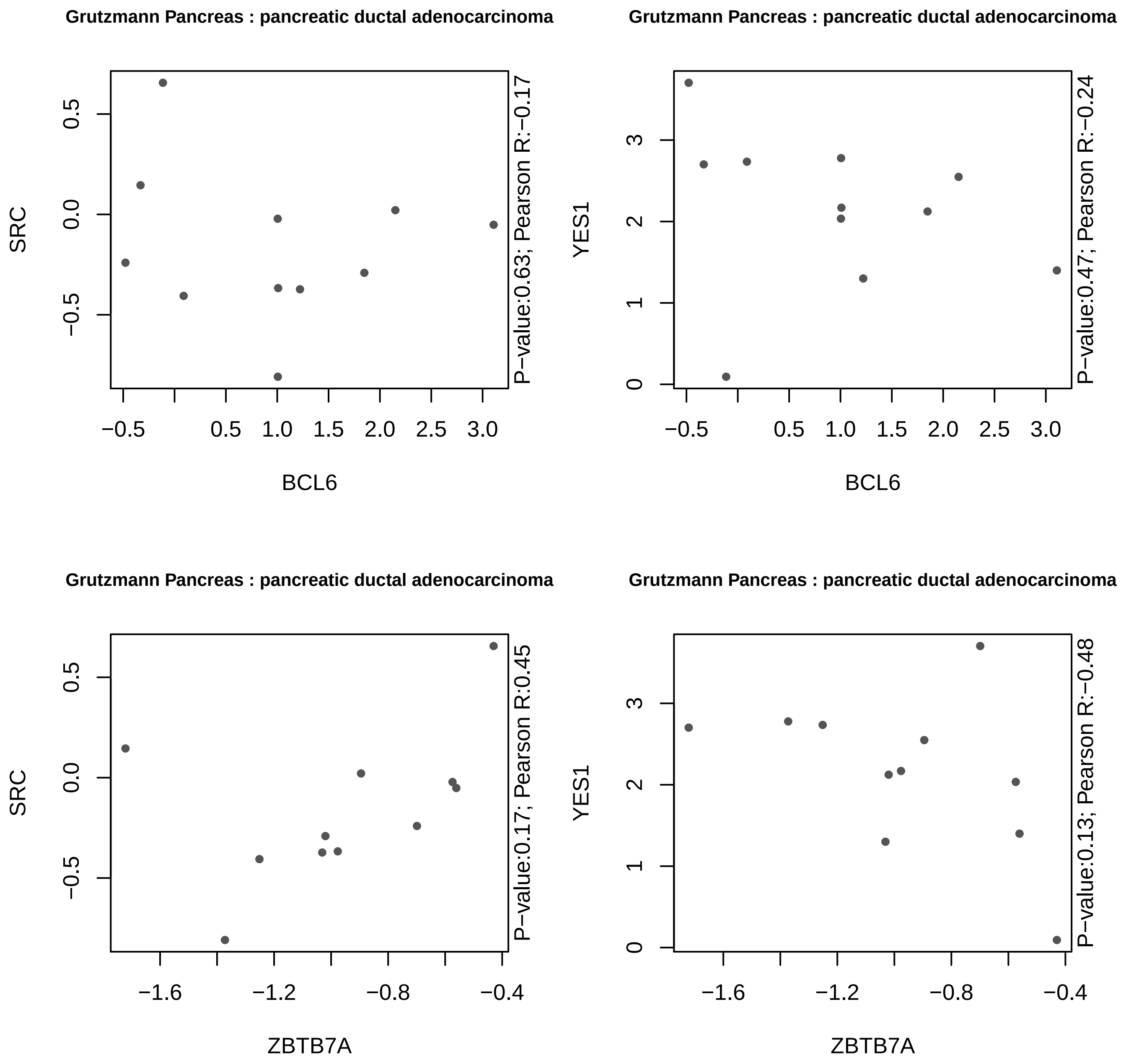
Grutzmann Pancreas : pancreatic ductal adenocarcinoma
SRC
BCL6
P−value:0.63; Pearson R:−0.17
Grutzmann Pancreas : pancreatic ductal adenocarcinoma
YES1
BCL6
P−value:0.47; Pearson R:−0.24
Grutzmann Pancreas : pancreatic ductal adenocarcinoma
SRC
ZBTB7A
P−value:0.17; Pearson R:0.45
Grutzmann Pancreas : pancreatic ductal adenocarcinoma
YES1
ZBTB7A
P−value:0.13; Pearson R:−0.48

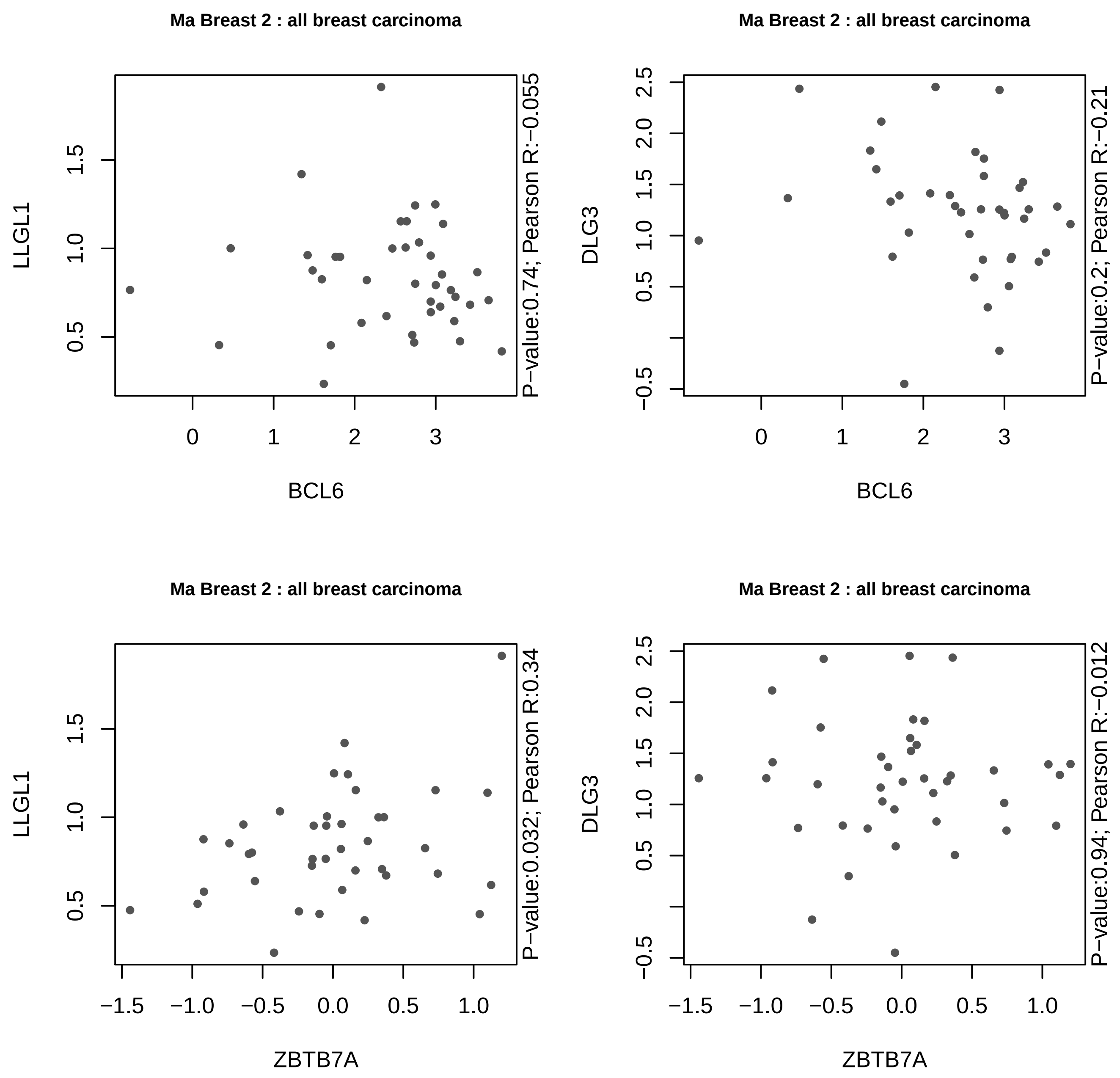
Ma Breast 2 : all breast carcinoma
LLGL1
BCL6
P−value:0.74; Pearson R:−0.055
Ma Breast 2 : all breast carcinoma
DLG3
BCL6
P−value:0.2; Pearson R:−0.21
Ma Breast 2 : all breast carcinoma
LLGL1
ZBTB7A
P−value:0.032; Pearson R:0.34
Ma Breast 2 : all breast carcinoma
DLG3
ZBTB7A
P−value:0.94; Pearson R:−0.012

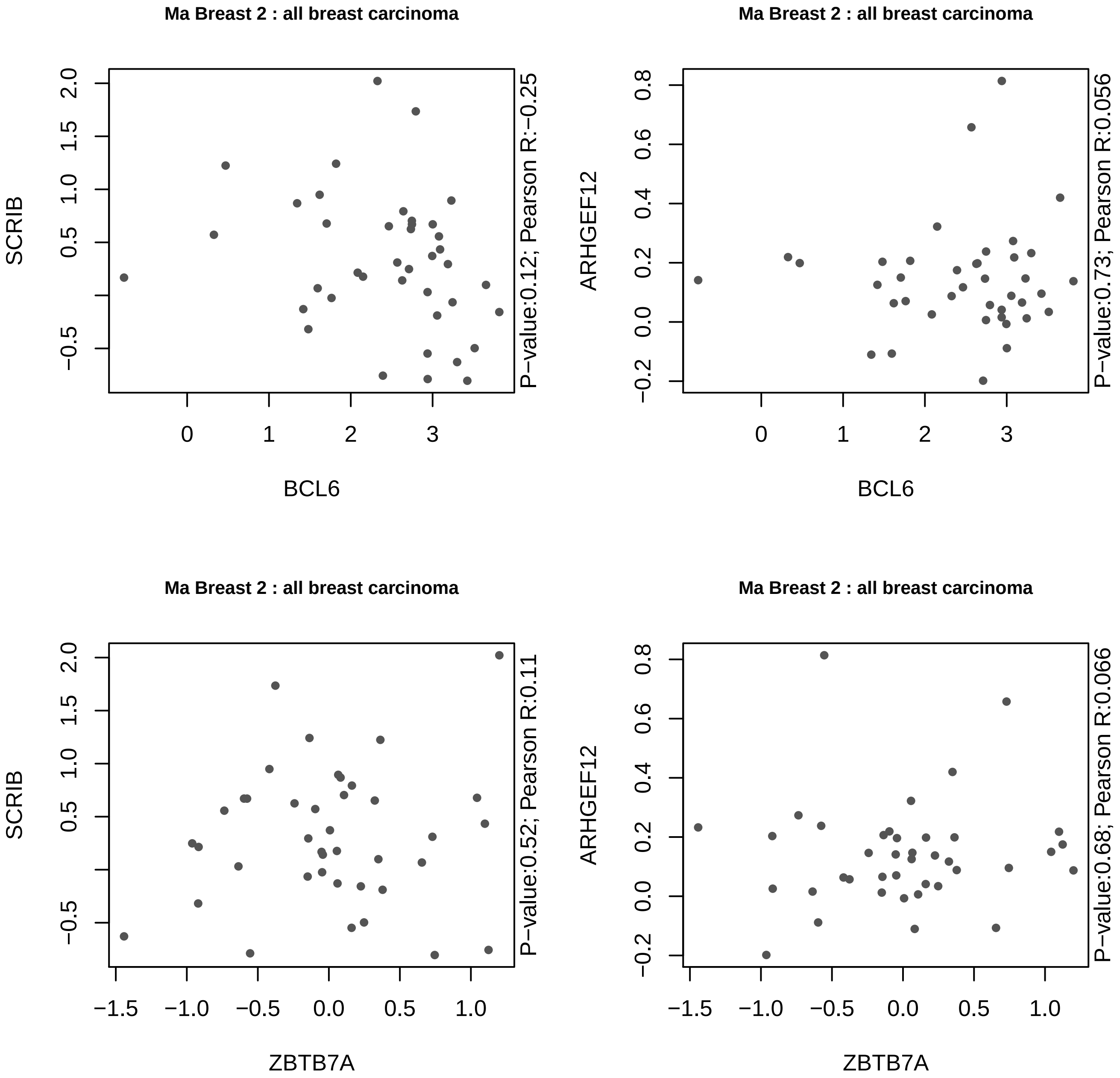
Ma Breast 2 : all breast carcinoma
SCRIB
BCL6
P−value:0.12; Pearson R:−0.25
Ma Breast 2 : all breast carcinoma
ARHGEF12
BCL6
P−value:0.73; Pearson R:0.056
Ma Breast 2 : all breast carcinoma
SCRIB
ZBTB7A
P−value:0.52; Pearson R:0.11
Ma Breast 2 : all breast carcinoma
ARHGEF12
ZBTB7A
P−value:0.68; Pearson R:0.066

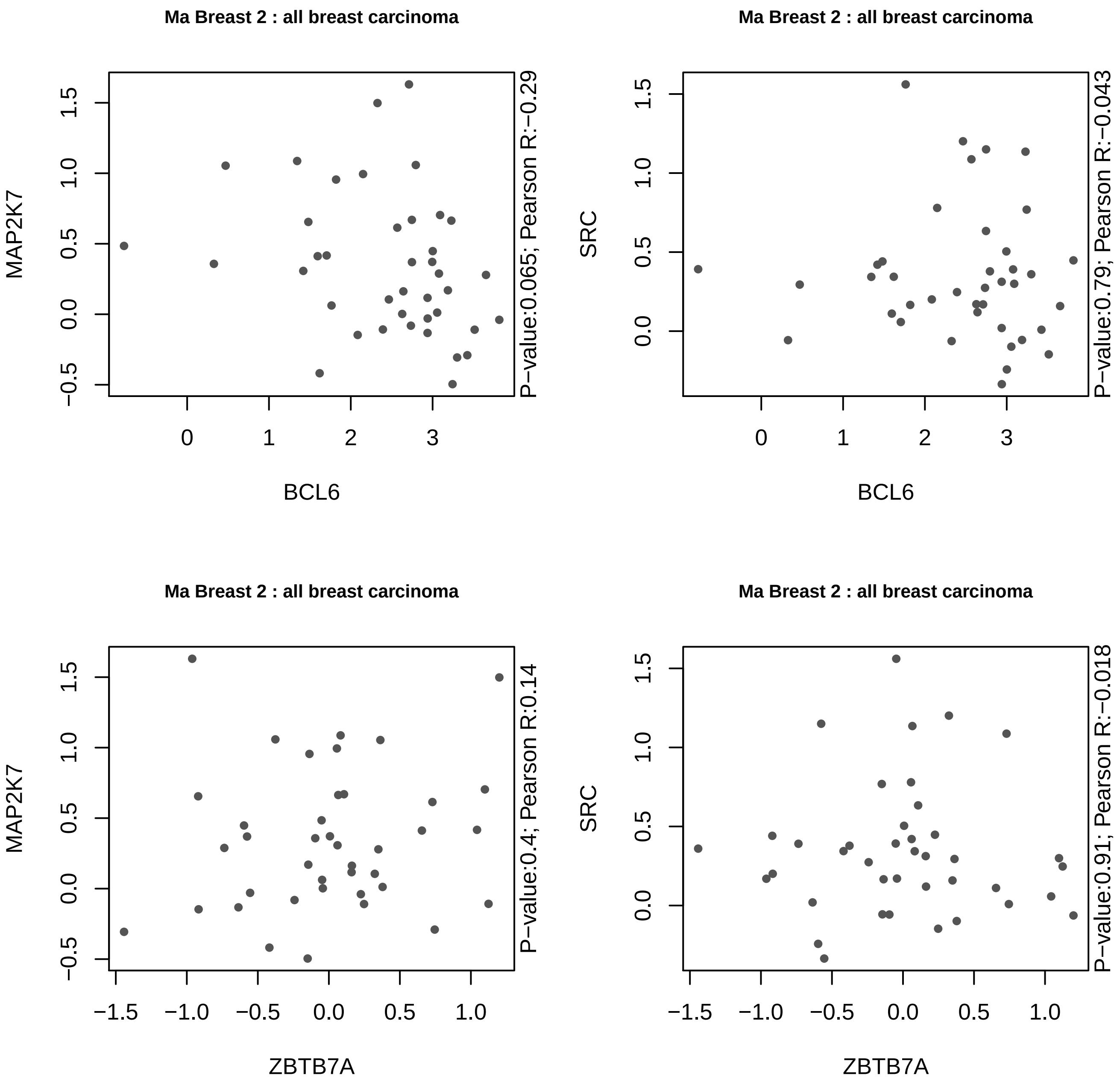
Ma Breast 2 : all breast carcinoma
MAP2K7
BCL6
P−value:0.065; Pearson R:−0.29
Ma Breast 2 : all breast carcinoma
SRC
BCL6
P−value:0.79; Pearson R:−0.043
Ma Breast 2 : all breast carcinoma
MAP2K7
ZBTB7A
P−value:0.4; Pearson R:0.14
Ma Breast 2 : all breast carcinoma
SRC
ZBTB7A
P−value:0.91; Pearson R:−0.018

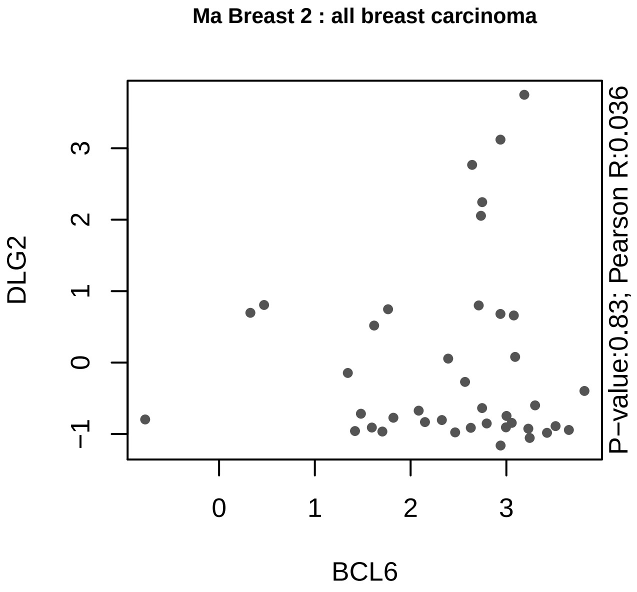
Ma Breast 2 : all breast carcinoma
DLG2
BCL6
P−value:0.83; Pearson R:0.036

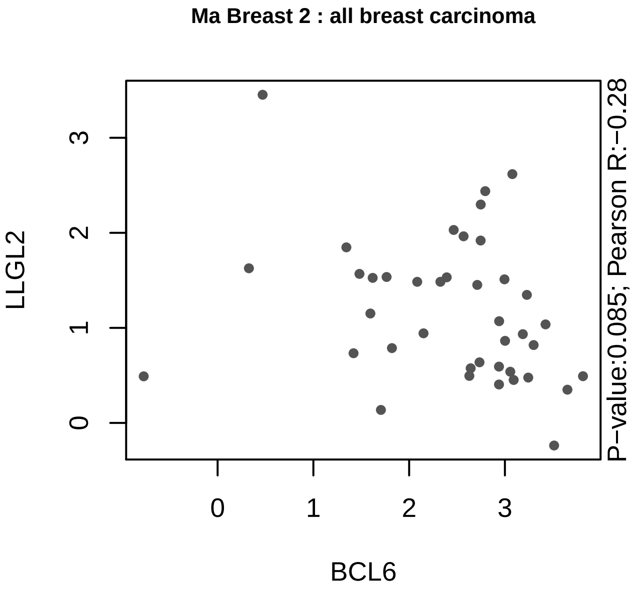
Ma Breast 2 : all breast carcinoma
LLGL2
BCL6
P−value:0.085; Pearson R:−0.28

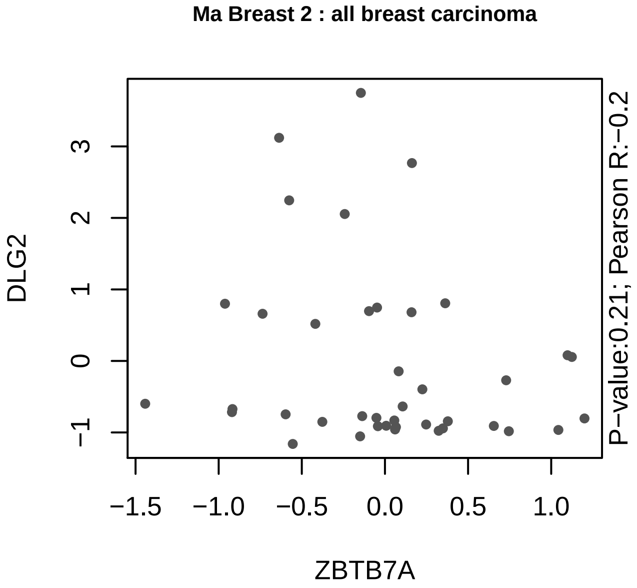
Ma Breast 2 : all breast carcinoma
DLG2
ZBTB7A
P−value:0.21; Pearson R:−0.2

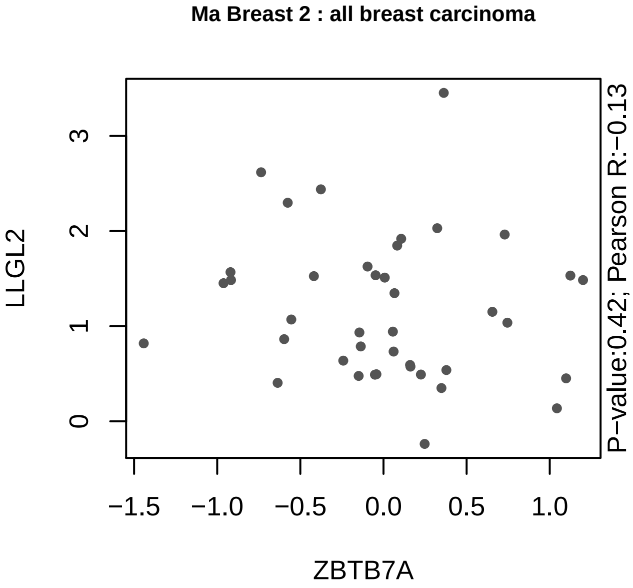
Ma Breast 2 : all breast carcinoma
LLGL2
ZBTB7A
P−value:0.42; Pearson R:−0.13

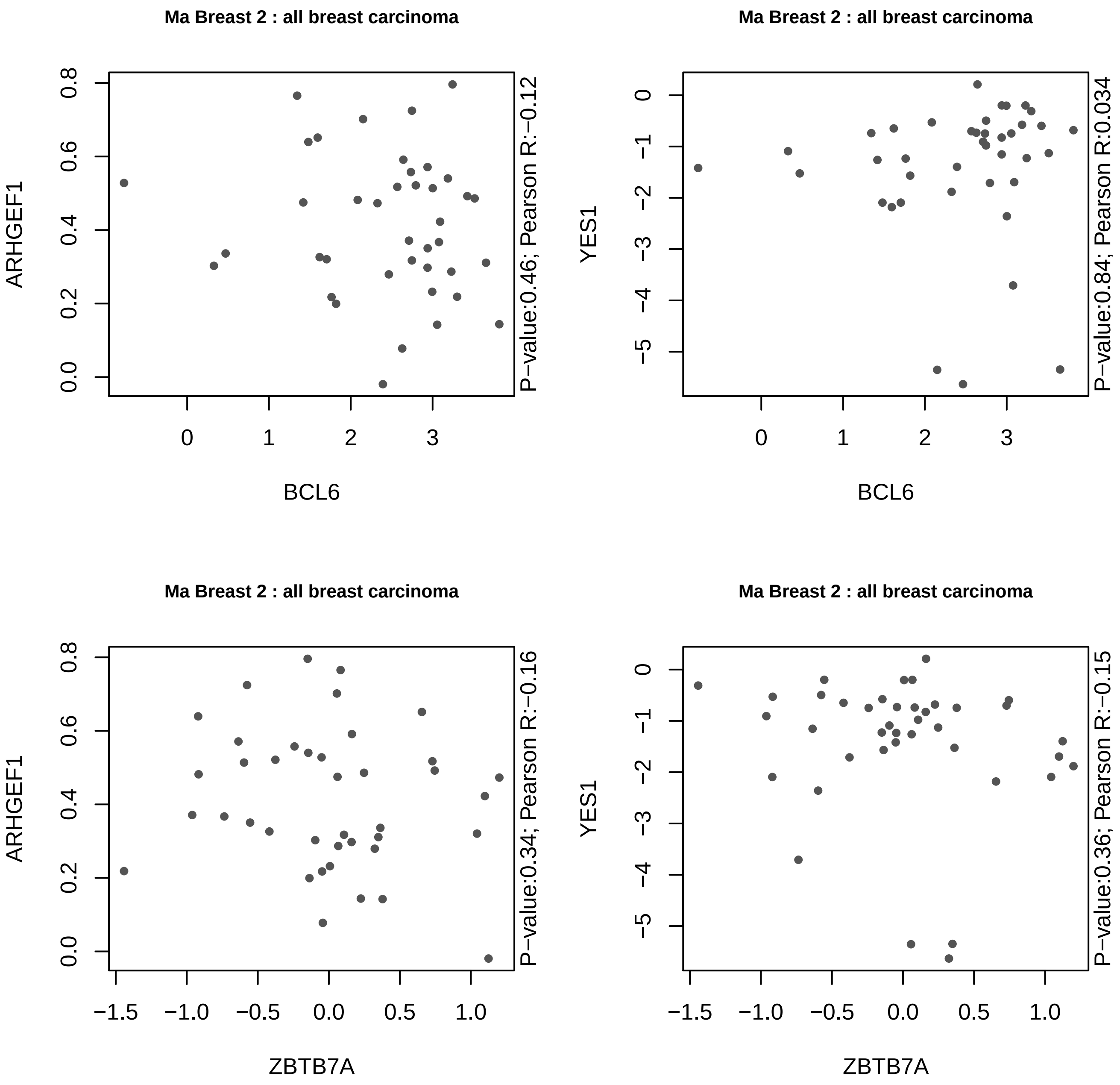
Ma Breast 2 : all breast carcinoma
ARHGEF1
BCL6
P−value:0.46; Pearson R:−0.12
0.0
0.2
0.4
0.6
0.8
0
1
2
3
Ma Breast 2 : all breast carcinoma
YES1
BCL6
P−value:0.84; Pearson R:0.034
0
−1
−2
−3
−4
−5
0
1
2
3
Ma Breast 2 : all breast carcinoma
ARHGEF1
ZBTB7A
P−value:0.34; Pearson R:−0.16
0.0
0.2
0.4
0.6
0.8
−1.5
−1.0
−0.5
0.0
0.5
1.0
Ma Breast 2 : all breast carcinoma
YES1
ZBTB7A
P−value:0.36; Pearson R:−0.15
0
−1
−2
−3
−4
−5
−1.5
−1.0
−0.5
0.0
0.5
1.0

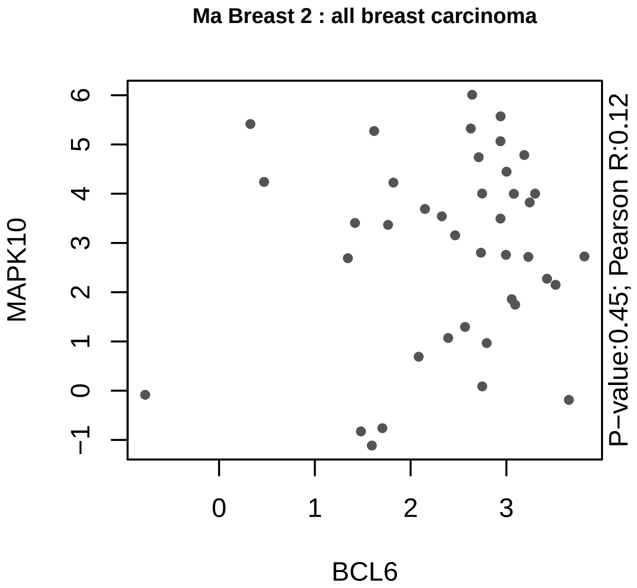
Ma Breast 2 : all breast carcinoma
MAPK10
−1 0 1 2 3 4 5 6
0 1 2 3
BCL6
P−value:0.45; Pearson R:0.12

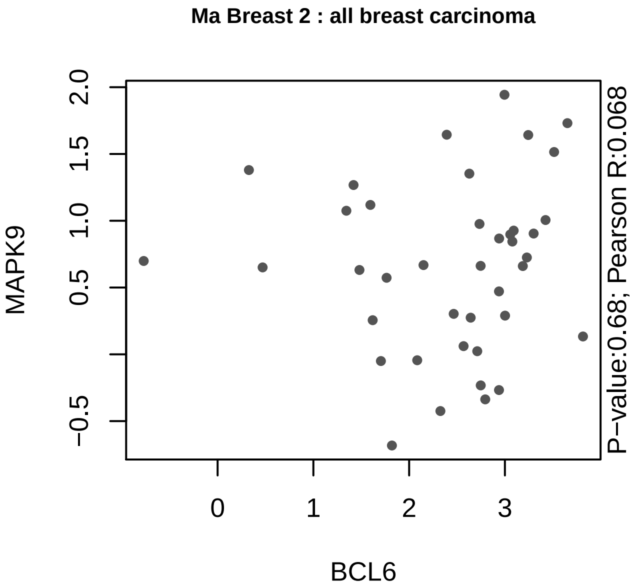
Ma Breast 2 : all breast carcinoma
MAPK9
−0.5 0.5 1.0 1.5 2.0
0 1 2 3
BCL6
P−value:0.68; Pearson R:0.068

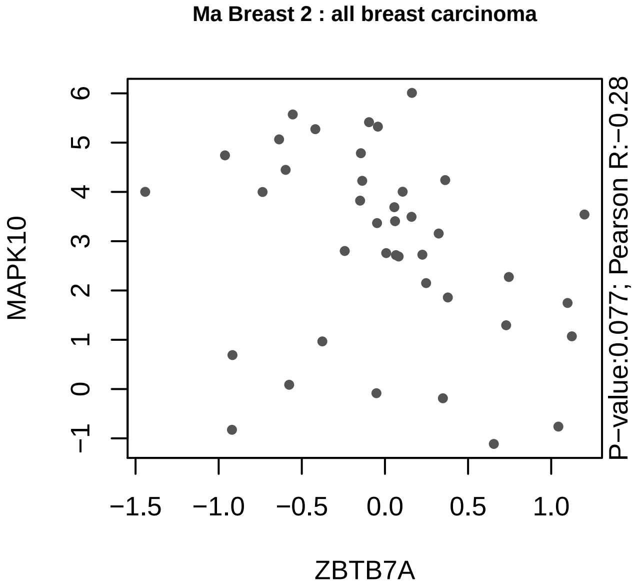
Ma Breast 2 : all breast carcinoma
MAPK10
−1 0 1 2 3 4 5 6
−1.5 −1.0 −0.5 0.0 0.5 1.0
ZBTB7A
P−value:0.077; Pearson R:−0.28

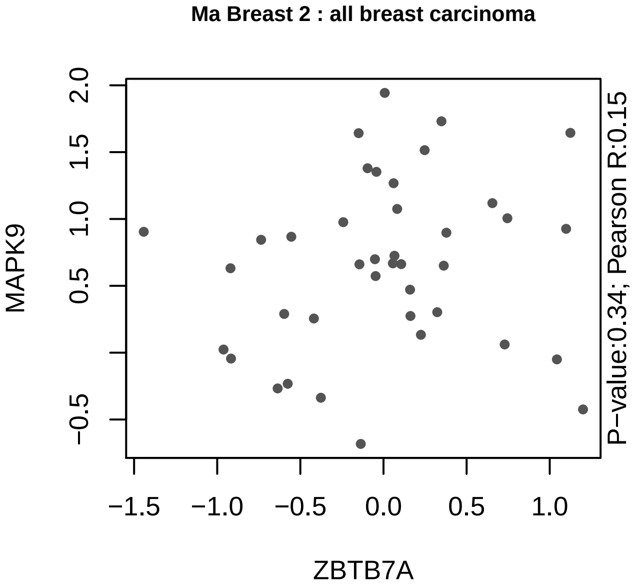
Ma Breast 2 : all breast carcinoma
MAPK9
−0.5 0.5 1.0 1.5 2.0
−1.5 −1.0 −0.5 0.0 0.5 1.0
ZBTB7A
P−value:0.34; Pearson R:0.15

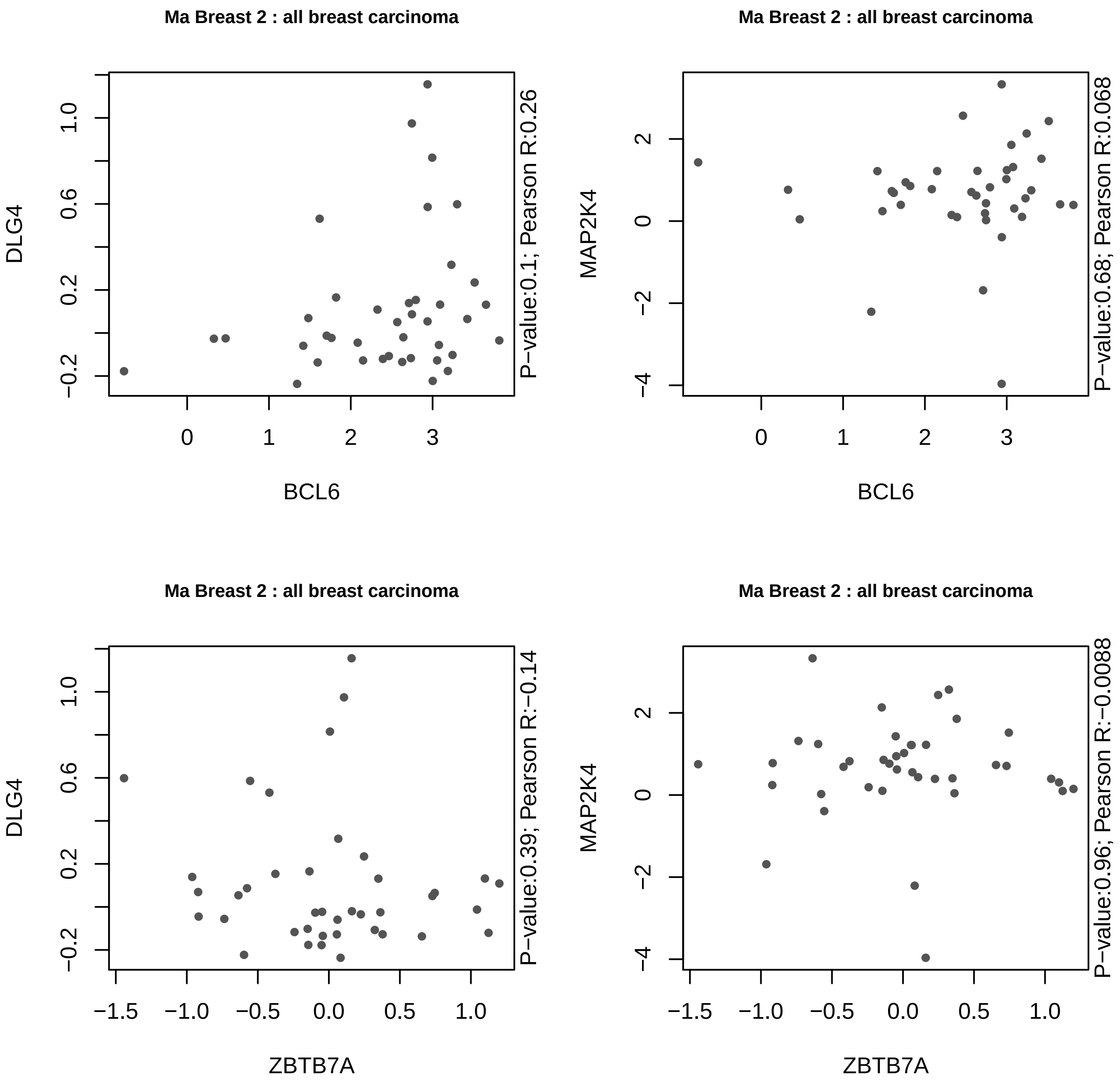
Ma Breast 2 : all breast carcinoma
DLG4
BCL6
P−value:0.1; Pearson R:0.26
Ma Breast 2 : all breast carcinoma
MAP2K4
BCL6
P−value:0.68; Pearson R:0.068
Ma Breast 2 : all breast carcinoma
DLG4
ZBTB7A
P−value:0.39; Pearson R:−0.14
Ma Breast 2 : all breast carcinoma
MAP2K4
ZBTB7A
P−value:0.96; Pearson R:−0.0088

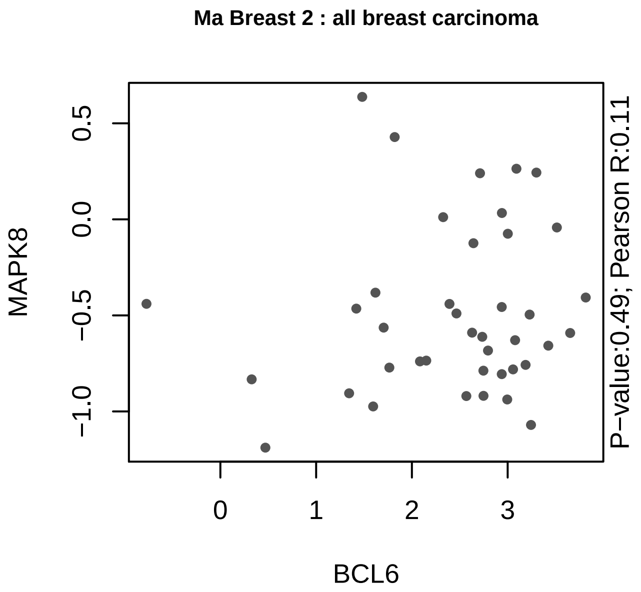
Ma Breast 2 : all breast carcinoma
MAPK8
BCL6
P−value:0.49; Pearson R:0.11

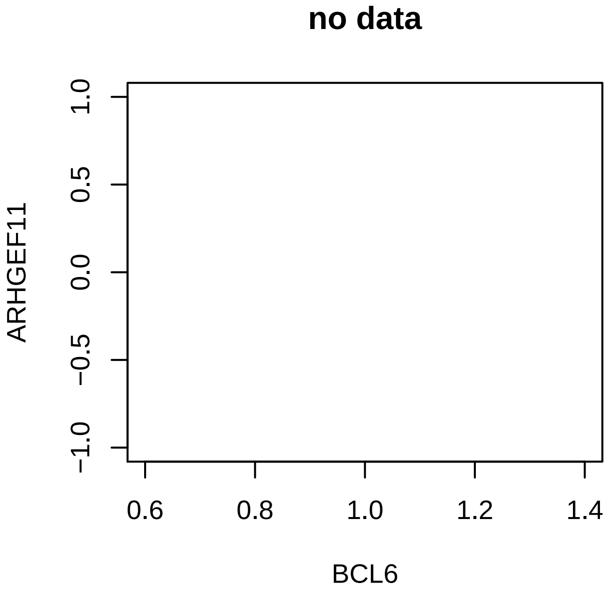
no data
ARHGEF11
BCL6

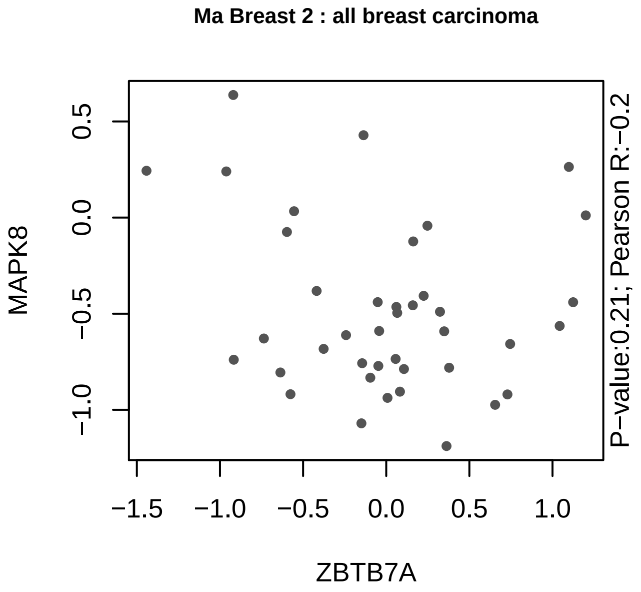
Ma Breast 2 : all breast carcinoma
MAPK8
ZBTB7A
P−value:0.21; Pearson R:−0.2

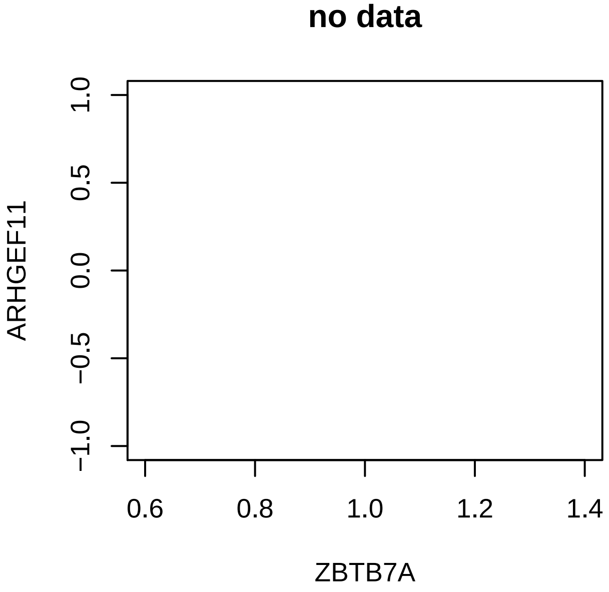
no data
ARHGEF11
ZBTB7A

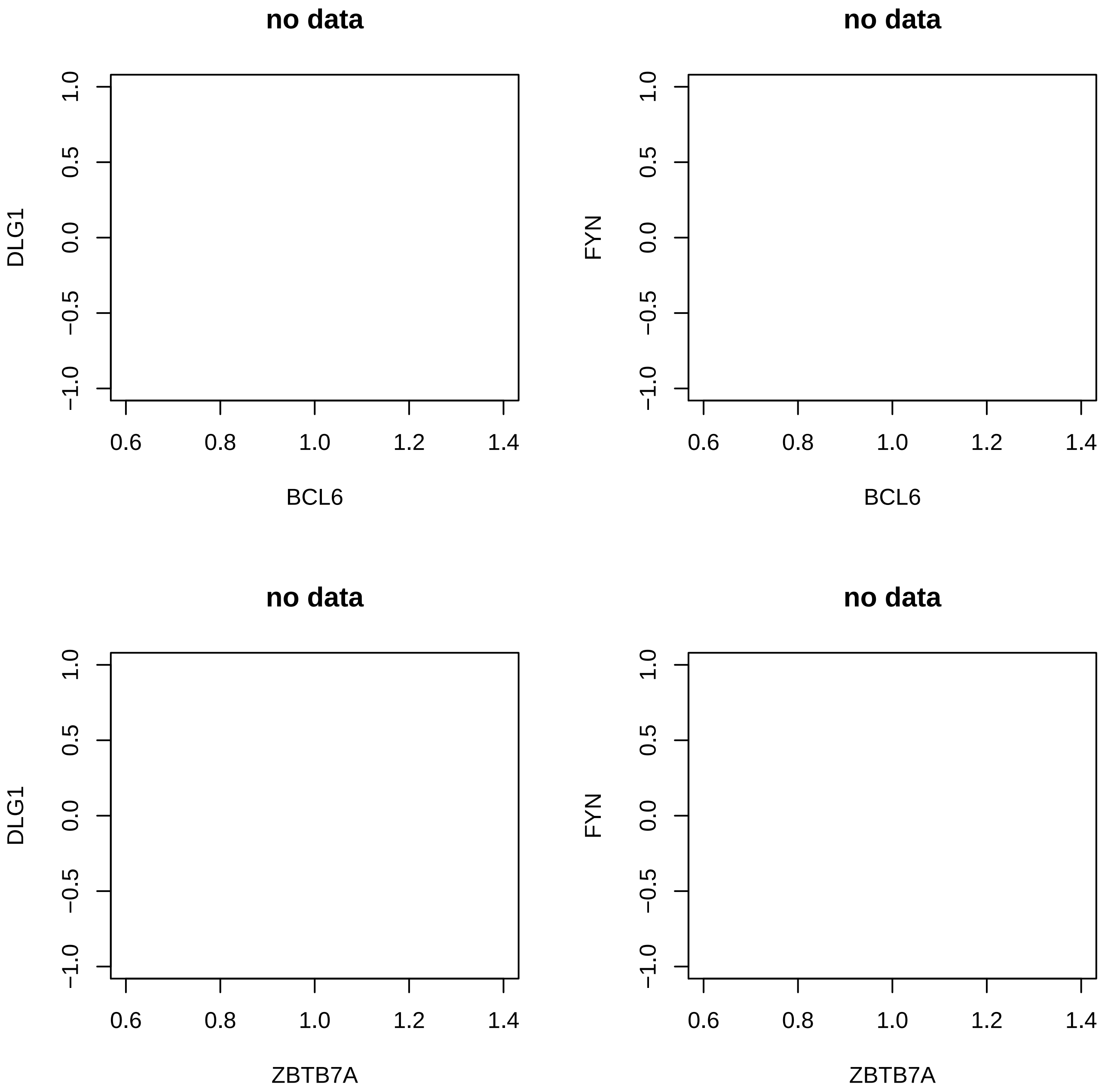
no data
DLG1
BCL6
no data
FYN
BCL6
no data
DLG1
ZBTB7A
no data
FYN
ZBTB7A
1.0
0.5
0.0
−0.5
−1.0
0.6
0.8
1.0
1.2
1.4

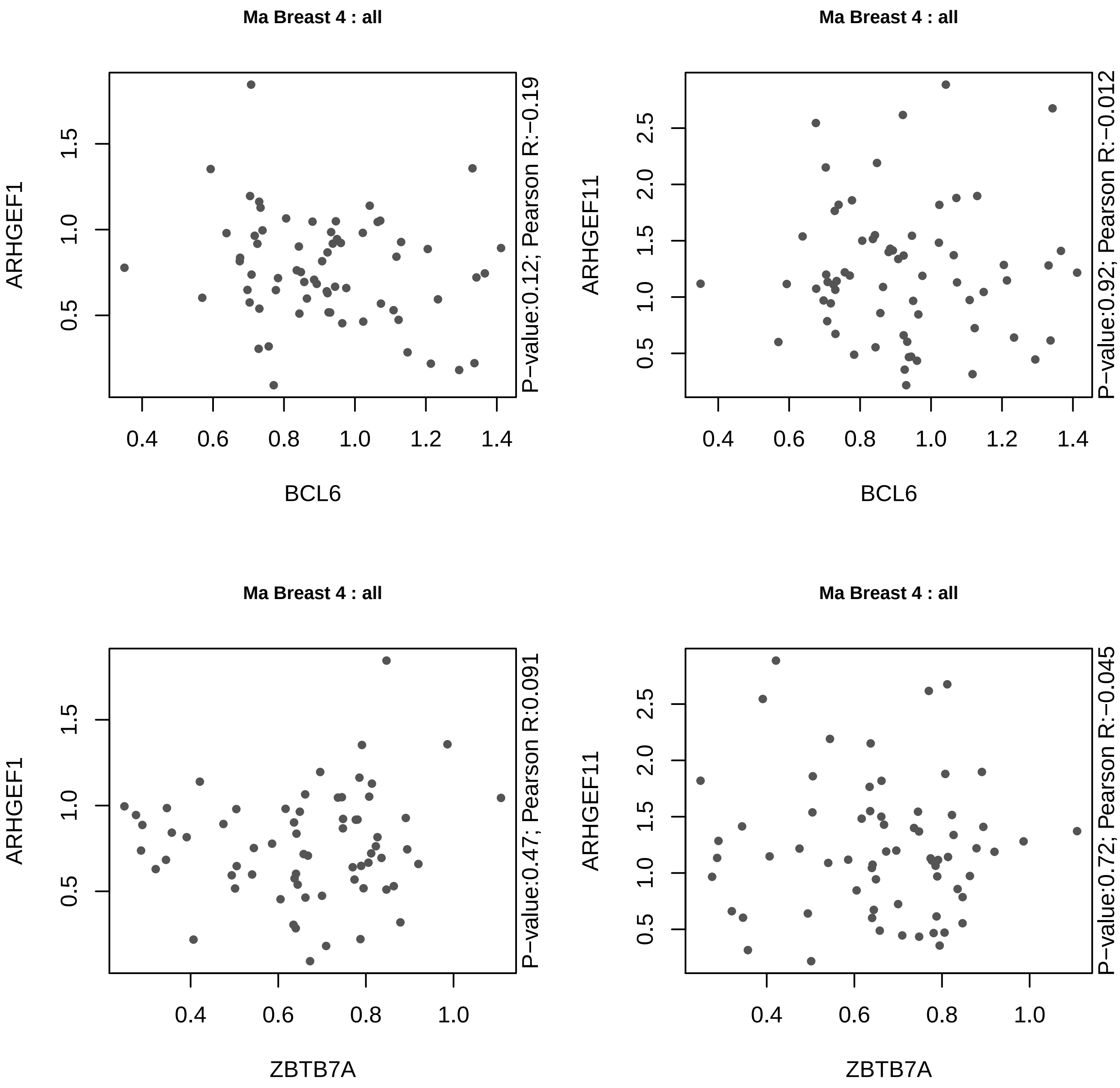
Ma Breast 4 : all
ARHGEF1
BCL6
P−value:0.12; Pearson R:−0.19
Ma Breast 4 : all
ARHGEF11
BCL6
P−value:0.92; Pearson R:−0.012
Ma Breast 4 : all
ARHGEF1
ZBTB7A
P−value:0.47; Pearson R:0.091
Ma Breast 4 : all
ARHGEF11
ZBTB7A
P−value:0.72; Pearson R:−0.045

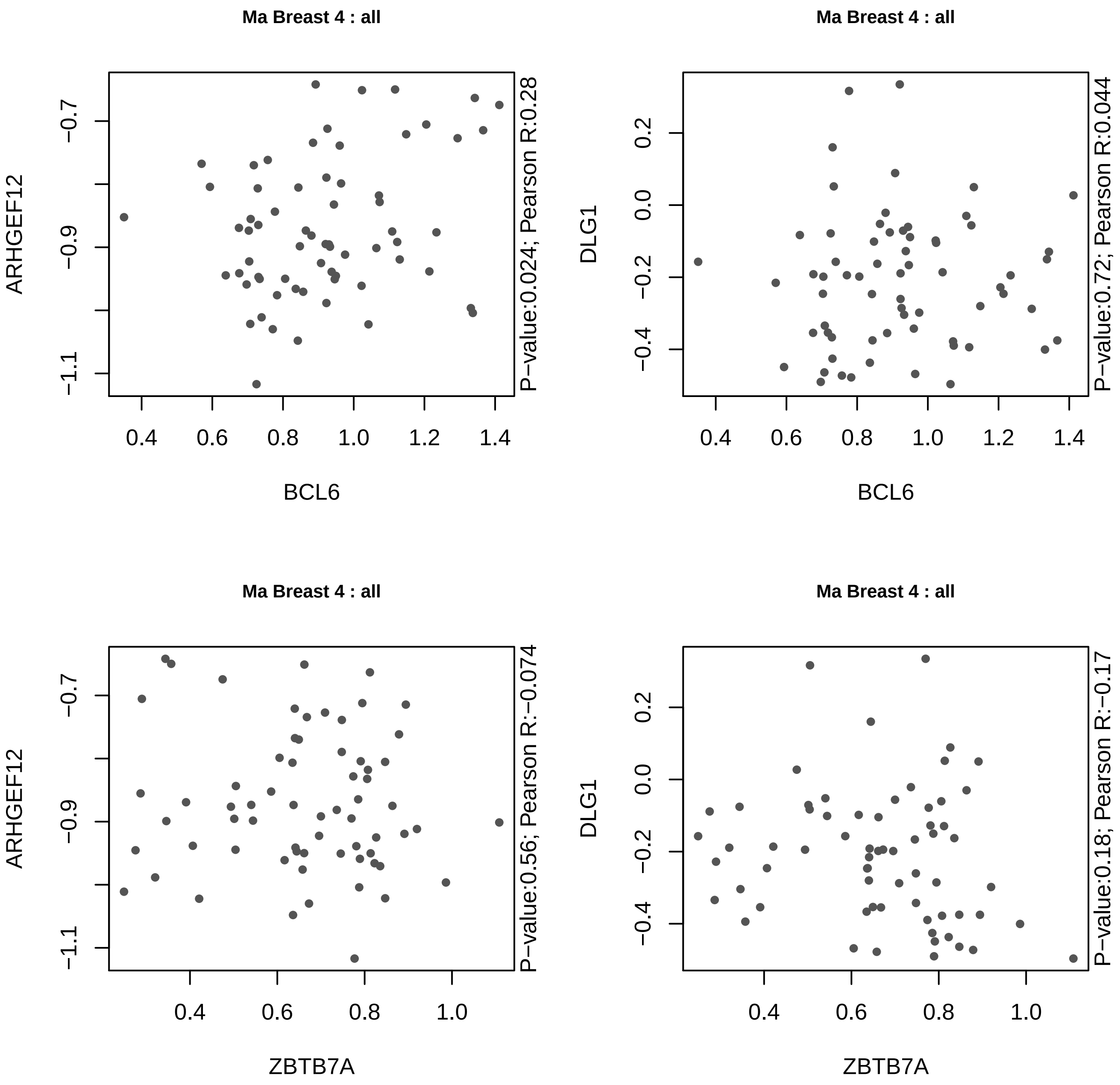
Ma Breast 4 : all
ARHGEF12
BCL6
P−value:0.024; Pearson R:0.28
Ma Breast 4 : all
DLG1
BCL6
P−value:0.72; Pearson R:0.044
Ma Breast 4 : all
ARHGEF12
ZBTB7A
P−value:0.56; Pearson R:−0.074
Ma Breast 4 : all
DLG1
ZBTB7A
P−value:0.18; Pearson R:−0.17

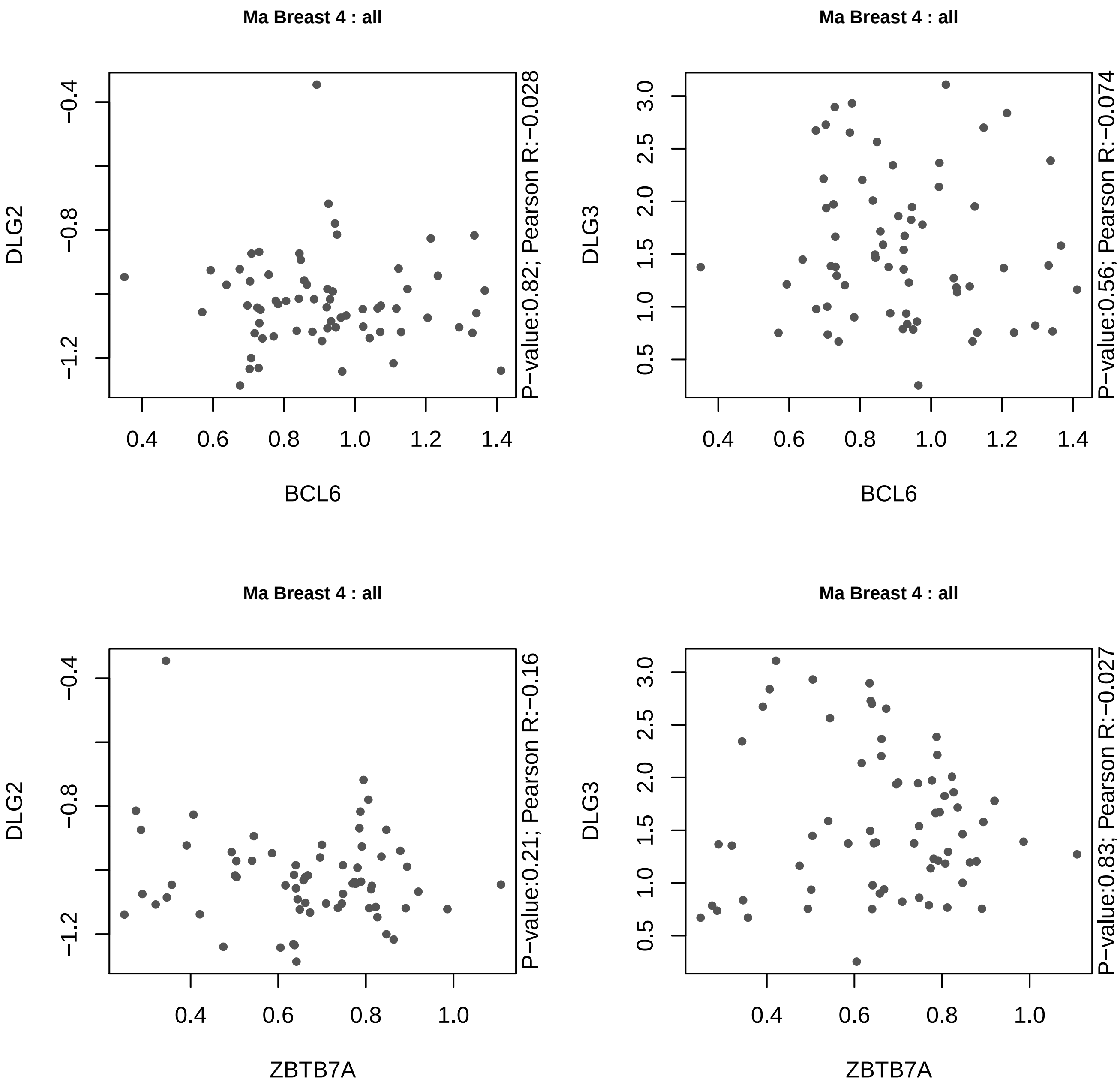
Ma Breast 4 : all
DLG2
BCL6
P−value:0.82; Pearson R:−0.028
Ma Breast 4 : all
DLG3
BCL6
P−value:0.56; Pearson R:−0.074
Ma Breast 4 : all
DLG2
ZBTB7A
P−value:0.21; Pearson R:−0.16
Ma Breast 4 : all
DLG3
ZBTB7A
P−value:0.83; Pearson R:−0.027

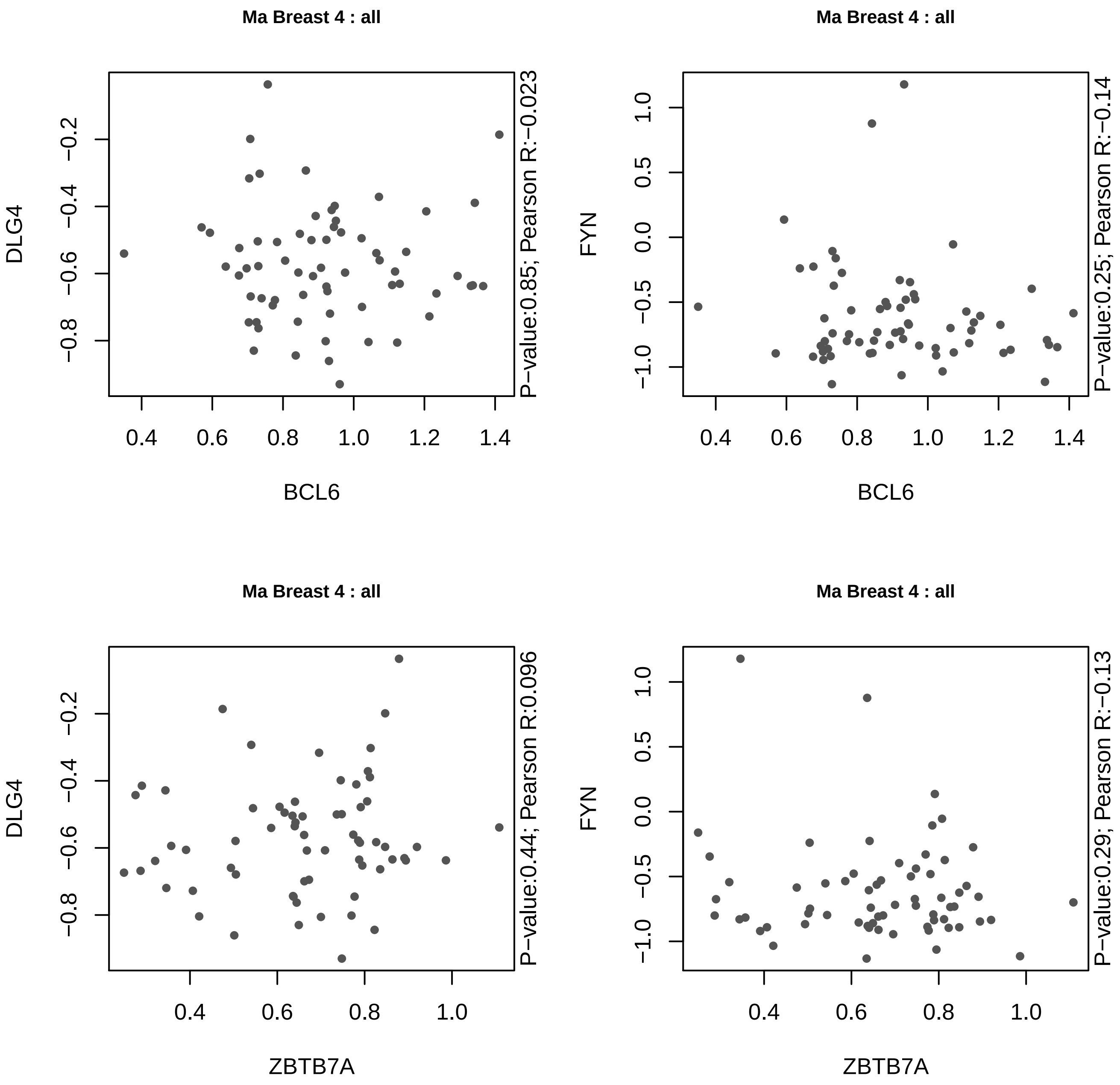
Ma Breast 4 : all
DLG4
BCL6
P−value:0.85; Pearson R:−0.023
Ma Breast 4 : all
FYN
BCL6
P−value:0.25; Pearson R:−0.14
Ma Breast 4 : all
DLG4
ZBTB7A
P−value:0.44; Pearson R:0.096
Ma Breast 4 : all
FYN
ZBTB7A
P−value:0.29; Pearson R:−0.13

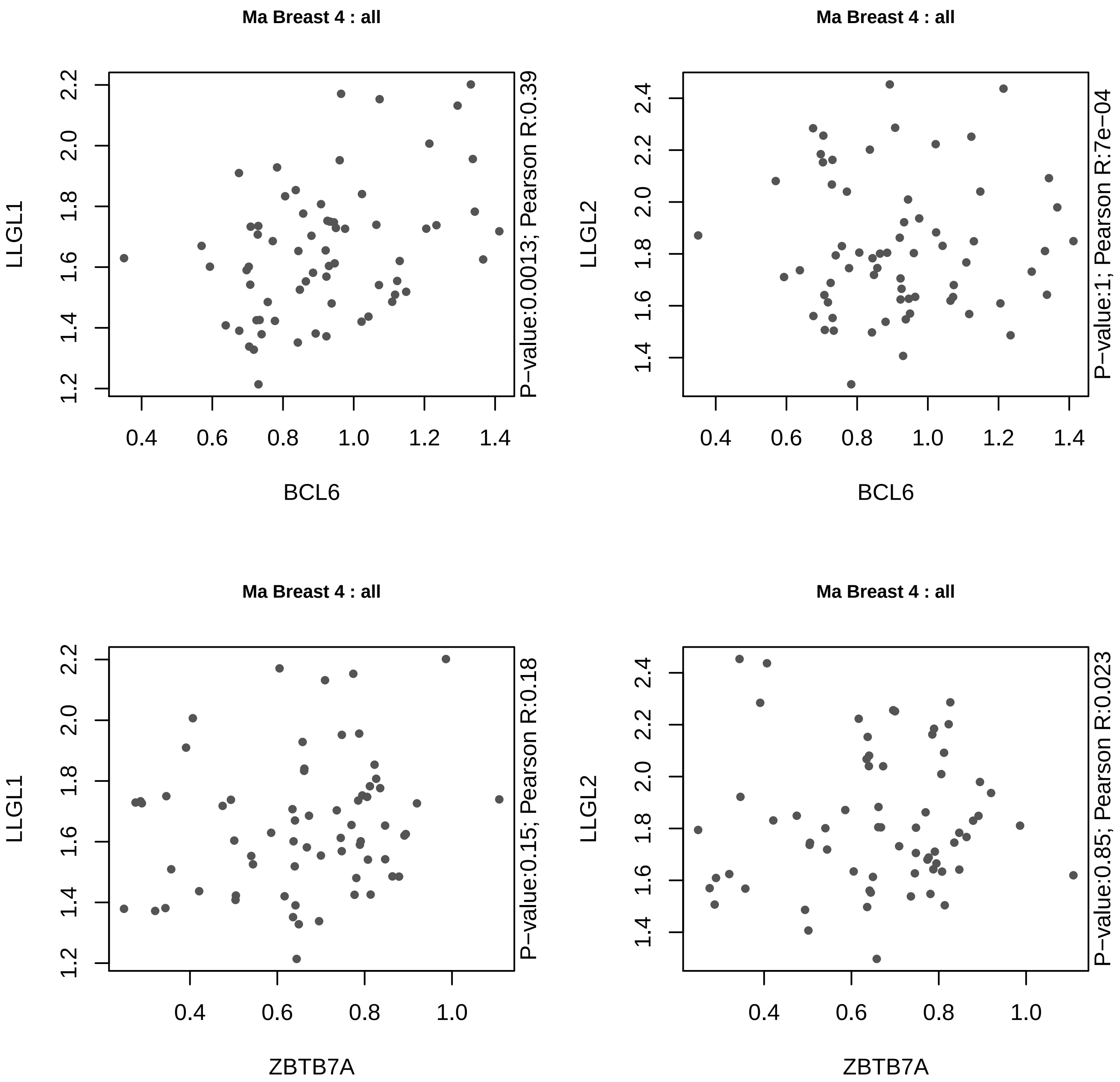
Ma Breast 4 : all
LLGL1
BCL6
P−value:0.0013; Pearson R:0.39
Ma Breast 4 : all
LLGL2
BCL6
P−value:1; Pearson R:7e−04
Ma Breast 4 : all
LLGL1
ZBTB7A
P−value:0.15; Pearson R:0.18
Ma Breast 4 : all
LLGL2
ZBTB7A
P−value:0.85; Pearson R:0.023

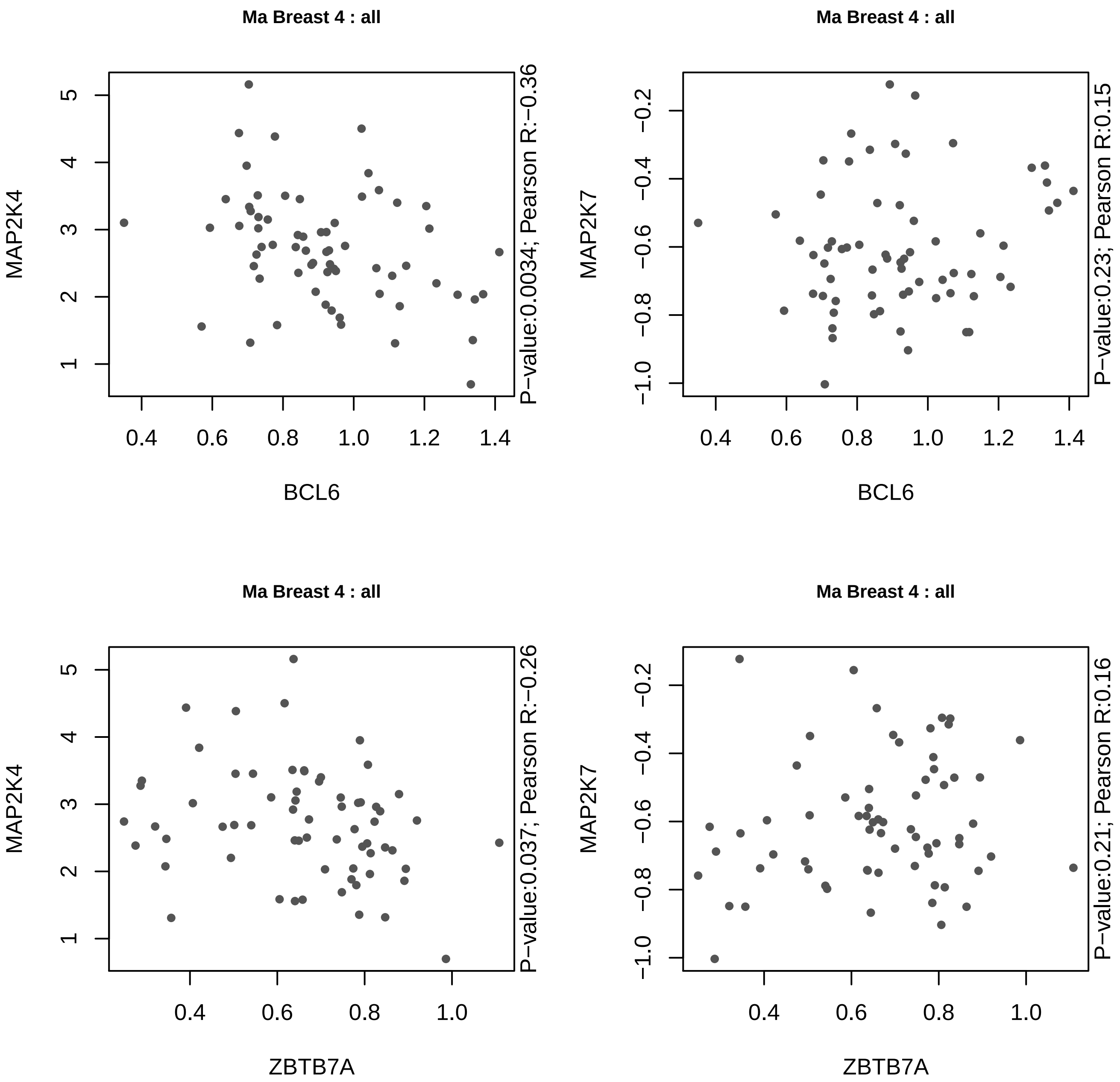

Ma Breast 4 : all
MAP2K4
BCL6
P−value:0.0034; Pearson R:−0.36
Ma Breast 4 : all
MAP2K7
BCL6
P−value:0.23; Pearson R:0.15
Ma Breast 4 : all
MAP2K4
ZBTB7A
P−value:0.037; Pearson R:−0.26
Ma Breast 4 : all
MAP2K7
ZBTB7A
P−value:0.21; Pearson R:0.16

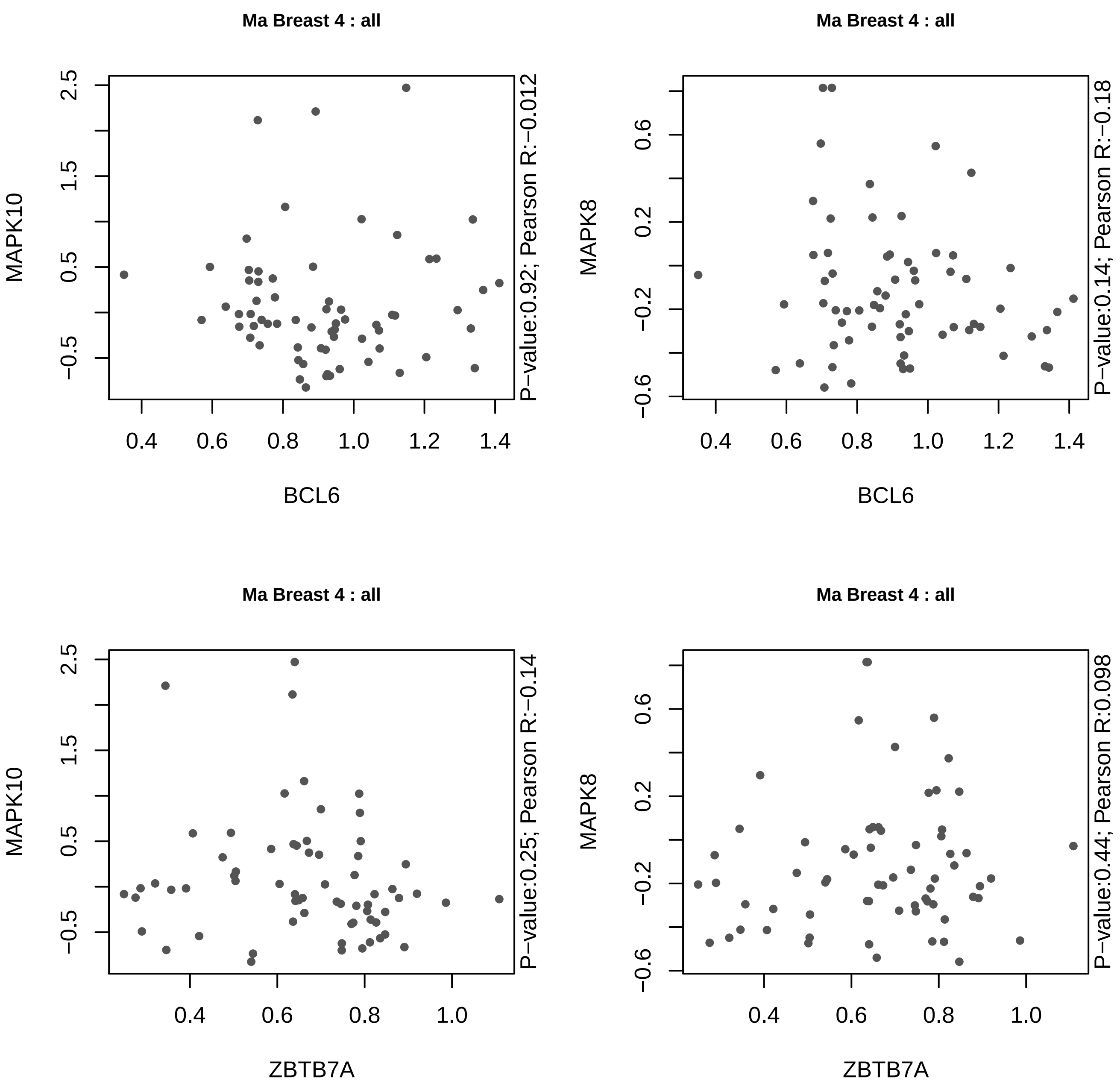
Ma Breast 4 : all
MAPK10
BCL6
P−value:0.92; Pearson R:−0.012
Ma Breast 4 : all
MAPK8
BCL6
P−value:0.14; Pearson R:−0.18
Ma Breast 4 : all
MAPK10
ZBTB7A
P−value:0.25; Pearson R:−0.14
Ma Breast 4 : all
MAPK8
ZBTB7A
P−value:0.44; Pearson R:0.098

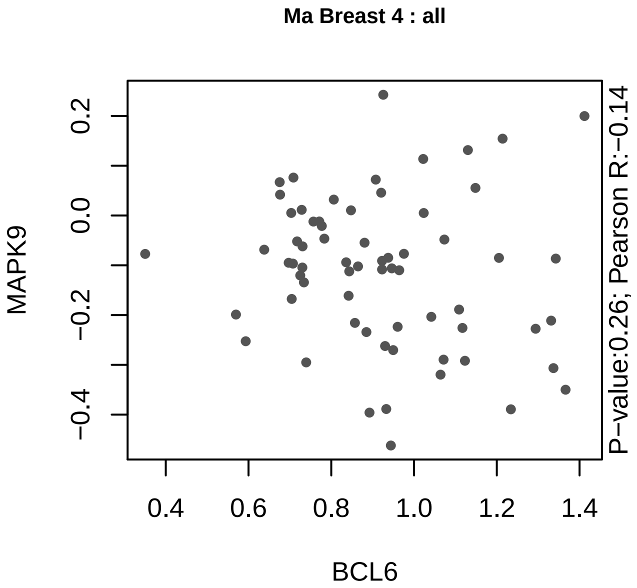
Ma Breast 4 : all
MAPK9
BCL6
P−value:0.26; Pearson R:−0.14
0.2
0.0
−0.2
−0.4
0.4
0.6
0.8
1.0
1.2
1.4

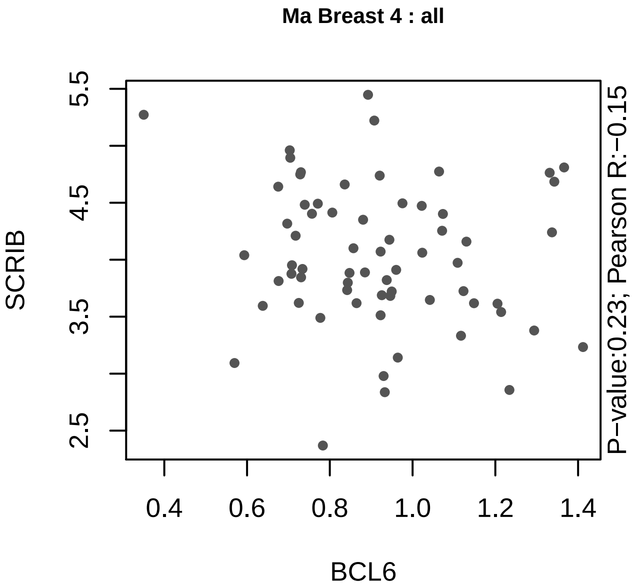
Ma Breast 4 : all
SCRIB
BCL6
P−value:0.23; Pearson R:−0.15
5.5
4.5
3.5
2.5
0.4
0.6
0.8
1.0
1.2
1.4

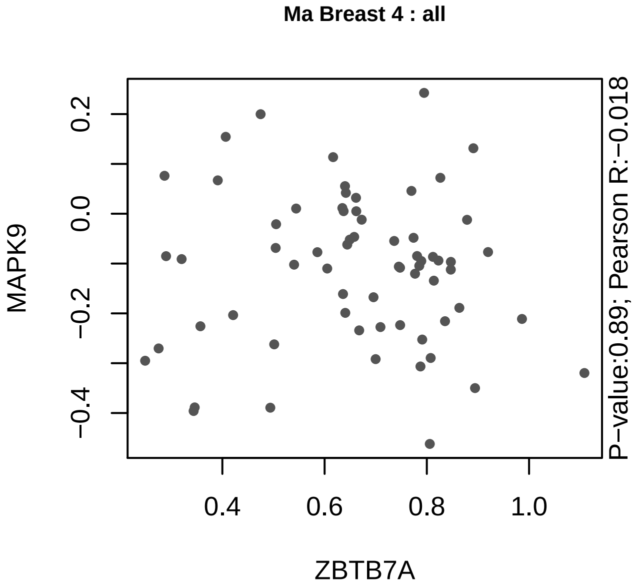
Ma Breast 4 : all
MAPK9
ZBTB7A
P−value:0.89; Pearson R:−0.018
0.2
0.0
−0.2
−0.4
0.4
0.6
0.8
1.0

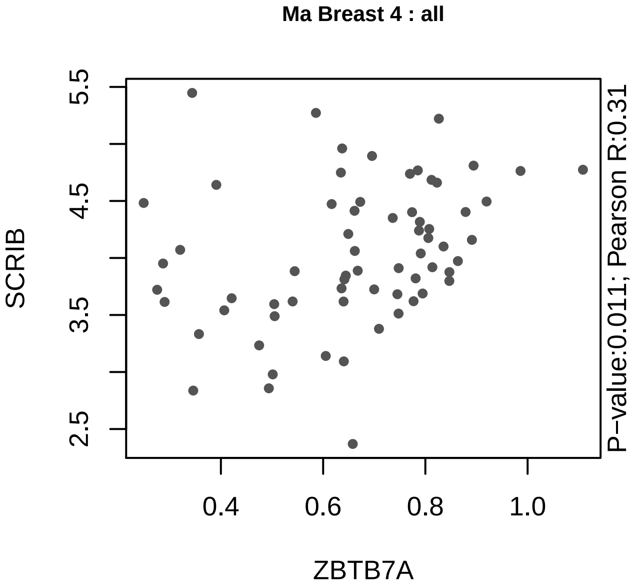
Ma Breast 4 : all
SCRIB
ZBTB7A
P−value:0.011; Pearson R:0.31
5.5
4.5
3.5
2.5
0.4
0.6
0.8
1.0

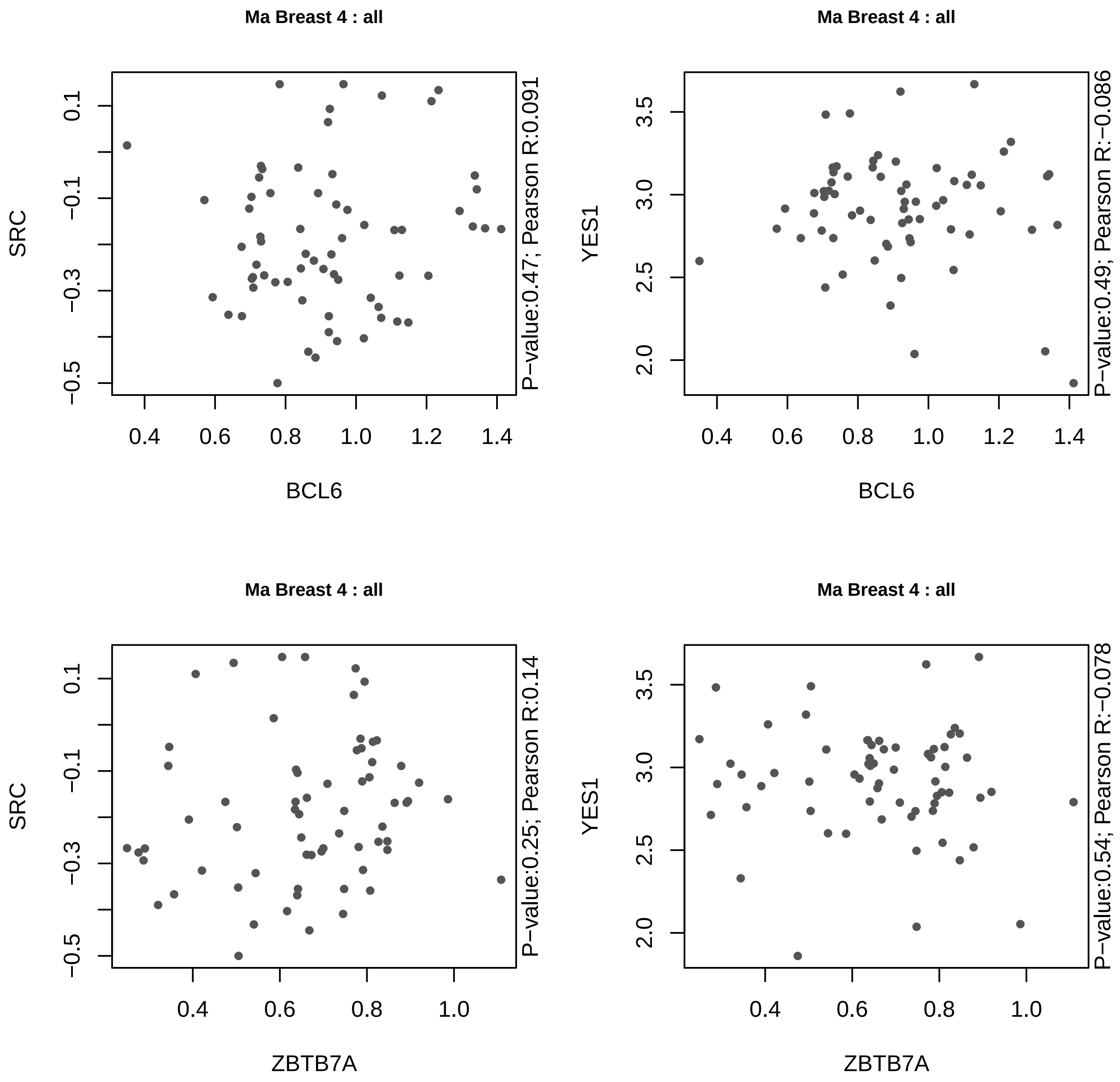
Ma Breast 4 : all
SRC
BCL6
P−value:0.47; Pearson R:0.091
0.1
−0.1
−0.3
−0.5
0.4
0.6
0.8
1.0
1.2
1.4
Ma Breast 4 : all
YES1
BCL6
P−value:0.49; Pearson R:−0.086
3.5
3.0
2.5
2.0
0.4
0.6
0.8
1.0
1.2
1.4
Ma Breast 4 : all
SRC
ZBTB7A
P−value:0.25; Pearson R:0.14
0.1
−0.1
−0.3
−0.5
0.4
0.6
0.8
1.0
Ma Breast 4 : all
YES1
ZBTB7A
P−value:0.54; Pearson R:−0.078
3.5
3.0
2.5
2.0
0.4
0.6
0.8
1.0

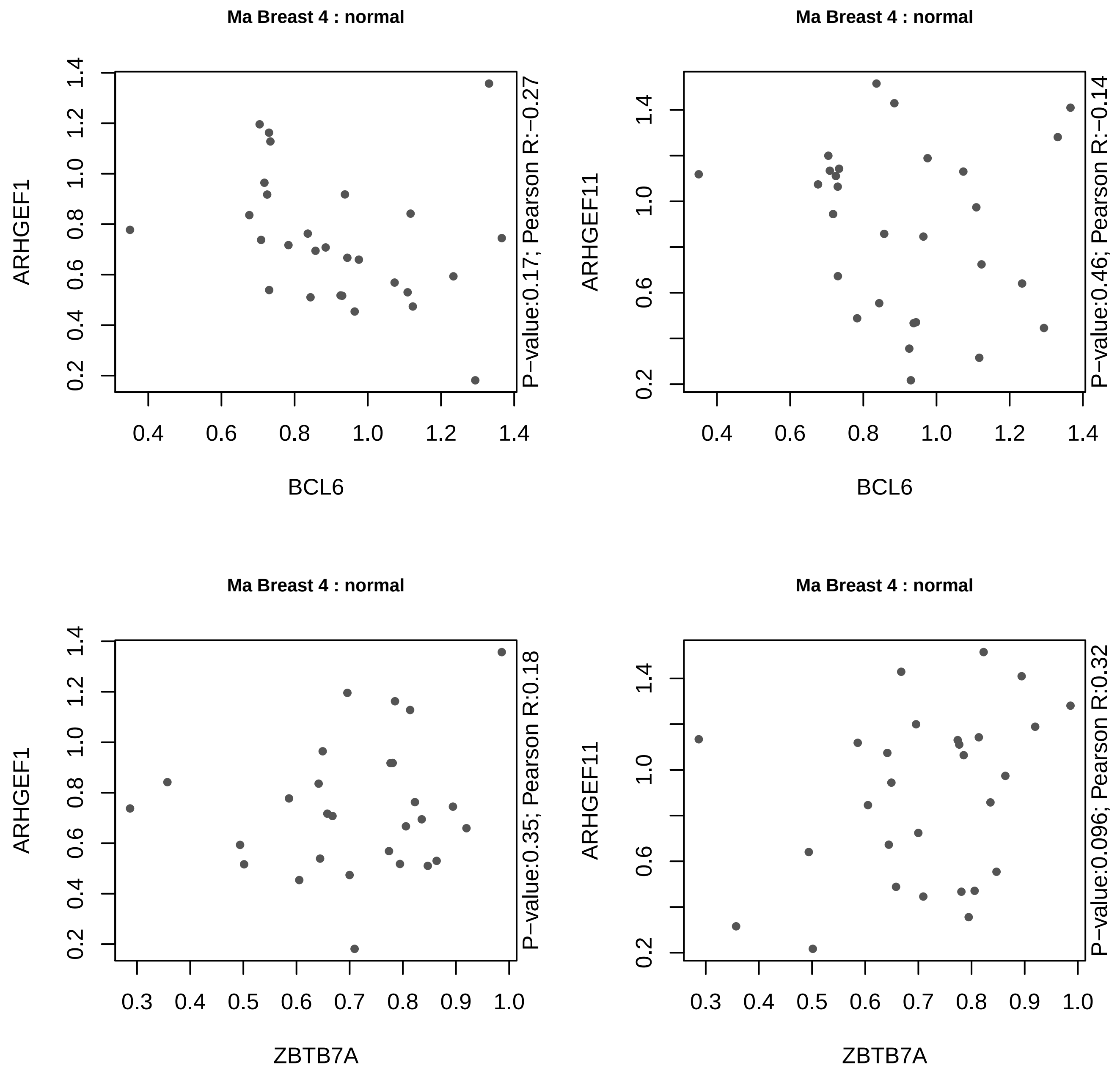
Ma Breast 4 : normal
ARHGEF1
BCL6
P−value:0.17; Pearson R:−0.27
Ma Breast 4 : normal
ARHGEF11
BCL6
P−value:0.46; Pearson R:−0.14
Ma Breast 4 : normal
ARHGEF1
ZBTB7A
P−value:0.35; Pearson R:0.18
Ma Breast 4 : normal
ARHGEF11
ZBTB7A
P−value:0.096; Pearson R:0.32

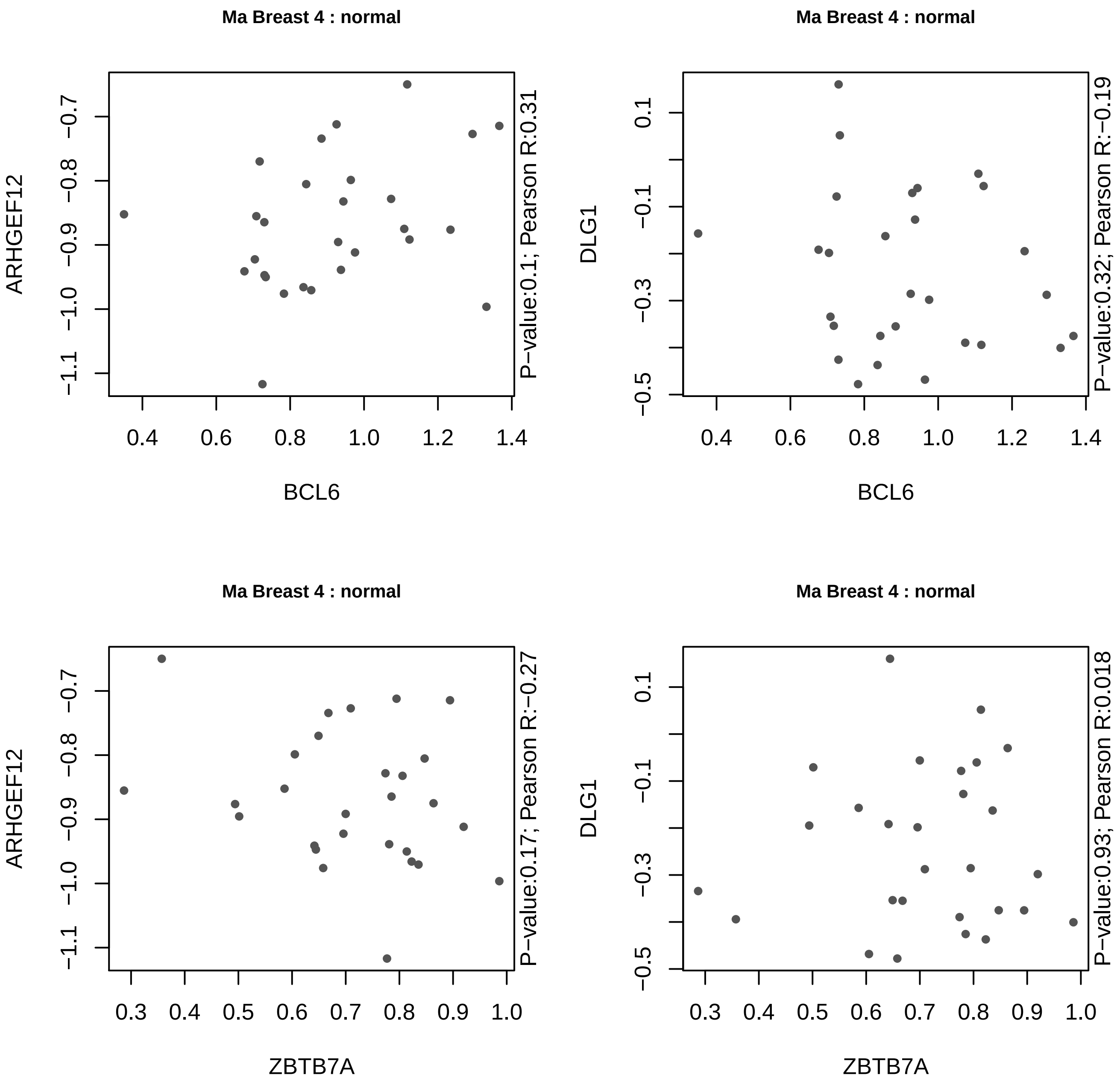
Ma Breast 4 : normal
ARHGEF12
BCL6
P−value:0.1; Pearson R:0.31
Ma Breast 4 : normal
DLG1
BCL6
P−value:0.32; Pearson R:−0.19
Ma Breast 4 : normal
ARHGEF12
ZBTB7A
P−value:0.17; Pearson R:−0.27
Ma Breast 4 : normal
DLG1
ZBTB7A
P−value:0.93; Pearson R:0.018

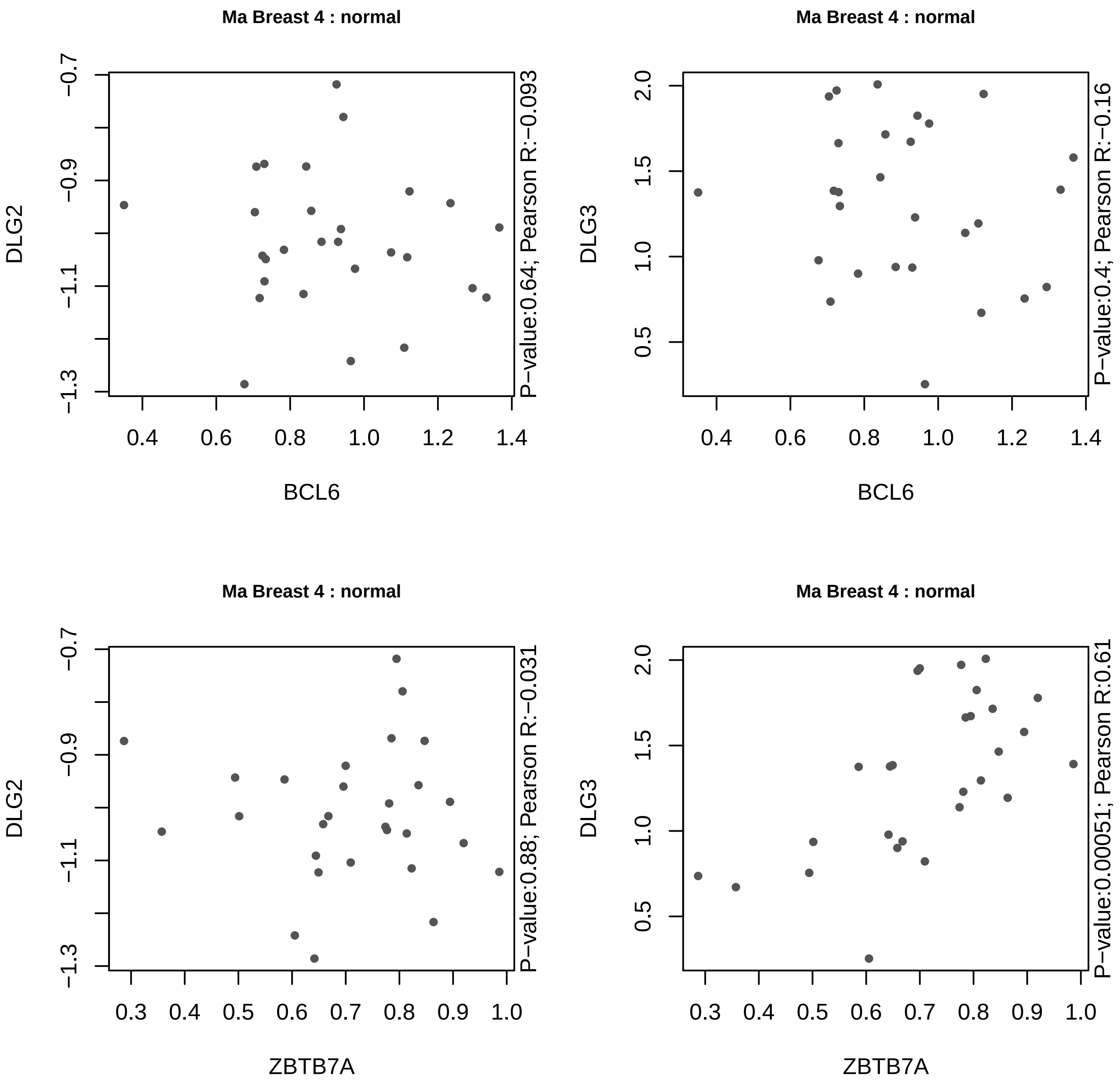
Ma Breast 4 : normal
DLG2
BCL6
P−value:0.64; Pearson R:−0.093
Ma Breast 4 : normal
DLG3
BCL6
P−value:0.4; Pearson R:−0.16
Ma Breast 4 : normal
DLG2
ZBTB7A
P−value:0.88; Pearson R:−0.031
Ma Breast 4 : normal
DLG3
ZBTB7A
P−value:0.00051; Pearson R:0.61

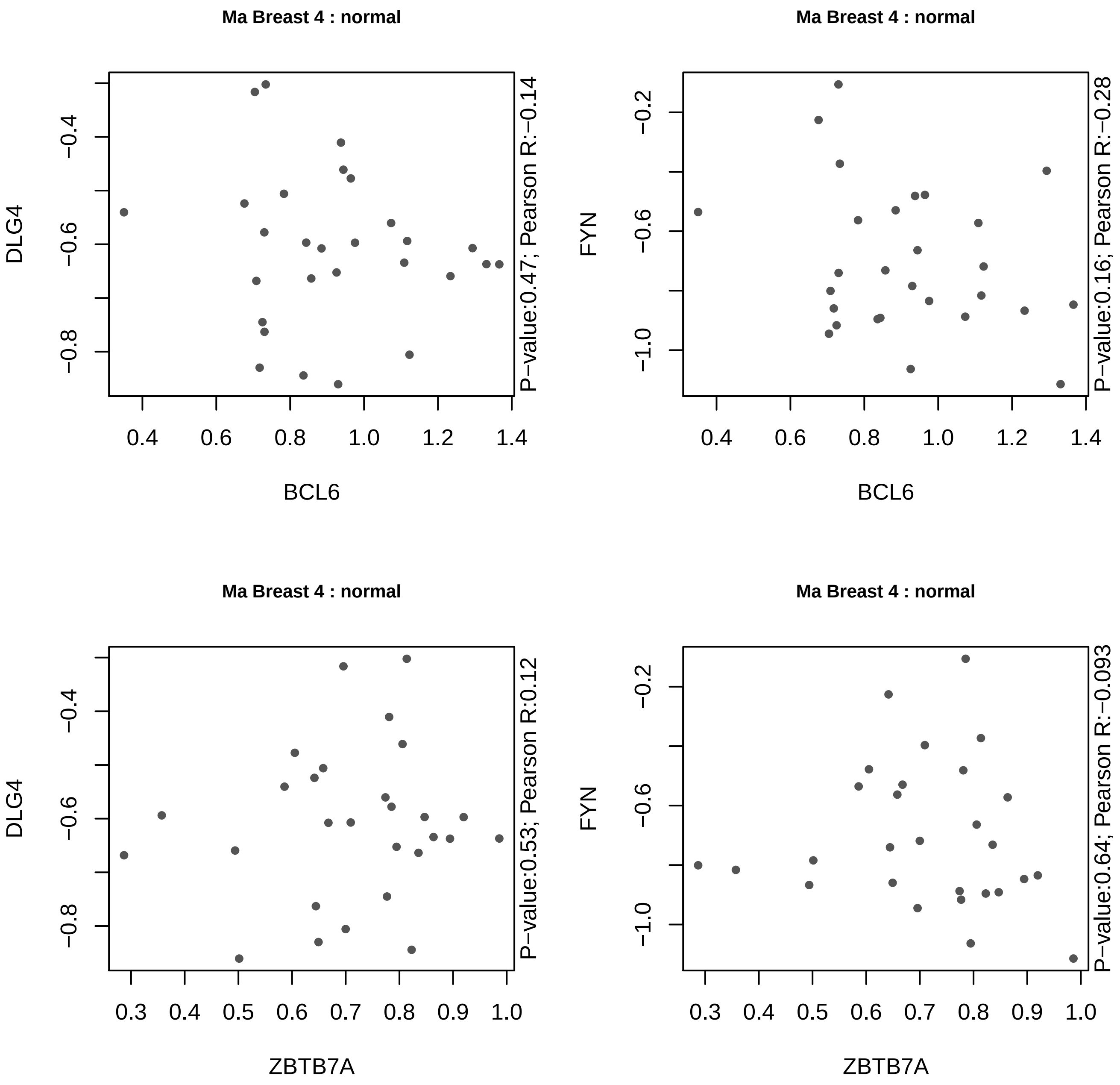
Ma Breast 4 : normal
DLG4
BCL6
P−value:0.47; Pearson R:−0.14
Ma Breast 4 : normal
FYN
BCL6
P−value:0.16; Pearson R:−0.28
Ma Breast 4 : normal
DLG4
ZBTB7A
P−value:0.53; Pearson R:0.12
Ma Breast 4 : normal
FYN
ZBTB7A
P−value:0.64; Pearson R:−0.093

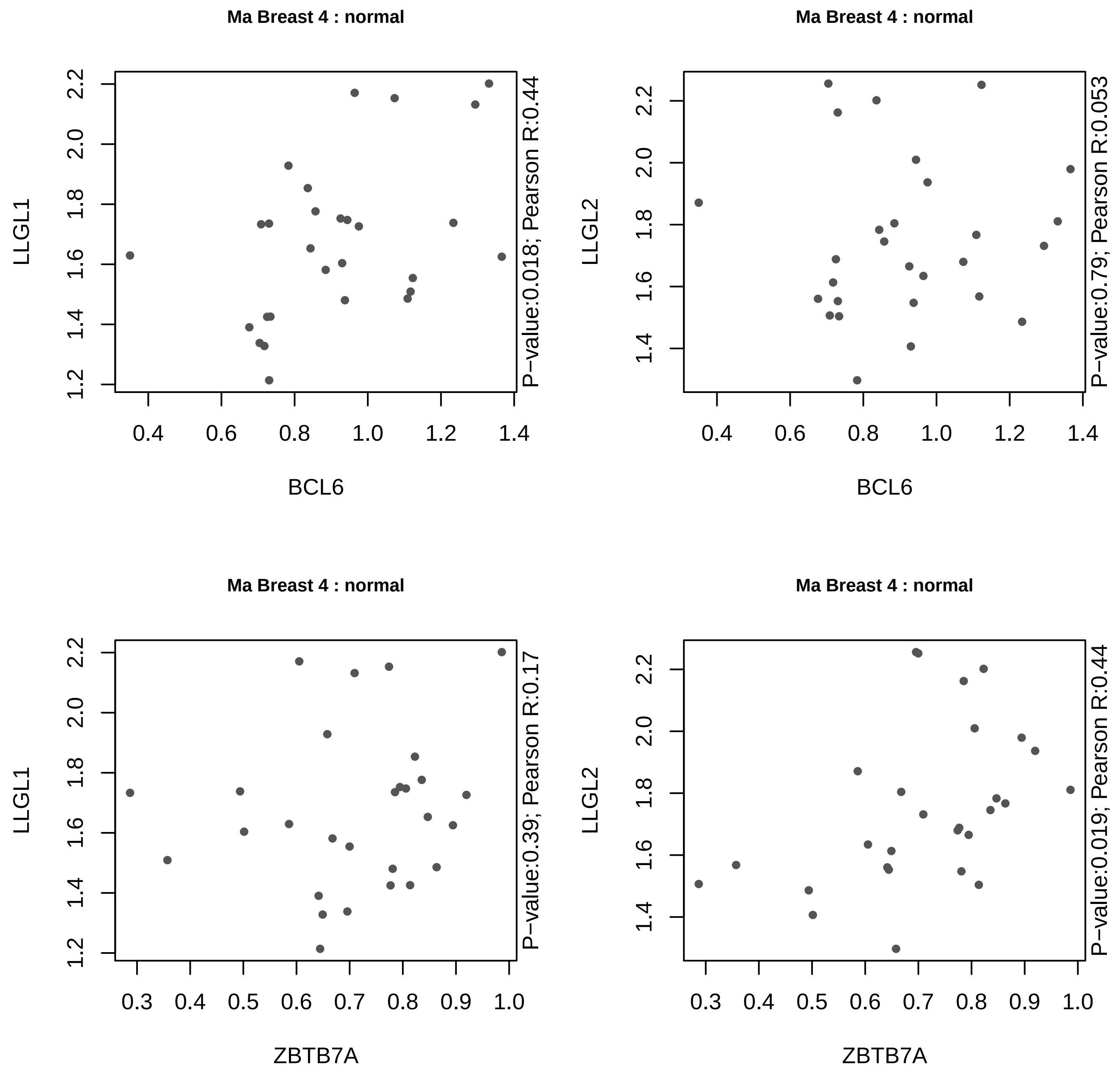
Ma Breast 4 : normal
LLGL1
BCL6
P−value:0.018; Pearson R:0.44
Ma Breast 4 : normal
LLGL2
BCL6
P−value:0.79; Pearson R:0.053
Ma Breast 4 : normal
LLGL1
ZBTB7A
P−value:0.39; Pearson R:0.17
Ma Breast 4 : normal
LLGL2
ZBTB7A
P−value:0.019; Pearson R:0.44

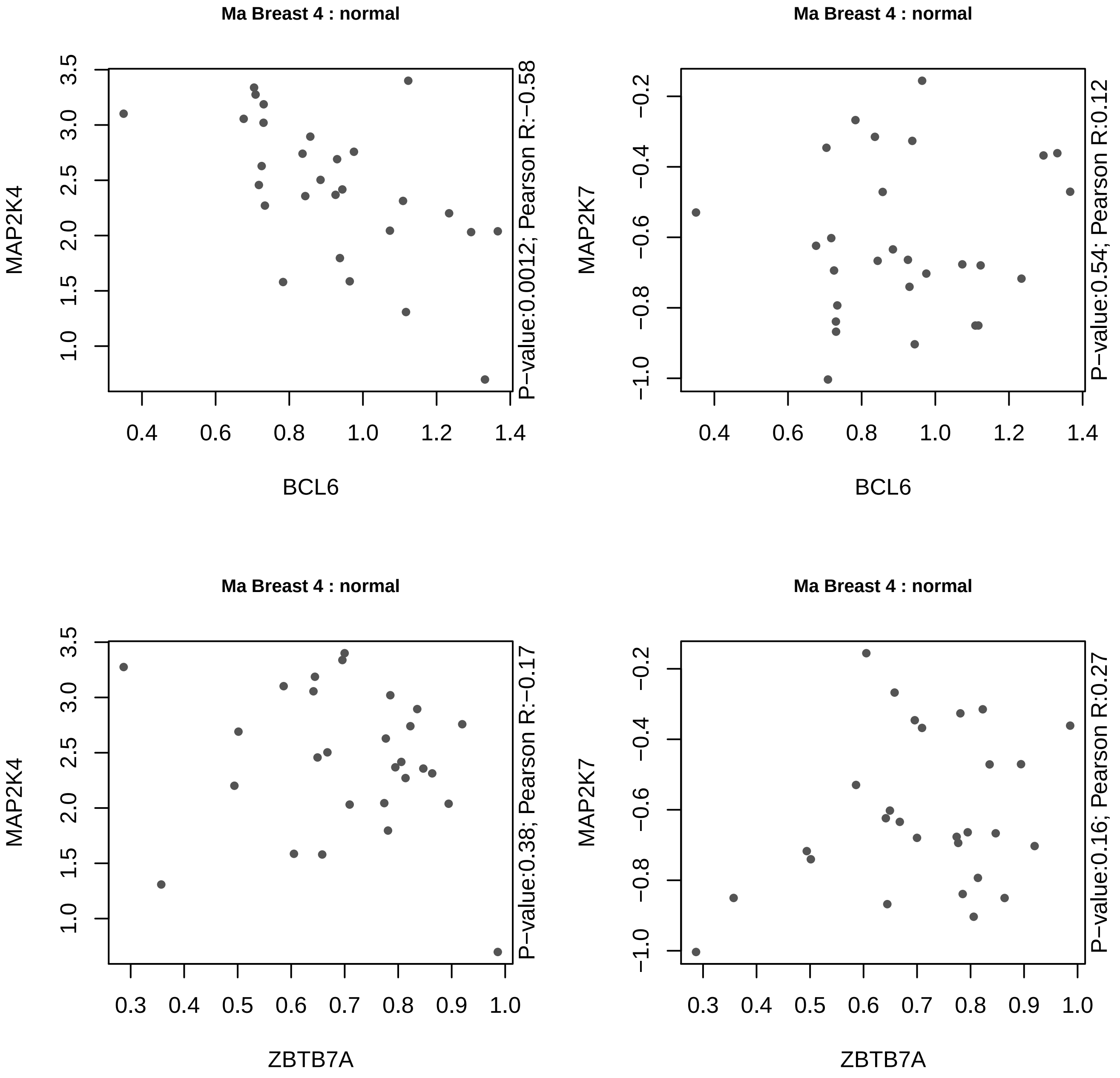
Ma Breast 4 : normal
MAP2K4
BCL6
P−value:0.0012; Pearson R:−0.58
Ma Breast 4 : normal
MAP2K7
BCL6
P−value:0.54; Pearson R:0.12
Ma Breast 4 : normal
MAP2K4
ZBTB7A
P−value:0.38; Pearson R:−0.17
Ma Breast 4 : normal
MAP2K7
ZBTB7A
P−value:0.16; Pearson R:0.27

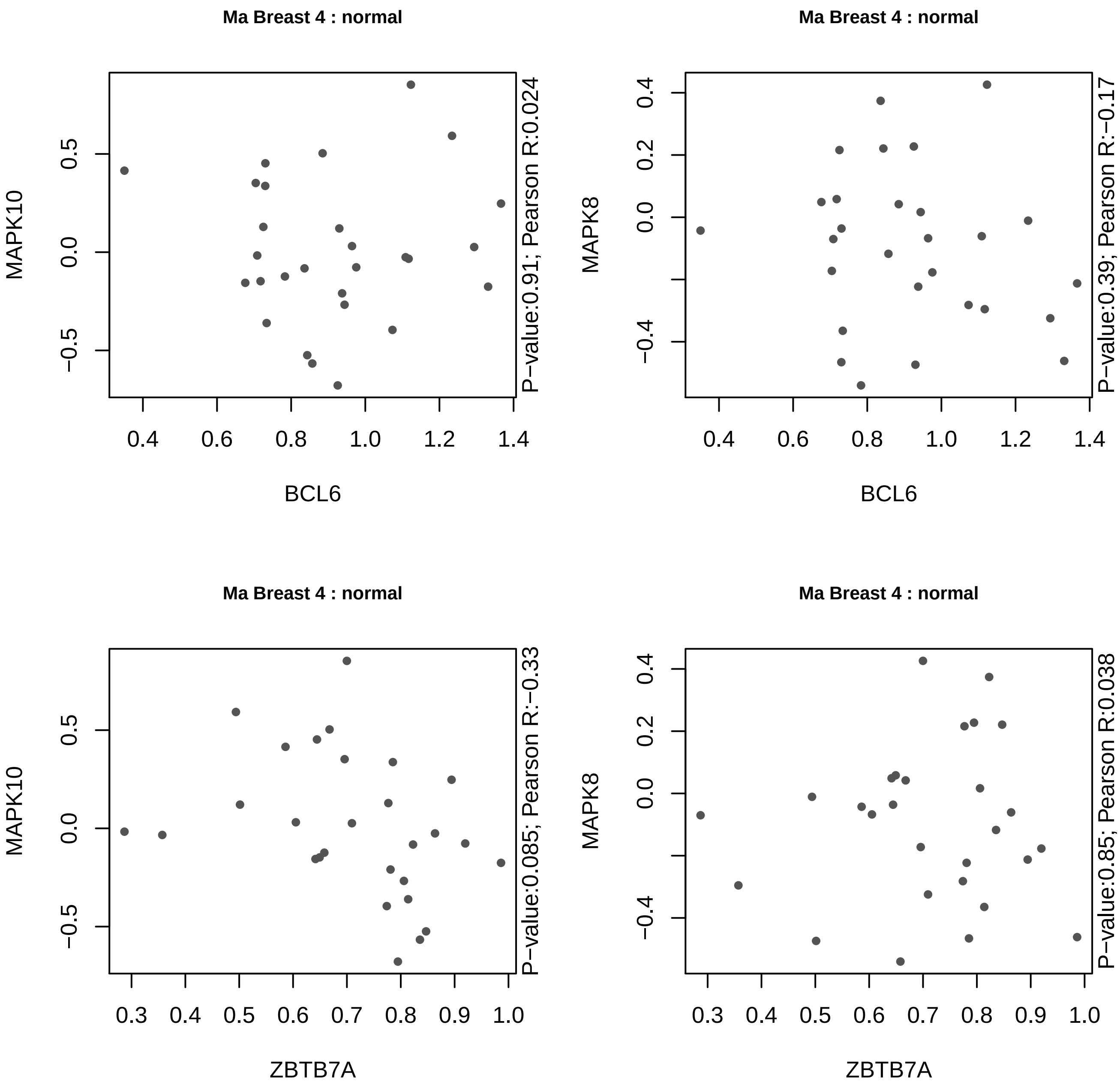
Ma Breast 4 : normal
MAPK10
BCL6
P−value:0.91; Pearson R:0.024
Ma Breast 4 : normal
MAPK8
BCL6
P−value:0.39; Pearson R:−0.17
Ma Breast 4 : normal
MAPK10
ZBTB7A
P−value:0.085; Pearson R:−0.33
Ma Breast 4 : normal
MAPK8
ZBTB7A
P−value:0.85; Pearson R:0.038

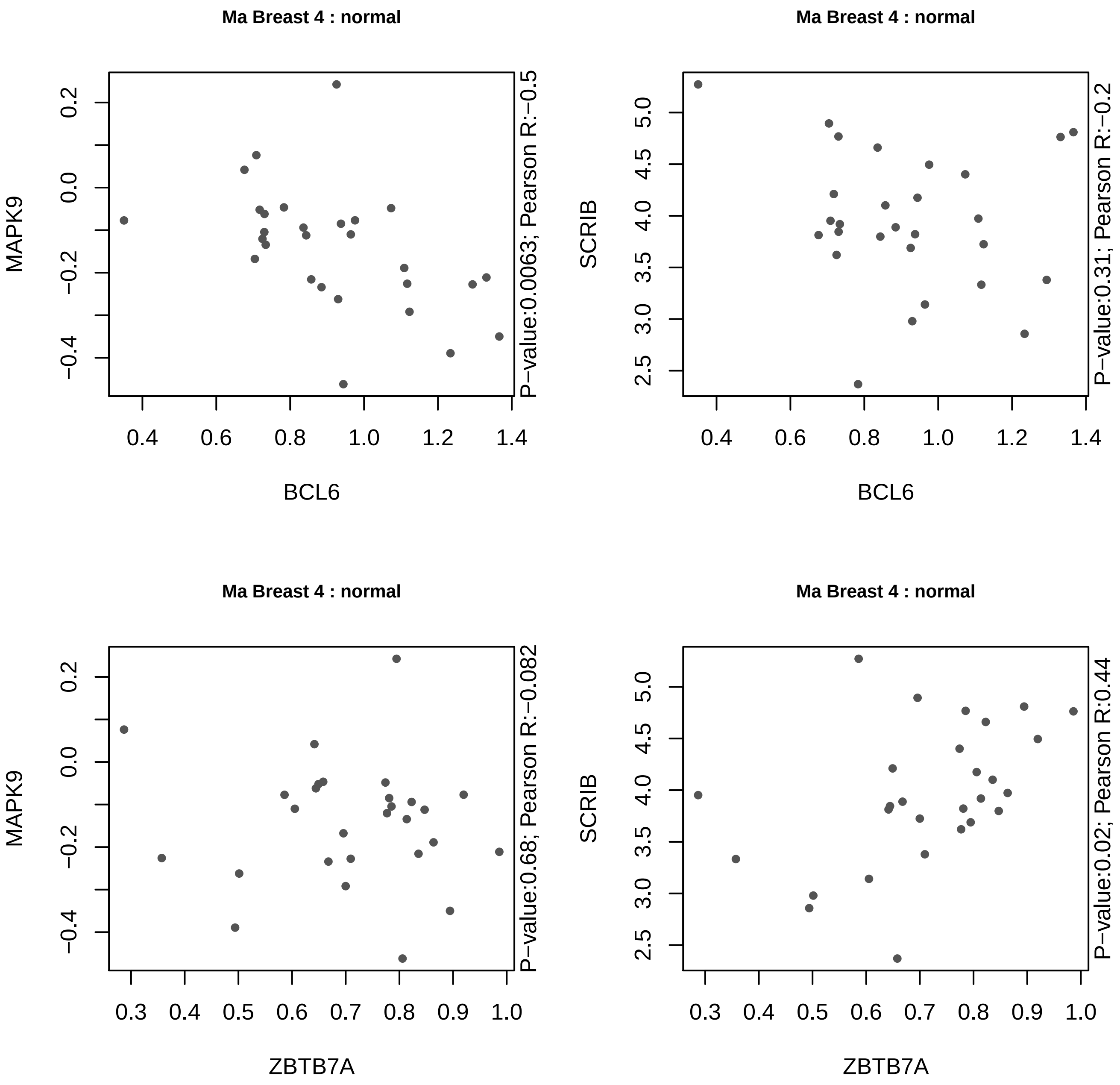
Ma Breast 4 : normal
MAPK9
BCL6
P−value:0.0063; Pearson R:−0.5
Ma Breast 4 : normal
SCRIB
BCL6
P−value:0.31; Pearson R:−0.2
Ma Breast 4 : normal
MAPK9
ZBTB7A
P−value:0.68; Pearson R:−0.082
Ma Breast 4 : normal
SCRIB
ZBTB7A
P−value:0.02; Pearson R:0.44

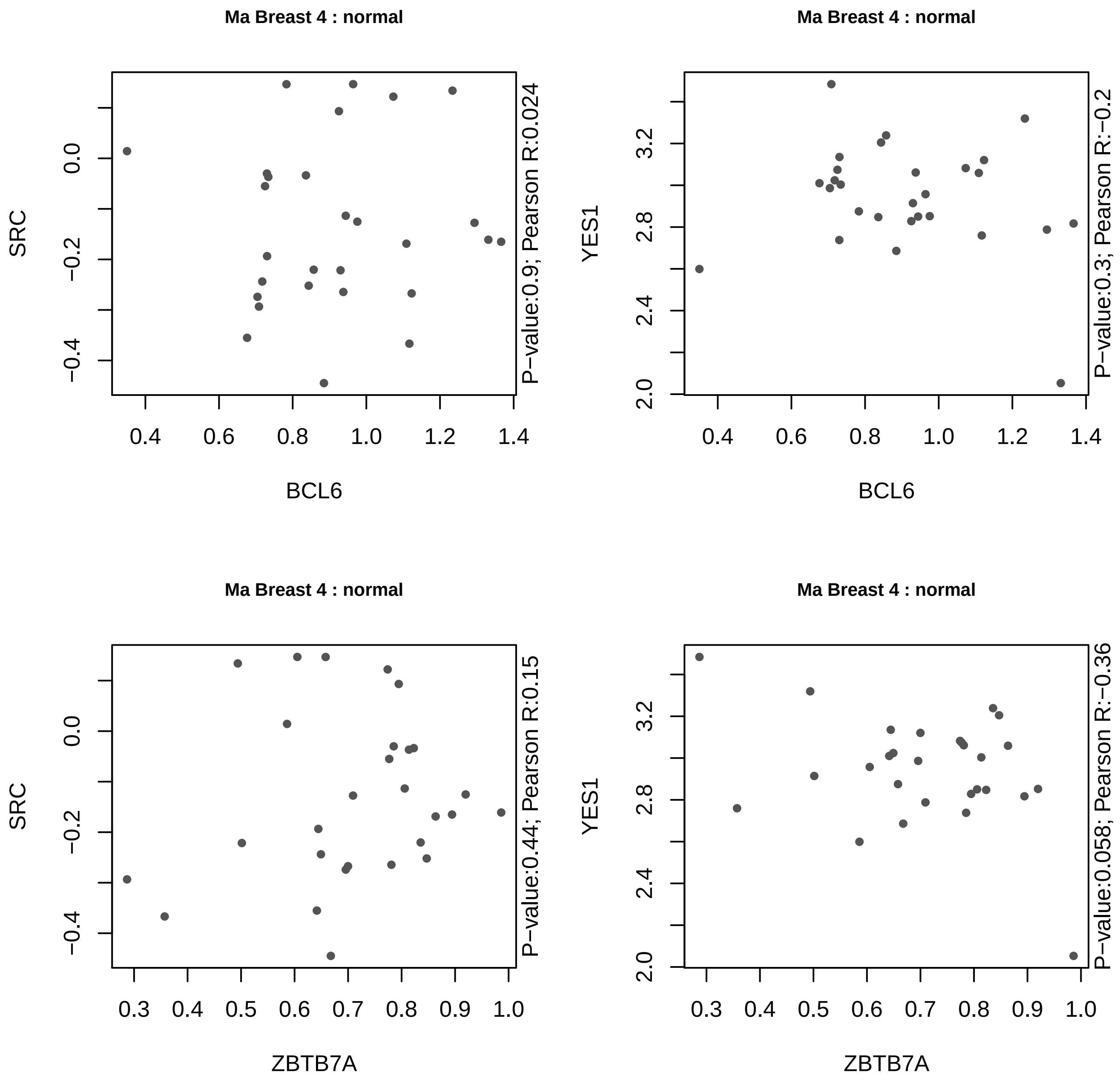
Ma Breast 4 : normal
SRC
BCL6
P−value:0.9; Pearson R:0.024
Ma Breast 4 : normal
YES1
BCL6
P−value:0.3; Pearson R:−0.2
Ma Breast 4 : normal
SRC
ZBTB7A
P−value:0.44; Pearson R:0.15
Ma Breast 4 : normal
YES1
ZBTB7A
P−value:0.058; Pearson R:−0.36

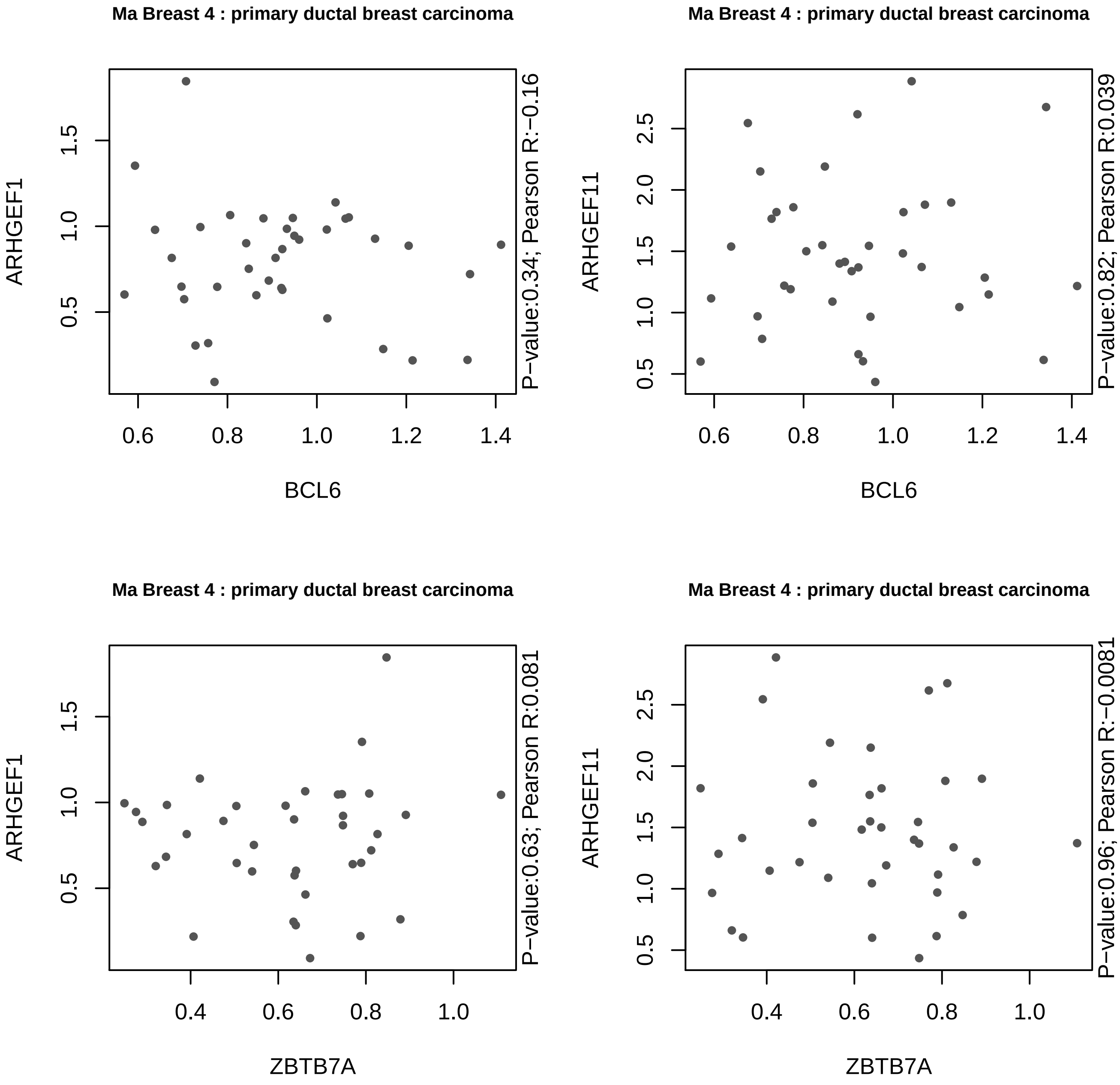
Ma Breast 4 : primary ductal breast carcinoma
ARHGEF1
BCL6
P−value:0.34; Pearson R:−0.16
Ma Breast 4 : primary ductal breast carcinoma
ARHGEF11
BCL6
P−value:0.82; Pearson R:0.039
Ma Breast 4 : primary ductal breast carcinoma
ARHGEF1
ZBTB7A
P−value:0.63; Pearson R:0.081
Ma Breast 4 : primary ductal breast carcinoma
ARHGEF11
ZBTB7A
P−value:0.96; Pearson R:−0.0081

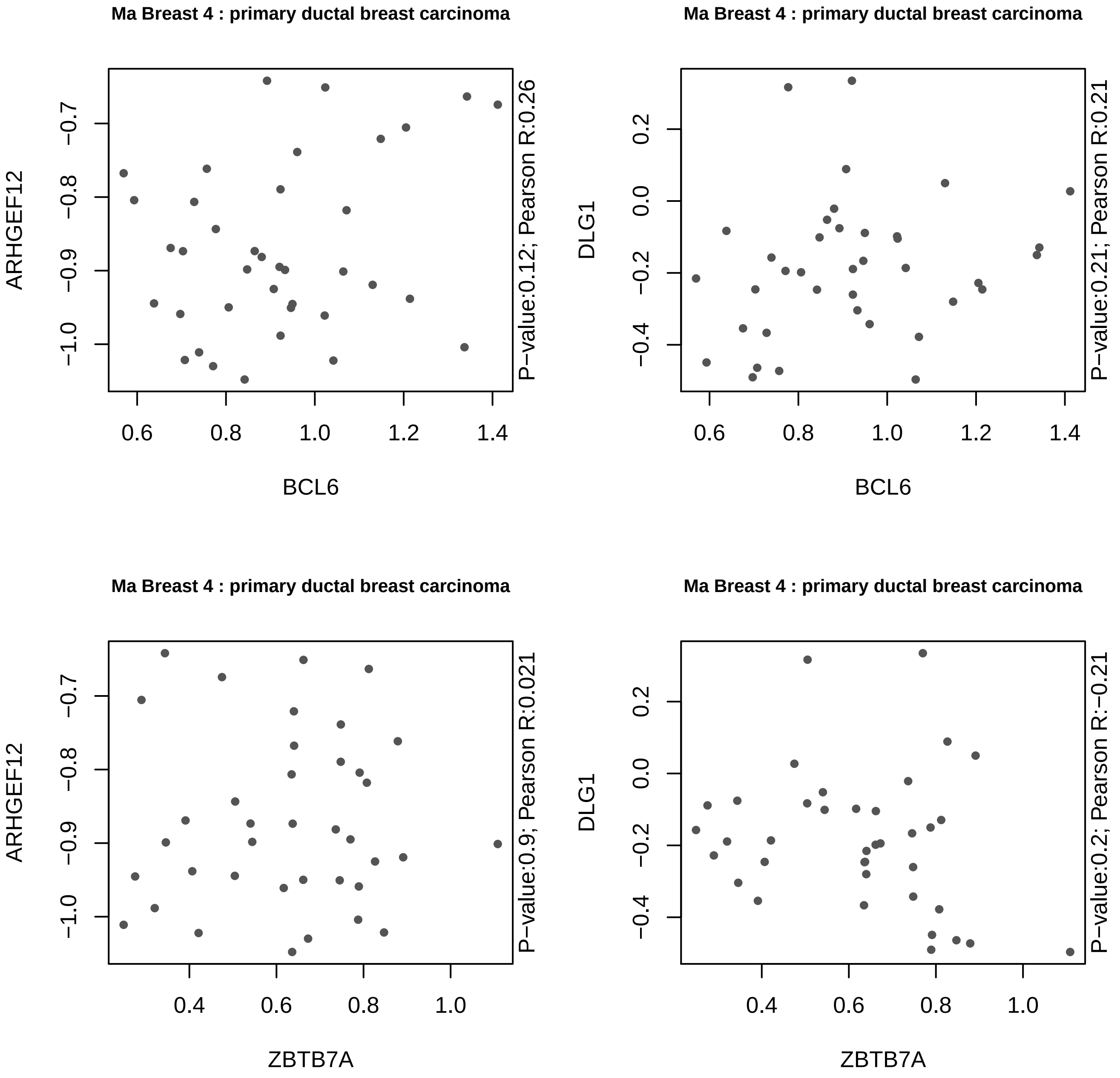
Ma Breast 4 : primary ductal breast carcinoma
ARHGEF12
BCL6
P−value:0.12; Pearson R:0.26
Ma Breast 4 : primary ductal breast carcinoma
DLG1
BCL6
P−value:0.21; Pearson R:0.21
Ma Breast 4 : primary ductal breast carcinoma
ARHGEF12
ZBTB7A
P−value:0.9; Pearson R:0.021
Ma Breast 4 : primary ductal breast carcinoma
DLG1
ZBTB7A
P−value:0.2; Pearson R:−0.21

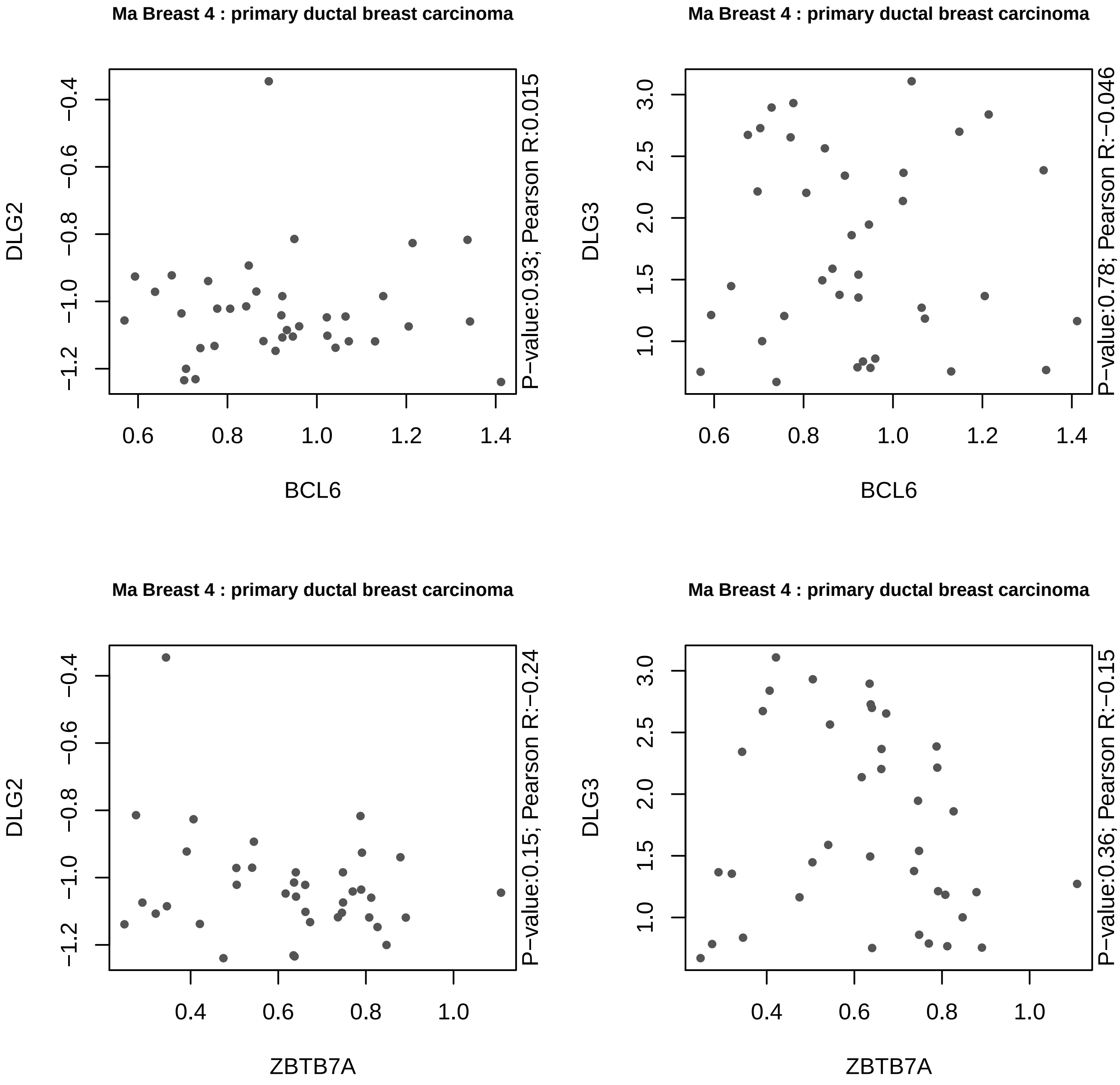
Ma Breast 4 : primary ductal breast carcinoma
DLG2
BCL6
P−value:0.93; Pearson R:0.015
Ma Breast 4 : primary ductal breast carcinoma
DLG3
BCL6
P−value:0.78; Pearson R:−0.046
Ma Breast 4 : primary ductal breast carcinoma
DLG2
ZBTB7A
P−value:0.15; Pearson R:−0.24
Ma Breast 4 : primary ductal breast carcinoma
DLG3
ZBTB7A
P−value:0.36; Pearson R:−0.15

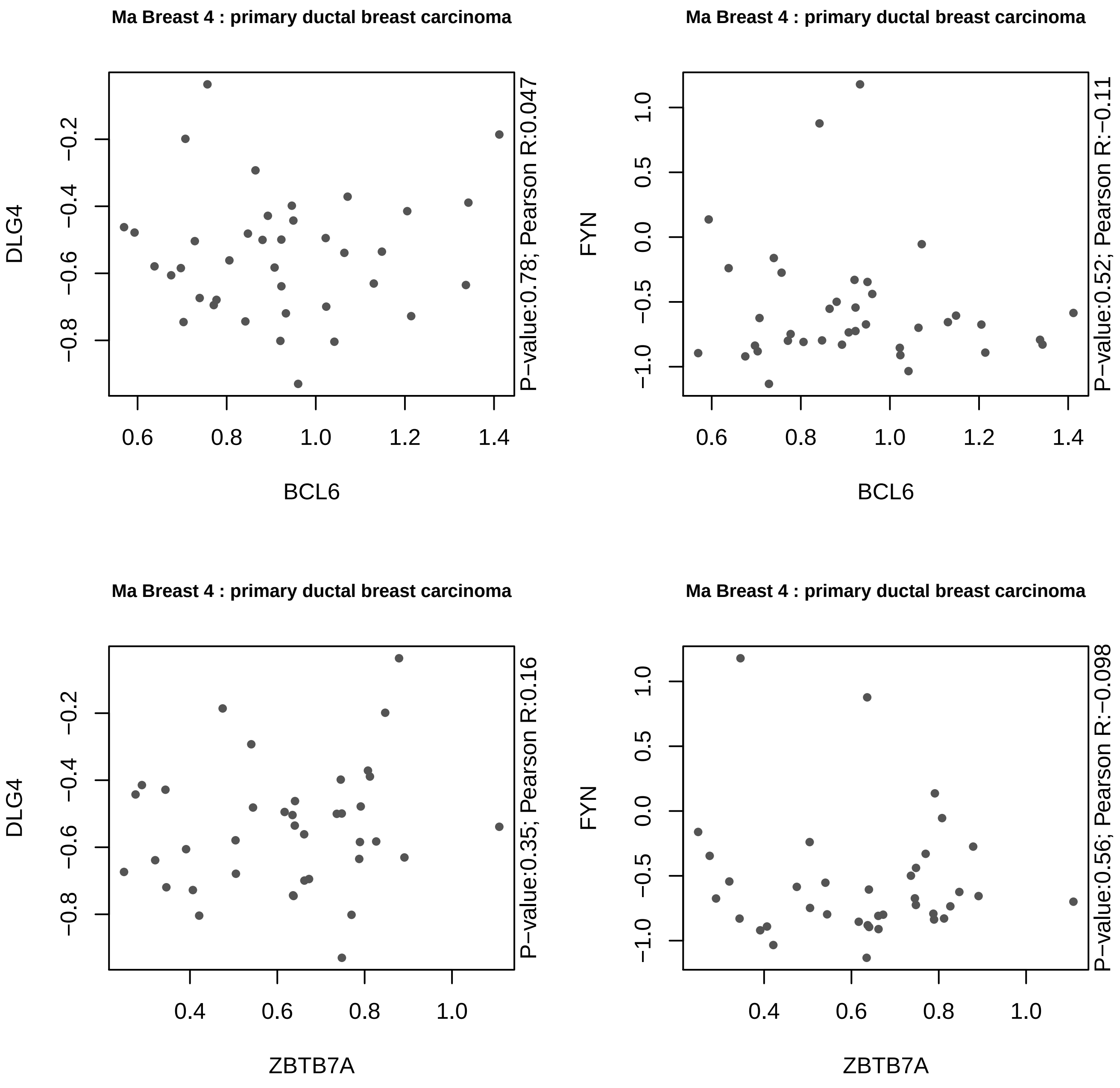
Ma Breast 4 : primary ductal breast carcinoma
DLG4
BCL6
P−value:0.78; Pearson R:0.047
Ma Breast 4 : primary ductal breast carcinoma
FYN
BCL6
P−value:0.52; Pearson R:−0.11
Ma Breast 4 : primary ductal breast carcinoma
DLG4
ZBTB7A
P−value:0.35; Pearson R:0.16
Ma Breast 4 : primary ductal breast carcinoma
FYN
ZBTB7A
P−value:0.56; Pearson R:−0.098

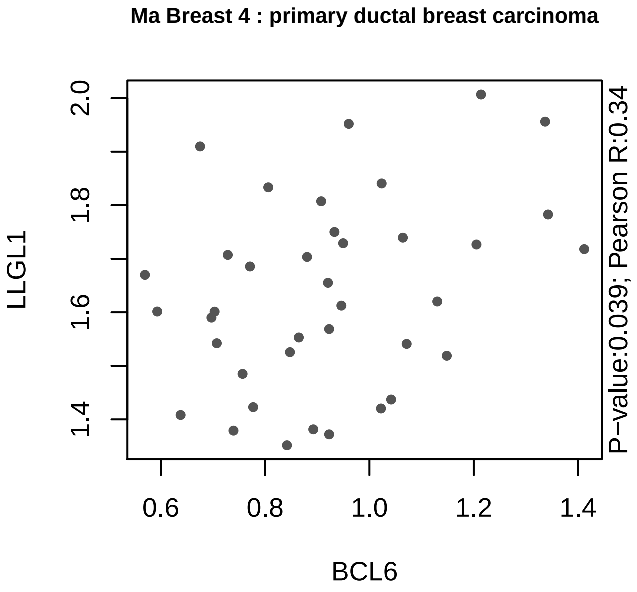
Ma Breast 4 : primary ductal breast carcinoma
LLGL1
BCL6
P−value:0.039; Pearson R:0.34

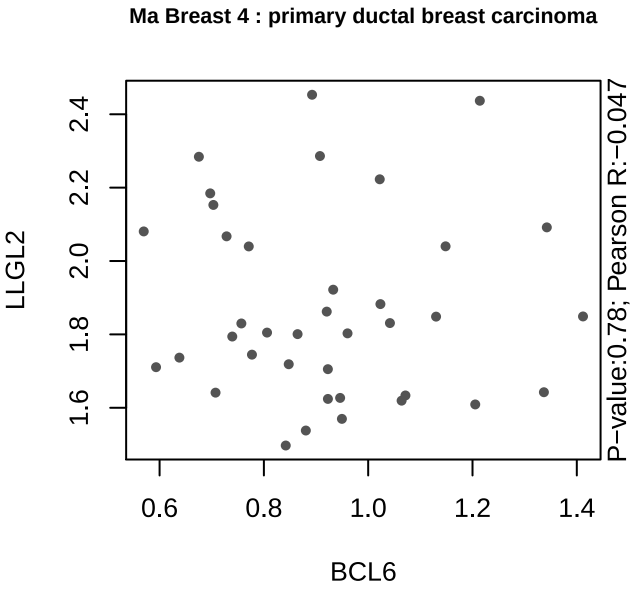
Ma Breast 4 : primary ductal breast carcinoma
LLGL2
BCL6
P−value:0.78; Pearson R:−0.047

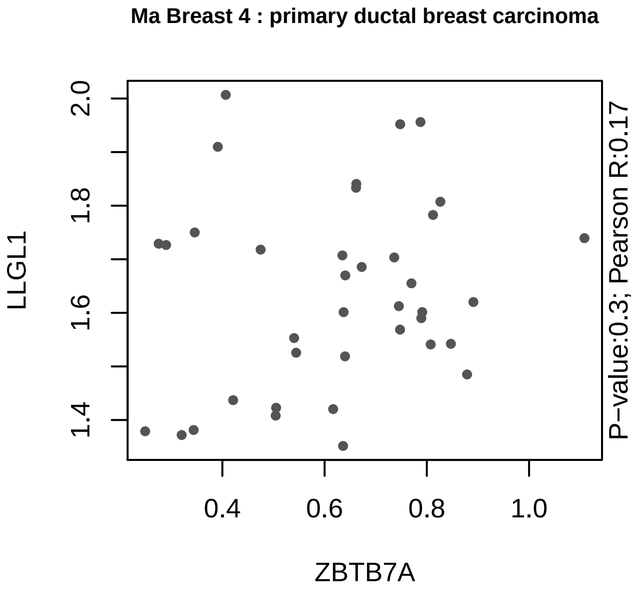
Ma Breast 4 : primary ductal breast carcinoma
LLGL1
ZBTB7A
P−value:0.3; Pearson R:0.17

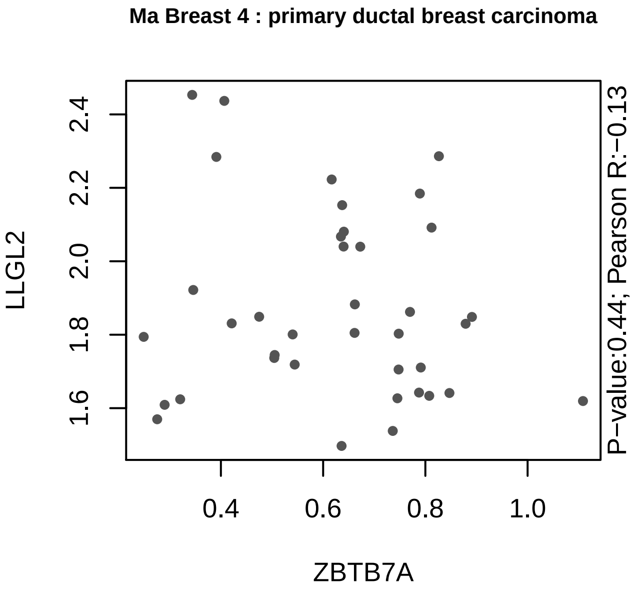
Ma Breast 4 : primary ductal breast carcinoma
LLGL2
ZBTB7A
P−value:0.44; Pearson R:−0.13

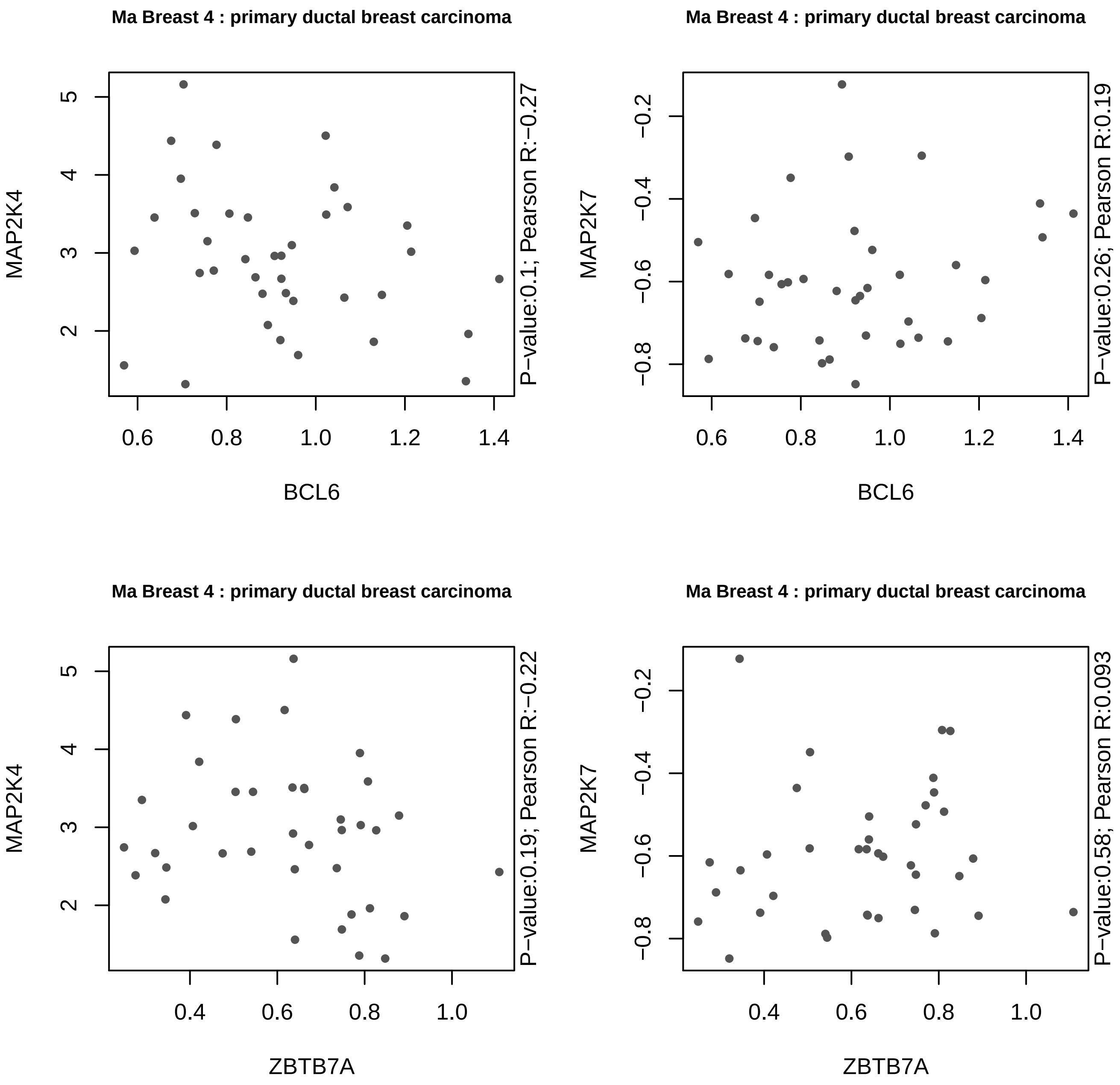

Ma Breast 4 : primary ductal breast carcinoma
MAP2K4
BCL6
P−value:0.1; Pearson R:−0.27
Ma Breast 4 : primary ductal breast carcinoma
MAP2K7
BCL6
P−value:0.26; Pearson R:0.19
Ma Breast 4 : primary ductal breast carcinoma
MAP2K4
ZBTB7A
P−value:0.19; Pearson R:−0.22
Ma Breast 4 : primary ductal breast carcinoma
MAP2K7
ZBTB7A
P−value:0.58; Pearson R:0.093

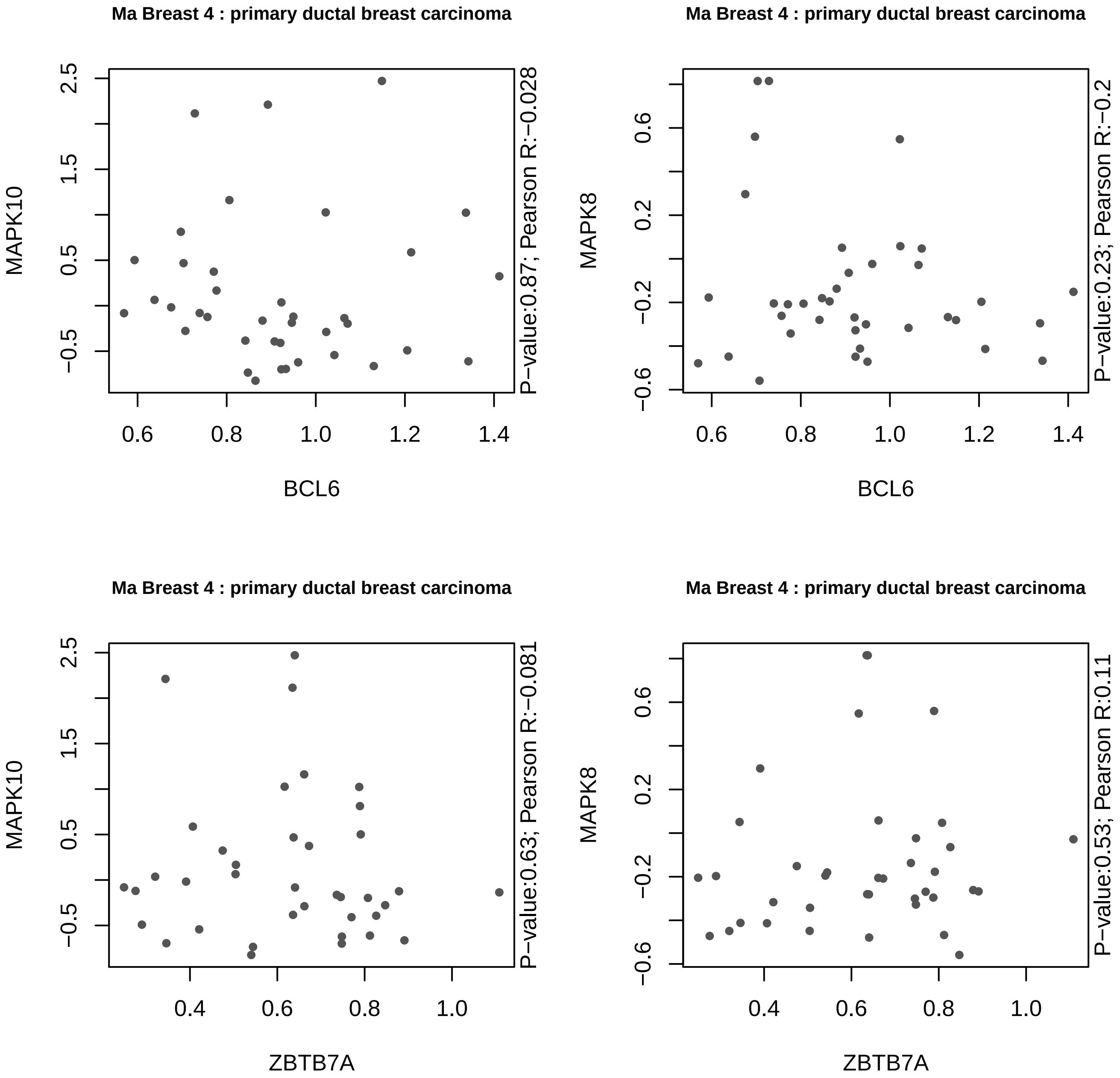
Ma Breast 4 : primary ductal breast carcinoma
MAPK10
BCL6
P−value:0.87; Pearson R:−0.028
Ma Breast 4 : primary ductal breast carcinoma
MAPK8
BCL6
P−value:0.23; Pearson R:−0.2
Ma Breast 4 : primary ductal breast carcinoma
MAPK10
ZBTB7A
P−value:0.63; Pearson R:−0.081
Ma Breast 4 : primary ductal breast carcinoma
MAPK8
ZBTB7A
P−value:0.53; Pearson R:0.11

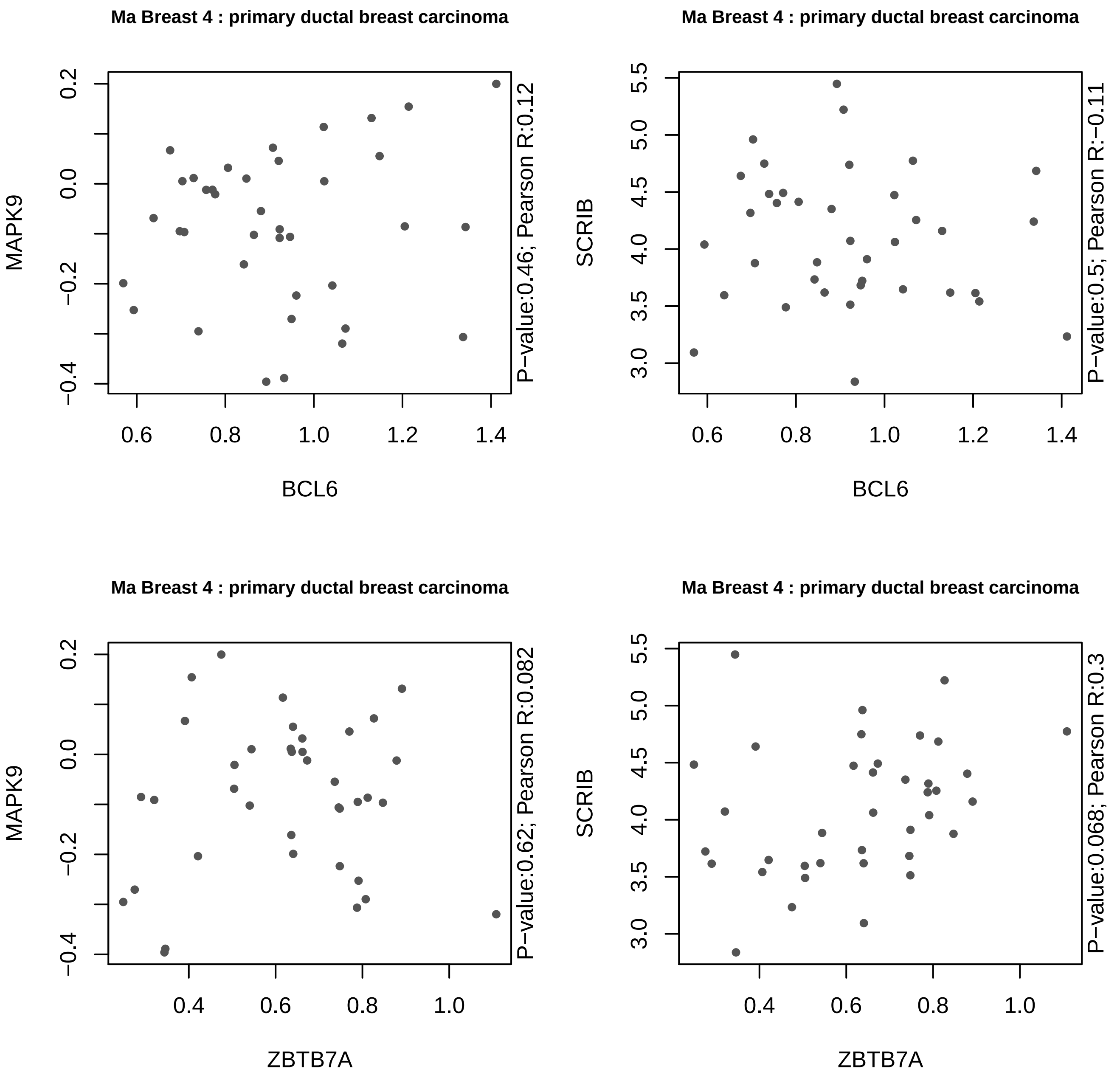
Ma Breast 4 : primary ductal breast carcinoma
MAPK9
BCL6
P−value:0.46; Pearson R:0.12
Ma Breast 4 : primary ductal breast carcinoma
SCRIB
BCL6
P−value:0.5; Pearson R:−0.11
Ma Breast 4 : primary ductal breast carcinoma
MAPK9
ZBTB7A
P−value:0.62; Pearson R:0.082
Ma Breast 4 : primary ductal breast carcinoma
SCRIB
ZBTB7A
P−value:0.068; Pearson R:0.3

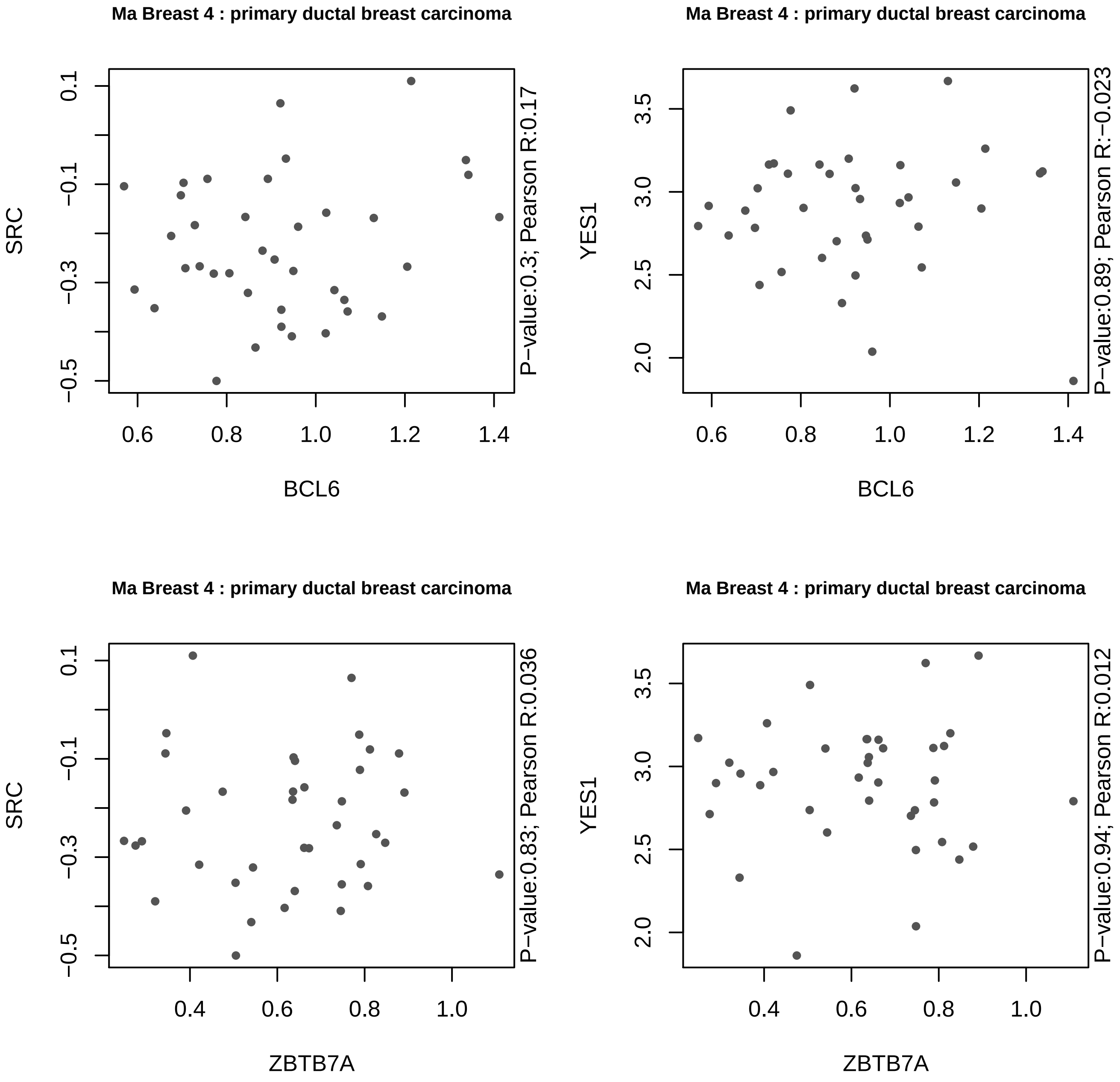
Ma Breast 4 : primary ductal breast carcinoma
SRC
BCL6
P−value:0.3; Pearson R:0.17
Ma Breast 4 : primary ductal breast carcinoma
YES1
BCL6
P−value:0.89; Pearson R:−0.023
Ma Breast 4 : primary ductal breast carcinoma
SRC
ZBTB7A
P−value:0.83; Pearson R:0.036
Ma Breast 4 : primary ductal breast carcinoma
YES1
ZBTB7A
P−value:0.94; Pearson R:0.012

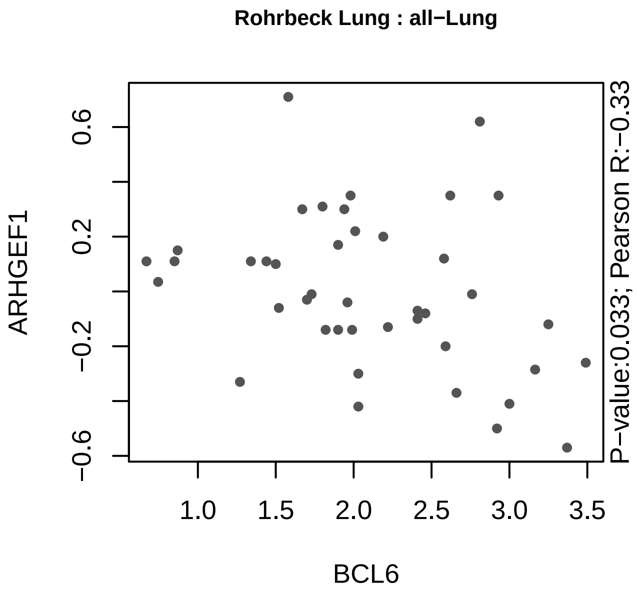
Rohrbeck Lung : all−Lung
ARHGEF1
BCL6
P−value:0.033; Pearson R:−0.33

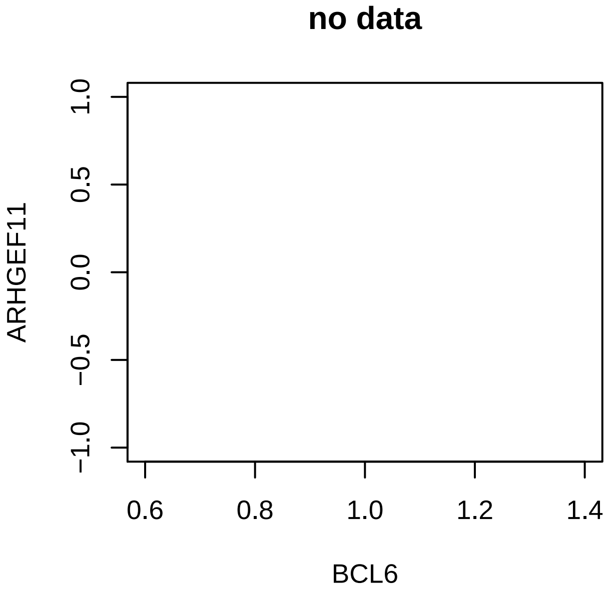
no data
ARHGEF11
BCL6

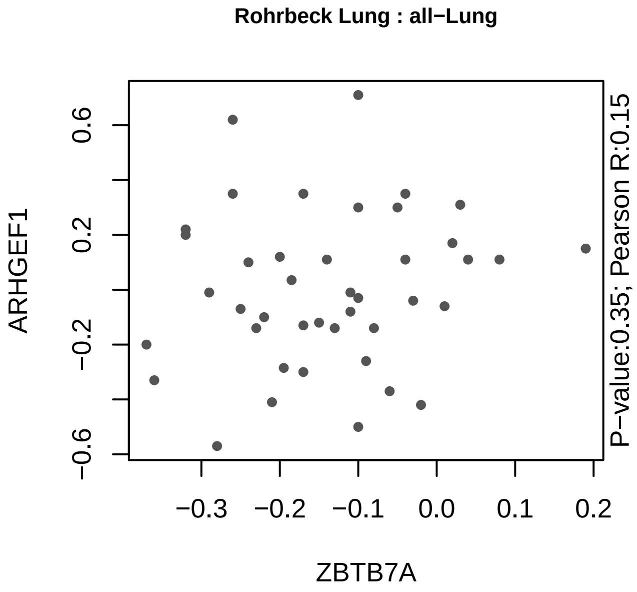
Rohrbeck Lung : all−Lung
ARHGEF1
ZBTB7A
P−value:0.35; Pearson R:0.15

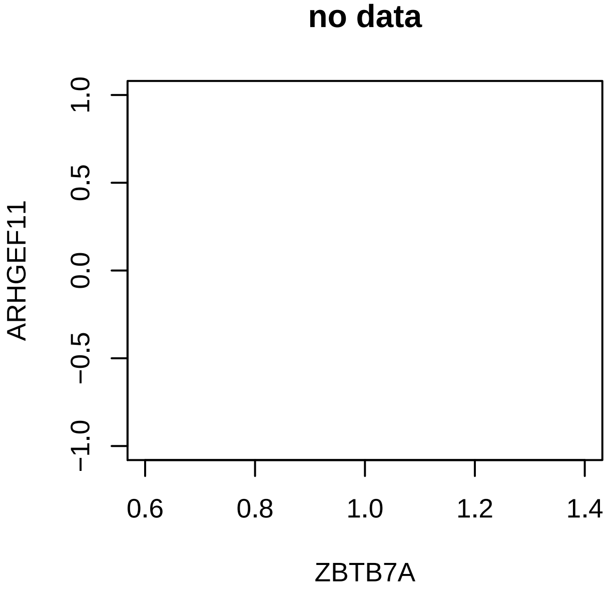
no data
ARHGEF11
ZBTB7A

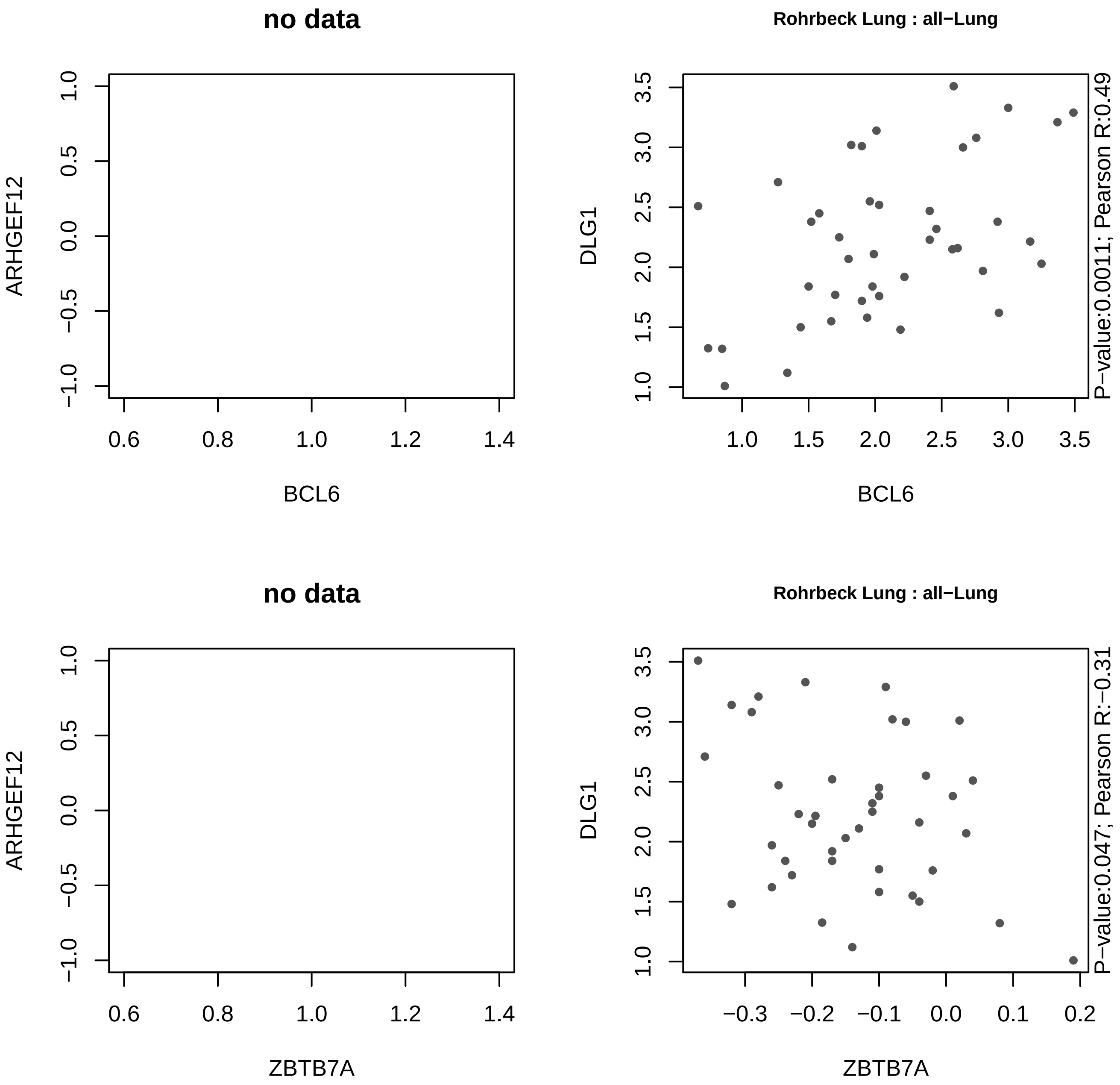
no data
ARHGEF12
BCL6
Rohrbeck Lung : all−Lung
DLG1
BCL6
P−value:0.0011; Pearson R:0.49
no data
ARHGEF12
ZBTB7A
Rohrbeck Lung : all−Lung
DLG1
ZBTB7A
P−value:0.047; Pearson R:−0.31

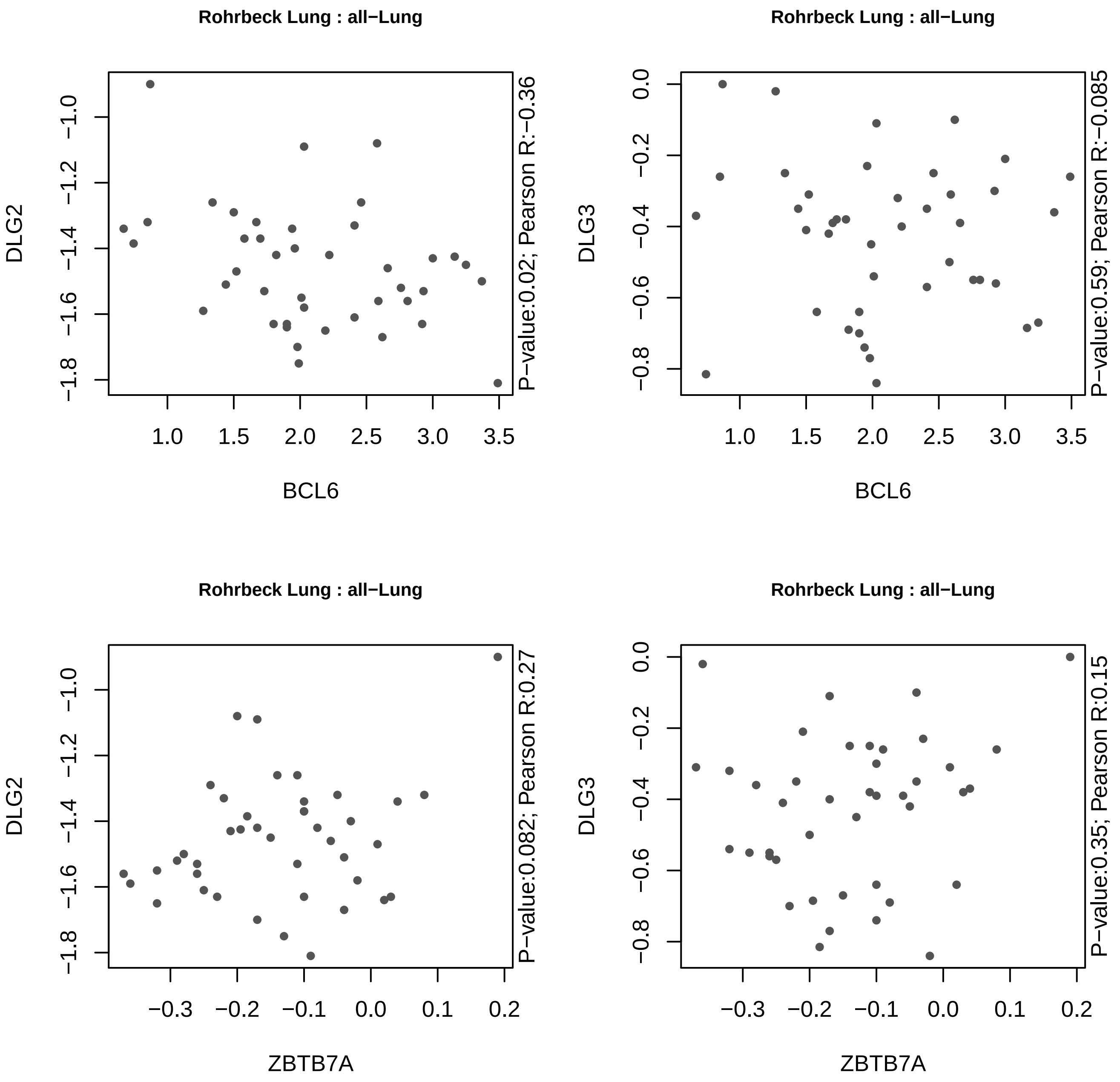
Rohrbeck Lung : all−Lung
DLG2
BCL6
P−value:0.02; Pearson R:−0.36
Rohrbeck Lung : all−Lung
DLG3
BCL6
P−value:0.59; Pearson R:−0.085
Rohrbeck Lung : all−Lung
DLG2
ZBTB7A
P−value:0.082; Pearson R:0.27
Rohrbeck Lung : all−Lung
DLG3
ZBTB7A
P−value:0.35; Pearson R:0.15

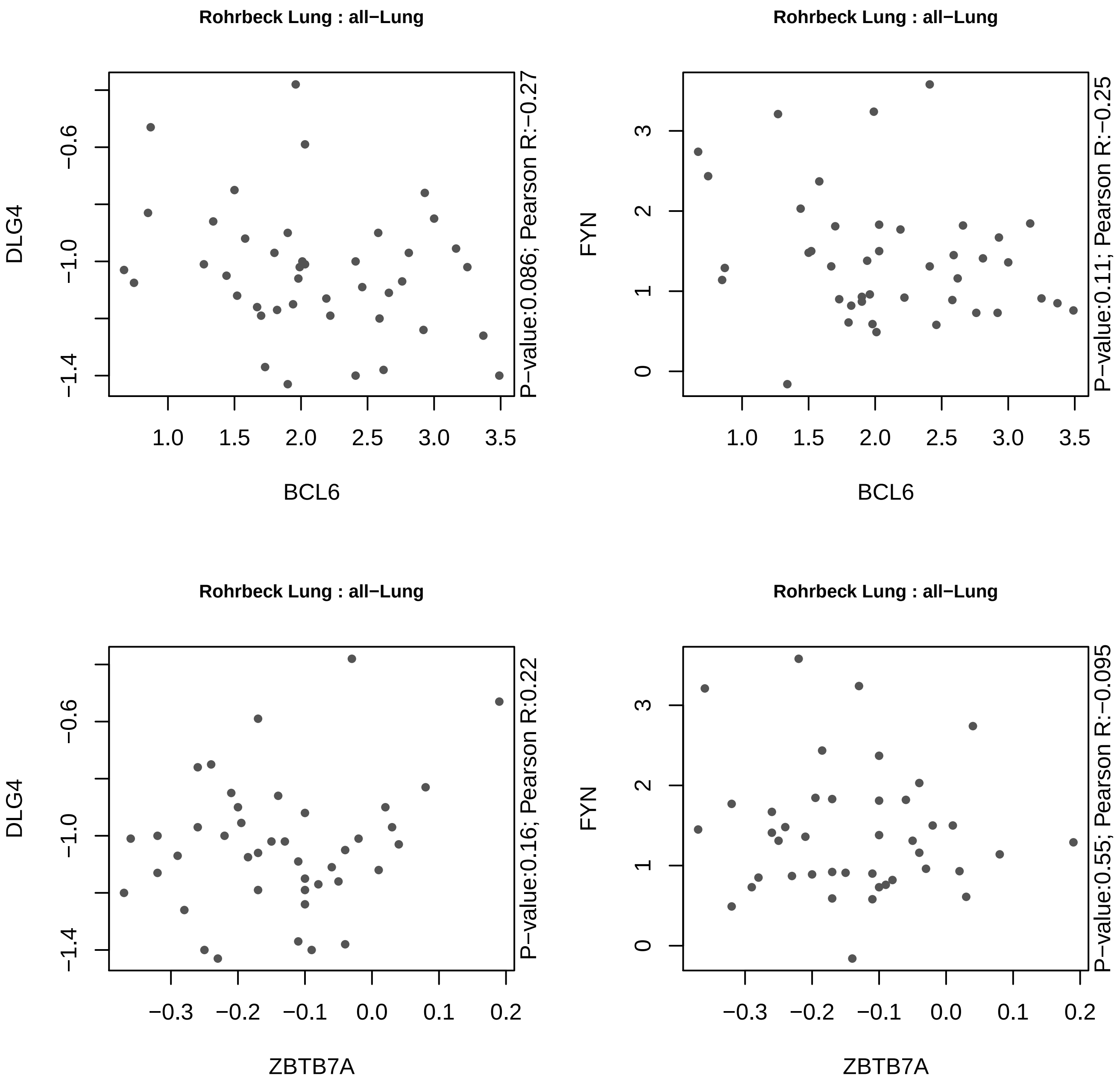
Rohrbeck Lung : all−Lung
DLG4
BCL6
P−value:0.086; Pearson R:−0.27
Rohrbeck Lung : all−Lung
FYN
BCL6
P−value:0.11; Pearson R:−0.25
Rohrbeck Lung : all−Lung
DLG4
ZBTB7A
P−value:0.16; Pearson R:0.22
Rohrbeck Lung : all−Lung
FYN
ZBTB7A
P−value:0.55; Pearson R:−0.095

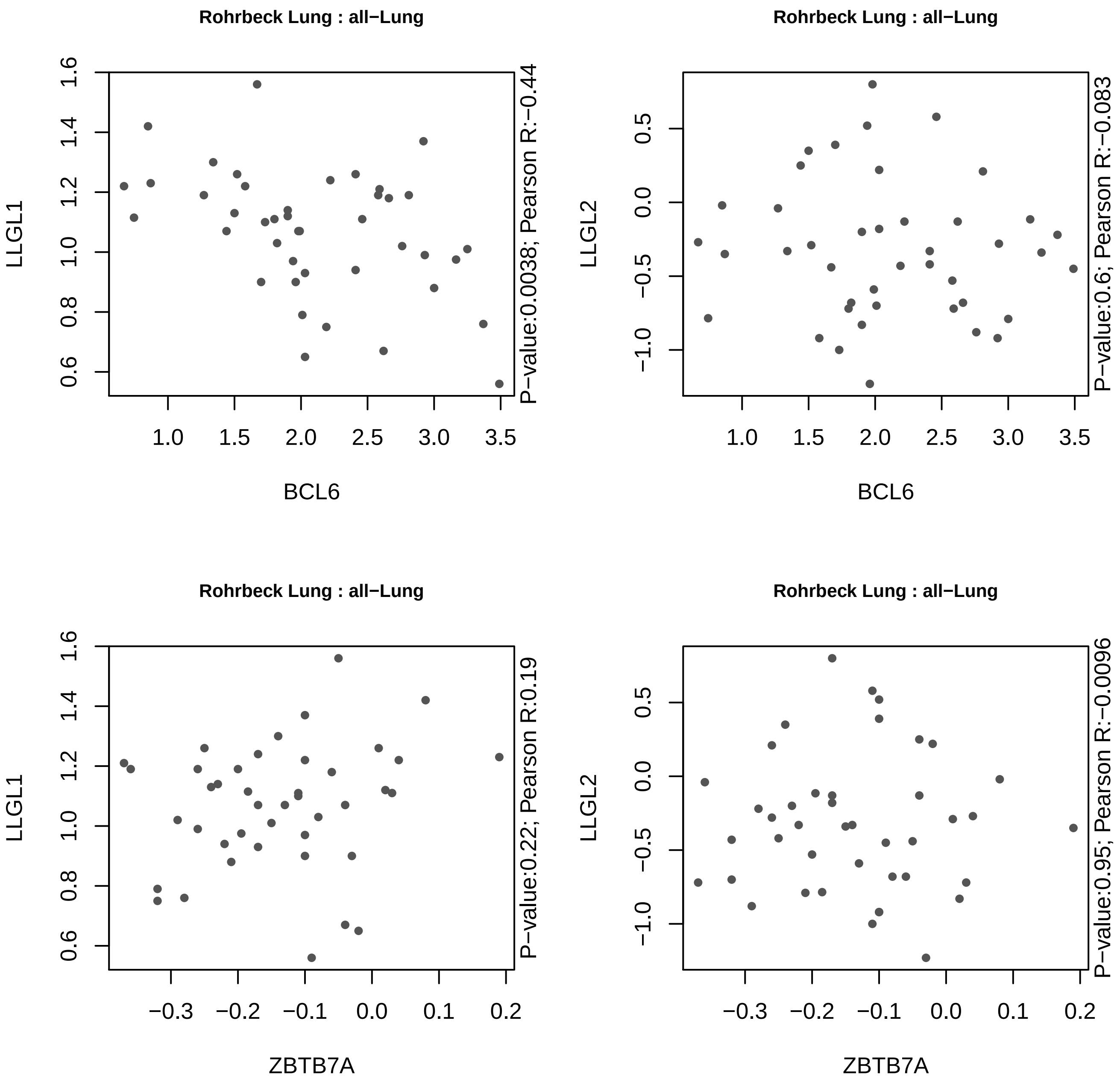
Rohrbeck Lung : all−Lung
LLGL1
BCL6
P−value:0.0038; Pearson R:−0.44
Rohrbeck Lung : all−Lung
LLGL2
BCL6
P−value:0.6; Pearson R:−0.083
Rohrbeck Lung : all−Lung
LLGL1
ZBTB7A
P−value:0.22; Pearson R:0.19
Rohrbeck Lung : all−Lung
LLGL2
ZBTB7A
P−value:0.95; Pearson R:−0.0096

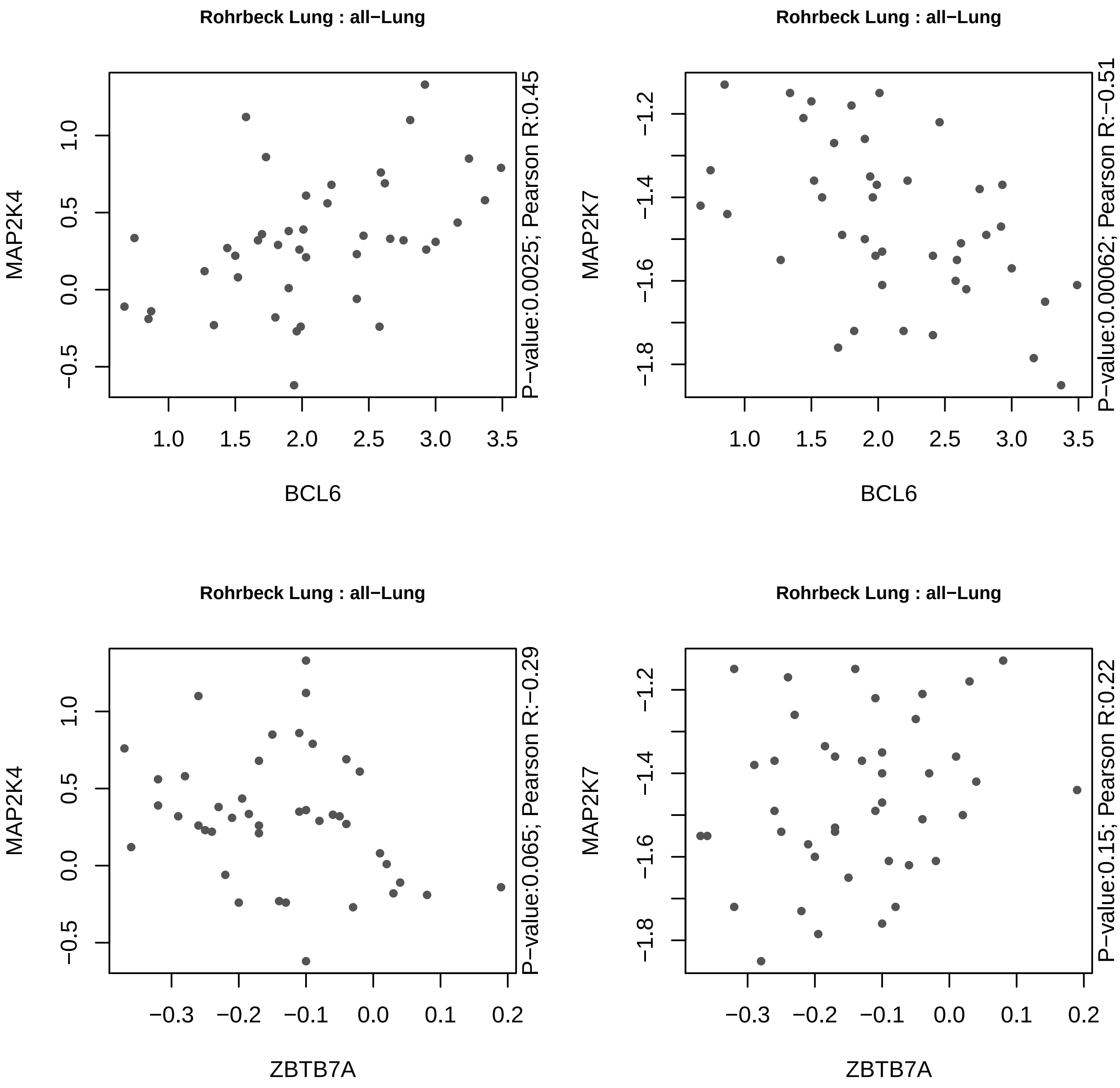
Rohrbeck Lung : all−Lung
MAP2K4
BCL6
P−value:0.0025; Pearson R:0.45
Rohrbeck Lung : all−Lung
MAP2K7
BCL6
P−value:0.00062; Pearson R:−0.51
Rohrbeck Lung : all−Lung
MAP2K4
ZBTB7A
P−value:0.065; Pearson R:−0.29
Rohrbeck Lung : all−Lung
MAP2K7
ZBTB7A
P−value:0.15; Pearson R:0.22

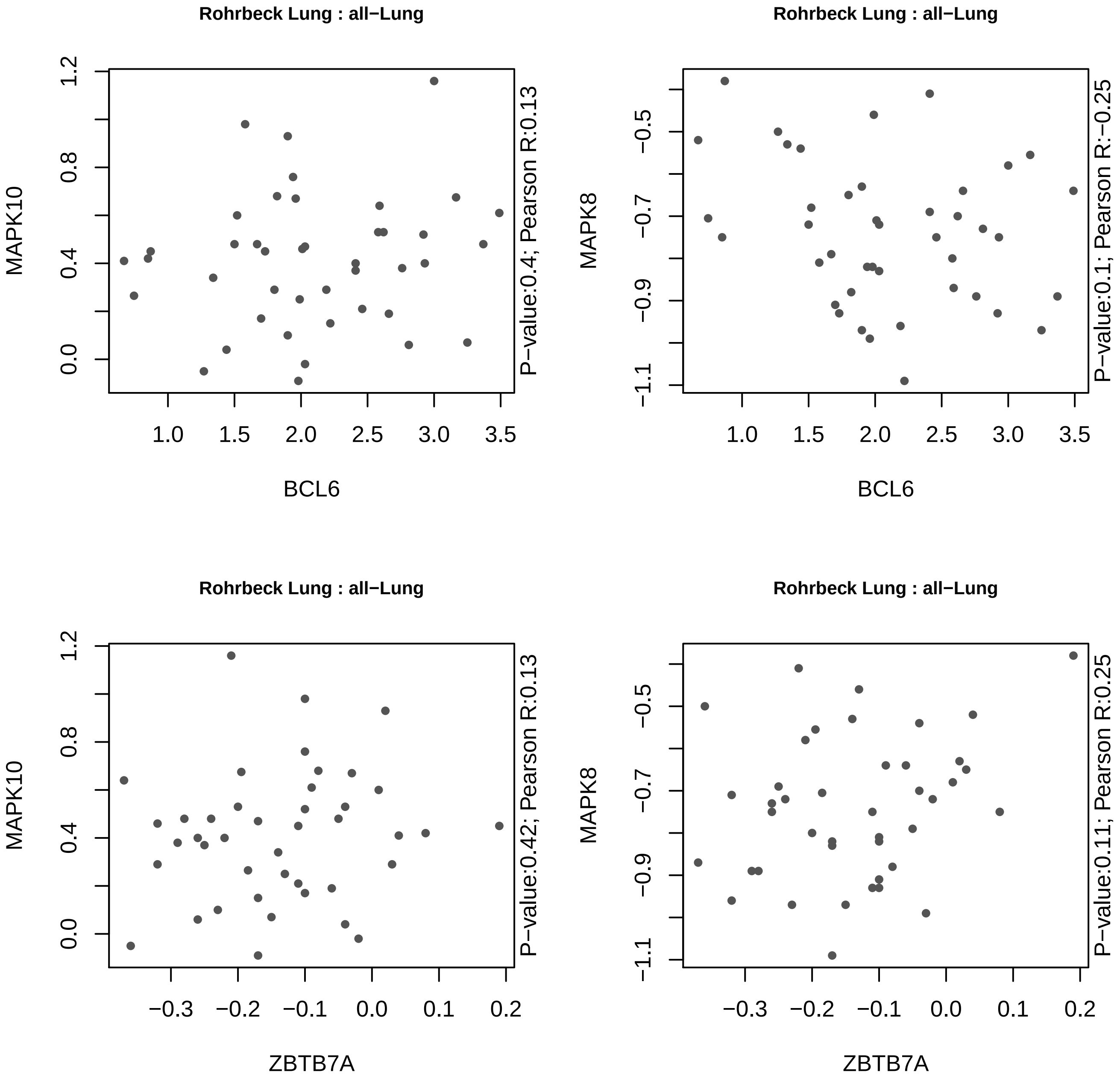
Rohrbeck Lung : all−Lung
MAPK10
BCL6
P−value:0.4; Pearson R:0.13
Rohrbeck Lung : all−Lung
MAPK8
BCL6
P−value:0.1; Pearson R:−0.25
Rohrbeck Lung : all−Lung
MAPK10
ZBTB7A
P−value:0.42; Pearson R:0.13
Rohrbeck Lung : all−Lung
MAPK8
ZBTB7A
P−value:0.11; Pearson R:0.25

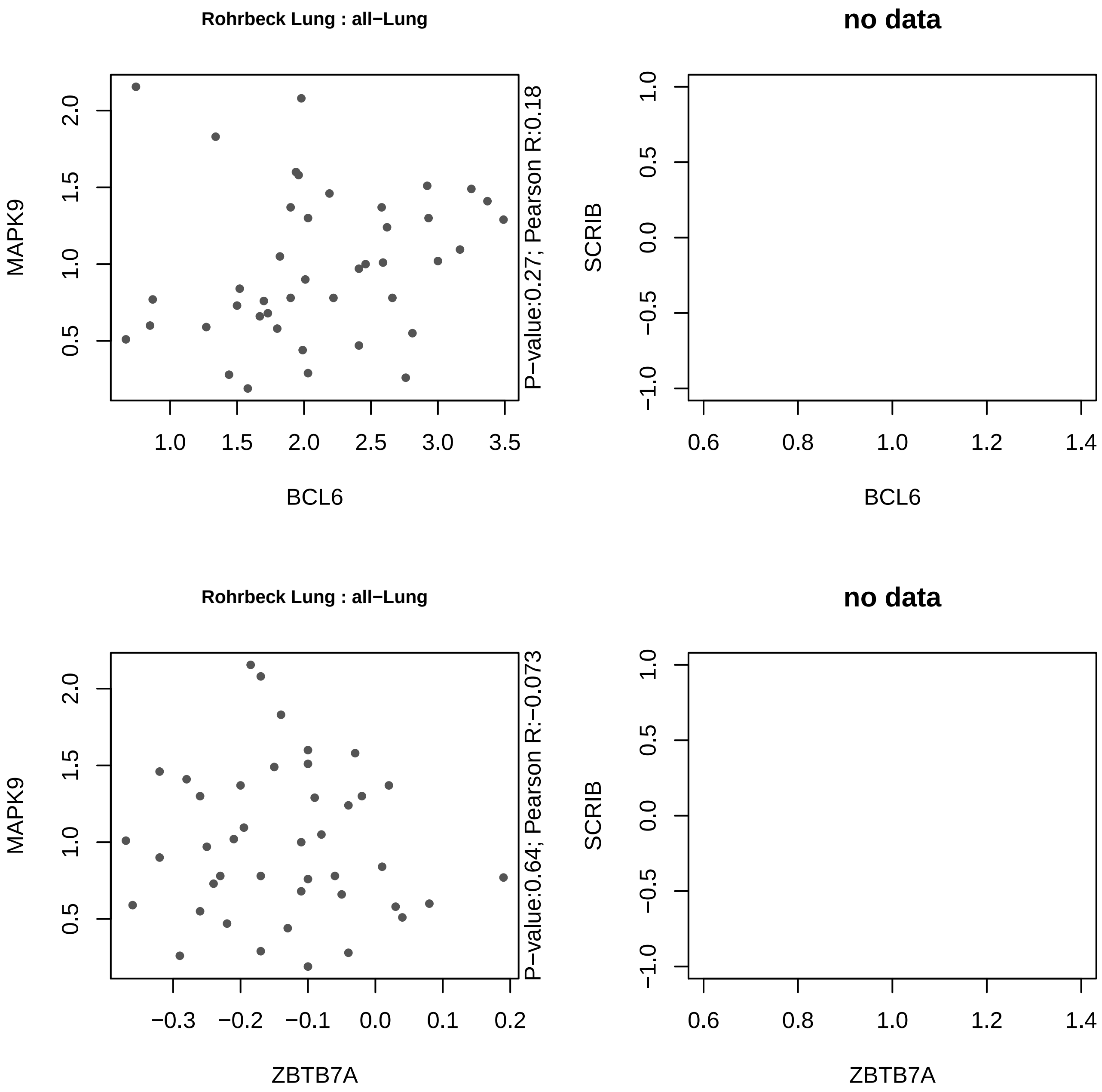
Rohrbeck Lung : all−Lung
MAPK9
BCL6
P−value:0.27; Pearson R:0.18
no data
SCRIB
BCL6
Rohrbeck Lung : all−Lung
MAPK9
ZBTB7A
P−value:0.64; Pearson R:−0.073
no data
SCRIB
ZBTB7A

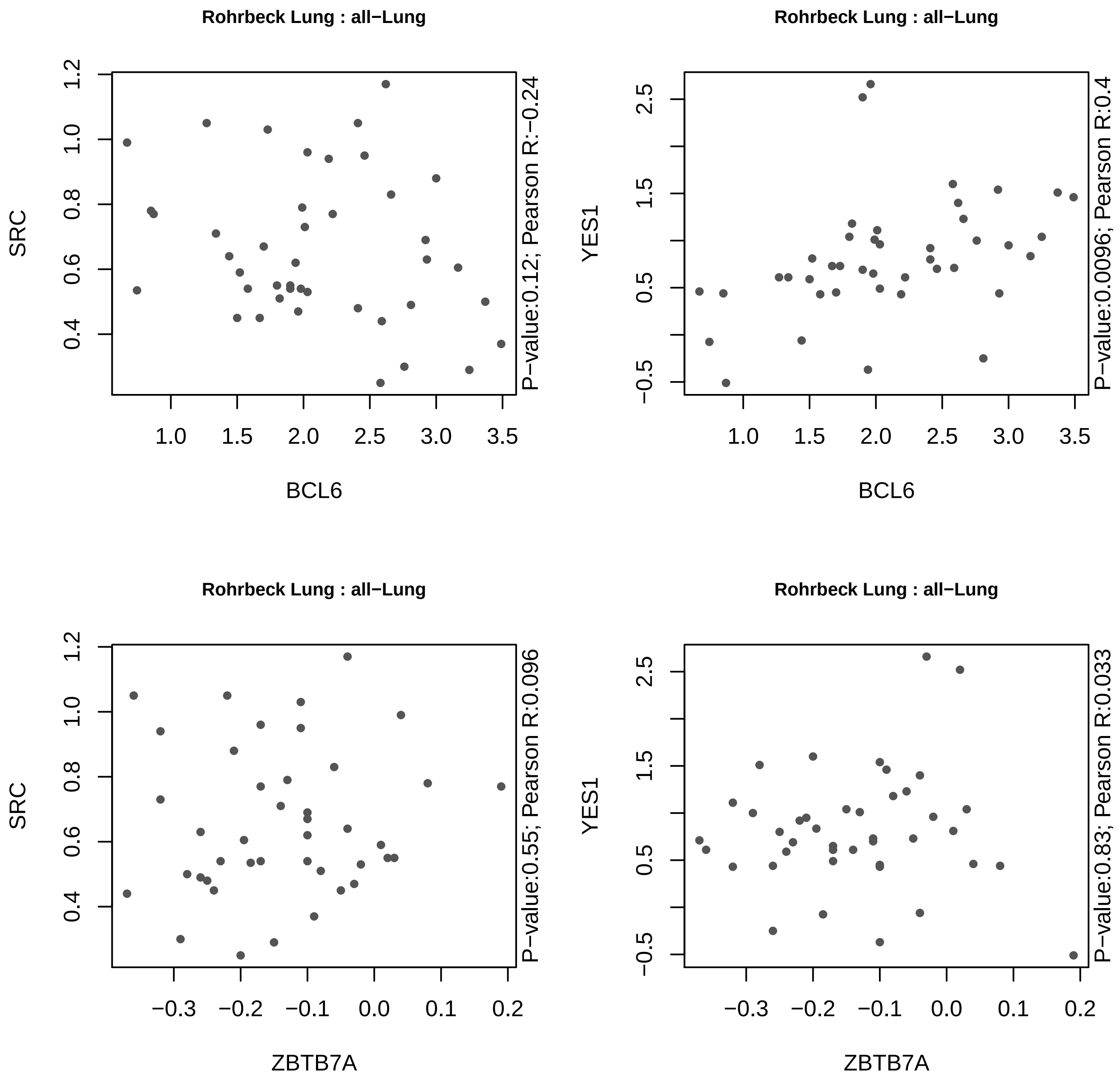
Rohrbeck Lung : all−Lung
SRC
BCL6
P−value:0.12; Pearson R:−0.24
Rohrbeck Lung : all−Lung
YES1
BCL6
P−value:0.0096; Pearson R:0.4
Rohrbeck Lung : all−Lung
SRC
ZBTB7A
P−value:0.55; Pearson R:0.096
Rohrbeck Lung : all−Lung
YES1
ZBTB7A
P−value:0.83; Pearson R:0.033

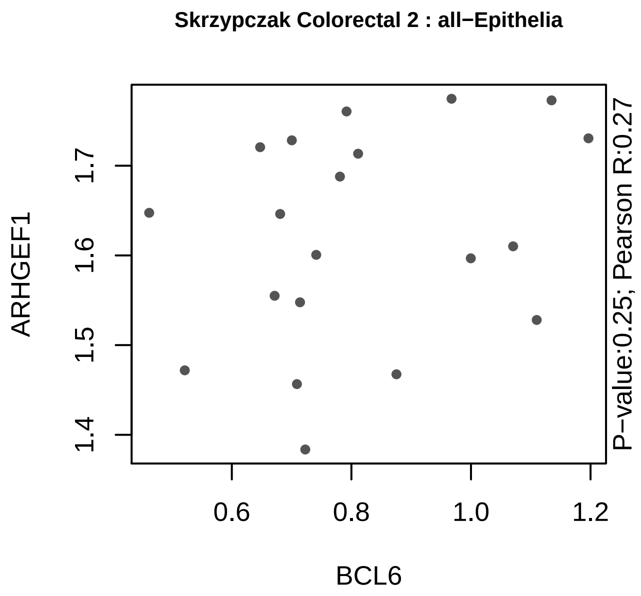
Skrzypczak Colorectal 2 : all−Epithelia
ARHGEF1
BCL6
P−value:0.25; Pearson R:0.27
1.4
1.5
1.6
1.7
0.6
0.8
1.0
1.2

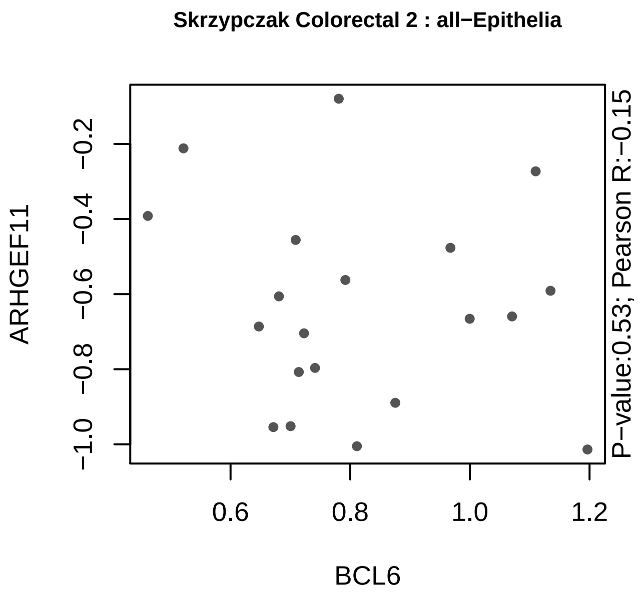
Skrzypczak Colorectal 2 : all−Epithelia
ARHGEF11
BCL6
P−value:0.53; Pearson R:−0.15
−1.0
−0.8
−0.6
−0.4
−0.2
0.6
0.8
1.0
1.2

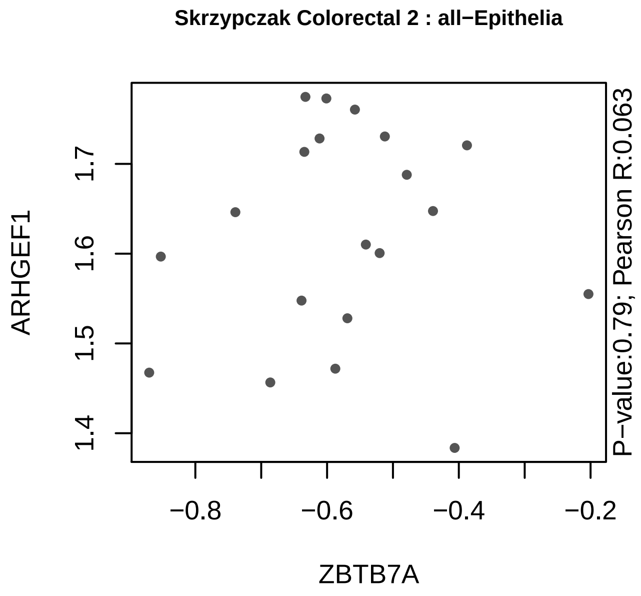
Skrzypczak Colorectal 2 : all−Epithelia
ARHGEF1
ZBTB7A
P−value:0.79; Pearson R:0.063
1.4
1.5
1.6
1.7
−0.8
−0.6
−0.4
−0.2

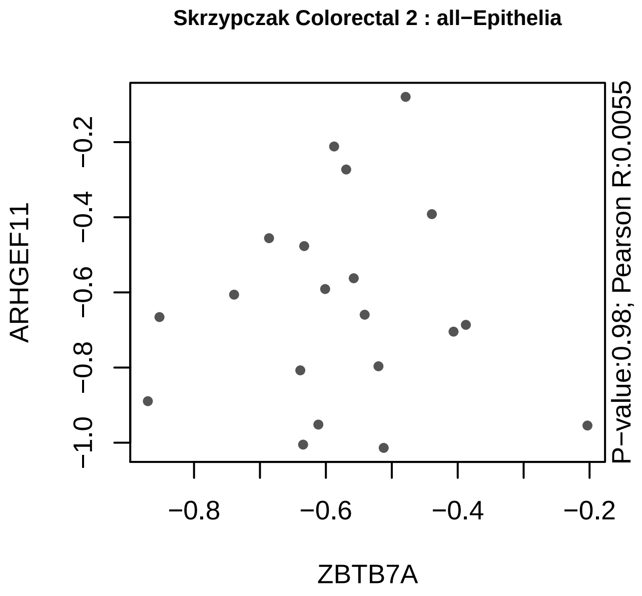
Skrzypczak Colorectal 2 : all−Epithelia
ARHGEF11
ZBTB7A
P−value:0.98; Pearson R:0.0055
−1.0
−0.8
−0.6
−0.4
−0.2
−0.8
−0.6
−0.4
−0.2

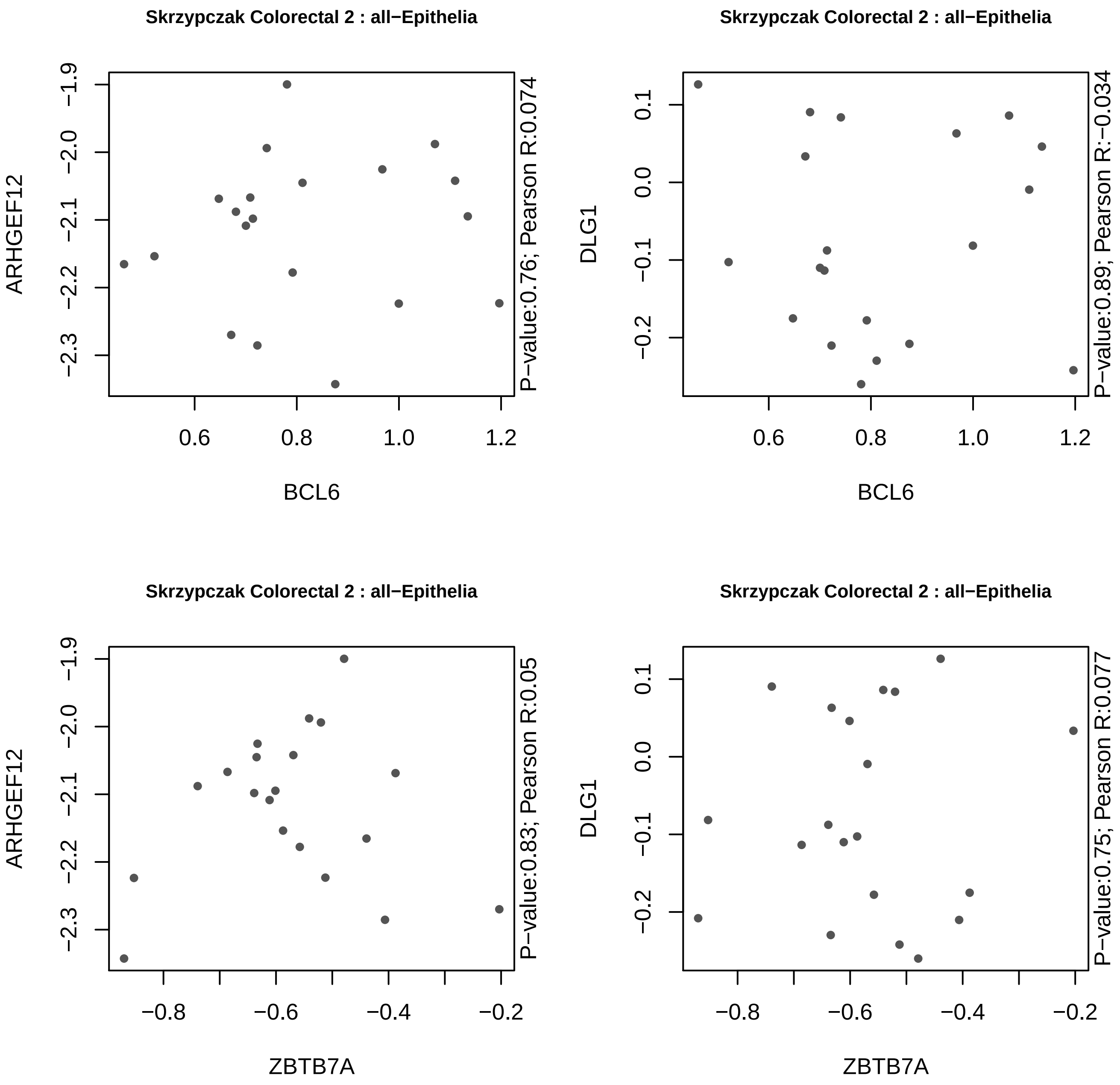
Skrzypczak Colorectal 2 : all−Epithelia
ARHGEF12
BCL6
P−value:0.76; Pearson R:0.074
Skrzypczak Colorectal 2 : all−Epithelia
DLG1
BCL6
P−value:0.89; Pearson R:−0.034
Skrzypczak Colorectal 2 : all−Epithelia
ARHGEF12
ZBTB7A
P−value:0.83; Pearson R:0.05
Skrzypczak Colorectal 2 : all−Epithelia
DLG1
ZBTB7A
P−value:0.75; Pearson R:0.077

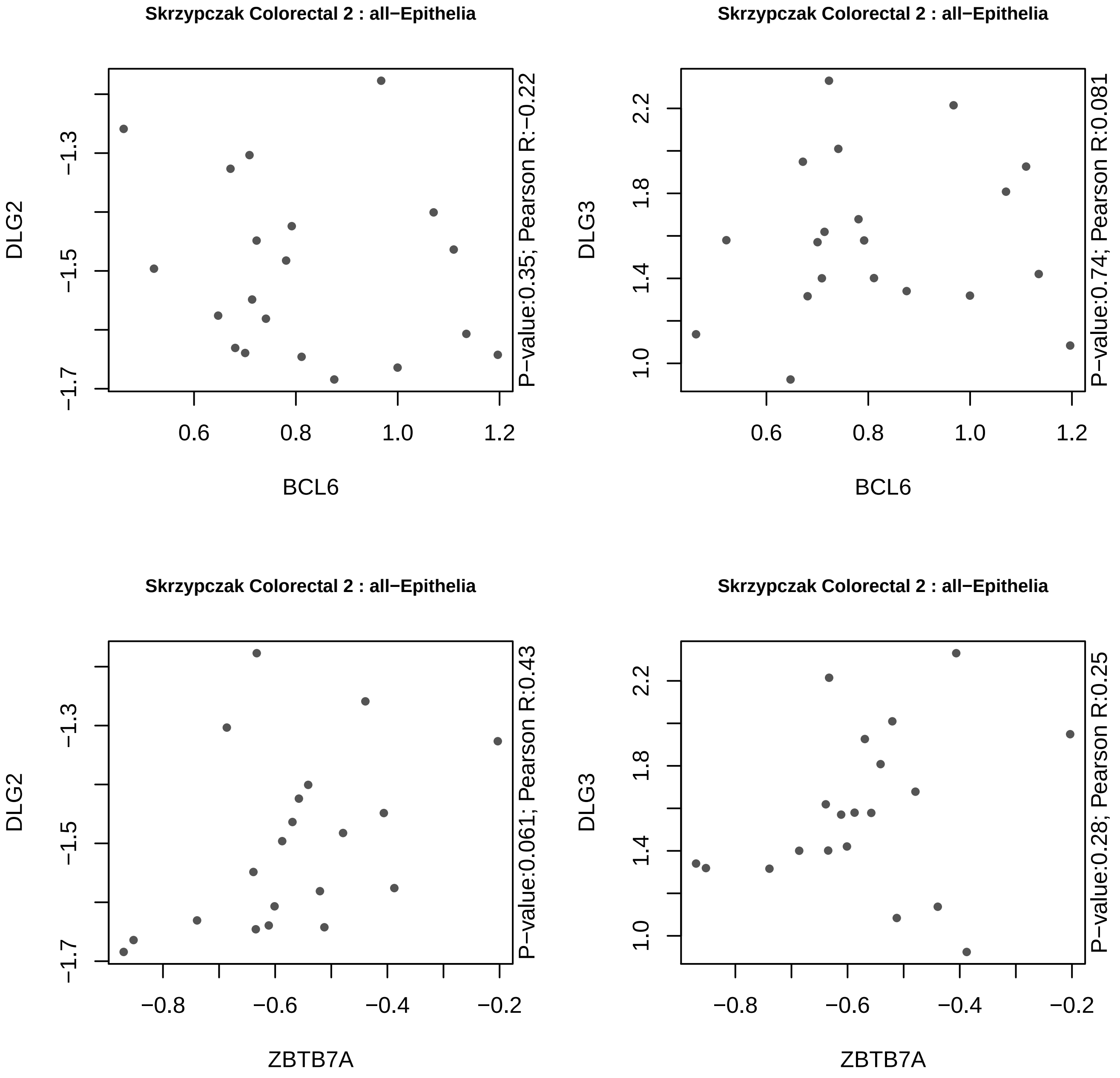
Skrzypczak Colorectal 2 : all−Epithelia
DLG2
−1.3
−1.5
−1.7
BCL6
0.6
0.8
1.0
1.2
P−value:0.35; Pearson R:−0.22
Skrzypczak Colorectal 2 : all−Epithelia
DLG3
2.2
1.8
1.4
1.0
BCL6
0.6
0.8
1.0
1.2
P−value:0.74; Pearson R:0.081
Skrzypczak Colorectal 2 : all−Epithelia
DLG2
−1.3
−1.5
−1.7
ZBTB7A
−0.8
−0.6
−0.4
−0.2
P−value:0.061; Pearson R:0.43
Skrzypczak Colorectal 2 : all−Epithelia
DLG3
2.2
1.8
1.4
1.0
ZBTB7A
−0.8
−0.6
−0.4
−0.2
P−value:0.28; Pearson R:0.25

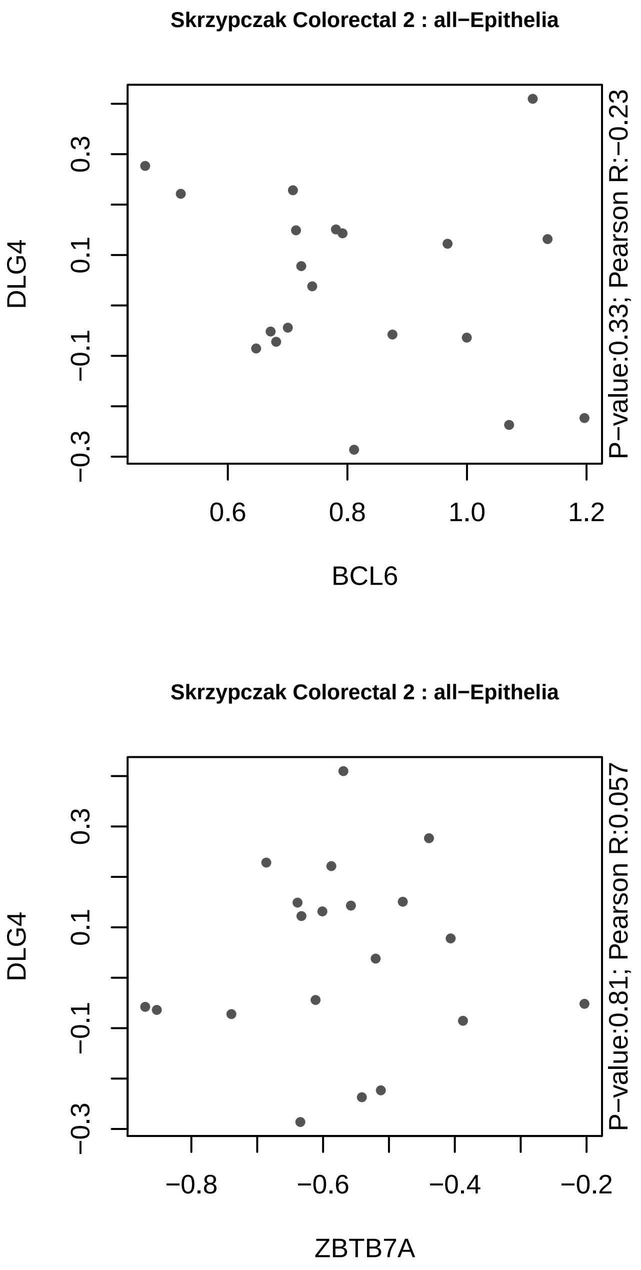
Skrzypczak Colorectal 2 : all−Epithelia
DLG4
BCL6
P−value:0.33; Pearson R:−0.23
Skrzypczak Colorectal 2 : all−Epithelia
FYN
BCL6
P−value:0.12; Pearson R:−0.36
Skrzypczak Colorectal 2 : all−Epithelia
DLG4
ZBTB7A
P−value:0.81; Pearson R:0.057
Skrzypczak Colorectal 2 : all−Epithelia
FYN
ZBTB7A
P−value:0.062; Pearson R:0.42

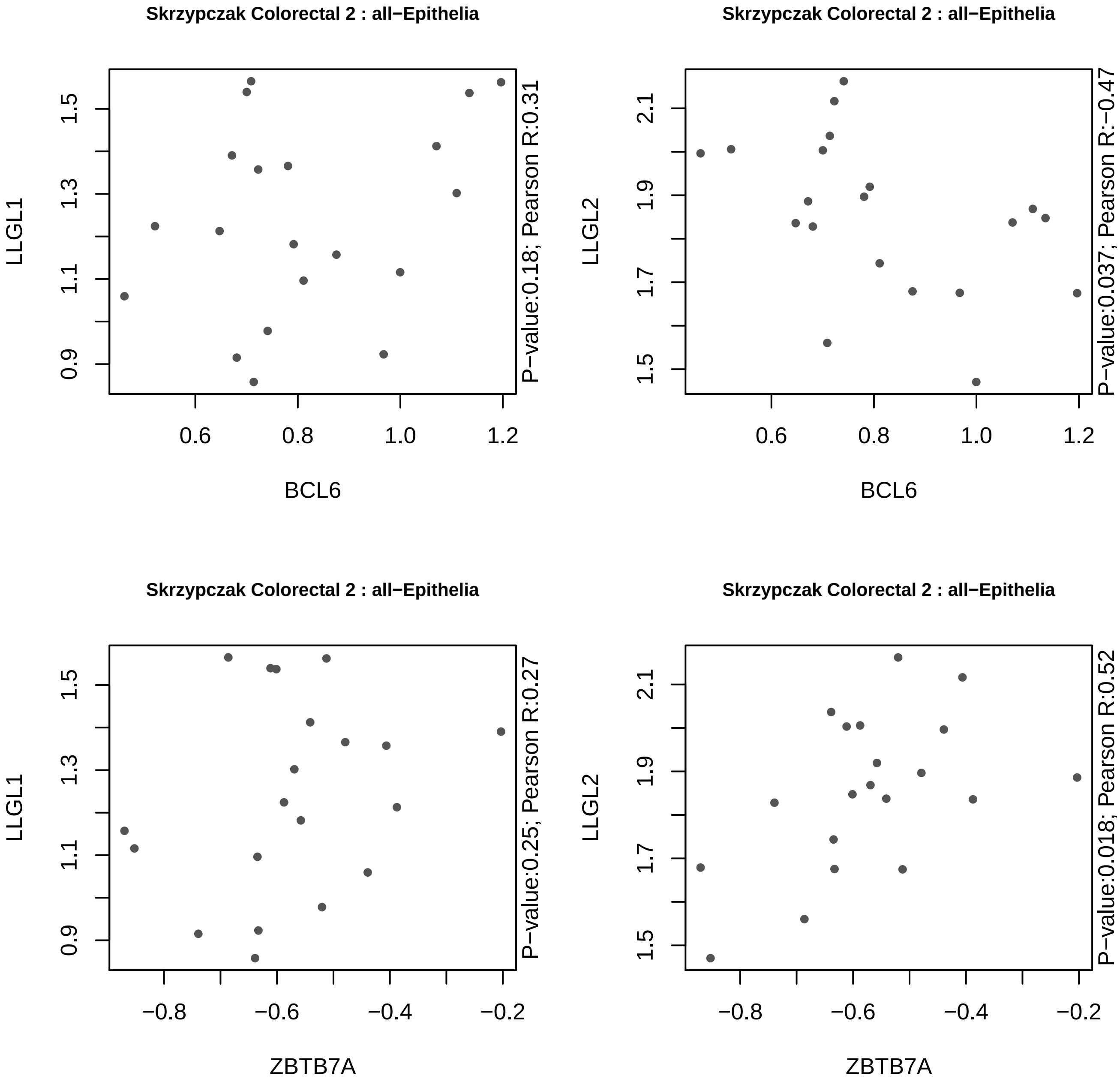
Skrzypczak Colorectal 2 : all−Epithelia
LLGL1
BCL6
P−value:0.18; Pearson R:0.31
Skrzypczak Colorectal 2 : all−Epithelia
LLGL2
BCL6
P−value:0.037; Pearson R:−0.47
Skrzypczak Colorectal 2 : all−Epithelia
LLGL1
ZBTB7A
P−value:0.25; Pearson R:0.27
Skrzypczak Colorectal 2 : all−Epithelia
LLGL2
ZBTB7A
P−value:0.018; Pearson R:0.52

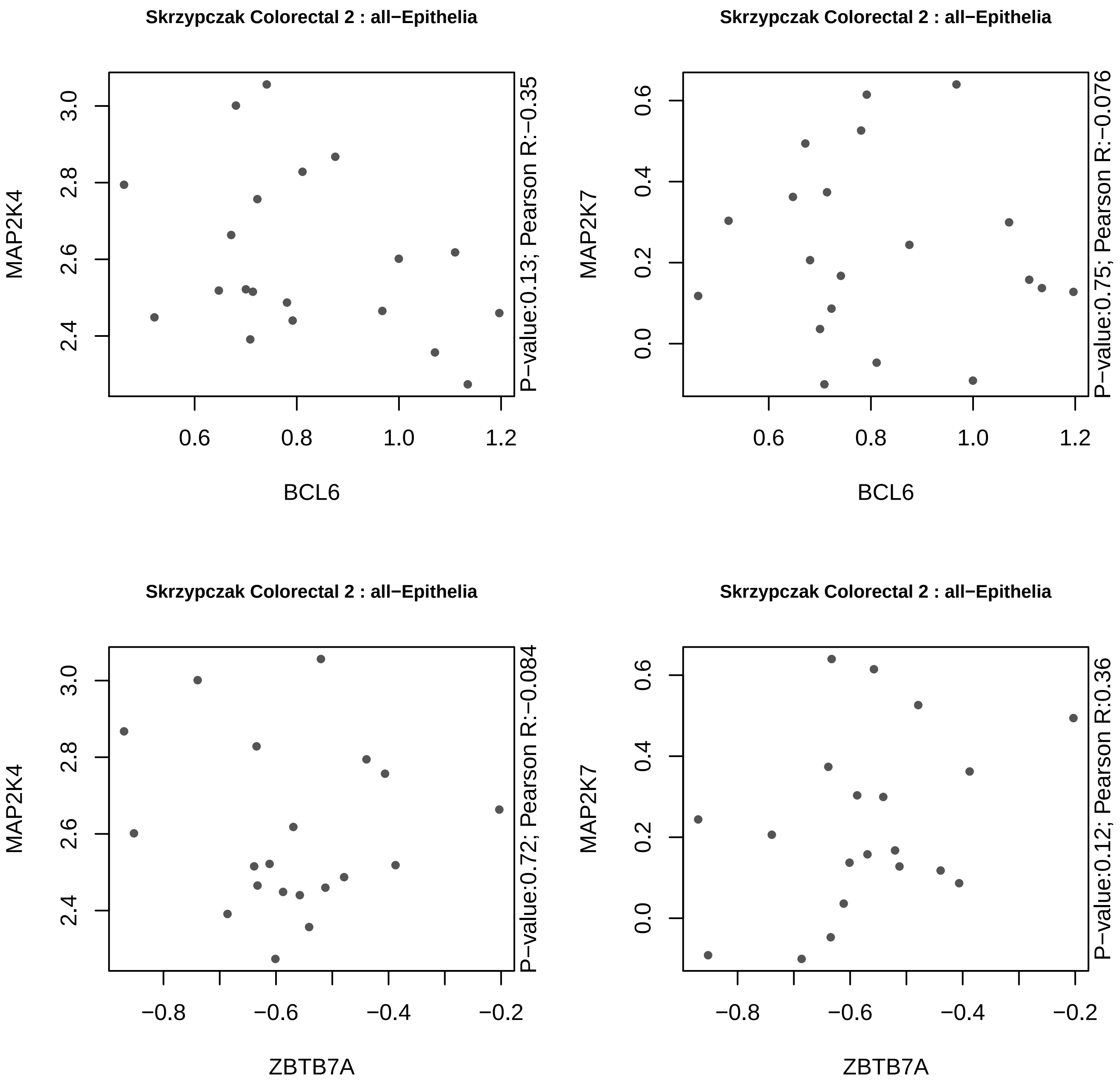
Skrzypczak Colorectal 2 : all−Epithelia
MAP2K4
BCL6
P−value:0.13; Pearson R:−0.35
Skrzypczak Colorectal 2 : all−Epithelia
MAP2K7
BCL6
P−value:0.75; Pearson R:−0.076
Skrzypczak Colorectal 2 : all−Epithelia
MAP2K4
ZBTB7A
P−value:0.72; Pearson R:−0.084
Skrzypczak Colorectal 2 : all−Epithelia
MAP2K7
ZBTB7A
P−value:0.12; Pearson R:0.36

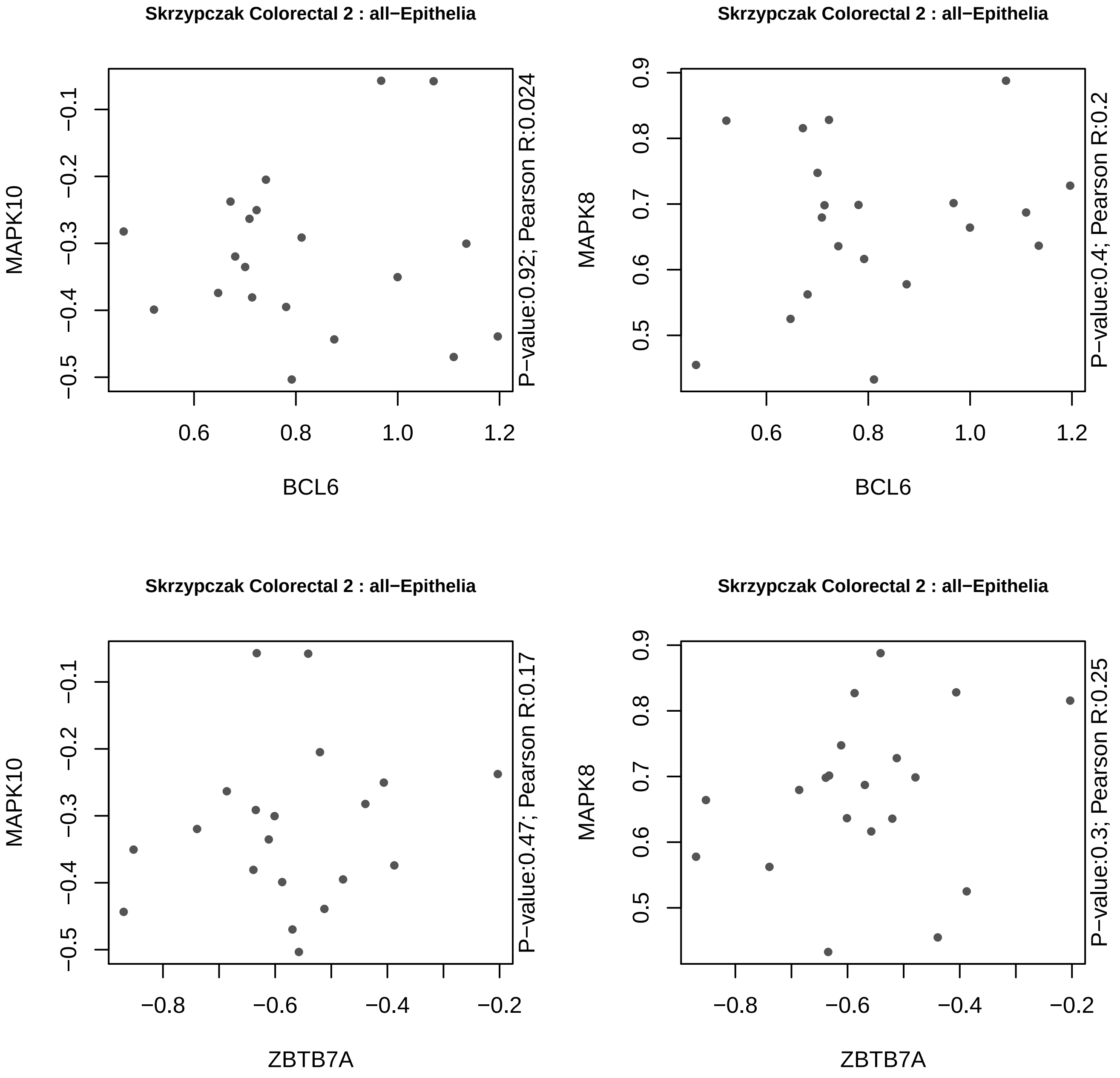
Skrzypczak Colorectal 2 : all−Epithelia
MAPK10
BCL6
P−value:0.92; Pearson R:0.024
Skrzypczak Colorectal 2 : all−Epithelia
MAPK8
BCL6
P−value:0.4; Pearson R:0.2
Skrzypczak Colorectal 2 : all−Epithelia
MAPK10
ZBTB7A
P−value:0.47; Pearson R:0.17
Skrzypczak Colorectal 2 : all−Epithelia
MAPK8
ZBTB7A
P−value:0.3; Pearson R:0.25

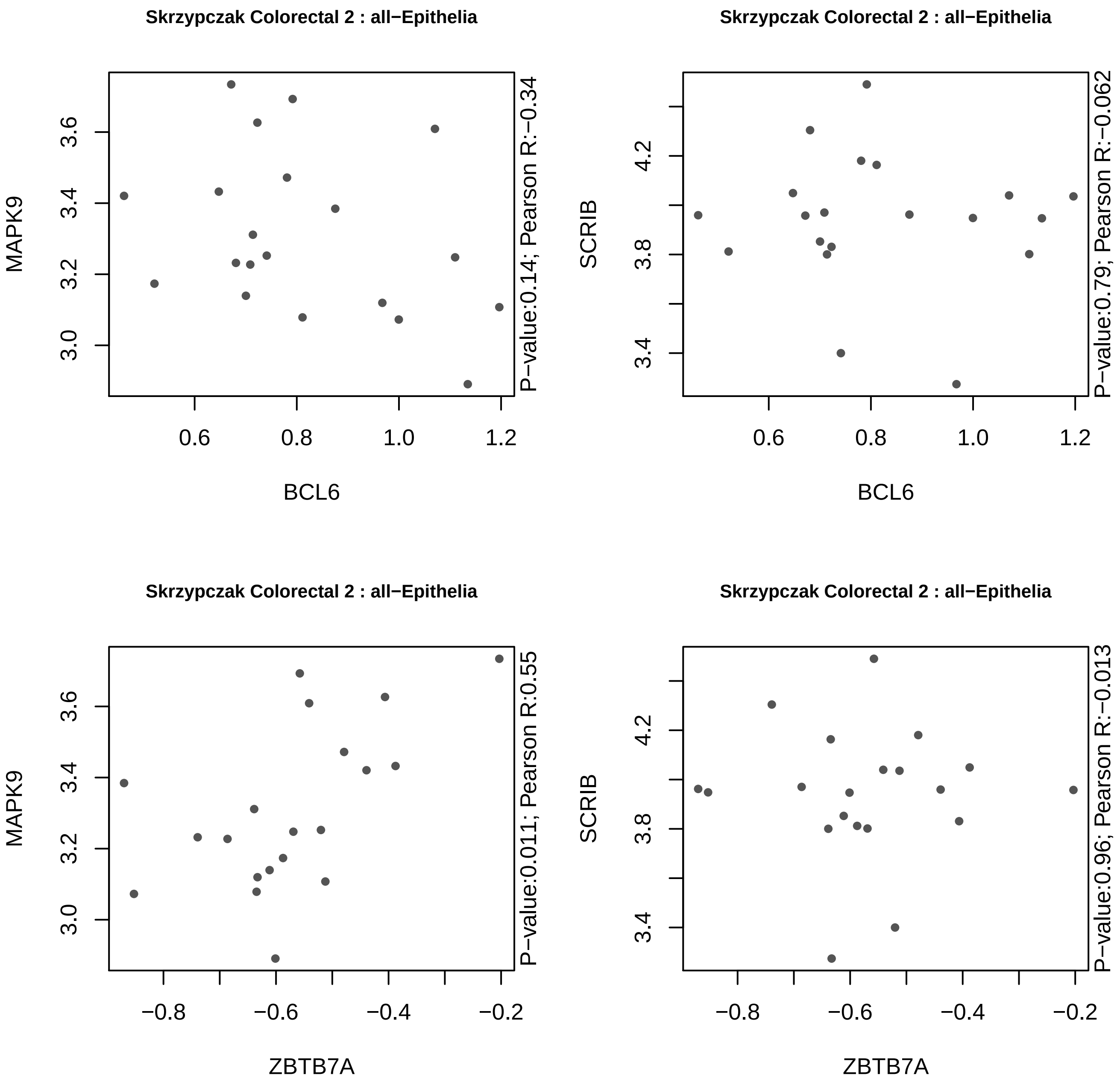
Skrzypczak Colorectal 2 : all−Epithelia
MAPK9
BCL6
P−value:0.14; Pearson R:−0.34
Skrzypczak Colorectal 2 : all−Epithelia
SCRIB
BCL6
P−value:0.79; Pearson R:−0.062
Skrzypczak Colorectal 2 : all−Epithelia
MAPK9
ZBTB7A
P−value:0.011; Pearson R:0.55
Skrzypczak Colorectal 2 : all−Epithelia
SCRIB
ZBTB7A
P−value:0.96; Pearson R:−0.013

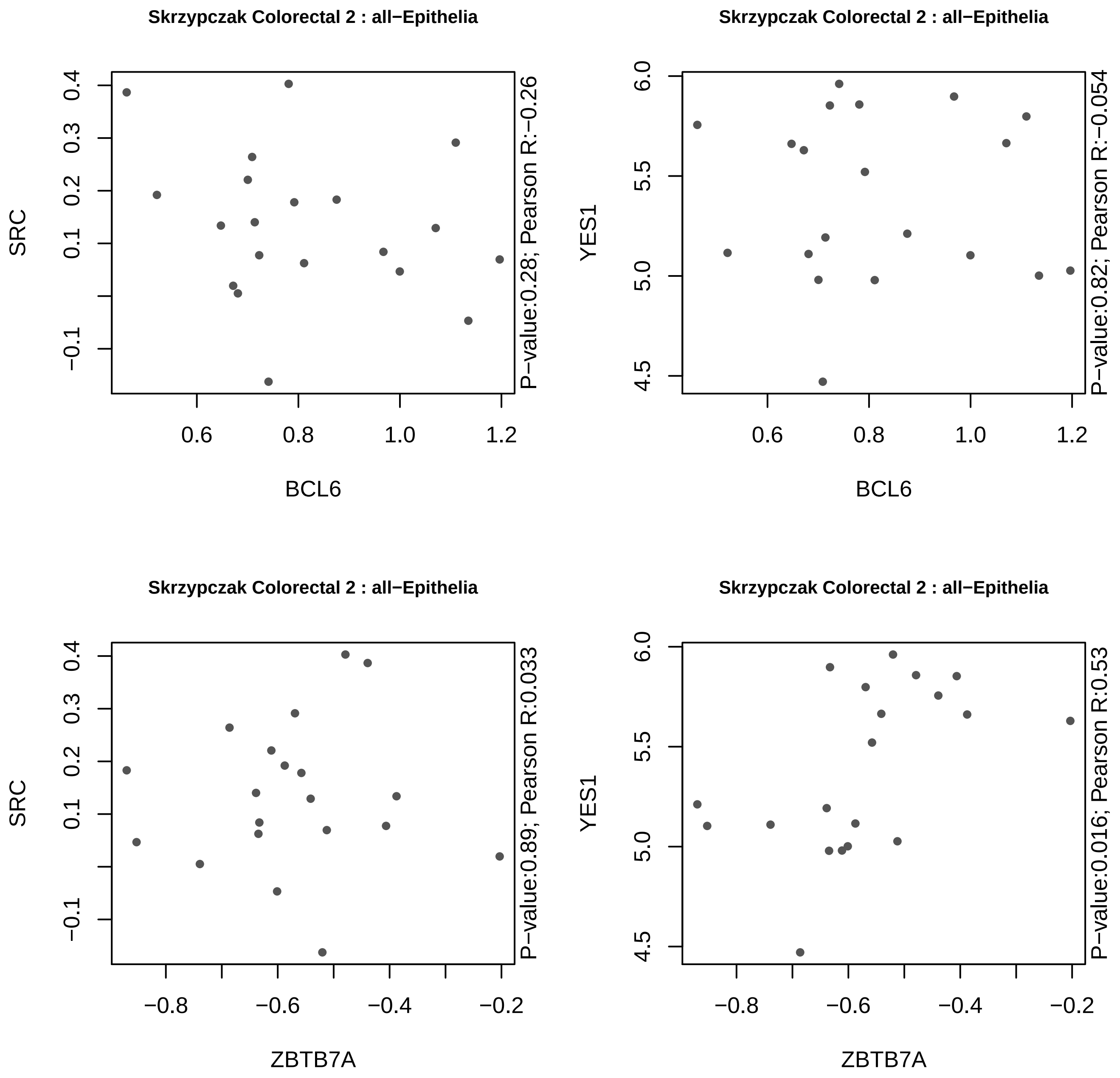
Skrzypczak Colorectal 2 : all−Epithelia
SRC
BCL6
P−value:0.28; Pearson R:−0.26
Skrzypczak Colorectal 2 : all−Epithelia
YES1
BCL6
P−value:0.82; Pearson R:−0.054
Skrzypczak Colorectal 2 : all−Epithelia
SRC
ZBTB7A
P−value:0.89; Pearson R:0.033
Skrzypczak Colorectal 2 : all−Epithelia
YES1
ZBTB7A
P−value:0.016; Pearson R:0.53

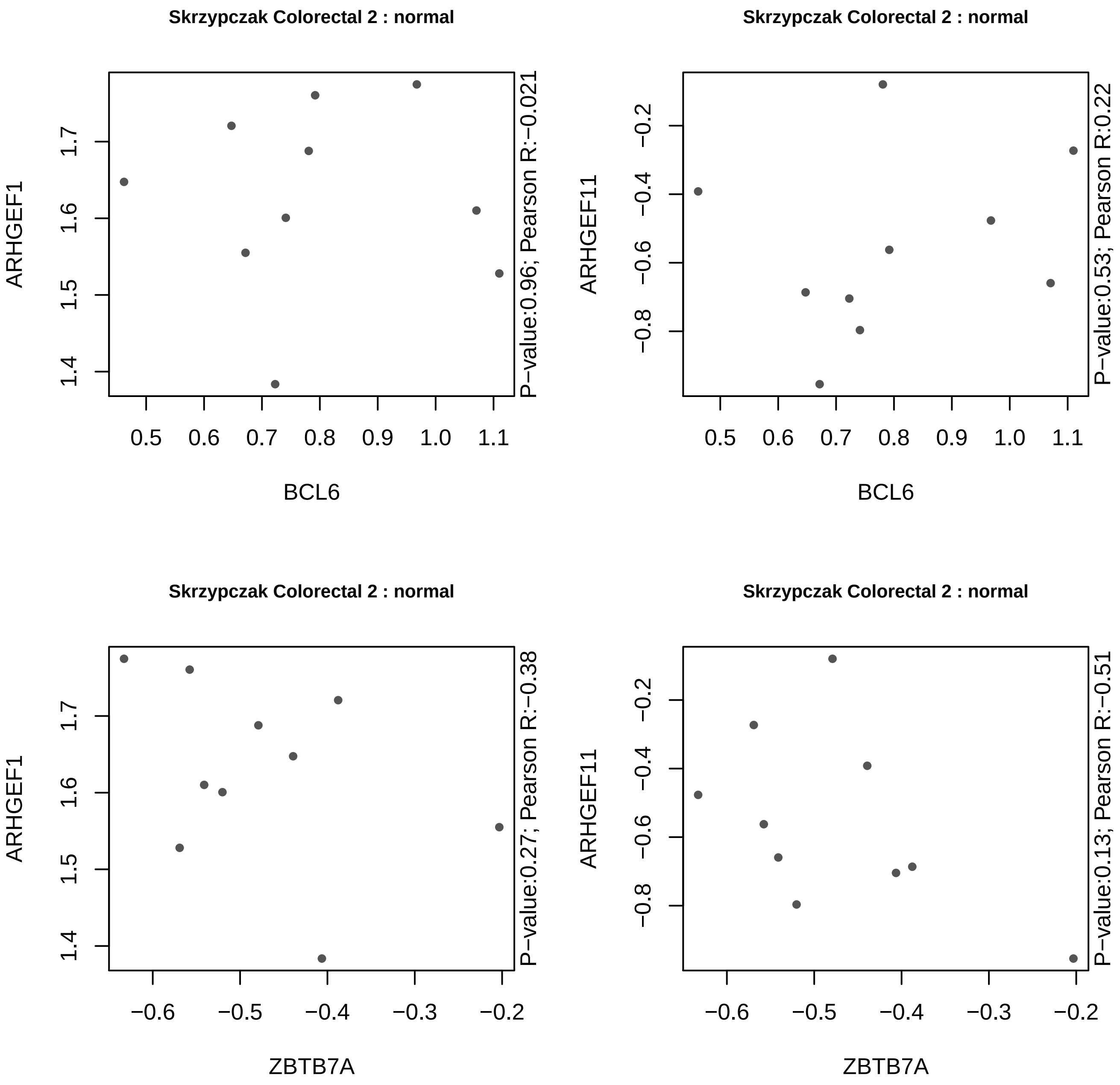
Skrzypczak Colorectal 2 : normal
ARHGEF1
BCL6
P−value:0.96; Pearson R:−0.021
Skrzypczak Colorectal 2 : normal
ARHGEF11
BCL6
P−value:0.53; Pearson R:0.22
Skrzypczak Colorectal 2 : normal
ARHGEF1
ZBTB7A
P−value:0.27; Pearson R:−0.38
Skrzypczak Colorectal 2 : normal
ARHGEF11
ZBTB7A
P−value:0.13; Pearson R:−0.51

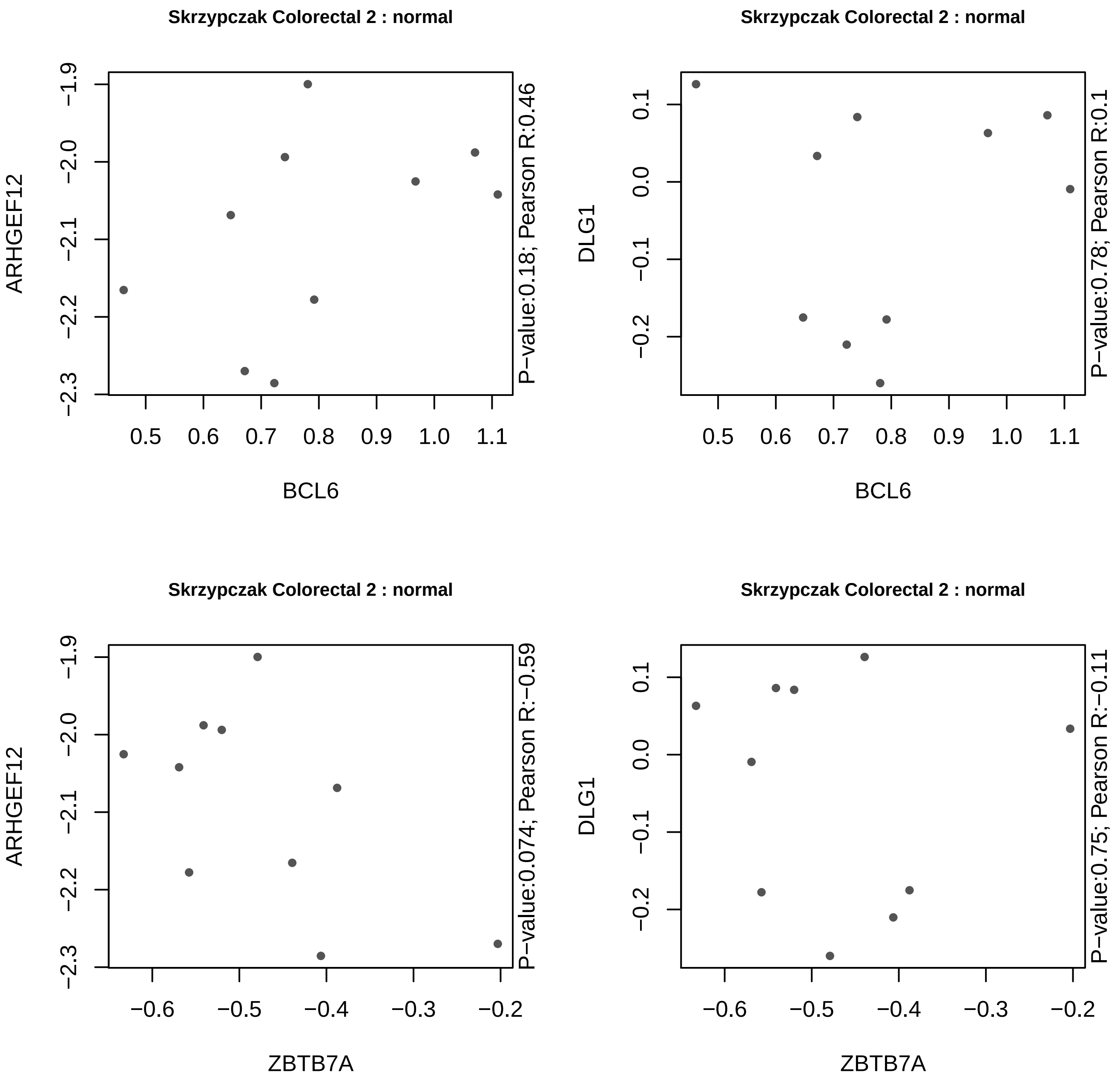
Skrzypczak Colorectal 2 : normal
ARHGEF12
BCL6
P−value:0.18; Pearson R:0.46
Skrzypczak Colorectal 2 : normal
DLG1
BCL6
P−value:0.78; Pearson R:0.1
Skrzypczak Colorectal 2 : normal
ARHGEF12
ZBTB7A
P−value:0.074; Pearson R:−0.59
Skrzypczak Colorectal 2 : normal
DLG1
ZBTB7A
P−value:0.75; Pearson R:−0.11

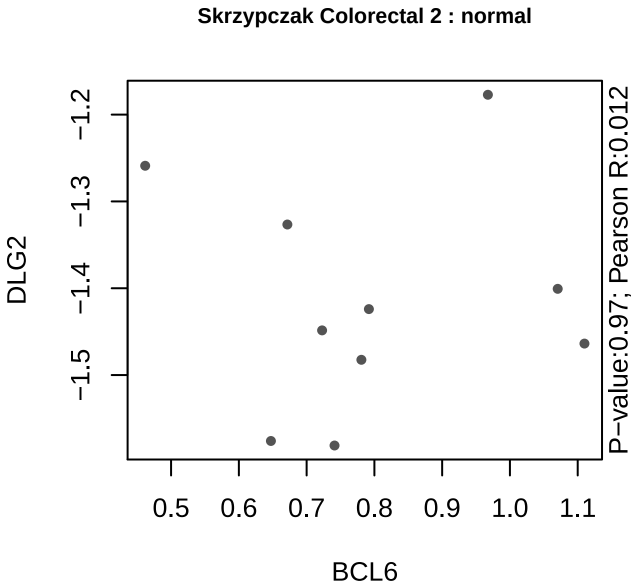
Skrzypczak Colorectal 2 : normal
DLG2
BCL6
P−value:0.97; Pearson R:0.012
−1.2
−1.3
−1.4
−1.5
0.5
0.6
0.7
0.8
0.9
1.0
1.1

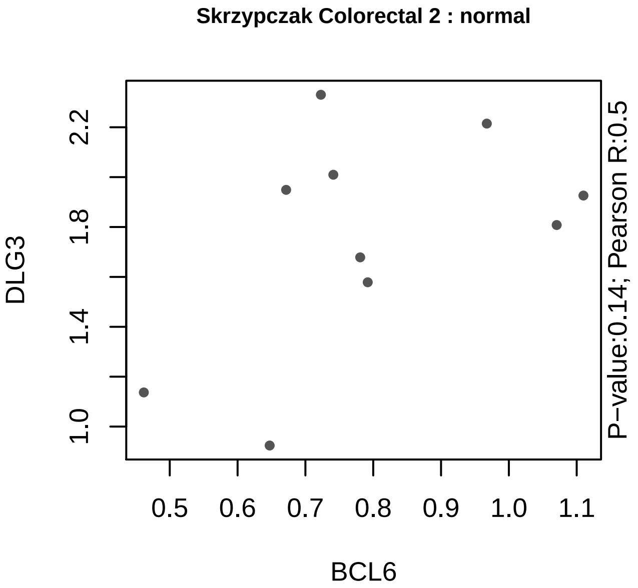
Skrzypczak Colorectal 2 : normal
DLG3
BCL6
P−value:0.14; Pearson R:0.5
2.2
1.8
1.4
1.0
0.5
0.6
0.7
0.8
0.9
1.0
1.1

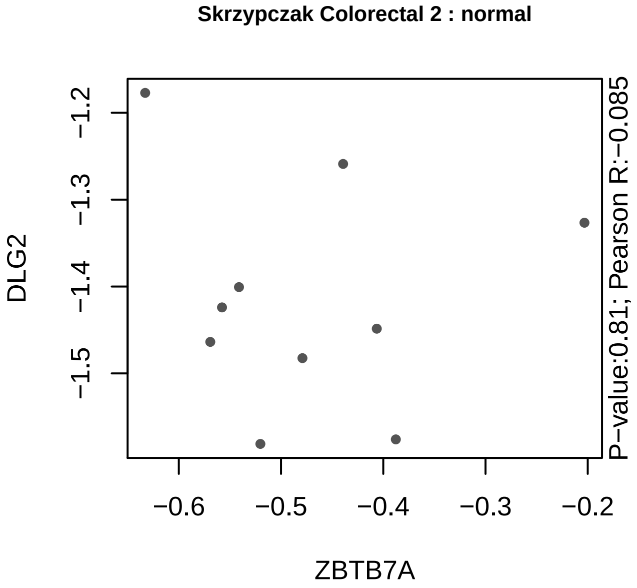
Skrzypczak Colorectal 2 : normal
DLG2
ZBTB7A
P−value:0.81; Pearson R:−0.085
−1.2
−1.3
−1.4
−1.5
−0.6
−0.5
−0.4
−0.3
−0.2

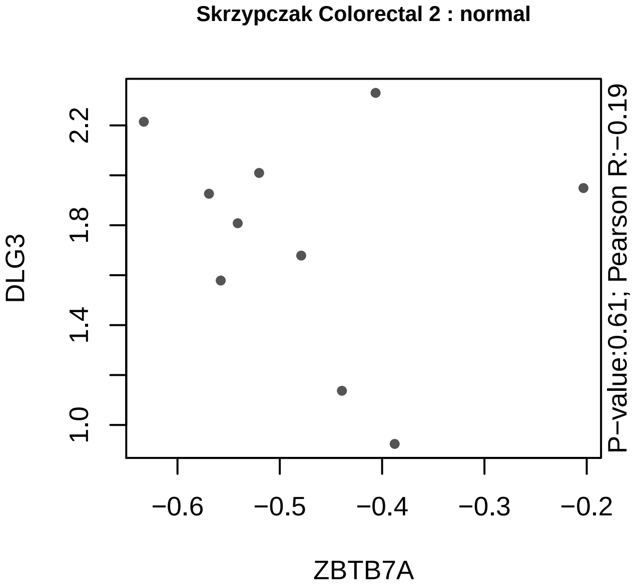
Skrzypczak Colorectal 2 : normal
DLG3
ZBTB7A
P−value:0.61; Pearson R:−0.19
2.2
1.8
1.4
1.0
−0.6
−0.5
−0.4
−0.3
−0.2

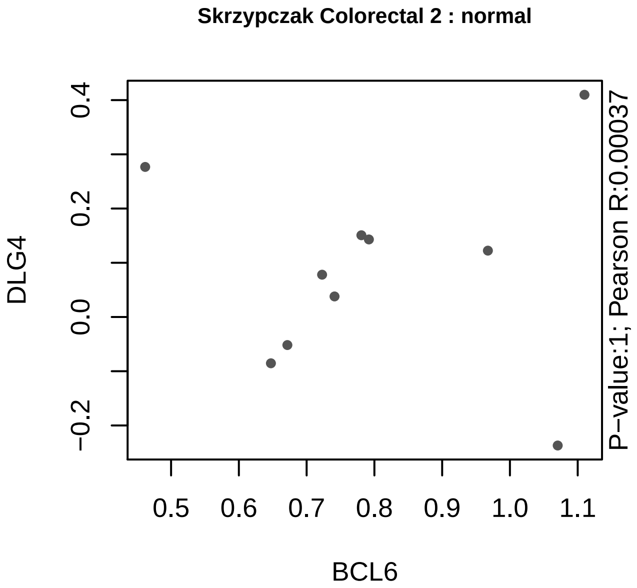
Skrzypczak Colorectal 2 : normal
DLG4
BCL6
P−value:1; Pearson R:0.00037
0.5 0.6 0.7 0.8 0.9 1.0 1.1
−0.2 0.0 0.2 0.4

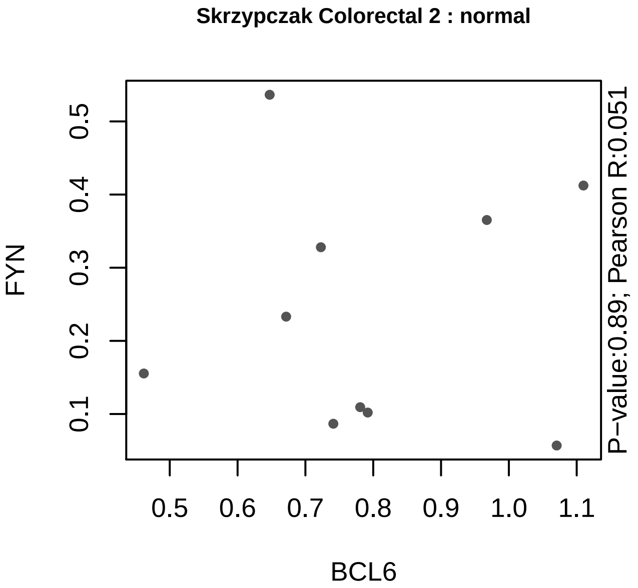
Skrzypczak Colorectal 2 : normal
FYN
BCL6
P−value:0.89; Pearson R:0.051
0.5 0.6 0.7 0.8 0.9 1.0 1.1
0.1 0.2 0.3 0.4 0.5

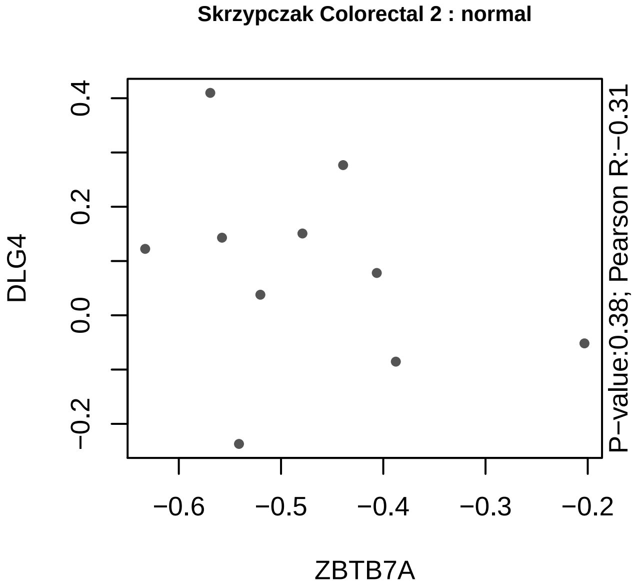
Skrzypczak Colorectal 2 : normal
DLG4
ZBTB7A
P−value:0.38; Pearson R:−0.31
−0.6 −0.5 −0.4 −0.3 −0.2
−0.2 0.0 0.2 0.4

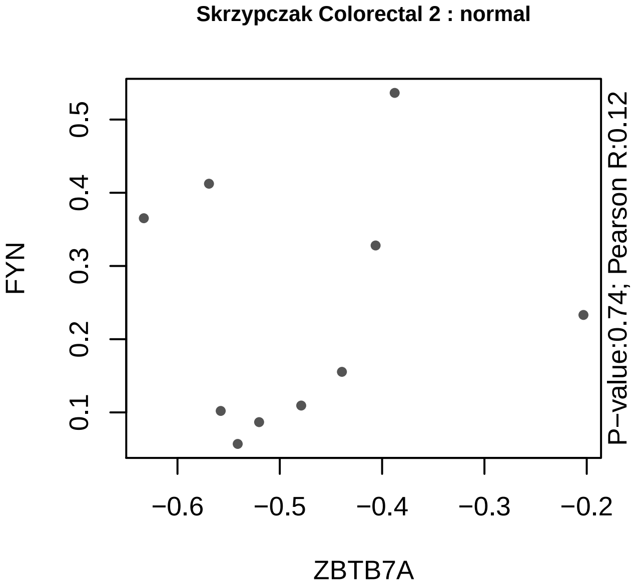
Skrzypczak Colorectal 2 : normal
FYN
ZBTB7A
P−value:0.74; Pearson R:0.12
−0.6 −0.5 −0.4 −0.3 −0.2
0.1 0.2 0.3 0.4 0.5

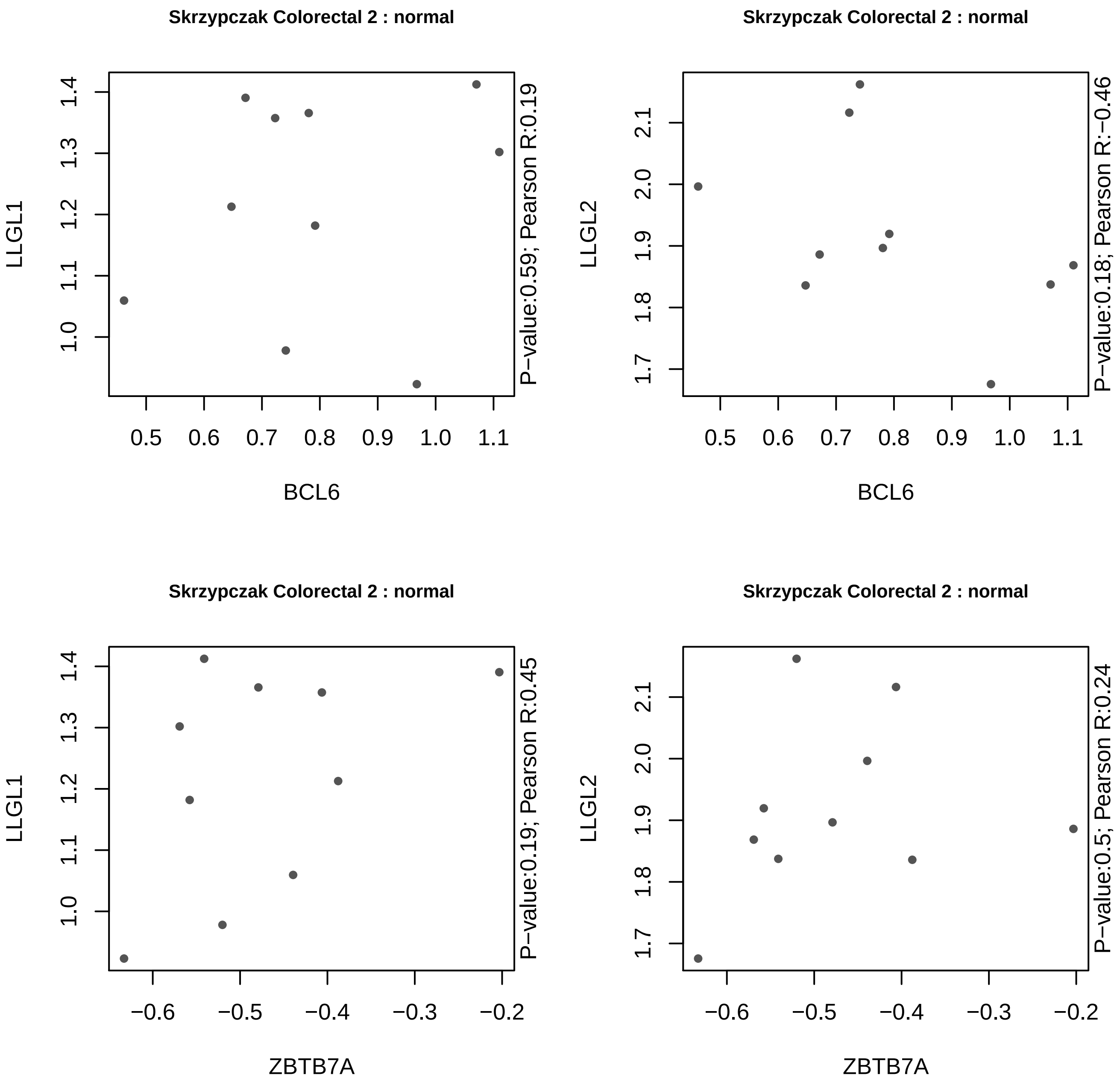
Skrzypczak Colorectal 2 : normal
LLGL1
BCL6
P−value:0.59; Pearson R:0.19
Skrzypczak Colorectal 2 : normal
LLGL2
BCL6
P−value:0.18; Pearson R:−0.46
Skrzypczak Colorectal 2 : normal
LLGL1
ZBTB7A
P−value:0.19; Pearson R:0.45
Skrzypczak Colorectal 2 : normal
LLGL2
ZBTB7A
P−value:0.5; Pearson R:0.24

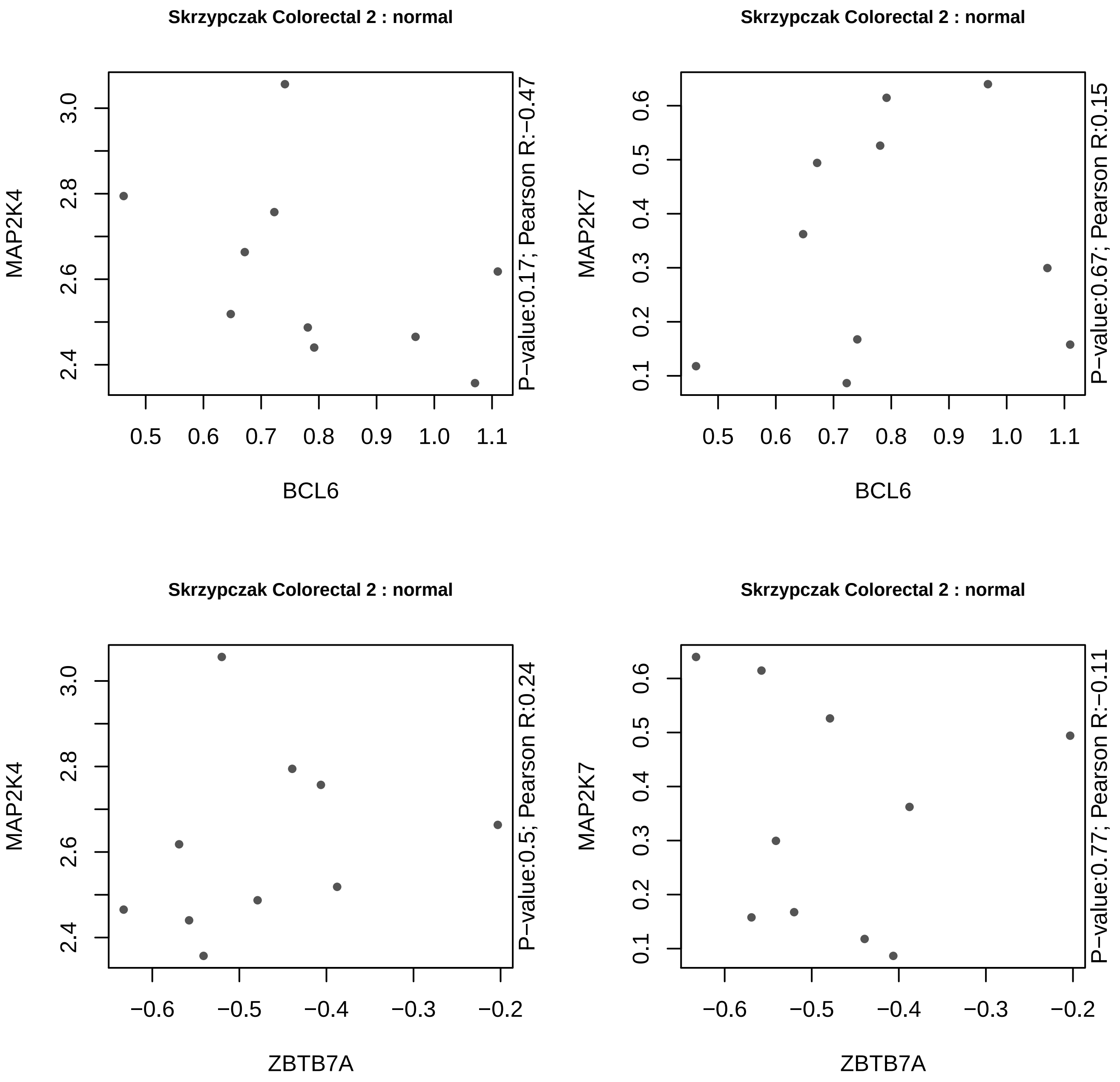
Skrzypczak Colorectal 2 : normal
MAP2K4
BCL6
P−value:0.17; Pearson R:−0.47
Skrzypczak Colorectal 2 : normal
MAP2K7
BCL6
P−value:0.67; Pearson R:0.15
Skrzypczak Colorectal 2 : normal
MAP2K4
ZBTB7A
P−value:0.5; Pearson R:0.24
Skrzypczak Colorectal 2 : normal
MAP2K7
ZBTB7A
P−value:0.77; Pearson R:−0.11

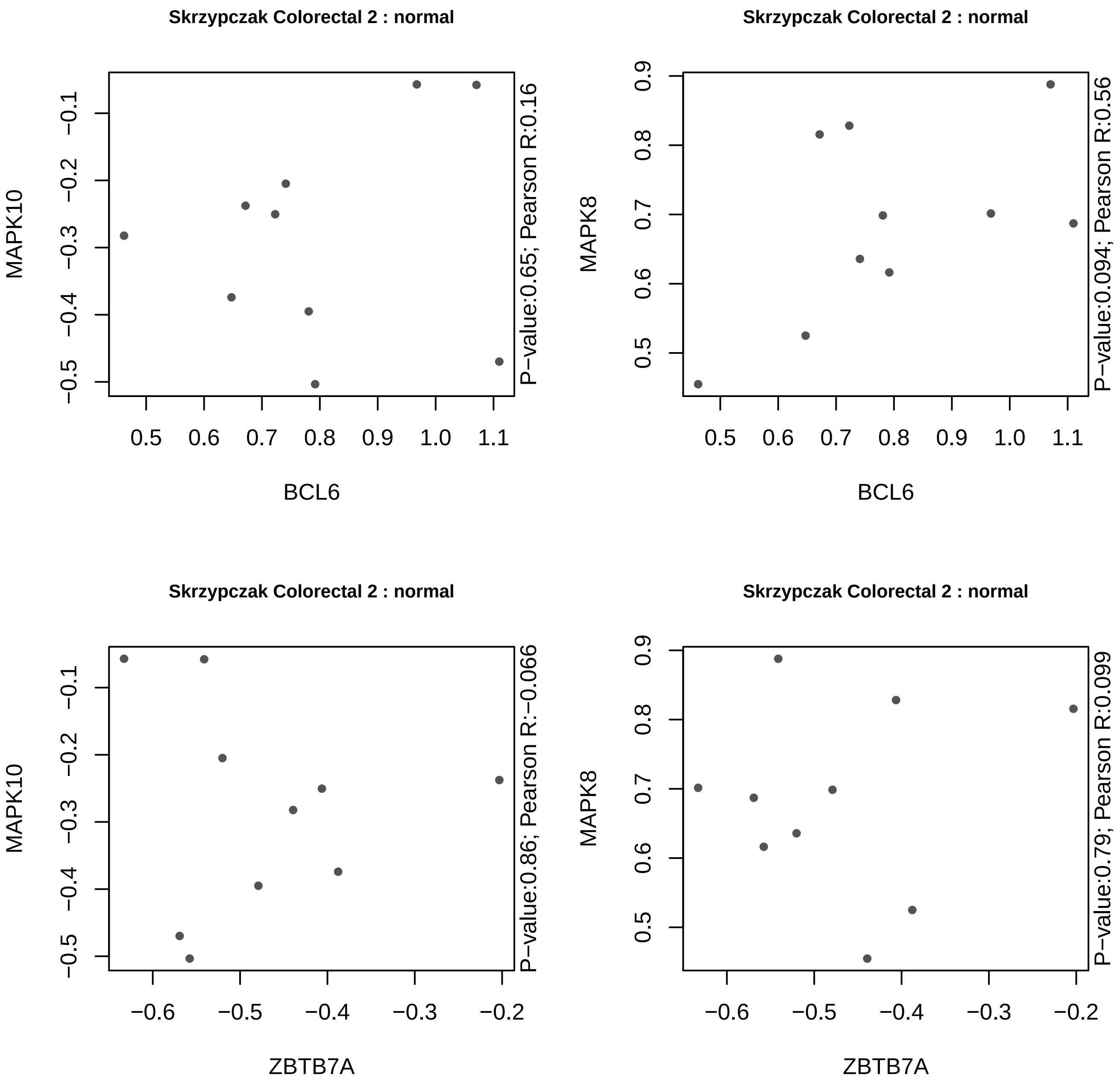
Skrzypczak Colorectal 2 : normal
MAPK10
BCL6
P−value:0.65; Pearson R:0.16
Skrzypczak Colorectal 2 : normal
MAPK8
BCL6
P−value:0.094; Pearson R:0.56
Skrzypczak Colorectal 2 : normal
MAPK10
ZBTB7A
P−value:0.86; Pearson R:−0.066
Skrzypczak Colorectal 2 : normal
MAPK8
ZBTB7A
P−value:0.79; Pearson R:0.099

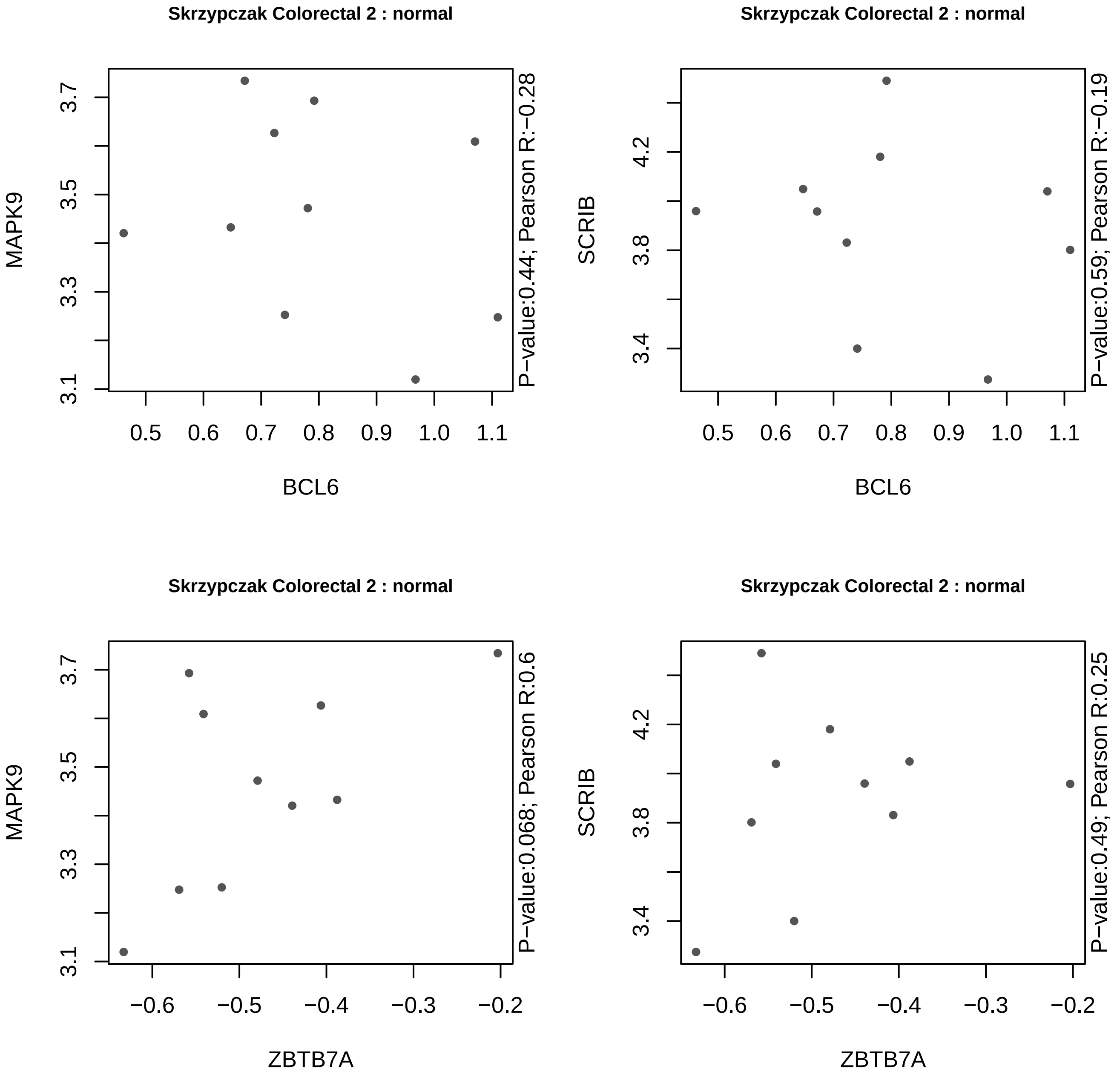
Skrzypczak Colorectal 2 : normal
MAPK9
BCL6
P−value:0.44; Pearson R:−0.28
Skrzypczak Colorectal 2 : normal
SCRIB
BCL6
P−value:0.59; Pearson R:−0.19
Skrzypczak Colorectal 2 : normal
MAPK9
ZBTB7A
P−value:0.068; Pearson R:0.6
Skrzypczak Colorectal 2 : normal
SCRIB
ZBTB7A
P−value:0.49; Pearson R:0.25

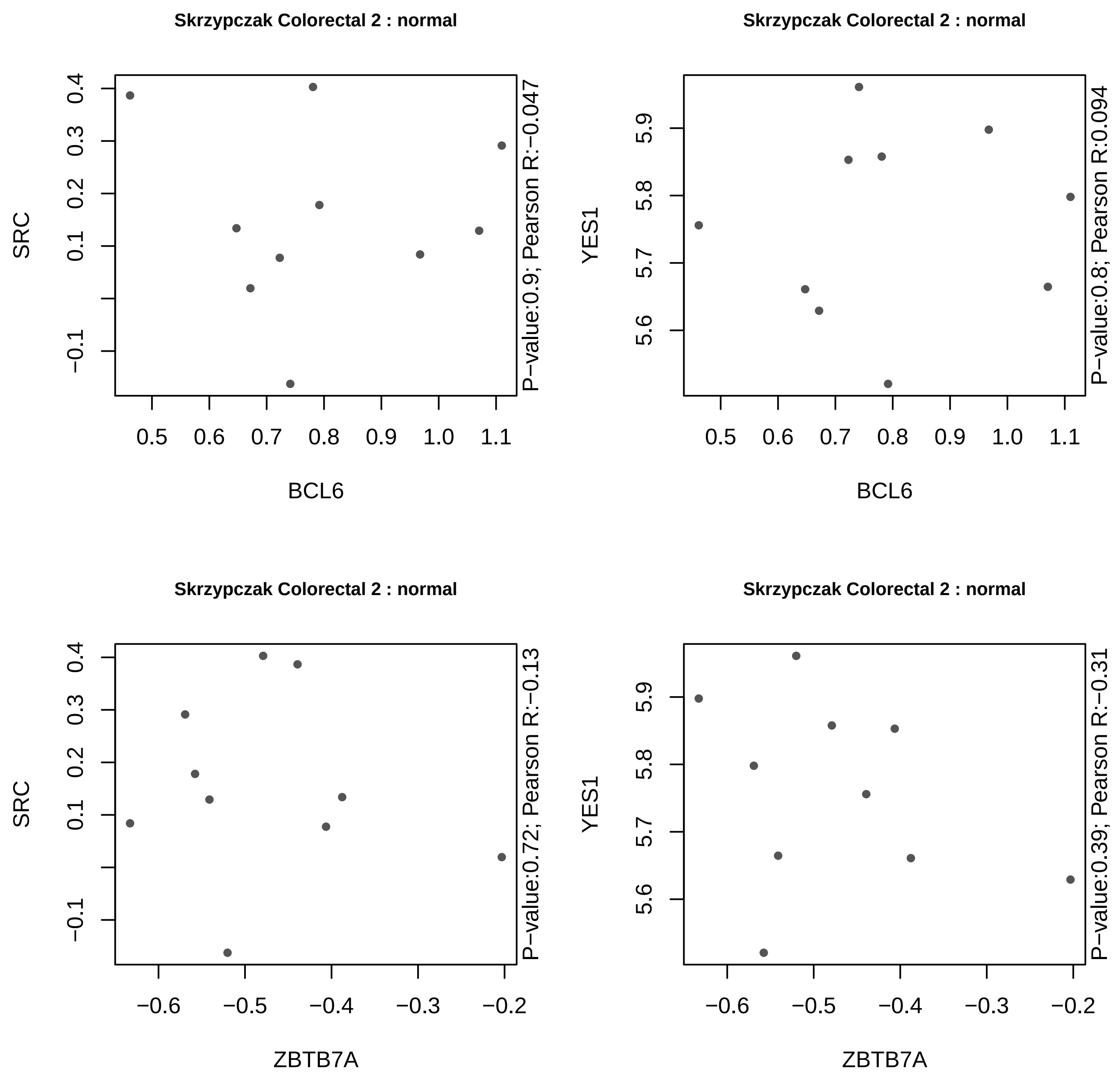
Skrzypczak Colorectal 2 : normal
SRC
BCL6
P−value:0.9; Pearson R:−0.047
0.5 0.6 0.7 0.8 0.9 1.0 1.1
−0.1 0.1 0.2 0.3 0.4
Skrzypczak Colorectal 2 : normal
YES1
BCL6
P−value:0.8; Pearson R:0.094
5.6 5.7 5.8 5.9
Skrzypczak Colorectal 2 : normal
SRC
ZBTB7A
P−value:0.72; Pearson R:−0.13
−0.6 −0.5 −0.4 −0.3 −0.2
Skrzypczak Colorectal 2 : normal
YES1
ZBTB7A
P−value:0.39; Pearson R:−0.31

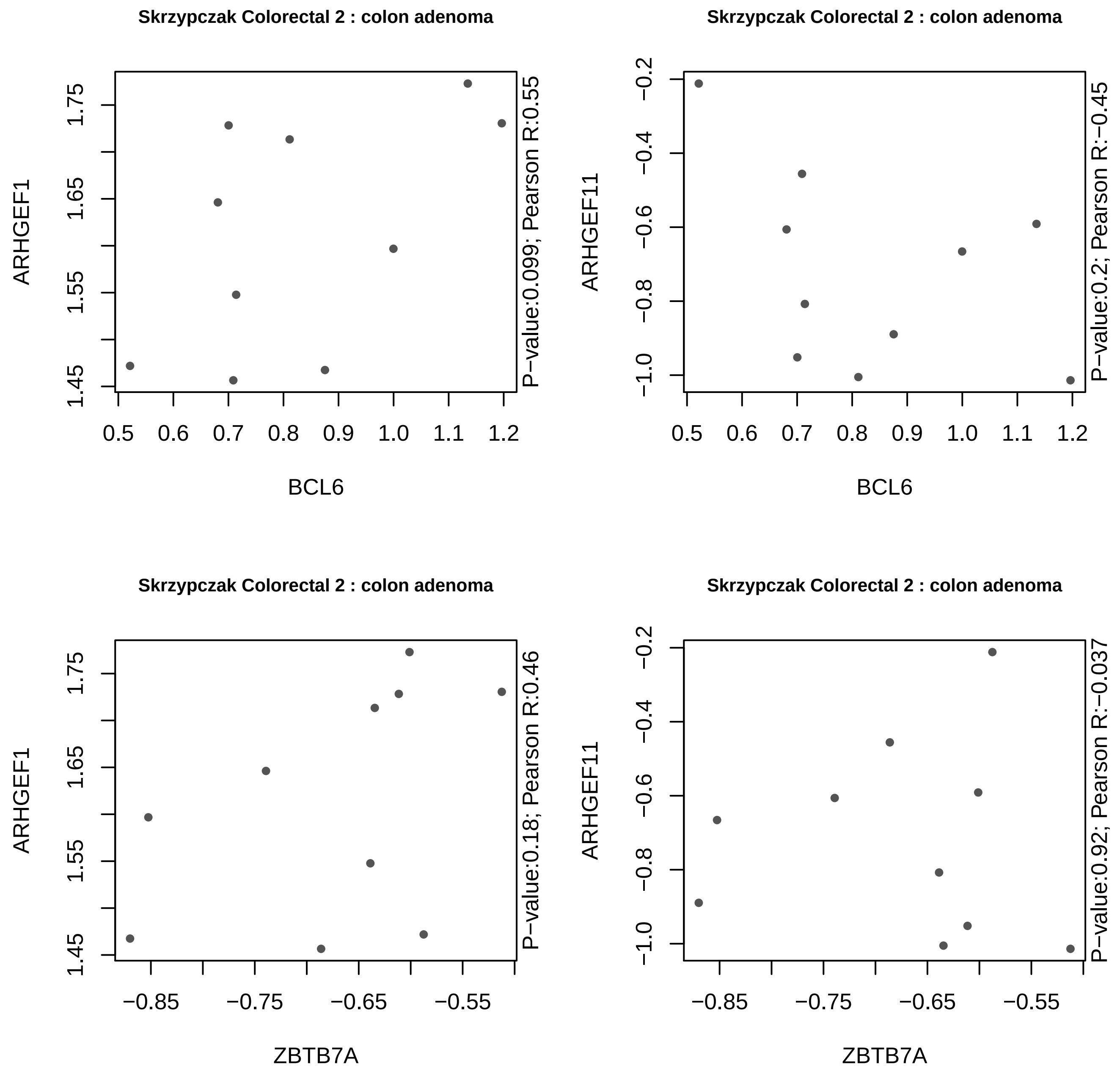
Skrzypczak Colorectal 2 : colon adenoma
ARHGEF1
BCL6
P−value:0.099; Pearson R:0.55
Skrzypczak Colorectal 2 : colon adenoma
ARHGEF11
BCL6
P−value:0.2; Pearson R:−0.45
Skrzypczak Colorectal 2 : colon adenoma
ARHGEF1
ZBTB7A
P−value:0.18; Pearson R:0.46
Skrzypczak Colorectal 2 : colon adenoma
ARHGEF11
ZBTB7A
P−value:0.92; Pearson R:−0.037

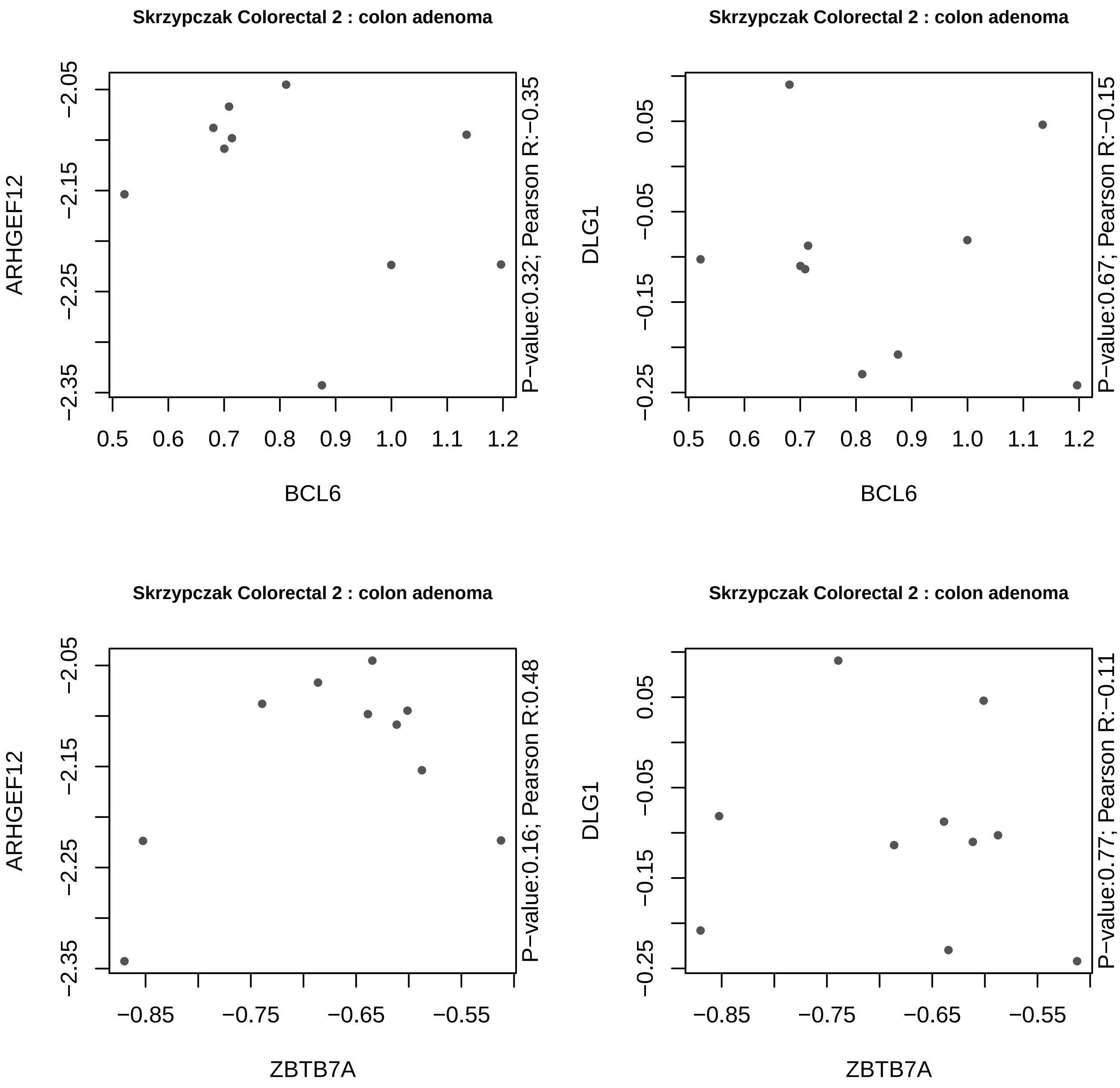
Skrzypczak Colorectal 2 : colon adenoma
ARHGEF12
BCL6
P−value:0.32; Pearson R:−0.35
Skrzypczak Colorectal 2 : colon adenoma
DLG1
BCL6
P−value:0.67; Pearson R:−0.15
Skrzypczak Colorectal 2 : colon adenoma
ARHGEF12
ZBTB7A
P−value:0.16; Pearson R:0.48
Skrzypczak Colorectal 2 : colon adenoma
DLG1
ZBTB7A
P−value:0.77; Pearson R:−0.11

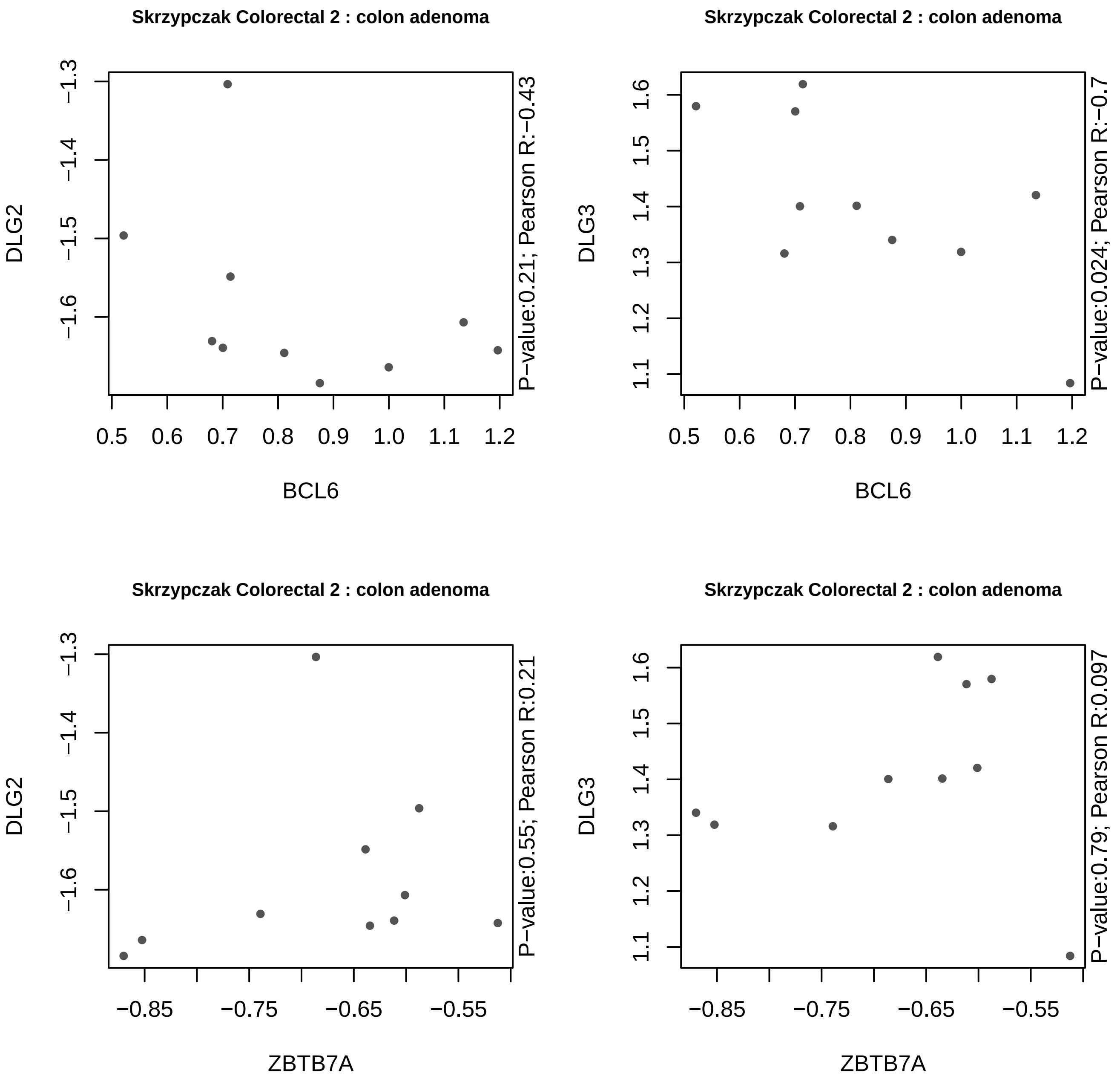
Skrzypczak Colorectal 2 : colon adenoma
DLG2
BCL6
P−value:0.21; Pearson R:−0.43
−1.3
−1.4
−1.5
−1.6
0.5
0.6
0.7
0.8
0.9
1.0
1.1
1.2
Skrzypczak Colorectal 2 : colon adenoma
DLG3
BCL6
P−value:0.024; Pearson R:−0.7
1.6
1.5
1.4
1.3
1.2
1.1
Skrzypczak Colorectal 2 : colon adenoma
DLG2
ZBTB7A
P−value:0.55; Pearson R:0.21
−0.85
−0.75
−0.65
−0.55
Skrzypczak Colorectal 2 : colon adenoma
DLG3
ZBTB7A
P−value:0.79; Pearson R:0.097

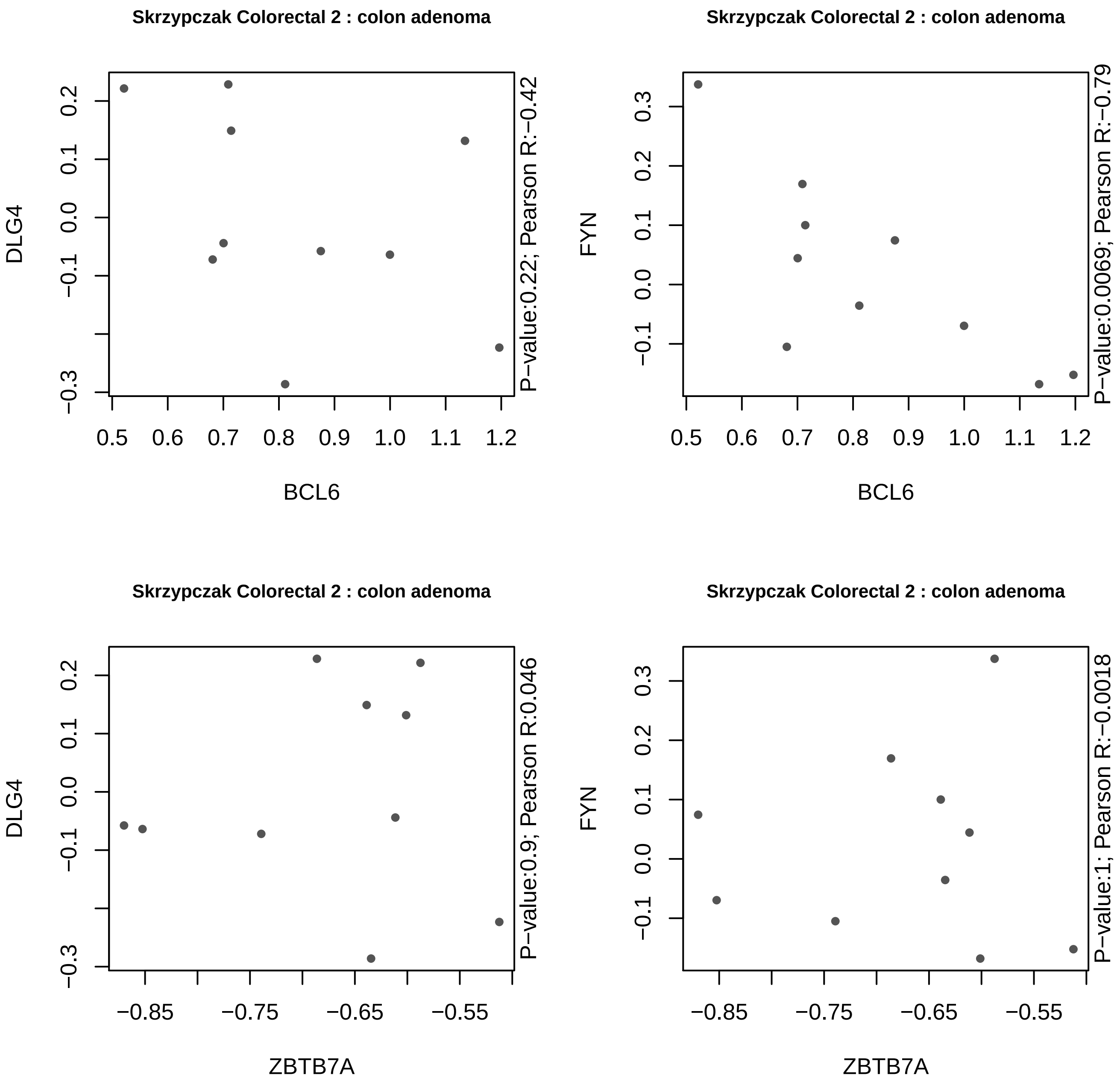
Skrzypczak Colorectal 2 : colon adenoma
DLG4
BCL6
P−value:0.22; Pearson R:−0.42
Skrzypczak Colorectal 2 : colon adenoma
FYN
BCL6
P−value:0.0069; Pearson R:−0.79
Skrzypczak Colorectal 2 : colon adenoma
DLG4
ZBTB7A
P−value:0.9; Pearson R:0.046
Skrzypczak Colorectal 2 : colon adenoma
FYN
ZBTB7A
P−value:1; Pearson R:−0.0018

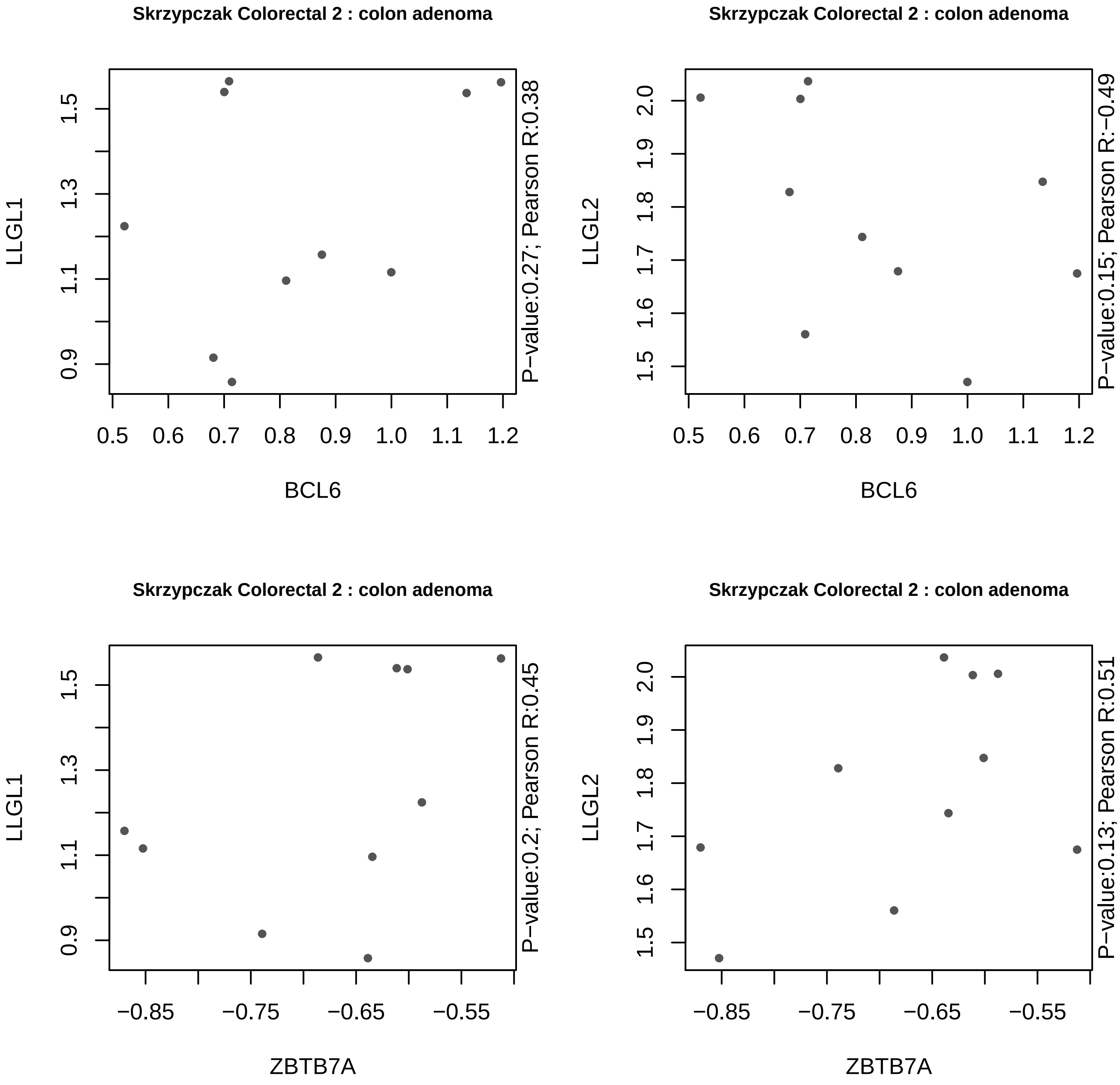
Skrzypczak Colorectal 2 : colon adenoma
LLGL1
BCL6
P−value:0.27; Pearson R:0.38
Skrzypczak Colorectal 2 : colon adenoma
LLGL2
BCL6
P−value:0.15; Pearson R:−0.49
Skrzypczak Colorectal 2 : colon adenoma
LLGL1
ZBTB7A
P−value:0.2; Pearson R:0.45
Skrzypczak Colorectal 2 : colon adenoma
LLGL2
ZBTB7A
P−value:0.13; Pearson R:0.51

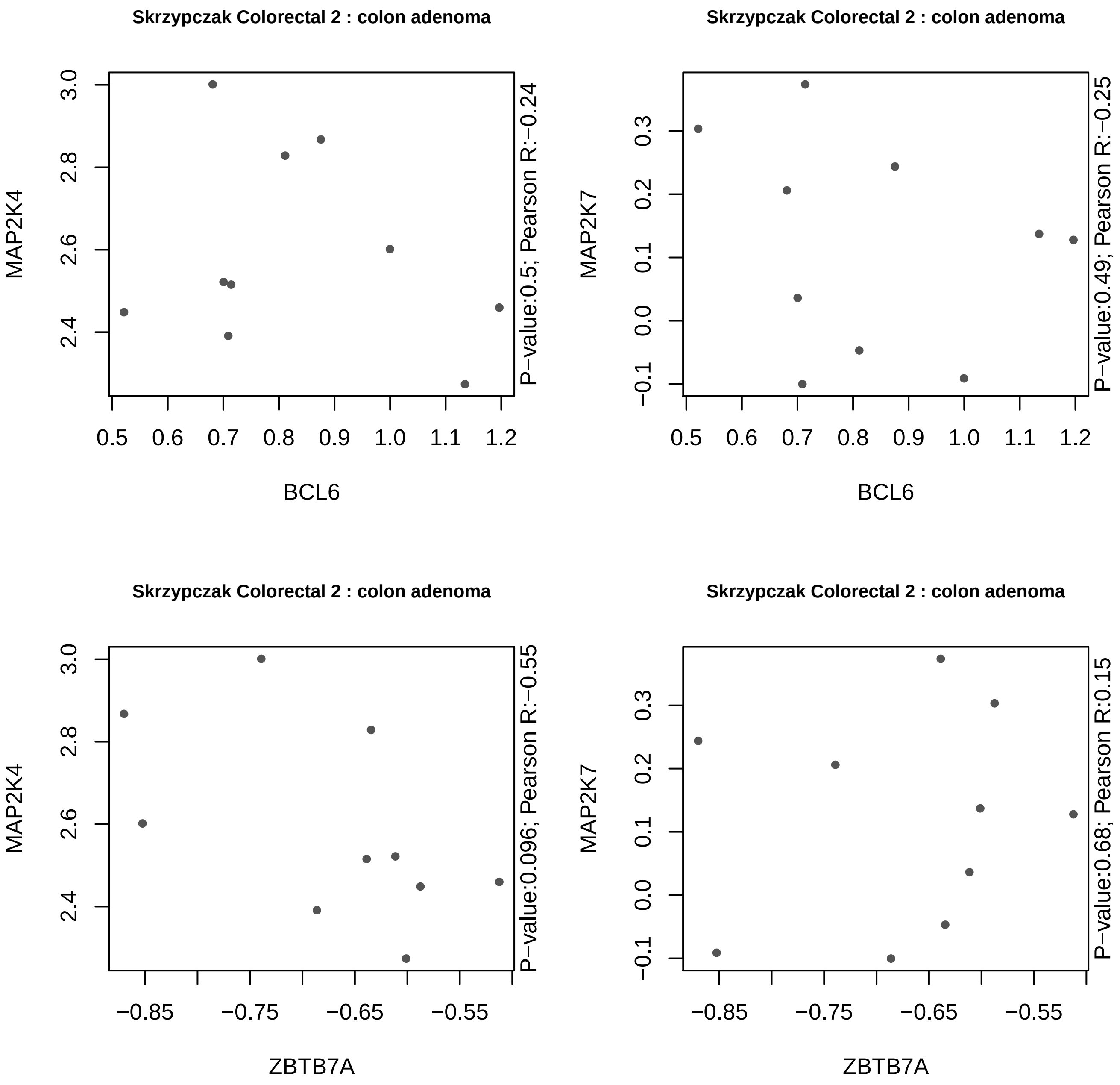
Skrzypczak Colorectal 2 : colon adenoma
MAP2K4
BCL6
P−value:0.5; Pearson R:−0.24
Skrzypczak Colorectal 2 : colon adenoma
MAP2K7
BCL6
P−value:0.49; Pearson R:−0.25
Skrzypczak Colorectal 2 : colon adenoma
MAP2K4
ZBTB7A
P−value:0.096; Pearson R:−0.55
Skrzypczak Colorectal 2 : colon adenoma
MAP2K7
ZBTB7A
P−value:0.68; Pearson R:0.15

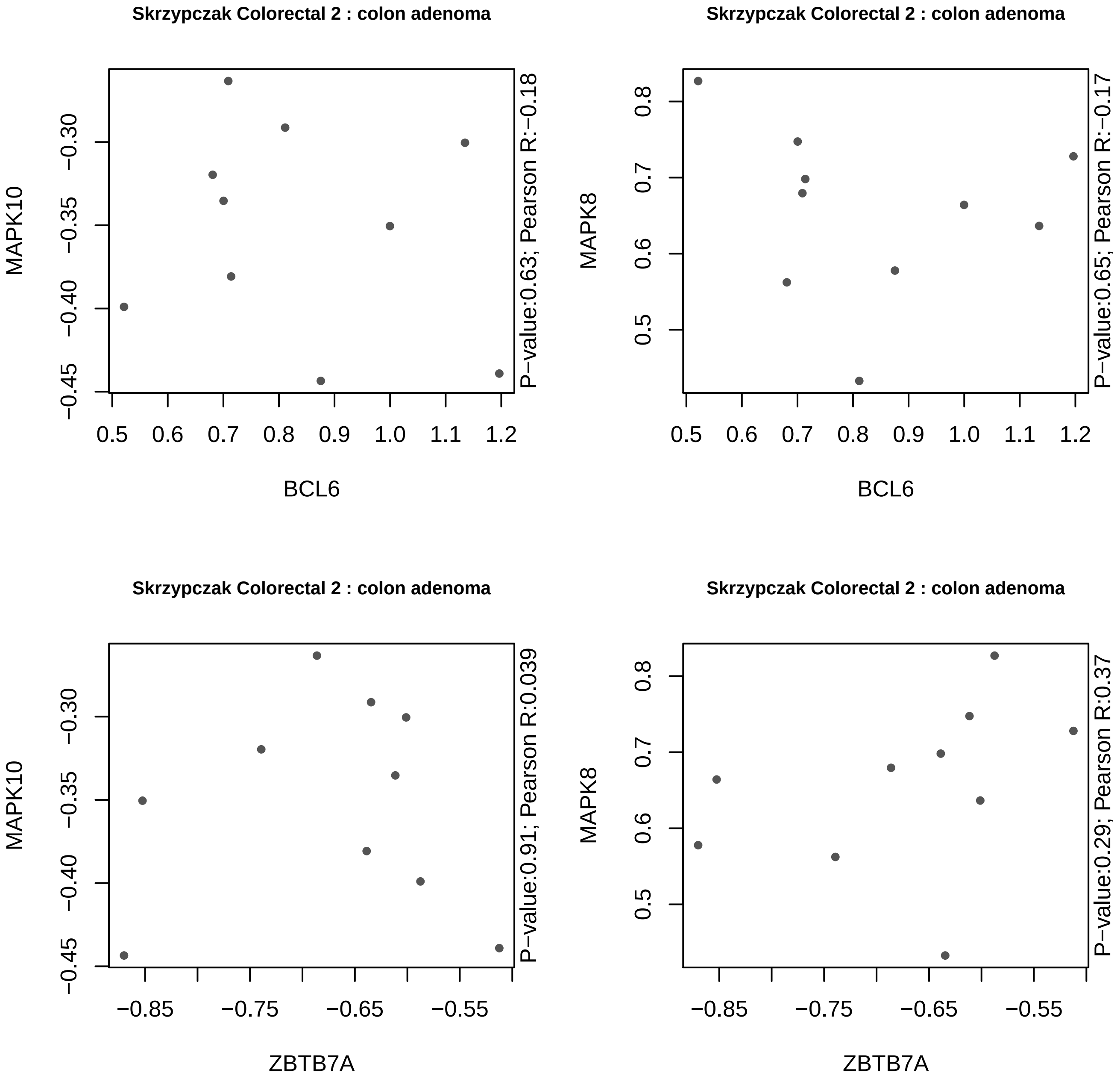
Skrzypczak Colorectal 2 : colon adenoma
MAPK10
BCL6
P−value:0.63; Pearson R:−0.18
Skrzypczak Colorectal 2 : colon adenoma
MAPK8
BCL6
P−value:0.65; Pearson R:−0.17
Skrzypczak Colorectal 2 : colon adenoma
MAPK10
ZBTB7A
P−value:0.91; Pearson R:0.039
Skrzypczak Colorectal 2 : colon adenoma
MAPK8
ZBTB7A
P−value:0.29; Pearson R:0.37

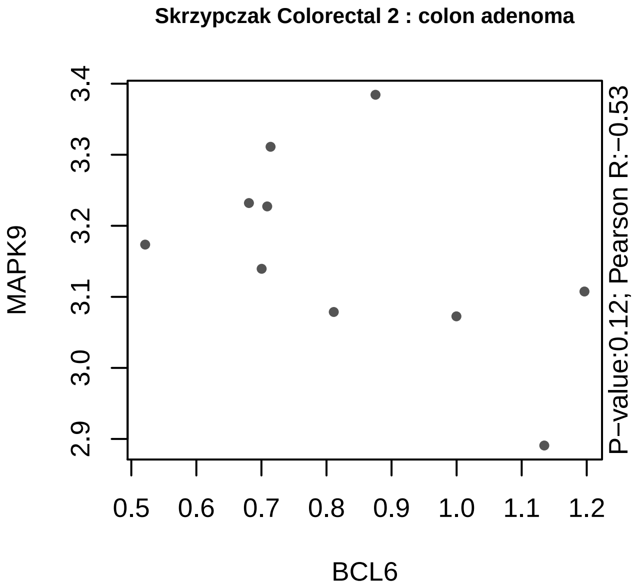
Skrzypczak Colorectal 2 : colon adenoma
MAPK9
BCL6
P−value:0.12; Pearson R:−0.53
3.4
3.3
3.2
3.1
3.0
2.9
0.5
0.6
0.7
0.8
0.9
1.0
1.1
1.2

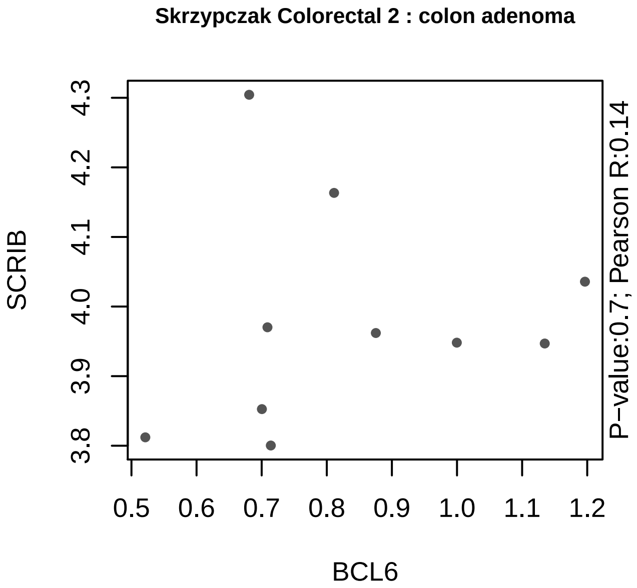
Skrzypczak Colorectal 2 : colon adenoma
SCRIB
BCL6
P−value:0.7; Pearson R:0.14
4.3
4.2
4.1
4.0
3.9
3.8
0.5
0.6
0.7
0.8
0.9
1.0
1.1
1.2

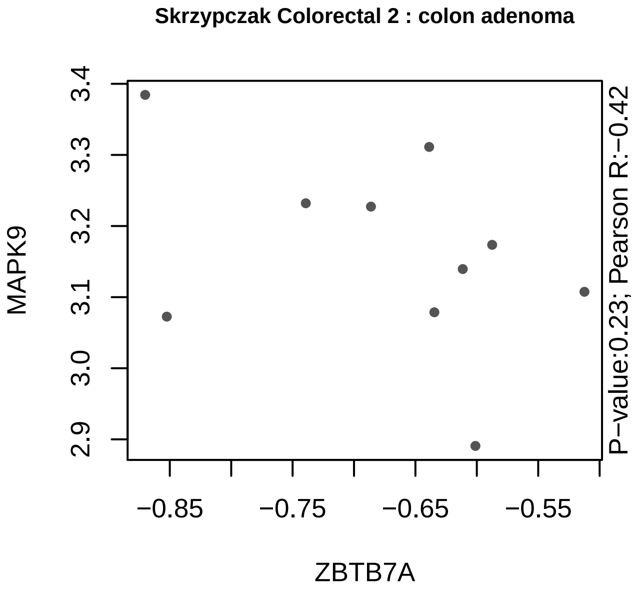
Skrzypczak Colorectal 2 : colon adenoma
MAPK9
ZBTB7A
P−value:0.23; Pearson R:−0.42
3.4
3.3
3.2
3.1
3.0
2.9
−0.85
−0.75
−0.65
−0.55

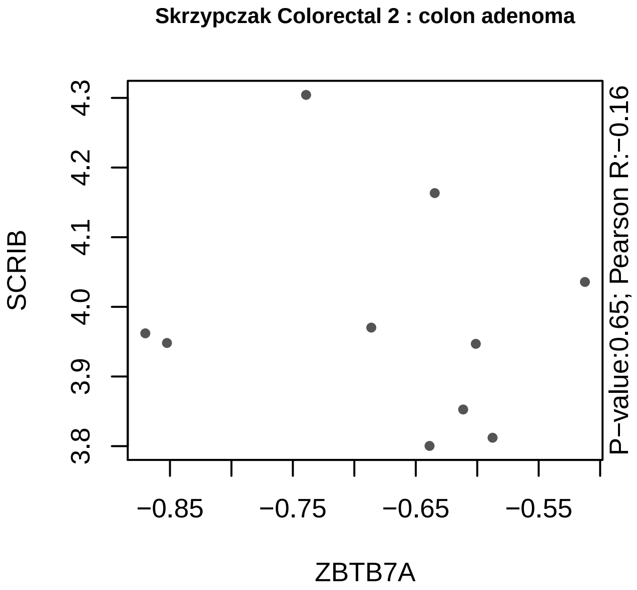
Skrzypczak Colorectal 2 : colon adenoma
SCRIB
ZBTB7A
P−value:0.65; Pearson R:−0.16
4.3
4.2
4.1
4.0
3.9
3.8
−0.85
−0.75
−0.65
−0.55

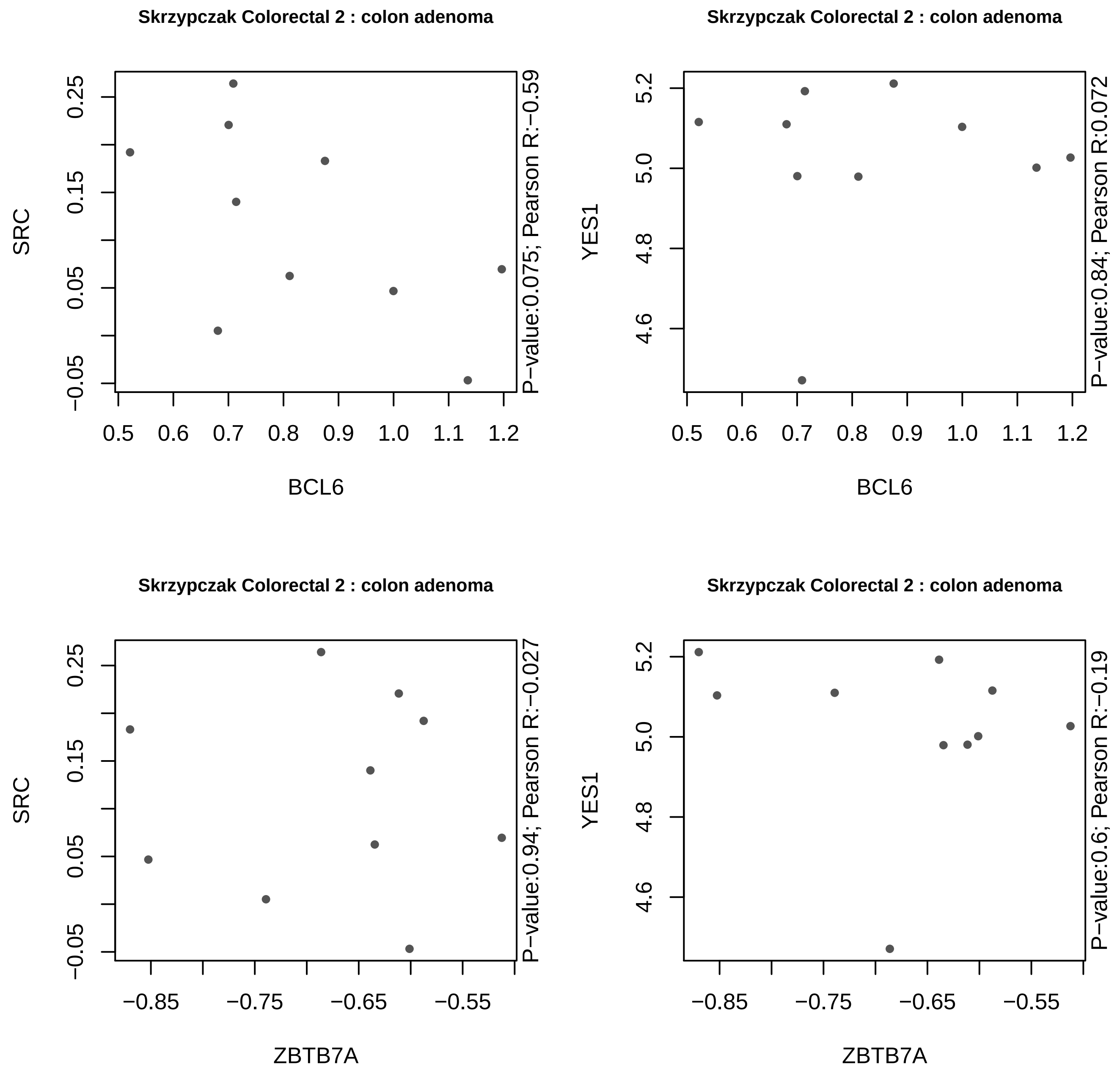
Skrzypczak Colorectal 2 : colon adenoma
SRC
BCL6
P−value:0.075; Pearson R:−0.59
Skrzypczak Colorectal 2 : colon adenoma
YES1
BCL6
P−value:0.84; Pearson R:0.072
Skrzypczak Colorectal 2 : colon adenoma
SRC
ZBTB7A
P−value:0.94; Pearson R:−0.027
Skrzypczak Colorectal 2 : colon adenoma
YES1
ZBTB7A
P−value:0.6; Pearson R:−0.19

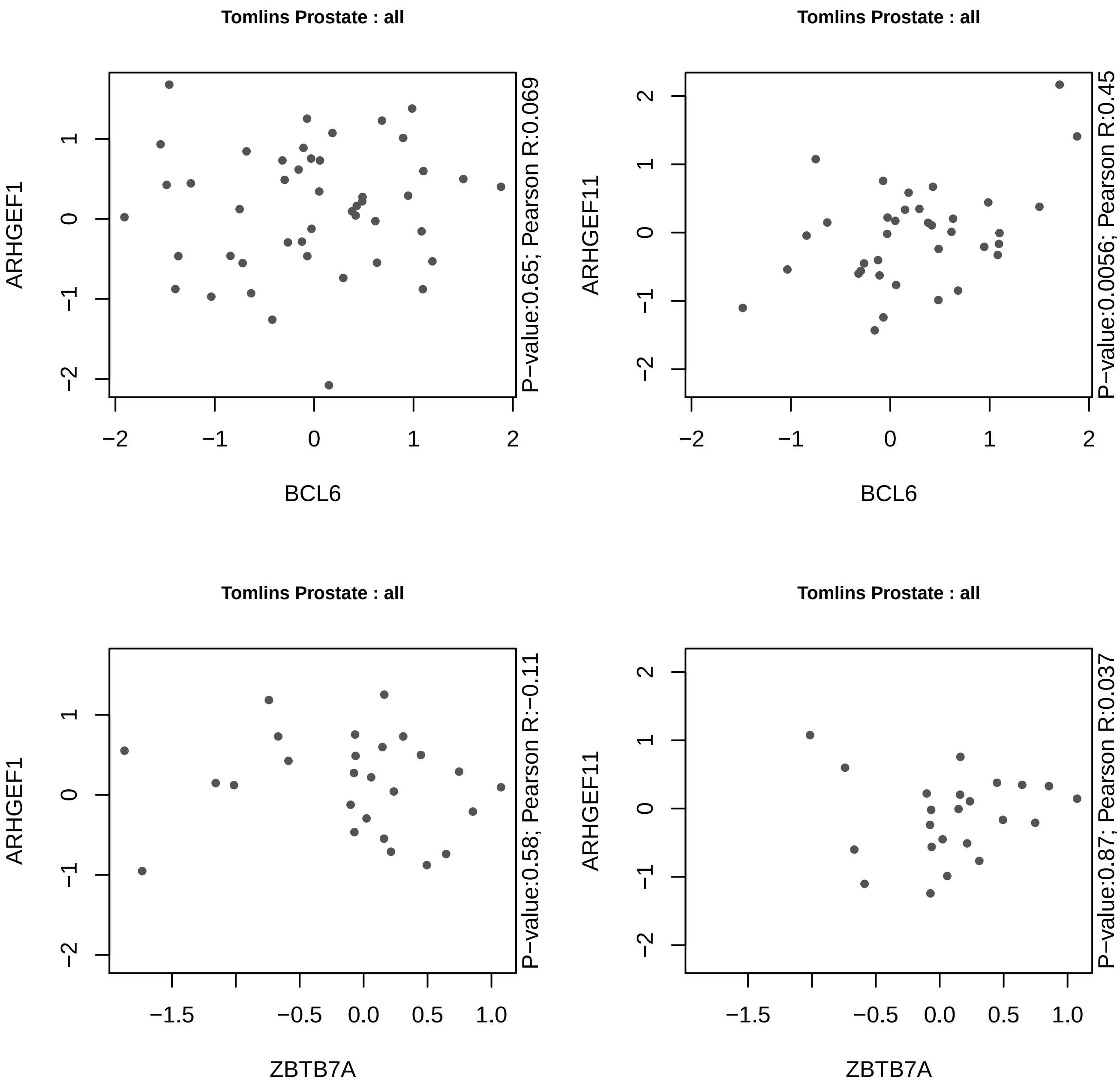
Tomlins Prostate : all
ARHGEF1
BCL6
P−value:0.65; Pearson R:0.069
Tomlins Prostate : all
ARHGEF11
BCL6
P−value:0.0056; Pearson R:0.45
Tomlins Prostate : all
ARHGEF1
ZBTB7A
P−value:0.58; Pearson R:−0.11
Tomlins Prostate : all
ARHGEF11
ZBTB7A
P−value:0.87; Pearson R:0.037

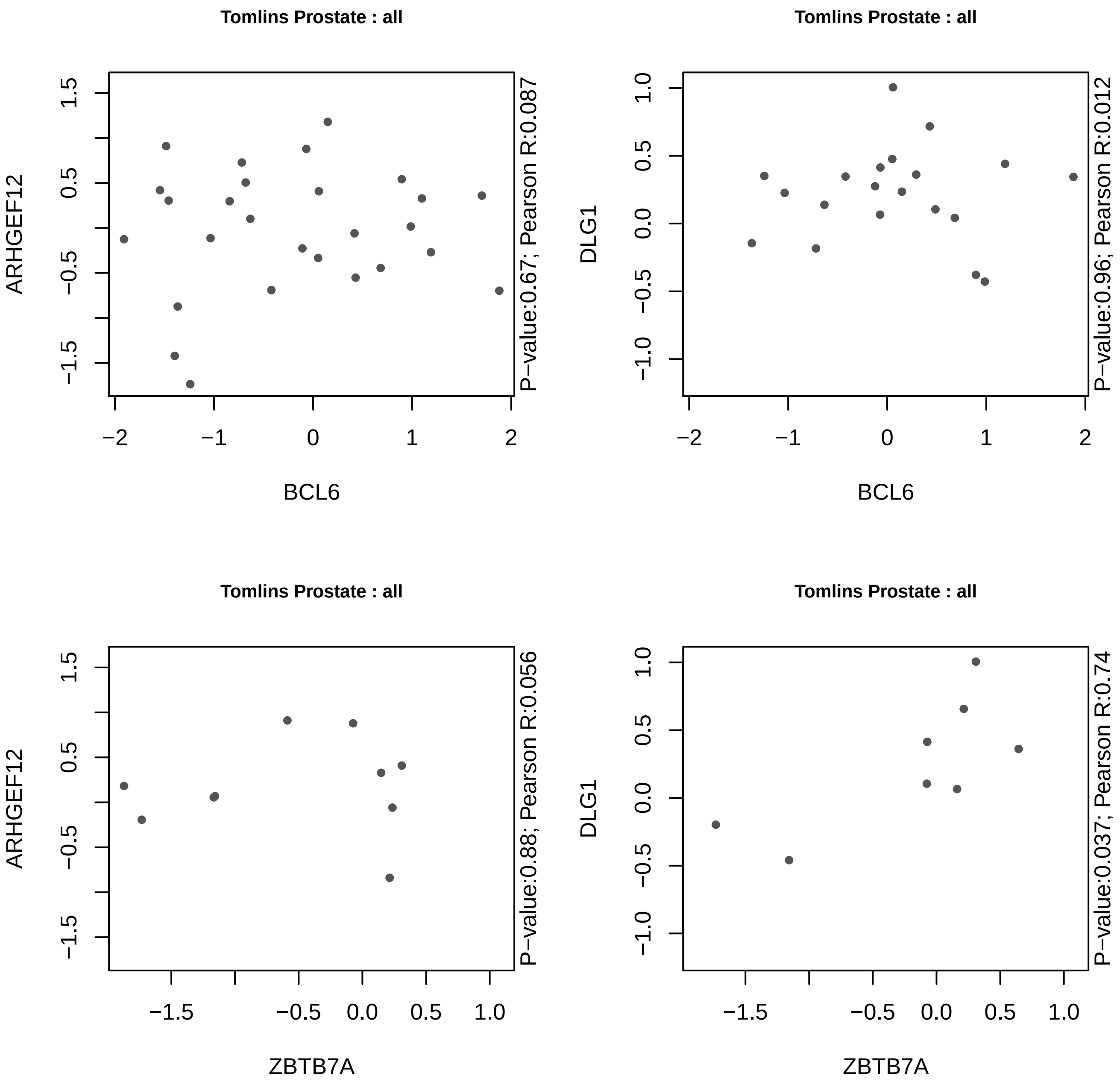
Tomlins Prostate : all
ARHGEF12
BCL6
P−value:0.67; Pearson R:0.087
Tomlins Prostate : all
DLG1
BCL6
P−value:0.96; Pearson R:0.012
Tomlins Prostate : all
ARHGEF12
ZBTB7A
P−value:0.88; Pearson R:0.056
Tomlins Prostate : all
DLG1
ZBTB7A
P−value:0.037; Pearson R:0.74

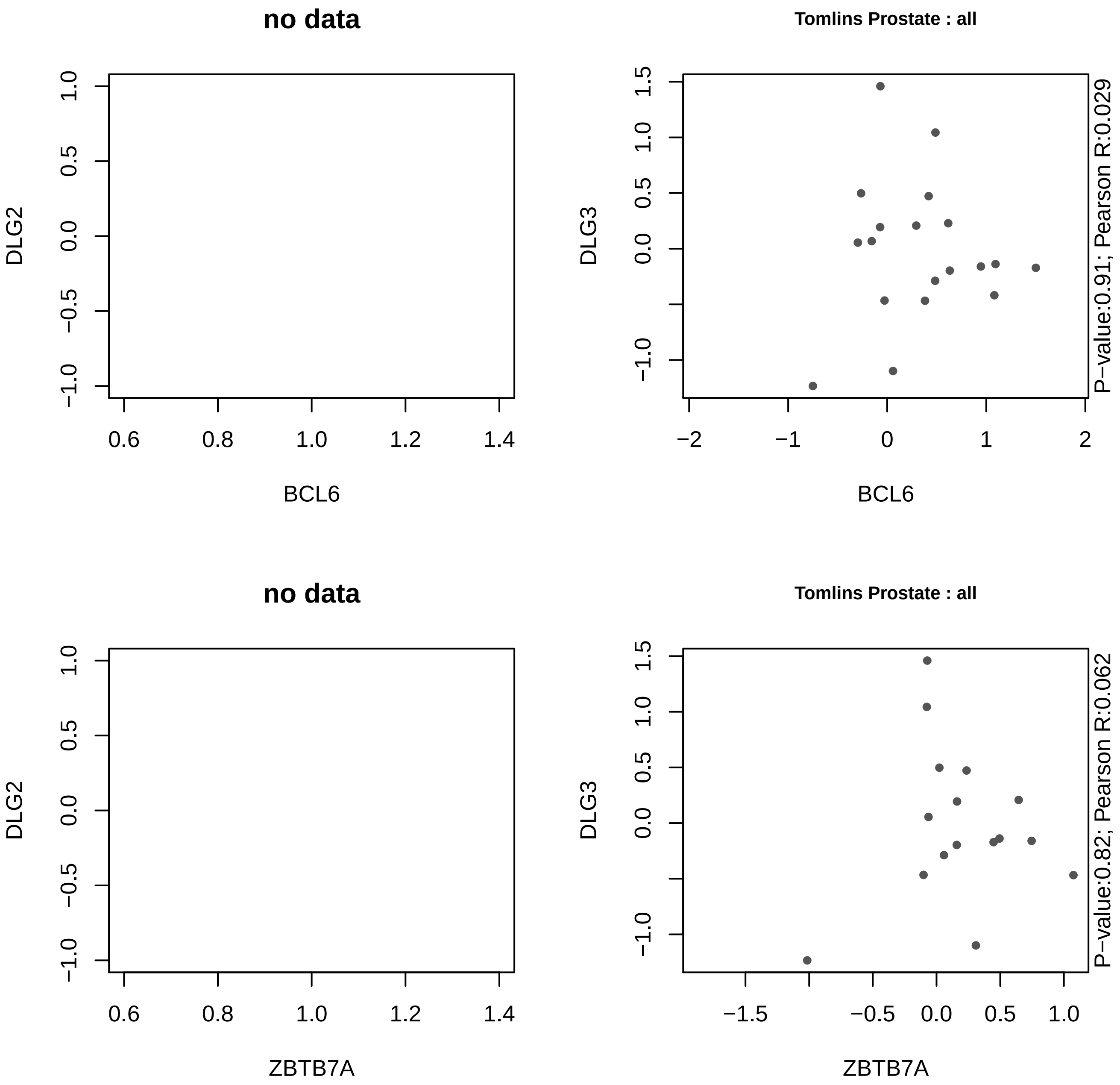
no data
DLG2
BCL6
Tomlins Prostate : all
DLG3
BCL6
P−value:0.91; Pearson R:0.029
no data
DLG2
ZBTB7A
Tomlins Prostate : all
DLG3
ZBTB7A
P−value:0.82; Pearson R:0.062

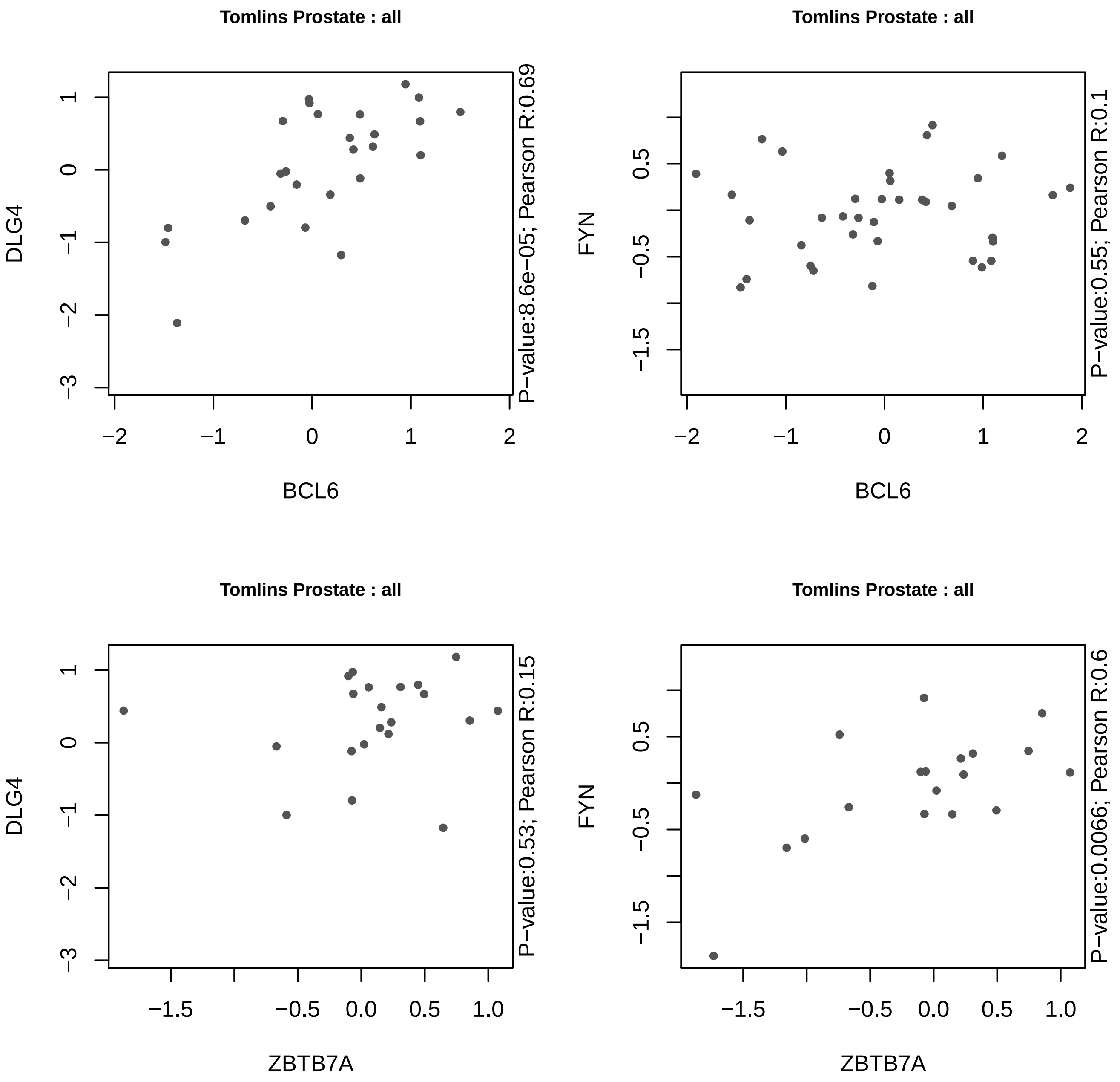
Tomlins Prostate : all
DLG4
BCL6
P−value:8.6e−05; Pearson R:0.69
Tomlins Prostate : all
FYN
BCL6
P−value:0.55; Pearson R:0.1
Tomlins Prostate : all
DLG4
ZBTB7A
P−value:0.53; Pearson R:0.15
Tomlins Prostate : all
FYN
ZBTB7A
P−value:0.0066; Pearson R:0.6

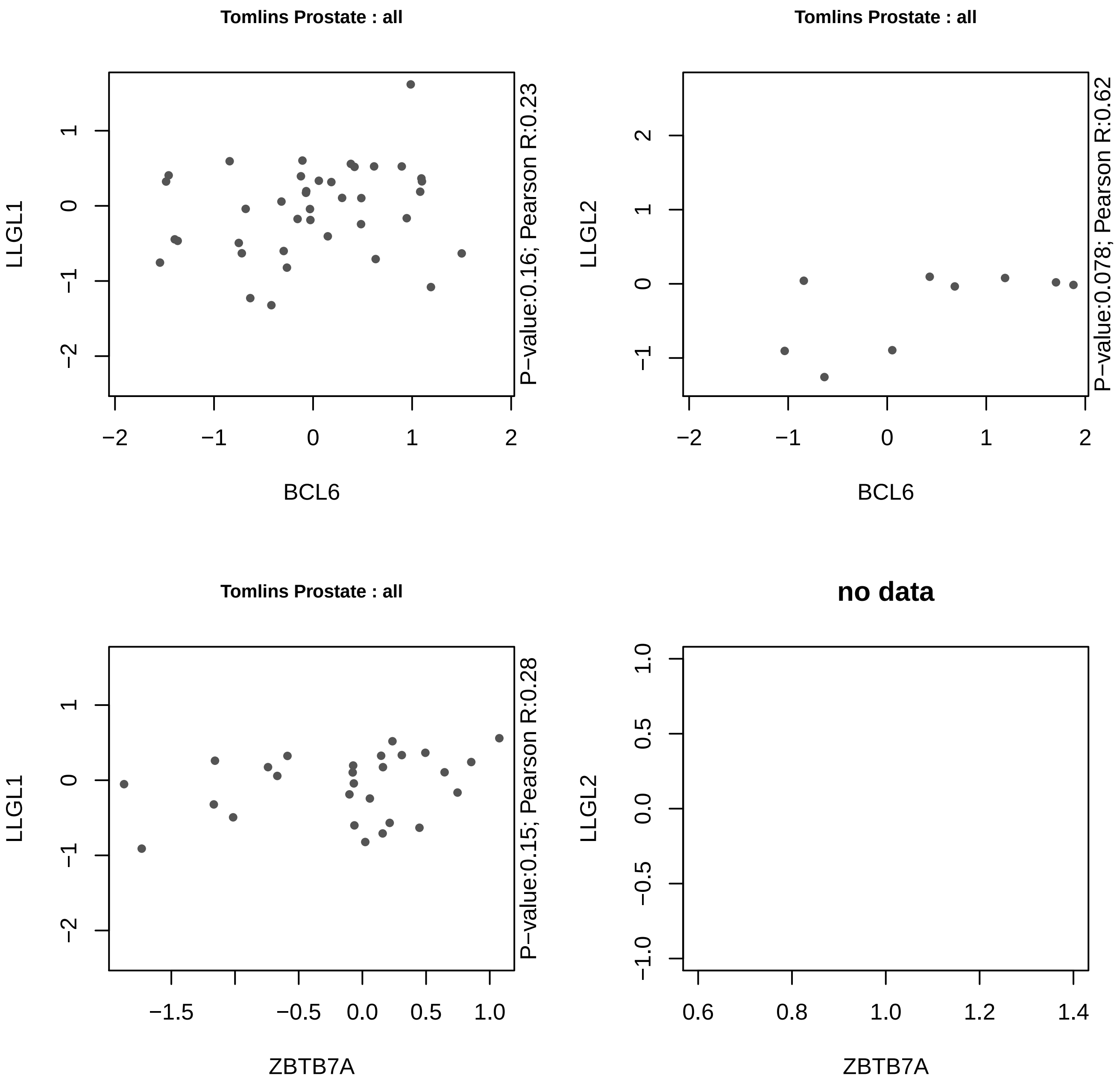
Tomlins Prostate : all
LLGL1
BCL6
P−value:0.16; Pearson R:0.23
Tomlins Prostate : all
LLGL2
BCL6
P−value:0.078; Pearson R:0.62
Tomlins Prostate : all
LLGL1
ZBTB7A
P−value:0.15; Pearson R:0.28
no data
LLGL2
ZBTB7A

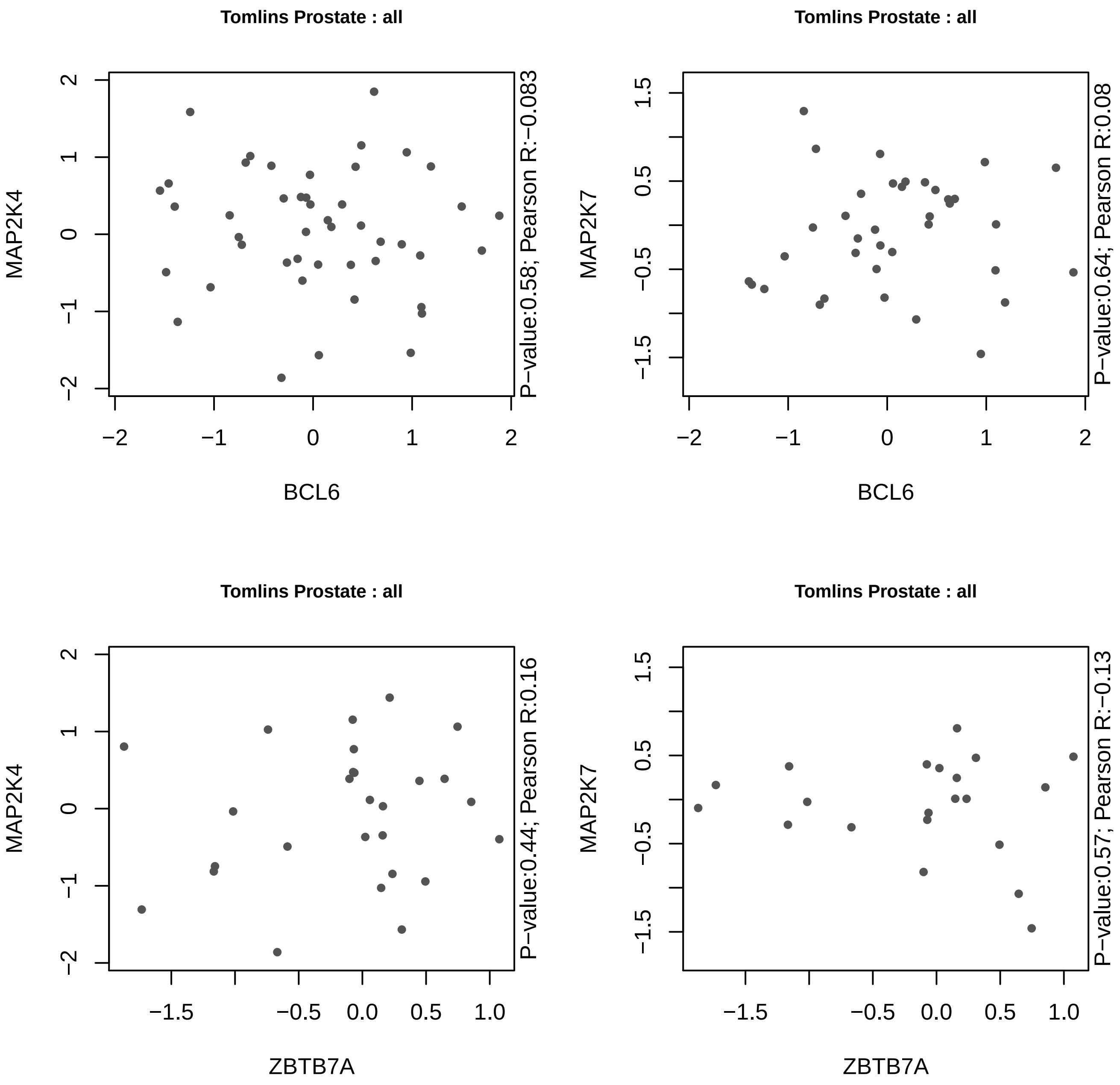
Tomlins Prostate : all
MAP2K4
BCL6
P−value:0.58; Pearson R:−0.083
Tomlins Prostate : all
MAP2K7
BCL6
P−value:0.64; Pearson R:0.08
Tomlins Prostate : all
MAP2K4
ZBTB7A
P−value:0.44; Pearson R:0.16
Tomlins Prostate : all
MAP2K7
ZBTB7A
P−value:0.57; Pearson R:−0.13

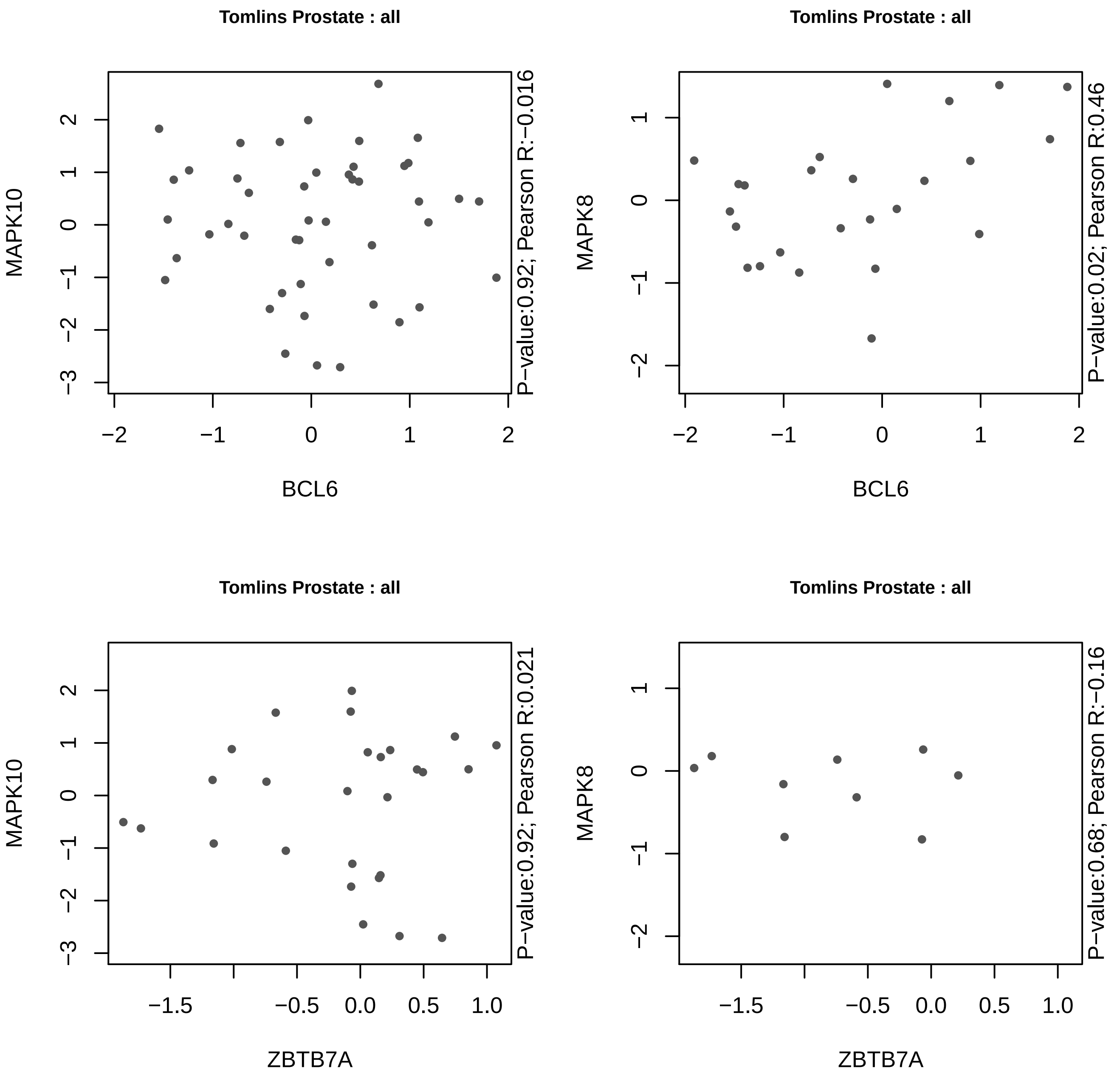

Tomlins Prostate : all
MAPK10
BCL6
P−value:0.92; Pearson R:−0.016
Tomlins Prostate : all
MAPK8
BCL6
P−value:0.02; Pearson R:0.46
Tomlins Prostate : all
MAPK10
ZBTB7A
P−value:0.92; Pearson R:0.021
Tomlins Prostate : all
MAPK8
ZBTB7A
P−value:0.68; Pearson R:−0.16

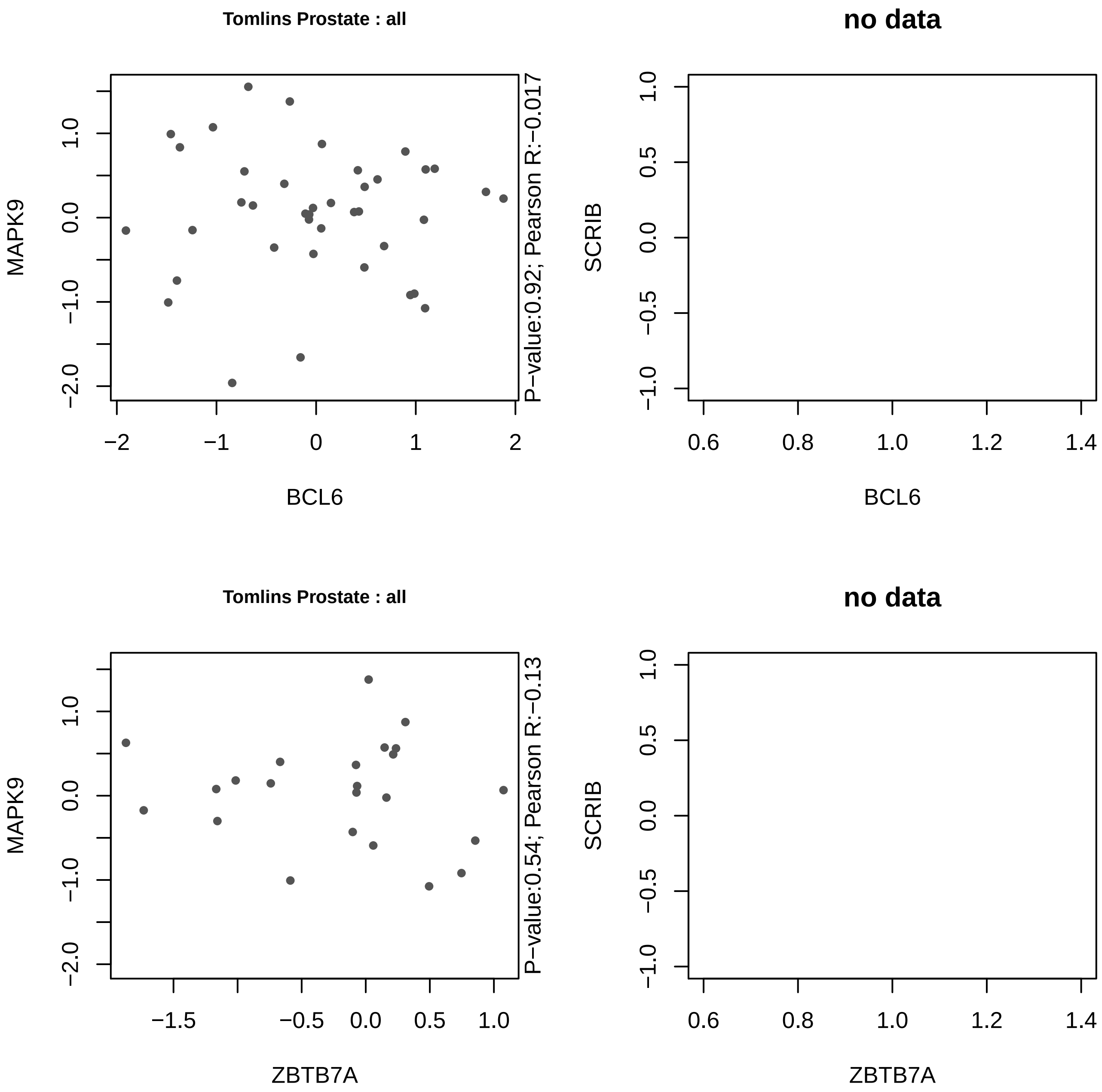
Tomlins Prostate : all
MAPK9
BCL6
P−value:0.92; Pearson R:−0.017
−2.0 −1.0 0.0 1.0
−2 −1 0 1 2
no data
SCRIB
BCL6
−1.0 −0.5 0.0 0.5 1.0
0.6 0.8 1.0 1.2 1.4
Tomlins Prostate : all
MAPK9
ZBTB7A
P−value:0.54; Pearson R:−0.13
−2.0 −1.0 0.0 1.0
−1.5 −0.5 0.0 0.5 1.0
no data
SCRIB
ZBTB7A
−1.0 −0.5 0.0 0.5 1.0
0.6 0.8 1.0 1.2 1.4

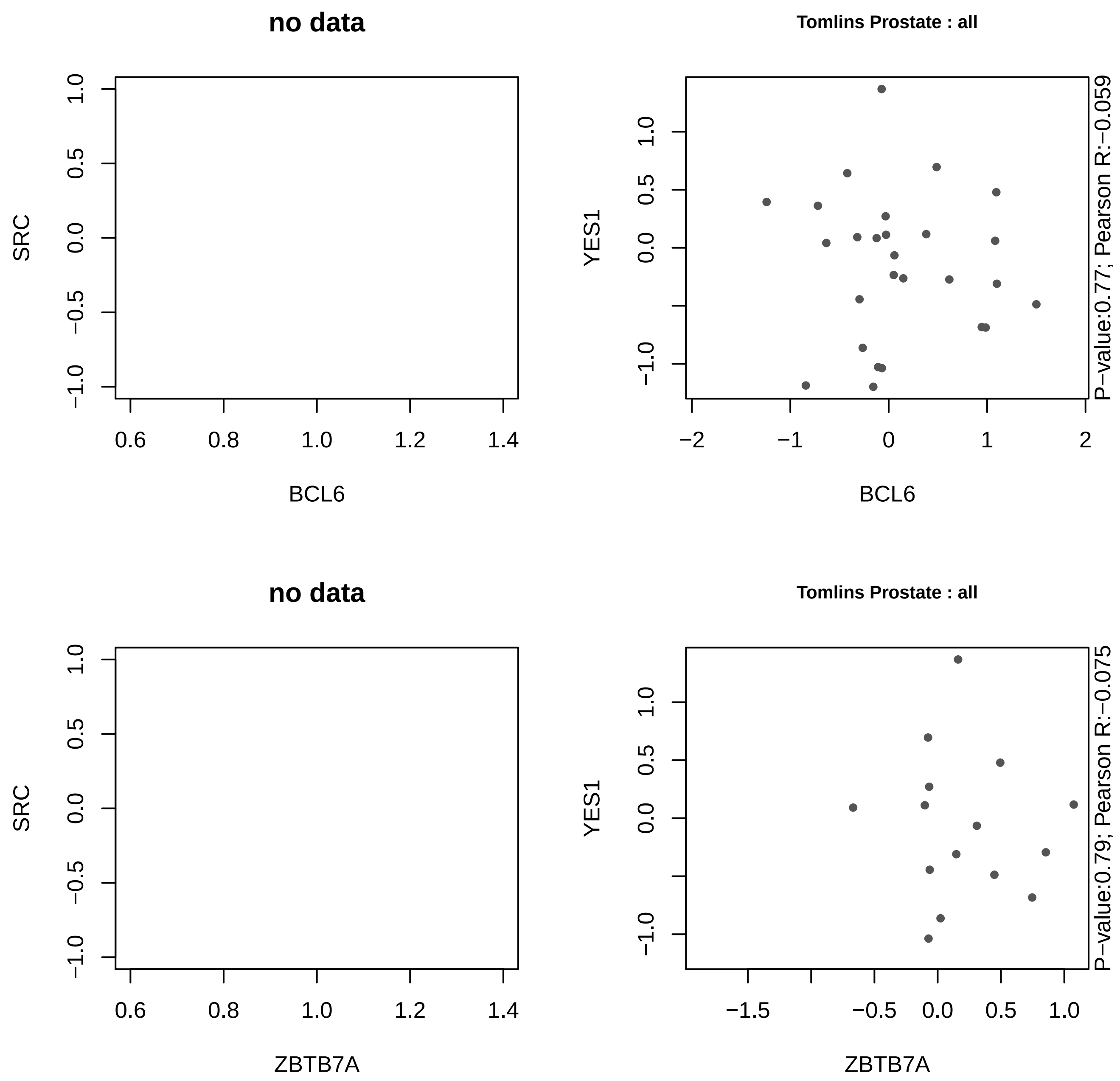
no data
SRC
BCL6
Tomlins Prostate : all
YES1
BCL6
P−value:0.77; Pearson R:−0.059
no data
SRC
ZBTB7A
Tomlins Prostate : all
YES1
ZBTB7A
P−value:0.79; Pearson R:−0.075

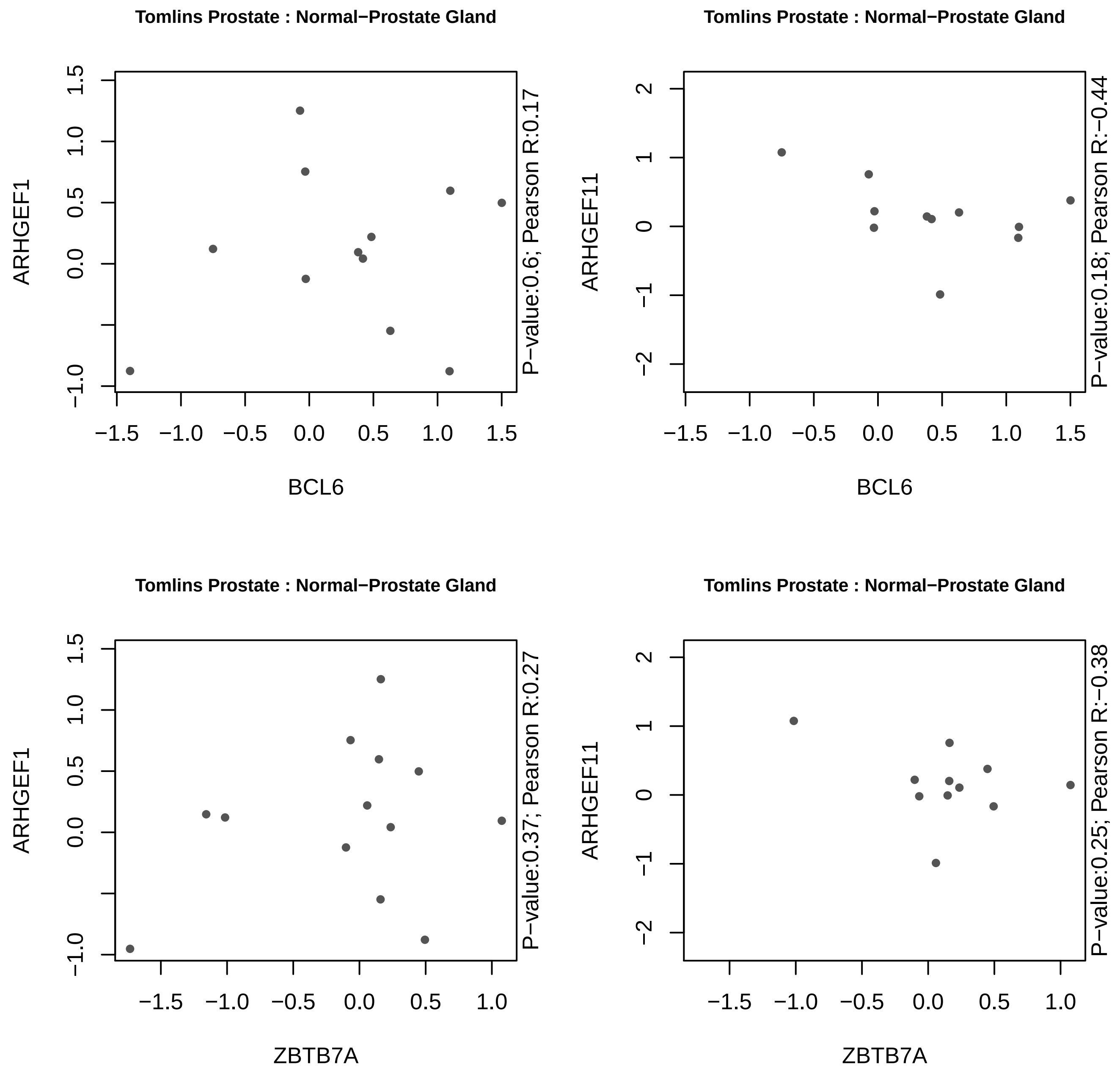
Tomlins Prostate : Normal−Prostate Gland
ARHGEF1
BCL6
P−value:0.6; Pearson R:0.17
Tomlins Prostate : Normal−Prostate Gland
ARHGEF11
BCL6
P−value:0.18; Pearson R:−0.44
Tomlins Prostate : Normal−Prostate Gland
ARHGEF1
ZBTB7A
P−value:0.37; Pearson R:0.27
Tomlins Prostate : Normal−Prostate Gland
ARHGEF11
ZBTB7A
P−value:0.25; Pearson R:−0.38

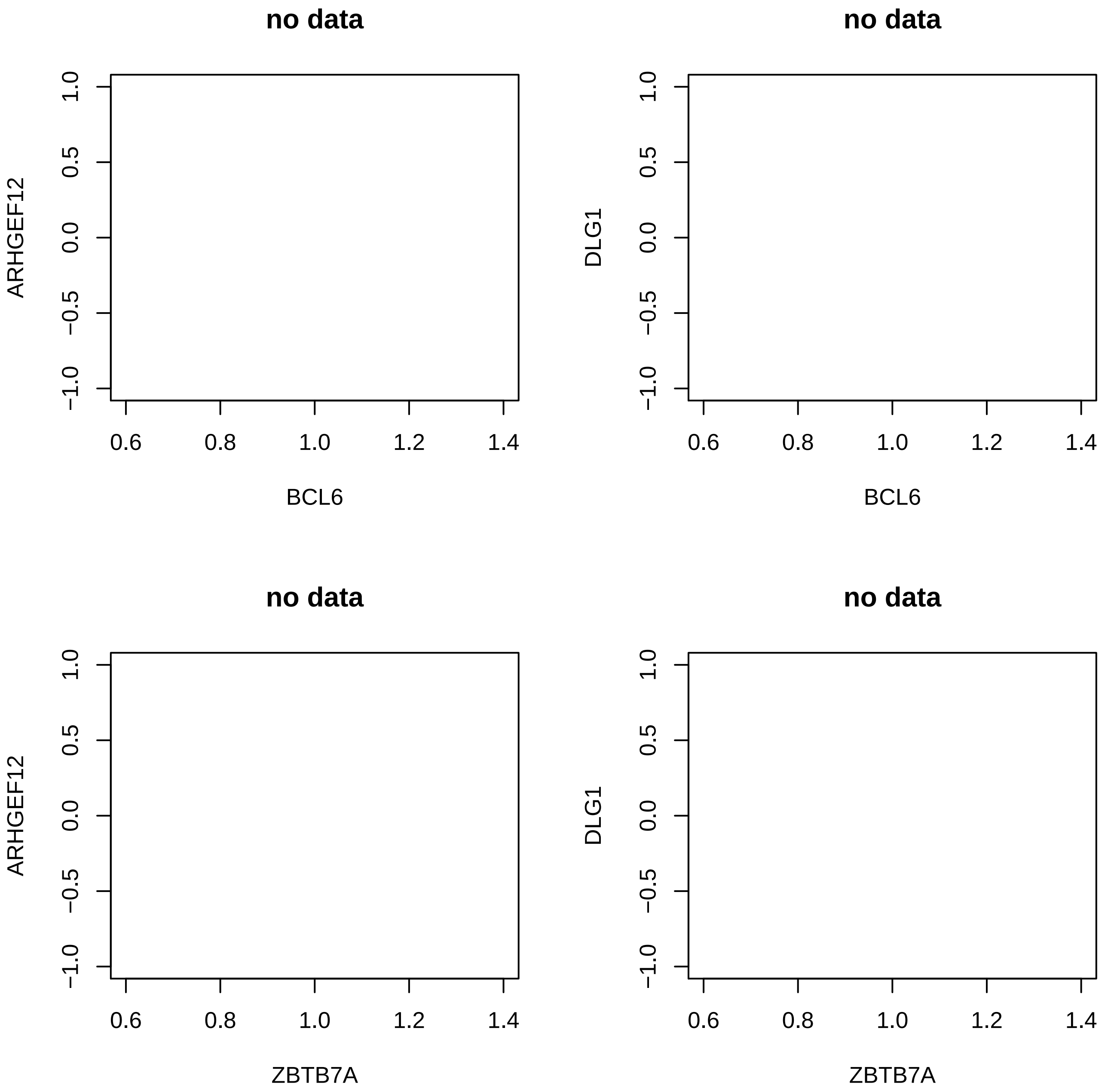
no data
ARHGEF12
1.0
0.5
0.0
−0.5
−1.0
0.6
0.8
1.0
1.2
1.4
BCL6
no data
DLG1
BCL6
no data
ARHGEF12
ZBTB7A
no data
DLG1
ZBTB7A

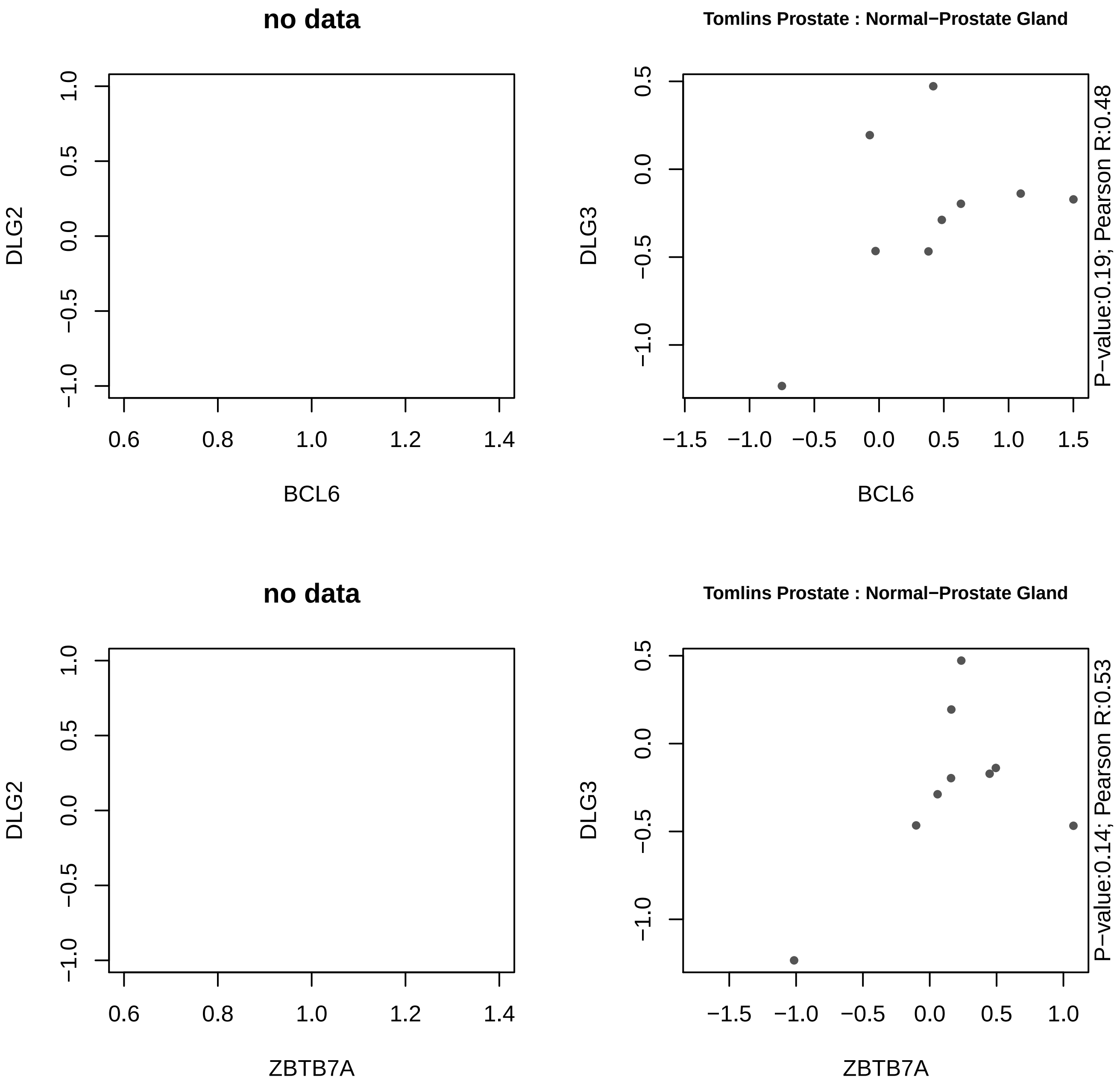
no data
DLG2
BCL6
Tomlins Prostate : Normal−Prostate Gland
DLG3
BCL6
P−value:0.19; Pearson R:0.48
no data
DLG2
ZBTB7A
Tomlins Prostate : Normal−Prostate Gland
DLG3
ZBTB7A
P−value:0.14; Pearson R:0.53

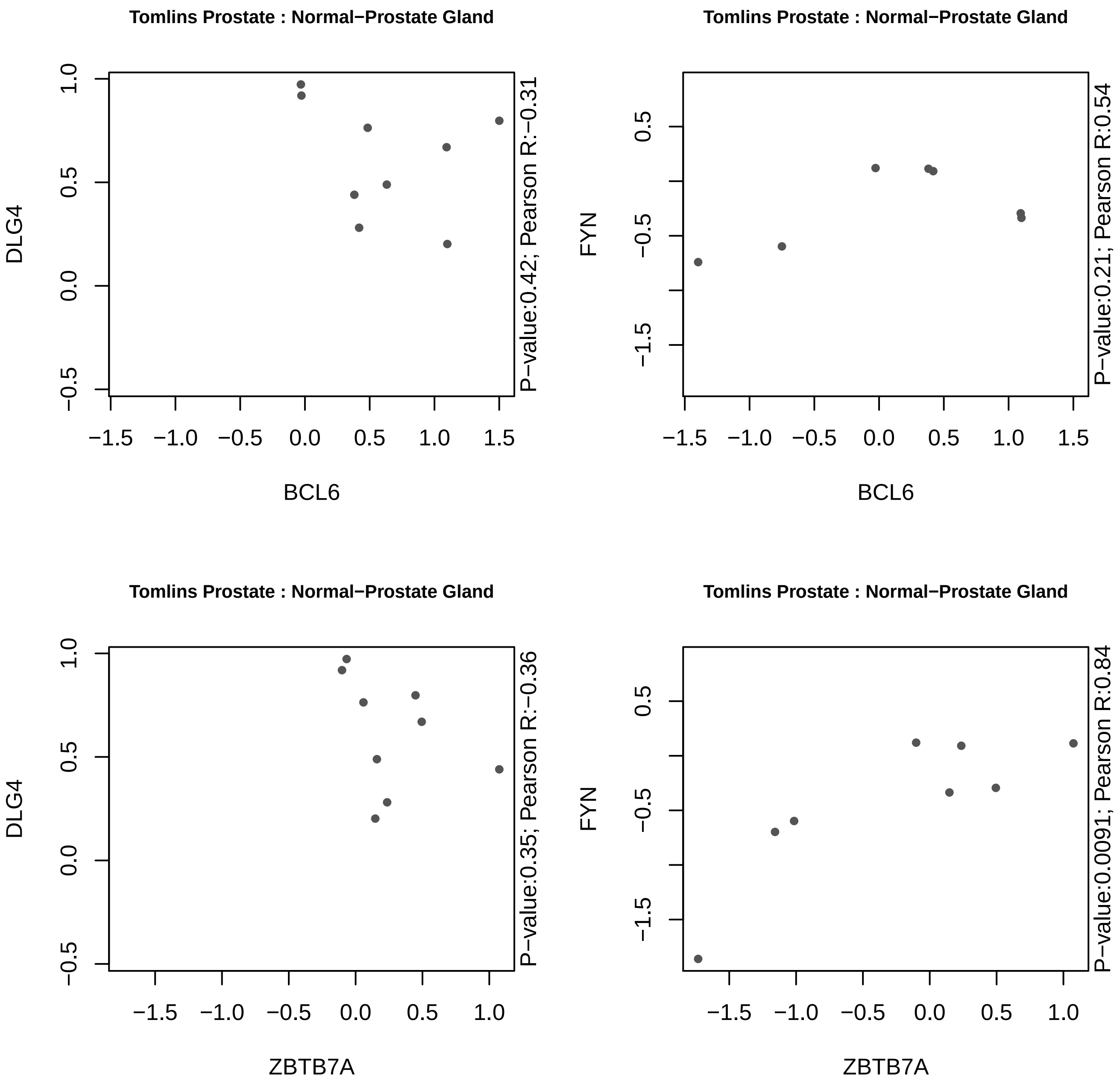
Tomlins Prostate : Normal−Prostate Gland
DLG4
BCL6
P−value:0.42; Pearson R:−0.31
Tomlins Prostate : Normal−Prostate Gland
FYN
BCL6
P−value:0.21; Pearson R:0.54
Tomlins Prostate : Normal−Prostate Gland
DLG4
ZBTB7A
P−value:0.35; Pearson R:−0.36
Tomlins Prostate : Normal−Prostate Gland
FYN
ZBTB7A
P−value:0.0091; Pearson R:0.84

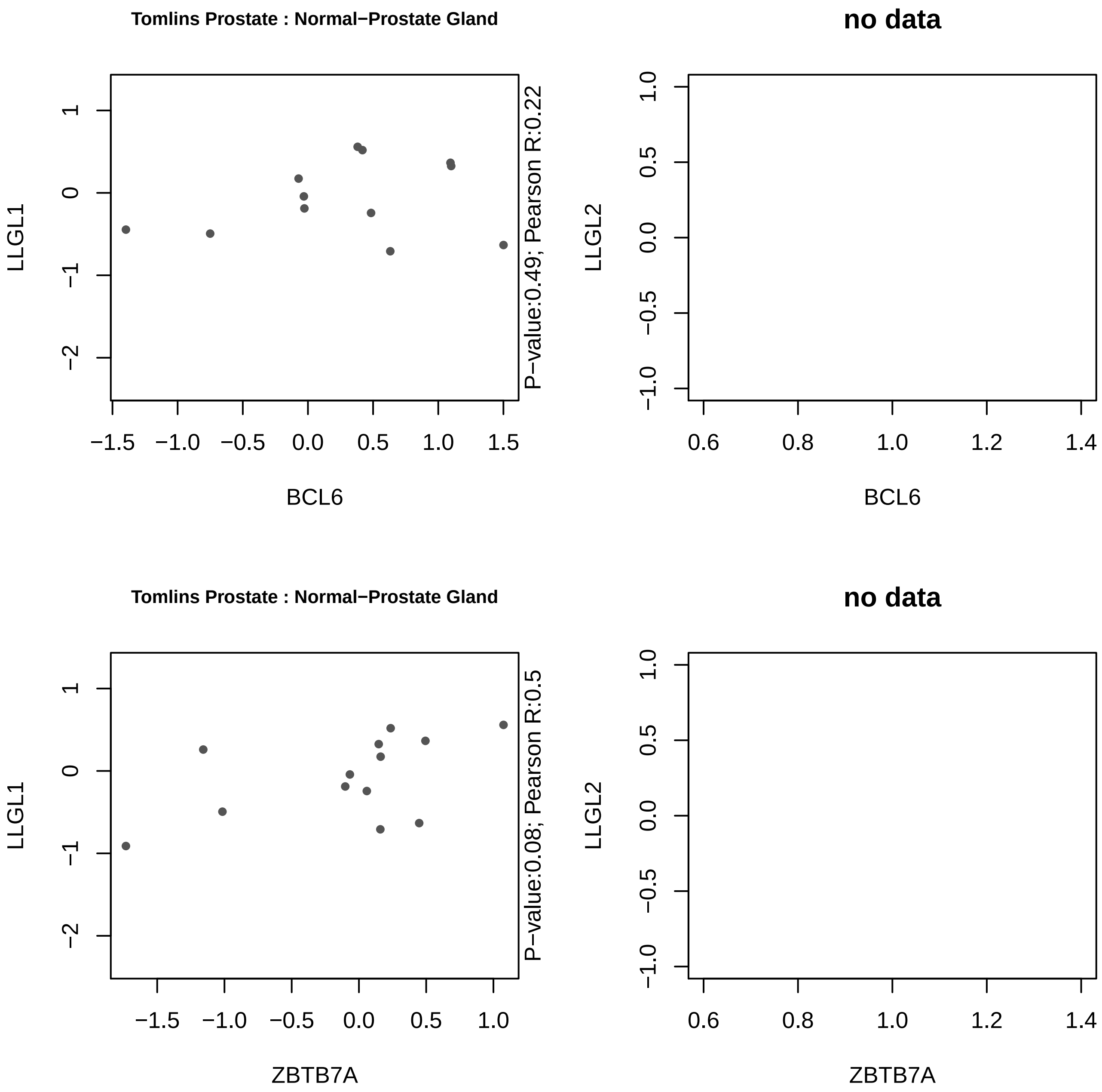
Tomlins Prostate : Normal−Prostate Gland
LLGL1
BCL6
P−value:0.49; Pearson R:0.22
no data
LLGL2
BCL6
Tomlins Prostate : Normal−Prostate Gland
LLGL1
ZBTB7A
P−value:0.08; Pearson R:0.5
no data
LLGL2
ZBTB7A

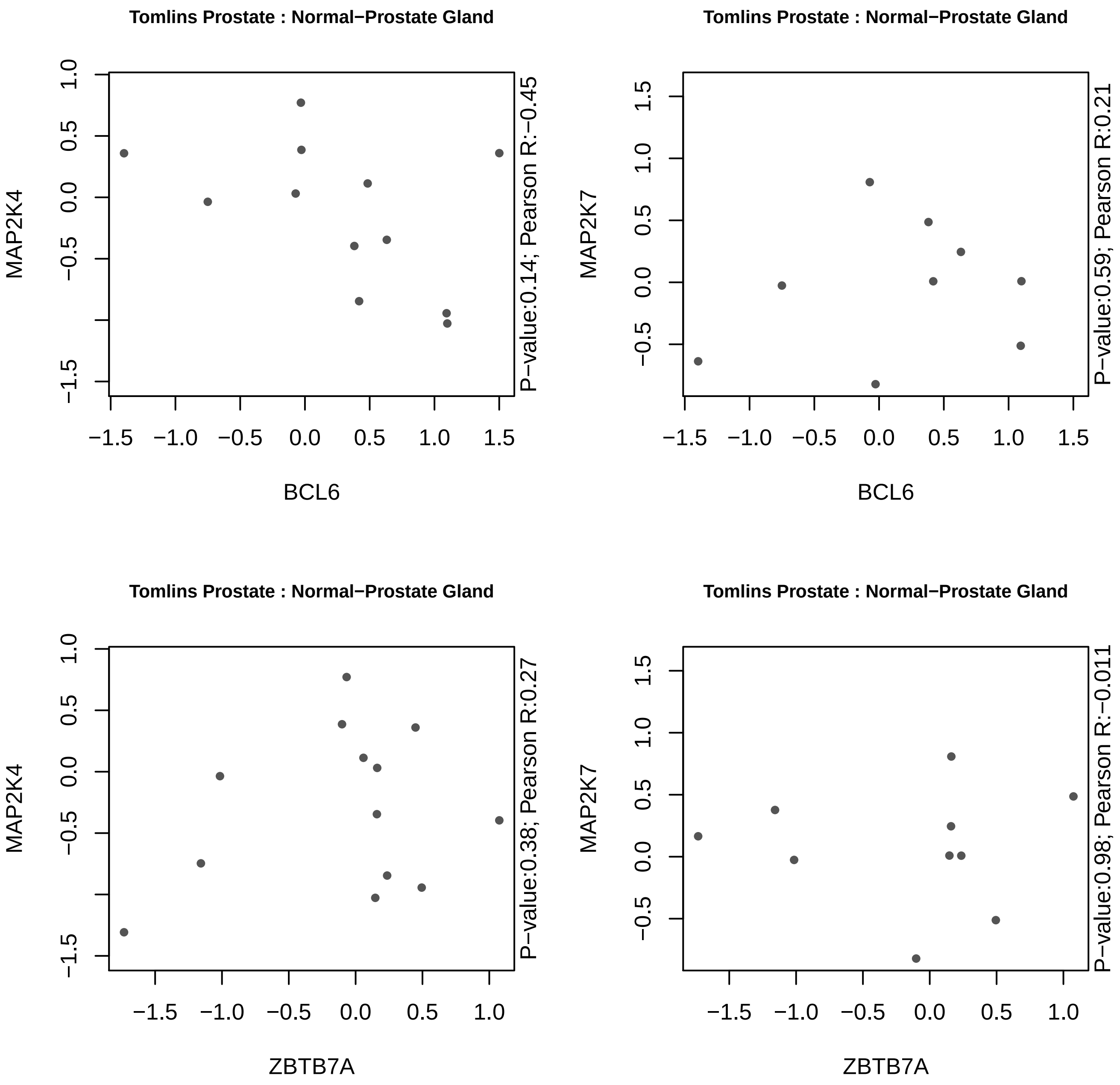
Tomlins Prostate : Normal−Prostate Gland
MAP2K4
BCL6
P−value:0.14; Pearson R:−0.45
Tomlins Prostate : Normal−Prostate Gland
MAP2K7
BCL6
P−value:0.59; Pearson R:0.21
Tomlins Prostate : Normal−Prostate Gland
MAP2K4
ZBTB7A
P−value:0.38; Pearson R:0.27
Tomlins Prostate : Normal−Prostate Gland
MAP2K7
ZBTB7A
P−value:0.98; Pearson R:−0.011

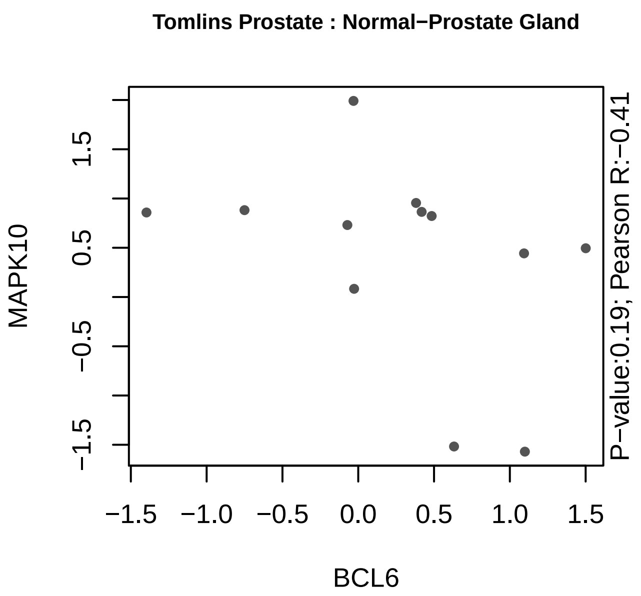
Tomlins Prostate : Normal−Prostate Gland
MAPK10
BCL6
P−value:0.19; Pearson R:−0.41
1.5
0.5
−0.5
−1.5
−1.5 −1.0 −0.5 0.0 0.5 1.0 1.5

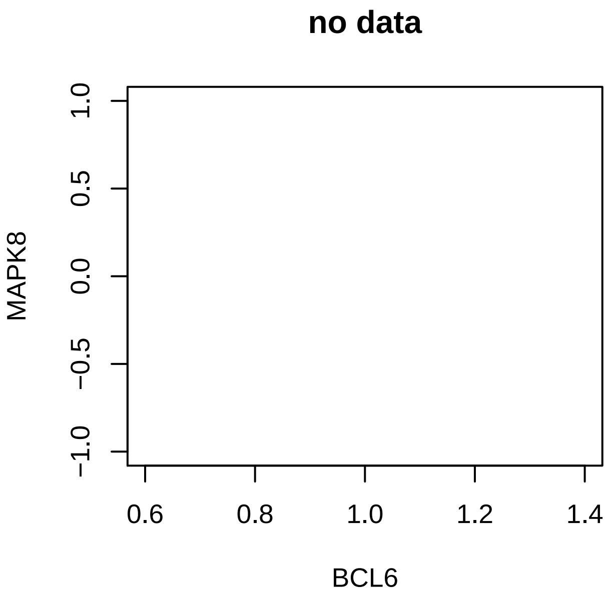
no data
MAPK8
BCL6
1.0
0.5
0.0
−0.5
−1.0
0.6 0.8 1.0 1.2 1.4

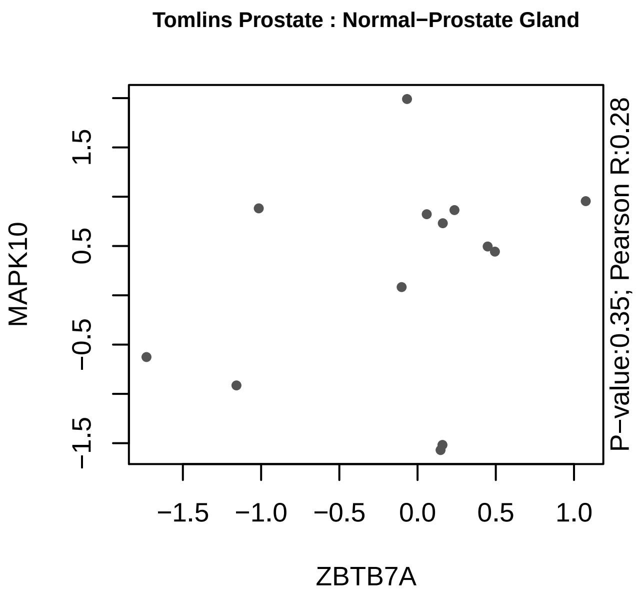
Tomlins Prostate : Normal−Prostate Gland
MAPK10
ZBTB7A
P−value:0.35; Pearson R:0.28
1.5
0.5
−0.5
−1.5
−1.5 −1.0 −0.5 0.0 0.5 1.0

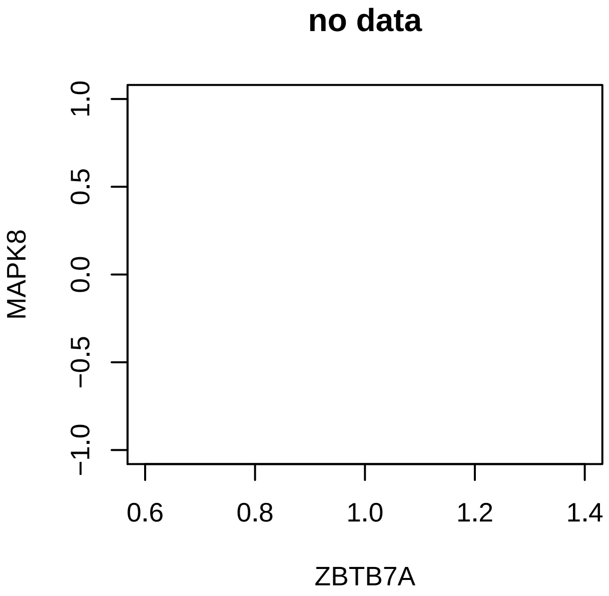
no data
MAPK8
ZBTB7A
1.0
0.5
0.0
−0.5
−1.0
0.6 0.8 1.0 1.2 1.4

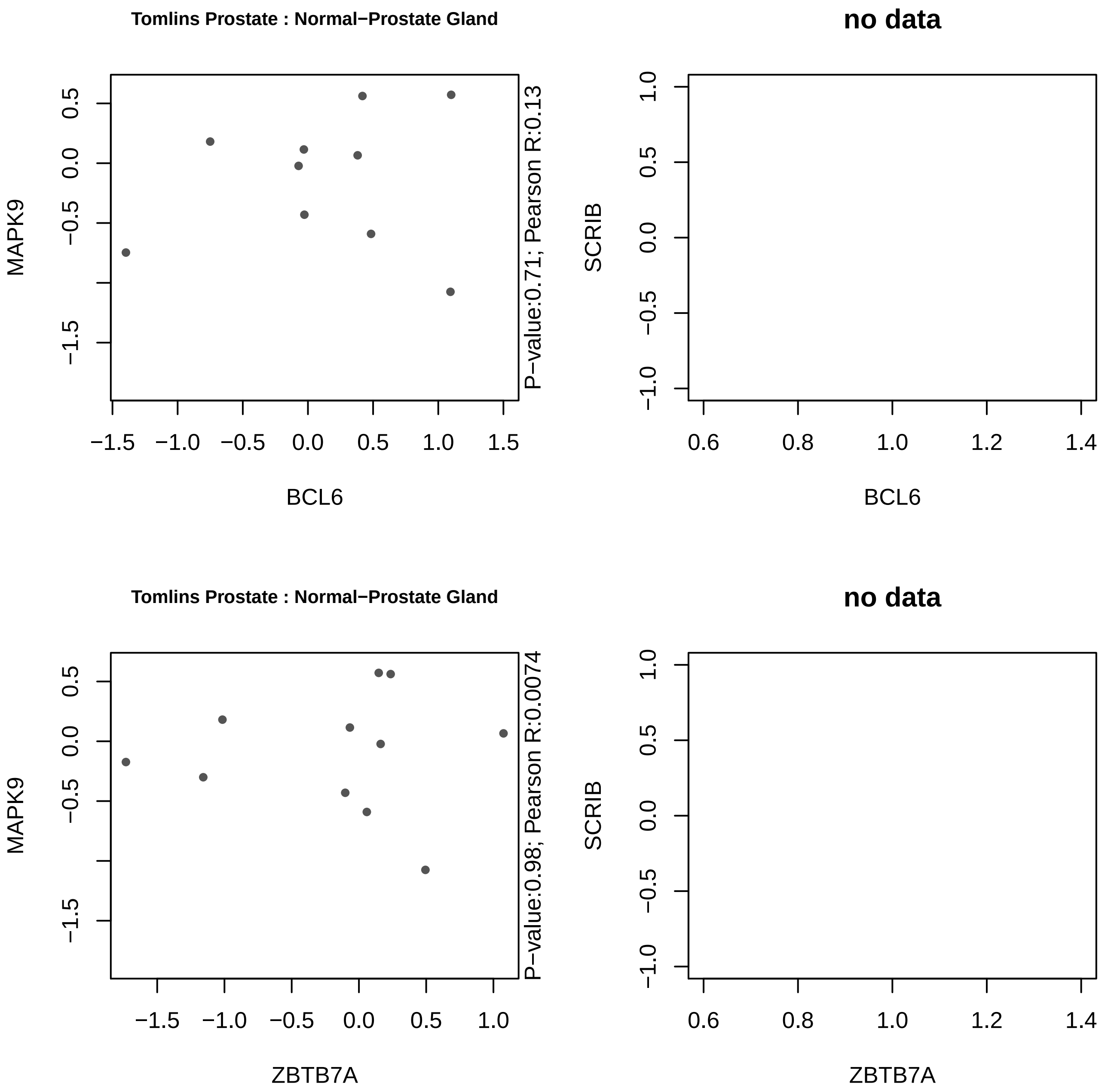
Tomlins Prostate : Normal−Prostate Gland
MAPK9
BCL6
P−value:0.71; Pearson R:0.13
no data
SCRIB
BCL6
Tomlins Prostate : Normal−Prostate Gland
MAPK9
ZBTB7A
P−value:0.98; Pearson R:0.0074
no data
SCRIB
ZBTB7A

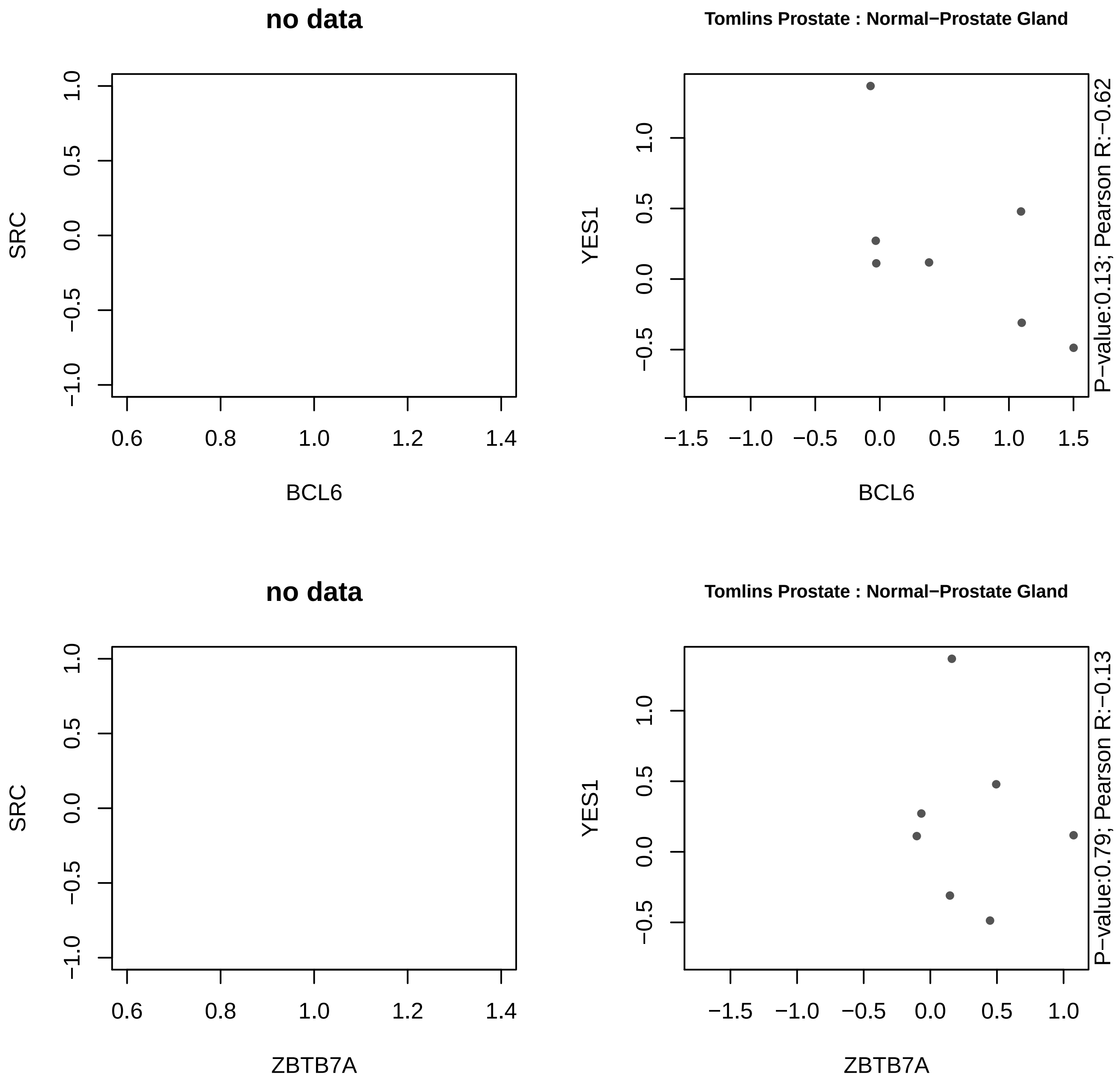
no data
SRC
BCL6
Tomlins Prostate : Normal−Prostate Gland
YES1
BCL6
P−value:0.13; Pearson R:−0.62
no data
SRC
ZBTB7A
Tomlins Prostate : Normal−Prostate Gland
YES1
ZBTB7A
P−value:0.79; Pearson R:−0.13

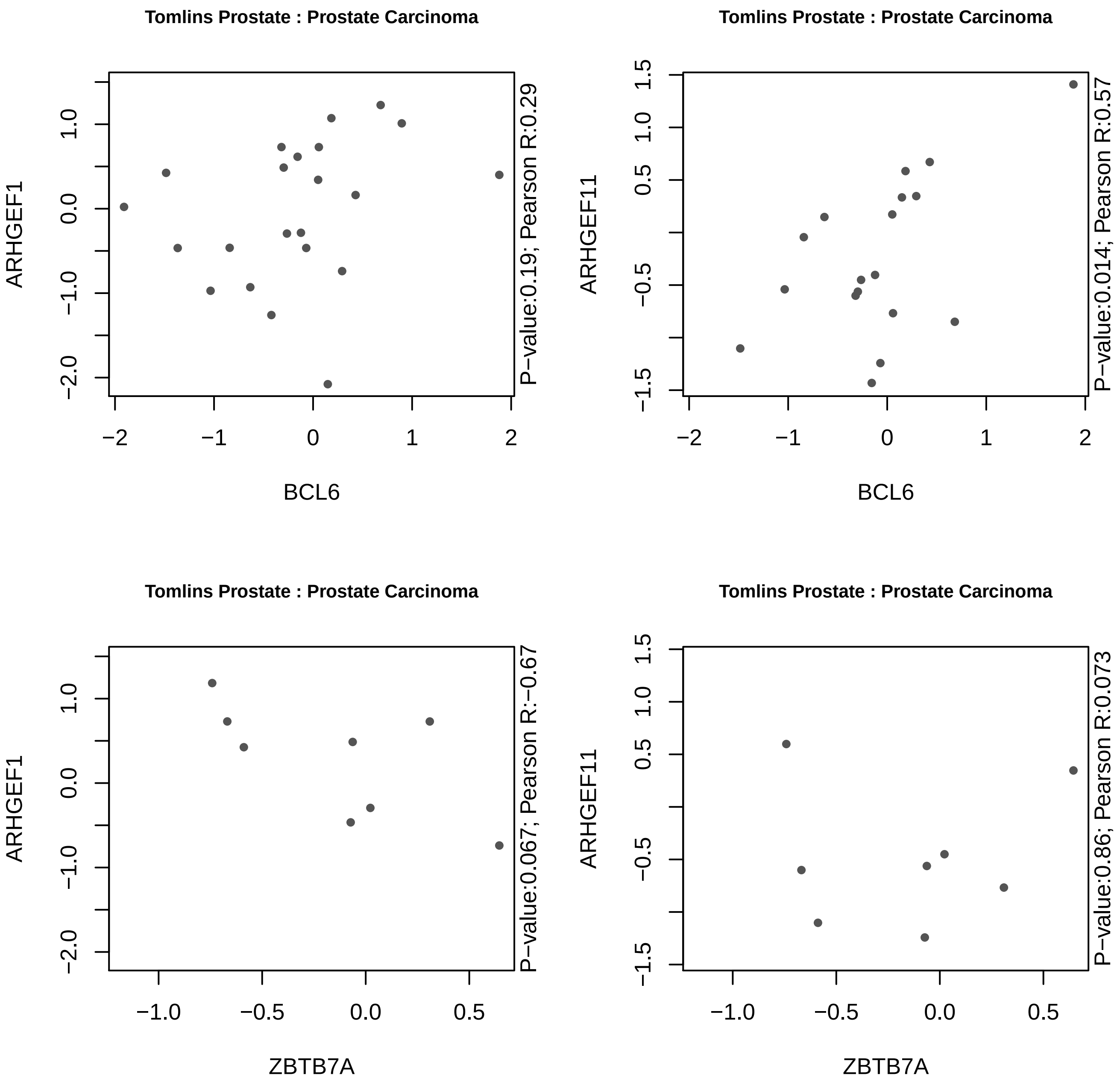
Tomlins Prostate : Prostate Carcinoma
ARHGEF1
BCL6
P−value:0.19; Pearson R:0.29
Tomlins Prostate : Prostate Carcinoma
ARHGEF11
BCL6
P−value:0.014; Pearson R:0.57
Tomlins Prostate : Prostate Carcinoma
ARHGEF1
ZBTB7A
P−value:0.067; Pearson R:−0.67
Tomlins Prostate : Prostate Carcinoma
ARHGEF11
ZBTB7A
P−value:0.86; Pearson R:0.073

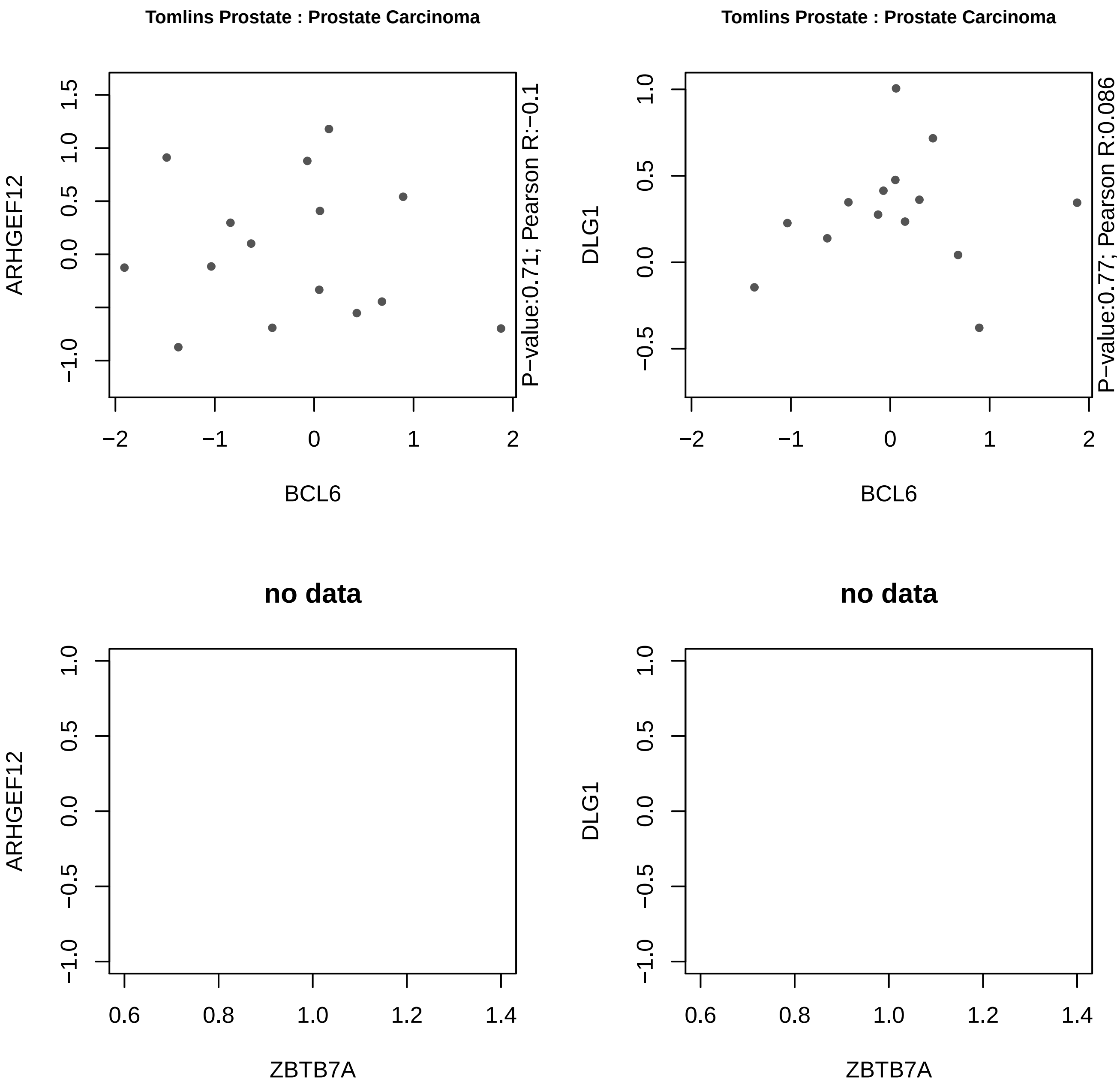
Tomlins Prostate : Prostate Carcinoma
ARHGEF12
BCL6
P−value:0.71; Pearson R:−0.1
Tomlins Prostate : Prostate Carcinoma
DLG1
BCL6
P−value:0.77; Pearson R:0.086
no data
ARHGEF12
ZBTB7A
no data
DLG1
ZBTB7A

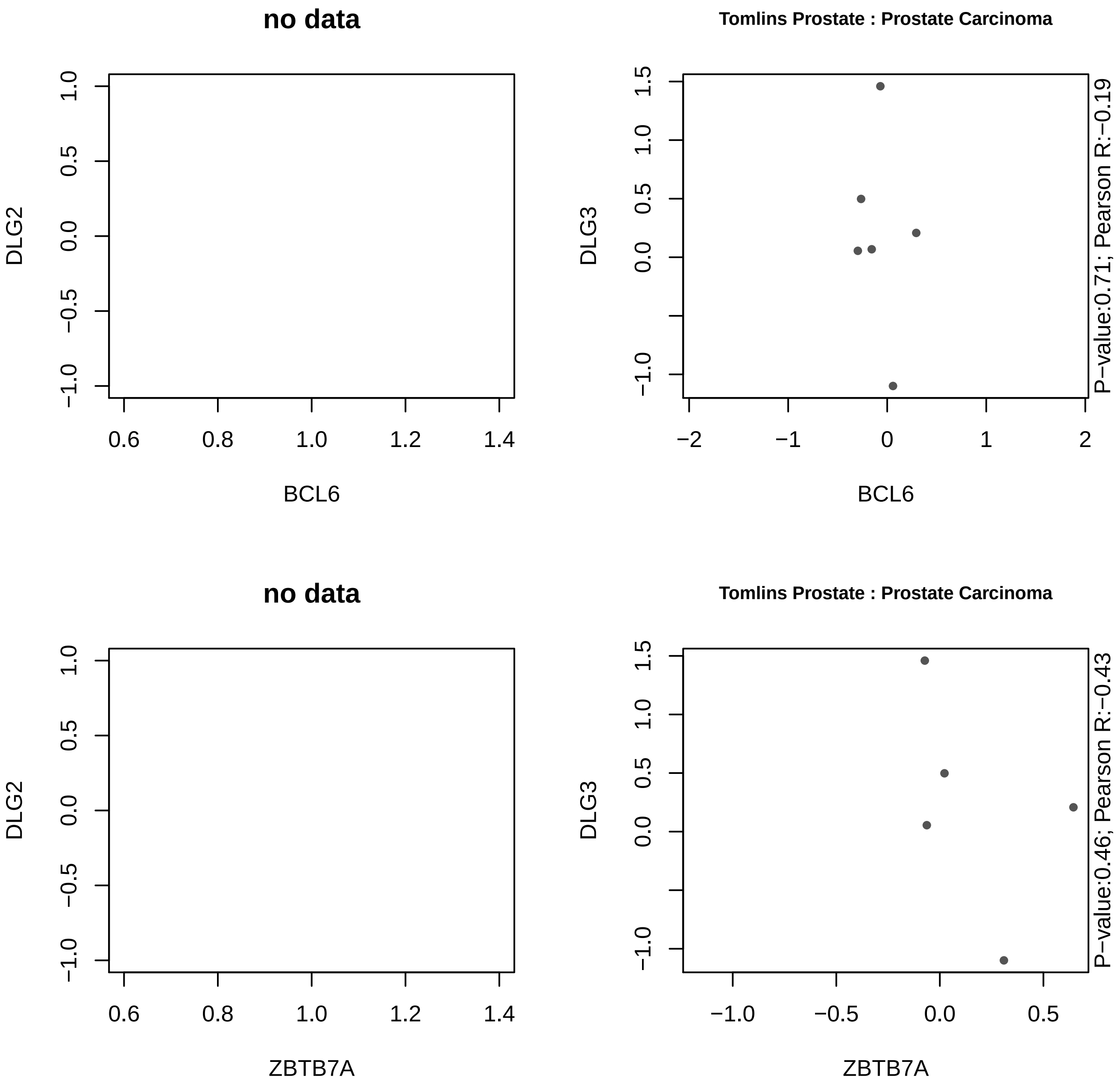
no data
DLG2
BCL6
−1.0 −0.5 0.0 0.5 1.0
0.6 0.8 1.0 1.2 1.4
Tomlins Prostate : Prostate Carcinoma
DLG3
BCL6
−1.0 0.0 0.5 1.0 1.5
−2 −1 0 1 2
P−value:0.71; Pearson R:−0.19
no data
DLG2
ZBTB7A
−1.0 −0.5 0.0 0.5 1.0
0.6 0.8 1.0 1.2 1.4
Tomlins Prostate : Prostate Carcinoma
DLG3
ZBTB7A
−1.0 0.0 0.5 1.0 1.5
−1.0 −0.5 0.0 0.5
P−value:0.46; Pearson R:−0.43

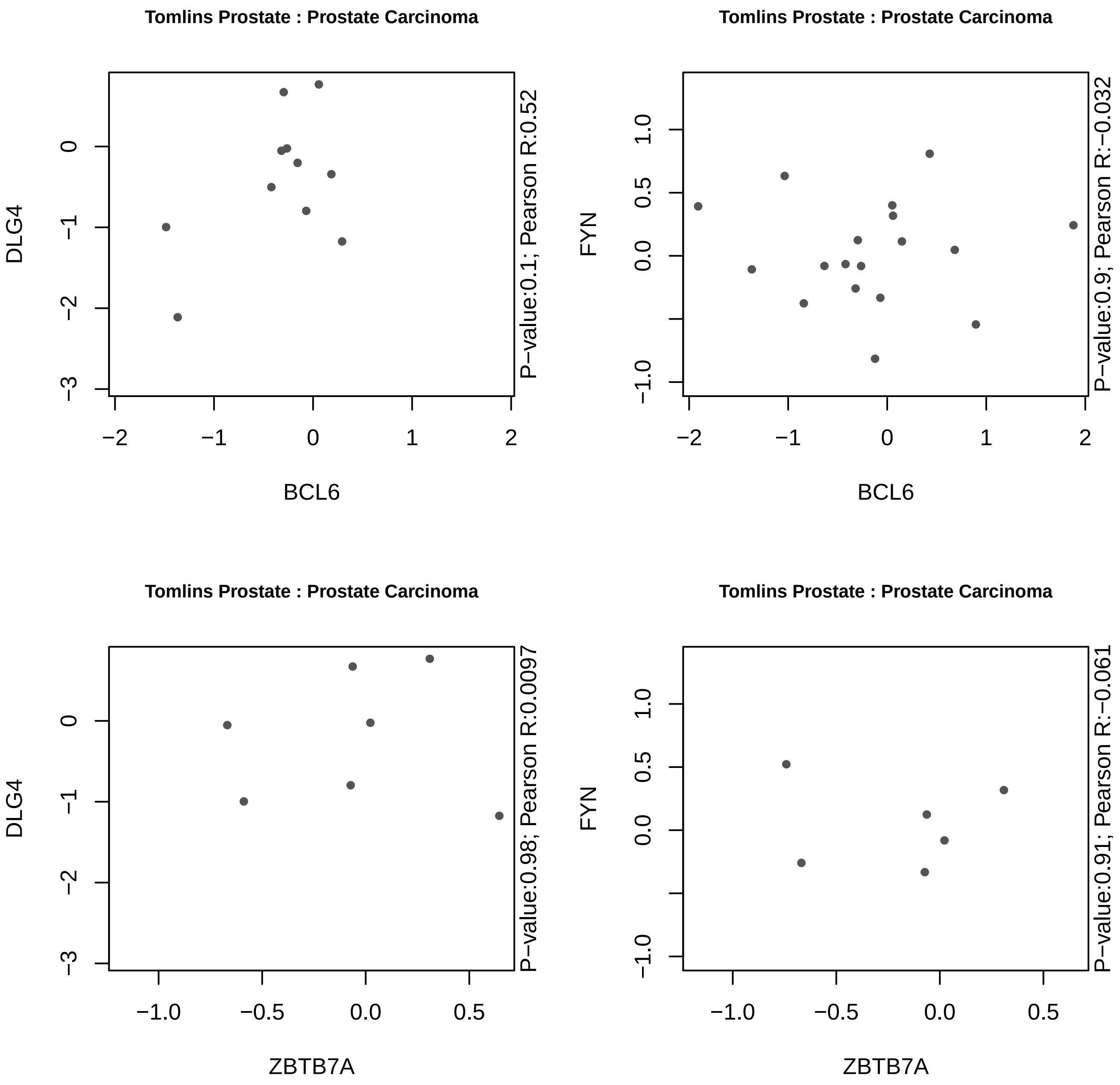
Tomlins Prostate : Prostate Carcinoma
DLG4
BCL6
P−value:0.1; Pearson R:0.52
Tomlins Prostate : Prostate Carcinoma
FYN
BCL6
P−value:0.9; Pearson R:−0.032
Tomlins Prostate : Prostate Carcinoma
DLG4
ZBTB7A
P−value:0.98; Pearson R:0.0097
Tomlins Prostate : Prostate Carcinoma
FYN
ZBTB7A
P−value:0.91; Pearson R:−0.061

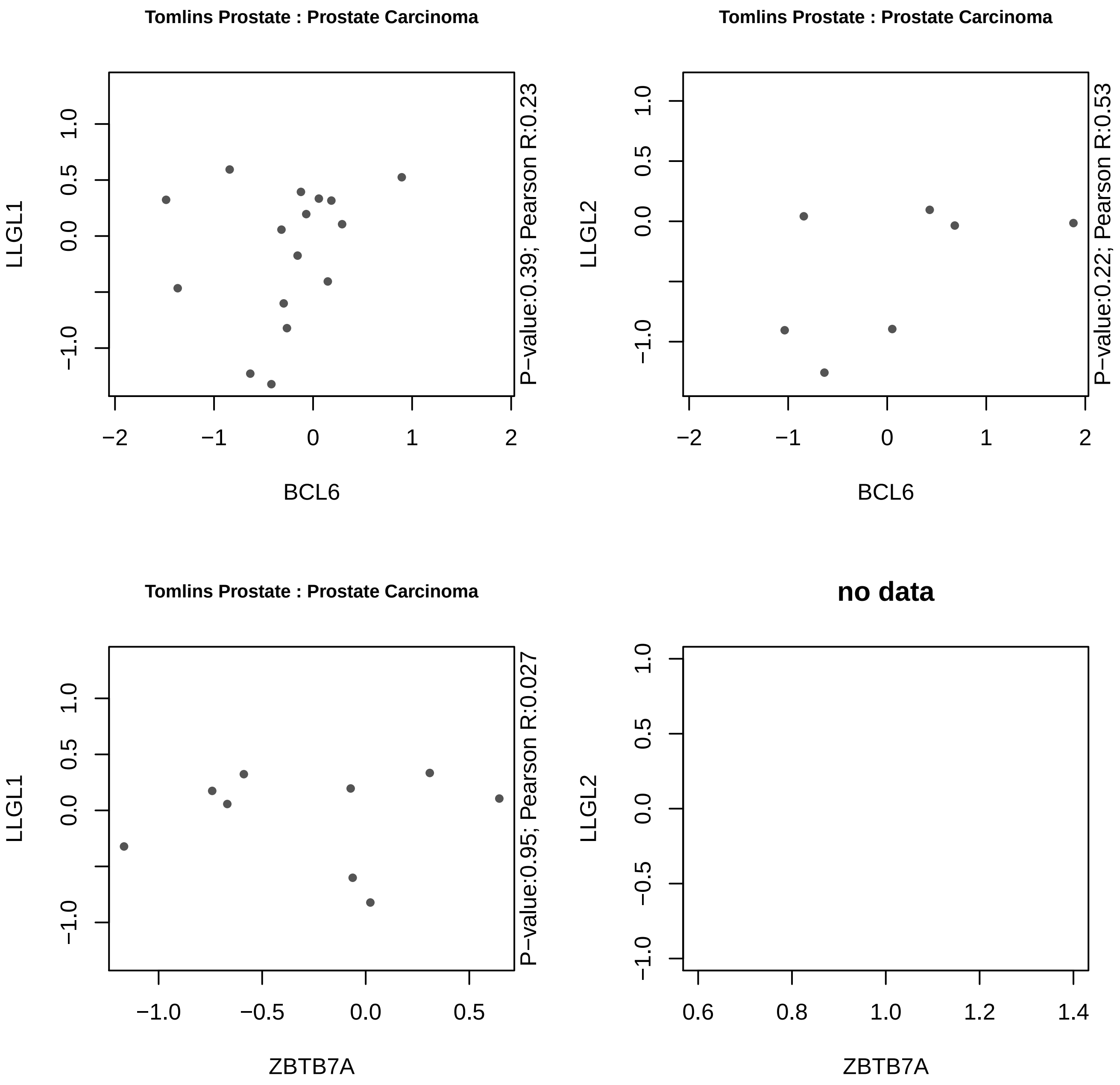
Tomlins Prostate : Prostate Carcinoma
P−value:0.39; Pearson R:0.23
LLGL1
BCL6
Tomlins Prostate : Prostate Carcinoma
P−value:0.22; Pearson R:0.53
LLGL2
BCL6
Tomlins Prostate : Prostate Carcinoma
P−value:0.95; Pearson R:0.027
LLGL1
ZBTB7A
no data
LLGL2
ZBTB7A

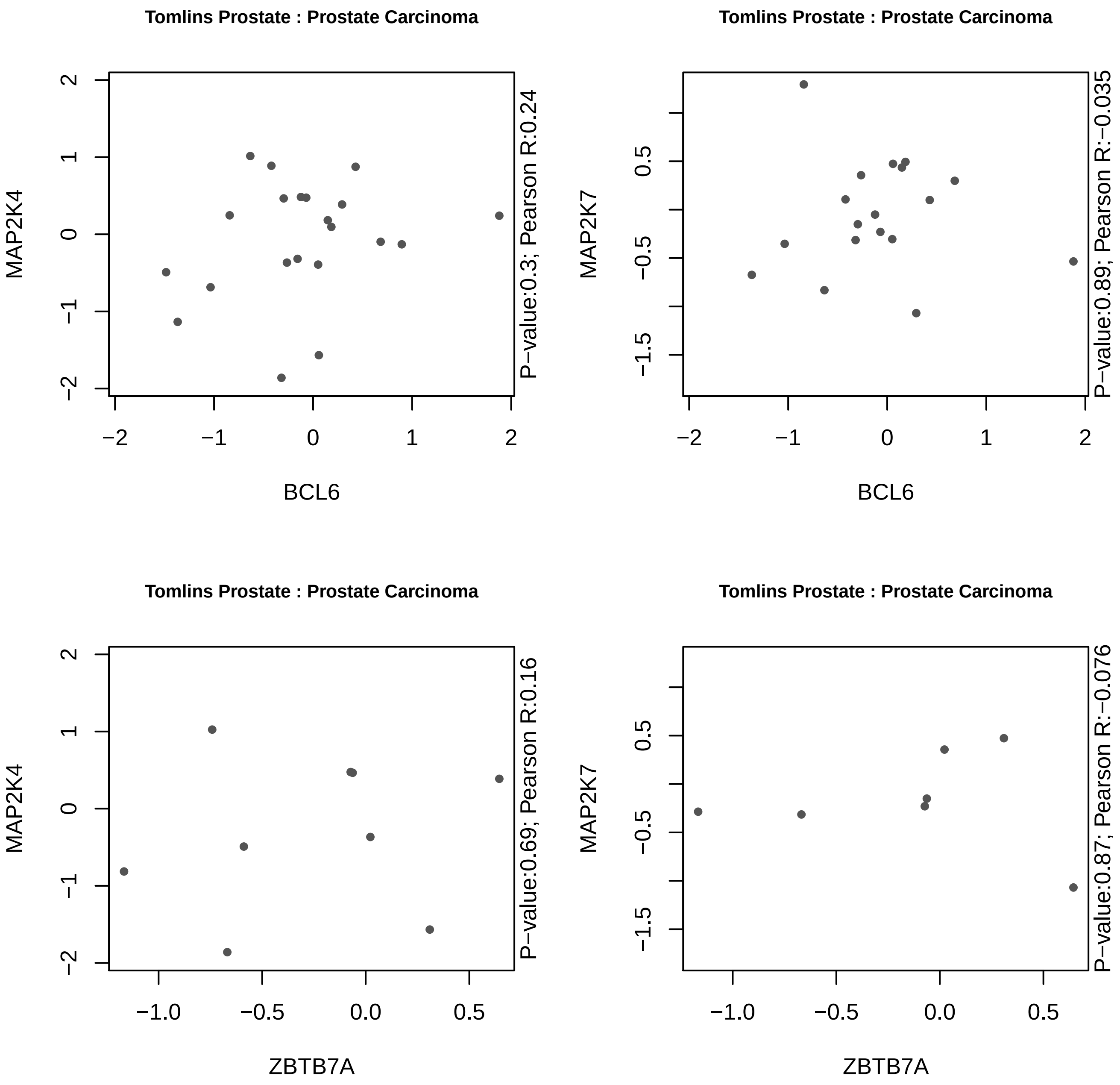
Tomlins Prostate : Prostate Carcinoma
MAP2K4
BCL6
P−value:0.3; Pearson R:0.24
Tomlins Prostate : Prostate Carcinoma
MAP2K7
BCL6
P−value:0.89; Pearson R:−0.035
Tomlins Prostate : Prostate Carcinoma
MAP2K4
ZBTB7A
P−value:0.69; Pearson R:0.16
Tomlins Prostate : Prostate Carcinoma
MAP2K7
ZBTB7A
P−value:0.87; Pearson R:−0.076

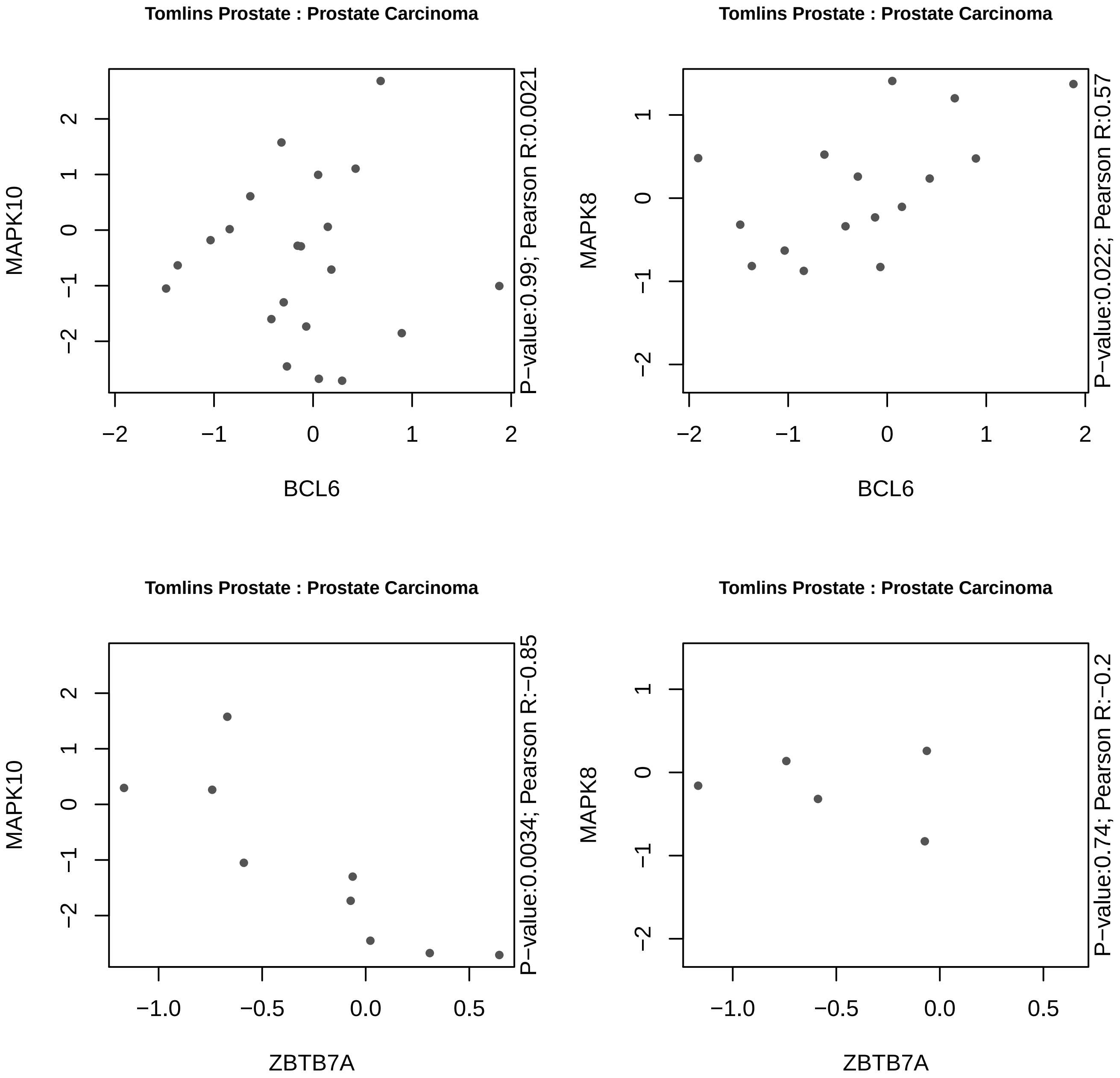
Tomlins Prostate : Prostate Carcinoma
MAPK10
BCL6
P−value:0.99; Pearson R:0.0021
Tomlins Prostate : Prostate Carcinoma
MAPK8
BCL6
P−value:0.022; Pearson R:0.57
Tomlins Prostate : Prostate Carcinoma
MAPK10
ZBTB7A
P−value:0.0034; Pearson R:−0.85
Tomlins Prostate : Prostate Carcinoma
MAPK8
ZBTB7A
P−value:0.74; Pearson R:−0.2

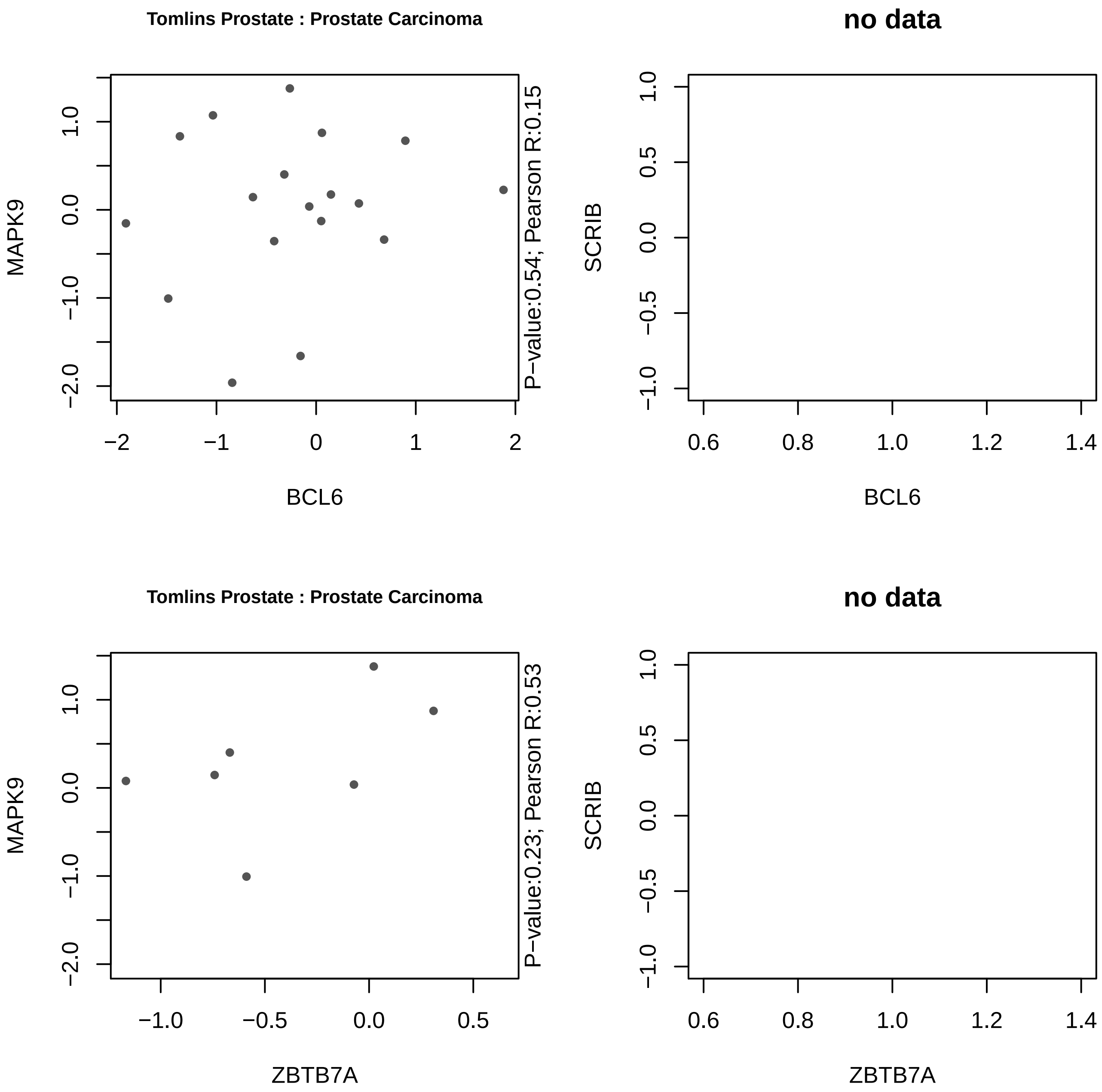
Tomlins Prostate : Prostate Carcinoma
MAPK9
BCL6
P−value:0.54; Pearson R:0.15
no data
SCRIB
BCL6
Tomlins Prostate : Prostate Carcinoma
MAPK9
ZBTB7A
P−value:0.23; Pearson R:0.53
no data
SCRIB
ZBTB7A

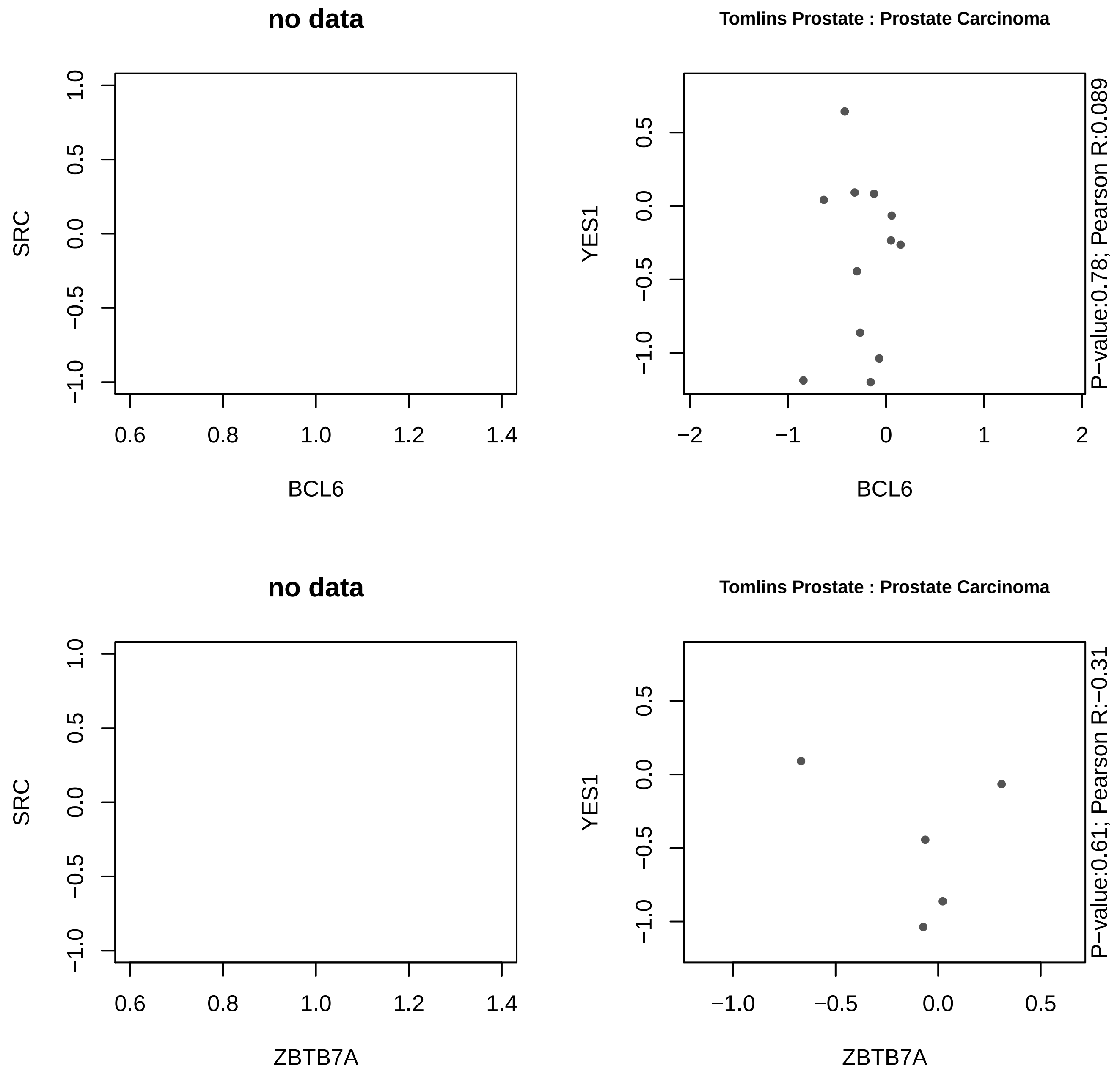
no data
SRC
BCL6
Tomlins Prostate : Prostate Carcinoma
YES1
BCL6
P−value:0.78; Pearson R:0.089
no data
SRC
ZBTB7A
Tomlins Prostate : Prostate Carcinoma
YES1
ZBTB7A
P−value:0.61; Pearson R:−0.31

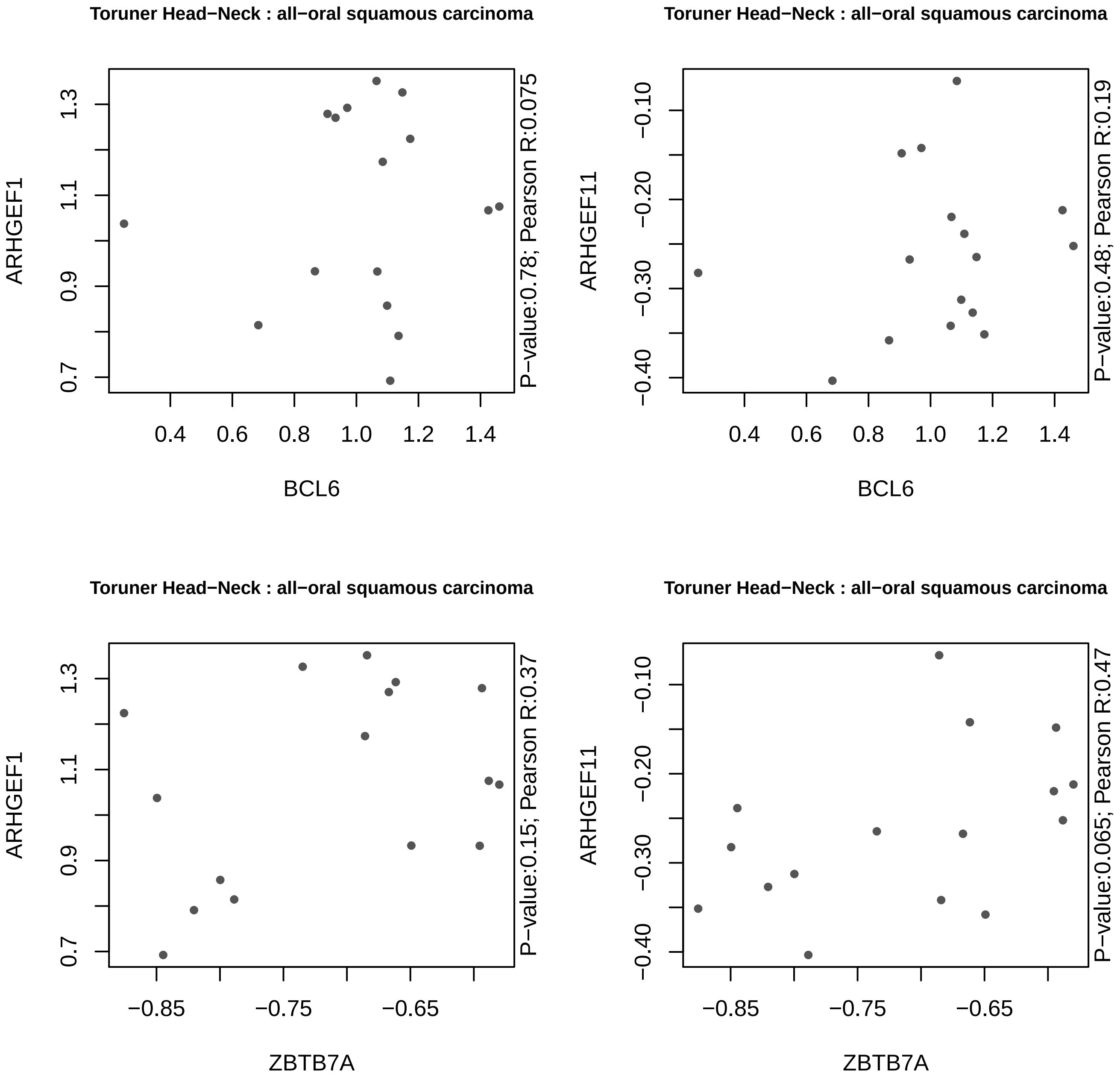
Toruner Head−Neck : all−oral squamous carcinoma
ARHGEF1
BCL6
P−value:0.78; Pearson R:0.075
Toruner Head−Neck : all−oral squamous carcinoma
ARHGEF11
BCL6
P−value:0.48; Pearson R:0.19
Toruner Head−Neck : all−oral squamous carcinoma
ARHGEF1
ZBTB7A
P−value:0.15; Pearson R:0.37
Toruner Head−Neck : all−oral squamous carcinoma
ARHGEF11
ZBTB7A
P−value:0.065; Pearson R:0.47

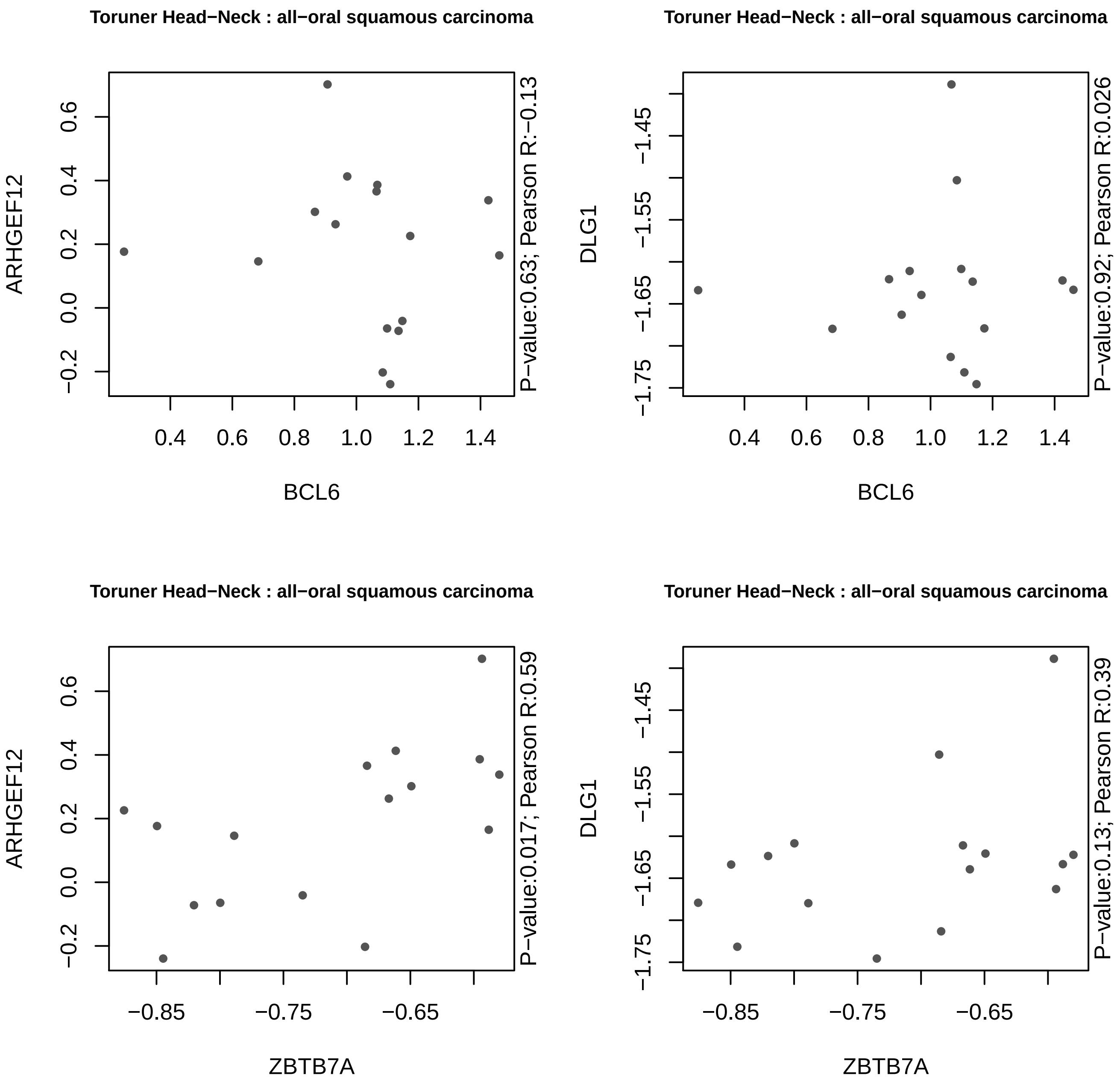
Toruner Head−Neck : all−oral squamous carcinoma
ARHGEF12
BCL6
P−value:0.63; Pearson R:−0.13
Toruner Head−Neck : all−oral squamous carcinoma
DLG1
BCL6
P−value:0.92; Pearson R:0.026
Toruner Head−Neck : all−oral squamous carcinoma
ARHGEF12
ZBTB7A
P−value:0.017; Pearson R:0.59
Toruner Head−Neck : all−oral squamous carcinoma
DLG1
ZBTB7A
P−value:0.13; Pearson R:0.39

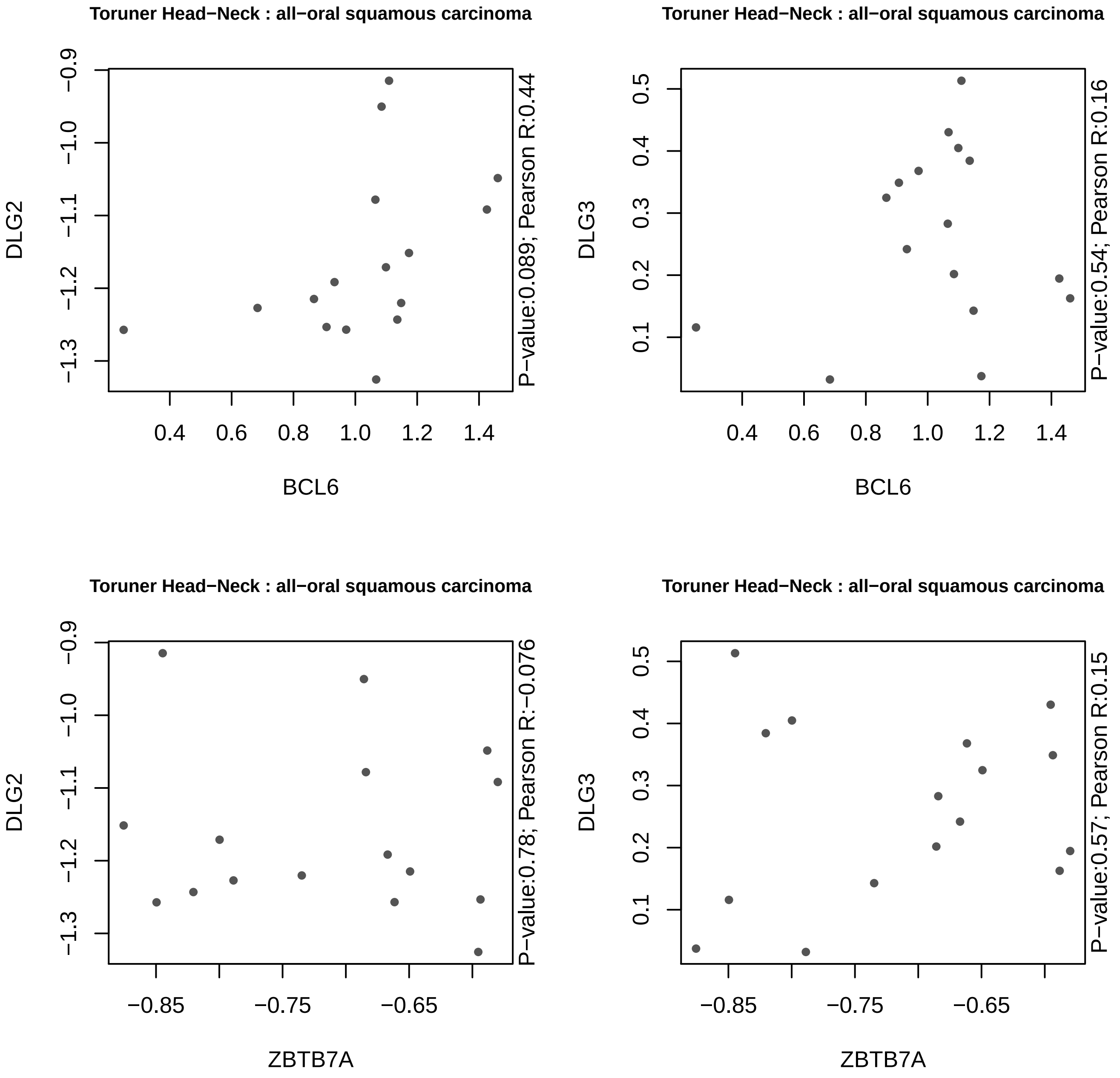
Toruner Head−Neck : all−oral squamous carcinoma
DLG2
BCL6
P−value:0.089; Pearson R:0.44
Toruner Head−Neck : all−oral squamous carcinoma
DLG3
BCL6
P−value:0.54; Pearson R:0.16
Toruner Head−Neck : all−oral squamous carcinoma
DLG2
ZBTB7A
P−value:0.78; Pearson R:−0.076
Toruner Head−Neck : all−oral squamous carcinoma
DLG3
ZBTB7A
P−value:0.57; Pearson R:0.15

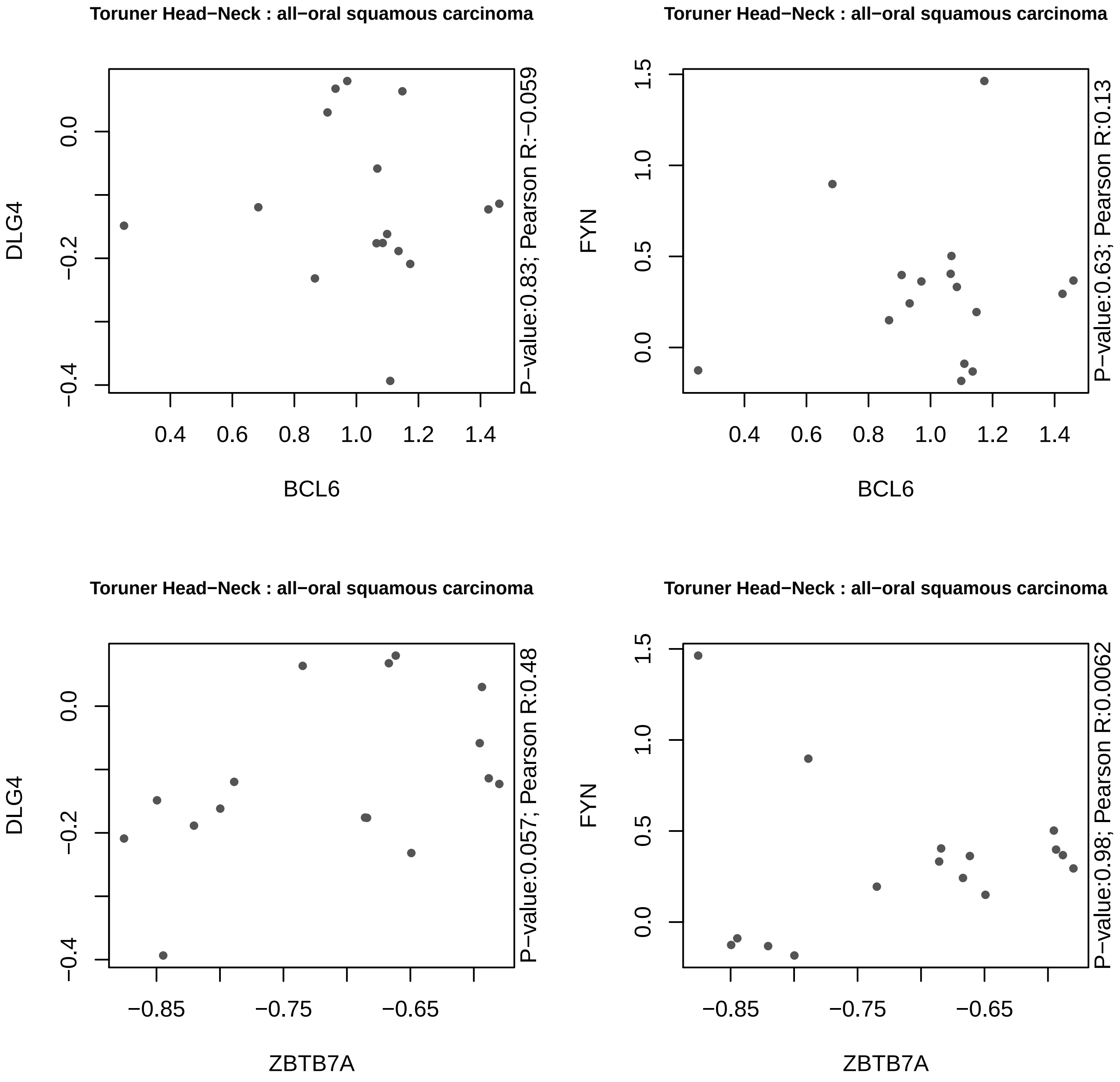
Toruner Head−Neck : all−oral squamous carcinoma
DLG4
BCL6
P−value:0.83; Pearson R:−0.059
Toruner Head−Neck : all−oral squamous carcinoma
FYN
BCL6
P−value:0.63; Pearson R:0.13
Toruner Head−Neck : all−oral squamous carcinoma
DLG4
ZBTB7A
P−value:0.057; Pearson R:0.48
Toruner Head−Neck : all−oral squamous carcinoma
FYN
ZBTB7A
P−value:0.98; Pearson R:0.0062

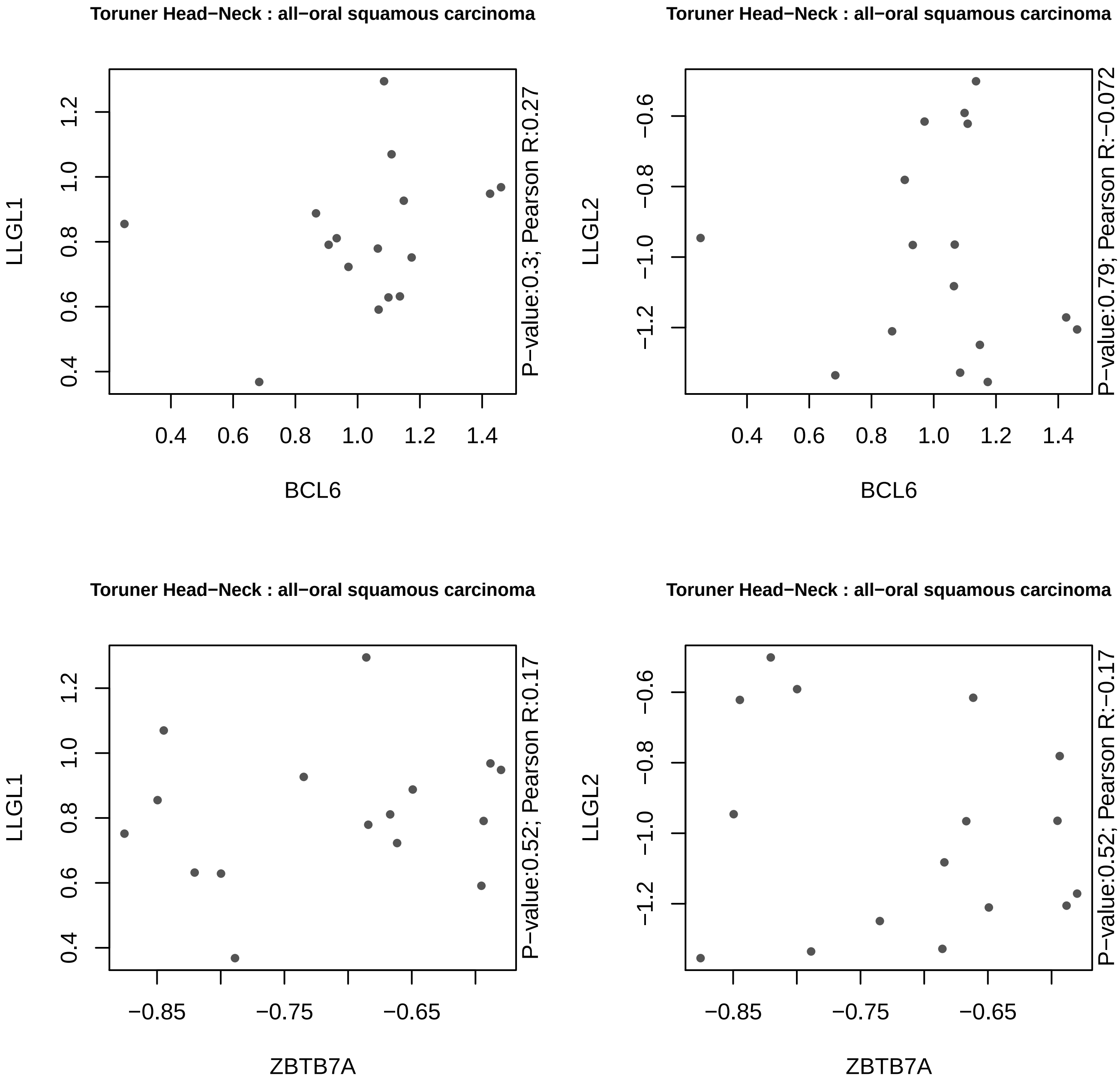
Toruner Head−Neck : all−oral squamous carcinoma
LLGL1
BCL6
P−value:0.3; Pearson R:0.27
Toruner Head−Neck : all−oral squamous carcinoma
LLGL2
BCL6
P−value:0.79; Pearson R:−0.072
Toruner Head−Neck : all−oral squamous carcinoma
LLGL1
ZBTB7A
P−value:0.52; Pearson R:0.17
Toruner Head−Neck : all−oral squamous carcinoma
LLGL2
ZBTB7A
P−value:0.52; Pearson R:−0.17

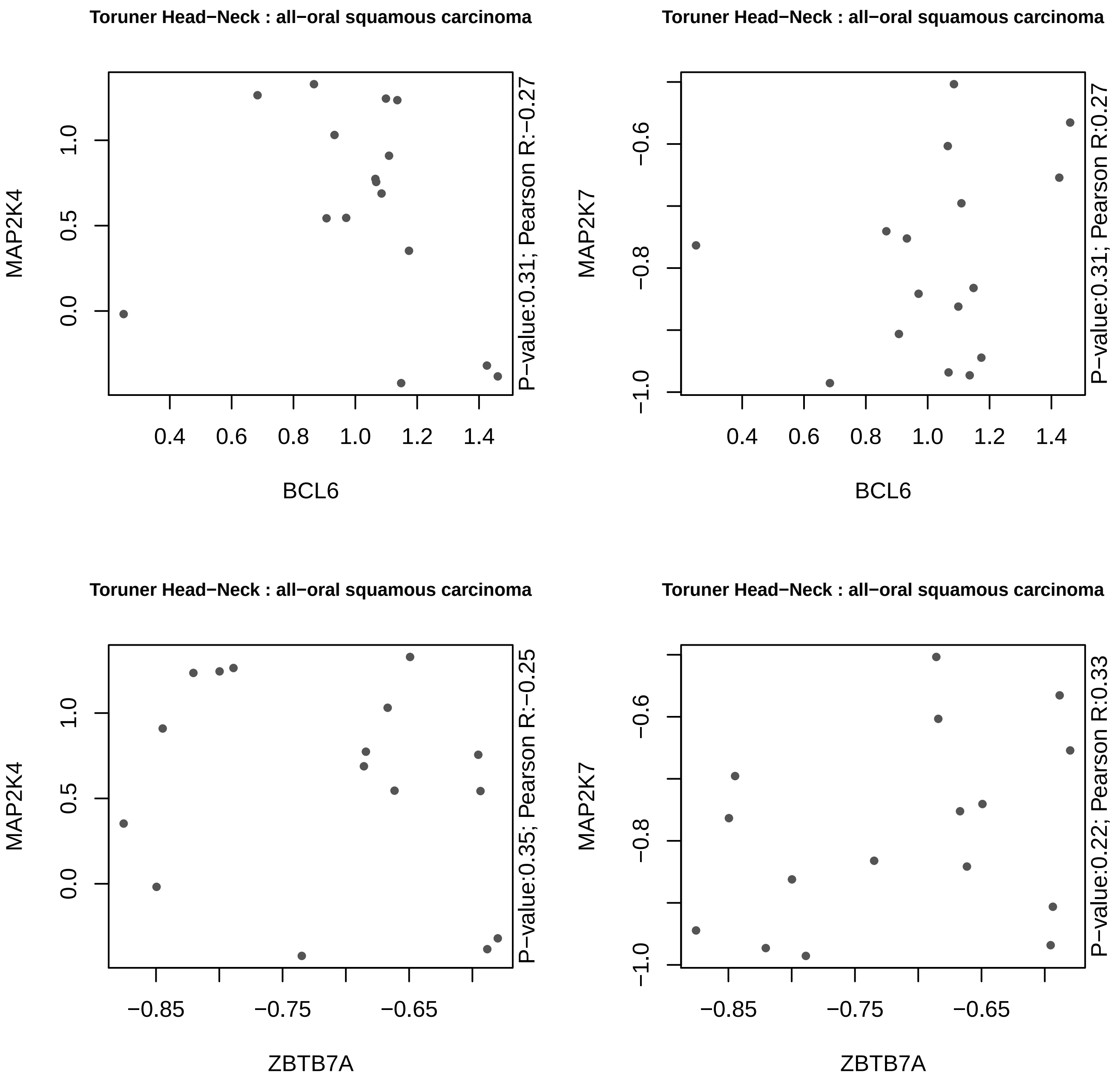
Toruner Head−Neck : all−oral squamous carcinoma
MAP2K4
BCL6
P−value:0.31; Pearson R:−0.27
Toruner Head−Neck : all−oral squamous carcinoma
MAP2K7
BCL6
P−value:0.31; Pearson R:0.27
Toruner Head−Neck : all−oral squamous carcinoma
MAP2K4
ZBTB7A
P−value:0.35; Pearson R:−0.25
Toruner Head−Neck : all−oral squamous carcinoma
MAP2K7
ZBTB7A
P−value:0.22; Pearson R:0.33

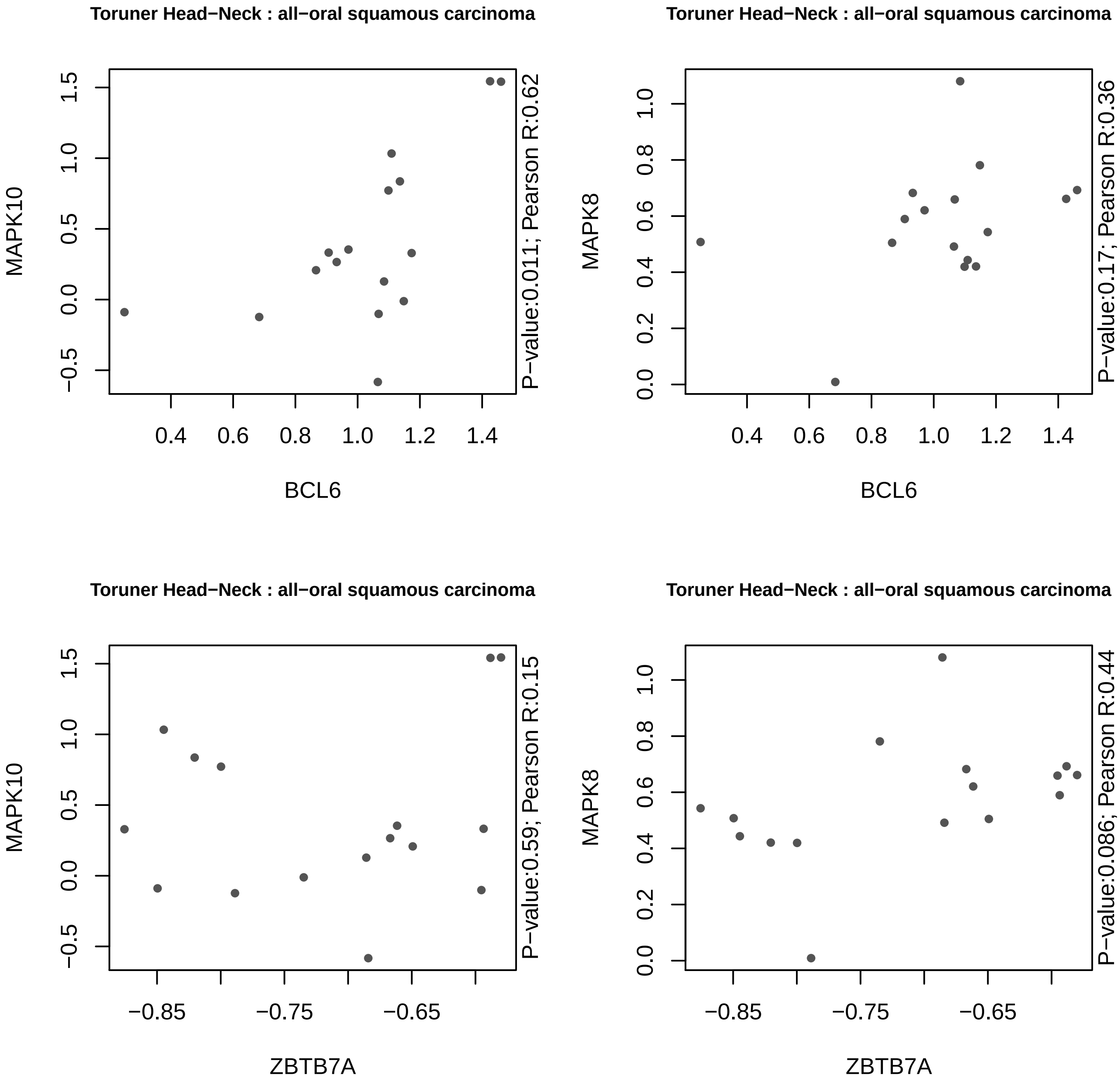
Toruner Head−Neck : all−oral squamous carcinoma
MAPK10
BCL6
P−value:0.011; Pearson R:0.62
Toruner Head−Neck : all−oral squamous carcinoma
MAPK8
BCL6
P−value:0.17; Pearson R:0.36
Toruner Head−Neck : all−oral squamous carcinoma
MAPK10
ZBTB7A
P−value:0.59; Pearson R:0.15
Toruner Head−Neck : all−oral squamous carcinoma
MAPK8
ZBTB7A
P−value:0.086; Pearson R:0.44

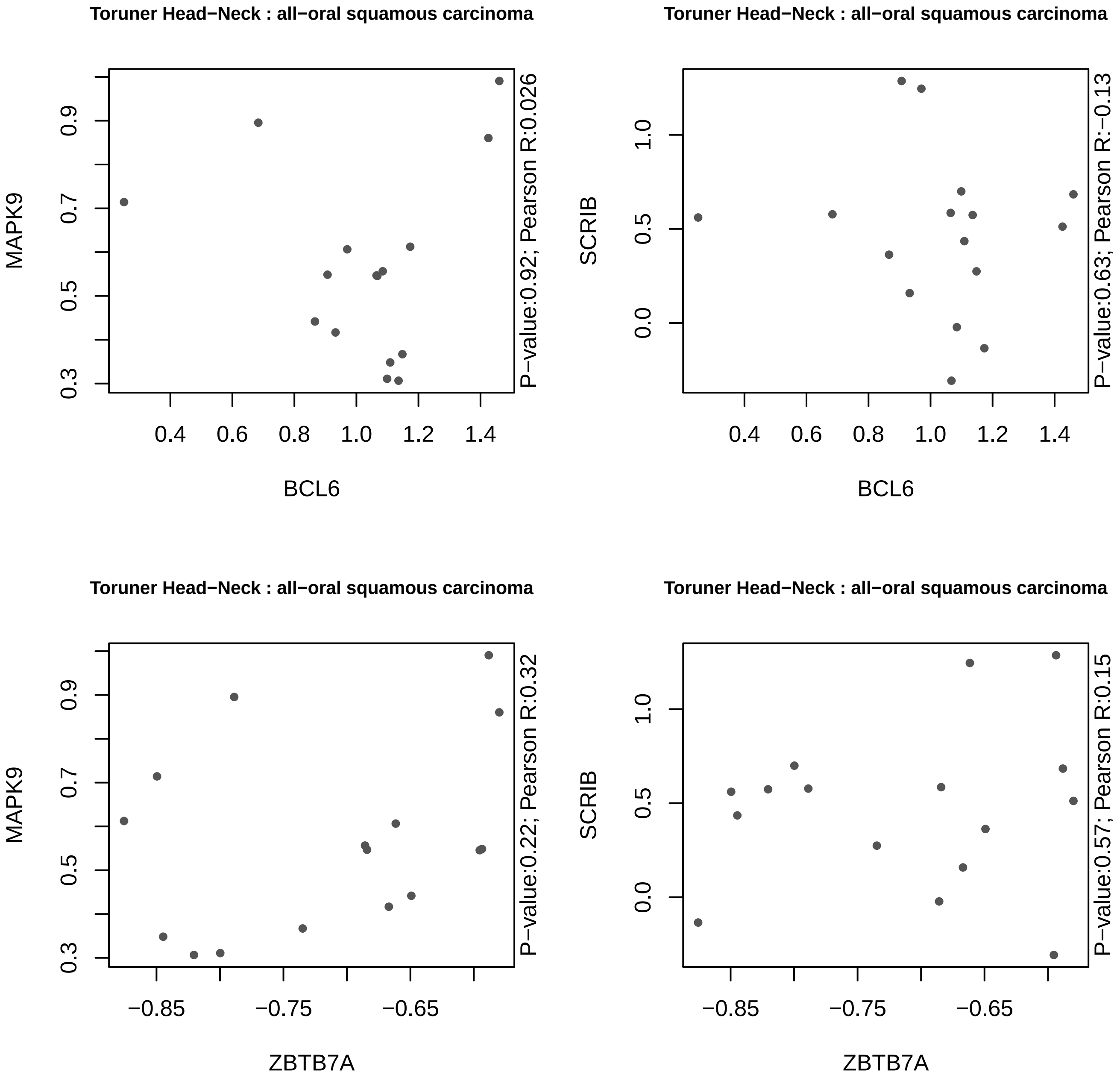
Toruner Head−Neck : all−oral squamous carcinoma
MAPK9
BCL6
P−value:0.92; Pearson R:0.026
Toruner Head−Neck : all−oral squamous carcinoma
SCRIB
BCL6
P−value:0.63; Pearson R:−0.13
Toruner Head−Neck : all−oral squamous carcinoma
MAPK9
ZBTB7A
P−value:0.22; Pearson R:0.32
Toruner Head−Neck : all−oral squamous carcinoma
SCRIB
ZBTB7A
P−value:0.57; Pearson R:0.15

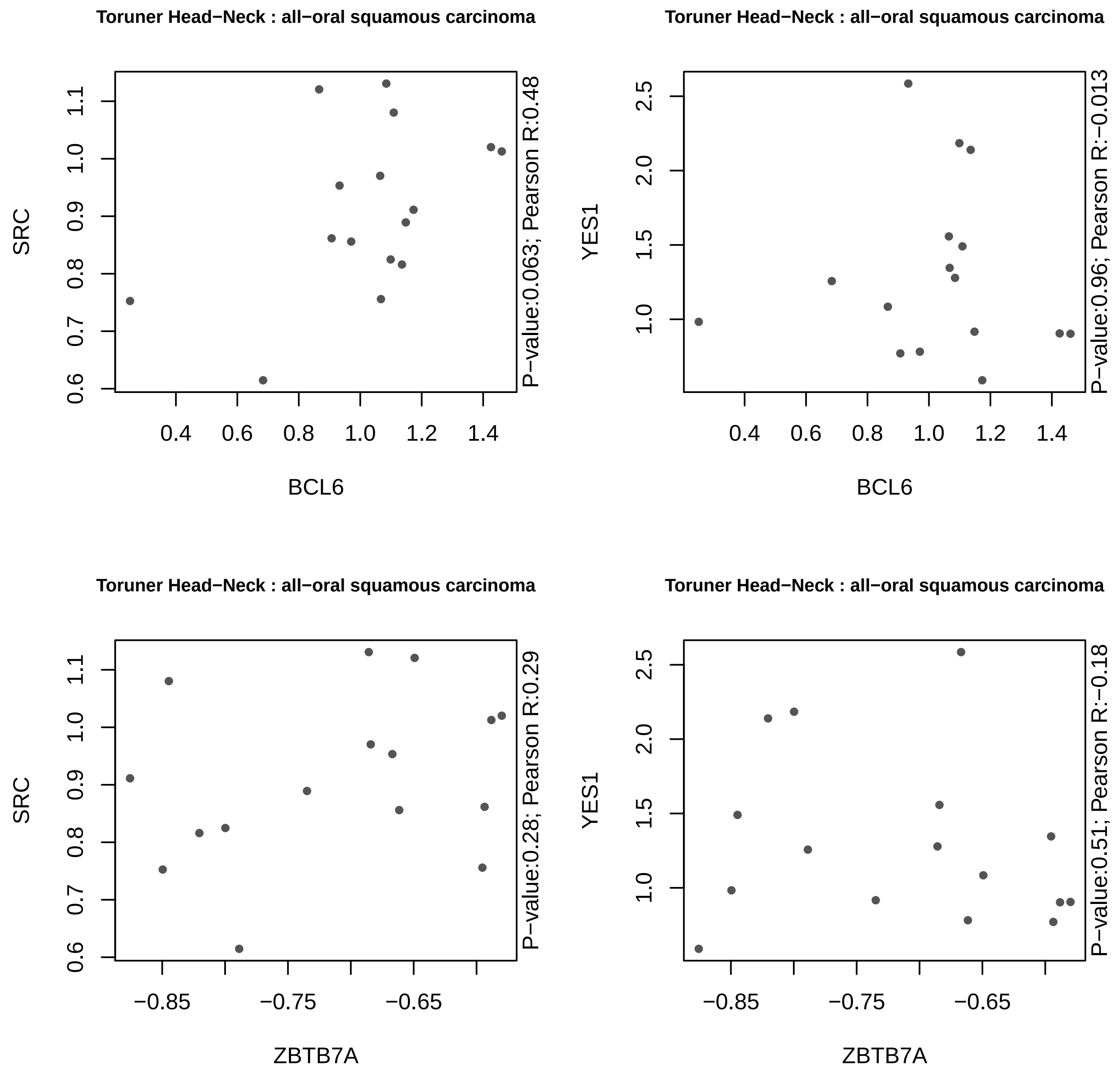
Toruner Head−Neck : all−oral squamous carcinoma
SRC
BCL6
P−value:0.063; Pearson R:0.48
Toruner Head−Neck : all−oral squamous carcinoma
YES1
BCL6
P−value:0.96; Pearson R:−0.013
Toruner Head−Neck : all−oral squamous carcinoma
SRC
ZBTB7A
P−value:0.28; Pearson R:0.29
Toruner Head−Neck : all−oral squamous carcinoma
YES1
ZBTB7A
P−value:0.51; Pearson R:−0.18

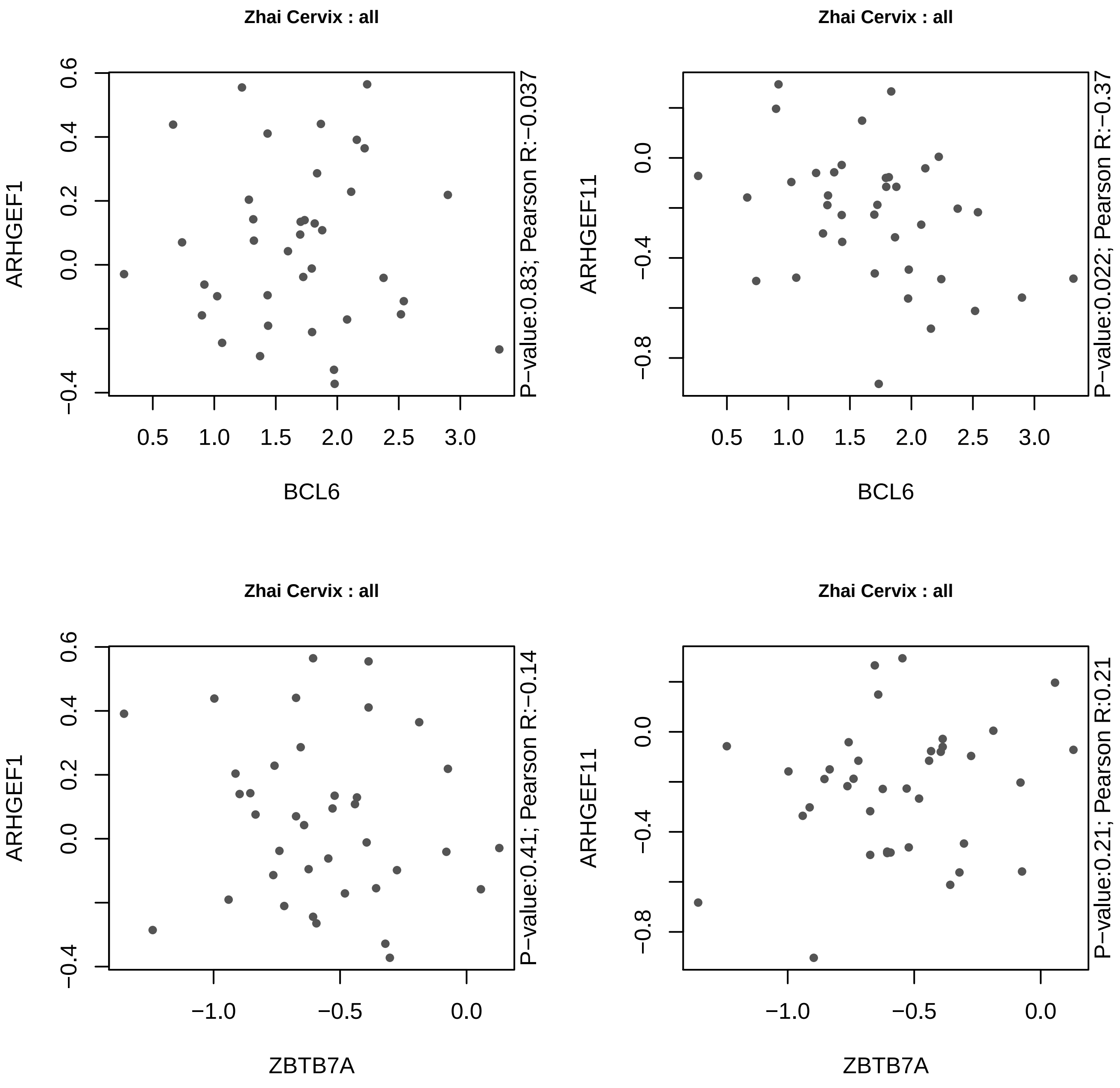
Zhai Cervix : all
ARHGEF1
BCL6
P−value:0.83; Pearson R:−0.037
Zhai Cervix : all
ARHGEF11
BCL6
P−value:0.022; Pearson R:−0.37
Zhai Cervix : all
ARHGEF1
ZBTB7A
P−value:0.41; Pearson R:−0.14
Zhai Cervix : all
ARHGEF11
ZBTB7A
P−value:0.21; Pearson R:0.21

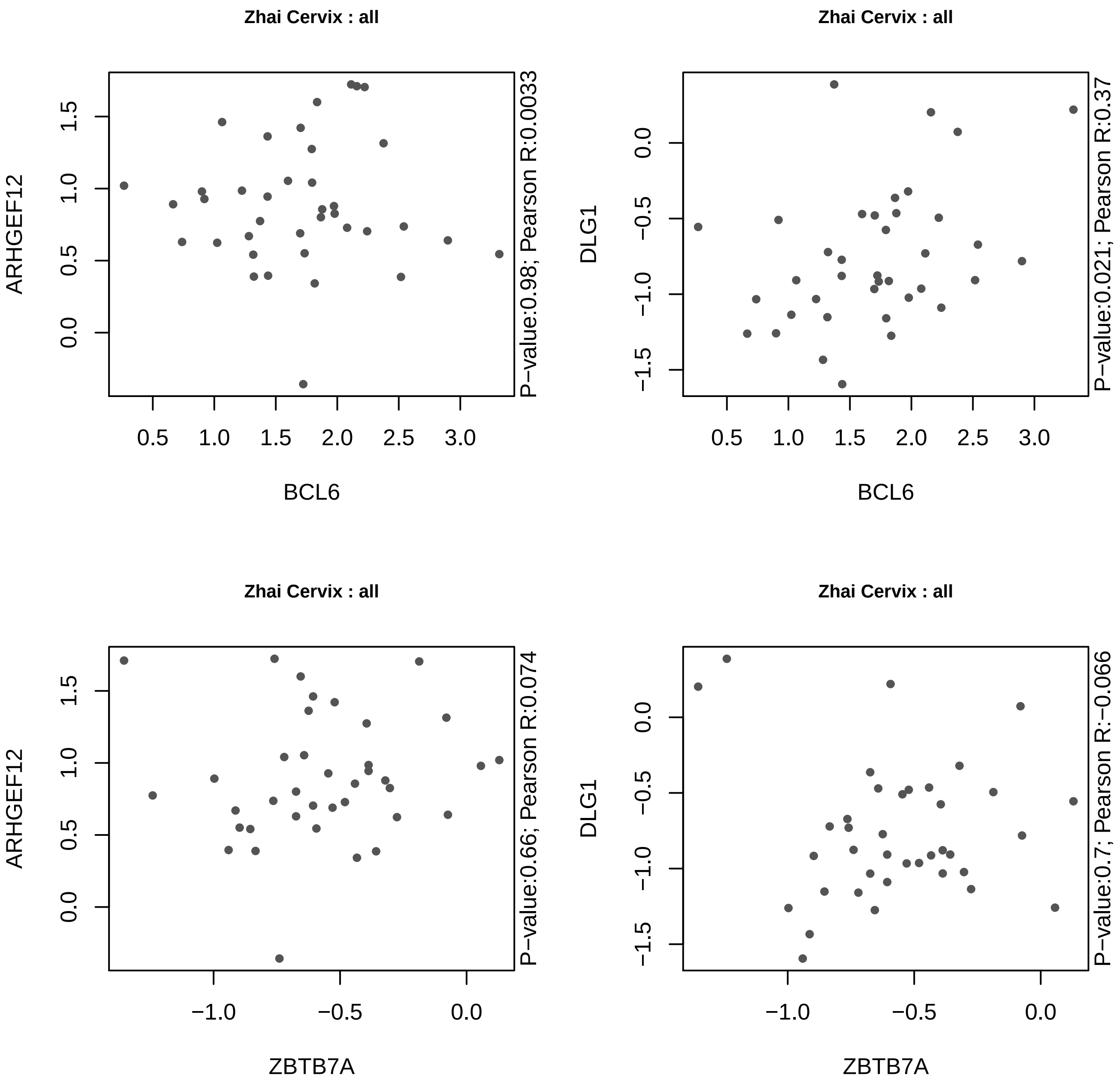
Zhai Cervix : all
ARHGEF12
BCL6
P−value:0.98; Pearson R:0.0033
Zhai Cervix : all
DLG1
BCL6
P−value:0.021; Pearson R:0.37
Zhai Cervix : all
ARHGEF12
ZBTB7A
P−value:0.66; Pearson R:0.074
Zhai Cervix : all
DLG1
ZBTB7A
P−value:0.7; Pearson R:−0.066

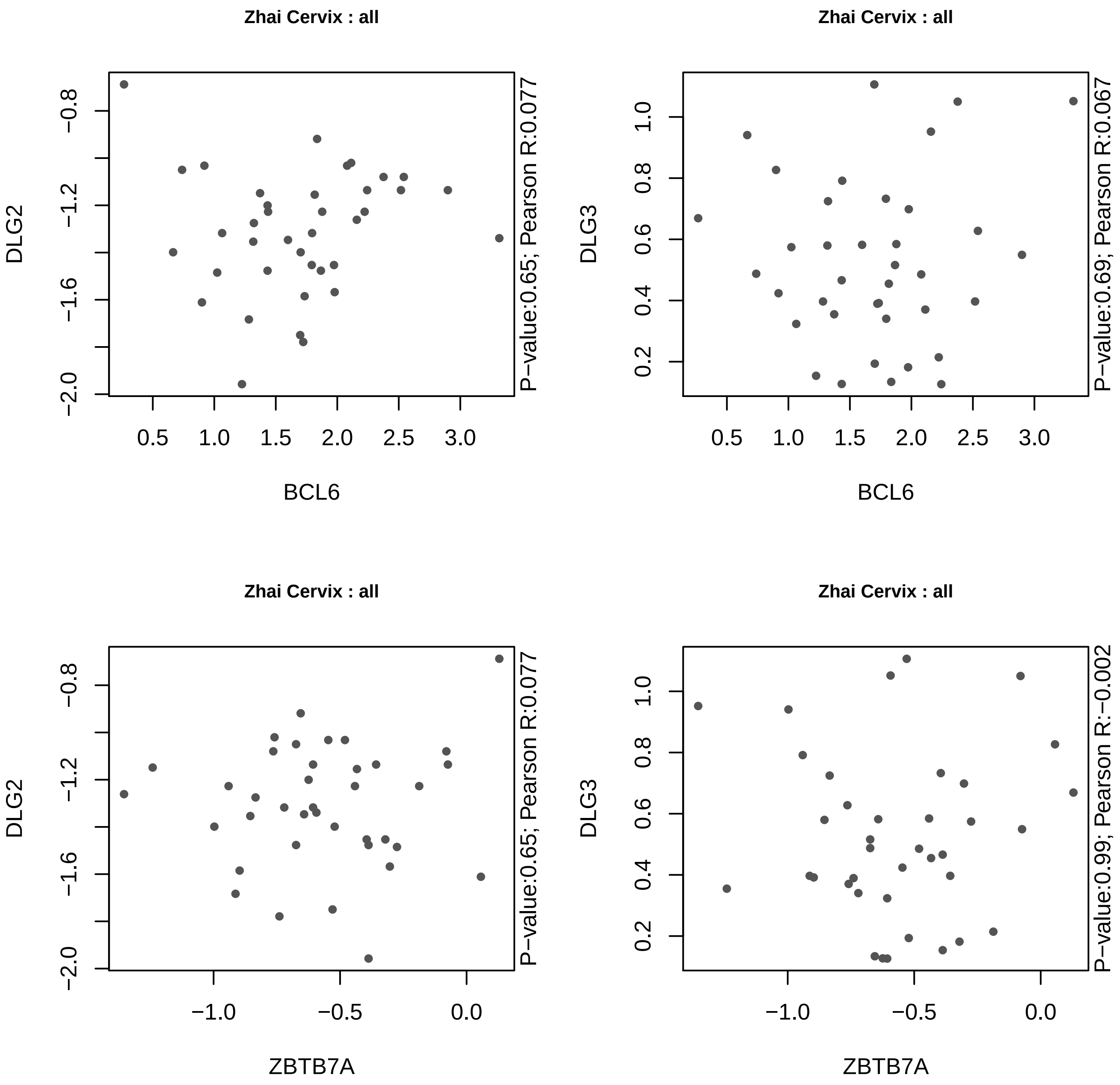
Zhai Cervix : all
DLG2
BCL6
P−value:0.65; Pearson R:0.077
Zhai Cervix : all
DLG3
BCL6
P−value:0.69; Pearson R:0.067
Zhai Cervix : all
DLG2
ZBTB7A
P−value:0.65; Pearson R:0.077
Zhai Cervix : all
DLG3
ZBTB7A
P−value:0.99; Pearson R:−0.002

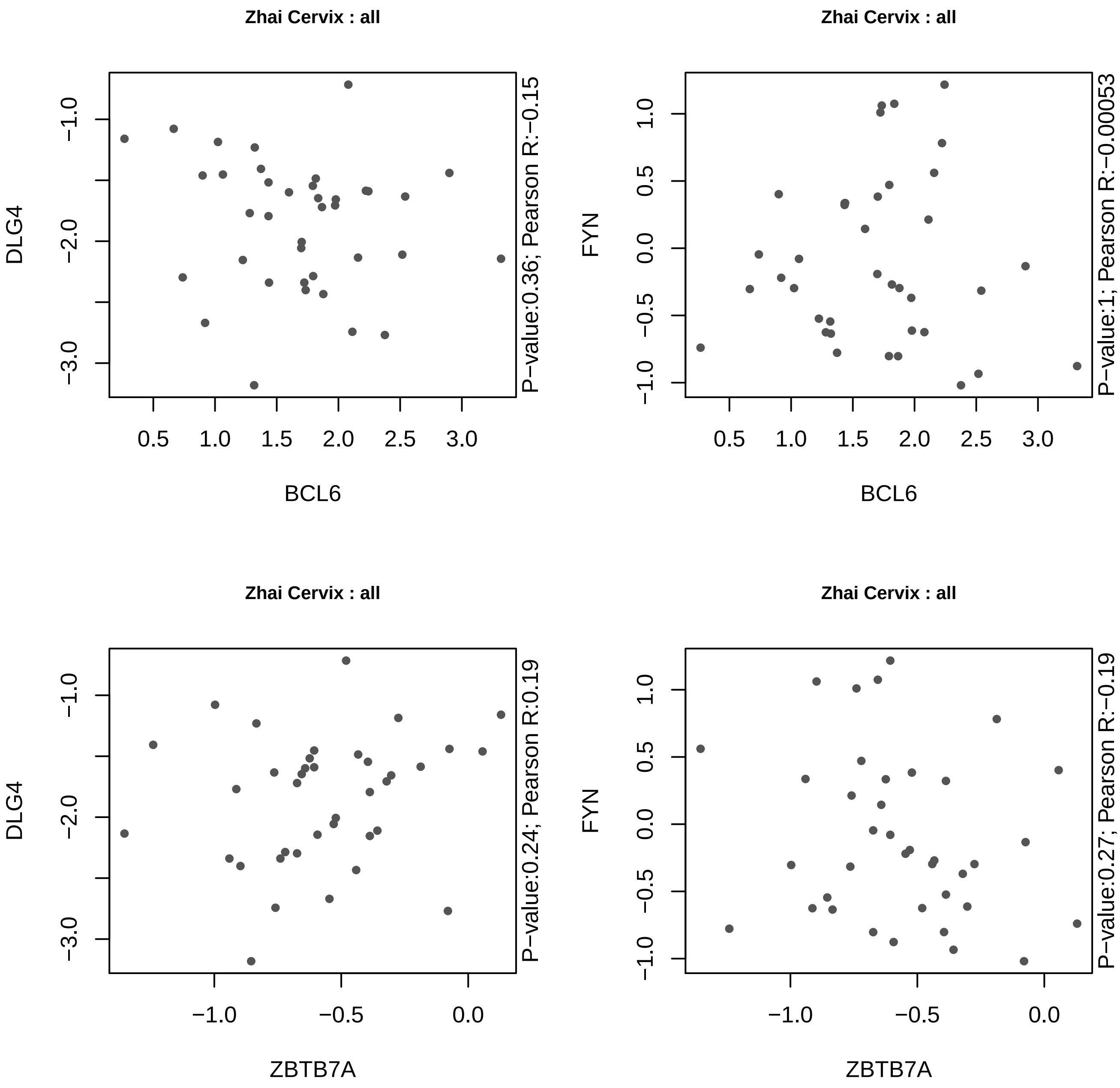
Zhai Cervix : all
DLG4
BCL6
P−value:0.36; Pearson R:−0.15
Zhai Cervix : all
FYN
BCL6
P−value:1; Pearson R:−0.00053
Zhai Cervix : all
DLG4
ZBTB7A
P−value:0.24; Pearson R:0.19
Zhai Cervix : all
FYN
ZBTB7A
P−value:0.27; Pearson R:−0.19

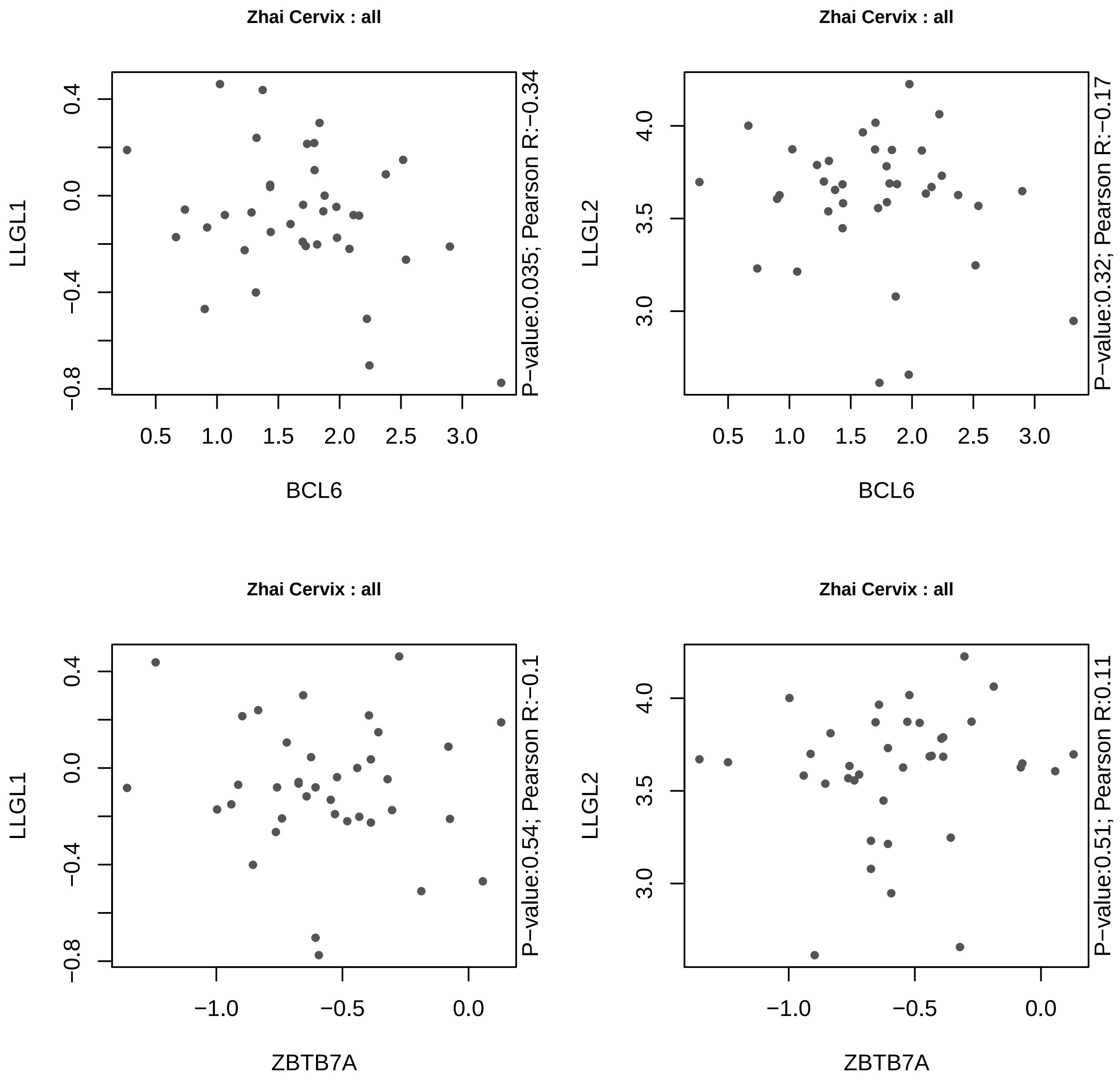
Zhai Cervix : all
LLGL1
BCL6
P−value:0.035; Pearson R:−0.34
Zhai Cervix : all
LLGL2
BCL6
P−value:0.32; Pearson R:−0.17
Zhai Cervix : all
LLGL1
ZBTB7A
P−value:0.54; Pearson R:−0.1
Zhai Cervix : all
LLGL2
ZBTB7A
P−value:0.51; Pearson R:0.11

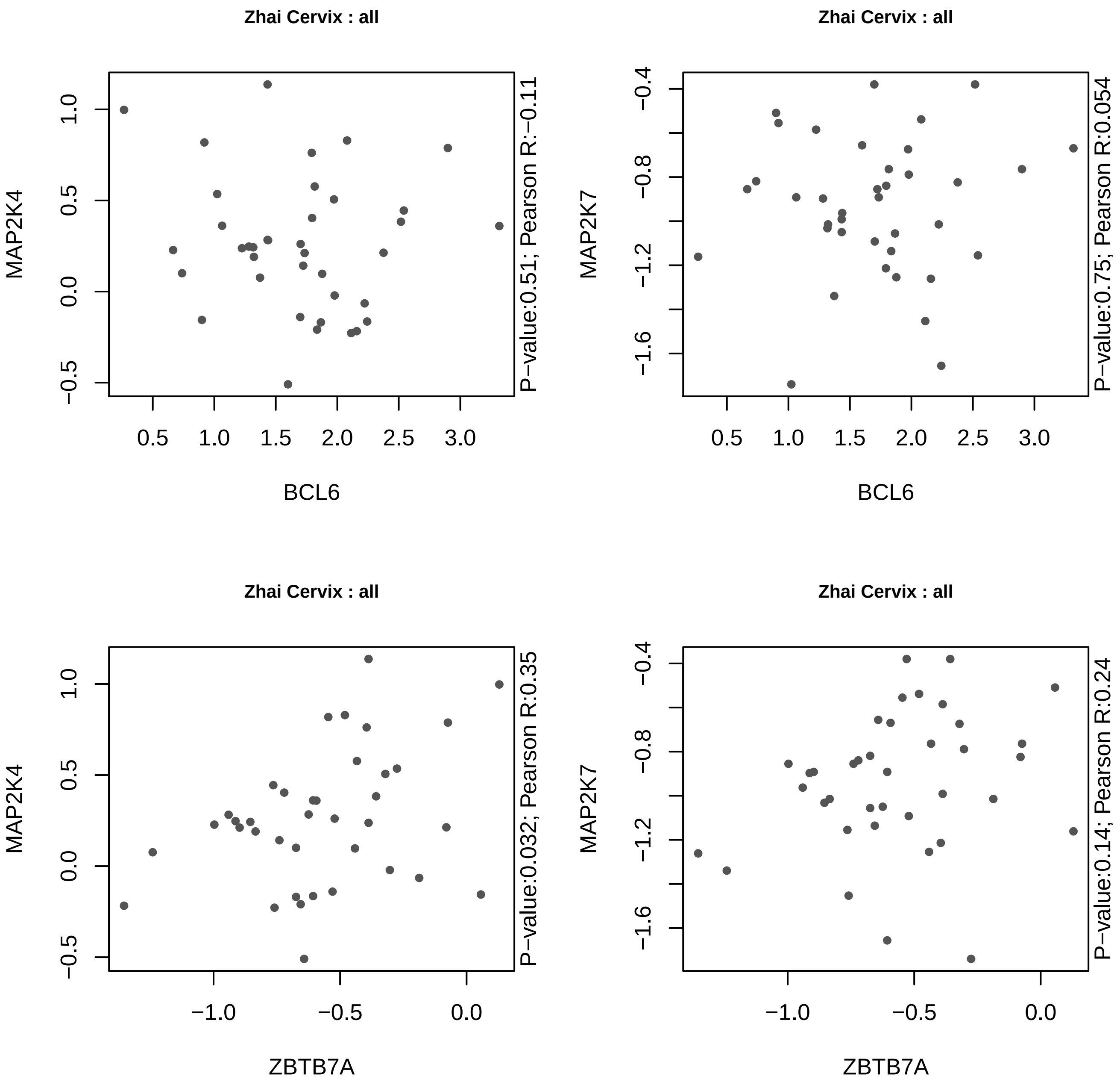
Zhai Cervix : all
MAP2K4
BCL6
P−value:0.51; Pearson R:−0.11
Zhai Cervix : all
MAP2K7
BCL6
P−value:0.75; Pearson R:0.054
Zhai Cervix : all
MAP2K4
ZBTB7A
P−value:0.032; Pearson R:0.35
Zhai Cervix : all
MAP2K7
ZBTB7A
P−value:0.14; Pearson R:0.24

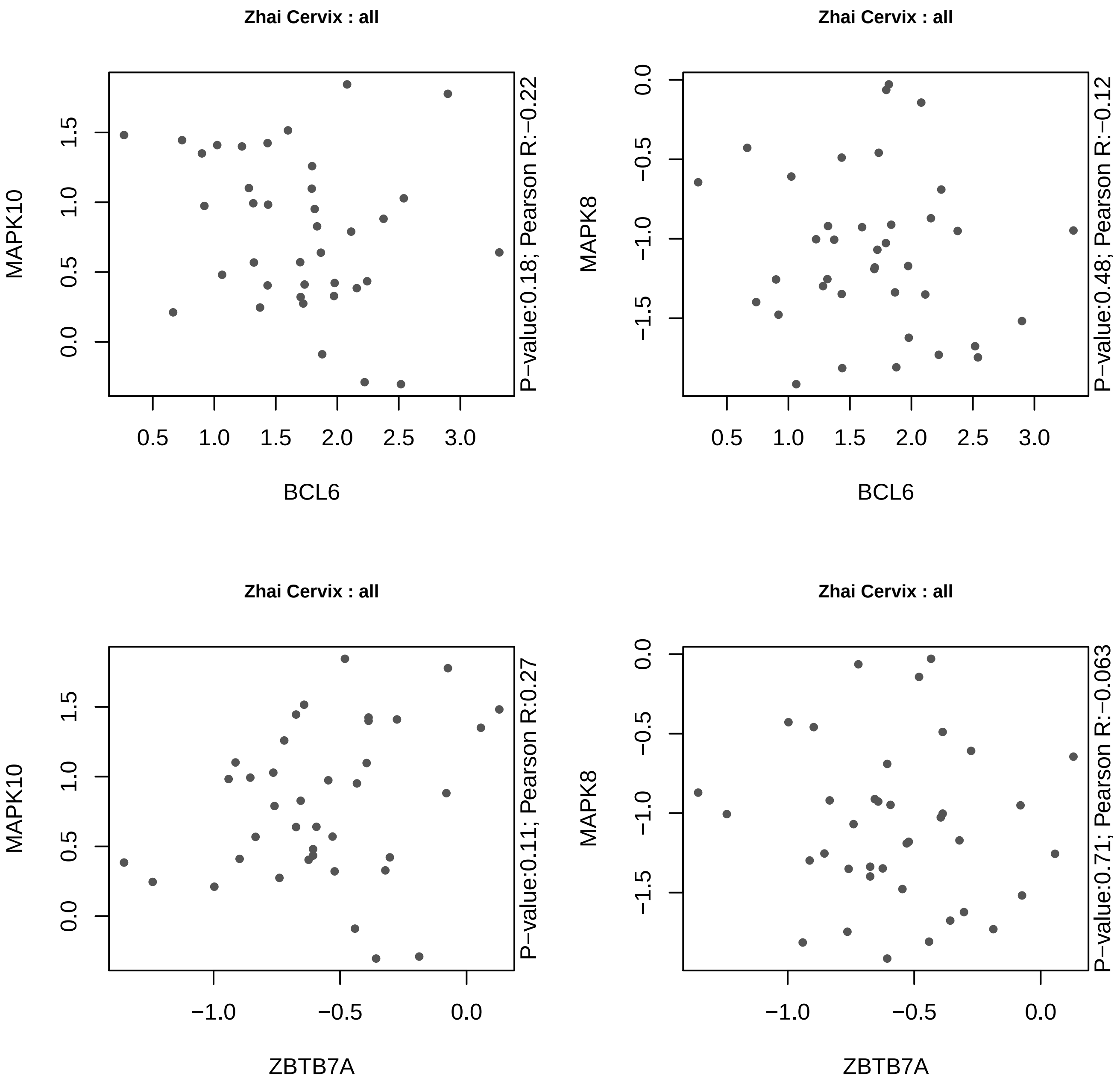
Zhai Cervix : all
MAPK10
BCL6
P−value:0.18; Pearson R:−0.22
Zhai Cervix : all
MAPK8
BCL6
P−value:0.48; Pearson R:−0.12
Zhai Cervix : all
MAPK10
ZBTB7A
P−value:0.11; Pearson R:0.27
Zhai Cervix : all
MAPK8
ZBTB7A
P−value:0.71; Pearson R:−0.063

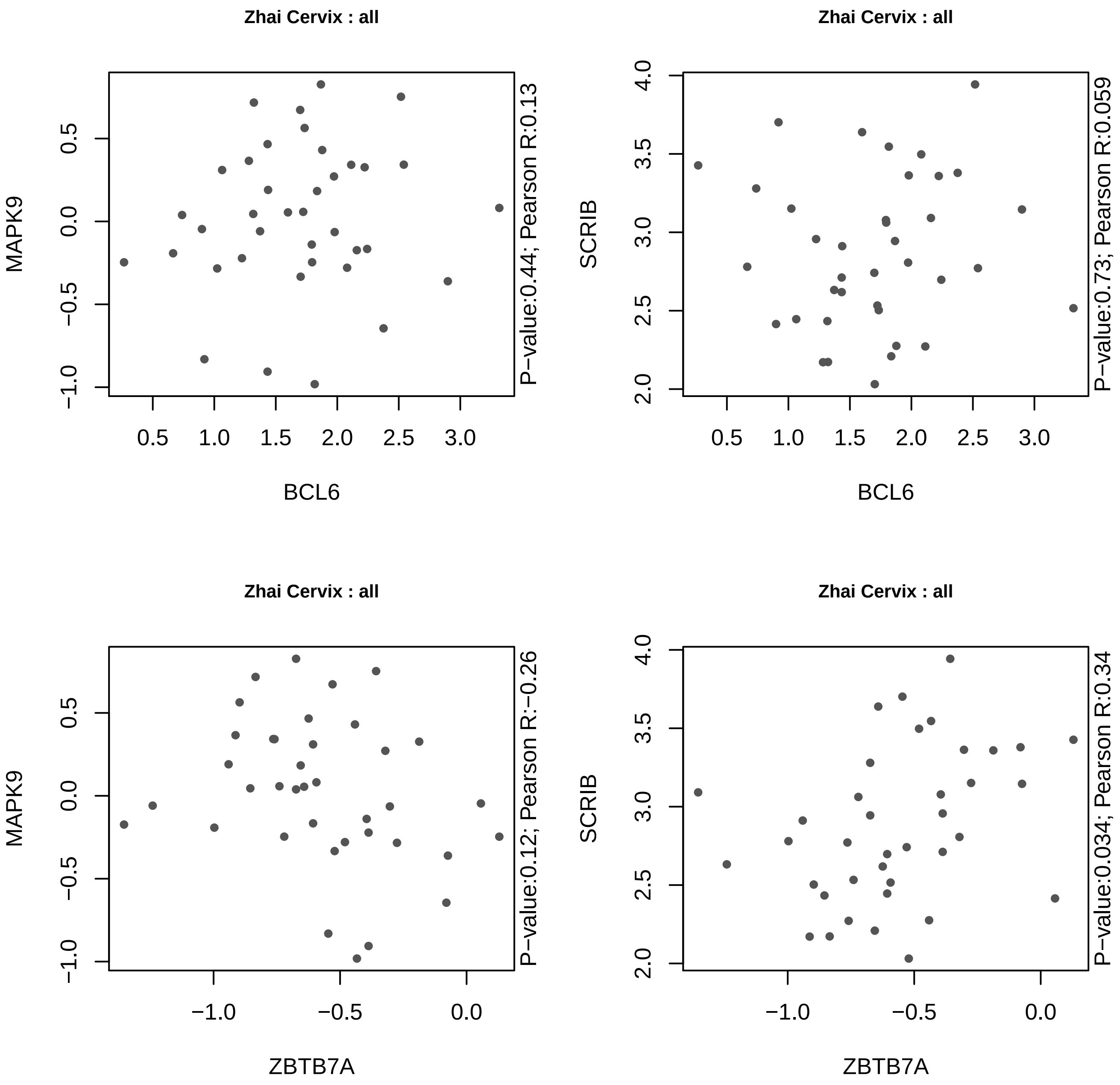
Zhai Cervix : all
MAPK9
BCL6
P−value:0.44; Pearson R:0.13
Zhai Cervix : all
SCRIB
BCL6
P−value:0.73; Pearson R:0.059
Zhai Cervix : all
MAPK9
ZBTB7A
P−value:0.12; Pearson R:−0.26
Zhai Cervix : all
SCRIB
ZBTB7A
P−value:0.034; Pearson R:0.34

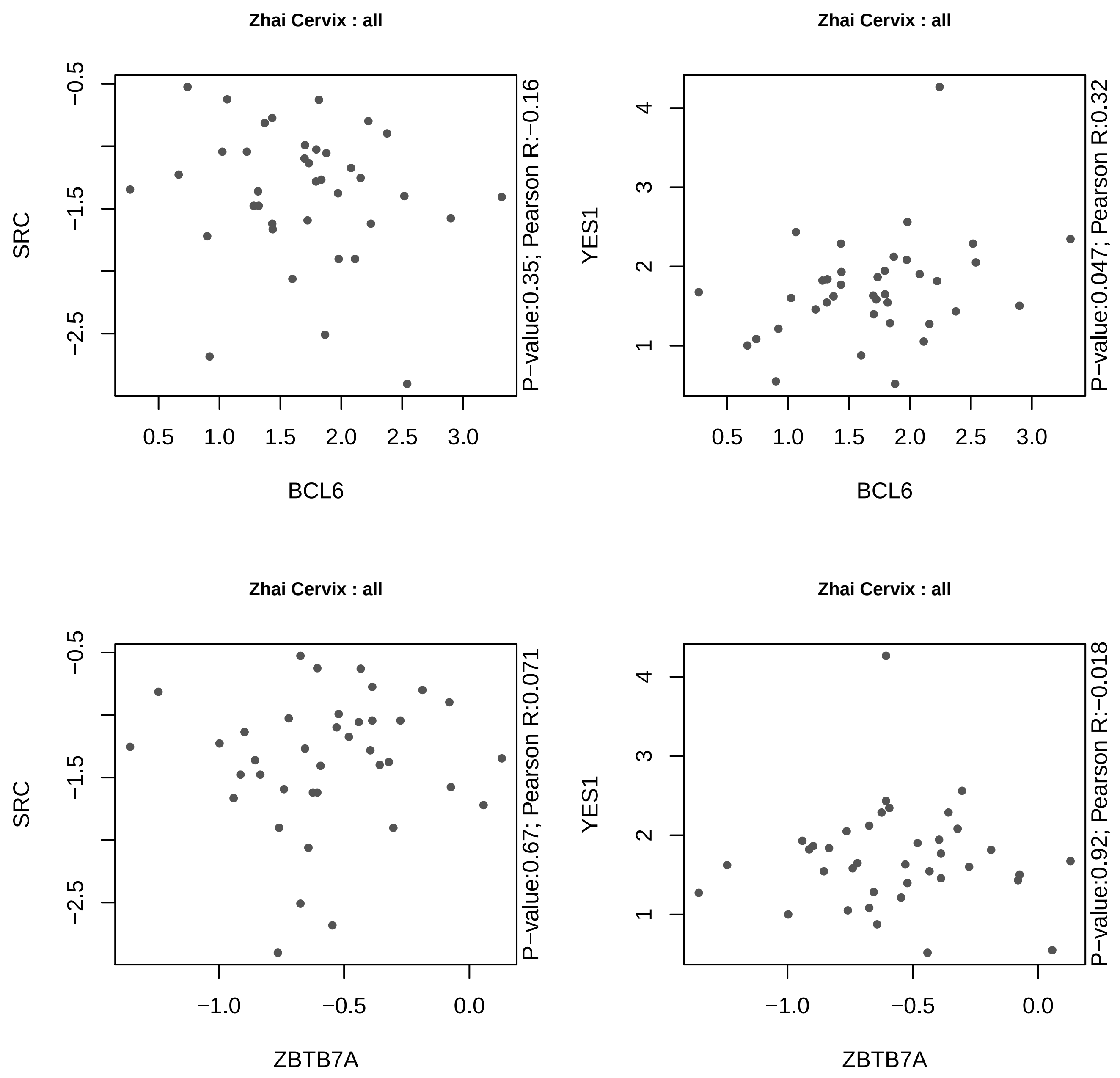

Zhai Cervix : all
SRC
BCL6
P−value:0.35; Pearson R:−0.16
Zhai Cervix : all
YES1
BCL6
P−value:0.047; Pearson R:0.32
Zhai Cervix : all
SRC
ZBTB7A
P−value:0.67; Pearson R:0.071
Zhai Cervix : all
YES1
ZBTB7A
P−value:0.92; Pearson R:−0.018

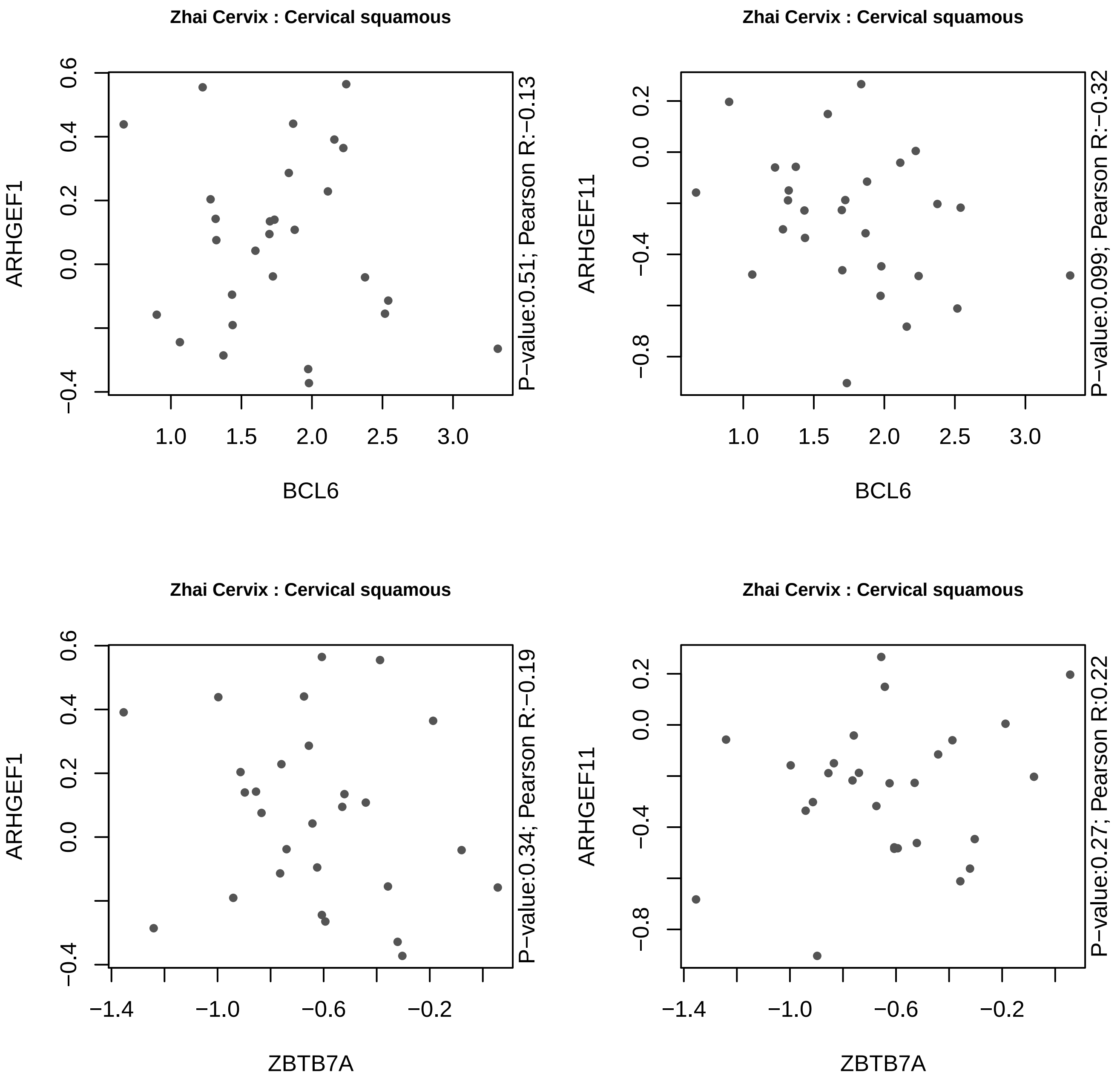
Zhai Cervix : Cervical squamous
ARHGEF1
BCL6
P−value:0.51; Pearson R:−0.13
Zhai Cervix : Cervical squamous
ARHGEF11
BCL6
P−value:0.099; Pearson R:−0.32
Zhai Cervix : Cervical squamous
ARHGEF1
ZBTB7A
P−value:0.34; Pearson R:−0.19
Zhai Cervix : Cervical squamous
ARHGEF11
ZBTB7A
P−value:0.27; Pearson R:0.22

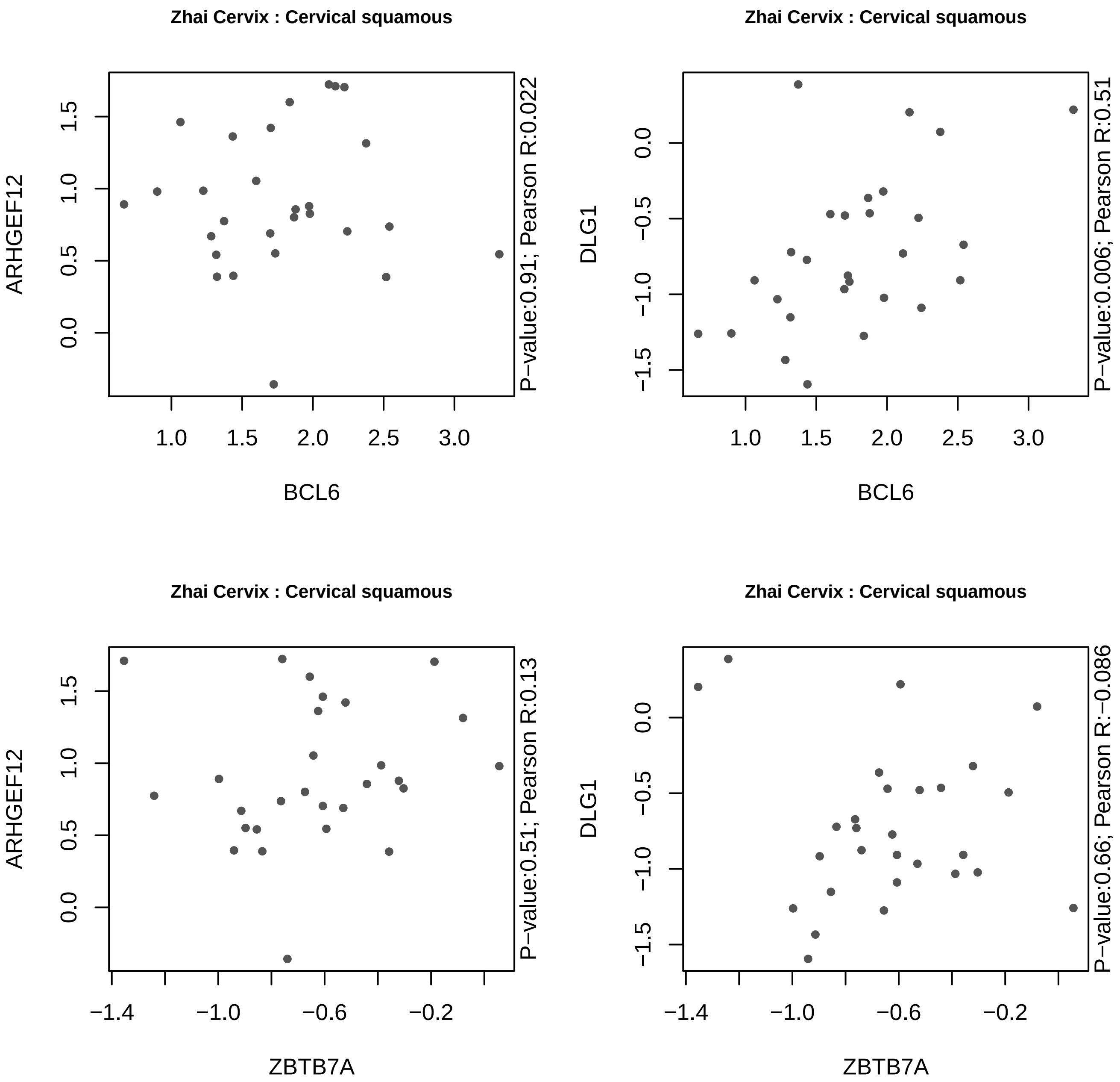
Zhai Cervix : Cervical squamous
ARHGEF12
BCL6
P−value:0.91; Pearson R:0.022
Zhai Cervix : Cervical squamous
DLG1
BCL6
P−value:0.006; Pearson R:0.51
Zhai Cervix : Cervical squamous
ARHGEF12
ZBTB7A
P−value:0.51; Pearson R:0.13
Zhai Cervix : Cervical squamous
DLG1
ZBTB7A
P−value:0.66; Pearson R:−0.086

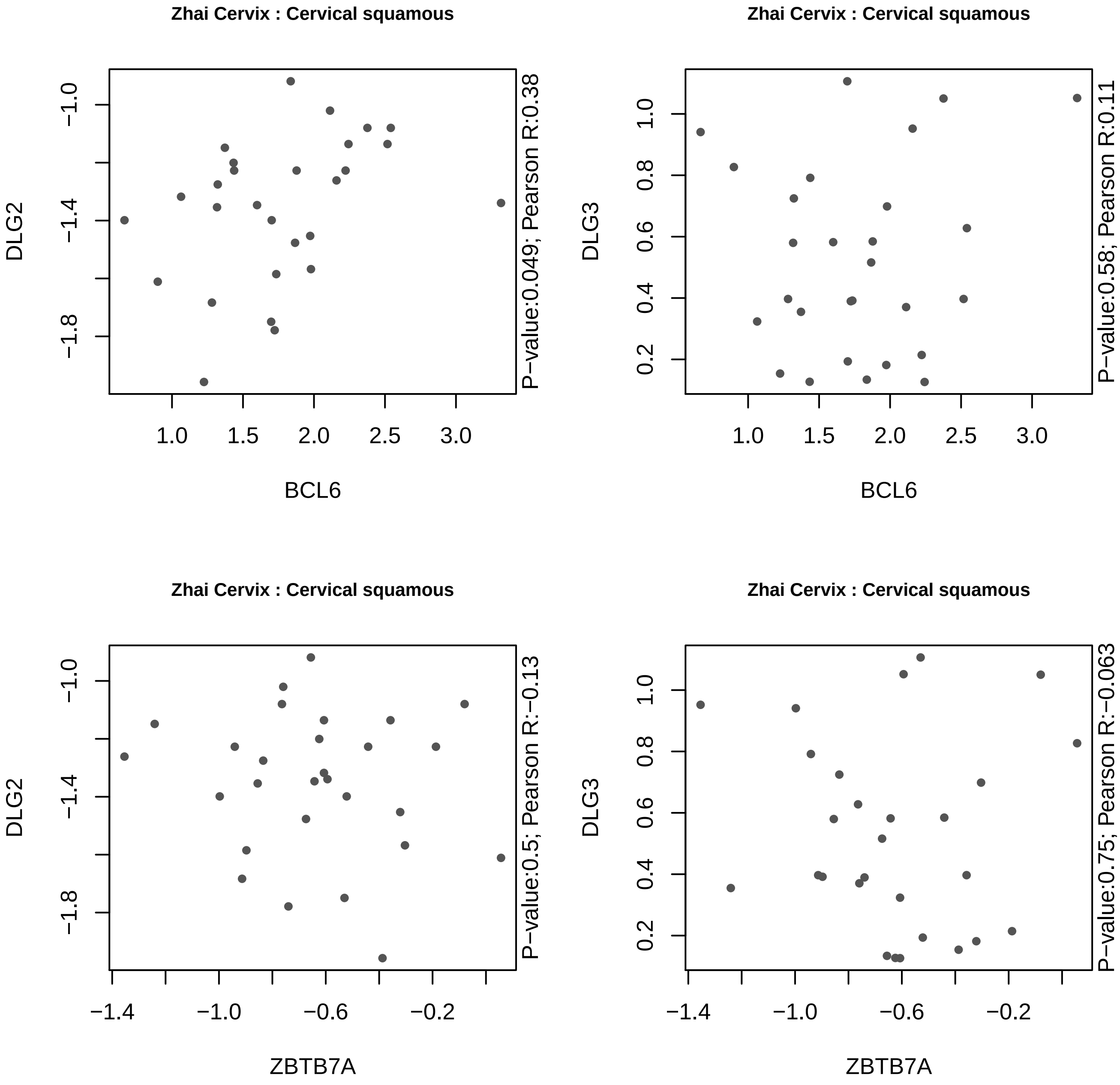
Zhai Cervix : Cervical squamous
DLG2
BCL6
P−value:0.049; Pearson R:0.38
Zhai Cervix : Cervical squamous
DLG3
BCL6
P−value:0.58; Pearson R:0.11
Zhai Cervix : Cervical squamous
DLG2
ZBTB7A
P−value:0.5; Pearson R:−0.13
Zhai Cervix : Cervical squamous
DLG3
ZBTB7A
P−value:0.75; Pearson R:−0.063

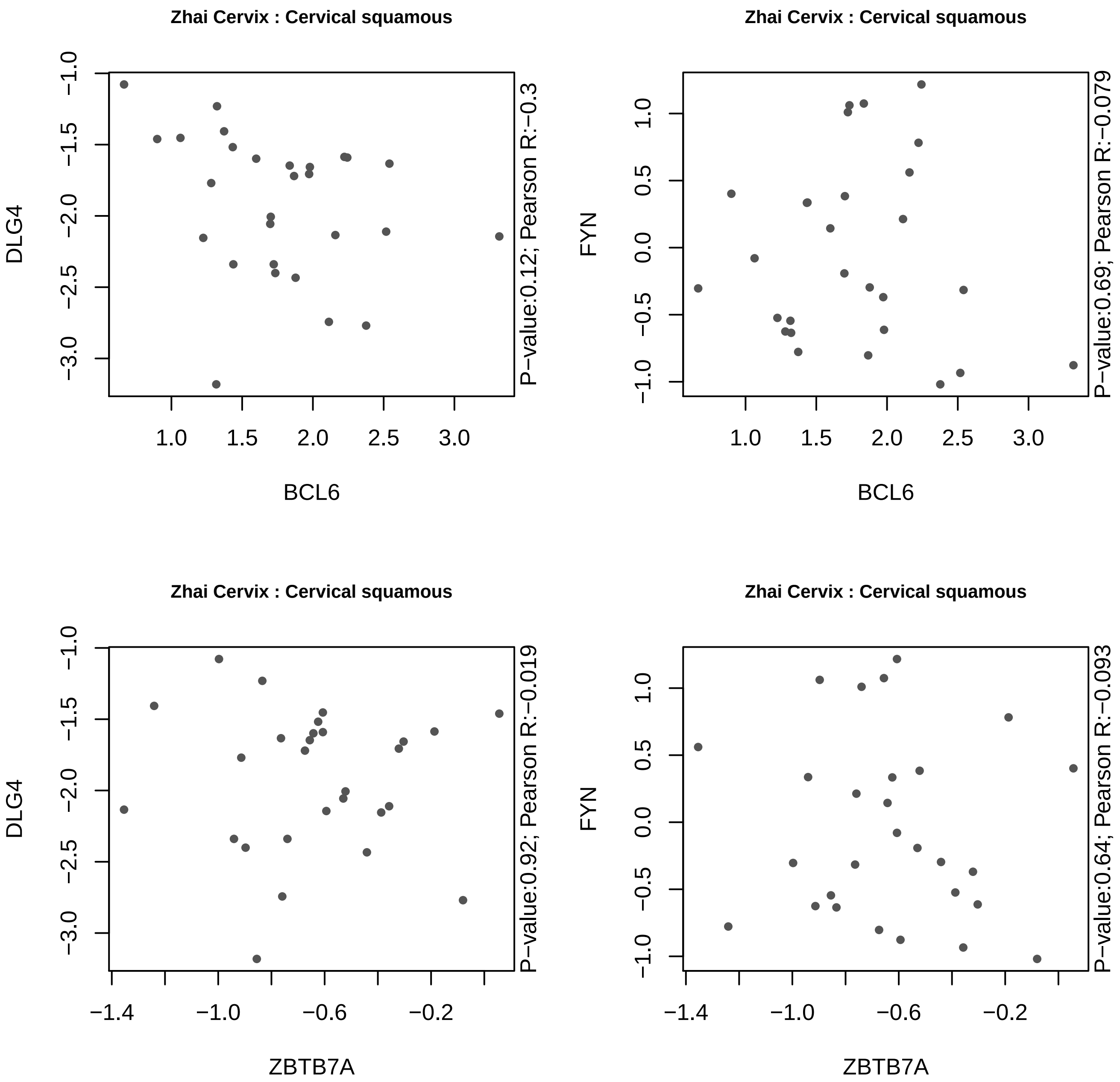
Zhai Cervix : Cervical squamous
DLG4
BCL6
P−value:0.12; Pearson R:−0.3
Zhai Cervix : Cervical squamous
FYN
BCL6
P−value:0.69; Pearson R:−0.079
Zhai Cervix : Cervical squamous
DLG4
ZBTB7A
P−value:0.92; Pearson R:−0.019
Zhai Cervix : Cervical squamous
FYN
ZBTB7A
P−value:0.64; Pearson R:−0.093

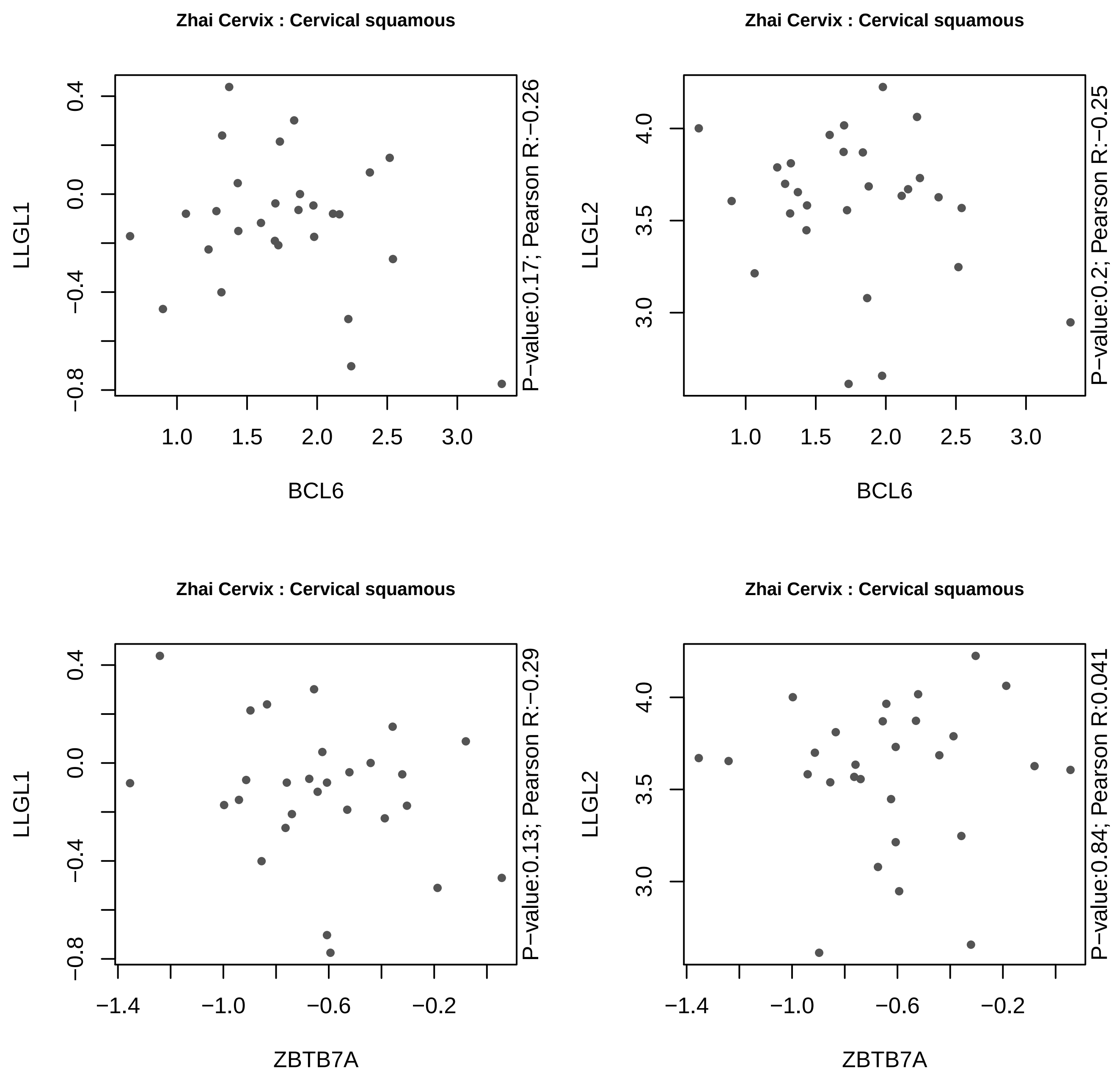
Zhai Cervix : Cervical squamous
LLGL1
BCL6
P−value:0.17; Pearson R:−0.26
Zhai Cervix : Cervical squamous
LLGL2
BCL6
P−value:0.2; Pearson R:−0.25
Zhai Cervix : Cervical squamous
LLGL1
ZBTB7A
P−value:0.13; Pearson R:−0.29
Zhai Cervix : Cervical squamous
LLGL2
ZBTB7A
P−value:0.84; Pearson R:0.041

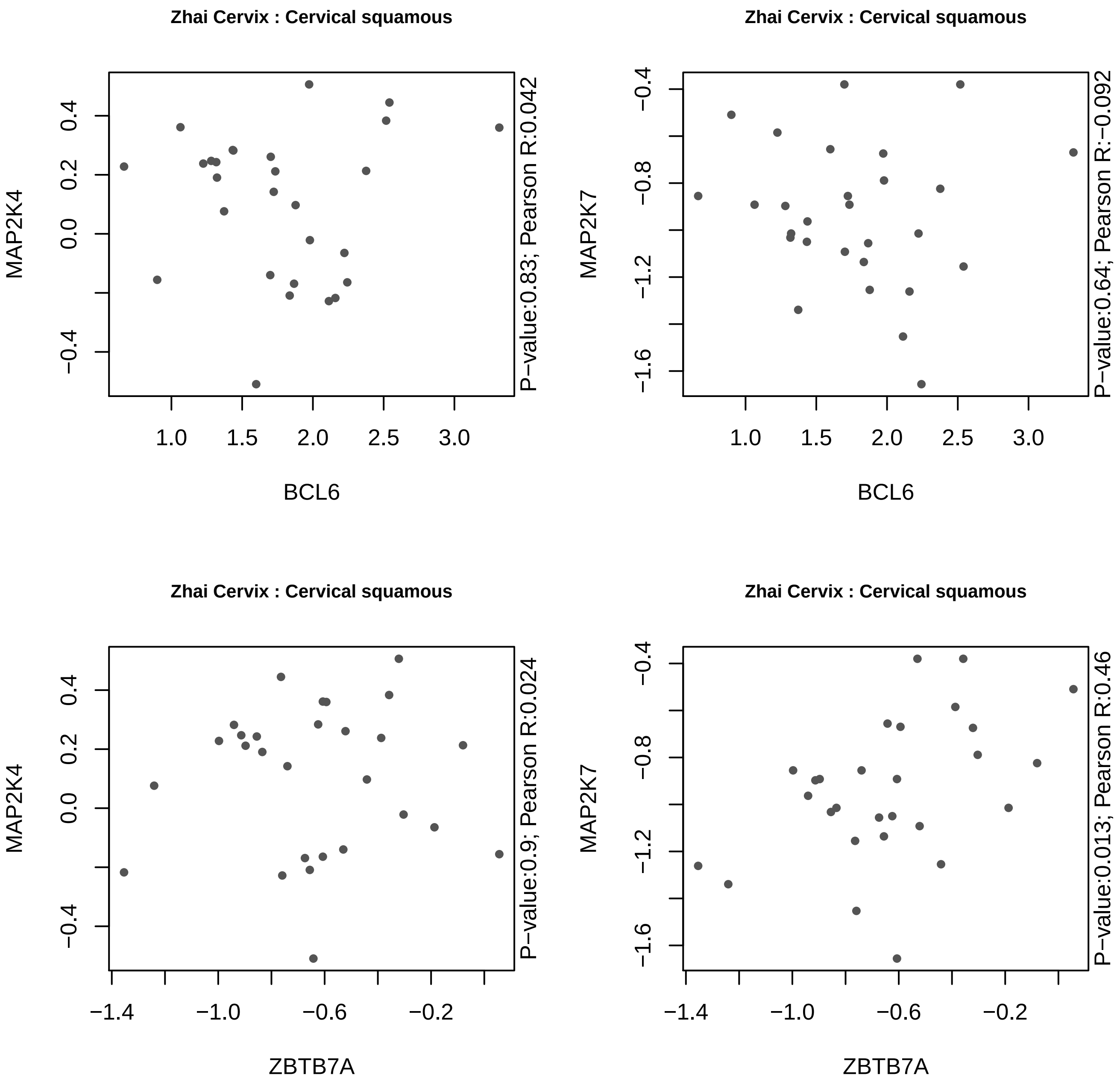
Zhai Cervix : Cervical squamous
MAP2K4
BCL6
P−value:0.83; Pearson R:0.042
Zhai Cervix : Cervical squamous
MAP2K7
BCL6
P−value:0.64; Pearson R:−0.092
Zhai Cervix : Cervical squamous
MAP2K4
ZBTB7A
P−value:0.9; Pearson R:0.024
Zhai Cervix : Cervical squamous
MAP2K7
ZBTB7A
P−value:0.013; Pearson R:0.46

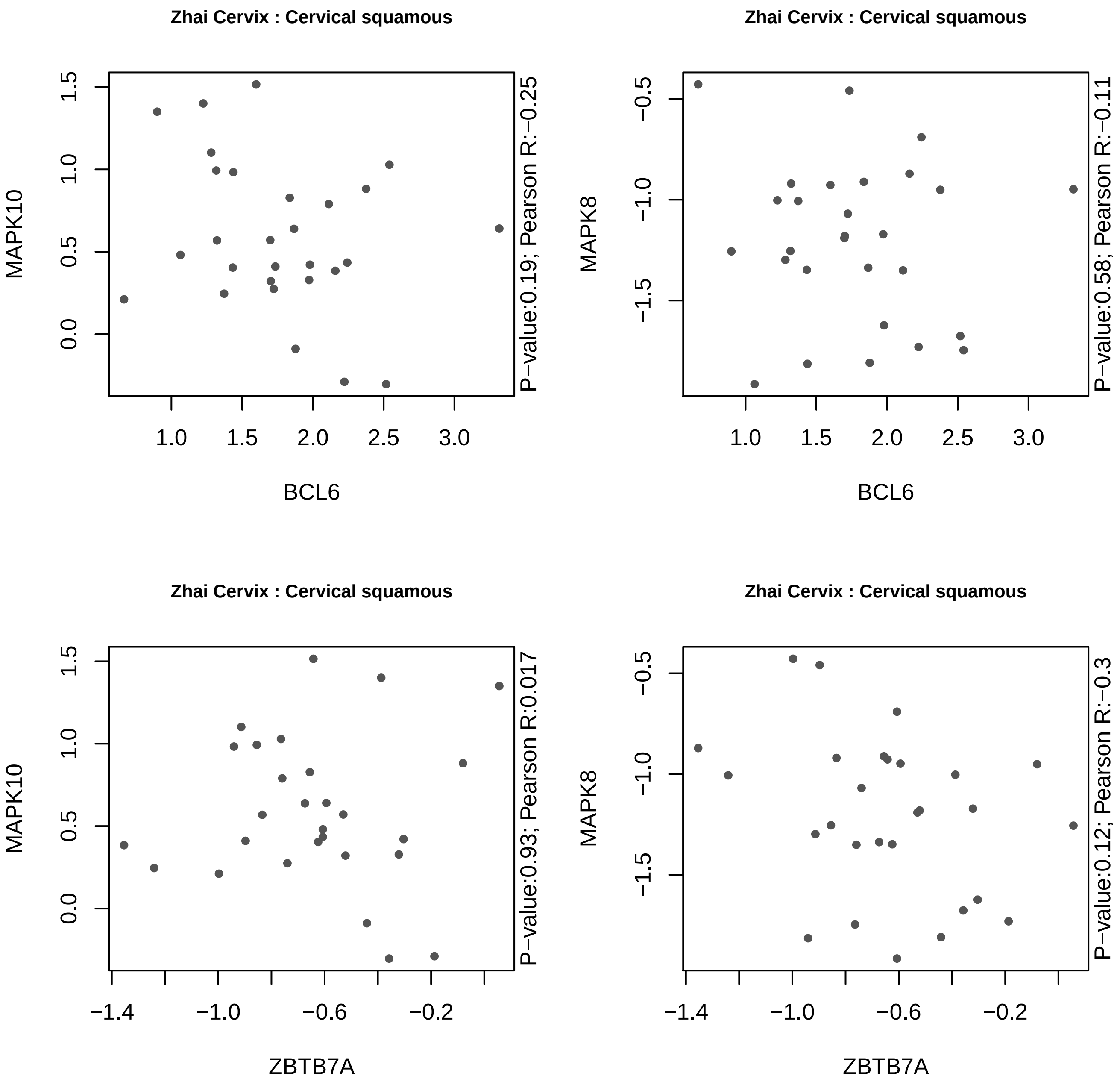
Zhai Cervix : Cervical squamous
MAPK10
BCL6
P−value:0.19; Pearson R:−0.25
Zhai Cervix : Cervical squamous
MAPK8
BCL6
P−value:0.58; Pearson R:−0.11
Zhai Cervix : Cervical squamous
MAPK10
ZBTB7A
P−value:0.93; Pearson R:0.017
Zhai Cervix : Cervical squamous
MAPK8
ZBTB7A
P−value:0.12; Pearson R:−0.3

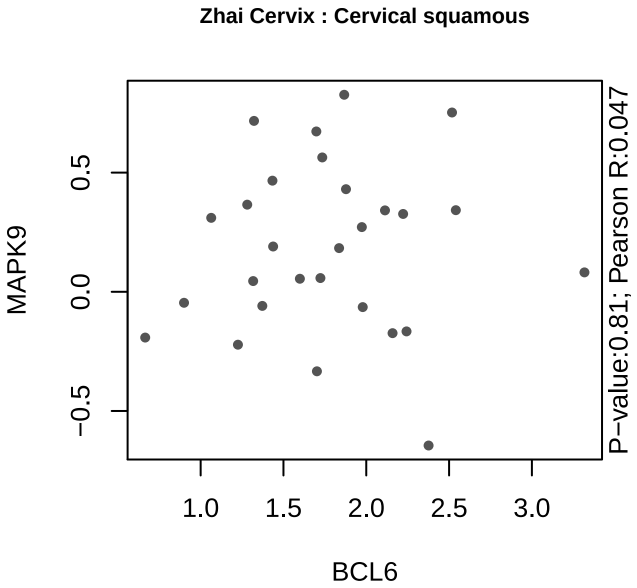
Zhai Cervix : Cervical squamous
MAPK9
BCL6
P−value:0.81; Pearson R:0.047
−0.5
0.0
0.5
1.0
1.5
2.0
2.5
3.0

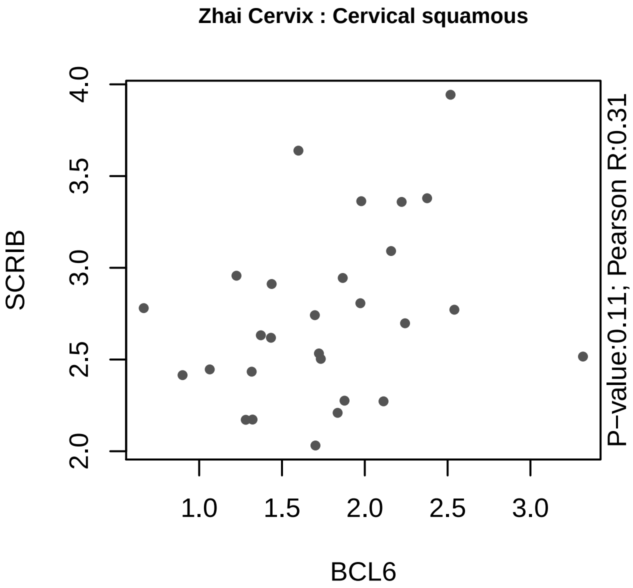
Zhai Cervix : Cervical squamous
SCRIB
BCL6
P−value:0.11; Pearson R:0.31
2.0
2.5
3.0
3.5
4.0
1.0
1.5
2.0
2.5
3.0

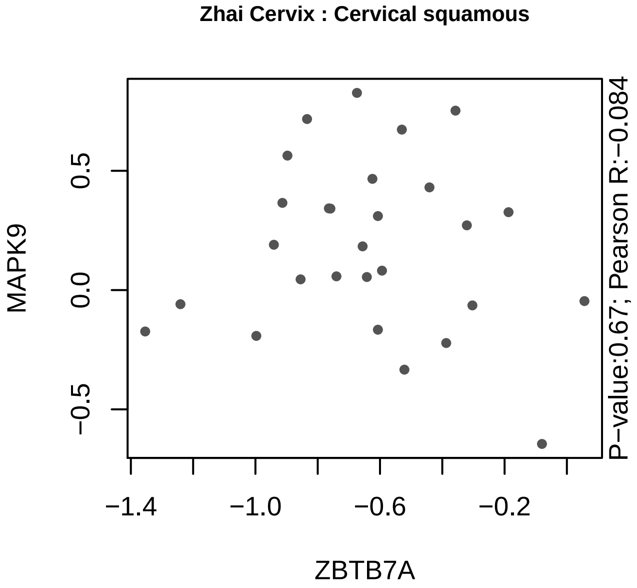
Zhai Cervix : Cervical squamous
MAPK9
ZBTB7A
P−value:0.67; Pearson R:−0.084
−0.5
0.0
0.5
−1.4
−1.0
−0.6
−0.2

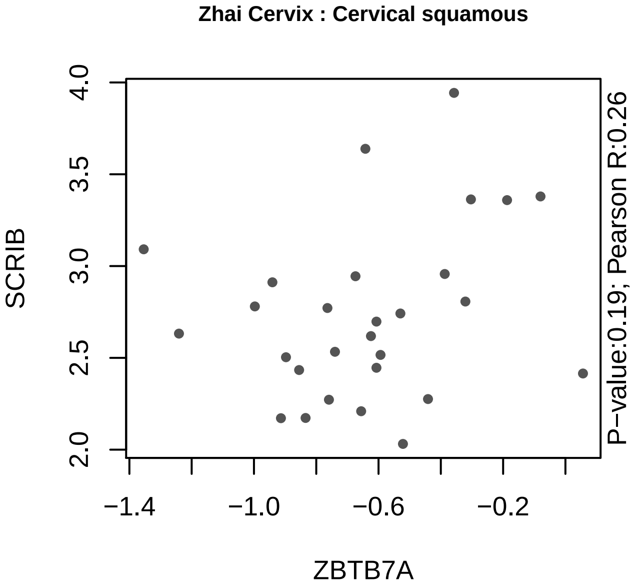
Zhai Cervix : Cervical squamous
SCRIB
ZBTB7A
P−value:0.19; Pearson R:0.26
2.0
2.5
3.0
3.5
4.0
−1.4
−1.0
−0.6
−0.2

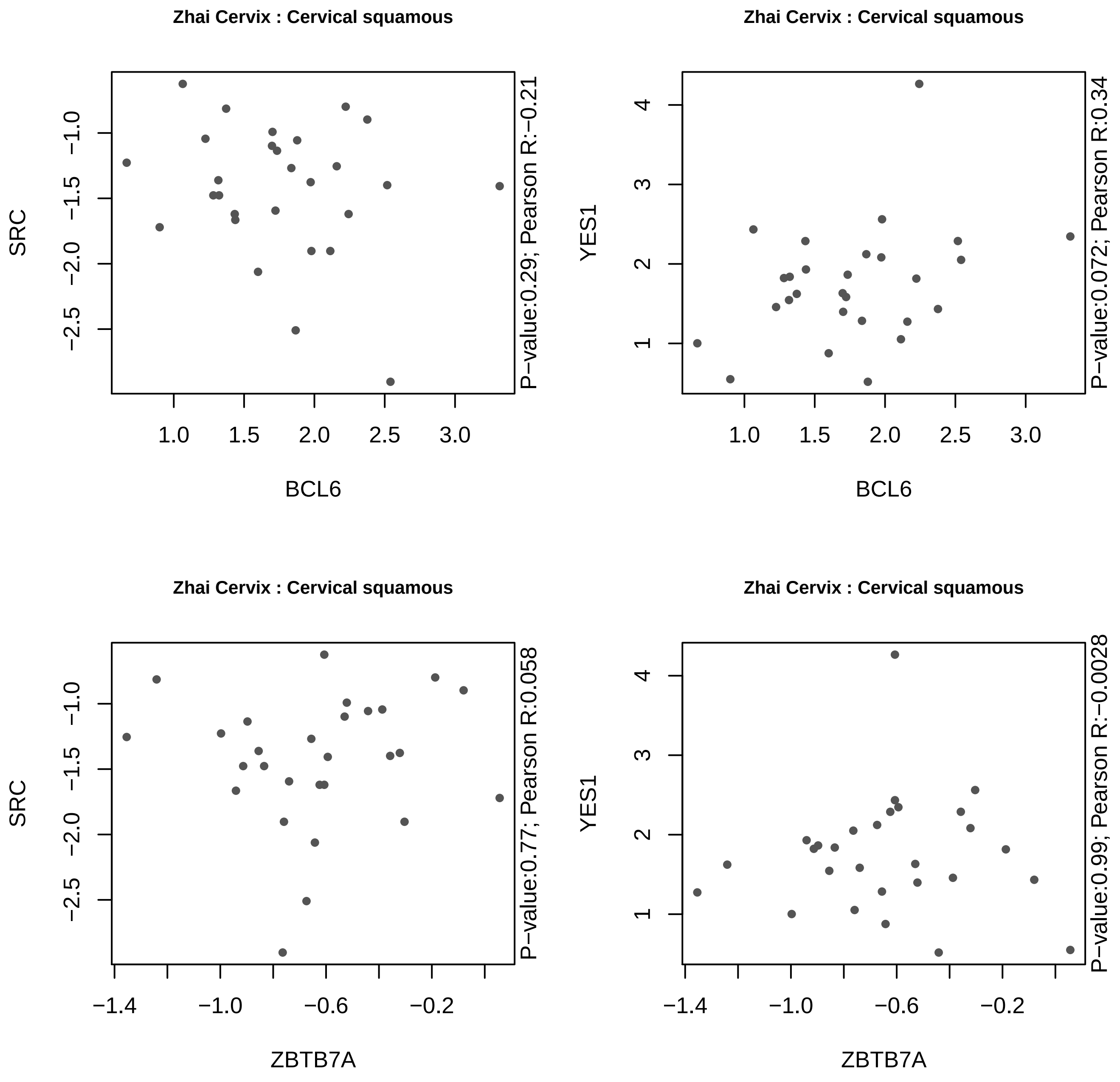
Zhai Cervix : Cervical squamous
SRC
BCL6
P−value:0.29; Pearson R:−0.21
Zhai Cervix : Cervical squamous
YES1
BCL6
P−value:0.072; Pearson R:0.34
Zhai Cervix : Cervical squamous
SRC
ZBTB7A
P−value:0.77; Pearson R:0.058
Zhai Cervix : Cervical squamous
YES1
ZBTB7A
P−value:0.99; Pearson R:−0.0028

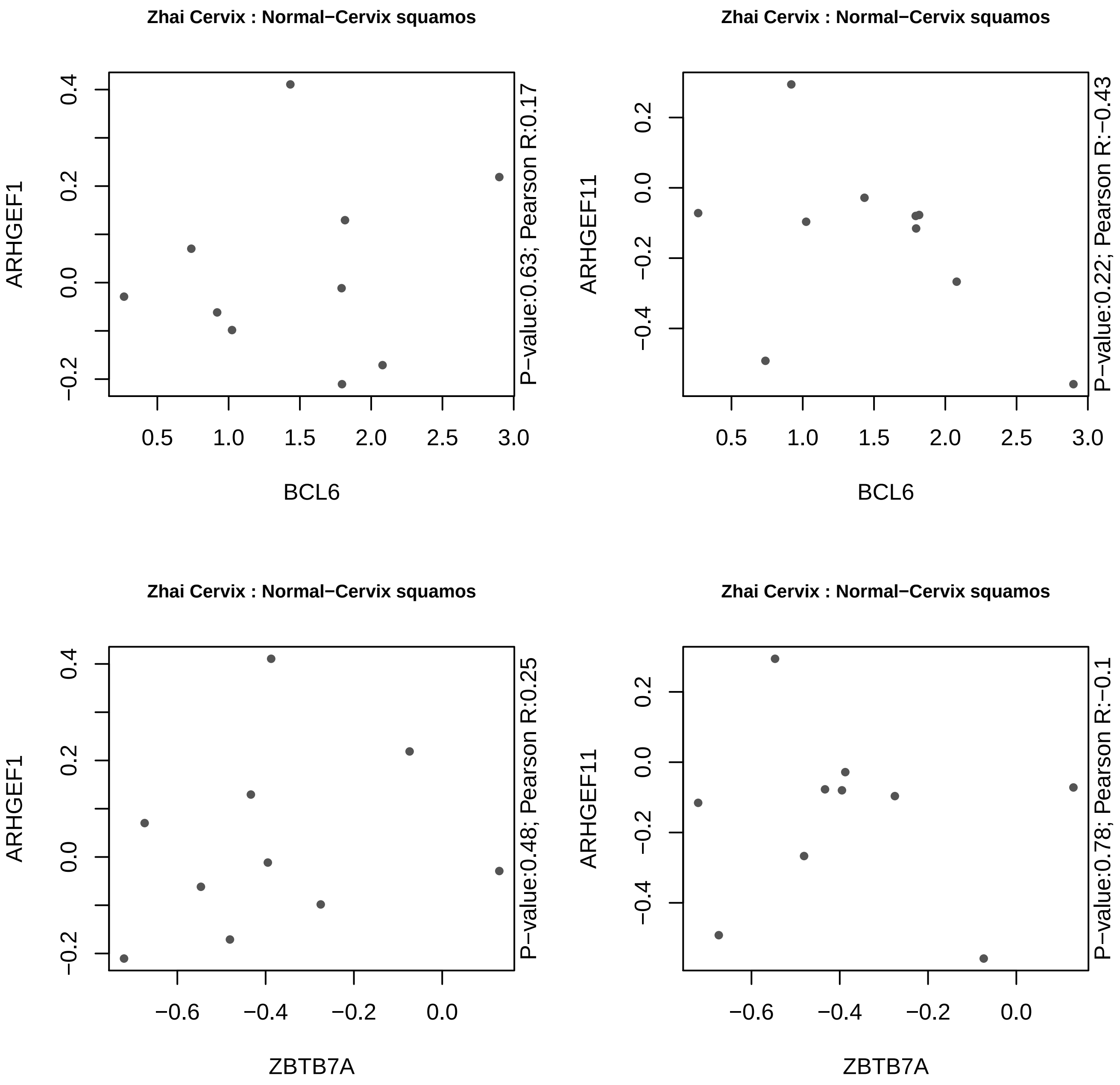
Zhai Cervix : Normal−Cervix squamos
ARHGEF1
BCL6
P−value:0.63; Pearson R:0.17
Zhai Cervix : Normal−Cervix squamos
ARHGEF11
BCL6
P−value:0.22; Pearson R:−0.43
Zhai Cervix : Normal−Cervix squamos
ARHGEF1
ZBTB7A
P−value:0.48; Pearson R:0.25
Zhai Cervix : Normal−Cervix squamos
ARHGEF11
ZBTB7A
P−value:0.78; Pearson R:−0.1

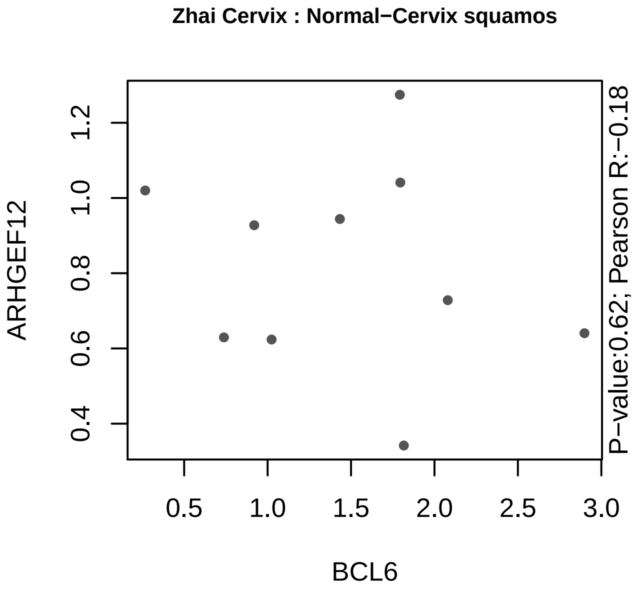
Zhai Cervix : Normal−Cervix squamos
ARHGEF12
BCL6
P−value:0.62; Pearson R:−0.18
0.4
0.6
0.8
1.0
1.2
0.5
1.0
1.5
2.0
2.5
3.0

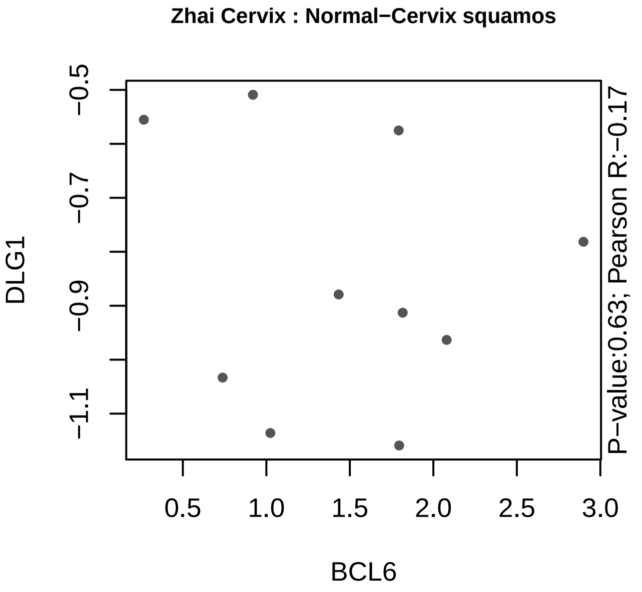
Zhai Cervix : Normal−Cervix squamos
DLG1
BCL6
P−value:0.63; Pearson R:−0.17
−0.5
−0.7
−0.9
−1.1
0.5
1.0
1.5
2.0
2.5
3.0

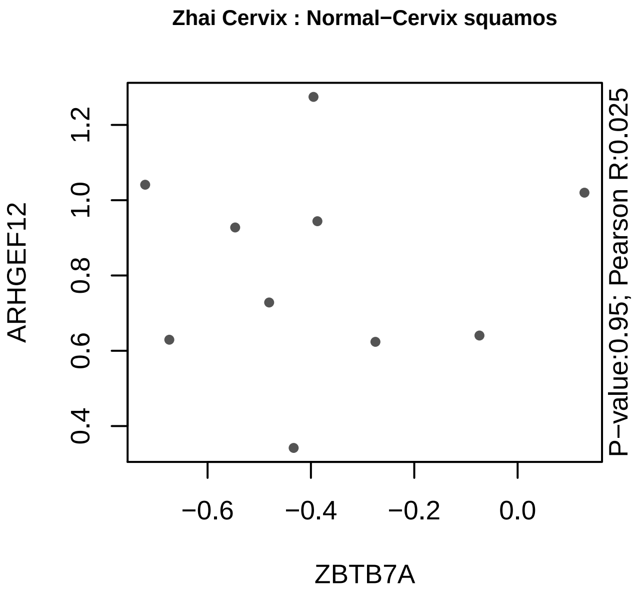
Zhai Cervix : Normal−Cervix squamos
ARHGEF12
ZBTB7A
P−value:0.95; Pearson R:0.025
0.4
0.6
0.8
1.0
1.2
−0.6
−0.4
−0.2
0.0

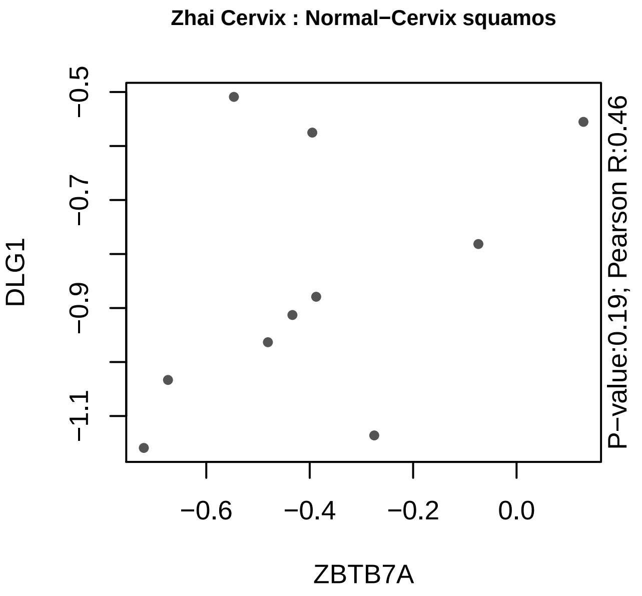
Zhai Cervix : Normal−Cervix squamos
DLG1
ZBTB7A
P−value:0.19; Pearson R:0.46
−0.5
−0.7
−0.9
−1.1
−0.6
−0.4
−0.2
0.0

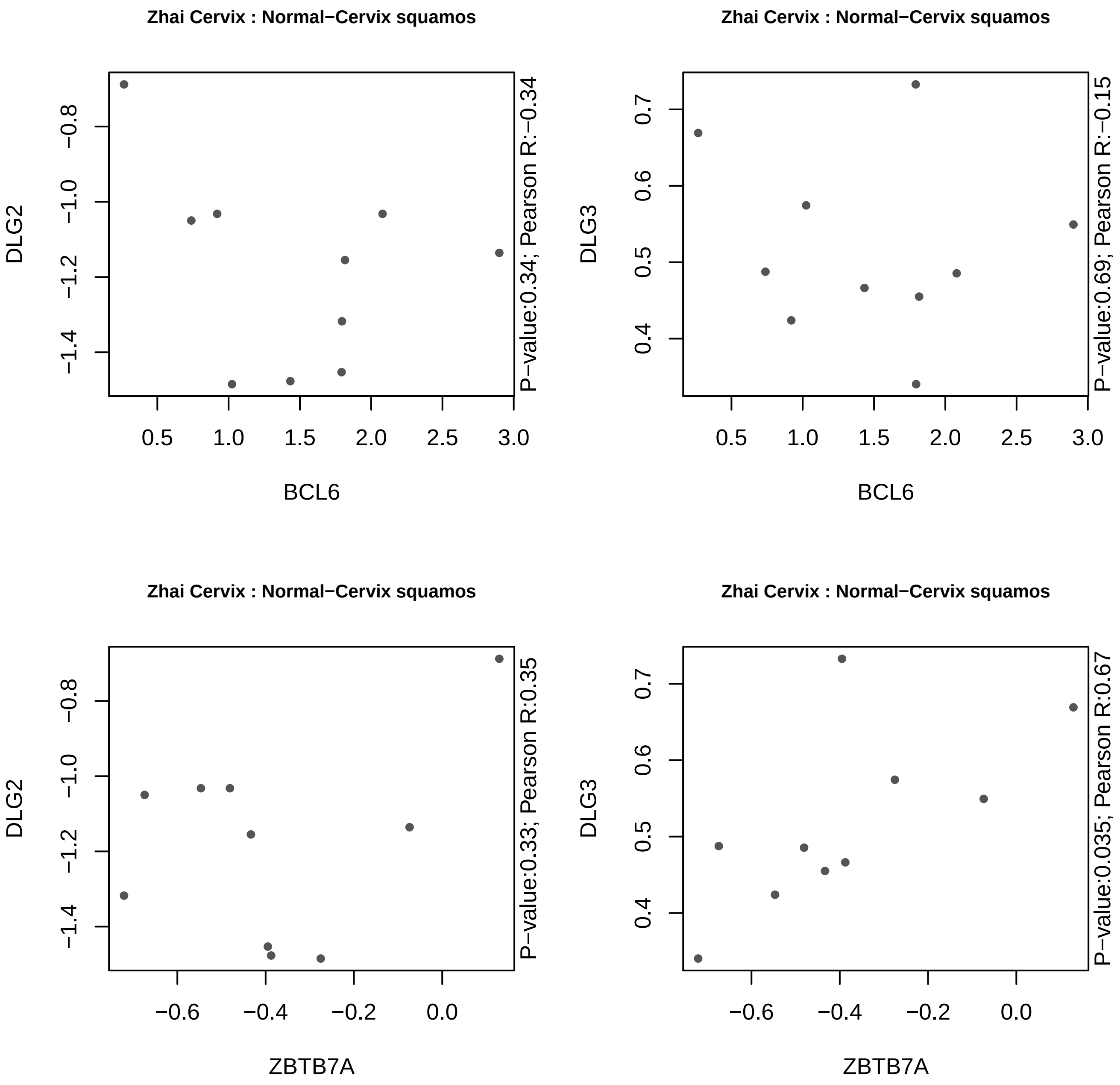
Zhai Cervix : Normal−Cervix squamos
DLG2
BCL6
P−value:0.34; Pearson R:−0.34
Zhai Cervix : Normal−Cervix squamos
DLG3
BCL6
P−value:0.69; Pearson R:−0.15
Zhai Cervix : Normal−Cervix squamos
DLG2
ZBTB7A
P−value:0.33; Pearson R:0.35
Zhai Cervix : Normal−Cervix squamos
DLG3
ZBTB7A
P−value:0.035; Pearson R:0.67

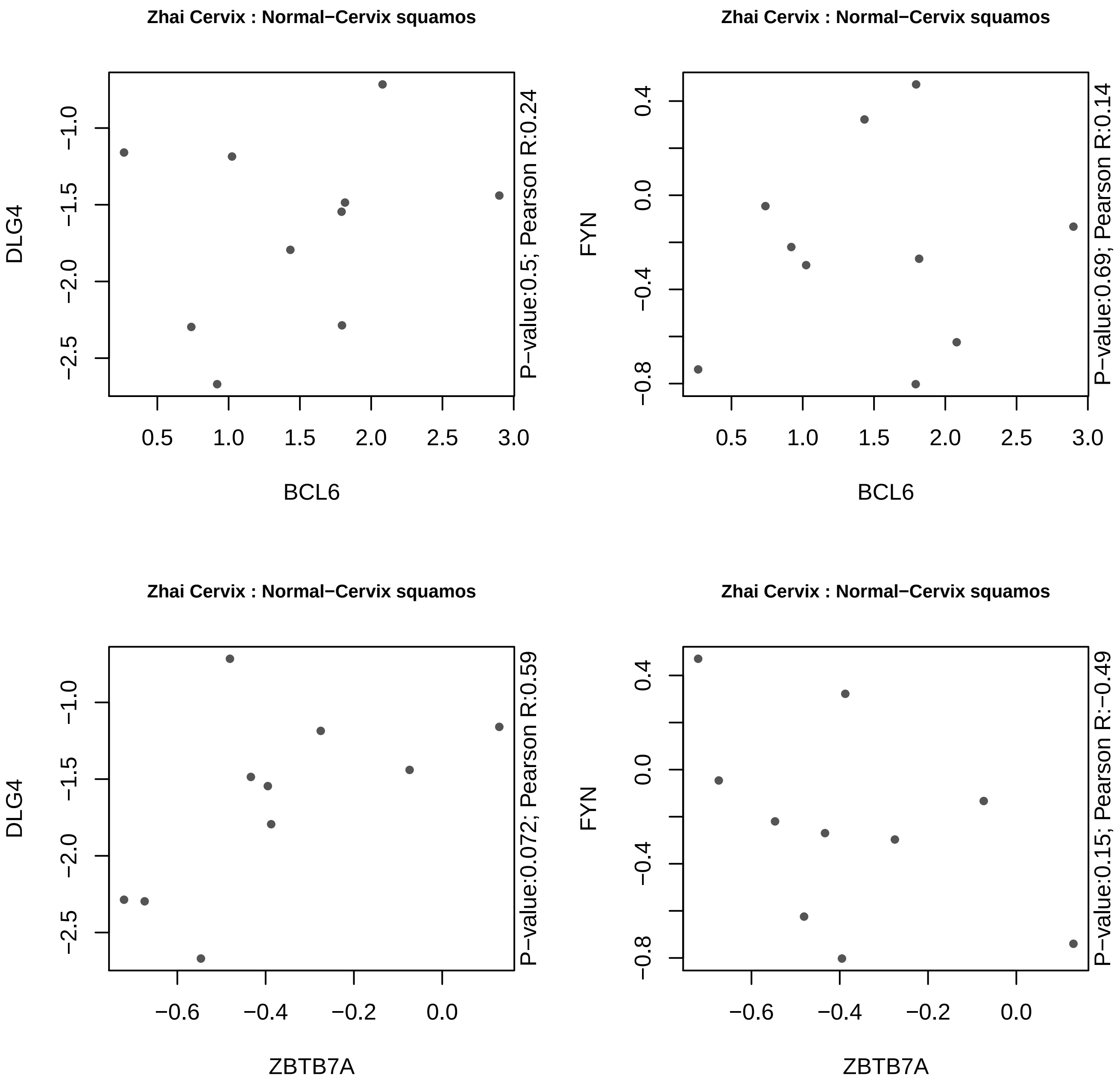
Zhai Cervix : Normal−Cervix squamos
DLG4
BCL6
P−value:0.5; Pearson R:0.24
Zhai Cervix : Normal−Cervix squamos
FYN
BCL6
P−value:0.69; Pearson R:0.14
Zhai Cervix : Normal−Cervix squamos
DLG4
ZBTB7A
P−value:0.072; Pearson R:0.59
Zhai Cervix : Normal−Cervix squamos
FYN
ZBTB7A
P−value:0.15; Pearson R:−0.49

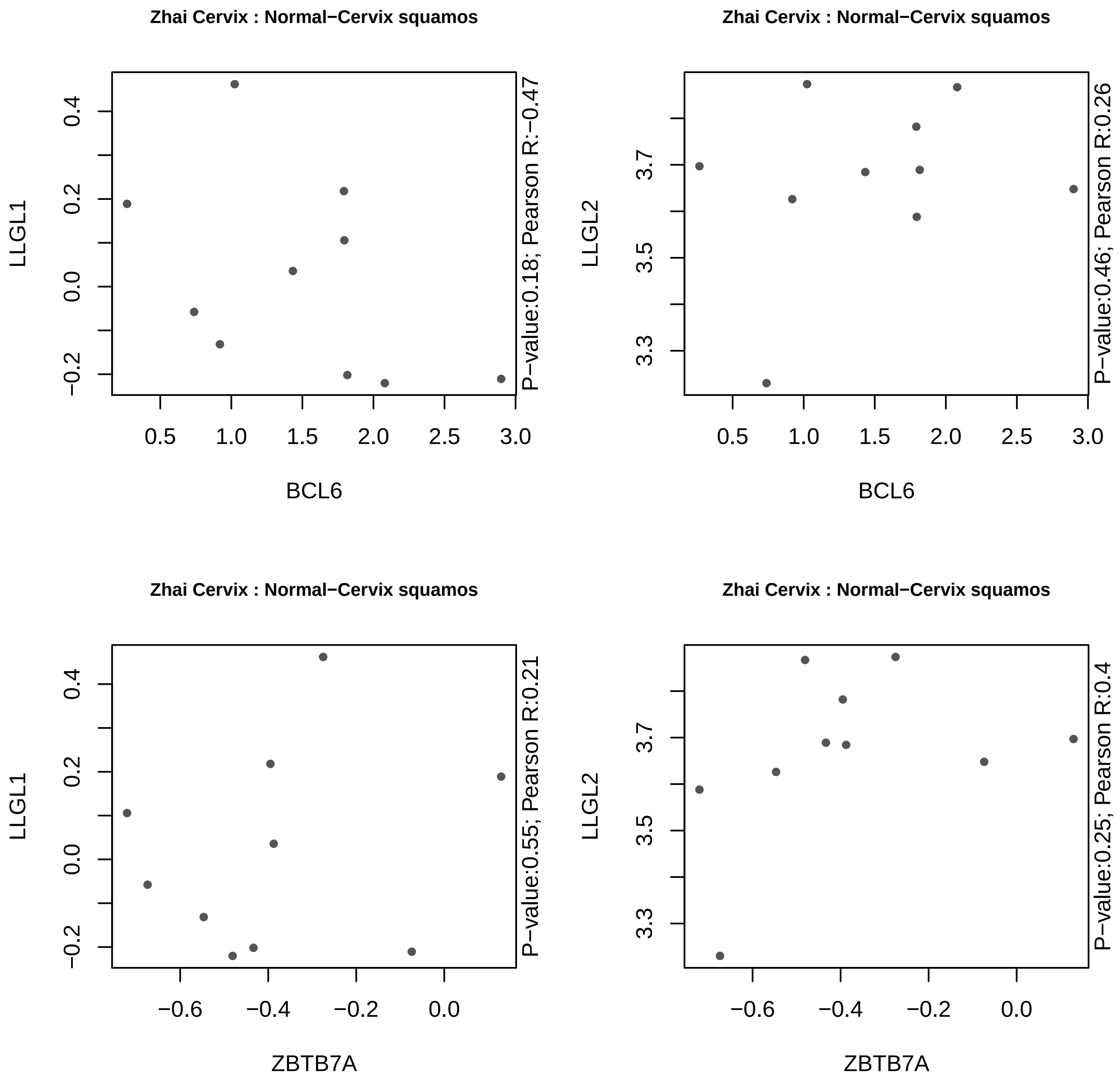
Zhai Cervix : Normal−Cervix squamos
LLGL1
BCL6
P−value:0.18; Pearson R:−0.47
Zhai Cervix : Normal−Cervix squamos
LLGL2
BCL6
P−value:0.46; Pearson R:0.26
Zhai Cervix : Normal−Cervix squamos
LLGL1
ZBTB7A
P−value:0.55; Pearson R:0.21
Zhai Cervix : Normal−Cervix squamos
LLGL2
ZBTB7A
P−value:0.25; Pearson R:0.4

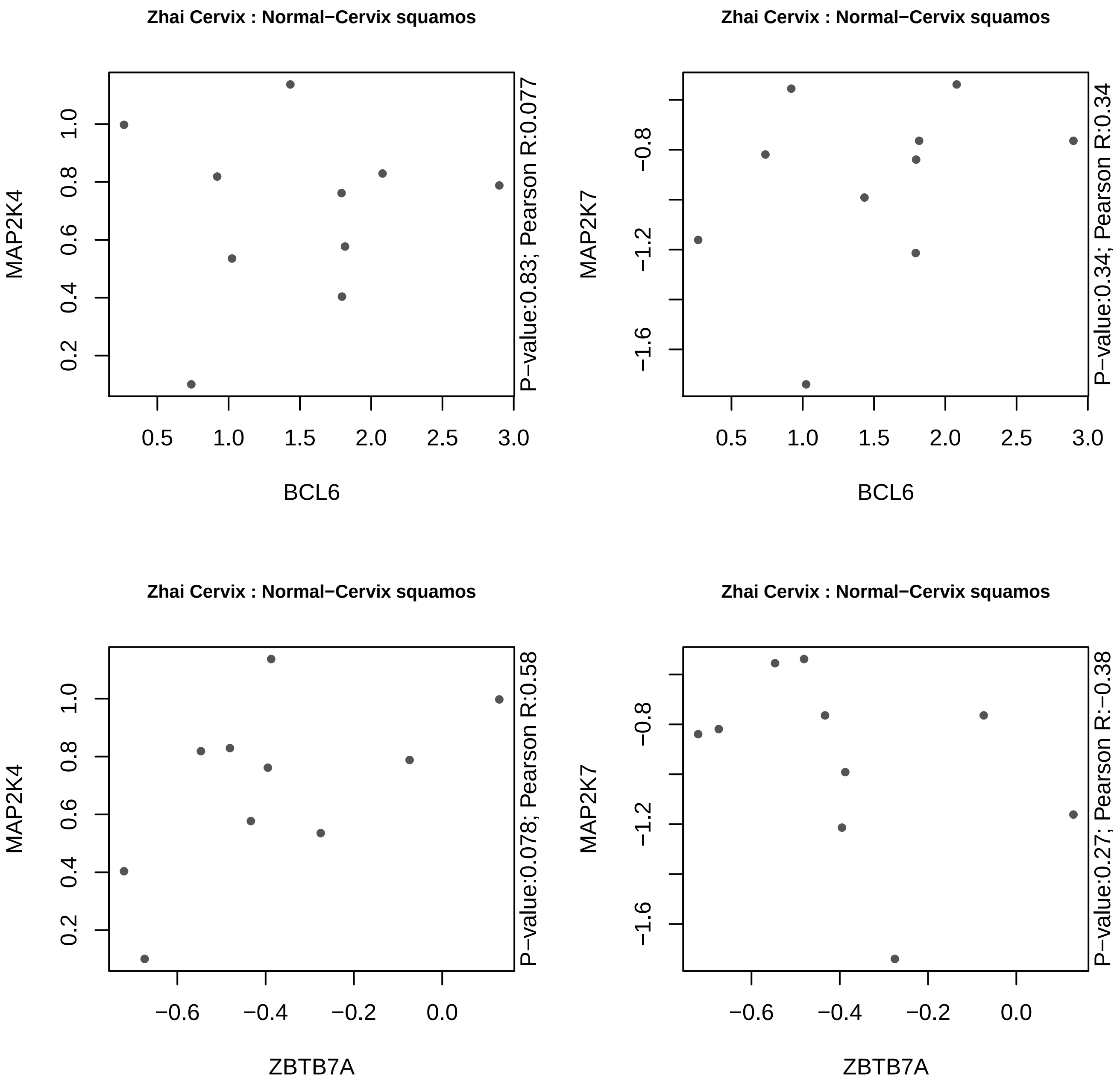
Zhai Cervix : Normal−Cervix squamos
MAP2K4
BCL6
P−value:0.83; Pearson R:0.077
Zhai Cervix : Normal−Cervix squamos
MAP2K7
BCL6
P−value:0.34; Pearson R:0.34
Zhai Cervix : Normal−Cervix squamos
MAP2K4
ZBTB7A
P−value:0.078; Pearson R:0.58
Zhai Cervix : Normal−Cervix squamos
MAP2K7
ZBTB7A
P−value:0.27; Pearson R:−0.38

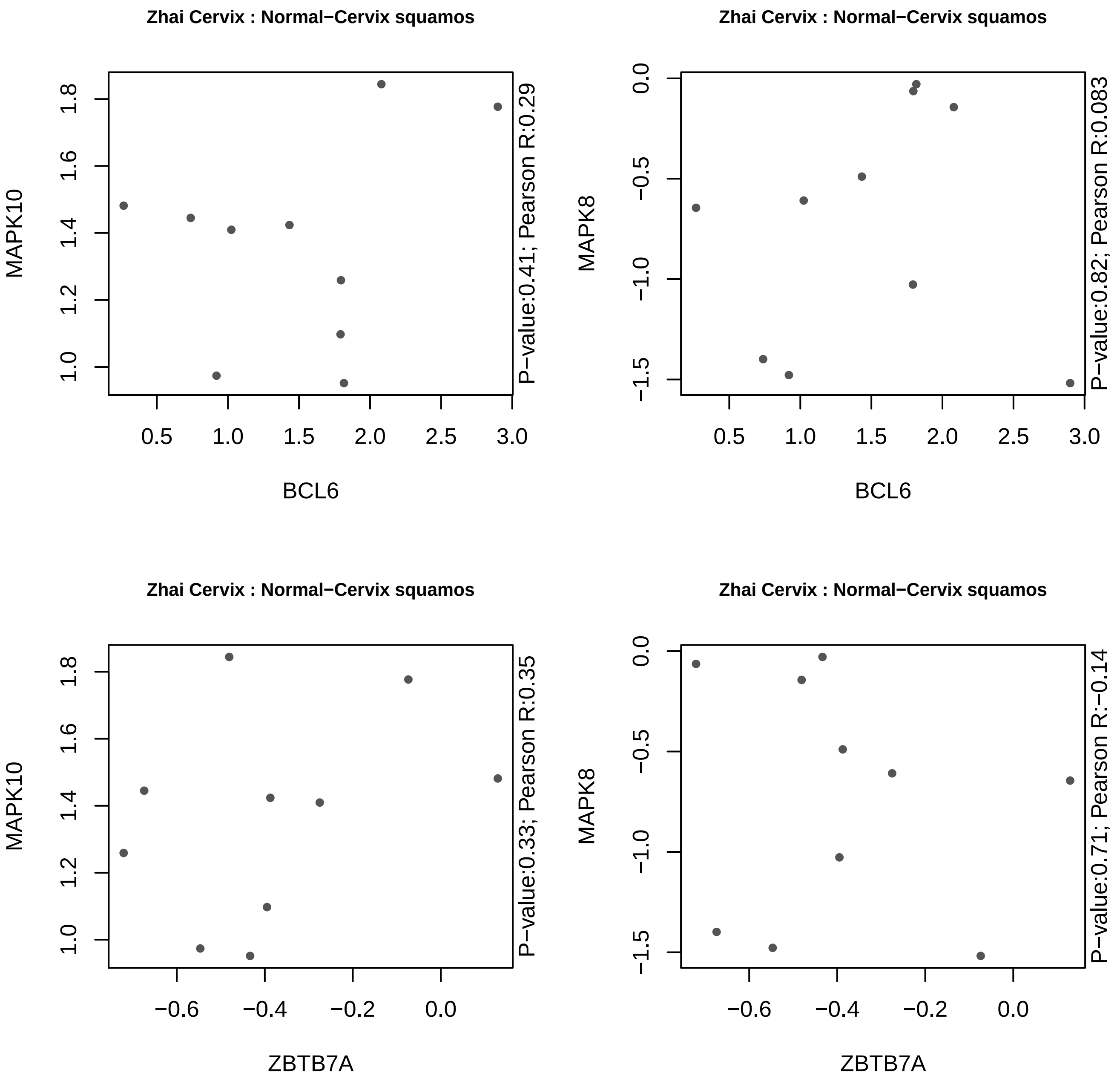
Zhai Cervix : Normal−Cervix squamos
MAPK10
BCL6
P−value:0.41; Pearson R:0.29
Zhai Cervix : Normal−Cervix squamos
MAPK8
BCL6
P−value:0.82; Pearson R:0.083
Zhai Cervix : Normal−Cervix squamos
MAPK10
ZBTB7A
P−value:0.33; Pearson R:0.35
Zhai Cervix : Normal−Cervix squamos
MAPK8
ZBTB7A
P−value:0.71; Pearson R:−0.14

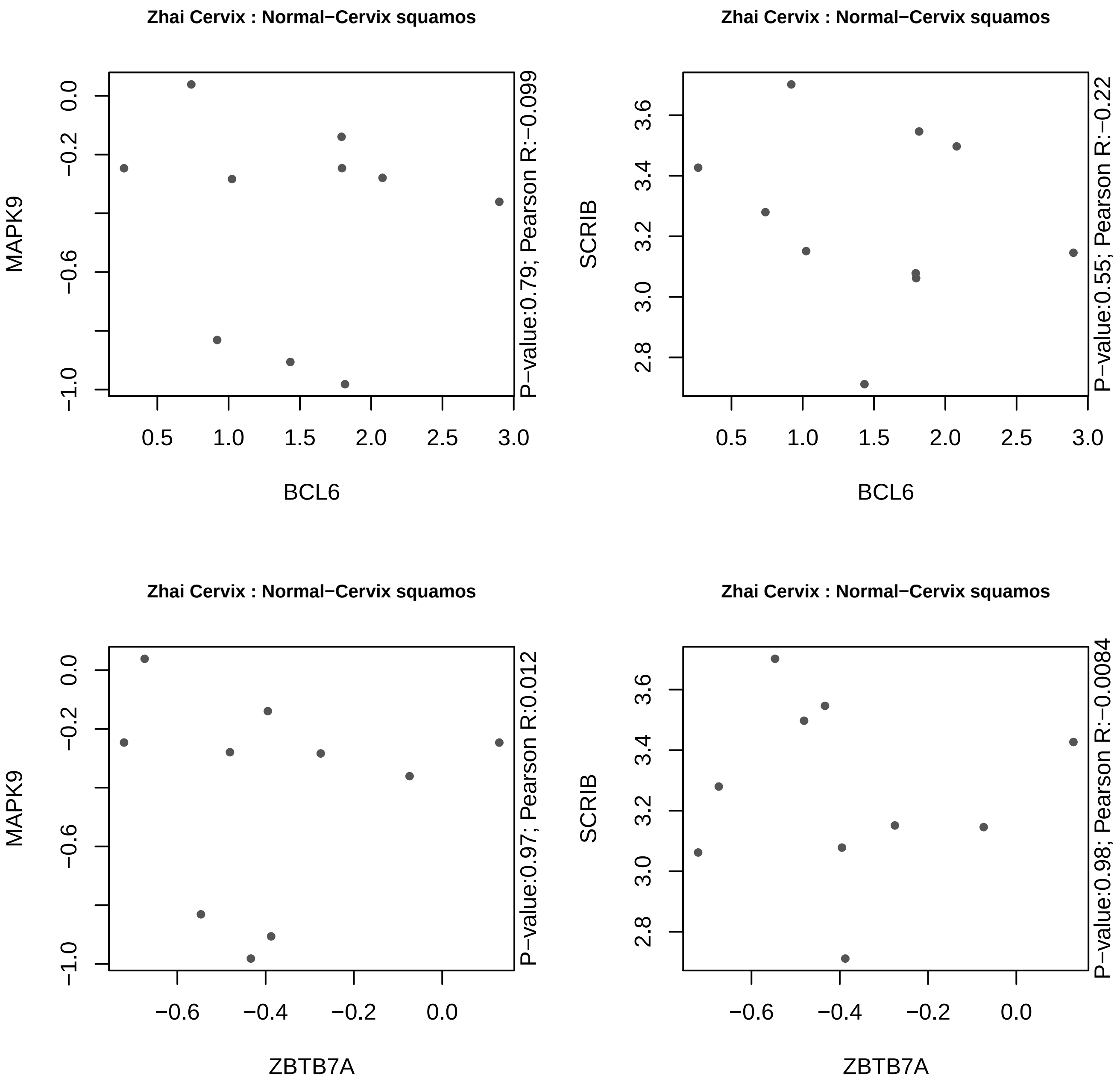
Zhai Cervix : Normal−Cervix squamos
MAPK9
BCL6
P−value:0.79; Pearson R:−0.099
Zhai Cervix : Normal−Cervix squamos
SCRIB
BCL6
P−value:0.55; Pearson R:−0.22
Zhai Cervix : Normal−Cervix squamos
MAPK9
ZBTB7A
P−value:0.97; Pearson R:0.012
Zhai Cervix : Normal−Cervix squamos
SCRIB
ZBTB7A
P−value:0.98; Pearson R:−0.0084

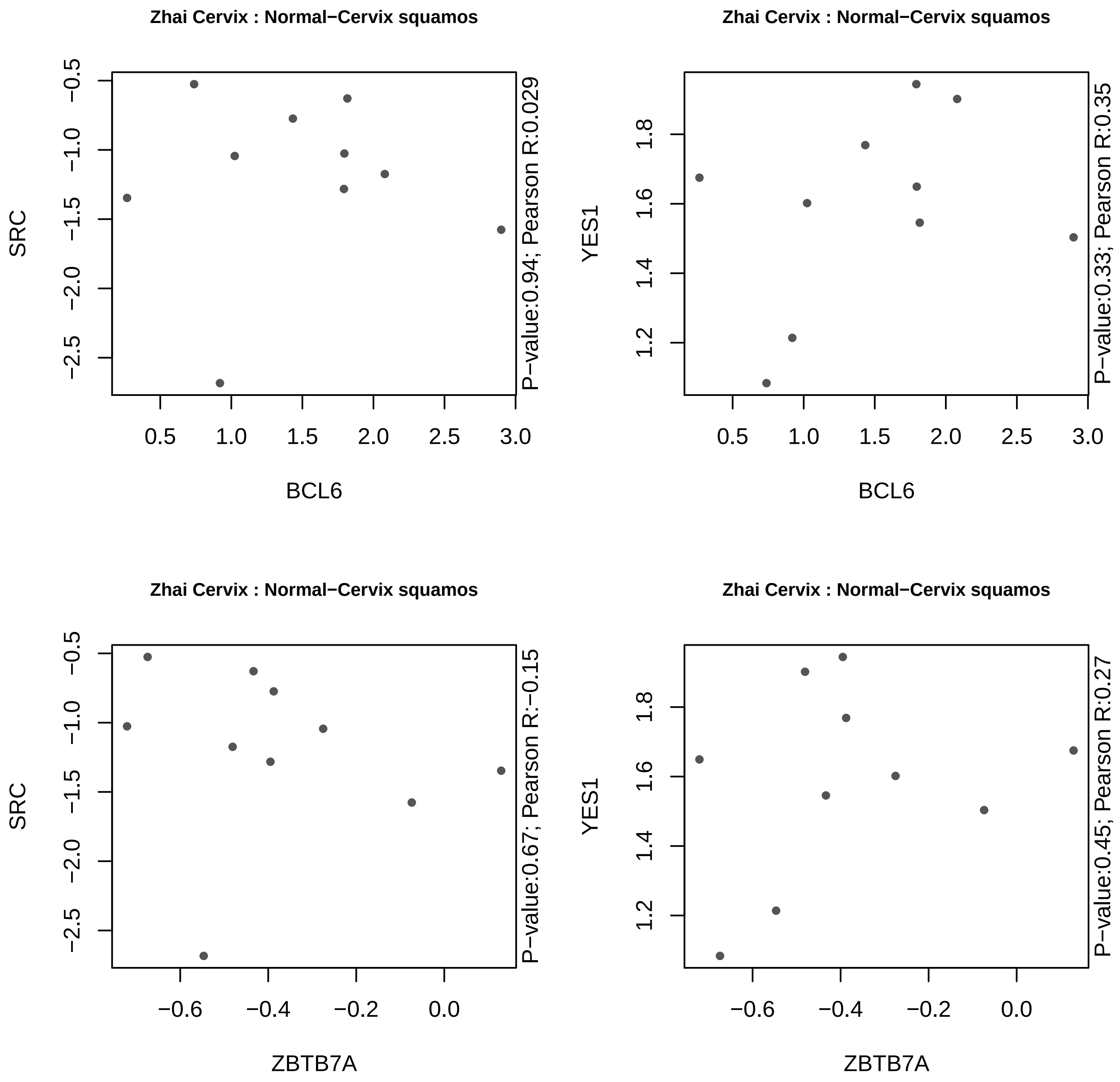
Zhai Cervix : Normal−Cervix squamos
SRC
BCL6
P−value:0.94; Pearson R:0.029
Zhai Cervix : Normal−Cervix squamos
YES1
BCL6
P−value:0.33; Pearson R:0.35
Zhai Cervix : Normal−Cervix squamos
SRC
ZBTB7A
P−value:0.67; Pearson R:−0.15
Zhai Cervix : Normal−Cervix squamos
YES1
ZBTB7A
P−value:0.45; Pearson R:0.27

**Supplementary Table 1:**
**All significant correlations in gene expression**

| Gene expression datasets | BTB-Zn gene | Polarity or cytoskeletal gene | P value | Pearson R | Notes |
|---|---|---|---|---|---|
| Gasper colon all | Bcl6 | ARHGEF1 | 1,00E-06 | -0,52 | A |
| Gaspar colorectal adenoma | Bcl6 | ARHGEF1 | 1,10E-05 | -0,55 | |
| Gaspar colon normal | Bcl6 | ARHGEF1 | 1,60E-02 | -0,51 | N |
| Rohrbeck all-Lung (cancer only) | Bcl6 | ARHGEF1 | 3,30E-02 | -0,33 | A |
| Boersma Breast all | ZBTB7A | ARHGEF1 | 1,00E-02 | 0,26 | A |
| Tomlins Prostate all | Bcl6 | ARHGEF11 | 5,60E-03 | 0,45 | A |
| Tomlins Prostate Carcinoma | Bcl6 | ARHGEF11 | 1,40E-02 | 0,57 | *, # |
| Zhai Cervix all | Bcl6 | ARHGEF11 | 2,20E-02 | -0,37 | A |
| Grutzmann Pancreas normal | Bcl6 | ARHGEF11 | 3,70E-02 | -0,63 | N |
| Grutzmann Pancreas all (no met) | Bcl6 | ARHGEF11 | 4,50E-02 | -0,43 | A |
| Boersma Breast all | ZBTB7A | ARHGEF11 | 2,80E-04 | -0,37 | A |
| Grutzmann Pancreas normal | Bcl6 | ARHGEF12 | 4,90E-02 | -0,6 | N |
| Ma Breast 4 all | Bcl6 | ARHGEF12 | 2,40E-02 | 0,28 | A |
| Toruner Head-Neck all oral squamous carcinoma (cancer only) | ZBTB7A | ARHGEF12 | 1,70E-02 | 0,59 | * |
| Grutzmann Pancreatic ductal adenocarcinoma | ZBTB7A | ARHGEF12 | 2,20E-02 | -0,68 | |
| Boersma Breast all | ZBTB7A | ARHGEF12 | 5,90E-10 | 0,58 | A |
| Rohrbeck all-Lung (cancer only) | Bcl6 | DLG1 | 1,10E-03 | 0,49 | |
| Zhai Cervix all | Bcl6 | DLG1 | 2,10E-02 | 0,37 | A |
| Zhai Cervix Cervical squamous | Bcl6 | DLG1 | 6,00E-03 | 0,51 | |
| Collisson Pancreas all-adenocarcinoma | Bcl6 | DLG1 | 4,60E-02 | 0,39 | |
| Rohrbeck all-Lung (cancer only) | ZBTB7A | DLG1 | 4,70E-02 | -0,31 | |
| Tomlins Prostate all | ZBTB7A | DLG1 | 3,70E-02 | 0,74 | A |
| Grutzmann Pancreas normal | ZBTB7A | DLG1 | 1,60E-02 | 0,7 | N |
| Grutzmann Pancreas all (no met) | ZBTB7A | DLG1 | 1,30E-02 | 0,52 | A |
| Gasper colon all | Bcl6 | DLG2 | 2,20E-15 | -0,75 | A |
| Gaspar colorectal adenoma | Bcl6 | DLG2 | 7,40E-13 | -0,79 | *, # |
| Gaspar colon normal | Bcl6 | DLG2 | 8,40E-04 | -0,66 | N |
| Rohrbeck all-Lung (cancer only) | Bcl6 | DLG2 | 2,00E-02 | -0,36 | * |
| Zhai Cervix Cervical squamous | Bcl6 | DLG2 | 4,90E-02 | 0,38 | |
| Boersma Breast all | Bcl6 | DLG2 | 1,20E-02 | 0,26 | A |
| Boersma Breast all | ZBTB7A | DLG2 | 7,90E-03 | -0,27 | A |
| Skrzypczak Colorectal 2 colon adenoma | Bcl6 | DLG3 | 2,40E-02 | -0,7 | |
| Zhai Cervix Normal-Cervix squamous | ZBTB7A | DLG3 | 3,50E-02 | 0,67 | N |
| Grutzmann Pancreas all (no met) | ZBTB7A | DLG3 | 1,90E-02 | -0,5 | A |
| Boersma Breast all | ZBTB7A | DLG3 | 5,10E-05 | -0,4 | A |
| Ma Breast 4 normal | ZBTB7A | DLG3 | 5,10E-04 | 0,61 | N |
| Tomlins Prostate all | Bcl6 | DLG4 | 8,60E-05 | 0,69 | A |
| Toruner Head-Neck all oral squamous carcinoma (cancer only) | ZBTB7A | DLG4 | 5,70E-02 | 0,48 | |

| | | | | | |
|---|---|---|---|---|---|
| Grutzmann Pancreas normal | ZBTB7A | DLG4 | 2,30E-02 | 0,67 | N |
| Grutzmann Pancreas all (no met) | ZBTB7A | DLG4 | 2,20E-02 | 0,49 | A |
| Gasper colon all | Bcl6 | FYN | 1,70E-06 | -0,51 | A |
| Gaspar colorectal adenoma | Bcl6 | FYN | 8,30E-07 | -0,6 | |
| Skrzypczak Colorectal 2 colon adenoma | Bcl6 | FYN | 6,90E-03 | -0,79 | |
| Tomlins Prostate all | ZBTB7A | FYN | 6,60E-03 | 0,6 | A |
| Tomlins Prostate Normal-Prostate Gland | ZBTB7A | FYN | 9,10E-03 | 0,84 | N |
| Boersma Breast all | ZBTB7A | FYN | 2,50E-03 | 0,31 | A |
| Gasper colon all | Bcl6 | LLGL1 | 4,40E-15 | 0,75 | A |
| Gaspar colorectal adenoma | Bcl6 | LLGL1 | 2,60E-11 | 0,75 | |
| Gaspar colon normal | Bcl6 | LLGL1 | 4,60E-05 | 0,76 | N |
| Rohrbeck all-Lung (cancer only) | Bcl6 | LLGL1 | 3,80E-03 | -0,44 | * |
| Zhai Cervix all | Bcl6 | LLGL1 | 3,50E-02 | -0,34 | A |
| Ma Breast 4 all | Bcl6 | LLGL1 | 1,30E-03 | 0,39 | A |
| Ma Breast 4 normal | Bcl6 | LLGL1 | 1,80E-02 | 0,44 | N |
| Ma Breast 4 primary ductal breast carcinoma | Bcl6 | LLGL1 | 3,90E-02 | 0,34 | |
| Ma Breast 2 all breast carcinoma | ZBTB7A | LLGL1 | 3,20E-02 | 0,34 | |
| Gasper colon all | Bcl6 | LLGL2 | 0,036 | 0,24 | A |
| Gaspar colorectal adenoma | Bcl6 | LLGL2 | 9,00E-04 | 0,43 | |
| Skrzypczak Colorectal 2-all Epithelia | Bcl6 | LLGL2 | 3,70E-02 | -0,47 | A |
| Skrzypczak Colorectal 2-all Epithelia | ZBTB7A | LLGL2 | 1,80E-02 | 0,52 | A |
| Boersma Breast all | ZBTB7A | LLGL2 | 1,20E-04 | -0,38 | A |
| Ma Breast 4 normal | ZBTB7A | LLGL2 | 1,90E-02 | 0,44 | N |
| Toruner Head-Neck all oral squamous carcinoma (cancer only) | Bcl6 | MAPK10 | 1,10E-02 | 0,62 | * |
| Boersma Breast normal | Bcl6 | MAPK10 | 5,00E-02 | 0,29 | N |
| Tomlins Prostate Carcinoma | ZBTB7A | MAPK10 | 3,40E-03 | -0,85 | |
| Gaspar colon normal | Bcl6 | MAPK8 | 2,50E-02 | -0,48 | N |
| Tomlins Prostate all | Bcl6 | MAPK8 | 2,00E-02 | 0,46 | A |
| Tomlins Prostate Carcinoma | Bcl6 | MAPK8 | 2,20E-02 | 0,57 | * |
| Grutzmann Pancreas normal | Bcl6 | MAPK8 | 2,10E-03 | -0,82 | N |
| Grutzmann Pancreas all (no met) | Bcl6 | MAPK8 | 6,30E-03 | -0,56 | A |
| Grutzmann Pancreas all (no met) | ZBTB7A | MAPK8 | 1,10E-02 | 0,53 | A |
| Gasper colon all | Bcl6 | MAPK9 | 3,20E-13 | 0,71 | A |
| Gaspar colorectal adenoma | Bcl6 | MAPK9 | 4,40E-09 | 0,69 | *, # |
| Gaspar colon normal | Bcl6 | MAPK9 | 3,10E-06 | 0,82 | N |
| Collisson Pancreas all-adenocarcinoma | Bcl6 | MAPK9 | 8,00E-03 | -0,5 | |
| Grutzmann Pancreas normal | Bcl6 | MAPK9 | 1,50E-02 | 0,71 | N |
| Ma Breast 4 normal | Bcl6 | MAPK9 | 6,30E-03 | -0,5 | N |
| Skrzypczak Colorectal 2-all Epithelia | ZBTB7A | MAPK9 | 1,10E-02 | 0,55 | A |
| Boersma Breast all | ZBTB7A | MAPK9 | 9,90E-08 | -0,51 | A |
| Boersma Breast normal | ZBTB7A | MAPK9 | 1,10E-02 | -0,37 | N |
| Gaspar colorectal adenoma | Bcl6 | MAP2K4 | 6,80E-03 | 0,36 | *, # |
| Grutzmann Pancreas all (no met) | Bcl6 | MAP2K4 | 8,50E-03 | 0,55 | A |
| Grutzmann Pancreas normal | Bcl6 | MAP2K4 | 4,00E-03 | 0,79 | N |
| Ma Breast 4 all | Bcl6 | MAP2K4 | 3,40E-03 | -0,36 | A |

| | | | | | |
|---|---|---|---|---|---|
| Ma Breast 4 normal | Bcl6 | MAP2K4 | 1,20E-03 | -0,58 | N |
| Rohrbeck all-Lung (cancer only) | Bcl6 | MAP2K4 | 2,50E-03 | 0,45 | * |
| Zhai cervix all | ZBTB7A | MAP2K4 | 3,20E-02 | 0,35 | A |
| Boersma Breast epithelial cancer | Bcl6 | MAP2K7 | 2,30E-03 | 0,43 | *, # |
| Gaspar colorectal adenoma | Bcl6 | MAP2K7 | 2,20E-02 | -0,31 | |
| Rohrbeck all-Lung (cancer only) | Bcl6 | MAP2K7 | 6,20E-04 | -0,51 | |
| Grutzmann Pancreas all (no met) | Bcl6 | MAP2K7 | 2,40E-03 | -0,61 | A |
| Grutzmann Pancreas normal | Bcl6 | MAP2K7 | 9,90E-03 | -0,74 | N |
| Grutzmann Pancreatic ductal adenocarcinoma | ZBTB7A | MAP2K7 | 5,10E-02 | 0,6 | *, # |
| Grutzmann Pancreas all (no met) | ZBTB7A | MAP2K7 | 1,20E-02 | 0,52 | A |
| Zhai Cervix Cervical squamous | ZBTB7A | MAP2K7 | 1,30E-02 | 0,46 | *, # |
| Zhai Cervix all | ZBTB7A | SCRIB | 3,40E-02 | 0,34 | A |
| Ma Breast 4 all | ZBTB7A | SCRIB | 1,10E-02 | 0,31 | A |
| Ma Breast 4 normal | ZBTB7A | SCRIB | 2,00E-02 | 0,44 | N |
| Gasper colon all | Bcl6 | SRC | 1,70E-10 | -0,65 | A |
| Gaspar colorectal adenoma | Bcl6 | SRC | 5,30E-08 | -0,65 | |
| Gaspar colon normal | Bcl6 | SRC | 1,60E-03 | -0,63 | N |
| Grutzmann Pancreas normal | Bcl6 | SRC | 1,40E-02 | -0,71 | N |
| Grutzmann Pancreas all (no met) | Bcl6 | SRC | 3,00E-02 | -0,46 | A |
| Collisson Pancreas all-adenocarcinoma | ZBTB7A | SRC | 3,10E-02 | 0,42 | * |
| Grutzmann Pancreas normal | ZBTB7A | SRC | 2,70E-03 | 0,81 | N |
| Grutzmann Pancreas all (no met) | ZBTB7A | SRC | 3,10E-04 | 0,7 | A |
| Gasper colon all | Bcl6 | YES1 | 1,60E-05 | 0,47 | A |
| Gaspar colorectal adenoma | Bcl6 | YES1 | 1,40E-04 | 0,49 | *, # |
| Gaspar colon normal | Bcl6 | YES1 | 3,40E-02 | 0,45 | N |
| Rohrbeck all-Lung (cancer only) | Bcl6 | YES1 | 9,60E-03 | 0,4 | * |
| Zhai Cervix all | Bcl6 | YES1 | 4,70E-02 | 0,32 | A |
| Grutzmann Pancreas normal | Bcl6 | YES1 | 5,60E-04 | 0,87 | N |
| Grutzmann Pancreas all (no met) | Bcl6 | YES1 | 8,40E-04 | 0,66 | A |
| Boersma Breast all | Bcl6 | YES1 | 5,00E-03 | -0,29 | A |
| Boersma Breast epithelial cancer | Bcl6 | YES1 | 1,40E-02 | -0,35 | |
| Skrzypczak Colorectal 2-all Epithelia | ZBTB7A | YES1 | 1,60E-02 | 0,53 | A |

Red = positive correlation, Blue = negative correlation, Brown = borderline significance with positive correlation, A = All (normal & cancer), N= Normal, * Bcl6/ZBTB7 high and correlated as expected with the test gene (highlighted in green), # Trend is stronger in cancer compared with normal tissue (highlighted in yellow)
